# The global, regional, and national burden of cirrhosis by cause in 195 countries and territories, 1990–2017: a systematic analysis for the Global Burden of Disease Study 2017

**DOI:** 10.1016/S2468-1253(19)30349-8

**Published:** 2020-01-22

**Authors:** GBD 2017 Cirrhosis Collaborators, Sadaf G Sepanlou, Saeid Safiri, Catherine Bisignano, Kevin S Ikuta, Shahin Merat, Mehdi Saberifiroozi, Hossein Poustchi, Derrick Tsoi, Danny V Colombara, Amir Abdoli, Rufus Adesoji Adedoyin, Mohsen Afarideh, Sutapa Agrawal, Sohail Ahmad, Elham Ahmadian, Ehsan Ahmadpour, Tomi Akinyemiju, Chisom Joyqueenet Akunna, Vahid Alipour, Amir Almasi-Hashiani, Abdulaziz M Almulhim, Rajaa M Al-Raddadi, Nelson Alvis-Guzman, Nahla Hamed Anber, Colin Angus, Amir Anoushiravani, Jalal Arabloo, Ephrem Mebrahtu Araya, Daniel Asmelash, Bahar Ataeinia, Zerihun Ataro, Maha Moh'd Wahbi Atout, Floriane Ausloos, Ashish Awasthi, Alaa Badawi, Maciej Banach, Diana Fernanda Bejarano Ramirez, Akshaya Srikanth Bhagavathula, Neeraj Bhala, Krittika Bhattacharyya, Antonio Biondi, Srinivasa Rao Bolla, Archith Boloor, Antonio M Borzì, Zahid A Butt, Luis LA Alberto Cámera, Ismael R Campos-Nonato, Félix Carvalho, Dinh-Toi Chu, Sheng-Chia Chung, Paolo Angelo Cortesi, Vera M Costa, Benjamin C Cowie, Ahmad Daryani, Barbora de Courten, Gebre Teklemariam Demoz, Rupak Desai, Samath Dhamminda Dharmaratne, Shirin Djalalinia, Hoa Thi Do, Fariba Dorostkar, Thomas M Drake, Manisha Dubey, Bruce B Duncan, Andem Effiong, Aziz Eftekhari, Aisha Elsharkawy, Arash Etemadi, Mohammad Farahmand, Farshad Farzadfar, Eduarda Fernandes, Irina Filip, Florian Fischer, Ketema Bizuwork Bizuwork Gebremedhin, Birhanu Geta, Syed Amir Gilani, Paramjit Singh Gill, Reyna Alma Gutirrez, Michael Tamene Haile, Arvin Haj-Mirzaian, Saeed S Hamid, Milad Hasankhani, Amir Hasanzadeh, Maryam Hashemian, Hamid Yimam Hassen, Simon I Hay, Khezar Hayat, Behnam Heidari, Andualem Henok, Chi Linh Hoang, Mihaela Hostiuc, Sorin Hostiuc, Vivian Chia-rong Hsieh, Ehimario U Igumbor, Olayinka Stephen Ilesanmi, Seyed Sina Naghibi Irvani, Nader Jafari Balalami, Spencer L James, Panniyammakal Jeemon, Ravi Prakash Jha, Jost B Jonas, Jacek Jerzy Jozwiak, Ali Kabir, Amir Kasaeian, Hagazi Gebremedhin Kassaye, Adane Teshome Kefale, Rovshan Khalilov, Muhammad Ali Khan, Ejaz Ahmad Khan, Amir Khater, Yun Jin Kim, Ai Koyanagi, Carlo La Vecchia, Lee-Ling Lim, Alan D Lopez, Stefan Lorkowski, Paulo A. Lotufo, Rafael Lozano, Muhammed Magdy Abd El Razek, Hue Thi Mai, Navid Manafi, Amir Manafi, Mohammad Ali Mansournia, Lorenzo Giovanni Mantovani, Giampiero Mazzaglia, Dhruv Mehta, Walter Mendoza, Ritesh G Menezes, Melkamu Merid Mengesha, Tuomo J Meretoja, Tomislav Mestrovic, Bartosz Miazgowski, Ted R Miller, Erkin M Mirrakhimov, Prasanna Mithra, Babak Moazen, Masoud Moghadaszadeh, Abdollah Mohammadian-Hafshejani, Shafiu Mohammed, Ali H Mokdad, Pablo A Montero-Zamora, Ghobad Moradi, Mukhammad David Naimzada, Vinod Nayak, Ionut Negoi, Trang Huyen Nguyen, Richard Ofori-Asenso, In-Hwan Oh, Tinuke O Olagunju, Jagadish Rao Padubidri, Keyvan Pakshir, Adrian Pana, Mona Pathak, Akram Pourshams, Navid Rabiee, Amir Radfar, Alireza Rafiei, Kiana Ramezanzadeh, Saleem Muhammad M Rana, Salman Rawaf, David Laith Rawaf, Robert C Reiner, Leonardo Roever, Robin Room, Gholamreza Roshandel, Saeed Safari, Abdallah M Samy, Juan Sanabria, Benn Sartorius, Maria Inês Schmidt, Subramanian Senthilkumaran, Masood Ali Shaikh, Mehdi Sharif, Amrollah Sharifi, Mika Shigematsu, Jasvinder A. Singh, Amin Soheili, Hafiz Ansar Rasul Suleria, Berhane Fseha Teklehaimanot, Berhe Etsay Tesfay, Marco Vacante, Amir Vahedian-Azimi, Pascual R Valdez, Tommi Juhani Vasankari, Giang Thu Vu, Yasir Waheed, Kidu Gidey Weldegwergs, Andrea Werdecker, Ronny Westerman, Dawit Zewdu Wondafrash, Adam Belay Wondmieneh, Yordanos Gizachew Yeshitila, Naohiro Yonemoto, Chuanhua Yu, Zoubida Zaidi, Afshin Zarghi, Shira Zelber-Sagi, Kaleab Alemayehu Zewdie, Zhi-Jiang Zhang, Xiu-Ju Zhao, Mohsen Naghavi, Reza Malekzadeh

## Abstract

**Background:**

Cirrhosis and other chronic liver diseases (collectively referred to as cirrhosis in this paper) are a major cause of morbidity and mortality globally, although the burden and underlying causes differ across locations and demographic groups. We report on results from the Global Burden of Diseases, Injuries, and Risk Factors Study (GBD) 2017 on the burden of cirrhosis and its trends since 1990, by cause, sex, and age, for 195 countries and territories.

**Methods:**

We used data from vital registrations, vital registration samples, and verbal autopsies to estimate mortality. We modelled prevalence of total, compensated, and decompensated cirrhosis on the basis of hospital and claims data. Disability-adjusted life-years (DALYs) were calculated as the sum of years of life lost due to premature death and years lived with disability. Estimates are presented as numbers and age-standardised or age-specific rates per 100 000 population, with 95% uncertainty intervals (UIs). All estimates are presented for five causes of cirrhosis: hepatitis B, hepatitis C, alcohol-related liver disease, non-alcoholic steatohepatitis (NASH), and other causes. We compared mortality, prevalence, and DALY estimates with those expected according to the Socio-demographic Index (SDI) as a proxy for the development status of regions and countries.

**Findings:**

In 2017, cirrhosis caused more than 1·32 million (95% UI 1·27–1·45) deaths (440 000 [416 000–518 000; 33·3%] in females and 883 000 [838 000–967 000; 66·7%] in males) globally, compared with less than 899 000 (829 000–948 000) deaths in 1990. Deaths due to cirrhosis constituted 2·4% (2·3–2·6) of total deaths globally in 2017 compared with 1·9% (1·8–2·0) in 1990. Despite an increase in the number of deaths, the age-standardised death rate decreased from 21·0 (19·2–22·3) per 100 000 population in 1990 to 16·5 (15·8–18·1) per 100 000 population in 2017. Sub-Saharan Africa had the highest age-standardised death rate among GBD super-regions for all years of the study period (32·2 [25·8–38·6] deaths per 100 000 population in 2017), and the high-income super-region had the lowest (10·1 [9·8–10·5] deaths per 100 000 population in 2017). The age-standardised death rate decreased or remained constant from 1990 to 2017 in all GBD regions except eastern Europe and central Asia, where the age-standardised death rate increased, primarily due to increases in alcohol-related liver disease prevalence. At the national level, the age-standardised death rate of cirrhosis was lowest in Singapore in 2017 (3·7 [3·3–4·0] per 100 000 in 2017) and highest in Egypt in all years since 1990 (103·3 [64·4–133·4] per 100 000 in 2017). There were 10·6 million (10·3–10·9) prevalent cases of decompensated cirrhosis and 112 million (107–119) prevalent cases of compensated cirrhosis globally in 2017. There was a significant increase in age-standardised prevalence rate of decompensated cirrhosis between 1990 and 2017. Cirrhosis caused by NASH had a steady age-standardised death rate throughout the study period, whereas the other four causes showed declines in age-standardised death rate. The age-standardised prevalence of compensated and decompensated cirrhosis due to NASH increased more than for any other cause of cirrhosis (by 33·2% for compensated cirrhosis and 54·8% for decompensated cirrhosis) over the study period. From 1990 to 2017, the number of prevalent cases more than doubled for compensated cirrhosis due to NASH and more than tripled for decompensated cirrhosis due to NASH. In 2017, age-standardised death and DALY rates were lower among countries and territories with higher SDI.

**Interpretation:**

Cirrhosis imposes a substantial health burden on many countries and this burden has increased at the global level since 1990, partly due to population growth and ageing. Although the age-standardised death and DALY rates of cirrhosis decreased from 1990 to 2017, numbers of deaths and DALYs and the proportion of all global deaths due to cirrhosis increased. Despite the availability of effective interventions for the prevention and treatment of hepatitis B and C, they were still the main causes of cirrhosis burden worldwide, particularly in low-income countries. The impact of hepatitis B and C is expected to be attenuated and overtaken by that of NASH in the near future. Cost-effective interventions are required to continue the prevention and treatment of viral hepatitis, and to achieve early diagnosis and prevention of cirrhosis due to alcohol-related liver disease and NASH.

**Funding:**

Bill & Melinda Gates Foundation.

**Research in context**

**Evidence before this study**

In 1994, an analysis of official death certification data derived from the WHO database was conducted, and reports were released on deaths due to cirrhosis from 1955 to 1990 in high-income countries. More recently, a 2014 report on findings from the Global Burden of Disease Study (GBD) 2010 reported the mortality of cirrhosis and other chronic liver diseases in 187 countries from 1980 to 2010. Both estimates showed increasing numbers and decreasing rates across almost all countries. Other local studies have also shown the contributions of vaccination and lifestyle modification to reductions in death rates, incidence, and prevalence of cirrhosis.

**Added value of this study**

In this study we present the results of the latest iteration of GBD (2017), regarding cirrhosis and other chronic liver diseases (hereafter referred to as cirrhosis). Compared with GBD 2016, we had access to more data points, we used novel and more robust methods, we made separate estimates for decompensated and compensated cirrhosis, and we differentiated non-alcoholic steatohepatitis as the fifth cause of cirrhosis along with hepatitis B and C, alcohol-related liver disease, and the broad category of other causes. Our study provides a valuable insight into the shifting burden of cirrhosis due to different causes. Although hepatitis B caused the greatest proportion of cirrhosis deaths in 2017, our findings suggest that non-alcoholic steatohepatitis might become the leading cause of cirrhosis in the near future.

**Implications of all the available evidence**

The number of deaths and prevalent cases of both decompensated and compensated cirrhosis are increasing, despite a decrease in age-standardised death rates. The contribution of different causes to mortality and morbidity is expected to change as a result of available prevention and treatment modalities. Because of variation in the contribution of the five causes of cirrhosis assessed in this study and varying amounts and trends of burden across nations, policies should be targeted at national and even subnational levels accordingly. We intend for our findings to be used by policy makers and the public to address the increasing burden of chronic liver diseases.

## Introduction

Cirrhosis is the leading cause of liver-related death globally.^1^ It is the end stage of progressive liver fibrosis, in which the hepatic architecture is distorted.^2^ In the initial stages, cirrhosis is compensated. Most patients are asymptomatic at this stage, and cirrhosis is usually discovered incidentally during medical encounters for other reasons. Thus, reports on the prevalence of compensated cirrhosis are almost always underestimated. Decompensation in patients with compensated cirrhosis is usually defined as the first occurrence of ascites, oesophageal variceal bleeding, hepatic encephalopathy, and, in some individuals, increased bilirubin concentration.^3^, ^4^ Because of the nature of decompensation, these patients are rapidly brought to medical attention, and thus reports on the prevalence of decompensated cirrhosis are probably much more accurate than those of compensated cirrhosis.^3^ Once decompensation occurs, the mortality and morbidity resulting from cirrhosis increase sharply, and depending on the cause of decompensation, the 1-year case-fatality rate can be as high as 80%.^5^, ^6^ Almost all of the mortality and morbidity resulting from cirrhosis is caused by the decompensated type. Such patients need frequent medical attention and an increasing amount of medication over the disease course. Quality of life is affected and frequent hospitalisations (admissions and stays) are required.^7^ As the disease progresses, hospital stays become more frequent and more prolonged. Finally, patients either die or receive a liver transplant, which is a high-burden option for patients, health-care systems, and health financing and governance.^7^

Compensated cirrhosis is more benign. Patients often have a life expectancy similar to that of healthy adults if cirrhosis remains compensated.^5^ However, identifying these patients is important because if they are not treated appropriately, they are at risk of progressing to decompensated cirrhosis.^3^

Cirrhosis is generally considered to be irreversible at later stages, although reversal has been documented in many individuals with compensated cirrhosis after treating the underlying cause.^8^, ^9^, ^10^ The most common causes of cirrhosis are chronic hepatitis B and C, alcohol-related liver disease, and non-alcoholic steatohepatitis (NASH).^7^ Patients with decompensated cirrhosis are susceptible to complications and a reduction in life expectancy.^11^

Despite being a global health challenge, estimates of cirrhosis mortality and morbidity are not widely available, especially at national levels, because of data sparsity in many regions where cirrhosis is fatal, particularly in Africa.^12^ Accurate mortality data are also scarce, because official death records under-report cirrhosis as a cause of death.^13^ The burden of cirrhosis differs considerably across locations, sexes, races and ethnicities, and socioeconomic strata, and the burden has also varied substantially over time. Moreover, the incidence and prevalence of cirrhosis are not well established, even in population-based studies. Studies are further limited by referral bias; the structure of the population under study (inpatient *vs* outpatient); absence of standardised definitions; and diversity in methods of assessment, including self-reporting, laboratory tests and non-invasive biomarkers, imaging, liver biopsies, and death certificates.^12^

Data by cause are also inaccurate and diverse. Clarification of the underlying cause and stage of cirrhosis is challenging, even in high-income countries.^14^ It is generally well-recognised that the contribution of underlying causes is variable across locations and has changed over time. With the availability of methods for controlling hepatitis B and curing hepatitis C,^15^ along with rising prevalence of metabolic syndrome and obesity as a plausible underlying cause, the proportion of cirrhosis mortality and morbidity caused by NASH is expected to increase in the near future.^16^, ^17^

Previous studies have investigated the burden of cirrhosis at the global level. In 1994, La Vecchia and colleagues^18^ analysed official death certification data from 1955 to 1990 derived from the WHO database to produce estimates covering 38 high-income countries. In 2014, Mokdad and colleagues^19^ reported the results of the 2010 iteration of the Global Burden of Disease Study (GBD) on mortality due to cirrhosis by four causes in 187 countries from 1980 to 2010. Both studies reported increasing numbers of deaths and decreasing age-standardised mortality rates in most countries. However, updated estimates on mortality and morbidity caused by cirrhosis and the prevalence of decompensated and compensated types by cause were not available, to our knowledge, before this study. Using data from GBD 2017, this study provides estimates of the prevalence, mortality, and disability-adjusted life-years (DALYs) of cirrhosis and other chronic liver diseases by cause, sex, and age at global, regional, and national levels across 195 countries and territories from 1990 to 2017.

## Methods

### Overview

This study is part of GBD 2017,^1^, ^20^, ^21^, ^22^ which was a systematic effort to estimate the levels and trends of burden caused by 359 diseases and injuries by sex, age, year (1990–2017), and location, including seven super-regions, 21 regions, and 195 countries and territories. We modelled the mortality and prevalence of cirrhosis and other chronic liver diseases, hereafter collectively referred to as cirrhosis. We report numbers and age-standardised and age-specific rates for mortality, prevalence, and DALYs, which are the sum of years of life lost (YLLs) due to premature death and years lived with disability (YLDs).^1^, ^20^, ^21^, ^22^ This study is compliant with the Guidelines for Accurate and Transparent Health Estimates Reporting.

We considered diagnoses coded as B18 and K70–77 in the International Classification of Diseases 10th revision (ICD-10) to be compensated and decompensated cirrhosis. ICD-10 codes B18.0–18.2 were mapped to chronic viral hepatitis B and C in the GBD cause list, B18.8 and 18.9 to other causes, K70 to alcohol-related liver disease, K75.81 to NASH, and the rest of the codes to the category of other causes, which included but was not limited to autoimmune hepatitis (K75.4). The detailed list of ICD-10 codes mapped to the GBD cause list is reported in the appendix (p 4). The causes grouped in the “other chronic liver diseases” category mainly included autoimmune hepatitis, toxic liver diseases, other inflammatory liver diseases, chronic hepatitis not specified, and other diseases of the liver (K76). ICD-10 codes for acute hepatitis were not included in this study. Non-alcoholic fatty liver disease was considered to be a separate entity from compensated and decompensated cirrhosis, and codes for diabetes were also excluded. Finally, deaths caused by hepatocellular carcinoma were excluded because: ICD-10 codes define hepatocellular carcinoma as a cause irrespective of liver cirrhosis; hepatocellular carcinoma can be distinguished from cirrhosis in countries with adequate data; and implications on natural history and management of the two causes are not similar.

Existing evidence shows that ICD-10 coding is valid for defining overall cirrhosis and chronic liver diseases in most of the data sources used to assemble the cause-of-death database but does not have the required accuracy for reporting cirrhosis by the five causes estimated in the current study.^23^, ^24^ The models that we used to split the parent cirrhosis mortality and morbidity into the five causes are described below.

### Mortality estimates

Details of the methods used to estimate mortality have been published previously.^1^, ^20^, ^21^, ^22^ We modelled decompensated cirrhosis mortality due to the five causes defined by the aforementioned ICD-10 codes using all available data in the cause-of-death database. We obtained data from vital registrations, vital registration samples, and verbal autopsies. Vital registrations are systems that governments use to record the vital events of their residents, including causes of death. Vital registration samples are nationally representative cluster samples in countries where vital registration has low coverage or is unavailable. Verbal autopsy is a method by which causes of death and cause-specific mortality fractions in populations without a complete vital registration system are determined. To record data through verbal autopsy, trained interviewers collect information about the symptoms, signs, and demographic characteristics of a recently deceased person from an individual familiar with the deceased person. The majority of the cause-of-death data are vital registration data obtained from the WHO Mortality Database, which consists of data submitted to WHO by individual countries. Vital registration data are also obtained from country-specific mortality databases operated by official offices. We collected 19 329 data points from vital registrations, 793 data points from vital registration samples, and 1263 points from verbal autopsies. To address problems of zero counts in vital registration and verbal autopsy for a given age group in a given year, we used a Bayesian noise-reduction algorithm.^1^ We used Cause of Death Ensemble models (CODEm) to estimate liver cirrhosis mortality with uncertainty by age, sex, country or territory, and year. CODEm is the framework used to model most cause-specific death rates in GBD.^1^ First, all available data were identified and gathered. Second, a diverse set of plausible models were developed to capture well-documented associations in the estimates. The use of a wide range of individual models to create an ensemble predictive model has been shown to outperform techniques that use only a single model, both in cause-of-death estimation and in more general prediction applications.^25^ Third, the out-of-sample predictive validity was assessed for all individual models, which were then ranked for use in the ensemble modelling stage. Finally, differently weighted combinations of individual models were evaluated to select the ensemble model with the highest out-of-sample predictive validity.

The methods we used for propagating uncertainty are the same as those used in previous GBD iterations. At each step of the computation process, we took 1000 draws and then computed final estimates using the mean estimate across the draws. 95% uncertainty intervals (UIs) were calculated as the 25th and 975th ranked values across all 1000 draws.

In GBD 2017, NASH was included as a fifth cause of cirrhosis for the first time. Cirrhosis caused by NASH was previously nested within the category of other cirrhosis. As discussed previously, we were able to use the cause-of-death database only for estimating overall cirrhosis and not its five causes. Instead, we used DisMod-MR 2.1 to split the parent cirrhosis mortality estimates and used liver cancer aetiological proportion models as covariates. These covariates included alcohol consumption (L per capita), HBsAg seroprevalence, hepatitis C (anti-hepatitis C virus antibody) seroprevalence, and obesity. Proportions from the five aetiological models were then rescaled to sum to one at the draw level and used to split the parent cirrhosis mortality estimates.

### Morbidity estimates

We modelled total cirrhosis prevalence and decompensated and compensated cirrhosis on the basis of hospital data and claims data, using different definitions for each model. Total cirrhosis prevalence was defined as any diagnosis of cirrhosis. The distinction between decompensated and compensated cirrhosis was based on the diagnosis made upon hospital admission. If cirrhosis was the primary diagnosis upon hospital admission, we considered the case to be decompensated. If cirrhosis was diagnosed in outpatient settings or was detected among patients admitted to the hospital for other causes, we considered the case to be compensated.

A systematic review of the literature was done to identify studies on the proportion of cirrhosis cases attributable to alcohol-related liver disease, hepatitis B, hepatitis C, NASH, and other causes. We re-extracted all literature within our databases on cirrhosis cause proportions for cirrhosis due to NASH. We searched the peer-reviewed literature via PubMed and solicited sources from GBD 2017 collaborators. The inclusion criteria stipulated that the publication year was 1980 or later; the sample was representative of patients with decompensated cirrhosis (eg, studies of patients with both hepatocellular carcinoma and hepatitis were excluded); sufficient information was provided on study method and sample characteristics to assess the quality of the study; and hepatitis B and C were confirmed via HBsAg in the case of hepatitis B, and anti-hepatitis C virus antibody in the case of hepatitis C. The number of site-years of data by cause is provided in the appendix (p 4).

We modelled cirrhosis prevalence using hospital data and cause-specific mortality rates, assuming no remission.^20^ Similar to the mortality estimates, we developed aetiological proportion models using DisMod-MR 2.1 and used the results of these models to split the parent total cirrhosis prevalence estimates. We adopted the aetiological proportion model from liver cancer. We first developed five single-parameter DisMod models, each to estimate the proportion of liver cancer due to a given cause (ie, alcohol, hepatitis B, hepatitis C, NASH, and other). Estimates from these liver cancer models were then used as covariates in the five corresponding cirrhosis aetiological models. Proportions from the five aetiological models were then rescaled to sum to one at the draw level and used to split the parent cirrhosis estimates. We multiplied these fractions by prevalence separately for decompensated and compensated cirrhosis by age, sex, year, and location, and estimated the prevalence due to cirrhosis by each cause. Upon calculation of cause fractions, we encountered some reports on cirrhosis due to more than one cause. In these cases, we redistributed the numbers proportionally on the number of cases diagnosed with only one cause.

To calculate YLDs, prevalence was multiplied by a disability weight, which represents the magnitude of the health loss associated with disease. Disability weights are measured on a scale from 0 to 1, where 0 is a state of full health and 1 is death. Compensated cirrhosis has a disability weight of 0 because of its asymptomatic nature. Decompensated cirrhosis from any cause has a disability weight of 0·178 (95% UI 0·122–0·250).^20^

We used the Socio-demographic Index (SDI) to determine the relationship between the development level of a region or country and cirrhosis mortality, prevalence, and DALYs. In GBD 2017, the SDI was revised to better reflect the development status of each country. The SDI ranges from 0 (worst) to 1 (best) and is a composite measure of the total fertility rate in women under the age of 25 years, mean education for individuals aged 15 years and older, and lag-distributed income per capita.

All estimates are reported in terms of counts, rates per 100 000 population, and percentages. Population estimates independently produced by GBD 2017 were used as references for calculating age-standardised death rates and age-standardised prevalence.^26^ 95% UIs, including all sources of uncertainty arising from measurement error, systematic biases, and modelling, are reported for all estimates. All estimates of mortality, prevalence, DALYs, YLDs, and YLLs are reported by sex and age, and across location and time period studied (1990–2017).

### Role of the funding source

The funder of the study had no role in study design, data collection, data analysis, data interpretation, or the writing of the report. The corresponding authors had full access to the data in the study and had final responsibility for the decision to submit for publication.

## Results

**Figure 1:**
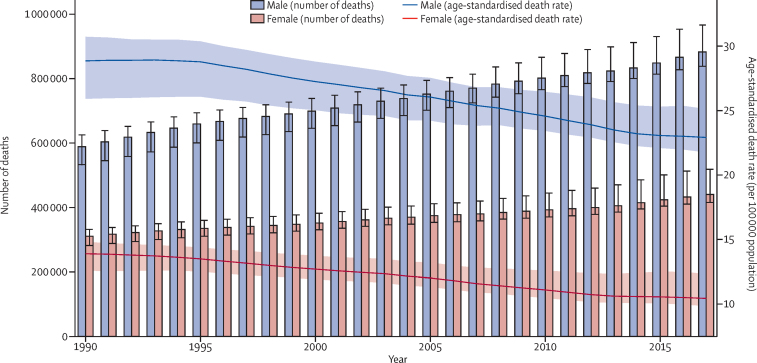
Counts and age-standardised rates of cirrhosis death at the global level by sex, 1990–2017 Error bars indicate 95% UIs for number of deaths. Shading indicates 95% UIs for age-standardised death rates. UI=uncertainty interval.

Globally, cirrhosis caused more than 1·32 million (95% UI 1·27–1·45) deaths in 2017, with 440 000 deaths (416 000–518 000; 33·3%) in females and 883 000 (838 000–967 000; 66·7%) in males, compared with less than 899 000 (829 000–948 000) deaths for both sexes in 1990 (figure 1; appendix p 5). These deaths constituted 2·4% (2·3–2·6) of all deaths globally in 2017 compared with 1·9% (1·8–2·0) in 1990. The age-standardised death rate at the global level decreased from 21·0 (19·2–22·3) per 100 000 population in 1990 to 16·5 (15·8–18·1) per 100 000 population in 2017 (figure 1). The age-standardised death rate was consistently lowest in the high-income super-region (10·1 [9·8–10·5] per 100 000 in 2017) and highest in the sub-Saharan Africa super-region (32·2 [25·8–38·6] per 100 000 in 2017) in all years from 1990 to 2017 (appendix p 237). The pattern of age-standardised death rates between 1990 and 2017 was similar in males and females, although the rates were consistently higher in males than females in all super-regions (estimates available through the GBD online results tool and data visualisation tool) and all years from 1990 to 2017 (figure 1).

Cirrhosis led to nearly 41·4 million (95% UI 39·6–45·1) DALYs in 2017, which was an increase from just over 30·5 million (28·6–32·2) in 1990. The age-standardised rate of DALYs decreased from 656·4 (612·8–689·2) per 100 000 population in 1990 to 510·7 (487·6–557·1) per 100 000 population in 2017 (appendix p 15). In 2017, cirrhosis caused 28·8 million DALYs (27·3–31·4) in males and 12·6 million DALYs (11·9–14·7) in females, compared with 20·6 million DALYs (18·7–21·8) in males and 9·9 million (9·1–11·1) DALYs in females in 1990. The age-standardised DALY rates in 2017 were 719·3 (683·0–784·9) per 100 000 population in males and 307·6 (288·5–359.6) per 100 000 in females, which was a substantial decrease from 1990 (903·1 [820·1–956·1] per 100 000 in males and 415·5 [382·5–457·1] per 100 000 in females in 1990; estimates available through the GBD online results tool).

The number of prevalent cases of decompensated cirrhosis globally increased from more than 5·20 million (95% UI 5·08–5·32) in 1990 to over 10·6 million (10·3–10·9) in 2017 (appendix p 23), of which 6·42 million (6·23–6·60; 60·3%) prevalent cases were in males and 4·23 million (4·10–4·35; 39·7%) were in females (data not shown). The age-standardised prevalence of decompensated cirrhosis increased from 110·6 (108·0–113·0) per 100 000 population in 1990 to 132·5 (128·6–136·2) per 100 000 population in 2017 (appendix p 23). In males, the age-standardised prevalence in 2017 was 162·7 (157·9–167·3) per 100 000, with a 21·1% (19·7–22·4) increase from 1990 to 2017. In females, the age-standardised prevalence in 2017 was 103·5 (100·6–106·3) per 100 000, with an 18·1% (16·9–19·2) increase from 1990 to 2017 (data not shown).

The number of global prevalent cases of compensated cirrhosis increased from 65·9 million (95% UI 63·4–68·7) in 1990 to just over 112 million (107–119) in 2017 (appendix p 32), of which 66·1 million (62·7–69·7; 58·8%) prevalent cases were in males and 46·3 million (43·8–48·8; 41·2%) were in females (data not shown). The age-standardised prevalence of compensated cirrhosis increased from 1354·5 (1300·6–1411·7) per 100 000 in 1990 to 1395·0 (1323·5–1470·5) in 2017 (appendix p 32).

The age-standardised prevalence of compensated cirrhosis in males was 1605·4 (95% UI 1543·3–1671·2) per 100 000 in 1990 and 1651·4 (1567·5–1742·8) per 100 000 in 2017. The age-standardised prevalence of compensated cirrhosis in females was 1103·3 (1055·6–1152·4) per 100 000 in 1990 and 1142·4 (1079·9–1204·0) per 100 000 in 2017 (data not shown). The percentage change in age-standardised prevalence rate of compensated cirrhosis from 1990 to 2017 was 2·9% (0·4–5·2) in males and 3·5% (1·3–5·6) in females (data not shown).

**Figure 2:**
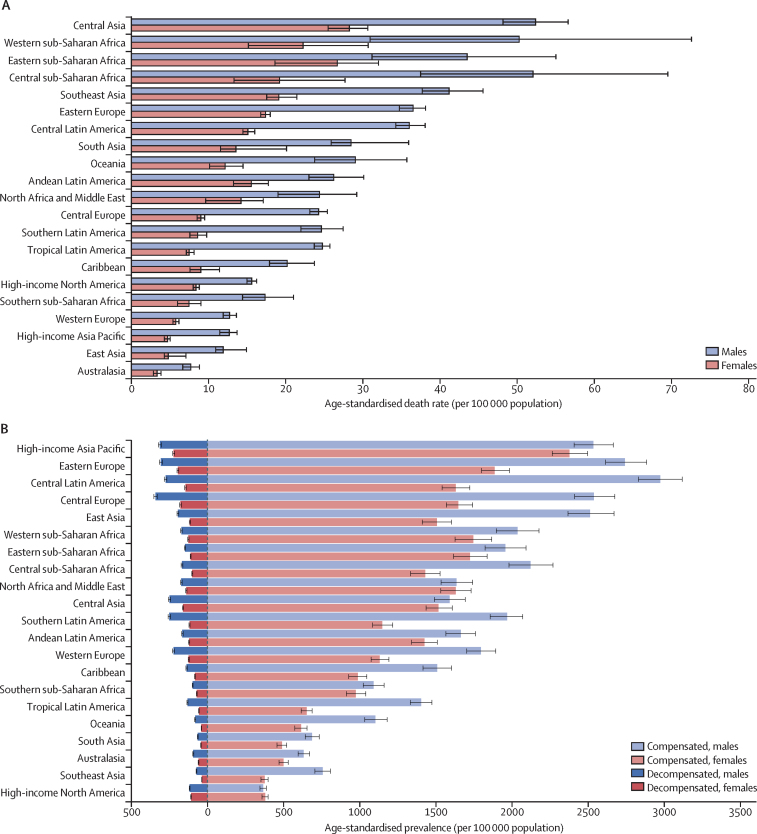
Age-standardised rates for cirrhosis by region and sex, 2017 (A) Age-standardised death rate. (B) Age-standardised prevalence rate of compensated and decompensated cirrhosis. Error bars indicate 95% uncertainty intervals for age-standardised rates.

At the regional level, central Asia had the highest age-standardised death rate due to cirrhosis in 2017 for males, females, and both sexes combined (39·0 [95% UI 36·2–41·5] per 100 000 for both sexes combined; figure 2A), with deaths largely driven by alcohol-related liver disease (36·6% of all cirrhosis deaths). Western, eastern, and central sub-Saharan Africa had the next highest age-standardised death rates for both sexes combined in 2017, with rates of 35·8 (23·9–49·9) per 100 000 population in western Africa, 34·8 (26·4–42·2) per 100 000 population in eastern Africa, and 34·3 (25·9–45·2) per 100 000 population in central sub-Saharan Africa (figure 2A). In central and eastern sub-Saharan Africa in 2017, the most common cause of death due to cirrhosis was hepatitis C (32·4% and 28·9%, respectively), followed by hepatitis B (31·2% and 25·9%, respectively; appendix p 41). However, in western sub-Saharan Africa, the most common cause of death due to cirrhosis was hepatitis B (48·9%) in all countries in the region, whereas the proportion of deaths from hepatitis C was much lower in this region (7·8%) than in the other sub-Saharan African regions (appendix p 41).

Southeast Asia ranked fifth in terms of age-standardised death rate from cirrhosis across regions in 2017 (29·5 [95% UI 27·7–31·9] per 100 000) but had low age-standardised prevalence rates of compensated and decompensated cirrhosis (figure 2A, B); both deaths and prevalent cases were mainly caused by hepatitis B and C (appendix pp 41, 48, 55).

Eastern Europe ranked sixth in terms of age-standardised death rate due to cirrhosis in 2017 (25·9 [95% UI 25·0–26·7] per 100 000), and, as in central Asia, deaths were primarily caused by alcohol-related liver disease (figure 2A; appendix pp 5, 41). These two regions were the only ones in which age-standardised death rates significantly increased over the study period: by 137·0% (129·5–146·2) in eastern Europe and by 50·5% (38·6–59·9) in central Asia (appendix p 5). The age-standardised death rate increased by more than 100% in all but one country (Moldova) in eastern Europe (appendix p 5). Eastern Europe had a high and increasing age-standardised prevalence of both decompensated and compensated cirrhosis from 1990 to 2017; prevalent cases were predominantly caused by alcohol-related liver disease (figure 2B; appendix pp 48, 55).

The age-standardised death rate was lowest in Australasia (5·4 [95% UI 4·9–6·0] per 100 000 population), east Asia (8·3 [7·6–10·7] per 100 000 population), and high-income Asia Pacific (8·6 [7·9–9·1] per 100 000 population; figure 2A). Australasia was among the regions with the lowest age-standardised prevalence of both compensated and decompensated cirrhosis (figure 2B). Deaths due to cirrhosis and prevalent cases of compensated and decompensated cirrhosis in this region were primarily caused by hepatitis C (appendix pp 48, 55). East Asia had the second-lowest age-standardised death rate, but it had a relatively high age-standardised prevalence rate for compensated and decompensated cirrhosis combined (figure 2A, B). Deaths due to cirrhosis and prevalent cases of compensated and decompensated cirrhosis in this region were driven primarily by hepatitis B (appendix pp 41, 48, 55).

High-income Asia Pacific had the third lowest age-standardised death rate in 2017 and all countries in this region experienced a steep decline over the study period, particularly South Korea (figure 2A; appendix p 5). However, this region had the highest age-standardised prevalence for both compensated cirrhosis (2455·0 [95% UI 2344·9–2575·8] per 100 000) and decompensated cirrhosis (267·4 [259·8–275·1] per 100 000) in 2017 (figure 2B). The high age-standardised prevalence in this region was primarily driven by high numbers of prevalent cases in Japan, where the highest proportions of compensated and decompensated cirrhosis were due to hepatitis C (appendix pp 23, 32, 48, 55).

Western Europe, southern sub-Saharan Africa, and high-income North America had the fourth to sixth lowest age-standardised death rates of cirrhosis in 2017 (figure 2A; appendix p 5). In western Europe, deaths were primarily caused by alcohol-related liver disease (41·7%), whereas in southern sub-Saharan Africa and high-income North America, deaths were primarily caused by hepatitis C (33·9% and 34·7%, respectively; appendix p 41). Western Europe and southern sub-Saharan Africa had modest age-standardised prevalence rates of both compensated and decompensated cirrhosis, whereas high-income North America had the lowest age-standardised prevalence of compensated cirrhosis of all regions; prevalent cases in this region were mainly caused by hepatitis C (figure 2B; appendix pp 48, 55).

South Asia and Oceania had modest age-standardised death rates but were among the regions with the lowest age-standardised prevalence of compensated and decompensated cirrhosis (figure 2A, B). Deaths due to cirrhosis and prevalent cases of compensated and decompensated cirrhosis were primarily caused by hepatitis B in these regions (appendix p 41).

Central Latin America was among the regions with the highest age-standardised prevalence of both compensated (2272·3 [95% UI 2167·0–2383·4] per 100 000) and decompensated (206·6 [200·4–212·6] per 100 000) cirrhosis (figure 2B; appendix pp 23, 32), although age-standardised death rates were modest in this region. Other regions in Latin America had modest age-standardised death rates and age-standardised prevalence rates of both types of cirrhosis. Cirrhosis deaths in Latin American regions were primarily driven by alcohol-related liver disease, except for tropical Latin America (38·1% in Andean Latin America, 36·6% in central Latin America, 34·8% in the Caribbean, and 34·6% in southern Latin America; appendix p 41). Cirrhosis deaths in tropical Latin America were mainly attributable to hepatitis C. In every Latin American region, the cause of the highest proportion of cirrhosis deaths was also the cause of the highest proportion of compensated and decompensated cirrhosis prevalent cases (appendix pp 48, 55).

Finally, north Africa and the Middle East had modest age-standardised death and prevalence rates from cirrhosis, with the largest proportion of deaths due to cirrhosis and prevalent cases of compensated and decompensated cirrhosis driven by hepatitis B and C.

**Figure 3:**
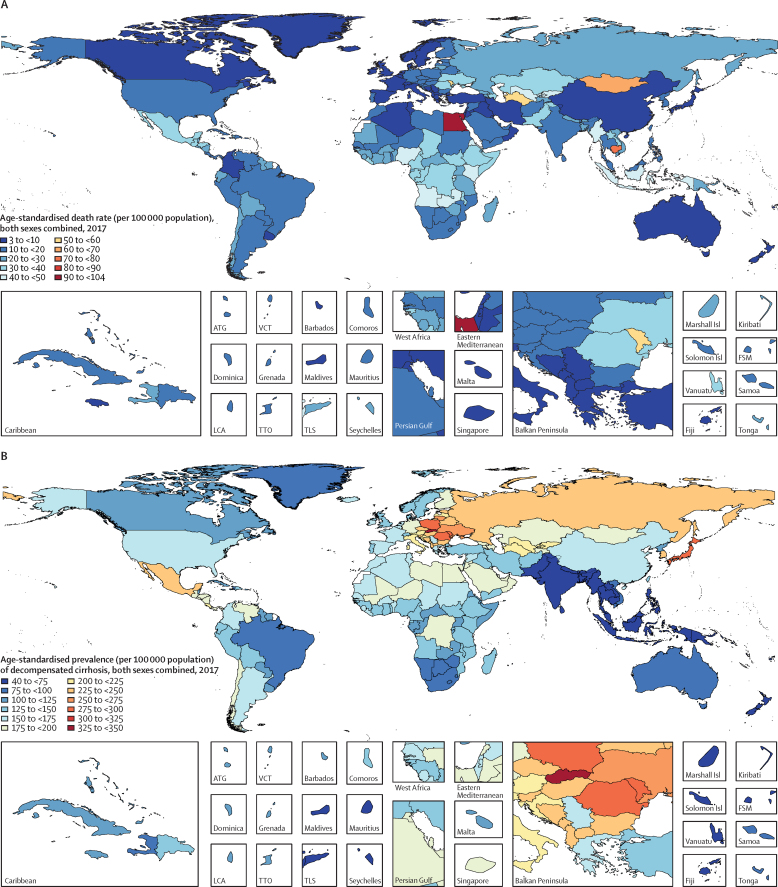

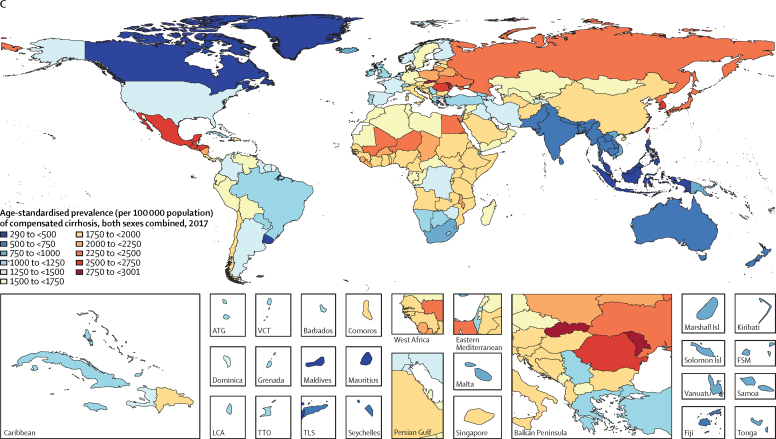
Age-standardised death rate for cirrhosis in 2017 (A), age-standardised prevalence for decompensated cirrhosis (B), and age-standardised prevalence for compensated cirrhosis (C), 2017 ATG=Antigua and Barbuda. FSM=Federated States of Micronesia. Isl=Islands. LCA=Saint Lucia. TLS=Timor-Leste. TTO=Trinidad and Tobago. VCT=Saint Vincent and the Grenadines.

Specific country and territory data can be found in the appendix (pp 5–14 for age-standardised death rates; pp 15–22 for DALYs, pp 23–40 for prevalence, and pp 41–68 for cause). Egypt had the highest age-standardised death rate of cirrhosis in all years from 1990 to 2017, despite a 22·4% (95% UI 1·5–42·1) decrease from 1990 (133·1 [76·4–150·8] per 100 000) to 2017 (103·3 [64·4–133·4] per 100 000). The second highest rate was in Rwanda in 1990 (88·1 [71·9–104·8] per 100 000) and in Cambodia in 2017 (79·4 [67·4–96·1] per 100 000; figure 3A; appendix p 5). In 2017, 41·5% of deaths due to cirrhosis in Egypt were caused by hepatitis B and 34·4% were caused by hepatitis C (appendix p 41).

The lowest age-standardised death rate of cirrhosis in 2017 was in Singapore (3·7 [95% UI 3·3–4·0] per 100 000 population). The largest increase in age-standardised death rate from 1990 to 2017 was in Lithuania (176·7% [154·1–203·2]; appendix p 5). The eight countries with the largest increases in age-standardised death rate from cirrhosis over the study period (in order: Lithuania, Ukraine, Belarus, Russia, Kazakhstan, Estonia, Latvia, and Armenia) were all in eastern Europe or central Asia (appendix p 5). In all of these countries, the largest proportion of deaths in 2017 was caused by alcohol-related liver disease (appendix p 41).

Among the ten countries with the highest age-standardised prevalence of decompensated cirrhosis in 2017, eight were from central and eastern Europe (in order: Slovakia [349·6, 95% UI 338·4–361·2] per 100 000, Moldova, Romania, Poland, Ukraine, Lithuania, Albania, and Hungary), in addition to Japan (third-highest; 279·8 [272·0–287·8] per 100 000) and South Korea (eighth-highest; 245·9 [237·8–253·9] per 100 000; figure 3B; appendix p 23). The Philippines had the lowest age-standardised prevalence of decompensated cirrhosis in 2017 (41·8 [40·2–43·4 per 100 000 population]), with the largest proportion of prevalent cases due to hepatitis B.

Despite decreasing age-standardised prevalence of compensated cirrhosis since 1990, South Korea and Japan were among the ten countries with the highest rates in 2017 (figure 3C; appendix p 32). The three countries or territories with the highest age-standardised prevalence of compensated cirrhosis were Moldova, Taiwan (Province of China), and Slovakia.

**Figure 4:**
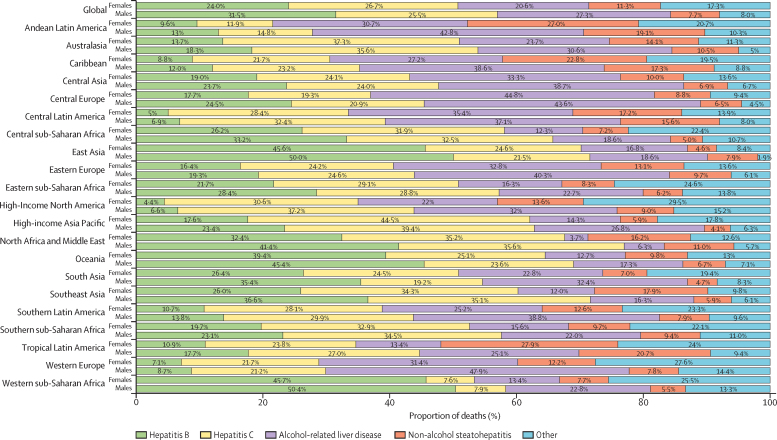
Proportion of deaths due to five causes of cirrhosis at global and regional levels by sex, 2017

Globally, in 2017, 31·5% of cirrhosis deaths in males were caused by hepatitis B, 25·5% were caused by hepatitis C, 27·3% were caused by alcohol-related liver disease, 7·7% were caused by NASH, and 8·0% resulted from other causes. In females, the proportions of cirrhosis deaths caused by hepatitis B (24·0%) and alcohol-related liver disease (20·6%) were lower than in males, the proportion caused by hepatitis C (26·7%) was similar to that in males, and the proportion caused by NASH (11·3%) and by other causes (17·3%) was higher than in males (figure 4).

**Figure 5:**
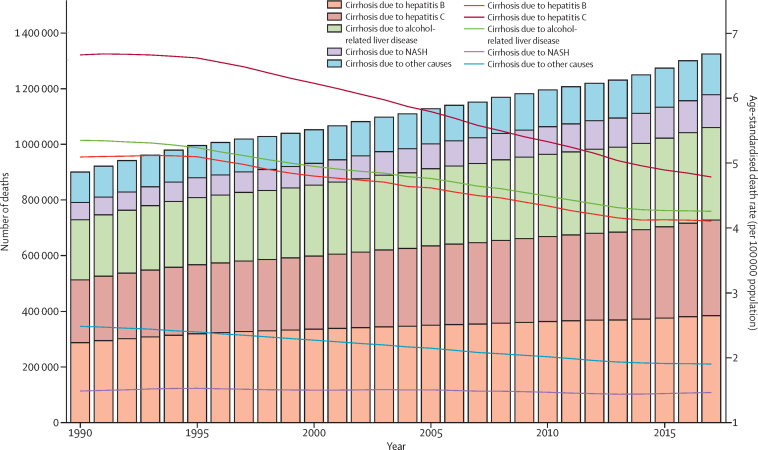
Number of deaths and age-standardised death rates at the global level by cause of cirrhosis, 1990–2017 Bars refer to number of deaths in each year. Lines refer to age-standardised death rate in each year. NASH=non-alcoholic steatohepatitis.

Hepatitis B caused around 287 000 (95% UI 252 000–318 000) deaths due to cirrhosis in 1990, which increased to almost 384 000 (349 000–442 000) in 2017. 278 000 (252 000–321 000; 72·5%) of these deaths in 2017 occurred in males, and 106 000 (94 000–130 000; 27·5%) occurred in females (appendix p 69). However, the age-standardised death rate for both sexes combined decreased steadily from 6·7 (5·8–7·4) per 100 000 in 1990 to 4·8 (4·3–5·5) per 100 000 in 2017 (figure 5; appendix p 69). The age-standardised death rate of cirrhosis due to hepatitis B was 7·2 (6·5–8·3) per 100 000 in males and 2·5 (2·2–3·1) per 100 000 in females in 2017. Hepatitis B caused 36·7 million (95% UI 34·1–39·5) prevalent cases of compensated cirrhosis and 2·97 million (2·81–3·12) cases of decompensated cirrhosis in 2017. The age-standardised prevalence of decompensated cirrhosis caused by hepatitis B increased from 30·9 (95% UI 29·3–32·2) per 100 000 in 1990 to 36·6 (34·7–38·4) per 100 000 in 2017 (appendix p 139). The age-standardised prevalence of compensated cirrhosis caused by hepatitis B did not change notably from 1990 (461·8 [435·8–491·2] per 100 000) to 2017 (451·9 [95% UI 420·0–485·9] per 100 000; appendix p 184). Across regions, the proportion of deaths from cirrhosis due to hepatitis B was highest in western sub-Saharan Africa (48·9%) and lowest in high-income North America (5·7%; figure 4). The proportion of cirrhosis mortality that was caused by hepatitis B ranged from 5·7% in the USA to 54·8% in Chad (appendix pp 41).

Hepatitis C caused more than 225 000 (95% UI 202 000–249 000) cirrhosis deaths in 1990, which increased to over 342 000 (313 000–381 000) in 2017. 225 000 (203 000–251 000; 65·8%) of these deaths in 2017 occurred in males, and 117 000 (107 000–135 000; 34·2%) occurred in females (appendix p 83). The age-standardised death rate decreased from 5·3 (4·8–5·9) per 100 000 in 1990 to 4·2 (3·9–4·7) per 100 000 in 2017 (figure 5; appendix p 83). The age-standardised death rate due to hepatitis C was 5·8 (5·3–6·5) per 100 000 in males and 2·8 (2·5–3·2) in females in 2017. Hepatitis C caused 27·72 million (25·52–30·00) cases of compensated cirrhosis and 2·64 million (2·49–2·81) cases of decompensated cirrhosis in 2017. The age-standardised prevalence of compensated cirrhosis caused by hepatitis C was 327·0 (303·9–349·7) per 100 000 in 1990 and 341·1 (314·1–368·7) per 100 000 in 2017. The age-standardised prevalence of decompensated cirrhosis increased from 27·2 (25·7–28·7) per 100 000 in 1990 to 32·5 (30·6–34·5) per 100 000 in 2017 (appendix pp 148, 193). Generally, the lowest proportions of cirrhosis mortality due to hepatitis C were observed in countries in western sub-Saharan Africa (7·8%) and the highest among countries in high-income Asia-Pacific (41·3%; figure 4). The proportion of cirrhosis mortality that was caused by hepatitis C ranged from 7·1% in Niger to 55·7% in Tunisia (appendix p 41).

Alcohol-related liver disease caused just over 215 000 (95% UI 195 000–235 000) cirrhosis deaths in 1990, which increased to just over 332 000 (303 000–373 000) deaths in 2017. 241 000 (220 000–268 000; 72·6%) of these deaths in 2017 occurred in males, and 91 000 (82 000–110 000; 27·4%) occurred in females. The age-standardised death rate decreased from 5·1 (4·6–5·5) per 100 000 in 1990 to 4·1 (3·7–4·6) per 100 000 in 2017 (figure 5; appendix p 97). The age-standardised death rate due to alcohol-related liver disease was 6·2 (5·7–6·9) per 100 000 in males and 2·1 (1·9–2·6) per 100 000 in females in 2017. Alcohol-related liver disease caused 23·6 million (21·9–25·5) cases of compensated cirrhosis and 2·46 million (2·32–2·61) cases of decompensated cirrhosis in 2017. The age-standardised prevalence of decompensated cirrhosis caused by alcohol-related liver disease increased from 25·3 (23·9–26·7) per 100 000 in 1990 to 30·0 (28·2–31·8) per 100 000 in 2017. The age-standardised prevalence of compensated cirrhosis caused by alcohol-related liver disease did not change notably from 1990 (290·0 [271·9–309·9] per 100 000) to 2017 (288·1 [267·5–311·3] per 100 000; appendix pp 157, 202). Among GBD regions, the highest proportions of cirrhosis deaths due to alcohol-related liver disease were in central Europe (44·0%), western Europe (41·7%), and Andean Latin America (38·1%; figure 4; appendix p 41). The proportion of cirrhosis deaths due to alcohol-related liver disease was lowest in north Africa and the Middle East (5·3%) and in Egypt specifically (4·8%), and highest in Belgium (53·5%; appendix p 41).

NASH caused almost 61 900 (95% UI 55 400–68 000) cirrhosis deaths in 1990, which increased to around 118 000 (109 000–129 000) in 2017. 68 000 (62 000–75 000; 57·6%) of these deaths in 2017 occurred in males, and 50 000 (46 000–56 000; 42·4%) occurred in females. NASH was the only cause for which the age-standardised death rate did not decrease: the rate was 1·5 (1·3–1·6) per 100 000 in 1990 and 1·5 (1·3–1·6) per 100 000 in 2017 (figure 5; appendix p 111). The age-standardised death rate due to NASH was 1·8 (1·6–1·9) per 100 000 in males and 1·2 (1·1–1·3) per 100 000 in females in 2017. NASH caused 4·06 million (3·70–4·45) prevalent cases of compensated cirrhosis in 1990 and 9·42 million (8·57–10·34) cases in 2017 (more than doubling over the study period). NASH caused 325 000 (302 000–349 000) cases of decompensated cirrhosis in 1990 and 917 000 (850 000–986 000) cases in 2017 (nearly tripling over the study period). The age-standardised prevalence of compensated cirrhosis caused by NASH increased from 86·7 (79·0–94·6) per 100 000 in 1990 to 115·5 (105–126·5) per 100 000 in 2017, a 33·2% increase. The age-standardised prevalence of decompensated cirrhosis increased from 7·3 (6·8–7·8) per 100 000 in 1990 to 11·3 (10·4–12·1) per 100 000 in 2017, a 54·8% increase (appendix pp 166, 211). At the regional level, the proportion of deaths due to NASH was highest in Latin American regions, with the highest proportion in Tropical Latin America (22·6%) and Andean Latin America (22·2%). The lowest proportion was in high-income Asia Pacific (4·7%; figure 4; appendix p 41). At the national level, the proportion of deaths due to NASH was highest in Ecuador (25·2%) and lowest in Singapore (2·7%).

Finally, other causes led to almost 110 000 (95% UI 97 000–127 000) cirrhosis deaths in 1990, which increased to around 146 000 (131 000–165 000) in 2017. 70 000 (62 000–80 000; 48·0%) of these deaths in 2017 occurred in males, and 76 000 (67 000–90 000) occurred in females (52·0%). The age-standardised death rate decreased from 2·5 (2·2–2·8) per 100 000 in 1990 to 1·9 (1·7–2·1) per 100 000 in 2017 (figure 5; appendix p 125). Other causes led to 15·0 million (13·6–16·2) cases of compensated cirrhosis and 1·65 million (1·55–1·76) cases of decompensated cirrhosis in 2017. The age-standardised prevalence of decompensated cirrhosis due to other causes was 20·0 (18·9–21·1) per 100 000 population in 1990 and 22·2 (20·9–23·5) per 100 000 in 2017. The age-standardised prevalence of compensated cirrhosis due to other causes was 189·0 (173·0–204·6) per 100 000 population in 1990 and 198·4 (180·1–215·4) per 100 000 in 2017 (appendix pp 175, 220). At the regional level, the proportion of deaths due to other causes of cirrhosis was highest in high-income North America (20·6%) and lowest in east Asia (3·8%; figure 4; appendix p 41). At the national level, the highest proportion of deaths was in the UK (30·0%) and the lowest was in China (3·8%) and the United Arab Emirates (3·8%).

**Figure 6:**
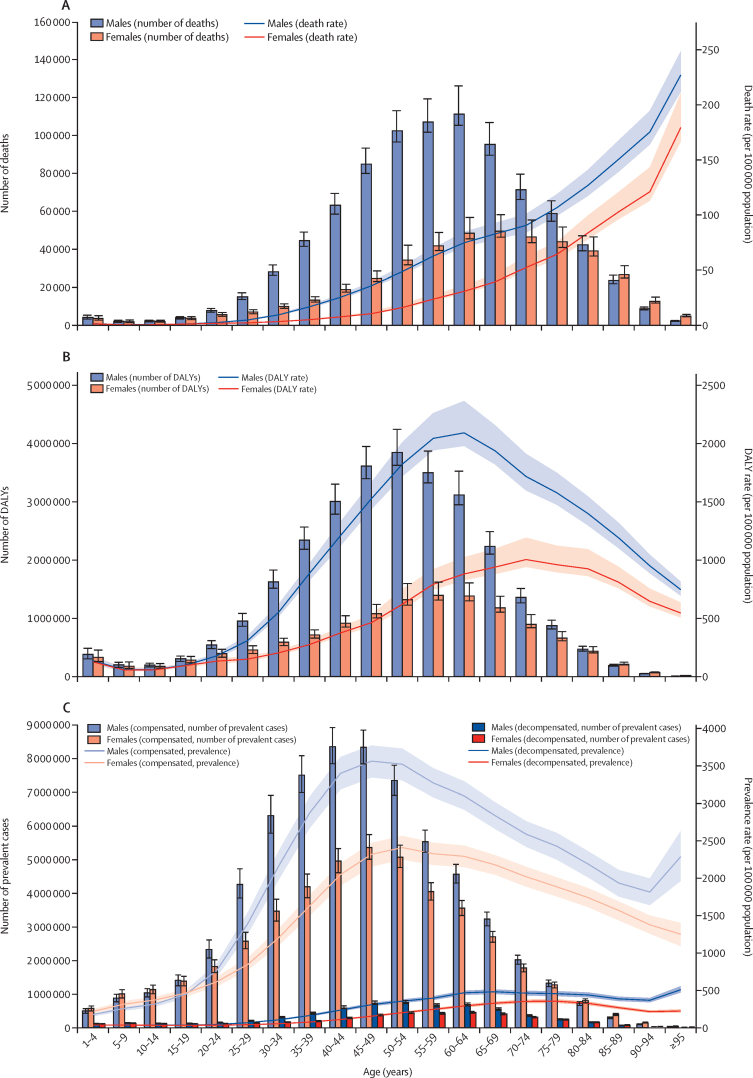
Age patterns of deaths, DALYs, and prevalence of cirrhosis by sex, 2017 (A) Number of deaths and age-specific death rate per 100 000 population. (B) Number of DALYs and age-specific DALY rate per 100 000 population. (C) Number of prevalent cases and age-specific prevalence rates per 100 000 population of decompensated and compensated cirrhosis. Error bars indicate 95% UIs for number of deaths, DALYs, and prevalent cases. Shading indicates 95% UIs for death rates, DALY rates, and prevalence rates. DALYs=disability-adjusted life-years. UI=uncertainty interval.

Patterns in burden by age were similar between males and females and across years from 1990 to 2017, although the numbers and rates were consistently higher in males than in females. The number of deaths peaked at 60–64 years in both sexes combined in the years 1990–2004 and 2011–17, and at 55–59 years for all other years (estimates available through the GBD results tool). In 2017, the number of deaths peaked in the 60–64 year age group for males and the 65–69 year age group for females, whereas death rates increased steadily with age in every year of the study period (figure 6A). In 2017, the number of DALYs peaked at 50–54 years in males and at 55–59 years in females, whereas DALY rates peaked at 60–64 years in males and 70–74 years in females (figure 6B). The number of prevalent cases of compensated cirrhosis peaked at 45–49 years in females and 40–44 years in males in 2017. The prevalence rate of compensated cirrhosis peaked at 45–49 years in males and at 50–54 years in females. The number of prevalent cases of decompensated cirrhosis peaked at 50–54 years in both sexes, whereas the prevalence rates increased up to 65–69 years for males and 75–79 years for females, decreased until 90–94 years for males before increasing again, and decreased through the oldest age groups for females (figure 6C).

Age patterns across regions are provided in the appendix (pp 241–61). In high-income North America in 2017, the number of deaths was highest in the age groups from 50 to 69 years for both sexes combined, with an increasing trend in both the number and rate of deaths in these age groups from 1990 to 2017. In both 1990 and 2017, death rates increased with age, up to and including the 95 years and older age group. The number of cirrhosis deaths in 2017 was also highest in the middle-aged age groups (50–74 years) in all other regions except Oceania (highest in the 40–55 year age groups) and high-income Asia Pacific (highest in the 75–84 year age groups). However, unlike high-income North America, these regions, with the exception of eastern Europe, all experienced a decreasing trend in death rates in the middle-aged age groups over the study period.

The age patterns in central and eastern Europe were markedly different from other regions. Although the number of deaths in these regions peaked at 55–69 years in 2017, similar to other regions, death rates did not consistently increase with age. In 2017, death rates in central Europe declined from 70–74 years to 90–94 years, and in eastern Europe from 65–69 years to 80–84 years and increased again in the oldest age groups in both regions. In all other regions, death rates increased with increasing in age in all years from 1990 to 2017.

**Figure 7:**
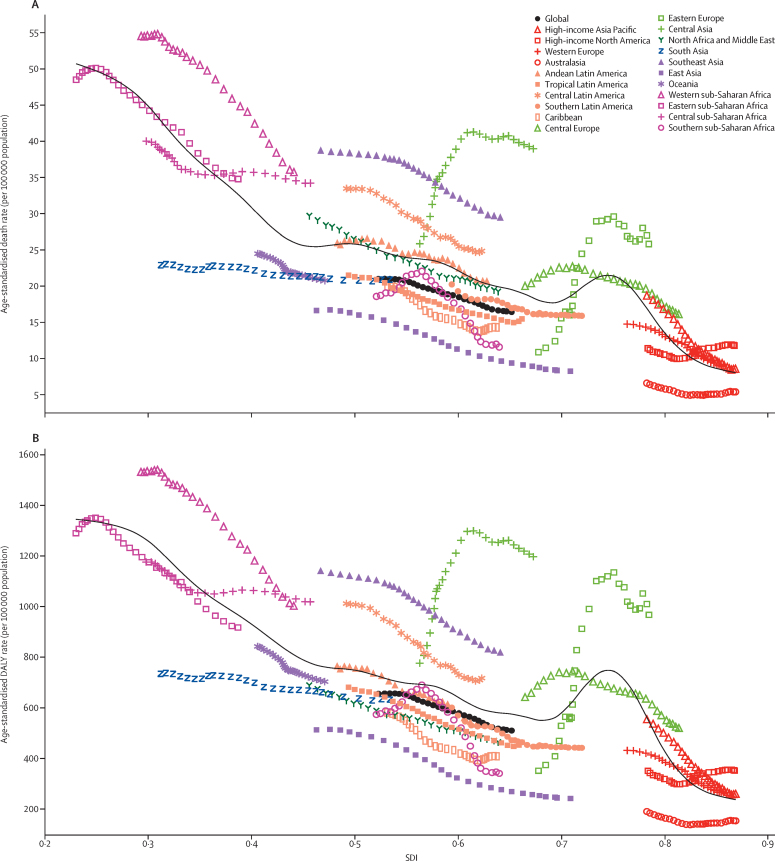

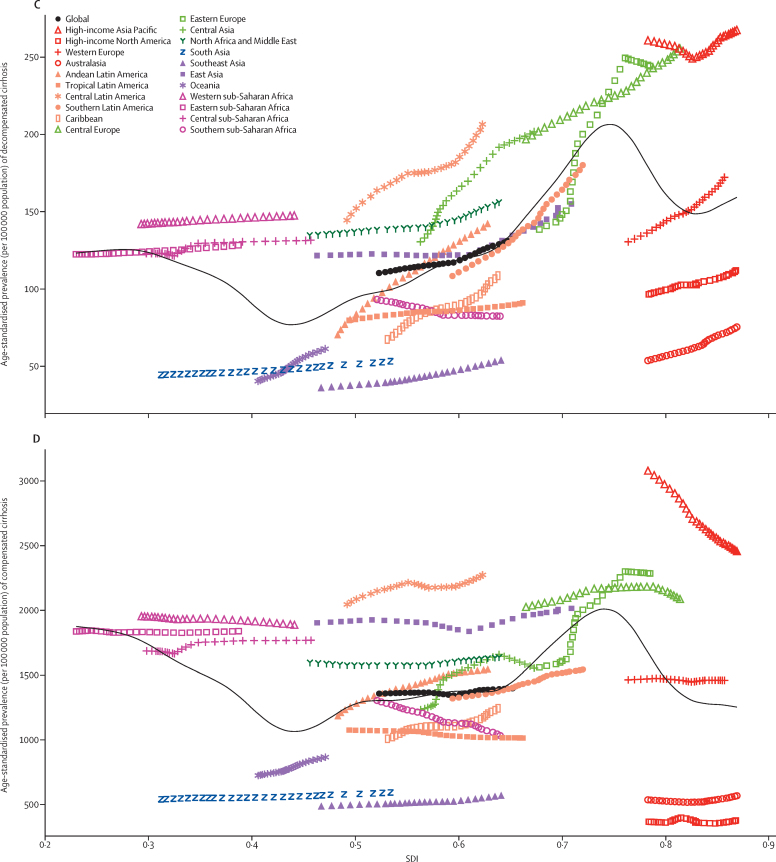
Age-standardised rates of cirrhosis globally and for 21 regions by SDI, 1990–2017 (A) Age-standardised death rate per 100 000 population. (B) Age-standardised DALY rate per 100 0000 population. (C) Age-standardised prevalence per 100 000 population of decompensated cirrhosis. (D) Age-standardised prevalence per 100 000 population of compensated cirrhosis. For each region, points from left to right depict estimates from each year from 1990 to 2017. Black lines show the expected death, DALY, or prevalence rates on the basis of SDI alone. DALYs=disability-adjusted life-years. SDI=Socio-demographic Index.

Between 1990 and 2017, the age-standardised death rate decreased or remained steady in all regions except central Asia and eastern Europe (figure 7A). In these two regions, the observed age-standardised death rates not only increased over the study period, but increased to much higher rates than those that would be expected based solely on SDI. The observed trend in these two regions was an increase in age-standardised death rate from 1990 to the late 2000s, then a slight decrease until 2017. Overall, the age-standardised death rate was lower at the regional level at higher SDI levels, with expected rates following a roughly linear decreasing trend until SDI equalled 0·70, an increase in age-standardised death rate until an SDI of 0·75, then a decline through the highest SDI levels. High and increasing rates in eastern Europe explain the rise in expected rates at 0·70 SDI. At the national level, age-standardised death rates were also lower at higher SDI levels in 2017 (appendix p 262).

The patterns of age-standardised DALY rates versus SDI were similar to those of age-standardised death rates (figure 7B; appendix p 262). In most regions, the observed age-standardised death and DALY rates were close to their expected value on the basis of SDI. The exceptions were western sub-Saharan Africa, southeast Asia, central Asia, and central Latin America, where observed levels were higher than expected in every year of the study period.

Age-standardised prevalence of decompensated cirrhosis was slightly higher at higher SDI levels, although it decreased at an SDI of 0·45 (because of low age-standardised prevalence in Oceania and south Asia) and peaked at an SDI of 0·75 (because of high age-standardised prevalence in central and eastern Europe; figure 7C). Southern sub-Saharan Africa was the only region where age-standardised prevalence of decompensated cirrhosis decreased over time. The pattern of compensated cirrhosis versus SDI was similar to that of decompensated cirrhosis, although most regions showed little change in rate over time. The high-income Asia Pacific region showed a decline in age-standardised prevalence of compensated cirrhosis over the study period, but it was still much higher than expected based on SDI (figure 7D).

## Discussion

In this paper, we described the results of GBD 2017 regarding cirrhosis mortality and morbidity by the four most common causes, and a fifth category of other causes. Of note, the four major causes of cirrhosis mortality and morbidity can generally be prevented by vaccination or lifestyle modification and are readily treatable if diagnosed early enough.^7^ It is essential for national health-care policy makers to know the death and prevalence rates of each cause to be able to plan and implement systematic interventions that prevent premature deaths and morbidity due to cirrhosis.

The age-standardised death, DALY, and prevalence rates were universally lower in females than in males in both 1990 and 2017. Generally, females had a lower proportion of cirrhosis deaths caused by hepatitis B and alcohol-related liver disease than males, and had a higher proportion due to NASH and other causes. This trend could be driven by hormonal factors; lower prevalence of high-risk behaviours; higher prevalence of obesity;^27^ lower consumption of alcohol;^28^ and diseases that almost exclusively affect women, such as autoimmune hepatitis, which has a simple and effective treatment and low mortality if treated in time.^29^

Our results show that in almost all regions, the number of deaths peaked in the middle-aged age groups (approximately 50–74 years) and rates increased steadily with increasing age. Central and eastern Europe were exceptions to these trends, and showed lower death rates in the older age groups (approximately 70–84 years) than in the middle-aged age groups throughout the study period. These findings suggest that cirrhosis incidence in these regions, where cirrhosis deaths are primarily driven by alcohol-related liver disease, was higher in the 1990s, coinciding with peak consumption of alcohol following the dissolution of the Soviet Union, than in 2017.^28^, ^30^ Among high-income regions, high-income North America showed an unusual age pattern, with an increase in death rates in the 50–69 year age group from 1990 to 2017. This finding is compatible with previous reports for the USA and Canada.^31^, ^32^

Our results show that the numbers of deaths and DALYs due to cirrhosis increased globally between 1990 and 2017. The increase in numbers was primarily driven by population growth and ageing across the globe, specifically in low-income and middle-income countries.^26^ By contrast, age-standardised death and DALY rates decreased, and were lower at higher SDI levels. Accordingly, we observed high age-standardised death rates in low-income sub-Saharan African regions in 2017, despite a substantial decline since 1990, which is compatible with previous reports.^33^ However, we observed lower death rates in higher SDI countries and territories (eg, the lowest rate was in Singapore). These findings are expected because most cirrhosis-related deaths can be avoided in high-income countries through better access to health care and stronger health infrastructure. Notable exceptions are countries located in eastern Europe and central Asia, where, unlike in other regions, the age-standardised death rates increased from 1990 to 2017, primarily driven by alcohol-related liver disease, which has also been reported previously.^34^ Similarly, in Latin American regions, a high proportion of deaths were due to alcohol-related liver disease, although age-standardised death rates were not as high as those in eastern Europe and central Asia. These patterns closely follow the distribution of alcohol consumption in these regions.^28^, ^30^

In western Europe, despite low age-standardised death rates, a high proportion of deaths were due to alcohol-related liver disease. This finding is consistent with previous evidence on high alcohol use in these regions^28^, ^30^, ^34^ and demonstrates a need for a systematic approach to reducing alcohol use in these countries. To this end, in 2010, the member states of WHO reached a consensus at the World Health Assembly on a global strategy to confront the harmful use of alcohol.^35^

High-income regions had low age-standardised rates of cirrhosis deaths. Most of these regions, including Australasia, high-income North America, and western Europe, also had low age-standardised prevalences of both compensated and decompensated cirrhosis as a result of concerted efforts to treat and prevent viral hepatitis.^34^, ^36^ The estimated age-standardised prevalence and death rates caused by cirrhosis in this study were slightly higher but generally compatible with previous reports from the USA and released by the US Centers for Disease Control and Prevention.^37^ The estimated age-standardised death rates in the UK were also consistent with previous reports.^38^

The high-income Asia Pacific countries, especially Japan and South Korea, had a high but declining age-standardised prevalence rate of compensated cirrhosis, despite having low mortality rates.^39^ Prevalence trends could indicate a high incidence in preceding decades followed by a recent decline in mortality because of improved health care and access to affordable and effective antiviral treatment. Accordingly, in 2005, the WHO Western Pacific region set a goal to reduce HBsAg seroprevalence in children 5 years of age and older to less than 2% by 2012. A supranational goal was selected to create a sense of political urgency to establish routine immunisation and improve access to health care.^39^ A similar approach might be successful in African and other Asian countries, as well as in the region of north Africa and the Middle East, to control the incidence of hepatitis B; cirrhosis deaths in these regions were mainly due to hepatitis B and C.

Southeast Asia had higher age-standardised death rates than expected, and south Asia and Oceania had modest death rates, but these three regions were among those with the lowest age-standardised prevalence of compensated and decompensated cirrhosis. Mortality was primarily due to hepatitis B in these regions. The low age-standardised prevalence of compensated cirrhosis in these regions might be because of a low detection rate, whereas the low age-standardised prevalence of decompensated cirrhosis might be because of a relatively poor medical infrastructure, leading to a short survival time once the cirrhosis starts to decompensate.^40^

In north Africa and the Middle East, alcohol-related liver disease constituted the lowest proportion of age-standardised prevalence and death rates.^19^ This finding could be expected, because alcohol is prohibited in many of the countries in this region, which could lead to both decreased use and the possibility of under-reporting. Instead, hepatitis B and C constitute larger proportions. Despite a decrease in age-standardised death rates in this region, hepatitis B is still the major cause of cirrhosis deaths, and although most countries in these regions have nationwide infant vaccination programmes for hepatitis B, none have been in place for more than 30 years.^41^ Most of the mortality of hepatitis B is in patients older than 40 years, so we expect hepatitis B to remain a major cause of cirrhosis death for another decade or two, even in countries with good vaccine coverage.

Hepatitis C, by contrast, now has an effective cure, which should have an impact much sooner. With the increased availability of cheap generic anti-hepatitis C virus medicines,^42^ we expect the death rate for hepatitis C to rapidly decrease in the near future, if countries are able to meet the goals set by WHO to eliminate the burden of hepatitis C by 2030.^43^ For example, in Egypt, which currently has the highest death rate due to hepatitis C,^19^ we expect a rapid decrease in the hepatitis C death rate within the next 5–10 years because of an active patient-finding and treatment programme enacted by the government in 2014.^44^ Australia has also been successful in finding and treating patients with hepatitis C and will probably have very low hepatitis C death rates in the near future.^45^

Between 1990 and 2017, we observed a doubling in the number of deaths, a more than tripling in prevalent cases of decompensated cirrhosis, and a more than doubling in prevalent cases of compensated cirrhosis due to NASH. Unlike the other four causes studied, NASH was the only one not to show a decreasing trend in age-standardised death rates. The highest proportions of deaths due to NASH were in Latin America and north Africa and the Middle East, and the lowest was in high-income Asia Pacific, as previously reported.^46^ The epidemiology of NASH closely follows the distribution of overweight and obesity and components of metabolic syndrome in these regions.^27^, ^47^ Because of the absence of practical means of preventing or treating NASH and a global increase in metabolic syndrome and obesity, we should expect an increase in the burden of this disease.^16^, ^17^

Despite the availability of an effective vaccine for hepatitis B for decades, the availability of an effective treatment for hepatitis C, and an increase in obesity worldwide, the proportional contribution of different causes to cirrhosis remained fairly constant between 1990 and 2017 at the global level. The development of cirrhosis and its progression to decompensation takes decades, and it might still be too early to see the effects of vaccination coverage or increased prevalence of obesity and metabolic syndrome.

The limitations of the methods of GBD impose biases on our estimates in the current study, as with all GBD research. The most important limitation is the low availability and low quality of data, although we used robust statistical methods to overcome data scarcity in countries with low data availability. In these locations, we relied on predictive covariates, trends in neighbouring countries, or a combination of these. The wide variation in the availability of high-quality data across locations is reflected in the uncertainty associated with all estimates. As a result of lower availability of population-based data on the prevalence of cirrhosis, dependence on hospital and claims data, and various definitions and diagnostic criteria for decompensated and compensated cirrhosis, it is possible that both types, especially compensated cirrhosis, have been underestimated. We made the assumption that patients admitted to hospitals had decompensated cirrhosis and that total cases detected upon admission constituted all cases of cirrhosis. Of note, although ICD-10 codes have been validated for overall cirrhosis and chronic liver diseases, detailed codes for the five causes have not been fully validated. To address this challenge, we combined the aetiological models for cirrhosis and liver cancer, as these two diseases share the same causes. Aetiological proportion models of liver cancer were used as covariates for cirrhosis aetiological proportion models. Finally, advances in imaging modalities and the availability of liver biopsy as the gold standard for detecting cirrhosis in population-based studies can considerably improve the accuracy of estimations.

Further work is needed to disentangle the separate entities that were categorised as other causes in this study, including autoimmune hepatitis as a sixth cause for cirrhosis. A detailed study on the epidemiology of NASH, particularly in north Africa and the Middle East and Latin America, is needed because it disproportionately affects age-standardised death rate as compared with other causes. Future studies should focus on evaluating common risk factors, dietary patterns, and screening practices affecting the incidence, mortality, and prevalence of cirrhosis by age, sex, and race, especially among countries and territories with the highest numbers of deaths. The financial burden of cirrhosis also merits attention in future studies.

In conclusion, the major causes of cirrhosis are preventable and treatable; however, setting cost-effective policies to prevent and treat cirrhosis requires high-quality, localised data. GBD provides the most up-to-date estimates on the burden of cirrhosis by cause to guide policy makers in designing effective preventive plans and implementing interventions at national and even subnational levels. In line with the Global Health Sector Strategy on Viral Hepatitis 2016 to 2021, the Sustainable Development Goals, and the WHO Global Strategy to Reduce Harmful Use of Alcohol, there is a need to implement comprehensive prevention efforts to achieve a sustained reduction in cirrhosis burden.
