# Supplementary material for: The global, regional, and national burden of cirrhosis by cause in 195 countries and territories, 1990–2017: a systematic analysis for the Global Burden of Disease Study 2017
Source: Lancet Gastroenterol Hepatol. 2020 Jan 22;5(3):245–66. doi: 10.1016/S2468-1253(19)30349-8 (PMC7026710; doi:10.1016/S2468-1253(19)30349-8)

THE LANCET
Gastroenterology & Hepatology

Supplementary appendix

This appendix formed part of the original submission and has been peer reviewed. We post it as supplied by the authors.

Supplement to: GBD 2017 Cirrhosis Collaborators. The global, regional, and national burden of cirrhosis by cause in 195 countries and territories, 1990–2017: a systematic analysis for the Global Burden of Disease Study 2017. *Lancet Gastroenterol Hepatol* 2020; published online Jan 22. https://doi.org/10.1016/S2468-1253(19)30349-8.

Supplementary appendix

Supplement to: **Global, regional, and national burden of cirrhosis by cause in 195 countries and territories, 1990–2017: a systematic analysis for the Global Burden of Disease Study 2017**

Contents:

Appendix Tables

Appendix Table 1: ICD-10 codes mapped to GBD cause list for cirrhosis. GBD=Global Burden of Disease.

Appendix Table 2: Number of site-years of data in GBD. GBD=Global Burden of Disease.

Appendix Table 3: Deaths and age-standardised death rates due to cirrhosis in 1990 and 2017 for both sexes and percentage change of age-standardised rates per 100 000 by location

Appendix Table 4: DALYs and age-standardised DALY rates due to cirrhosis in 1990 and 2017 for both sexes and percentage change of age-standardised rates per 100 000 by location. DALYs=disability-adjusted life-years.

Appendix Table 5: Prevalent cases and age-standardised prevalence rates of decompensated cirrhosis per 100 000 in 1990 and 2017 for both sexes combined by location

Appendix Table 6: Prevalent cases and age-standardised prevalence rates of compensated cirrhosis per 100 000 in 1990 and 2017 for both sexes combined by location

Appendix Table 7: Proportion of causes for deaths due to cirrhosis in 2017 in both sexes

Appendix Table 8: Proportion of causes for prevalent cases of decompensated cirrhosis in 2017 in both sexes

Appendix Table 9: Proportion of causes for prevalent cases of compensated cirrhosis in 2017 in both sexes

Appendix Table 10: Proportion of causes for DALYs due to cirrhosis in 2017 in both sexes. DALYs=disability-adjusted life-years.

Appendix Table 11: Number of deaths and age-standardised death rates of cirrhosis due to hepatitis B per 100 000 in 1990 and 2017 for both sexes and the percentage change by location

Appendix Table 12: Number of deaths and age-standardised death rates of cirrhosis due to hepatitis C per 100 000 in 1990 and 2017 for both sexes and the percentage change by location

Appendix Table 13: Number of deaths and age-standardised death rates of cirrhosis due to alcohol-related liver disease per 100 000 in 1990 and 2017 for both sexes and the percentage change by location

Appendix Table 14: Number of deaths and age-standardised death rates of cirrhosis due to non-alcoholic steatohepatitis (NASH) per 100 000 in 1990 and 2017 for both sexes and the percentage change by location

Appendix Table 15: Number of deaths and age-standardised death rates of cirrhosis due to other causes per 100 000 in 1990 and 2017 for both sexes and the percentage change by location

Appendix Table 16: Number of prevalent cases and age-standardised prevalence rates of decompensated cirrhosis due to hepatitis B per 100 000 in 1990 and 2017 for both sexes by location

Appendix Table 17: Number of prevalent cases and age-standardised prevalence rates of decompensated cirrhosis due to hepatitis C per 100 000 in 1990 and 2017 for both sexes by location

Appendix Table 18: Number of prevalent cases and age-standardised prevalence rates of decompensated cirrhosis due to alcohol-related liver disease per 100 000 in 1990 and 2017 for both sexes by location

Appendix Table 19: Number of prevalent cases and age-standardised prevalence rates of decompensated cirrhosis due to non-alcoholic steatohepatitis (NASH) per 100 000 in 1990 and 2017 for both sexes by location

Appendix Table 20: Number of prevalent cases and age-standardised prevalence rates of decompensated cirrhosis due to other causes per 100 000 in 1990 and 2017 for both sexes by location

Appendix Table 21: Number of prevalent cases and age-standardised prevalence rates of compensated cirrhosis due to hepatitis B per 100 000 in 1990 and 2017 for both sexes by location

Appendix Table 22: Number of prevalent cases and age-standardised prevalence rates of compensated cirrhosis due to hepatitis C per 100 000 in 1990 and 2017 for both sexes by location

Appendix Table 23: Number of prevalent cases and age-standardised prevalence rates of compensated cirrhosis due to alcohol-related liver disease per 100 000 in 1990 and 2017 for both sexes by location

Appendix Table 24: Number of prevalent cases and age-standardised prevalence rates of compensated cirrhosis due to non-alcoholic steatohepatitis (NASH) per 100 000 in 1990 and 2017 for both sexes by location

Appendix Table 25: Number of prevalent cases and age-standardised prevalence rates of compensated cirrhosis due to other causes per 100 000 in 1990 and 2017 for both sexes by location

Appendix Table 26: Data quality rating from 0 to 5 stars, maximum percentage well-certified per five-year interval and percentage well-certified across time series for 195 countries and territories, 1980–2017

Appendix Table 27: Parent cirrhosis CODEm model covariate table

Appendix Figures

Appendix Figure 1: Trends in age-standardised death rates from 1990 to 2017 for both sexes across seven super-regions

Appendix Figure 2: The trend of age-standardised death rate of cirrhosis due to hepatitis B across regions from 1990 to 2017, versus the levels of SDI. The black line represents expected rates based solely on SDI. For each region, points from left to right depict estimates for each year from 1990 to 2017. SDI=Socio-demographic Index.

Appendix Figure 3: The trend of age-standardised death rate of cirrhosis due to hepatitis C across regions from 1990 to 2017, versus the levels of SDI. The black line represents expected rates based solely on SDI. For each region, points from left to right depict estimates for each year from 1990 to 2017. SDI=Socio-demographic Index.

Appendix Figure 4: The trend of age-standardised death rate of cirrhosis due to alcohol-related liver disease across regions from 1990 to 2017, versus the levels of SDI. The black line represents expected rates based solely on SDI. For each region, points from left to right depict estimates for each year from 1990 to 2017. SDI=Socio-demographic Index.

Appendix Figure 5: The trend of age-standardised death rate of cirrhosis due to NASH across regions from 1990 to 2017, versus the levels of SDI. NASH=non-alcoholic steatohepatitis. The black line represents expected rates based solely on SDI. For each region, points from left to right depict estimates for each year from 1990 to 2017. SDI=Socio-demographic Index.

Appendix Figure 6: The trend of age-standardised death rate of cirrhosis due to other causes across regions from 1990 to 2017, versus the levels of SDI. The black line represents expected rates based solely on SDI. For each region, points from left to right depict estimates for each year from 1990 to 2017. SDI=Socio-demographic Index.

Appendix Figure 7: The counts and age-standardised rates of mortality by cause across age groups in 2017

Appendix Figure 8: The proportion of YLLs and YLDs out of DALYs by age in 2017. YLLs=years of life lost due to premature death. YLDs=years lived with disability. DALYs=disability-adjusted life-years.

Appendix Figure 9: The age pattern of numbers and rates of death at regional level in 1990, 2000, 2010, and 2017.

Appendix Figure 10: The trend of age-standardised rates of A) deaths, B) DALYs due to cirrhosis across nations in 2017, versus levels of SDI. DALYs=disability-adjusted life-years. SDI=Socio-demographic Index.

Appendix Table 1: ICD-10 codes mapped to GBD cause list for cirrhosis

ICD-10 code	Definition	GBD cirrhosis cause
**B18**	**Chronic viral hepatitis**	
B18.0	Chronic viral hepatitis B with delta-agent	Cirrhosis due to hepatitis B
B18.1	Chronic viral hepatitis B without delta-agent	
B18.2	Chronic viral hepatitis C	Cirrhosis due to hepatitis C
B18.8	Other chronic viral hepatitis	Cirrhosis due to other causes
B18.9	Chronic viral hepatitis, unspecified	
**K70**	**Alcoholic liver disease**	Alcoholic liver disease
K70.0	Alcoholic fatty liver	
K70.1	Alcoholic hepatitis	
K70.10	Alcoholic hepatitis without ascites	
K70.11	Alcoholic hepatitis with ascites	
K70.2	Alcoholic fibrosis and sclerosis of liver	
K70.3	Alcoholic cirrhosis of liver	
K70.30	Alcoholic cirrhosis of liver without ascites	
K70.31	Alcoholic cirrhosis of liver with ascites	
K71.7	Toxic liver disease with fibrosis and cirrhosis of liver	Cirrhosis due to other causes
**K73**	**Chronic hepatitis, not elsewhere classified**	
K73.0	Chronic persistent hepatitis, not elsewhere classified	
K73.1	Chronic lobular hepatitis, not elsewhere classified	
K73.2	Chronic active hepatitis, not elsewhere classified	
K73.8	Other chronic hepatitis, not elsewhere classified	
K73.9	Chronic hepatitis, unspecified	
K75.2	Nonspecific reactive hepatitis	
K75.4	Autoimmune hepatitis	
K75.8	Other specified inflammatory liver diseases	
K75.81	Nonalcoholic steatohepatitis (NASH)	Cirrhosis due to NASH
K75.89	Other specified inflammatory liver diseases	Cirrhosis due to other causes
K75.9	Inflammatory liver disease, unspecified	
**K76**	**Other diseases of liver**	
K76.1	Chronic passive congestion of liver	
K76.2	Central hemorrhagic necrosis of liver	
K76.4	Peliosis hepatitis	
K76.5	Hepatic veno-occlusive disease	
K76.6	Portal hypertension	
K76.7	Hepatorenal syndrome	
K76.8	Other specified diseases of liver	
K76.81	Hepatopulmonary syndrome	
K76.89	Other specified diseases of liver	
K76.9	Liver disease, unspecified	
K77.8	Liver disorders in other diseases classified elsewhere	

ICD=International Classification of Diseases. GBD=Global Burden of Disease.

Appendix Table 2. Number of site-years of data in GBD

	Hepatitis B	Hepatitis C	Alcohol-related liver disease	NASH	Other
**Site-years (total)**	76	80	55	24	34
**Number of countries with data**	32	35	23	14	18
**Number of GBD super-regions with data (n=7)**	7	7	6	5	6
**Number of GBD regions with data (n=21)**	17	17	13	10	11

GBD=Global Burden of Disease. NASH=non-alcoholic steatohepatitis.

Appendix Table 3: Deaths and age-standardised death rates due to cirrhosis in 1990 and 2017 for both sexes and percentage change of age-standardised rates per 100 000 by location

	1990		2017		Percentage change in age-standardised rates between 1990 and 2017
	Counts (95% UI)	Rate (95% UI)	Counts (95% UI)	Rate (95% UI)	
**Global**	898991 (828934, 948209)	21 (19·2, 22·3)	1322868 (1268197, 1449133)	16·5 (15·8, 18·1)	-21·7 (-28·2, -12·2)
**High-income North America**	38759 (37543, 39457)	11·5 (11·1, 11·7)	67346 (65130, 69616)	11·8 (11·4, 12·2)	3·2 (-0·4, 7·8)
**Canada**	2944 (2835, 3042)	9 (8·7, 9·3)	4845 (4492, 5234)	7·5 (7, 8·1)	-16·7 (-23, -9·7)
**Greenland**	4 (4, 5)	9·4 (8, 11·3)	7 (5, 8)	9·2 (7, 10·5)	-1·1 (-30·7, 26·8)
**USA**	35810 (34608, 36500)	11·7 (11·4, 12)	62493 (60414, 64625)	12·3 (11·9, 12·8)	5·2 (1·2, 10·2)
**Australasia**	1534 (1468, 1579)	6·6 (6·4, 6·8)	2479 (2234, 2744)	5·4 (4·9, 6)	-18·2 (-26·7, -8·4)
**Australia**	1339 (1269, 1383)	6·9 (6·6, 7·2)	2187 (1953, 2450)	5·7 (5·1, 6·4)	-17·7 (-27·2, -6·7)
**New Zealand**	195 (184, 208)	5 (4·8, 5·4)	292 (270, 319)	3·9 (3·6, 4·3)	-22·3 (-28·5, -15·4)
**High-income Asia Pacific**	37974 (37236, 38668)	18·7 (18·3, 19·1)	35078 (32398, 37041)	8·6 (7·9, 9·1)	-54·1 (-58, -51·2)
**Brunei**	14 (12, 16)	11·3 (9·7, 12·8)	25 (21, 31)	6·6 (5·7, 9·2)	-41·3 (-52·6, -16·1)
**Japan**	22684 (22208, 23074)	13·4 (13·1, 13·7)	25352 (23653, 26613)	7·6 (7, 8)	-43·6 (-47·4, -40·7)
**Singapore**	193 (182, 202)	8·1 (7·6, 8·4)	254 (227, 280)	3·7 (3·3, 4)	-54·8 (-59·5, -50·4)
**South Korea**	15083 (14611, 15612)	43·9 (42·3, 45·3)	9447 (8152, 10528)	11·2 (9·7, 12·5)	-74·4 (-78, -71·1)
**Western Europe**	81430 (80035, 82972)	14·8 (14·6, 15·1)	74230 (70803, 78164)	9·1 (8·7, 9·6)	-38·7 (-41·6, -35·4)
**Andorra**	6 (5, 8)	10·7 (7·9, 13·6)	10 (8, 13)	7·5 (5·6, 9·7)	-29·8 (-47·5, -7·3)
**Austria**	2287 (2226, 2359)	21·2 (20·6, 21·9)	1860 (1710, 2002)	11·6 (10·7, 12·6)	-45·1 (-49·7, -40)
**Belgium**	1702 (1635, 1782)	11·7 (11·3, 12·2)	2004 (1832, 2171)	9·7 (8·8, 10·5)	-17·1 (-24·6, -9·9)
**Cyprus**	90 (71, 115)	11 (8·8, 14·2)	123 (106, 141)	6·6 (5·7, 7·6)	-40·3 (-55·3, -22·6)
**Denmark**	734 (704, 771)	10·6 (10·2, 11·1)	947 (869, 1030)	9·5 (8·7, 10·3)	-10·7 (-19·3, -0·8)
**Finland**	572 (545, 612)	8·6 (8·2, 9·1)	1178 (1073, 1283)	12·5 (11·4, 13·7)	45·7 (29, 62·6)

**France**	13236 (12799, 13868)	17·1 (16·6, 17·9)	10621 (9656, 11575)	8·9 (8·1, 9·8)	-47·7 (-53·3, -42·6)
**Germany**	20334 (19655, 20935)	17·1 (16·6, 17·6)	19558 (17400, 21904)	11·9 (10·6, 13·3)	-30·5 (-38·4, -22)
**Greece**	1598 (1521, 1664)	10·5 (10, 10·9)	1308 (1204, 1428)	5·9 (5·5, 6·4)	-43·8 (-48·6, -38·5)
**Iceland**	12 (11, 13)	4·4 (4·1, 4·7)	19 (17, 21)	3·8 (3·5, 4·1)	-14·5 (-23, -5·1)
**Ireland**	190 (180, 200)	4·8 (4·6, 5)	393 (351, 435)	5·7 (5·1, 6·3)	18·9 (4·5, 32·6)
**Israel**	393 (373, 416)	8·1 (7·7, 8·6)	707 (651, 774)	6·2 (5·7, 6·9)	-23·1 (-29·5, -15·8)
**Italy**	17688 (17129, 18206)	20·1 (19·5, 20·7)	11695 (10621, 12746)	8·2 (7·4, 9)	-59·1 (-62·8, -55·3)
**Luxembourg**	93 (89, 99)	17·6 (16·8, 18·6)	99 (83, 114)	10·7 (8·9, 12·4)	-39·3 (-49·4, -29)
**Malta**	35 (32, 37)	8·1 (7·5, 8·6)	44 (40, 47)	5·5 (5, 5·9)	-32·4 (-39·3, -24·5)
**Netherlands**	1382 (1327, 1445)	7 (6·8, 7·4)	1681 (1554, 1851)	5·1 (4·7, 5·6)	-27·5 (-33·1, -21·2)
**Norway**	345 (334, 361)	5·7 (5·5, 5·9)	373 (341, 397)	4·1 (3·8, 4·4)	-27 (-34·1, -21·8)
**Portugal**	3541 (3438, 3639)	26·7 (26, 27·5)	2133 (1940, 2351)	10·4 (9·4, 11·5)	-61·1 (-65, -57·1)
**Spain**	10690 (10359, 11025)	20 (19·4, 20·6)	8132 (7510, 8833)	8·9 (8·1, 9·6)	-55·7 (-59·5, -51·7)
**Sweden**	827 (797, 864)	6·1 (5·9, 6·4)	1071 (995, 1146)	5·6 (5·2, 6)	-8·6 (-15, -1·6)
**Switzerland**	806 (767, 847)	8·2 (7·8, 8·6)	940 (851, 1041)	5·8 (5·2, 6·5)	-28·8 (-37·1, -19·3)
**UK**	4788 (4590, 4875)	5·8 (5·6, 5·9)	9258 (9007, 9675)	8·7 (8·5, 9)	48·6 (43·6, 58·8)
**Southern Latin America**	9586 (9335, 9883)	20·3 (19·7, 20·9)	12822 (11686, 14020)	15·9 (14·5, 17·4)	-21·4 (-28·4, -14·1)
**Argentina**	5360 (5156, 5549)	16·4 (15·8, 17)	7346 (6448, 8262)	14·1 (12·3, 15·9)	-14·1 (-25·6, -2·7)
**Chile**	3718 (3578, 3943)	34·6 (33·3, 36·7)	4980 (4399, 5603)	21·5 (18·9, 24·1)	-38·1 (-45·2, -29·7)
**Uruguay**	507 (478, 531)	13·1 (12·4, 13·8)	495 (438, 562)	9·4 (8·3, 10·8)	-28·2 (-37·1, -18·3)
**Eastern Europe**	30564 (29058, 31582)	10·9 (10·3, 11·3)	78289 (75809, 80578)	25·9 (25, 26·7)	137 (129·5, 146·2)
**Belarus**	865 (796, 927)	6·7 (6·1, 7·1)	2533 (2230, 2816)	17·5 (15·4, 19·4)	162·5 (122, 198·9)

**Estonia**	140<br>(132, 157)	7<br>(6·6, 7·8)	292<br>(251, 337)	14·4<br>(12·3, 16·7)	105·8<br>(69·3, 143·2)
**Latvia**	244<br>(231, 273)	6·9<br>(6·5, 7·6)	434<br>(385, 491)	14<br>(12·3, 15·8)	104·1<br>(75·8, 134·4)
**Lithuania**	351<br>(332, 372)	7·8<br>(7·4, 8·3)	942<br>(872, 1020)	21·7<br>(20, 23·5)	176·7<br>(154·1, 203·2)
**Moldova**	3187<br>(3082, 3290)	67·5<br>(65·4, 69·6)	3094<br>(2914, 3274)	55·6<br>(52·3, 58·7)	-17·7<br>(-24, -12·2)
**Russia**	17451<br>(16015, 18377)	9·6<br>(8·8, 10·1)	50910<br>(49591, 52079)	24·3<br>(23·7, 24·9)	152·3<br>(140·3, 176·1)
**Ukraine**	8324<br>(8024, 8709)	11·9<br>(11·4, 12·4)	20083<br>(18374, 21669)	31·7<br>(28·9, 34·3)	167·2<br>(140·9, 191)
**Central Europe**	30083<br>(29453, 30901)	20<br>(19·6, 20·6)	30986<br>(29825, 32149)	16·2<br>(15·5, 16·8)	-19·4<br>(-22·7, -15·9)
**Albania**	243<br>(203, 263)	11<br>(8·8, 12)	309<br>(247, 414)	7·7<br>(6·1, 10·3)	-30·5<br>(-46·5, 2·3)
**Bosnia and Herzegovina**	744<br>(696, 842)	16·6<br>(15·5, 18·3)	577<br>(499, 808)	10<br>(8·6, 13·9)	-40<br>(-48·1, -22·2)
**Bulgaria**	1867<br>(1792, 1939)	14·9<br>(14·4, 15·5)	2043<br>(1879, 2213)	16·4<br>(15, 17·7)	9·6<br>(-0·5, 20·2)
**Croatia**	1756<br>(1694, 1829)	26·7<br>(25·8, 27·8)	1095<br>(1008, 1185)	13·8<br>(12·7, 14·9)	-48·5<br>(-52·6, -44)
**Czech Republic**	2174<br>(2085, 2304)	16·3<br>(15·7, 17·3)	2175<br>(2016, 2366)	12<br>(11·1, 13·1)	-26·3<br>(-33·4, -18·9)
**Hungary**	5941<br>(5786, 6112)	42·7<br>(41·6, 43·9)	3228<br>(2995, 3459)	19·1<br>(17·8, 20·5)	-55·2<br>(-58·5, -51·7)
**Montenegro**	37<br>(32, 43)	5·7<br>(4·8, 6·5)	49<br>(41, 58)	5·2<br>(4·3, 6·1)	-9·2<br>(-26·1, 9·2)
**North Macedonia**	152<br>(137, 179)	7·8<br>(7, 9)	235<br>(196, 266)	7·1<br>(6, 8·1)	-8<br>(-24·4, 7·1)
**Poland**	4827<br>(4594, 5099)	10·8<br>(10·3, 11·4)	7788<br>(7213, 8462)	12·8<br>(11·8, 13·9)	18·8<br>(6·8, 30·9)
**Romania**	8379<br>(8075, 8658)	29·3<br>(28·2, 30·3)	10114<br>(9419, 10805)	30·1<br>(28·1, 32·2)	2·8<br>(-4·4, 10·4)
**Serbia**	1520<br>(1216, 1773)	12·6<br>(10·2, 14·6)	1304<br>(1172, 1473)	8·9<br>(8, 10)	-29·8<br>(-41·9, -12·5)
**Slovakia**	1654<br>(1477, 2039)	28<br>(25, 34·1)	1527<br>(1248, 1713)	17·9<br>(14·5, 20)	-36·1<br>(-53·8, -22·6)
**Slovenia**	787<br>(757, 820)	31·4<br>(30·2, 32·7)	541<br>(490, 593)	14·3<br>(12·9, 15·6)	-54·5<br>(-58·9, -50·1)
**Central Asia**	13061<br>(12777, 13479)	25·9<br>(25·3, 26·7)	30863<br>(28655, 32907)	39<br>(36·2, 41·5)	50·5<br>(38·6, 59·9)
**Armenia**	413<br>(391, 442)	14·8<br>(14, 15·9)	1107<br>(1040, 1195)	27·3<br>(25·6, 29·5)	84·7<br>(71·5, 97·7)

**Azerbaijan**	1406 (1268, 1499)	26·8 (23·5, 28·5)	3141 (2596, 3726)	36·2 (28·7, 43·7)	35·1 (13·9, 66)
**Georgia**	1475 (1404, 1553)	23·5 (22·4, 24·7)	1381 (1256, 1553)	25·6 (23·3, 28·6)	8·9 (-1·3, 21·8)
**Kazakhstan**	2171 (2068, 2277)	16·2 (15·5, 17)	6849 (5700, 7689)	38·6 (32·4, 43·2)	137·6 (97·9, 169·9)
**Kyrgyzstan**	1060 (1007, 1115)	33·3 (31·7, 35·1)	2159 (1918, 2336)	42·7 (38·3, 46)	28·3 (13·2, 40·2)
**Mongolia**	717 (648, 811)	62·3 (56·4, 69·6)	1374 (1197, 1545)	62·3 (50, 70·4)	0 (-21·6, 16·1)
**Tajikistan**	818 (752, 980)	24·8 (22·8, 30·8)	1536 (1369, 1734)	25·2 (22·4, 29·5)	1·5 (-9·6, 13·6)
**Turkmenistan**	842 (804, 871)	38·6 (36·8, 40)	2341 (2139, 2548)	51·8 (47·6, 56·1)	34 (21·3, 47·9)
**Uzbekistan**	4159 (4032, 4297)	32·6 (31·6, 33·8)	10974 (9762, 12331)	43·7 (39, 48·8)	33·9 (18·8, 50·3)
**Central Latin America**	32018 (31379, 32519)	33·5 (32·8, 34·1)	59053 (56628, 62086)	24·9 (23·9, 26·2)	-25·7 (-29·3, -21·5)
**Colombia**	1953 (1857, 2032)	10·5 (9·9, 10·9)	3886 (3437, 4420)	7·2 (6·4, 8·2)	-31·3 (-38·9, -21·8)
**Costa Rica**	309 (296, 322)	16·5 (15·9, 17·2)	840 (759, 947)	16·9 (15·3, 19·1)	2·4 (-7·8, 16·2)
**El Salvador**	914 (765, 975)	27·5 (23·3, 29·3)	1480 (1207, 1925)	25·9 (21·1, 33·7)	-5·8 (-25·1, 32·7)
**Guatemala**	2064 (1970, 2146)	44·6 (42, 46·7)	4623 (4146, 5142)	39·2 (35·3, 43·5)	-12 (-21·7, 0·1)
**Honduras**	982 (832, 1277)	38·5 (32·4, 50·9)	2388 (1795, 3236)	37·8 (28, 51·4)	-2 (-23·8, 22·3)
**Mexico**	23104 (22664, 23480)	47·1 (46·2, 47·8)	40509 (39068, 42316)	34·6 (33·3, 36·1)	-26·6 (-29·8, -22·8)
**Nicaragua**	427 (387, 467)	23·3 (20·9, 25·4)	1127 (815, 1306)	23·4 (17, 27)	0·8 (-23·1, 17·1)
**Panama**	203 (190, 216)	13·2 (12·3, 14)	455 (420, 495)	11·5 (10·6, 12·5)	-12·9 (-21·3, -3·4)
**Venezuela**	2062 (1975, 2148)	19·3 (18·4, 20·2)	3745 (3154, 4446)	13 (11, 15·3)	-32·7 (-43·4, -20)
**Andean Latin America**	6021 (5530, 6745)	26 (24, 29·5)	11234 (10164, 12321)	20·7 (18·7, 22·7)	-20·3 (-30·4, -9·9)
**Bolivia**	1102 (801, 1501)	31·1 (23, 41·3)	2215 (1727, 2813)	25·9 (20·3, 32·8)	-16·6 (-35·2, 12·5)
**Ecuador**	1421 (1367, 1476)	23·7 (22·8, 24·6)	3457 (3119, 3821)	23·6 (21·3, 26·1)	-0·4 (-10·6, 11·4)
**Peru**	3498 (3169, 3888)	25·7 (23·2, 28·9)	5562 (4646, 6508)	18·1 (15·1, 21·1)	-29·7 (-42·4, -15·3)

**Caribbean**	5453<br>(4998, 5984)	20<br>(18·3, 22)	7294<br>(6490, 8619)	14·3<br>(12·8, 17)	-28·4<br>(-35·1, -11·4)
**Antigua and Barbuda**	8<br>(8, 9)	16·5<br>(15·4, 17·8)	11<br>(10, 12)	11<br>(10·1, 12)	-33·6<br>(-40·4, -25·1)
**The Bahamas**	38<br>(36, 41)	22·5<br>(20·9, 24·1)	54<br>(48, 60)	13·7<br>(12·3, 15·2)	-39·2<br>(-46·6, -31)
**Barbados**	35<br>(33, 38)	12·7<br>(11·8, 13·5)	39<br>(35, 43)	8·3<br>(7·5, 9·3)	-34·4<br>(-42·1, -25·8)
**Belize**	22<br>(20, 23)	21·4<br>(19·3, 23·4)	57<br>(52, 62)	20·4<br>(18·5, 22·2)	-4·5<br>(-13·4, 6·1)
**Bermuda**	9<br>(8, 10)	13·7<br>(12·5, 15·1)	7<br>(6, 8)	5·8<br>(5·2, 6·7)	-57·8<br>(-63·7, -48·7)
**Cuba**	1110<br>(1059, 1192)	10·6<br>(10·1, 11·4)	1869<br>(1636, 2150)	10·3<br>(9, 11·8)	-2·7<br>(-13·8, 9·7)
**Dominica**	10<br>(9, 10)	13·9<br>(12·9, 14·9)	10<br>(9, 11)	11<br>(10, 12·1)	-20·7<br>(-29·4, -11·1)
**Dominican Republic**	1398<br>(974, 1562)	33·8<br>(22·7, 38)	1612<br>(1249, 2653)	17·5<br>(13·6, 28·4)	-48·3<br>(-62·4, 15·5)
**Grenada**	15<br>(14, 16)	21·3<br>(19·8, 22·8)	19<br>(17, 20)	13<br>(11·9, 14·2)	-39<br>(-45·1, -32·3)
**Guyana**	166<br>(158, 174)	37·6<br>(35·6, 39·4)	174<br>(151, 200)	26·4<br>(23·1, 30·1)	-29·8<br>(-39·6, -18·5)
**Haiti**	1004<br>(670, 1433)	27·8<br>(19·5, 42)	1656<br>(1214, 2439)	23·5<br>(17·3, 35·5)	-15·6<br>(-34·1, 11·3)
**Jamaica**	161<br>(148, 174)	8·8<br>(8·1, 9·5)	192<br>(156, 246)	6·6<br>(5·3, 8·4)	-25·4<br>(-40·5, 1·8)
**Puerto Rico**	1018<br>(981, 1065)	27·7<br>(26·7, 29)	953<br>(880, 1031)	14·7<br>(13·5, 15·9)	-47<br>(-51·6, -41·7)
**Saint Lucia**	22<br>(21, 23)	24·1<br>(22·6, 25·6)	28<br>(25, 30)	13·1<br>(11·9, 14·3)	-45·6<br>(-51·2, -39)
**Saint Vincent and the Grenadines**	12<br>(11, 13)	15·9<br>(14·5, 17·2)	17<br>(15, 19)	12·5<br>(11·3, 13·8)	-21·8<br>(-31·5, -9·3)
**Suriname**	71<br>(65, 76)	25·9<br>(23·8, 28)	124<br>(108, 139)	21<br>(18·5, 23·6)	-19<br>(-29·4, -7·3)
**Trinidad and Tobago**	149<br>(142, 157)	16·5<br>(15·7, 17·4)	181<br>(148, 223)	10·1<br>(8·3, 12·4)	-38·7<br>(-50·2, -25)
**Virgin Islands**	20<br>(18, 22)	22·1<br>(20·1, 24·6)	30<br>(25, 35)	17·1<br>(14·2, 19·6)	-22·7<br>(-37·5, -9·1)
**Tropical Latin America**	22455<br>(21876, 23020)	21·6<br>(20·9, 22·1)	36940<br>(35695, 38039)	15·5<br>(15, 16)	-28<br>(-30·9, -25·5)
**Brazil**	22134<br>(21506, 22717)	21·8<br>(21·1, 22·3)	36269<br>(35089, 37363)	15·6<br>(15·1, 16·1)	-28·3<br>(-31·1, -25·7)
**Paraguay**	321<br>(271, 428)	13·6<br>(11·4, 18)	671<br>(528, 994)	12·3<br>(9·7, 18)	-9·5<br>(-29·6, 15·4)

**East Asia**	161530 (132157, 174086)	16·7 (13·7, 18)	167637 (154663, 215370)	8·3 (7·6, 10·7)	-50·3 (-57, -29·4)
**China**	150718 (121900, 162617)	16·4 (13·3, 17·7)	153769 (140873, 199903)	8 (7·3, 10·4)	-51·2 (-58·2, -29·1)
**North Korea**	2879 (2314, 3588)	16·5 (13·3, 20·4)	4657 (3534, 5996)	14·9 (11·4, 19·1)	-9·7 (-32·8, 16·7)
**Taiwan (province of China)**	5242 (5074, 5415)	32·9 (31·9, 34·1)	6510 (6122, 6979)	17·6 (16·6, 18·9)	-46·4 (-50, -42·3)
**Southeast Asia**	110986 (98342, 120206)	38·8 (34·2, 42·4)	176318 (164698, 190078)	29·5 (27·7, 31·9)	-23·9 (-32·4, -14·1)
**Cambodia**	4237 (3312, 5334)	83·7 (66·9, 103·9)	9018 (7635, 10827)	79·4 (67·4, 96·1)	-5·1 (-24·6, 22·3)
**Indonesia**	58847 (51638, 63855)	55·4 (47·2, 60·5)	82145 (76441, 90687)	40·6 (37·7, 44·2)	-26·7 (-35·3, -15)
**Laos**	877 (628, 1139)	37·1 (27·6, 49·1)	1595 (1144, 2123)	35 (25·2, 46·6)	-5·5 (-35·1, 35·8)
**Malaysia**	1477 (1242, 1624)	15 (12·1, 16·6)	3082 (2530, 3789)	12·1 (9·8, 15·1)	-19 (-34·3, -2·3)
**Maldives**	10 (7, 14)	11 (7·4, 13·9)	18 (14, 21)	6·2 (4·9, 7·1)	-43·8 (-59, -8·5)
**Mauritius**	245 (233, 255)	29·2 (27·9, 30·5)	238 (217, 263)	14·3 (13·1, 15·7)	-51·1 (-55·9, -45·3)
**Myanmar**	16887 (12300, 21520)	60·8 (44·3, 77·2)	23171 (19458, 26982)	47 (40·3, 55)	-22·8 (-41·7, 9·8)
**Philippines**	3796 (3510, 4119)	10·5 (9·8, 11·3)	12696 (10889, 14747)	17 (14·7, 19·5)	61·5 (38·4, 89·7)
**Sri Lanka**	3431 (2818, 3745)	28·4 (24·5, 30·7)	4021 (3278, 4893)	16·6 (13·6, 20)	-41·5 (-52·4, -28·9)
**Seychelles**	13 (12, 16)	23·7 (21, 27·4)	27 (24, 30)	24·6 (21·4, 27·1)	3·6 (-10·6, 22·9)
**Thailand**	9367 (8150, 10560)	22·7 (19·6, 25·7)	17239 (13925, 19624)	17·6 (14·4, 19·9)	-22·5 (-35·2, -6·9)
**East Timor**	86 (60, 115)	23·1 (14·9, 32·1)	227 (136, 304)	28·1 (17·1, 37·5)	21·6 (-13·9, 73·9)
**Vietnam**	11566 (9031, 14715)	28·1 (21·7, 35·9)	22607 (18553, 27928)	24·2 (19·9, 29·9)	-13·8 (-39·1, 23·1)
**Oceania**	940 (814, 1095)	24·5 (21·4, 28·2)	1735 (1444, 2105)	20·8 (17·8, 24·5)	-15·1 (-29·8, 1·5)
**American Samoa**	4 (3, 5)	16·1 (12·8, 20)	6 (5, 7)	13·4 (11·9, 15·3)	-16·8 (-34·6, 3·6)
**Federated States of Micronesia**	16 (12, 24)	29·1 (22·3, 44·3)	14 (9, 20)	18·9 (13, 26·6)	-35·2 (-52·1, -11·4)
**Fiji**	38 (31, 45)	8·6 (6·9, 10·2)	66 (56, 77)	8·7 (7·5, 10·1)	1·7 (-19·5, 29·9)

**Guam**	21<br>(16, 25)	23·8<br>(19, 27·9)	34<br>(31, 40)	19·5<br>(17·3, 22·4)	-18·2<br>(-33·6, 5·4)
**Kiribati**	12<br>(9, 17)	27·8<br>(20·2, 39·4)	18<br>(12, 25)	24<br>(15·8, 36·4)	-13·7<br>(-30·9, 7·3)
**Marshall Islands**	6<br>(4, 8)	30·1<br>(21·8, 37)	9<br>(6, 12)	24·3<br>(16, 32·4)	-19·4<br>(-36·1, -0·2)
**Northern Mariana Islands**	5<br>(4, 6)	22·6<br>(18·7, 27·9)	9<br>(8, 10)	17·4<br>(15·4, 19·8)	-23<br>(-40·2, -4·8)
**Papua New Guinea**	667<br>(555, 807)	26·5<br>(22·5, 31·7)	1307<br>(1030, 1665)	21·8<br>(17·4, 26·9)	-17·6<br>(-34·9, 2·1)
**Samoa**	19<br>(14, 30)	20·7<br>(15·2, 33)	23<br>(17, 32)	16·5<br>(12·4, 23·1)	-20·3<br>(-36, -2)
**Solomon Islands**	41<br>(32, 50)	23·3<br>(18·4, 28·3)	75<br>(61, 89)	19·2<br>(16, 22·4)	-17·6<br>(-34·5, 2·2)
**Tonga**	15<br>(13, 18)	26·8<br>(22·8, 30·9)	20<br>(16, 24)	24·7<br>(20·3, 29·6)	-7·9<br>(-25·2, 13·8)
**Vanuatu**	33<br>(16, 55)	39·6<br>(19·1, 66·5)	59<br>(29, 107)	31·7<br>(15·2, 58·3)	-20<br>(-39·6, 7·3)
**North Africa and Middle East**	48989<br>(37286, 55723)	29·8<br>(20·1, 34·7)	77391<br>(61260, 90640)	19·3<br>(14·7, 22·6)	-35·1<br>(-48·8, -20·8)
**Afghanistan**	1453<br>(673, 2151)	20·8<br>(10·2, 32·3)	2243<br>(1441, 3104)	18·6<br>(12·1, 27)	-10·4<br>(-29·9, 54·5)
**Algeria**	1614<br>(1168, 2388)	12·8<br>(8·8, 19·6)	2935<br>(2265, 3750)	9·2<br>(7·1, 11·9)	-28·1<br>(-44·3, -6·7)
**Bahrain**	38<br>(32, 43)	20·9<br>(16·6, 24)	84<br>(70, 98)	9·4<br>(7·6, 10·7)	-55·2<br>(-62·7, -45·9)
**Egypt**	29307<br>(20398, 32344)	133·1<br>(76·4, 150·8)	44692<br>(32254, 58243)	103·3<br>(64·4, 133·4)	-22·4<br>(-42·1, -1·5)
**Iran**	2724<br>(2375, 3057)	9·8<br>(8·4, 11·3)	5400<br>(4957, 5741)	8·1<br>(7·5, 8·7)	-17·1<br>(-31·2, -2·9)
**Iraq**	966<br>(783, 1229)	11·1<br>(9·1, 14·4)	1185<br>(1071, 1330)	4·7<br>(4·2, 5·4)	-58<br>(-65·7, -47·6)
**Jordan**	195<br>(158, 245)	12·7<br>(10·4, 15·8)	445<br>(368, 522)	8·1<br>(6·5, 9·5)	-36·5<br>(-51·2, -13·2)
**Kuwait**	54<br>(50, 58)	6·7<br>(6·2, 7·3)	159<br>(132, 180)	6<br>(5·1, 6·7)	-11·5<br>(-28·8, 2·9)
**Lebanon**	298<br>(236, 360)	13·6<br>(10·9, 16·5)	519<br>(367, 664)	8·9<br>(6·3, 11·3)	-34·9<br>(-60·2, -12·1)
**Libya**	309<br>(230, 425)	15·6<br>(11·4, 21·8)	568<br>(387, 735)	12·8<br>(8·8, 16·3)	-18·2<br>(-41·5, 23·9)
**Morocco**	2047<br>(1578, 2762)	13·9<br>(10·2, 19·6)	3298<br>(2729, 3958)	11<br>(9·1, 13·3)	-20·9<br>(-43·1, 8·9)
**Palestine**	118<br>(87, 153)	12·1<br>(9, 15·6)	222<br>(198, 248)	9·2<br>(8·1, 10·4)	-24·2<br>(-40, 5)

**Oman**	89<br>(69, 111)	11·8<br>(8·7, 15·1)	176<br>(128, 221)	8·6<br>(6·5, 10·5)	-27·2<br>(-47·8, -3·7)
**Qatar**	27<br>(22, 32)	23·5<br>(18·7, 28·1)	114<br>(84, 141)	13·5<br>(10·4, 16·4)	-42·6<br>(-55·2, -25·9)
**Saudi Arabia**	1575<br>(742, 2146)	26·4<br>(10·5, 36·5)	2461<br>(1573, 3151)	18·4<br>(10·3, 23·5)	-30·1<br>(-51·8, 9·8)
**Sudan**	2248<br>(1365, 3476)	23·5<br>(12·5, 39·2)	3148<br>(2006, 4612)	17·2<br>(9·9, 26·4)	-26·9<br>(-47·5, 9·1)
**Syria**	846<br>(717, 973)	14·6<br>(12·6, 16·7)	1525<br>(1256, 1841)	12·3<br>(10·3, 14·7)	-15·7<br>(-33·8, 7·2)
**Tunisia**	548<br>(405, 723)	11·4<br>(8·3, 15·4)	1001<br>(776, 1269)	8·8<br>(6·9, 11·1)	-23<br>(-46·8, 7·5)
**Turkey**	3404<br>(2386, 4533)	9·2<br>(6·6, 12·4)	5024<br>(4374, 5705)	5·8<br>(5·1, 6·6)	-36·8<br>(-56·2, -9)
**United Arab Emirates**	76<br>(32, 111)	15·1<br>(5·8, 22·7)	413<br>(202, 561)	10·7<br>(4·8, 14·5)	-29<br>(-52·4, 9·4)
**Yemen**	1021<br>(551, 1632)	19·2<br>(9·5, 32·7)	1708<br>(1228, 2400)	13·1<br>(9, 18·9)	-31·8<br>(-56, 16·7)
**South Asia**	159399<br>(143501, 184962)	23<br>(21, 25·6)	295615<br>(268243, 378263)	21<br>(19, 27·1)	-8·7<br>(-18·9, 8·6)
**Bangladesh**	22245<br>(17520, 25700)	34·1<br>(25·5, 39·4)	26390<br>(22510, 31671)	21·3<br>(18·3, 25·7)	-37·6<br>(-50·5, -7·5)
**Bhutan**	107<br>(87, 131)	35<br>(28·2, 43·5)	157<br>(111, 211)	23·6<br>(17, 31·7)	-32·5<br>(-52, -5·6)
**India**	110091<br>(94539, 141189)	19·6<br>(16·9, 24·5)	217896<br>(192942, 299365)	19·1<br>(16·8, 26·5)	-2·6<br>(-14·8, 13·1)
**Nepal**	3139<br>(2408, 4059)	27·2<br>(20·7, 35·6)	5671<br>(4188, 7405)	25·5<br>(19, 33·1)	-6·3<br>(-31, 24·4)
**Pakistan**	23817<br>(16501, 29930)	37·3<br>(24·1, 48·4)	45501<br>(33840, 57211)	37·2<br>(26·8, 47)	-0·3<br>(-20·6, 29·1)
**Southern sub-Saharan Africa**	5789<br>(4628, 6513)	18·6<br>(14·3, 21·3)	6784<br>(5629, 7912)	11·6<br>(9·7, 13·5)	-37·6<br>(-51·4, -18·2)
**Botswana**	118<br>(77, 169)	18·2<br>(12·1, 25·7)	187<br>(141, 262)	12·9<br>(10, 17·8)	-29·2<br>(-47·3, -5·6)
**eSwatini**	104<br>(76, 140)	30<br>(22·2, 41·1)	127<br>(96, 170)	20<br>(15·2, 26·7)	-33·3<br>(-52·9, -7·2)
**Lesotho**	183<br>(79, 281)	17·2<br>(7·3, 26·4)	209<br>(96, 318)	16·3<br>(7·4, 24·4)	-5·2<br>(-36·4, 48·6)
**Namibia**	94<br>(46, 139)	12·3<br>(6, 18·2)	217<br>(131, 330)	14·3<br>(8·7, 21·7)	16·4<br>(-24·9, 72·2)
**South Africa**	4168<br>(3235, 4843)	17·5<br>(13, 20·7)	4605<br>(4125, 5095)	10<br>(9, 11·1)	-42·7<br>(-50·7, -25)
**Zimbabwe**	1123<br>(928, 1310)	25·4<br>(21, 29·6)	1440<br>(453, 2504)	20<br>(6·2, 34·5)	-21·3<br>(-76·7, 38·4)

**Western sub-Saharan Africa**	53268 (33630, 75518)	54·6 (33·2, 78·3)	72598 (48859, 101647)	35·8 (23·9, 49·9)	-34·5 (-50·8, -13·6)
**Benin**	899 (628, 1289)	39·8 (27·2, 58·3)	1269 (901, 1740)	24·1 (16·9, 32·9)	-39·6 (-68·3, -8·4)
**Burkina Faso**	1847 (1497, 2150)	40·5 (32·8, 47·2)	2090 (199, 3727)	21·9 (1·7, 39·2)	-46 (-96·1, 2·6)
**Cameroon**	2451 (1891, 2948)	49·3 (37·8, 59·5)	4080 (1862, 6075)	31·5 (14, 47·7)	-36·1 (-65·8, -8·6)
**Cape Verde**	37 (29, 46)	16·4 (12·9, 20·3)	86 (76, 100)	18·4 (16·1, 21·4)	12·3 (-14·8, 49·3)
**Chad**	1024 (663, 1416)	32·8 (20·7, 45·8)	2040 (1545, 2597)	32·1 (23·9, 41·8)	-2·1 (-26·4, 40·7)
**Côte d'Ivoire**	1972 (1467, 2654)	40·1 (29·5, 55·2)	3511 (2670, 4553)	29·1 (22·2, 38·4)	-27·5 (-51·9, -3·3)
**The Gambia**	132 (80, 195)	31·9 (19, 46·7)	284 (205, 366)	27·7 (20·1, 35·3)	-13·3 (-40, 32·5)
**Ghana**	2749 (2237, 3393)	38·7 (31·2, 47·6)	4004 (1347, 7070)	24 (7·7, 42·6)	-38 (-82·2, 6·2)
**Guinea**	1370 (869, 2277)	38 (23·2, 64·3)	1689 (1077, 2658)	28·9 (18·2, 46)	-24 (-43·2, 13)
**Guinea-Bissau**	211 (139, 288)	45·9 (30·5, 61)	290 (220, 392)	35·3 (27·2, 47·6)	-23·1 (-42·1, 9·9)
**Liberia**	651 (468, 880)	54·2 (38, 75·4)	733 (537, 980)	33·4 (24·2, 44·7)	-38·3 (-53·1, -20·7)
**Mali**	1826 (1201, 2808)	41·6 (26·2, 66·2)	1784 (695, 3024)	18·7 (6·7, 32·4)	-55·1 (-88·2, -20)
**Mauritania**	372 (276, 504)	33·9 (25·3, 46·8)	426 (301, 574)	20·1 (14·4, 27)	-40·6 (-53·9, -24·6)
**Niger**	1068 (764, 1613)	30·7 (21·8, 48)	1504 (417, 2560)	17·4 (4·4, 30·2)	-43·2 (-81·3, -13·7)
**Nigeria**	33926 (16623, 54146)	69·2 (32·9, 110·3)	45474 (19958, 76700)	47·9 (20·7, 81·4)	-30·7 (-50·3, -0·1)
**São Tomé and Príncipe**	51 (34, 64)	70·2 (46·2, 88·6)	79 (61, 110)	69·3 (52·7, 94·6)	-1·2 (-35·2, 45·5)
**Senegal**	981 (624, 1359)	26·8 (16·5, 37·6)	1421 (590, 2146)	18·1 (7·2, 27·2)	-32·4 (-59, -12·4)
**Sierra Leone**	1103 (753, 1635)	52 (35, 78·2)	1086 (434, 1577)	28·1 (10·7, 41·4)	-46 (-86·1, 2·1)
**Togo**	597 (454, 785)	40·4 (30·2, 53·9)	747 (249, 1155)	19·1 (6·1, 29·3)	-52·8 (-87·9, -16·6)
**Eastern sub-Saharan Africa**	38733 (32173, 46352)	48·6 (41·7, 56·9)	57847 (44087, 69973)	34·8 (26·4, 42·2)	-28·3 (-47·4, -4·1)
**Burundi**	1671 (1087, 2470)	71·1 (47·5, 103·5)	1719 (526, 2766)	38·9 (11·2, 62·8)	-45·3 (-85·1, -10·3)

**Comoros**	47 (7, 82)	21·4 (2·8, 37·6)	88 (18, 138)	19·2 (3·8, 30·3)	-10·1 (-30·3, 41·9)
**Djibouti**	46 (30, 67)	27·1 (18, 39·2)	143 (90, 207)	24·7 (15·3, 35·9)	-9 (-37·3, 29·9)
**Eritrea**	519 (318, 839)	48·6 (32·2, 75·6)	1112 (851, 1439)	44 (34, 55·8)	-9·3 (-36·4, 41·3)
**Ethiopia**	10886 (6858, 16649)	53·1 (35·1, 76·9)	16069 (13632, 19052)	38·6 (33·2, 45·8)	-27·3 (-51·3, 20·8)
**Kenya**	5273 (3388, 7468)	59·7 (38·7, 84·4)	10398 (6919, 14461)	47·1 (31·9, 65·5)	-21·1 (-52·1, 3·5)
**Madagascar**	2050 (1466, 3060)	36·3 (26·1, 53·6)	2939 (2123, 3717)	26·2 (18·9, 33·1)	-27·9 (-60·3, 13·2)
**Malawi**	2328 (1358, 2968)	54·6 (35·5, 68)	2640 (545, 4156)	33·1 (6·6, 51·9)	-39·4 (-88·8, 19·7)
**Mozambique**	1933 (1285, 2586)	29·6 (19·7, 39·6)	2380 (413, 3843)	19·9 (3·3, 31·9)	-32·9 (-89·6, 36·3)
**Rwanda**	2716 (2141, 3229)	88·1 (71·9, 104·8)	2668 (1696, 3707)	44·9 (28·5, 61·7)	-49 (-69·8, -25·3)
**Somalia**	961 (447, 1681)	34·6 (18·6, 57·5)	2522 (1562, 3755)	37·8 (23·8, 56·3)	9·3 (-24·2, 73·1)
**South Sudan**	836 (459, 1389)	33·6 (20·4, 53·2)	1397 (935, 2026)	34·9 (23·7, 49·8)	3·7 (-27·4, 60·2)
**Tanzania**	4542 (3346, 5819)	39·4 (30·1, 49·8)	6161 (2520, 9250)	24·4 (9·5, 37·2)	-38·3 (-76·2, -2·3)
**Uganda**	2724 (1699, 3870)	39 (24·2, 56)	4316 (1714, 6471)	29·4 (11·1, 43·9)	-24·8 (-58·8, 6·1)
**Zambia**	2182 (1683, 2609)	69·9 (55·4, 83·3)	3257 (2724, 3832)	47·3 (39·8, 55·9)	-32·3 (-46·9, -14·2)
**Central sub-Saharan Africa**	10419 (7738, 14093)	40·1 (29·1, 55·1)	20330 (15739, 26353)	34·3 (25·9, 45·2)	-14·5 (-29·6, 6·3)
**Angola**	2102 (1154, 3386)	46·2 (24·6, 77)	5294 (3920, 7402)	42·9 (31·1, 62·8)	-7·2 (-35·7, 51·9)
**Central African Republic**	538 (303, 773)	38·3 (21·4, 54)	699 (298, 1143)	27·4 (11·4, 43·7)	-28·6 (-51·5, -5·8)
**Congo**	589 (405, 903)	48·2 (32·7, 73·8)	1014 (653, 1475)	36·7 (23·4, 54·8)	-23·9 (-42·4, 3·2)
**DR Congo**	6771 (4985, 9242)	37·5 (27·1, 51·5)	12753 (9386, 17189)	31·8 (23·2, 43·6)	-15·2 (-34·7, 7·9)
**Equatorial Guinea**	78 (24, 135)	34·4 (10·4, 58·1)	149 (82, 242)	27·1 (14·5, 43·7)	-21·2 (-50·3, 67)
**Gabon**	341 (234, 477)	57 (38·8, 82·4)	421 (280, 618)	37·8 (24·7, 56·6)	-33·7 (-47·8, -16)

Appendix Table 4: DALYs and age-standardised DALY rates due to cirrhosis in 1990 and 2017 for both sexes and percentage change of age-standardised rates per 100 000 by location. DALYs=disability-adjusted life-years.

	1990		2017		Percentage change in age-standardised rates between 1990 and 2017
	Counts (95% UI)	Rate (95% UI)	Counts (95% UI)	Rate (95% UI)	
**Global**	30523343 (28633671, 32178401)	656·4 (612·8, 689·2)	41397988 (39550486, 45145938)	510·7 (487·6, 557·1)	-22·2 (-27·6, -15)
**High-income North America**	1127267 (1093235, 1157333)	351·1 (340·3, 360·4)	1842408 (1775953, 1911748)	352 (338·8, 366·1)	0·3 (-3·5, 5·1)
**Canada**	77802 (74920, 80634)	245·4 (236·4, 254·4)	119651 (110413, 129273)	208·8 (192·9, 225·9)	-14·9 (-21·5, -8·1)
**Greenland**	170 (147, 197)	326·4 (281·2, 384)	228 (173, 263)	302·3 (227·5, 347·8)	-7·4 (-33·9, 18·2)
**USA**	1049270 (1016853, 1077423)	363 (351·7, 372·7)	1722496 (1658806, 1790382)	368·9 (354·7, 384·4)	1·6 (-2·5, 6·9)
**Australasia**	43213 (41372, 44733)	190·7 (182·4, 197·8)	62493 (56287, 69582)	153·9 (138·3, 171·3)	-19·3 (-27·6, -9·8)
**Australia**	38098 (36289, 39569)	201·3 (191·6, 209·4)	55561 (49461, 62660)	163·1 (145·3, 184)	-19 (-27·9, -8·1)
**New Zealand**	5115 (4830, 5459)	136·1 (128·5, 145·2)	6932 (6415, 7541)	105·2 (97·1, 114·4)	-22·7 (-28·9, -16·3)
**High-income Asia Pacific**	1151538 (1115870, 1192803)	554·5 (537, 575)	835992 (758394, 906687)	259·6 (233·5, 284·1)	-53·2 (-57·2, -49·7)
**Brunei**	546 (422, 629)	354·8 (294·5, 405·5)	975 (851, 1124)	229·3 (201·8, 273·2)	-35·4 (-46·6, -15·7)
**Japan**	613874 (590334, 640533)	360·6 (345·5, 377·7)	539871 (491918, 583922)	224·2 (203·4, 244·9)	-37·8 (-42·1, -34·3)
**Singapore**	6289 (5912, 6684)	229·8 (216·3, 243·9)	8564 (7574, 9738)	121·4 (107·3, 138·5)	-47·2 (-52·5, -41·9)
**South Korea**	530829 (512951, 552031)	1358 (1313, 1410·6)	286583 (248738, 323323)	346 (300·3, 389·6)	-74·5 (-77·9, -71·2)
**Western Europe**	2183224 (2133625, 2236055)	432·1 (422·1, 443)	1840821 (1740698, 1948972)	266·2 (250·6, 283·4)	-38·4 (-41·8, -35)
**Andorra**	195 (147, 243)	319·1 (241·6, 398·1)	303 (229, 383)	240·7 (181·9, 303·2)	-24·6 (-43·4, -1·5)
**Austria**	65949 (63793, 68404)	661·4 (639·5, 686·9)	48925 (44939, 52841)	346 (317, 374·5)	-47·7 (-52, -43)
**Belgium**	44365 (42725, 46007)	334·7 (322·6, 346·8)	49938 (45540, 54372)	281·5 (256·2, 307)	-15·9 (-23·1, -8·8)
**Cyprus**	2136 (1685, 2665)	258·2 (204·1, 323)	2954 (2598, 3413)	168·2 (147·8, 195·6)	-34·8 (-48·5, -14·7)
**Denmark**	23648 (22555, 24763)	363·8 (346·7, 381·2)	25917 (23795, 28223)	290·8 (266·1, 317·5)	-20·1 (-27·6, -11·2)
**Finland**	18611 (17592, 19678)	291·7 (275·6, 309·3)	34697 (31735, 37782)	416 (379·4, 455·4)	42·6 (26·7, 58·6)
**France**	360515 (349196, 373585)	504·2 (488·1, 521·8)	270000 (241744, 296972)	269·3 (239·4, 298·3)	-46·6 (-52·4, -41·2)
**Germany**	581924 (561819, 601094)	532·5 (515·2, 550)	495800 (442312, 555573)	353·7 (316·4, 396·3)	-33·6 (-40·6, -25·4)
**Greece**	35527 (33803, 37175)	246·2 (234·1, 257·5)	30483 (28067, 33211)	173·4 (159·1, 189·9)	-29·6 (-35·4, -23·3)
**Iceland**	362 (335, 388)	137·8 (127·7, 148·4)	593 (531, 655)	129·8 (115·8, 144·1)	-5·9 (-15·2, 4·4)
**Ireland**	5243 (4949, 5557)	138·1 (130, 146·9)	11662 (10323, 12973)	180·7 (159·3, 201·2)	30·8 (15·1, 45·3)
**Israel**	9693 (9130, 10335)	201·5 (189·5, 214·6)	16372 (14799, 18052)	160·3 (144·1, 178)	-20·5 (-26·7, -13·1)
**Italy**	433796 (420301, 448387)	534·6 (517, 552·9)	246753 (223204, 270531)	221·2 (198·2, 243·3)	-58·6 (-62·5, -54·8)

**Luxembourg**	2643<br>(2497, 2792)	521·9<br>(492·1, 551)	2730<br>(2279, 3160)	317·4<br>(264·4, 368·5)	-39·2<br>(-49·2, -30·1)
**Malta**	971<br>(898, 1036)	224·8<br>(208·2, 240)	1131<br>(1037, 1233)	163·4<br>(149·8, 178·2)	-27·3<br>(-34·6, -18·8)
**Netherlands**	33561<br>(32053, 35126)	181·8<br>(173·5, 190·4)	38452<br>(35081, 41938)	137·7<br>(125·1, 150·1)	-24·3<br>(-30·2, -18)
**Norway**	9085<br>(8704, 9483)	170·6<br>(163·4, 178·3)	9701<br>(8733, 10578)	125·5<br>(112·4, 138)	-26·4<br>(-33·5, -21·3)
**Portugal**	99616<br>(96590, 102494)	792·1<br>(768·6, 814·6)	54457<br>(48991, 60061)	310<br>(277·8, 344·8)	-60·9<br>(-65·1, -56·6)
**Spain**	274774<br>(266235, 284122)	551·3<br>(534, 570·4)	187474<br>(172959, 203480)	243<br>(223·3, 264·7)	-55·9<br>(-59·5, -51·8)
**Sweden**	21437<br>(20522, 22406)	182<br>(174, 190·7)	25488<br>(23507, 27440)	160·1<br>(146·9, 173·4)	-12<br>(-18·4, -5·6)
**Switzerland**	22210<br>(21057, 23352)	242·8<br>(230, 255·3)	22917<br>(20297, 25456)	164·7<br>(144·6, 184)	-32·2<br>(-40·5, -24·2)
**UK**	134858<br>(129733, 139678)	183·6<br>(176·7, 190·6)	262166<br>(252972, 272312)	278·2<br>(268·2, 289·5)	51·5<br>(47·5, 58·7)
**Southern Latin America**	287279<br>(279358, 297167)	601·7<br>(585, 622·5)	342023<br>(312479, 374971)	442·5<br>(404·7, 485·4)	-26·5<br>(-32·8, -19·4)
**Argentina**	157403<br>(151726, 163011)	478·5<br>(461·1, 495·6)	196571<br>(172705, 223692)	393·8<br>(346, 449·6)	-17·7<br>(-28·1, -6·5)
**Chile**	116591<br>(111721, 123850)	1040·1<br>(996·6, 1104·1)	133587<br>(117416, 150410)	584·5<br>(513·7, 659·3)	-43·8<br>(-50·4, -35·8)
**Uruguay**	13273<br>(12605, 13860)	362·6<br>(344·8, 379·1)	11850<br>(10544, 13535)	258·4<br>(229·4, 295·1)	-28·7<br>(-37·3, -19·3)
**Eastern Europe**	961173<br>(904792, 1001426)	351·9<br>(330, 367·7)	2729903<br>(2617994, 2833616)	967·1<br>(925·1, 1006·3)	174·8<br>(165·8, 185·5)
**Belarus**	26656<br>(24191, 28588)	212·4<br>(192, 228·3)	83810<br>(73119, 93620)	622·9<br>(541·8, 698·3)	193·3<br>(148·7, 234)
**Estonia**	4782<br>(4473, 5154)	250·3<br>(233·3, 270)	9569<br>(8244, 11075)	522·7<br>(444·9, 606·7)	108·9<br>(74·4, 144·6)
**Latvia**	7835<br>(7345, 8359)	230·7<br>(215·7, 246·5)	14008<br>(12291, 15765)	508·7<br>(444·7, 576·9)	120·5<br>(91·4, 153·5)
**Lithuania**	12059<br>(11373, 12773)	278·1<br>(262·4, 295·3)	31650<br>(29223, 34187)	805·4<br>(742, 873·1)	189·6<br>(166·7, 217·2)
**Moldova**	96120<br>(92922, 99751)	2014·2<br>(1946·4, 2089)	91041<br>(85316, 96339)	1694·4<br>(1583·7, 1792·7)	-15·9<br>(-22·8, -10)
**Russia**	549721<br>(497352, 583440)	307·6<br>(276·9, 327·3)	1753143<br>(1705696, 1802317)	893·5<br>(868, 919·9)	190·4<br>(176·5, 217·9)
**Ukraine**	263999<br>(252539, 275223)	395·6<br>(378·5, 412·9)	746681<br>(678703, 808440)	1262·5<br>(1141·1, 1370·7)	219·1<br>(185·1, 248·6)
**Central Europe**	943631<br>(919953, 973769)	642·1<br>(625·6, 662·9)	910197<br>(871665, 951307)	520·1<br>(496·6, 544·2)	-19<br>(-22·6, -15·4)
**Albania**	7472<br>(6873, 8173)	298·9<br>(273·9, 324·2)	8657<br>(6971, 11720)	232·3<br>(186·4, 315·7)	-22·3<br>(-37·7, 4·3)
**Bosnia and Herzegovina**	24078<br>(22433, 27886)	505·7<br>(472·8, 576·6)	16608<br>(14420, 22599)	307<br>(266·9, 416·3)	-39·3<br>(-46·6, -25·4)
**Bulgaria**	56347<br>(53953, 58632)	472·2<br>(451·6, 491·5)	60350<br>(55352, 65443)	545·7<br>(499, 592·8)	15·6<br>(5·4, 26·3)
**Croatia**	54241<br>(52267, 56362)	840·2<br>(809, 872·8)	29237<br>(27066, 31576)	410·2<br>(379·5, 445·3)	-51·2<br>(-54·8, -46·9)
**Czech Republic**	67616<br>(64798, 71383)	529·5<br>(507·2, 559)	64483<br>(59454, 69941)	393<br>(361·1, 428)	-25·8<br>(-33·1, -18·1)
**Hungary**	200575<br>(194990, 206401)	1510·7<br>(1468·7, 1555·6)	93669<br>(86836, 100554)	603·2<br>(558·9, 649·3)	-60·1<br>(-62·9, -56·9)
**Montenegro**	1346<br>(1182, 1525)	202·4<br>(178, 228·9)	1691<br>(1444, 2011)	191·4<br>(163·1, 226·6)	-5·4<br>(-20·3, 12·3)
**North Macedonia**	5142<br>(4698, 5986)	249·7<br>(228·3, 288·7)	7438<br>(6385, 8405)	239·9<br>(205·4, 270·8)	-3·9<br>(-20·1, 10·5)
**Poland**	146019<br>(138029, 155056)	330·7<br>(312·4, 350·8)	251185<br>(231362, 272517)	450·2<br>(413·8, 488·7)	36·2<br>(22·9, 49·2)

**Romania**	253977 (244178, 263455)	907·3 (871·4, 940·9)	276609 (258723, 296176)	907·3 (847·8, 974)	0 (-7·3, 7·9)
**Serbia**	47616 (39029, 54996)	397·9 (328·1, 455·4)	36680 (33004, 42014)	274·1 (245·8, 316·4)	-31·1 (-42·3, -16·7)
**Slovakia**	54426 (48805, 64424)	937·5 (841·7, 1103·8)	48598 (38674, 54419)	603·7 (479·9, 675·9)	-35·6 (-52·5, -23·1)
**Slovenia**	24776 (23673, 25860)	1003·1 (958·2, 1047·3)	14992 (13575, 16305)	436·9 (394·9, 476·4)	-56·4 (-60·5, -52·5)
**Central Asia**	431614 (421034, 448982)	776·3 (757·6, 807·3)	1048825 (967341, 1120738)	1197·1 (1107·7, 1275·3)	54·2 (40·8, 65·1)
**Armenia**	12517 (11850, 13283)	409·8 (388·6, 434·4)	24506 (23001, 26132)	623 (586·9, 661·7)	52 (41·4, 62·5)
**Azerbaijan**	42119 (39012, 46710)	720·2 (669·7, 790·4)	89166 (75779, 104015)	881·6 (751, 1027·7)	22·4 (3·6, 45·9)
**Georgia**	43456 (41186, 45828)	691 (654·2, 729·6)	40367 (36807, 44677)	810·7 (737·8, 892·5)	17·3 (5·7, 30·4)
**Kazakhstan**	66676 (63410, 69707)	459·6 (438·2, 479·8)	225144 (184597, 255052)	1196·7 (984·6, 1351·3)	160·4 (112·3, 196·7)
**Kyrgyzstan**	34698 (32908, 36581)	1018·1 (964·5, 1075·1)	76824 (67649, 83813)	1386·6 (1232·1, 1506)	36·2 (18·5, 50·3)
**Mongolia**	24463 (21757, 27613)	1764 (1574·2, 2051·7)	44389 (37999, 52174)	1583·6 (1379·9, 1782·4)	-10·2 (-21·8, 3·6)
**Tajikistan**	31991 (29027, 35899)	798·9 (732·5, 958·3)	60412 (53661, 67190)	816·8 (729·3, 911·1)	2·2 (-10·7, 14·3)
**Turkmenistan**	30117 (28824, 31335)	1183·7 (1132·6, 1226·4)	90746 (82578, 98999)	1842·5 (1681·7, 2006·2)	55·7 (41·1, 71·1)
**Uzbekistan**	145578 (140914, 150260)	1010·8 (979·2, 1043·8)	397270 (352250, 444626)	1362·3 (1209·8, 1520·6)	34·8 (19·3, 51·3)
**Central Latin America**	1106335 (1085204, 1126838)	1012 (993·1, 1030·6)	1774331 (1698187, 1860687)	717·9 (687·5, 753·1)	-29·1 (-32·3, -25·4)
**Colombia**	63247 (60114, 66170)	281·5 (268·3, 294)	106544 (93989, 120334)	199·5 (175·9, 225·4)	-29·1 (-36·7, -20·5)
**Costa Rica**	9710 (9276, 10143)	468·9 (448·5, 488·6)	23176 (20975, 25857)	459·1 (416, 512·1)	-2·1 (-11·2, 10·4)
**El Salvador**	33015 (26774, 35681)	878·8 (741·6, 940·2)	43720 (35311, 57837)	761·5 (615·6, 1005·5)	-13·4 (-31·5, 24·6)
**Guatemala**	83848 (80264, 87457)	1511·1 (1450·2, 1571·5)	160358 (142832, 179430)	1227·9 (1096·7, 1372·5)	-18·7 (-27·9, -8·2)
**Honduras**	39054 (33145, 49298)	1223·6 (1021·9, 1650·2)	76380 (58534, 101899)	1083 (827·1, 1464·1)	-11·5 (-30·4, 12·3)
**Mexico**	787959 (773767, 803028)	1428·3 (1402·8, 1454·4)	1198862 (1152734, 1252679)	970·9 (934, 1014·6)	-32 (-34·9, -28·6)
**Nicaragua**	15460 (13969, 16983)	702·9 (636·8, 768)	36176 (26226, 42330)	695·5 (507·2, 810·5)	-1·1 (-25·5, 15·8)
**Panama**	6032 (5664, 6425)	342·2 (320·7, 364·3)	12468 (11438, 13509)	313·3 (287·7, 339·2)	-8·4 (-16·7, 0·2)
**Venezuela**	68011 (65158, 70833)	551·5 (528, 574)	116647 (99145, 136910)	382·1 (324·9, 447·6)	-30·7 (-41·3, -18·2)
**Andean Latin America**	211414 (192305, 236063)	764·5 (701·1, 848·1)	298097 (267305, 328824)	532·2 (478, 587·1)	-30·4 (-39·2, -20·6)
**Bolivia**	37795 (26208, 53872)	856·6 (620·3, 1176·5)	59812 (46141, 75490)	638·5 (496·3, 807·8)	-25·5 (-45·2, 4·3)
**Ecuador**	48437 (46341, 50735)	692·3 (664·9, 720·1)	89123 (79337, 99547)	582·2 (520·1, 649·8)	-15·9 (-24·8, -5·6)
**Peru**	125182 (110368, 138273)	771·3 (693·7, 850·9)	149162 (123441, 175426)	477·3 (396·1, 561·3)	-38·1 (-48·9, -25·5)
**Caribbean**	170327 (155334, 187834)	582·2 (532·8, 636·6)	206692 (181660, 249171)	409 (359·1, 494·4)	-29·8 (-36·8, -13·7)
**Antigua and Barbuda**	232 (216, 250)	459·5 (425·9, 497·1)	312 (283, 344)	298 (271·5, 327·9)	-35·1 (-42·2, -26·9)
**The Bahamas**	1265 (1170, 1363)	661·5 (611·1, 711·4)	1680 (1501, 1877)	400·5 (360·3, 445·8)	-39·5 (-47·2, -30·6)

**Barbados**	914<br>(855, 974)	357·1<br>(333·3, 381·2)	1042<br>(927, 1168)	241·3<br>(215, 270)	-32·4<br>(-40·7, -23·4)
**Belize**	672<br>(615, 733)	585·1<br>(529·7, 638·9)	1839<br>(1693, 1997)	586·5<br>(538·1, 636·9)	0·2<br>(-9·7, 12)
**Bermuda**	244<br>(223, 267)	371·7<br>(340·8, 407·4)	179<br>(160, 208)	162·9<br>(144·1, 189·8)	-56·2<br>(-62·9, -46·9)
**Cuba**	29340<br>(28010, 31322)	276·5<br>(263·7, 295·5)	52350<br>(45759, 60164)	304·1<br>(266·9, 347·8)	10<br>(-3·5, 23·9)
**Dominica**	255<br>(238, 274)	389·3<br>(361·8, 419)	269<br>(244, 296)	321·7<br>(291·4, 352·9)	-17·4<br>(-26·3, -7·7)
**Dominican Republic**	46903<br>(35095, 52840)	924·6<br>(662·4, 1035·5)	43525<br>(33354, 75295)	450·1<br>(345·9, 768·3)	-51·3<br>(-64·6, 7·9)
**Grenada**	407<br>(378, 436)	614·6<br>(568, 662·5)	500<br>(458, 547)	382<br>(349·7, 417·3)	-37·9<br>(-44·1, -30·8)
**Guyana**	5794<br>(5505, 6095)	1140·9<br>(1084, 1199·2)	5573<br>(4829, 6444)	785·3<br>(683·5, 905·3)	-31·2<br>(-41, -19·5)
**Haiti**	35138<br>(22039, 48622)	810·6<br>(539·4, 1144·2)	52369<br>(37763, 75117)	634·8<br>(462·4, 924·4)	-21·7<br>(-41·5, 7·4)
**Jamaica**	4950<br>(4572, 5348)	260·7<br>(239·9, 281·7)	5572<br>(4570, 7138)	192·5<br>(157·8, 246)	-26·2<br>(-40·8, -1)
**Puerto Rico**	29722<br>(28568, 31042)	817·4<br>(786, 854)	22953<br>(21108, 24902)	404·9<br>(371·6, 441·9)	-50·5<br>(-54·8, -45)
**Saint Lucia**	640<br>(601, 681)	675·4<br>(632·9, 719·8)	787<br>(714, 864)	371·5<br>(336·9, 407·6)	-45<br>(-50·8, -38·1)
**Saint Vincent and the Grenadines**	349<br>(317, 377)	465<br>(421·7, 503·6)	508<br>(458, 564)	380·2<br>(344·2, 421·5)	-18·2<br>(-28·2, -6)
**Suriname**	2230<br>(2054, 2418)	737·1<br>(679·4, 798·8)	3525<br>(3079, 3991)	573·9<br>(502·7, 648·7)	-22·1<br>(-32·6, -10·5)
**Trinidad and Tobago**	4859<br>(4606, 5118)	499·4<br>(473·2, 526·1)	5498<br>(4489, 6728)	312·7<br>(256·2, 380·7)	-37·4<br>(-49·3, -23·8)
**Virgin Islands**	586<br>(528, 658)	585·4<br>(530, 655·5)	778<br>(643, 908)	463·8<br>(387·8, 534·4)	-20·8<br>(-36·1, -5·5)
**Tropical Latin America**	812621<br>(791721, 833876)	682·1<br>(665·4, 699·4)	1131886<br>(1093665, 1170479)	464·5<br>(448·9, 480·2)	-31·9<br>(-34·2, -29·1)
**Brazil**	802164<br>(781208, 823988)	689·3<br>(672·4, 707·5)	1111695<br>(1073714, 1150858)	467·7<br>(451·5, 483·9)	-32·2<br>(-34·4, -29·3)
**Paraguay**	10457<br>(8821, 13834)	379·8<br>(318·4, 511·7)	20191<br>(15831, 30288)	344·6<br>(272·3, 514·9)	-9·3<br>(-29, 14·8)
**East Asia**	5604590<br>(4556901, 6045875)	513·5<br>(418·2, 554)	5000632<br>(4603996, 6164299)	242·1<br>(222·4, 297·5)	-52·8<br>(-58·7, -34·6)
**China**	5256956<br>(4223922, 5684869)	507·2<br>(409·2, 548·3)	4599051<br>(4210440, 5703844)	233·9<br>(214, 288·7)	-53·9<br>(-59·9, -34·9)
**North Korea**	95726<br>(76545, 120397)	490·7<br>(396·1, 616·5)	141076<br>(104851, 189941)	438·3<br>(326·7, 586·7)	-10·7<br>(-33·8, 19·9)
**Taiwan (province of China)**	158535<br>(153660, 163786)	869·3<br>(843·8, 898·1)	179948<br>(168160, 193242)	513·4<br>(479·6, 550·9)	-40·9<br>(-45·1, -36)
**Southeast Asia**	3916840<br>(3420502, 4299406)	1141·1<br>(1006·7, 1235·2)	5430302<br>(5059995, 5816943)	818·9<br>(762·9, 878·6)	-28·2<br>(-35·3, -17·2)
**Cambodia**	154110<br>(118801, 198447)	2362·1<br>(1848·9, 2978·7)	280713<br>(236791, 331674)	2097·4<br>(1775·9, 2488·4)	-11·2<br>(-28·6, 20)
**Indonesia**	2072497<br>(1828335, 2293423)	1539·9<br>(1364·5, 1670·1)	2430249<br>(2263456, 2680719)	1007·6<br>(937·9, 1102)	-34·6<br>(-41·3, -25·3)
**Laos**	32099<br>(22038, 41967)	1156·6<br>(816·5, 1499·8)	53682<br>(38484, 70424)	991·8<br>(716·3, 1314·4)	-14·3<br>(-43·8, 26·9)
**Malaysia**	46342<br>(41151, 51103)	403·1<br>(345, 442·7)	87860<br>(74193, 105063)	309·2<br>(259·2, 371·3)	-23·3<br>(-38·1, -7·6)
**Maldives**	341<br>(212, 511)	282·4<br>(182·1, 394·9)	535<br>(445, 615)	151·5<br>(124·5, 173·3)	-46·3<br>(-64·2, -9·6)
**Mauritius**	8545<br>(8134, 8963)	928·3<br>(884·2, 971·2)	7349<br>(6700, 8132)	439·4<br>(401·2, 484·5)	-52·7<br>(-57·5, -47·1)
**Myanmar**	661592<br>(477455, 867490)	2117<br>(1530·1, 2712·8)	818991<br>(652734, 985074)	1549<br>(1263·2, 1850·5)	-26·8<br>(-46·5, 7·2)

**Philippines**	141782<br>(130866, 153848)	331·6<br>(305·6, 358·9)	406664<br>(348141, 476246)	470·3<br>(403·3, 545·9)	41·9<br>(20·5, 68·5)
**Sri Lanka**	117926<br>(91253, 130041)	847·8<br>(671·7, 930·9)	117453<br>(91886, 146062)	467·9<br>(369, 579·4)	-44·8<br>(-56, -31·2)
**Seychelles**	419<br>(370, 490)	727·7<br>(637·8, 853·5)	845<br>(740, 936)	708·4<br>(620·8, 784·1)	-2·7<br>(-17·1, 17·3)
**Thailand**	331995<br>(285930, 375116)	694·8<br>(605, 781·9)	546302<br>(442369, 622915)	563·7<br>(464·9, 639)	-18·9<br>(-32·4, -1)
**East Timor**	3400<br>(2525, 4492)	695·7<br>(479·6, 940·7)	6893<br>(3936, 9246)	761·1<br>(436·3, 1023)	9·4<br>(-31, 61·6)
**Vietnam**	340594<br>(273223, 437681)	740·6<br>(581·3, 959·1)	665620<br>(542231, 828968)	653·2<br>(535, 803·5)	-11·8<br>(-38·8, 23)
**Oceania**	39622<br>(34287, 46284)	842·4<br>(729, 981·4)	71820<br>(59251, 87330)	704·1<br>(588·5, 853)	-16·4<br>(-32·2, 0·9)
**American Samoa**	159<br>(131, 189)	473·4<br>(384·5, 573·9)	205<br>(181, 232)	398·5<br>(352·4, 449·4)	-15·8<br>(-31·6, 2·7)
**Federated States of Micronesia**	611<br>(453, 879)	898·4<br>(675·3, 1306·8)	512<br>(317, 720)	565·4<br>(358·4, 793·9)	-37·1<br>(-56·5, -13·5)
**Fiji**	1497<br>(1259, 1779)	264·4<br>(219·4, 312·9)	2351<br>(2031, 2743)	269·7<br>(232·2, 313·6)	2<br>(-19·1, 26·7)
**Guam**	750<br>(591, 888)	683·8<br>(544·8, 805·5)	1106<br>(978, 1257)	608·9<br>(539·1, 695·1)	-11<br>(-28·1, 14·3)
**Kiribati**	491<br>(342, 634)	895·9<br>(632·9, 1197)	696<br>(427, 953)	736·5<br>(467, 1016·5)	-17·8<br>(-36·2, 3·7)
**Marshall Islands**	249<br>(178, 307)	931·2<br>(669·2, 1164·4)	340<br>(214, 483)	733·4<br>(469·6, 1020)	-21·2<br>(-38·9, -0·4)
**Northern Mariana Islands**	208<br>(166, 259)	615·2<br>(504·2, 759)	268<br>(232, 309)	476<br>(414, 542·8)	-22·6<br>(-39·3, -2·3)
**Papua New Guinea**	28846<br>(23866, 34979)	964·7<br>(803·4, 1167·1)	55838<br>(44018, 70731)	764·5<br>(605·2, 968·4)	-20·8<br>(-38·9, 0·1)
**Samoa**	712<br>(536, 1061)	627·3<br>(470·2, 951·8)	750<br>(555, 1054)	466·4<br>(347, 653·5)	-25·6<br>(-42·2, -5·4)
**Solomon Islands**	1628<br>(1293, 2011)	774·1<br>(610·4, 953·9)	2945<br>(2380, 3522)	623·4<br>(508·8, 738·8)	-19·5<br>(-36·9, 2·2)
**Tonga**	504<br>(433, 583)	725·2<br>(628·4, 834·6)	632<br>(519, 752)	704·6<br>(577·3, 841·8)	-2·8<br>(-21·1, 19·9)
**Vanuatu**	1354<br>(659, 2195)	1331·7<br>(643·7, 2191·5)	2220<br>(1101, 3900)	1011<br>(496·6, 1799·1)	-24·1<br>(-44·7, 5·6)
**North Africa and Middle East**	1483249<br>(1308019, 1634394)	690<br>(558·9, 772·1)	2214073<br>(1915813, 2570616)	465·2<br>(391·9, 541·1)	-32·6<br>(-45·1, -19·5)
**Afghanistan**	47665<br>(19616, 73288)	572·1<br>(254·5, 841·4)	91736<br>(58758, 121732)	511<br>(336·6, 695·4)	-10·7<br>(-33·3, 73·4)
**Algeria**	55079<br>(43696, 74731)	329·3<br>(250·6, 465·2)	87737<br>(70889, 108034)	242·9<br>(195·7, 301·5)	-26·2<br>(-40·7, -10·9)
**Bahrain**	1270<br>(1097, 1436)	491·2<br>(416·7, 551·8)	3015<br>(2556, 3490)	235·6<br>(200·6, 268·1)	-52<br>(-59·6, -43·1)
**Egypt**	803465<br>(724692, 859333)	2563·1<br>(1867·8, 2805·9)	1175431<br>(909039, 1523644)	1918·4<br>(1417·4, 2480·2)	-25·2<br>(-43·2, -4·5)
**Iran**	108975<br>(93544, 122618)	270<br>(237·4, 301·6)	159319<br>(145337, 170236)	209·9<br>(192·1, 223·8)	-22·3<br>(-32·5, -10·3)
**Iraq**	37003<br>(29877, 46660)	328·9<br>(268·1, 411·2)	50003<br>(44381, 55325)	153<br>(138·2, 169·7)	-53·5<br>(-63·4, -41·2)
**Jordan**	7446<br>(5983, 9335)	349<br>(284·9, 437·9)	15657<br>(13634, 17924)	210·1<br>(181·6, 241·9)	-39·8<br>(-54, -19·4)
**Kuwait**	2267<br>(2081, 2465)	200·4<br>(184·1, 216·3)	5822<br>(4901, 6669)	170·2<br>(144·6, 192·4)	-15·1<br>(-29·7, -3·3)
**Lebanon**	9466<br>(7557, 11318)	342·3<br>(270·2, 410·4)	15777<br>(11902, 20209)	230·9<br>(172·8, 293·9)	-32·5<br>(-54·3, -9·1)
**Libya**	10255<br>(7842, 13359)	398·2<br>(300·6, 536·4)	18500<br>(13120, 23677)	330·9<br>(236, 417·6)	-16·9<br>(-38·7, 19·4)
**Morocco**	66448<br>(53949, 81849)	364·8<br>(296·4, 466·7)	95428<br>(78661, 113359)	284·5<br>(236·8, 336·6)	-22<br>(-40·6, -1·4)

**Palestine**	4109<br>(2995, 5550)	318·9<br>(240·1, 415)	7541<br>(6776, 8382)	241·4<br>(217·2, 266·9)	-24·3<br>(-41·9, 4)
**Oman**	3372<br>(2783, 4060)	326·9<br>(255·3, 402·8)	6778<br>(5065, 8399)	235·9<br>(180·2, 288·8)	-27·8<br>(-44·8, -6·3)
**Qatar**	1056<br>(887, 1257)	537·3<br>(436·3, 635)	4474<br>(3465, 5583)	307·3<br>(234·7, 373)	-42·8<br>(-55·4, -25·9)
**Saudi Arabia**	42850<br>(29043, 55375)	553<br>(283·5, 751·8)	77709<br>(57422, 97205)	378·7<br>(251·1, 474·6)	-31·5<br>(-52·7, -0·5)
**Sudan**	77347<br>(44674, 117251)	585·3<br>(366·5, 875·6)	105267<br>(77901, 141827)	424·2<br>(293, 595·2)	-27·5<br>(-46·1, 8·8)
**Syria**	30963<br>(25471, 38132)	392·1<br>(332·4, 454)	47918<br>(38995, 57047)	322·2<br>(265·1, 380·9)	-17·8<br>(-33·9, 5·4)
**Tunisia**	17302<br>(13851, 21522)	277·3<br>(214·8, 351·9)	25374<br>(19907, 31775)	209·1<br>(164·4, 261·7)	-24·6<br>(-44·4, -1·7)
**Turkey**	115132<br>(78683, 153228)	257<br>(179·6, 337)	140846<br>(124523, 157465)	162·5<br>(143·6, 182·6)	-36·7<br>(-52·8, -6·4)
**United Arab Emirates**	2933<br>(1542, 4131)	394·6<br>(171·1, 569·8)	16833<br>(9623, 22061)	279·7<br>(144·6, 369·5)	-29·1<br>(-51·4, 7·3)
**Yemen**	37891<br>(20247, 55899)	493·2<br>(271·4, 782)	60842<br>(45798, 81037)	336·8<br>(245·5, 458·5)	-31·7<br>(-54·8, 18·2)
**South Asia**	6329326<br>(5523004, 7738897)	734·7<br>(656·9, 864·2)	10118561<br>(9209547, 12797543)	633·1<br>(575·7, 803·2)	-13·8<br>(-22·8, -0·8)
**Bangladesh**	1013235<br>(806819, 1178722)	1225·1<br>(940·4, 1415·4)	857197<br>(725707, 1009115)	608·6<br>(517·4, 720·3)	-50·3<br>(-61, -30·7)
**Bhutan**	4186<br>(3352, 5221)	1099·5<br>(897·7, 1343·6)	5268<br>(3598, 7035)	667·4<br>(469·9, 897)	-39·3<br>(-58·9, -11·4)
**India**	4331241<br>(3649309, 5751620)	630·5<br>(539·4, 817·3)	7401548<br>(6597109, 10026479)	582·2<br>(518·4, 790·5)	-7·7<br>(-18·6, 8·9)
**Nepal**	123508<br>(92659, 156435)	883·6<br>(678·4, 1137·6)	178703<br>(126999, 236615)	718·1<br>(513·4, 950)	-18·7<br>(-41·5, 11·1)
**Pakistan**	857156<br>(675494, 1039120)	1122·8<br>(808·2, 1390·1)	1675846<br>(1302686, 2084646)	1050·9<br>(793, 1317·3)	-6·4<br>(-24·9, 16·1)
**Southern sub-Saharan Africa**	205606<br>(173950, 226799)	574·1<br>(472·3, 640)	222780<br>(185409, 261150)	341·3<br>(284·8, 399·9)	-40·5<br>(-53·3, -25·7)
**Botswana**	4104<br>(2642, 5910)	539·2<br>(356, 765·9)	6179<br>(4520, 8659)	355·7<br>(266·3, 494·4)	-34<br>(-50·7, -11·8)
**eSwatini**	3736<br>(2742, 4968)	895·9<br>(659·7, 1200·5)	4569<br>(3436, 6140)	610·7<br>(461·6, 818)	-31·8<br>(-52·3, -3·3)
**Lesotho**	6177<br>(2854, 9411)	520·3<br>(234·6, 795·8)	7173<br>(3445, 11037)	495·8<br>(238·2, 760·8)	-4·7<br>(-37·2, 52·7)
**Namibia**	3119<br>(1631, 4580)	353·7<br>(181·5, 529·1)	7213<br>(4451, 11179)	423·6<br>(262, 644·4)	19·8<br>(-23·7, 79·2)
**South Africa**	151725<br>(128566, 170833)	561·5<br>(455·7, 641·8)	148069<br>(132916, 163511)	294·6<br>(263·6, 325·1)	-47·5<br>(-53·7, -36·9)
**Zimbabwe**	36746<br>(30426, 42968)	667·4<br>(555·6, 779·2)	49576<br>(17130, 85660)	545·1<br>(182·4, 938·9)	-18·3<br>(-74·3, 46·4)
**Western sub-Saharan Africa**	1828673<br>(1229336, 2520617)	1531·6<br>(977, 2146·5)	2620827<br>(1809700, 3638507)	1001·9<br>(684·4, 1404·1)	-34·6<br>(-51, -12·8)
**Benin**	31210<br>(22684, 43219)	1101<br>(780·7, 1556·6)	46871<br>(34344, 63484)	688·5<br>(492, 947·8)	-37·5<br>(-65, -9·6)
**Burkina Faso**	62283<br>(50633, 73862)	1072·2<br>(870·8, 1247·6)	76968<br>(13226, 132788)	605·6<br>(80·9, 1066·6)	-43·5<br>(-92·9, 3·2)
**Cameroon**	86065<br>(66967, 103205)	1393·2<br>(1076·1, 1662·4)	147091<br>(71397, 214680)	904·1<br>(428·8, 1332·6)	-35·1<br>(-63·6, -8·7)
**Cape Verde**	1294<br>(1016, 1591)	547·5<br>(435·8, 669·6)	2908<br>(2561, 3328)	590·5<br>(520·7, 675·7)	7·8<br>(-15·3, 38·3)
**Chad**	35190<br>(24394, 47292)	945·8<br>(628·7, 1301)	77279<br>(60136, 97024)	953<br>(724·3, 1209·1)	0·8<br>(-23·9, 41·9)
**Côte d'Ivoire**	76867<br>(57632, 102018)	1142·6<br>(854·9, 1531·9)	133723<br>(103991, 170121)	852·3<br>(654·9, 1099·1)	-25·4<br>(-49·9, -0·8)
**The Gambia**	4986<br>(3136, 7248)	913<br>(562·2, 1350·3)	9944<br>(7262, 12818)	789·3<br>(576·5, 1003·6)	-13·6<br>(-39, 31·2)

**Ghana**	98651 (81006, 122478)	1087·4 (884·9, 1332·7)	139350 (52567, 238188)	672·3 (244·4, 1172·1)	-38·2 (-79·1, 2·2)
**Guinea**	46206 (31077, 73306)	1061·1 (690·2, 1731·5)	58132 (38626, 89394)	808·7 (528, 1245·5)	-23·8 (-42·7, 7·1)
**Guinea-Bissau**	7826 (5148, 10999)	1337·4 (890·2, 1827·9)	11138 (8435, 15025)	1033·5 (787·4, 1389·8)	-22·7 (-42·5, 9·7)
**Liberia**	21441 (15847, 28033)	1522 (1114·4, 2024·7)	26791 (19587, 36135)	926·1 (687·9, 1232·6)	-39·1 (-53·4, -22·5)
**Mali**	67686 (44299, 100744)	1181·3 (782·2, 1799·3)	71580 (33232, 112753)	562 (237·5, 940·5)	-52·4 (-84·7, -18·1)
**Mauritania**	12306 (9089, 16251)	944·8 (708·1, 1269)	14252 (9995, 19271)	557·9 (396·4, 757·8)	-40·9 (-54·2, -23·7)
**Niger**	44112 (30396, 62317)	902·4 (656·1, 1371)	61380 (22491, 98988)	518·9 (164·3, 873·7)	-42·5 (-76·9, -13·3)
**Nigeria**	1134833 (600489, 1741364)	1958 (967·4, 3102·1)	1623367 (754087, 2697630)	1325·9 (589·9, 2230·2)	-32·3 (-52·3, 0)
**São Tomé and Príncipe**	1780 (1232, 2193)	2121·5 (1420·4, 2662·2)	2759 (2148, 3873)	1984·1 (1533·6, 2770·6)	-6·5 (-36·6, 31)
**Senegal**	35786 (23680, 48626)	759·9 (494·8, 1050·5)	49965 (24238, 74333)	522·7 (235·7, 781·3)	-31·2 (-55·1, -12)
**Sierra Leone**	37276 (26297, 54420)	1472 (1015·8, 2155)	39458 (17498, 56634)	820·5 (352, 1179·8)	-44·3 (-83, -0·6)
**Togo**	22825 (17624, 29731)	1143·8 (871, 1497·9)	27843 (10575, 41549)	560·4 (202·2, 850·4)	-51 (-85·4, -15·3)
**Eastern sub-Saharan Africa**	1302658 (1057855, 1600427)	1290·3 (1070·7, 1543·1)	1942504 (1498999, 2340490)	917·5 (707·6, 1105)	-28·9 (-47·8, -3)
**Burundi**	57737 (37353, 86475)	1974·7 (1279·1, 2906·3)	59326 (19853, 94832)	1041·2 (332·3, 1663·3)	-47·3 (-84·1, -11)
**Comoros**	1642 (334, 2825)	602·7 (104·9, 1044·6)	2737 (687, 4253)	511·7 (124·8, 794·3)	-15·1 (-34·9, 24·7)
**Djibouti**	1790 (1187, 2589)	733·5 (491·7, 1053·6)	4904 (3170, 7090)	646·5 (412·6, 925·3)	-11·9 (-38·5, 27·5)
**Eritrea**	19672 (11184, 32144)	1376·4 (869·2, 2205·2)	41149 (31060, 54247)	1221·7 (941·6, 1570)	-11·2 (-39·6, 43·3)
**Ethiopia**	371207 (228365, 583703)	1391·8 (887·4, 2117·3)	524217 (439909, 616423)	991·5 (845, 1171·3)	-28·8 (-55·5, 22·1)
**Kenya**	169349 (109550, 239694)	1555·8 (1001·2, 2203·3)	338240 (224938, 466322)	1227·4 (820·9, 1695·4)	-21·1 (-51·4, 3·6)
**Madagascar**	73862 (52870, 112271)	1032·1 (748·9, 1525·8)	104531 (77513, 131138)	713·2 (524·9, 897)	-30·9 (-61, 8·4)
**Malawi**	73614 (36201, 97353)	1395·8 (783·4, 1804·9)	86430 (20607, 136308)	889·2 (208·1, 1389·3)	-36·3 (-86·6, 45·9)
**Mozambique**	69875 (46287, 91850)	799·9 (536·2, 1065·5)	90551 (19906, 144289)	544 (109·7, 868·8)	-32 (-87·3, 37·3)
**Rwanda**	90751 (69954, 108164)	2369·2 (1839·6, 2818·8)	86965 (55901, 122322)	1169·4 (752·5, 1629·6)	-50·6 (-69·1, -26)
**Somalia**	34525 (14345, 62012)	912 (441·4, 1573·9)	88201 (54341, 132713)	1003·4 (627, 1483·8)	10 (-28·5, 94·7)
**South Sudan**	27267 (13426, 47001)	871·6 (478·6, 1459)	48915 (32785, 71056)	927·1 (625·4, 1337·5)	6·4 (-29·7, 77·9)
**Tanzania**	147022 (98302, 190042)	1035·8 (747·8, 1331·9)	204710 (93393, 293985)	643 (279·9, 951·1)	-37·9 (-73·4, 0·2)
**Uganda**	89946 (57165, 126052)	1048·9 (669·6, 1486·7)	149135 (64395, 223473)	790 (328·7, 1176·7)	-24·7 (-57, 7·9)
**Zambia**	73736 (52704, 90124)	1856·4 (1418, 2229·7)	111273 (92856, 131234)	1236·3 (1033·7, 1448·3)	-33·4 (-47·9, -10·8)
**Central sub-Saharan Africa**	383144 (286930, 506821)	1176·1 (877·8, 1583)	752822 (598548, 950901)	1018·9 (795·5, 1312·6)	-13·4 (-28·3, 8·8)
**Angola**	78981 (43467, 125015)	1334·2 (742·6, 2138·7)	195238 (145570, 264544)	1237·7 (922·3, 1717·5)	-7·2 (-35, 57·1)
**Central African Republic**	20253 (11203, 29519)	1206 (689·9, 1724·1)	26504 (11796, 43648)	856·1 (373·6, 1390·3)	-29 (-52·2, -4·6)

**Congo**	21136<br>(14633, 31613)	1448·7<br>(996·6, 2208·3)	35703<br>(23503, 51082)	1038·7<br>(680·9, 1498·6)	-28·3<br>(-47, 0·6)
**DR Congo**	248725<br>(183181, 330680)	1099·9<br>(809, 1490·3)	475995<br>(357892, 632326)	959·2<br>(709·1, 1281·2)	-12·8<br>(-32·1, 12·9)
**Equatorial Guinea**	2882<br>(931, 4968)	1075·8<br>(346·2, 1864·7)	5534<br>(3172, 8908)	791<br>(445·6, 1268·5)	-26·5<br>(-55·8, 59·9)
**Gabon**	11167<br>(7762, 15111)	1682·1<br>(1158·6, 2296·5)	13848<br>(9575, 19748)	1073·3<br>(727·5, 1554·9)	-36·2<br>(-50·3, -17·7)

Appendix Table 5: Prevalent cases and age-standardised prevalence rates of decompensated cirrhosis per 100 000 in 1990 and 2017 for both sexes combined by location

	1990		2017	
	**Counts (95% UI)**	**Rate (95% UI)**	**Counts (95% UI)**	**Rate (95% UI)**
**Global**	5203234 (5079997, 5324997)	110·6 (108, 113)	10643731 (10319645, 10945143)	132·5 (128·6, 136·2)
**High-income North America**	315799 (308627, 322956)	96·8 (94·5, 99)	569044 (554217, 584023)	112·3 (109·3, 115·1)
**Canada**	27723 (27001, 28501)	87·8 (85·5, 90·2)	55842 (53937, 57537)	104 (100·5, 107·1)
**Greenland**	34 (33, 35)	69·3 (67·1, 71·4)	58 (56, 60)	85·6 (82·9, 88·5)
**USA**	288035 (281553, 294438)	97·8 (95·6, 100)	513134 (499947, 526521)	113·2 (110·3, 116·1)
**Australasia**	12022 (11709, 12348)	54·0 (52·6, 55·4)	28385 (27449, 29220)	75·7 (73·3, 78)
**Australia**	9897 (9633, 10177)	53·2 (51·8, 54·7)	24150 (23326, 24903)	76·3 (73·9, 78·6)
**New Zealand**	2125 (2065, 2185)	57·8 (56·1, 59·4)	4235 (4101, 4373)	72·7 (70·5, 75·1)
**High-income Asia-Pacific**	521335 (509825, 533006)	260·7 (255·2, 266·2)	774152 (751160, 797770)	267·4 (259·8, 275·1)
**Brunei**	316 (307, 325)	175 (169·6, 180)	948 (915, 980)	230·5 (222·4, 237·8)
**Japan**	404257 (395402, 413184)	257·3 (251·9, 262·9)	571878 (555078, 588980)	279·8 (272, 287·8)
**Singapore**	4051 (3942, 4161)	139·9 (136·2, 143·7)	13307 (12899, 13744)	195·8 (190·1, 201·9)
**South Korea**	112711 (109566, 115604)	292·3 (284·3, 299·7)	188019 (181153, 194862)	245·9 (237·8, 253·9)
**Western Europe**	623132 (609143, 637088)	130·8 (127·9, 133·7)	1057823 (1024683, 1090218)	172·3 (167·2, 177·5)
**Andorra**	74 (71, 76)	122·2 (118·1, 126·4)	205 (198, 213)	175·1 (169, 181·7)
**Austria**	18634 (18141, 19120)	196 (191, 201·2)	27193 (26285, 28090)	216·9 (210, 223·7)
**Belgium**	14079 (13695, 14465)	113·1 (110·1, 116·1)	26634 (25761, 27518)	169·2 (163·7, 174·6)
**Cyprus**	602 (581, 622)	73·9 (71·4, 76·4)	1834 (1772, 1897)	112·5 (108·8, 116·3)
**Denmark**	7064 (6874, 7263)	112·8 (109·8, 115·9)	14704 (14198, 15214)	186 (179·8, 192·3)
**Finland**	6407 (6225, 6585)	106·3 (103·4, 109·2)	14532 (14048, 15017)	186 (180·2, 191·9)
**France**	93739 (91362, 96225)	136·1 (132·6, 139·7)	147882 (143046, 152890)	164·1 (159, 169·5)

**Germany**	142580 (138927, 146353)	138 (134·4, 141·5)	244706 (236440, 253210)	198·3 (191·8, 205·1)
**Greece**	13354 (12993, 13705)	102·5 (99·8, 105·2)	24005 (23221, 24792)	157·8 (153, 163·1)
**Iceland**	213 (206, 221)	81·5 (78·8, 84·3)	736 (710, 763)	174 (167·9, 180·1)
**Ireland**	3099 (3009, 3189)	83·8 (81·4, 86·3)	9869 (9557, 10209)	161·9 (156·8, 167·2)
**Israel**	4368 (4254, 4491)	91·7 (89·3, 94·3)	14693 (14245, 15204)	152·6 (147·9, 157·8)
**Italy**	139254 (135650, 142831)	187·6 (182·9, 192·4)	198790 (192109, 205230)	217·6 (210·8, 224·9)
**Luxembourg**	798 (776, 822)	167 (162·3, 172)	1835 (1771, 1901)	232·6 (225, 240·7)
**Malta**	289 (280, 299)	68·9 (66·9, 71·1)	693 (670, 717)	110·2 (106·7, 114·1)
**Netherlands**	15425 (15000, 15846)	88·7 (86·2, 91·1)	36465 (35217, 37678)	152·3 (147·4, 157·4)
**Norway**	5379 (5238, 5530)	110·6 (107·5, 113·7)	9635 (9337, 9935)	141·2 (137, 145·4)
**Portugal**	14234 (13833, 14636)	114·2 (111, 117·4)	21075 (20287, 21881)	132·8 (127·8, 137·7)
**Spain**	65480 (63748, 67121)	136·7 (133·3, 140·2)	111406 (107597, 115134)	165·5 (159·9, 171)
**Sweden**	9538 (9281, 9811)	90·6 (88, 93·2)	18983 (18346, 19685)	141·6 (136·8, 146·8)
**Switzerland**	8520 (8278, 8755)	98·7 (95·9, 101·3)	16505 (15938, 17089)	134·2 (129·8, 138·5)
**UK**	59403 (57827, 60986)	88 (85·7, 90·3)	114347 (110763, 117833)	129·4 (125·5, 133·5)
**Southern Latin America**	52308 (51122, 53583)	108·7 (106·3, 111·4)	135014 (130995, 139076)	180·2 (174·9, 185·6)
**Argentina**	31809 (31035, 32635)	96·4 (94·1, 98·9)	84857 (82224, 87748)	173·6 (168·1, 179·3)
**Chile**	17382 (16927, 17842)	152·6 (148·7, 156·7)	42927 (41510, 44319)	197 (190·4, 203·1)
**Uruguay**	3115 (3028, 3206)	87·6 (85·2, 90·1)	7224 (6995, 7462)	169 (163·6, 174·3)
**Eastern Europe**	356970 (348120, 365986)	138·6 (135·2, 142)	670132 (651068, 689734)	244·2 (237·2, 251·4)
**Belarus**	15905 (15474, 16359)	136·7 (132·8, 140·6)	29113 (28145, 30093)	231·8 (224·7, 239·2)
**Estonia**	2645 (2566, 2728)	147·6 (143·3, 152·1)	4170 (4045, 4309)	239·3 (232·1, 247·2)
**Latvia**	4181 (4056, 4308)	133·5 (129·5, 137·7)	5642 (5458, 5839)	215·8 (208·7, 223)
**Lithuania**	6430 (6254, 6598)	155·6 (151·2, 159·6)	9868 (9537, 10179)	258·4 (250·3, 266·3)
**Moldova**	16043 (15561, 16527)	336·6 (326·8, 346·2)	14427 (13984, 14902)	289·6 (281·1, 298·3)

**Russia**	219624 (213795, 225159)	129·8 (126·5, 133·1)	451809 (438889, 465275)	238 (231·2, 245)
**Ukraine**	92143 (89648, 94690)	148·1 (144·2, 151·9)	155102 (150077, 160289)	264 (255·6, 272·7)
**Central Europe**	279739 (272896, 286246)	196·8 (192·1, 201·3)	416866 (405102, 430196)	255·4 (248·2, 263·4)
**Albania**	4356 (4220, 4492)	159·4 (154·4, 164·5)	8723 (8414, 9033)	242·3 (234·2, 250·9)
**Bosnia and Herzegovina**	8307 (8036, 8572)	176·8 (171·3, 182·2)	11554 (11130, 11993)	237 (228·9, 245·1)
**Bulgaria**	18879 (18393, 19418)	168·2 (163·9, 173)	25132 (24260, 26046)	238 (230·4, 245·8)
**Croatia**	14069 (13690, 14462)	228·8 (222·9, 234·9)	14266 (13819, 14757)	224 (217·3, 231·6)
**Czech Republic**	20821 (20228, 21438)	169·8 (165·1, 174·9)	34778 (33674, 35971)	226·7 (219·6, 234·1)
**Hungary**	37546 (36503, 38628)	286 (278·1, 294)	34271 (33172, 35514)	240 (232·7, 247·6)
**Montenegro**	1026 (995, 1059)	157·4 (152·5, 162·2)	1910 (1845, 1979)	229·8 (222·3, 237·9)
**North Macedonia**	3243 (3138, 3350)	157·8 (152·9, 162·9)	6766 (6521, 7010)	233 (225, 240·9)
**Poland**	72197 (70233, 74087)	170 (165·5, 174·6)	145697 (141228, 150795)	275·1 (266·7, 284·4)
**Romania**	62096 (60430, 63865)	225·8 (220·1, 232)	79091 (76602, 81810)	275·3 (266·9, 284·2)
**Serbia**	14248 (13727, 14777)	125·6 (121·3, 130·1)	21148 (20392, 21963)	171·8 (165·8, 178·2)
**Slovakia**	16894 (16384, 17419)	294·5 (285·9, 303·3)	26200 (25305, 27107)	349·6 (338·4, 361·2)
**Slovenia**	6058 (5899, 6227)	254·1 (247·4, 260·7)	7329 (7083, 7588)	235·7 (228·4, 243·3)
**Central Asia**	76397 (74602, 78172)	130·8 (127·8, 133·8)	175051 (169931, 180324)	201·8 (196·1, 207·8)
**Armenia**	3729 (3630, 3836)	116·2 (113·1, 119·5)	7143 (6930, 7371)	193·5 (187·9, 199·5)
**Azerbaijan**	7992 (7763, 8215)	129·3 (125·8, 132·9)	20764 (20046, 21463)	196·6 (189·8, 203·1)
**Georgia**	8102 (7883, 8314)	131·9 (128·5, 135·3)	9336 (9058, 9628)	195·5 (189·7, 201·4)
**Kazakhstan**	16445 (15986, 16882)	107·4 (104·4, 110·2)	36511 (35350, 37767)	199·3 (193·2, 205·9)
**Kyrgyzstan**	5292 (5149, 5430)	145·6 (141·9, 149·4)	11437 (11072, 11798)	206 (199·6, 212·2)
**Mongolia**	2065 (2002, 2127)	136·4 (132·3, 140·4)	5236 (5069, 5419)	178·4 (172·9, 184·2)
**Tajikistan**	5287 (5134, 5446)	133·9 (130·2, 137·7)	14195 (13719, 14686)	193·5 (187·1, 199·9)
**Turkmenistan**	4072 (3968, 4177)	149·5 (145·6, 153·2)	10207 (9879, 10512)	217·5 (210·9, 223·9)

**Uzbekistan**	23414 (22819, 24022)	150·4 (146·6, 154·1)	60221 (58427, 62202)	208·3 (202·4, 214·8)
**Central Latin America**	160477 (156992, 164009)	144·6 (141·5, 147·8)	509311 (494117, 524421)	206·6 (200·4, 212·6)
**Colombia**	20492 (19921, 21045)	85·4 (83·1, 87·7)	93332 (90050, 96461)	174·9 (168·8, 180·7)
**Costa Rica**	2557 (2493, 2626)	116 (112·8, 119·2)	10253 (9914, 10600)	204·1 (197·4, 210·9)
**El Salvador**	4086 (3977, 4195)	112·2 (109, 115·4)	10986 (10621, 11354)	192 (185·6, 198·5)
**Guatemala**	6351 (6185, 6512)	123·4 (120·2, 126·7)	23327 (22541, 24133)	187·1 (180·9, 193·7)
**Honduras**	3500 (3387, 3611)	121·8 (117·7, 125·5)	13191 (12717, 13666)	189·1 (182·4, 195·9)
**Mexico**	105601 (103298, 107904)	187·3 (183·2, 191·2)	281656 (272995, 290108)	228·9 (222·1, 235·6)
**Nicaragua**	2595 (2518, 2666)	116·2 (112·9, 119·6)	10302 (9963, 10662)	198·3 (191·6, 205·4)
**Panama**	1800 (1748, 1852)	98·9 (96·1, 102)	7373 (7125, 7624)	184 (177·9, 190·2)
**Venezuela**	13496 (13168, 13847)	102·9 (100·4, 105·7)	58891 (56750, 61073)	192·6 (185·8, 199·6)
**Andean Latin America**	18440 (17969, 18919)	70·6 (68·9, 72·5)	80489 (78332, 82777)	142·3 (138·5, 146·5)
**Bolivia**	2574 (2493, 2656)	60·5 (58·7, 62·5)	12514 (12129, 12909)	129·1 (125, 133·4)
**Ecuador**	4709 (4590, 4823)	68·5 (66·8, 70·2)	22211 (21585, 22896)	144·3 (140·2, 148·7)
**Peru**	11157 (10855, 11484)	74·3 (72·3, 76·3)	45765 (44504, 47179)	145·3 (141·2, 149·9)
**Caribbean**	19775 (19296, 20237)	67·7 (66·1, 69·3)	54465 (52766, 56260)	108·9 (105·6, 112·5)
**Antigua and Barbuda**	37 (36, 38)	69·5 (67·4, 71·5)	114 (111, 118)	111·9 (108·5, 115·5)
**The Bahamas**	142 (138, 146)	73·5 (71·4, 75·8)	427 (412, 441)	106·3 (103, 109·9)
**Barbados**	172 (168, 177)	66 (64, 67·9)	455 (439, 471)	109·9 (106·3, 113·5)
**Belize**	83 (80, 85)	69·2 (67·2, 71·2)	357 (346, 369)	113·1 (109·6, 116·8)
**Bermuda**	48 (47, 50)	74·8 (72·8, 77·1)	124 (120, 128)	121·2 (117·3, 125·3)
**Cuba**	6465 (6298, 6641)	61·2 (59·6, 62·8)	18815 (18174, 19462)	115·7 (112, 119·4)
**Dominica**	43 (42, 44)	64 (62, 65·9)	91 (88, 94)	109·4 (105·8, 112·8)
**Dominican Republic**	3548 (3453, 3642)	73·5 (71·5, 75·6)	10926 (10548, 11294)	111·9 (108·1, 115·7)
**Grenada**	48 (47, 50)	68 (66, 70·2)	139 (135, 144)	106·4 (102·9, 110)

**Guyana**	393 (382, 404)	76 (73·9, 78)	711 (687, 736)	103·3 (99·9, 106·7)
**Haiti**	2239 (2163, 2317)	51·3 (49·6, 53·1)	7495 (7241, 7757)	85·5 (82·6, 88·4)
**Jamaica**	1103 (1073, 1135)	57·7 (56, 59·4)	3076 (2969, 3186)	106·7 (103, 110·5)
**Puerto Rico**	3729 (3636, 3828)	102·2 (99·6, 104·9)	6779 (6551, 7017)	123·3 (119·2, 127·6)
**Saint Lucia**	74 (72, 76)	73·5 (71·4, 75·7)	226 (218, 234)	110·2 (106·8, 113·9)
**Saint Vincent and the Grenadines**	53 (51, 54)	64 (62·2, 66·1)	143 (138, 148)	109·6 (106·1, 113·5)
**Suriname**	211 (204, 216)	69·7 (67·6, 71·7)	647 (626, 670)	107·3 (104, 111)
**Trinidad and Tobago**	638 (620, 655)	64·5 (62·7, 66·3)	1803 (1740, 1864)	105·8 (102·1, 109·1)
**Virgin Islands**	73 (70, 75)	73 (70·9, 75·1)	179 (173, 186)	115·6 (111·9, 119·6)
**Tropical Latin America**	94178 (91967, 96311)	80·0 (78·1, 81·7)	216579 (210211, 222811)	91·3 (88·7, 93·9)
**Brazil**	92150 (89987, 94240)	80·2 (78·4, 82)	210481 (204287, 216507)	91·1 (88·5, 93·7)
**Paraguay**	2028 (1965, 2090)	70·3 (68·2, 72·7)	6099 (5899, 6303)	101·8 (98·4, 105·1)
**East Asia**	1372763 (1334572, 1411311)	121·9 (118·6, 125·1)	2986859 (2886002, 3084172)	155·1 (150·2, 159·8)
**China**	1317922 (1280743, 1355313)	123·2 (119·8, 126·5)	2856264 (2759334, 2949376)	155·9 (151, 160·7)
**North Korea**	14405 (13919, 14872)	74·3 (71·9, 76·6)	37646 (36312, 38959)	123·1 (118·9, 127·1)
**Taiwan (province of China)**	17567 (17142, 18010)	95 (92·7, 97·3)	44832 (43409, 46264)	134·9 (130·9, 138·9)
**Southeast Asia**	129083 (125528, 132900)	36·4 (35·4, 37·4)	358408 (346420, 370727)	54·2 (52·5, 55·9)
**Cambodia**	2179 (2116, 2245)	31·7 (30·7, 32·7)	7188 (6933, 7440)	51·9 (50, 53·6)
**Indonesia**	61169 (59363, 62972)	43·7 (42·5, 45)	112444 (108525, 116389)	45 (43·6, 46·4)
**Laos**	894 (866, 921)	30·5 (29·6, 31·5)	2896 (2799, 2997)	51·6 (49·9, 53·3)
**Malaysia**	3781 (3661, 3896)	28·1 (27·2, 28·9)	16502 (15920, 17075)	56·1 (54·3, 58)
**Maldives**	46 (44, 47)	31·6 (30·5, 32·7)	258 (248, 268)	60·9 (58·9, 63)
**Mauritius**	357 (346, 367)	37·8 (36·8, 38·8)	871 (842, 901)	55·5 (53·7, 57·2)
**Myanmar**	9610 (9303, 9902)	31·1 (30·1, 32·1)	25455 (24525, 26349)	49·7 (48, 51·4)
**Philippines**	7939 (7676, 8190)	16·8 (16·3, 17·3)	37619 (36069, 39091)	41·8 (40·2, 43·4)

**Sri Lanka**	4767 (4628, 4904)	33·5 (32·5, 34·5)	14000 (13533, 14493)	57·4 (55·5, 59·3)
**Seychelles**	20 (20, 21)	33·1 (32·1, 34·2)	70 (68, 73)	61·8 (59·7, 63·8)
**Thailand**	15584 (15110, 16088)	33 (32, 34)	54550 (52692, 56631)	59·1 (57·2, 61·1)
**East Timor**	155 (150, 160)	30·2 (29·1, 31·2)	525 (509, 543)	54·1 (52·2, 56)
**Vietnam**	22411 (21642, 23244)	43·4 (42, 45·1)	85558 (81824, 89140)	85·4 (82, 88·6)
**Oceania**	2028 (1966, 2091)	40·7 (39·5, 41·9)	6387 (6168, 6605)	61·6 (59·6, 63·6)
**American Samoa**	19 (18, 20)	50 (48·5, 51·5)	42 (41, 44)	80·2 (77·5, 82·8)
**Federated States of Micronesia**	37 (36, 38)	47·6 (45·9, 49·1)	66 (64, 68)	70·3 (68, 72·8)
**Fiji**	235 (227, 243)	37·8 (36·6, 39)	577 (556, 598)	65 (62·8, 67·4)
**Guam**	64 (62, 66)	55 (53·3, 56·7)	149 (144, 154)	82·9 (80·1, 85·5)
**Kiribati**	26 (26, 27)	44·5 (43·1, 45·9)	68 (65, 70)	67·3 (64·9, 69·6)
**Marshall Islands**	15 (15, 16)	47·2 (45·8, 48·8)	35 (34, 36)	71·5 (69·1, 73·9)
**Northern Mariana Islands**	22 (21, 22)	58 (56·2, 59·9)	47 (45, 49)	84·9 (82·2, 87·6)
**Papua New Guinea**	1206 (1168, 1247)	38·4 (37·3, 39·7)	4329 (4171, 4488)	58·3 (56·3, 60·4)
**Samoa**	66 (64, 69)	53·2 (51·4, 55)	142 (137, 147)	82·6 (79·9, 85·5)
**Solomon Islands**	108 (104, 111)	43·9 (42·5, 45·3)	341 (329, 354)	66·5 (64·3, 68·8)
**Tonga**	40 (39, 42)	53 (51·5, 54·6)	77 (75, 80)	83·1 (80·3, 85·9)
**Vanuatu**	56 (55, 58)	49·9 (48·2, 51·6)	162 (157, 168)	68·8 (66·3, 71·1)
**North Africa and Middle East**	334976 (325704, 344552)	134·9 (131·4, 138·5)	799410 (776591, 822307)	156·2 (151·8, 160·7)
**Afghanistan**	10845 (10470, 11199)	126·1 (122, 130)	29000 (27997, 30100)	139·7 (135·3, 144·2)
**Algeria**	24935 (24196, 25653)	138·1 (134·1, 142·3)	61230 (59326, 63134)	167·2 (162, 172·4)
**Bahrain**	513 (498, 529)	150·1 (145·7, 154·7)	2434 (2346, 2524)	176·4 (171, 182)
**Egypt**	71042 (69048, 73192)	181·7 (176·8, 186·6)	137046 (132768, 141310)	180·2 (174·9, 185·9)
**Iran**	52004 (50500, 53489)	118·9 (115·8, 122·1)	114950 (111811, 118307)	145·8 (141·9, 149·8)
**Iraq**	16013 (15503, 16537)	130·9 (127·2, 134·7)	47426 (45958, 48929)	142·5 (138·4, 146·7)

**Jordan**	3667 (3523, 3820)	147·9 (142·9, 152·9)	15434 (14847, 16036)	185·3 (178·5, 191·7)
**Kuwait**	1685 (1633, 1738)	134 (130·3, 137·7)	6449 (6228, 6674)	180·8 (175·1, 186·3)
**Lebanon**	4227 (4099, 4351)	138·3 (134·1, 142·4)	12711 (12320, 13117)	179·8 (174·1, 185·5)
**Libya**	4118 (3987, 4247)	143·9 (139·5, 148·1)	10164 (9849, 10500)	176·8 (171·4, 182·6)
**Morocco**	25057 (24310, 25812)	130 (126·2, 133·8)	53527 (51889, 55289)	157 (152·2, 161·9)
**Palestine**	1916 (1857, 1980)	140 (136, 144·3)	5860 (5675, 6048)	164·9 (160, 169·8)
**Oman**	1889 (1829, 1949)	150 (145·5, 154·4)	5936 (5723, 6144)	179 (173·1, 184·7)
**Qatar**	472 (455, 488)	164·7 (159·9, 169·7)	3992 (3845, 4151)	195·9 (189·4, 202·3)
**Saudi Arabia**	15844 (15362, 16335)	154 (149·4, 158·8)	48368 (46733, 50060)	186 (180·5, 192)
**Sudan**	19667 (19025, 20325)	139·3 (135·1, 143·5)	44114 (42730, 45439)	160·8 (155·8, 166·1)
**Syria**	11614 (11258, 11996)	135·1 (131, 139·1)	26080 (25253, 26905)	167·7 (162·5, 173)
**Tunisia**	9118 (8839, 9407)	137·7 (133·7, 142)	20531 (19883, 21175)	168·5 (163·2, 173·5)
**Turkey**	46917 (45291, 48543)	103·7 (100·1, 107·2)	109211 (105489, 113203)	126·7 (122·4, 131·2)
**United Arab Emirates**	1794 (1729, 1858)	149·6 (145, 154·3)	14631 (14002, 15288)	179·2 (173·3, 185·1)
**Yemen**	11424 (11065, 11818)	128·8 (124·7, 133)	29571 (28623, 30527)	144·4 (139·9, 148·9)
**South Asia**	410653 (398707, 422195)	44·7 (43·5, 45·9)	882628 (857168, 908094)	53·6 (52·1, 55)
**Bangladesh**	41925 (40618, 43254)	49·8 (48·3, 51·2)	79002 (76541, 81632)	54·2 (52·5, 56)
**Bhutan**	227 (220, 235)	53 (51·4, 54·7)	502 (484, 520)	58·8 (56·8, 60·8)
**India**	320134 (310930, 329405)	43·6 (42·4, 44·8)	696047 (676065, 716235)	53·5 (52, 55)
**Nepal**	6874 (6651, 7093)	44·9 (43·5, 46·3)	13622 (13188, 14048)	50·9 (49·3, 52·6)
**Pakistan**	41493 (40173, 42795)	48·9 (47·3, 50·3)	93454 (90361, 96611)	53·6 (51·8, 55·4)
**Southern sub-Saharan Africa**	38854 (37795, 40030)	93·5 (91·1, 96)	57200 (55565, 58859)	82·7 (80·4, 85)
**Botswana**	761 (738, 787)	77·9 (75·5, 80·3)	1649 (1600, 1704)	85·9 (83·4, 88·8)
**eSwatini**	458 (444, 474)	80·4 (78, 82·8)	729 (705, 755)	82·7 (80·2, 85·5)
**Lesotho**	1172 (1135, 1213)	80·5 (78·1, 83·1)	1298 (1255, 1341)	79·5 (76·9, 82·1)

**Namibia**	699 (676, 723)	63·8 (61·8, 65·8)	1499 (1453, 1548)	77·4 (74·9, 80)
**South Africa**	28673 (27897, 29587)	95·7 (93·2, 98·4)	41851 (40679, 43038)	81·5 (79·3, 83·8)
**Zimbabwe**	7091 (6858, 7332)	94·5 (91·8, 97·2)	10174 (9845, 10524)	89·6 (86·8, 92·3)
**Western sub-Saharan Africa**	190794 (185667, 195881)	142·0 (138·1, 146)	431691 (419424, 445004)	147·5 (143·3, 151·9)
**Benin**	4825 (4679, 4978)	148·4 (144, 153)	12096 (11751, 12490)	155·4 (150·9, 160·1)
**Burkina Faso**	11647 (11306, 11999)	170·1 (165·2, 174·6)	23702 (22948, 24521)	164·6 (159·1, 170·2)
**Cameroon**	9717 (9420, 10005)	137 (133·1, 141·1)	26976 (26104, 27866)	142·7 (138·3, 147·3)
**Cape Verde**	410 (398, 421)	151·9 (147·4, 156·4)	791 (768, 816)	163·4 (158·6, 168·4)
**Chad**	6773 (6556, 7001)	158·6 (153·8, 163·5)	14996 (14511, 15530)	155·2 (150·4, 160·2)
**Côte d'Ivoire**	10693 (10357, 11034)	135·4 (131·4, 139·5)	24565 (23785, 25403)	141·9 (137·6, 146·8)
**The Gambia**	972 (941, 1003)	151·4 (146·7, 156·2)	2341 (2271, 2414)	159 (154·2, 164)
**Ghana**	12891 (12509, 13260)	125·3 (121·7, 129)	31251 (30252, 32281)	138·8 (134·3, 143·2)
**Guinea**	6785 (6587, 6999)	147·3 (142·9, 151·9)	12106 (11731, 12520)	148 (143·5, 152·6)
**Guinea-Bissau**	1011 (981, 1042)	145·9 (141·5, 150·5)	1797 (1739, 1853)	143·5 (139·2, 147·9)
**Liberia**	2412 (2340, 2486)	159·5 (155, 164·4)	5429 (5258, 5607)	166·5 (161·4, 172)
**Mali**	10712 (10382, 11047)	172·6 (167·5, 177·6)	24654 (23845, 25478)	182·8 (177, 188·8)
**Mauritania**	2082 (2022, 2147)	140·8 (136·5, 145·2)	4382 (4252, 4520)	154 (149·5, 159·1)
**Niger**	8919 (8636, 9208)	169 (164, 174·1)	23960 (23141, 24828)	177·8 (172·3, 183·5)
**Nigeria**	84733 (82316, 87208)	133·6 (129·5, 137·5)	189264 (183555, 195508)	139·6 (135·1, 144·2)
**São Tomé and Príncipe**	156 (152, 161)	175·7 (170·8, 180·8)	265 (257, 274)	178·7 (173·1, 184·5)
**Senegal**	7538 (7310, 7776)	144·4 (139·9, 148·8)	16487 (15924, 17013)	155·6 (150·5, 160·5)
**Sierra Leone**	5062 (4915, 5222)	181·9 (176·6, 187·5)	8663 (8381, 8943)	159·9 (154·8, 165)
**Togo**	3449 (3340, 3560)	145·9 (141·5, 150)	7963 (7704, 8232)	146·3 (141·7, 151·2)
**Eastern sub-Saharan Africa**	147505 (143344, 151841)	122·7 (119·4, 126·1)	323090 (313747, 332844)	128·6 (124·8, 132·4)
**Burundi**	4105 (3979, 4237)	118·3 (114·7, 121·8)	8445 (8162, 8729)	122·3 (118·3, 126·6)

**Comoros**	374 (362, 386)	122·7 (118·9, 126·6)	757 (732, 783)	132·4 (127·9, 136·9)
**Djibouti**	366 (355, 378)	124·6 (121·1, 128·2)	1155 (1120, 1192)	139·3 (134·9, 143·8)
**Eritrea**	1963 (1900, 2032)	112·7 (109·1, 116·4)	4573 (4430, 4719)	119·8 (116·2, 123·6)
**Ethiopia**	39336 (38213, 40496)	121·4 (117·9, 124·8)	83981 (81606, 86419)	127·7 (124·1, 131·2)
**Kenya**	18071 (17588, 18593)	134 (130·3, 137·7)	43088 (41872, 44383)	134·5 (130·7, 138·5)
**Madagascar**	9605 (9296, 9908)	123·1 (119·3, 126·9)	21840 (21110, 22573)	128·9 (124·8, 133)
**Malawi**	7971 (7728, 8235)	128·2 (124·3, 132·2)	14801 (14327, 15275)	132·5 (128·4, 136·9)
**Mozambique**	10827 (10492, 11165)	112·3 (109, 115·8)	23480 (22729, 24310)	127·2 (123·3, 131·5)
**Rwanda**	6311 (6116, 6502)	138·3 (134·1, 142·5)	11283 (10929, 11650)	131·9 (127·7, 136·5)
**Somalia**	5202 (5035, 5382)	114·2 (110·5, 117·8)	13133 (12691, 13557)	123·3 (119·1, 127·2)
**South Sudan**	4975 (4817, 5147)	131·6 (127·5, 136·3)	8608 (8335, 8904)	136·9 (132·7, 141·5)
**Tanzania**	19973 (19384, 20586)	120 (116·6, 123·6)	45878 (44344, 47464)	129·1 (124·9, 133·6)
**Uganda**	12490 (12098, 12875)	117·7 (113·8, 121·2)	28877 (27948, 29860)	123·8 (119·8, 127·8)
**Zambia**	5862 (5673, 6032)	123·9 (120·2, 127·6)	12988 (12580, 13373)	120·9 (117·3, 124·6)
**Central sub-Saharan Africa**	46006 (44566, 47414)	123·2 (119·5, 126·9)	110747 (107304, 114290)	131·8 (127·8, 136·1)
**Angola**	8796 (8504, 9099)	129·1 (124·8, 133·4)	24850 (24063, 25688)	133·5 (129·4, 137·9)
**Central African Republic**	1991 (1928, 2061)	104·8 (101·6, 108·2)	3725 (3606, 3852)	110·6 (107, 114)
**Congo**	1775 (1719, 1836)	105·9 (102·6, 109·5)	4329 (4192, 4471)	115·7 (112·1, 119·3)
**DR Congo**	32241 (31192, 33271)	124·4 (120·5, 128·4)	74998 (72498, 77555)	133·9 (129·6, 138·5)
**Equatorial Guinea**	339 (328, 351)	113·3 (109·9, 117·1)	1145 (1108, 1182)	133·9 (129·6, 138·5)
**Gabon**	863 (839, 889)	117·3 (113·9, 120·9)	1699 (1645, 1753)	123·9 (120, 127·9)

Appendix Table 6: Prevalent cases and age-standardised prevalence rates of compensated cirrhosis per 100 000 in 1990 and 2017 for both sexes combined by location

	1990		2017	
	Counts (95% UI)	Rate (95% UI)	Counts (95% UI)	Rate (95% UI)
**Global**	65947703 (63387294, 68744980)	1354·5 (1300·6, 1411·7)	112363787 (106563283, 118533648)	1395·0 (1323·5, 1470·5)
**High-income North America**	1154736 (1096397, 1210694)	361·9 (344·5, 379·3)	1827033 (1726898, 1923029)	372·2 (352·9, 391·6)
**Canada**	92868 (87394, 98463)	295·7 (278·2, 313·3)	159085 (146793, 170963)	309·2 (286·3, 331·8)
**Greenland**	135 (126, 145)	253·1 (235·8, 270)	203 (186, 219)	291·5 (268·8, 313·9)
**USA**	1061707 (1008400, 1111883)	369·3 (351·2, 387·4)	1667713 (1577300, 1753150)	379·6 (360·3, 399)
**Australasia**	116360 (111434, 121772)	531·6 (509·7, 556·7)	189719 (178728, 200928)	563·1 (531·2, 596·2)
**Australia**	94668 (90391, 99125)	516·8 (493·7, 541·4)	157920 (148408, 167724)	553·4 (521·3, 586·8)
**New Zealand**	21692 (20639, 22801)	603·6 (574·5, 634)	31798 (29961, 33692)	615·4 (582·2, 652·6)
**High-income Asia Pacific**	5935372 (5737229, 6139500)	3077·8 (2972·9, 3186·6)	5682247 (5406606, 5967781)	2455 (2344·9, 2575·8)
**Brunei**	4736 (4489, 4983)	2047 (1945·3, 2152·7)	10203 (9652, 10802)	2245·6 (2122·1, 2374·3)
**Japan**	3865916 (3724253, 4011413)	2658·4 (2559·7, 2760)	3871612 (3677934, 4067292)	2443·7 (2332, 2566·2)
**Singapore**	62582 (59762, 65414)	1914·3 (1829, 2000·6)	122654 (116059, 129680)	1919·3 (1812·4, 2029·8)
**South Korea**	2002138 (1928253, 2075664)	4607·5 (4448·7, 4775·8)	1677778 (1589127, 1762453)	2537·9 (2410·8, 2663·2)
**Western Europe**	6573349 (6365747, 6785623)	1460·5 (1414·9, 1507·4)	7937663 (7525465, 8362885)	1456·5 (1386·3, 1532·8)
**Andorra**	740 (699, 782)	1170·1 (1105·3, 1234·5)	1461 (1366, 1557)	1350·8 (1267·2, 1435·2)
**Austria**	186235 (177073, 202887)	2063·2 (1964·5, 2233·4)	217276 (206472, 228148)	1928·2 (1834·2, 2021·7)
**Belgium**	143455 (137343, 149491)	1214·3 (1165, 1265·5)	187650 (177533, 199532)	1340·1 (1273·2, 1419·8)
**Cyprus**	6674 (6316, 7098)	819·3 (775·5, 871·1)	13457 (12618, 14352)	859·6 (808·7, 915·7)
**Denmark**	60076 (57400, 62646)	996·8 (953·3, 1038·8)	104205 (98494, 110338)	1493·6 (1416·4, 1578·4)
**Finland**	55593 (53044, 58199)	944·5 (901, 987·4)	100549 (94983, 106593)	1493·5 (1417·9, 1578·9)
**France**	1002131 (963491, 1041081)	1524·9 (1467·5, 1581)	1037465 (976855, 1102260)	1315·6 (1244·7, 1394·7)

**Germany**	1407397 (1359942, 1458831)	1447·4 (1399, 1497·8)	1822601 (1721917, 1925840)	1688·9 (1604·4, 1780·8)
**Greece**	149473 (143019, 155967)	1222·6 (1171·2, 1274·4)	154483 (145125, 163799)	1155·9 (1091, 1224·9)
**Iceland**	1493 (1418, 1574)	569·9 (541·7, 600·5)	3845 (3598, 4116)	989·8 (927·8, 1057·1)
**Ireland**	26358 (25114, 27580)	725·2 (691·4, 758·5)	65846 (62068, 70158)	1150·1 (1089·3, 1223·7)
**Israel**	46249 (44225, 48312)	978·9 (936·4, 1021·9)	108697 (102718, 115613)	1174·7 (1111·2, 1249)
**Italy**	1516088 (1458470, 1574971)	2214·6 (2133, 2299·2)	1536168 (1449451, 1627198)	1913·1 (1813·6, 2020·2)
**Luxembourg**	8515 (8101, 8910)	1820·1 (1735·6, 1901·7)	14675 (13821, 15616)	1959·8 (1851·8, 2076·2)
**Malta**	2964 (2835, 3102)	710·9 (680·8, 743·7)	4261 (4015, 4537)	778·5 (736·5, 826)
**Netherlands**	164390 (156881, 171932)	952·4 (909·3, 993·8)	240863 (225842, 256941)	1140·4 (1070·4, 1213·4)
**Norway**	54885 (52314, 57613)	1151·1 (1099·4, 1208·5)	82893 (78376, 87776)	1342·9 (1272·6, 1420·6)
**Portugal**	185171 (177854, 192518)	1615·2 (1553·1, 1675·5)	140174 (130895, 150925)	1016·1 (952·8, 1087·7)
**Spain**	764499 (739809, 796968)	1723·7 (1667·2, 1796·2)	818843 (773592, 866632)	1349·1 (1278·1, 1426)
**Sweden**	108747 (103929, 114142)	1089·3 (1043·3, 1141)	189524 (179584, 200883)	1585·6 (1501·7, 1677·9)
**Switzerland**	76043 (72534, 79387)	916 (875·3, 954·8)	109487 (103284, 115938)	1002 (947·7, 1058·8)
**UK**	599834 (573566, 627191)	919·9 (881·4, 961·4)	975013 (927753, 1027361)	1213·1 (1156·5, 1277·1)
**Southern Latin America**	629728 (607657, 652050)	1316·0 (1270·1, 1361·7)	1120623 (1060773, 1178396)	1540·2 (1459·1, 1619·7)
**Argentina**	378756 (364178, 394330)	1164·9 (1119·6, 1212·7)	692909 (652322, 730989)	1453·5 (1369·3, 1533·2)
**Chile**	217791 (209852, 225667)	1823·3 (1756·6, 1886·1)	377096 (357774, 395943)	1779·7 (1690·5, 1866·9)
**Uruguay**	33155 (31714, 34473)	996·2 (953·2, 1035·3)	50569 (47620, 53607)	1287·1 (1216·1, 1364·2)
**Eastern Europe**	3854642 (3697193, 4020387)	1556·2 (1493·4, 1625·4)	5719309 (5454966, 6010972)	2283·4 (2183·2, 2395·8)
**Belarus**	155158 (147517, 163877)	1370·5 (1301·3, 1448·2)	231686 (218482, 245417)	2031·3 (1919·6, 2147·5)
**Estonia**	22994 (21794, 24263)	1341·7 (1269·9, 1417·3)	29954 (28164, 31895)	1920 (1811·4, 2041)
**Latvia**	38188 (36226, 40113)	1275·6 (1210, 1341·2)	42232 (39895, 45039)	1816·2 (1720·9, 1928·8)
**Lithuania**	59773 (56939, 62973)	1482·2 (1411·8, 1558·6)	75555 (71289, 80029)	2234 (2115·8, 2363·3)
**Moldova**	155473 (149690, 161584)	3388·5 (3264·6, 3520·1)	137489 (130242, 145196)	3000·4 (2842·8, 3166·4)

**Russia**	2505834 (2392741, 2621703)	1521·2 (1454·3, 1591·8)	3913270 (3730449, 4116688)	2252·7 (2147·3, 2364·8)
**Ukraine**	917222 (877590, 961069)	1579·9 (1511·8, 1659·1)	1289123 (1225960, 1356217)	2411·2 (2296·9, 2532·6)
**Central Europe**	2730726 (2631165, 2826082)	2022·5 (1946·4, 2094·8)	2906876 (2764240, 3059980)	2084·2 (1985·9, 2194·2)
**Albania**	46455 (44070, 48947)	1568·4 (1489·1, 1651·7)	57096 (53416, 61071)	1822·5 (1709·5, 1945)
**Bosnia and Herzegovina**	84451 (80578, 88507)	1773·9 (1691, 1859·5)	74800 (70112, 79502)	1814·6 (1702·2, 1930·6)
**Bulgaria**	178219 (170682, 186155)	1751·4 (1677·3, 1831·4)	170043 (160543, 180226)	1952·4 (1848·9, 2068·5)
**Croatia**	135481 (129555, 140891)	2391·3 (2286·2, 2488·7)	102365 (96894, 107835)	1933·5 (1838·7, 2030·1)
**Czech Republic**	188404 (179890, 197180)	1636 (1562·3, 1710·4)	224091 (211882, 236432)	1719·2 (1629·8, 1810)
**Hungary**	336374 (323867, 349023)	2814·2 (2708·4, 2920)	237847 (225842, 250524)	1996·9 (1896·4, 2104·8)
**Montenegro**	8239 (7796, 8703)	1274·7 (1206·4, 1347·1)	11328 (10641, 12108)	1566·3 (1475·4, 1670·1)
**North Macedonia**	28293 (26857, 29853)	1369·6 (1299·4, 1445·8)	42021 (39456, 44748)	1630·7 (1533·5, 1735·2)
**Poland**	737923 (706816, 773942)	1775·7 (1699·9, 1862·1)	1007331 (948109, 1073134)	2181 (2055·7, 2318·5)
**Romania**	654328 (630293, 678960)	2580·5 (2485·4, 2679·8)	617146 (585947, 649790)	2554·9 (2425·4, 2686·1)
**Serbia**	117244 (111300, 123646)	1117·5 (1060·8, 1178)	127244 (119744, 135155)	1216·7 (1148·9, 1288·9)
**Slovakia**	158560 (151216, 166466)	2845·8 (2713·9, 2988·2)	186083 (174800, 197064)	2828·6 (2672, 2989)
**Slovenia**	56755 (54506, 59152)	2521·2 (2421·3, 2622·3)	49482 (46520, 52357)	1902·1 (1799·1, 2013)
**Central Asia**	756000 (725504, 787983)	1229·3 (1181·1, 1280·7)	1443250 (1360853, 1529019)	1550·8 (1464·2, 1641·5)
**Armenia**	31056 (29437, 32730)	935·5 (888·4, 984·5)	42999 (40318, 45831)	1259·1 (1183, 1341·9)
**Azerbaijan**	77753 (73920, 81989)	1185·6 (1130·3, 1247·5)	152614 (142680, 163415)	1365·5 (1278·5, 1459·5)
**Georgia**	65685 (62683, 68752)	1127·7 (1078·2, 1180·4)	51727 (45879, 55480)	1241·4 (1097·6, 1335·9)
**Kazakhstan**	155573 (148069, 163541)	968·6 (924·4, 1016·4)	291051 (273420, 309384)	1541·4 (1450·1, 1636·7)
**Kyrgyzstan**	53407 (50906, 56141)	1417·8 (1351·8, 1488·2)	99282 (93598, 104876)	1622 (1527·9, 1713·8)
**Mongolia**	25902 (24463, 27483)	1557·4 (1465·6, 1650)	51643 (48431, 55101)	1524·4 (1427·6, 1625·5)
**Tajikistan**	53057 (50189, 55934)	1237·6 (1177·7, 1300·3)	116479 (109406, 124543)	1371 (1290·3, 1459·9)
**Turkmenistan**	44996 (42899, 47206)	1512·5 (1445·4, 1580·7)	94340 (89182, 100192)	1841·6 (1740·5, 1954·7)

**Uzbekistan**	248570 (237337, 260450)	1474·5 (1408·9, 1538·7)	543115 (512595, 576273)	1677·3 (1584·3, 1778·9)
**Central Latin America**	2407025 (2328935, 2482875)	2043·3 (1975·9, 2108·5)	5758402 (5492758, 6038576)	2272·3 (2167, 2383·4)
**Colombia**	275131 (262397, 288247)	1097 (1045·8, 1151)	768374 (725745, 814359)	1449·6 (1368·8, 1537·3)
**Costa Rica**	33145 (31604, 34671)	1439 (1372·6, 1502)	97787 (92047, 103527)	1926·8 (1814·9, 2039·7)
**El Salvador**	63119 (60412, 65845)	1676·8 (1600·8, 1750·3)	130665 (122684, 138734)	2257·2 (2116·2, 2397·7)
**Guatemala**	112089 (107789, 116154)	2078·9 (1998·1, 2154·3)	340447 (323388, 357795)	2546·8 (2415·2, 2673·1)
**Honduras**	62969 (60080, 66032)	2103·1 (2000·5, 2209·4)	186521 (174926, 199500)	2522·2 (2363·1, 2696·9)
**Mexico**	1605519 (1556102, 1653391)	2673 (2589·6, 2753·7)	3508527 (3346555, 3663839)	2749·8 (2621·8, 2873)
**Nicaragua**	36838 (35155, 38513)	1574·3 (1500·9, 1650)	113341 (107193, 120129)	2045·3 (1936·4, 2165·5)
**Panama**	22826 (21730, 23973)	1201·4 (1141·5, 1263·7)	65217 (61628, 69011)	1621·8 (1531·9, 1716)
**Venezuela**	195388 (186276, 204230)	1398·2 (1336·9, 1462·1)	547522 (515425, 581984)	1726·8 (1626·3, 1836·4)
**Andean Latin America**	346704 (332166, 361643)	1180·4 (1131, 1230·3)	906156 (855141, 956163)	1541·4 (1454·5, 1626·6)
**Bolivia**	53163 (50202, 56074)	1123·1 (1059·6, 1184·9)	164191 (153407, 174544)	1592·7 (1486·6, 1694)
**Ecuador**	86626 (83179, 90451)	1115·9 (1071·2, 1161·8)	251822 (237720, 265106)	1572·1 (1482·1, 1655·7)
**Peru**	206914 (197255, 216573)	1225·2 (1169·6, 1282·3)	490143 (462360, 518680)	1510·4 (1424·2, 1598·1)
**Caribbean**	303268 (290337, 316180)	1004·6 (960·6, 1047·3)	615530 (579170, 653381)	1240·3 (1167·6, 1315·1)
**Antigua and Barbuda**	506 (481, 532)	955·5 (909·2, 1005·2)	1193 (1120, 1267)	1147·2 (1076·5, 1217·7)
**The Bahamas**	2400 (2280, 2520)	1124·3 (1068·7, 1183·1)	5152 (4838, 5458)	1220·7 (1145·3, 1292)
**Barbados**	2165 (2055, 2282)	853·6 (811·5, 898·5)	4059 (3806, 4311)	1072·2 (1004·6, 1141·8)
**Belize**	1273 (1206, 1338)	1035·5 (983·7, 1088·7)	4753 (4475, 5025)	1360·8 (1280·4, 1437·4)
**Bermuda**	616 (585, 648)	917·9 (871·8, 964·9)	980 (917, 1045)	1074·4 (1005·8, 1145·2)
**Cuba**	84755 (80232, 89218)	788 (746·3, 829·1)	172531 (162016, 183393)	1148·8 (1079·9, 1219·3)
**Dominica**	547 (520, 575)	856·6 (814·2, 900·3)	883 (828, 938)	1124·9 (1057·5, 1197·3)
**Dominican Republic**	65224 (62029, 68559)	1238·6 (1175·1, 1302·7)	137410 (127918, 146575)	1339·3 (1247·7, 1428·1)
**Grenada**	701 (666, 736)	1039·9 (986, 1093)	1403 (1319, 1489)	1152·7 (1084·8, 1222·6)

**Guyana**	7742 (7355, 8129)	1363·2 (1294·8, 1431)	10551 (9919, 11185)	1447·7 (1360·6, 1534·8)
**Haiti**	43781 (41031, 46325)	940·1 (878·8, 995·8)	127756 (118621, 137171)	1287·4 (1194, 1376)
**Jamaica**	13991 (13317, 14739)	731·5 (696·4, 771·3)	29899 (28003, 31839)	1023·7 (958·1, 1090·3)
**Puerto Rico**	52710 (50345, 55141)	1455·7 (1391·1, 1522·6)	64057 (60254, 67743)	1351·7 (1273, 1430·5)
**Saint Lucia**	1116 (1059, 1170)	1103·9 (1046·3, 1159·2)	2459 (2313, 2615)	1194·7 (1125·3, 1270·3)
**Saint Vincent and the Grenadines**	724 (685, 763)	884·9 (839·5, 931·1)	1450 (1365, 1543)	1135·3 (1068·1, 1206·5)
**Suriname**	3647 (3476, 3821)	1143·6 (1088·9, 1198·9)	8444 (7938, 8980)	1366·3 (1284·3, 1452·9)
**Trinidad and Tobago**	9826 (9339, 10293)	946·9 (900, 992·9)	18573 (17397, 19845)	1115·6 (1046·6, 1192·3)
**Virgin Islands**	1164 (1103, 1226)	1087·9 (1029·9, 1145·7)	1841 (1723, 1959)	1346 (1263·6, 1429·1)
**Tropical Latin America**	1358901 (1303250, 1415474)	1071·6 (1026·8, 1115·8)	2464317 (2338368, 2589457)	1010·2 (959·2, 1060·4)
**Brazil**	1335400 (1280809, 1391331)	1078·4 (1033·4, 1122·7)	2401071 (2278151, 2521879)	1010·5 (959·5, 1060·6)
**Paraguay**	23502 (22123, 24985)	790 (743·4, 838·9)	63246 (58691, 67668)	1001·9 (930·3, 1073·7)
**East Asia**	22340734 (21365407, 23435590)	1901·3 (1813·9, 1993·1)	38719143 (36466683, 41091034)	2012·9 (1904·7, 2131·4)
**China**	21124232 (20197693, 22183413)	1892·9 (1805·2, 1985·4)	36435366 (34297849, 38673072)	1991·4 (1883·1, 2109·9)
**North Korea**	326833 (310108, 344844)	1631·1 (1546·5, 1720·1)	720873 (678581, 769035)	2328·6 (2193·1, 2480·9)
**Taiwan (province of China)**	517467 (498332, 539344)	2618·4 (2517·2, 2723·3)	939161 (891939, 985827)	2913·7 (2772·9, 3052·9)
**Southeast Asia**	1823474 (1724938, 1923879)	480·8 (454·5, 507·2)	3952897 (3686888, 4214112)	563·8 (527·4, 600·1)
**Cambodia**	38407 (36301, 40692)	534·5 (503·6, 567·1)	101098 (94235, 108234)	684 (635·6, 732·6)
**Indonesia**	887611 (837110, 936420)	582·5 (549·4, 615·7)	1351031 (1259164, 1443368)	498·4 (466, 530·7)
**Laos**	13071 (12269, 13861)	430·5 (403·4, 458·5)	36250 (33649, 38696)	587·1 (543·9, 628·5)
**Malaysia**	43224 (40791, 45799)	300·7 (284·2, 317·8)	141440 (132094, 151404)	456·4 (426·1, 487·9)
**Maldives**	469 (439, 500)	308·3 (289·3, 327·6)	2097 (1958, 2252)	441·6 (413·4, 471·7)
**Mauritius**	4828 (4591, 5067)	481·8 (458·3, 505·8)	7342 (6889, 7785)	470·5 (442, 499·4)
**Myanmar**	174939 (163660, 185798)	538·2 (501·6, 571·9)	348334 (324296, 372609)	637·6 (593·8, 681·4)

**Philippines**	94782 (90211, 100050)	190·4 (181·8, 200·3)	315388 (285703, 337295)	328·4 (298·4, 351·3)
**Sri Lanka**	63101 (59678, 66745)	412·9 (390·3, 437·2)	123707 (115201, 132477)	509·7 (475·2, 544·3)
**Seychelles**	243 (229, 257)	396·8 (373·7, 418·4)	687 (639, 738)	576·5 (538·5, 615·8)
**Thailand**	202020 (189893, 213441)	391·7 (368·4, 413·6)	478903 (447270, 510156)	524 (490·5, 556·6)
**East Timor**	2154 (2012, 2294)	376·2 (352·1, 401·5)	5673 (5261, 6081)	575·3 (530·1, 617·5)
**Vietnam**	296204 (278108, 313701)	569·2 (533·3, 602·5)	1035745 (960564, 1107096)	973·5 (904·2, 1038·3)
**Oceania**	37308 (35252, 39481)	719·7 (682·5, 760·2)	95922 (89911, 102404)	861·8 (807·5, 917·5)
**American Samoa**	260 (247, 276)	664·1 (629·3, 702·8)	427 (400, 456)	799·3 (750, 854·7)
**Federated States of Micronesia**	639 (601, 680)	824·7 (775·8, 875·1)	868 (811, 928)	888·8 (829·6, 951)
**Fiji**	3246 (3069, 3445)	497·9 (471·3, 527·1)	6368 (5973, 6789)	696·4 (652·6, 742·3)
**Guam**	1070 (1012, 1131)	852·5 (807, 900·6)	1639 (1542, 1738)	926·9 (873, 984·5)
**Kiribati**	487 (457, 517)	798·7 (749·4, 846·8)	1000 (931, 1071)	949·4 (883·4, 1015·4)
**Marshall Islands**	267 (251, 283)	841·7 (792·7, 892·1)	495 (462, 531)	942·8 (880·1, 1011·3)
**Northern Mariana Islands**	356 (336, 378)	834·1 (790·6, 881·5)	487 (455, 519)	904·2 (846·4, 962·8)
**Papua New Guinea**	23982 (22640, 25436)	736·9 (696·5, 779·5)	69731 (65311, 74383)	867·4 (811·8, 921·2)
**Samoa**	980 (924, 1043)	774 (730·9, 822·1)	1556 (1454, 1668)	899·8 (840·5, 964·6)
**Solomon Islands**	1831 (1727, 1950)	750·9 (709·4, 795·2)	4721 (4425, 5044)	877 (823·1, 935·5)
**Tonga**	634 (599, 673)	837·9 (790·9, 887·3)	920 (861, 985)	982·5 (919·8, 1052·1)
**Vanuatu**	1094 (1027, 1170)	954·6 (893·7, 1019·1)	2423 (2252, 2601)	995·3 (924·6, 1068)
**North Africa and Middle East**	4231679 (3998952, 4475038)	1593·4 (1505·7, 1679·7)	9347394 (8772972, 9935923)	1634·8 (1535·9, 1736·9)
**Afghanistan**	137510 (129007, 146562)	1682·3 (1577·6, 1790·8)	444613 (414843, 477026)	1912·2 (1783·3, 2036·2)
**Algeria**	277264 (259896, 295078)	1434 (1349·2, 1519·4)	647675 (606059, 693729)	1608·2 (1505·3, 1721)
**Bahrain**	6801 (6392, 7244)	1569·8 (1476·5, 1664·6)	27213 (25383, 29129)	1599·1 (1493·6, 1707·6)
**Egypt**	1267454 (1192747, 1345291)	2921·5 (2747·6, 3098·6)	2071082 (1932283, 2210508)	2406·9 (2247·3, 2572·7)

**Iran**	517372 (485255, 551568)	1139·6 (1074·3, 1206·2)	1153426 (1082550, 1229211)	1346·4 (1264·7, 1433)
**Iraq**	173263 (162371, 184411)	1331·7 (1255·7, 1408·9)	487621 (455350, 519333)	1318·7 (1237·1, 1401·4)
**Jordan**	44969 (42254, 47934)	1629·5 (1536·2, 1724·7)	181207 (169865, 193412)	1903·1 (1784·2, 2030·8)
**Kuwait**	17483 (16445, 18554)	1120·5 (1056·9, 1186·8)	63934 (60050, 68318)	1379·4 (1298·7, 1472·1)
**Lebanon**	46934 (44168, 49862)	1434·2 (1351·4, 1517·6)	132682 (123826, 142009)	1687·8 (1577·5, 1802·5)
**Libya**	46363 (43643, 49407)	1477·5 (1395·7, 1569·8)	117319 (109621, 125488)	1720·9 (1610·7, 1836·9)
**Morocco**	297545 (280397, 317433)	1438·7 (1352·6, 1529·1)	589525 (551154, 629243)	1633·3 (1528·4, 1743·7)
**Palestine**	20258 (18885, 21581)	1405·2 (1319·6, 1489·3)	63891 (59496, 68264)	1591·7 (1487·4, 1693·6)
**Oman**	22396 (20954, 23931)	1497·7 (1406·2, 1589·6)	73119 (68014, 78297)	1627·7 (1521·3, 1730·9)
**Qatar**	6552 (6147, 7004)	1617·3 (1526·1, 1714·9)	50021 (46657, 53896)	1686·1 (1583·6, 1798·2)
**Saudi Arabia**	195432 (182849, 207664)	1631·3 (1530·8, 1732·6)	620553 (578117, 663755)	1770·3 (1653·6, 1892·5)
**Sudan**	254608 (239206, 271604)	1703·1 (1600·9, 1809·2)	577751 (539624, 619167)	1854·6 (1727·8, 1980·3)
**Syria**	131035 (122672, 139440)	1414·7 (1331·2, 1493·7)	273306 (255943, 291219)	1626 (1524·1, 1731·3)
**Tunisia**	97224 (91350, 103538)	1379·6 (1299·4, 1466·9)	201323 (188110, 215321)	1613·3 (1508·3, 1726·5)
**Turkey**	495209 (464617, 527161)	1002 (941·9, 1064·3)	950431 (891961, 1012312)	1087·3 (1021, 1156·9)
**United Arab Emirates**	24808 (23140, 26626)	1499·8 (1405, 1597·3)	209468 (193797, 226352)	1720·5 (1601·1, 1843·9)
**Yemen**	148470 (139254, 158369)	1602·1 (1506·3, 1699·9)	402504 (376129, 431062)	1724·4 (1611·7, 1838·7)
**South Asia**	5061248 (4776766, 5369182)	534·9 (505·6, 566·3)	10174230 (9504231, 10854055)	588·3 (549·3, 627·9)
**Bangladesh**	581031 (550632, 616862)	677·8 (642·6, 717·3)	894821 (838444, 956287)	586·4 (549·5, 626·1)
**Bhutan**	3041 (2869, 3234)	702 (660·4, 744·6)	5901 (5469, 6353)	629·3 (584·7, 675·5)
**India**	3834111 (3611599, 4068470)	504·6 (476·1, 534·5)	7920418 (7396535, 8456054)	581·3 (542·6, 620·3)
**Nepal**	89651 (84194, 95290)	578·7 (544·1, 615·4)	154550 (140947, 166531)	561·6 (512, 605)
**Pakistan**	553414 (521673, 588368)	660·3 (621·8, 702·1)	1198541 (1117231, 1283184)	645·7 (603·1, 689·9)
**Southern sub-Saharan Africa**	545717 (516449, 577644)	1301·2 (1231, 1375·6)	747141 (697989, 796627)	1029·0 (963, 1096·8)
**Botswana**	10204 (9573, 10887)	1071·9 (1007·1, 1143·7)	23029 (21501, 24637)	1092·2 (1020·9, 1166·9)

**eSwatini**	6877 (6466, 7326)	1261·3 (1187·2, 1341·1)	10867 (10146, 11670)	1180·4 (1104·7, 1262·5)
**Lesotho**	16204 (15129, 17301)	1160·5 (1085·6, 1235·6)	19651 (18269, 21150)	1146·9 (1066·7, 1232·7)
**Namibia**	9230 (8625, 9856)	866·7 (812·1, 921·4)	20834 (19463, 22356)	1033 (967·1, 1105·4)
**South Africa**	397305 (375457, 420674)	1290·1 (1220·9, 1365·4)	516301 (483219, 549392)	956·4 (895·2, 1017·8)
**Zimbabwe**	105897 (99761, 112471)	1486·4 (1400·9, 1574·8)	156459 (145848, 168349)	1341·4 (1247·7, 1436·1)
**Western sub-Saharan Africa**	2714638 (2561626, 2876475)	1950·4 (1839·1, 2064·5)	5970623 (5568528, 6378365)	1883·5 (1756·6, 2010·9)
**Benin**	64112 (60213, 68112)	1959·7 (1838, 2077·2)	156580 (146708, 167677)	1872 (1753, 2001)
**Burkina Faso**	149718 (141698, 158505)	2242·5 (2113·6, 2368·1)	333715 (309882, 359877)	2191·5 (2036·9, 2353·5)
**Cameroon**	141340 (133259, 149696)	1929·1 (1819·2, 2038·6)	375973 (350779, 402859)	1827·9 (1699·5, 1953·6)
**Cape Verde**	3937 (3729, 4163)	1520 (1441·7, 1605)	8606 (8115, 9181)	1653 (1557·3, 1758·2)
**Chad**	84899 (79583, 90110)	2019·9 (1891·2, 2141·6)	200360 (187712, 214631)	2007·7 (1880·6, 2146·4)
**Côte d'Ivoire**	157835 (148553, 167549)	1862·4 (1752·1, 1974·8)	358886 (335900, 383360)	1844 (1728·3, 1965·3)
**The Gambia**	12456 (11693, 13243)	1844·9 (1734·7, 1955·8)	30326 (28336, 32460)	1917 (1797·3, 2041)
**Ghana**	189862 (179549, 200913)	1741·7 (1646, 1842·5)	435721 (404390, 467226)	1751·2 (1626·9, 1878·2)
**Guinea**	89803 (84402, 95445)	1955·4 (1837·3, 2077·4)	165748 (154401, 177483)	1931·9 (1804, 2066·1)
**Guinea-Bissau**	14536 (13710, 15418)	2088 (1967·5, 2214)	26545 (24844, 28406)	1948·2 (1820·4, 2083·2)
**Liberia**	32461 (30562, 34358)	2165·5 (2036·1, 2294)	75349 (70514, 80582)	2064·3 (1929·3, 2205·1)
**Mali**	144672 (136049, 154169)	2304·3 (2165·9, 2449·2)	317793 (295478, 341736)	2268·6 (2115·9, 2428·5)
**Mauritania**	26080 (24570, 27685)	1733·6 (1635·4, 1838·3)	51300 (47852, 54868)	1712·9 (1598·8, 1829·4)
**Niger**	113383 (106240, 120401)	2086·9 (1957·8, 2218·7)	315839 (292648, 340054)	2286·8 (2126, 2454·9)
**Nigeria**	1276277 (1199500, 1356523)	1912 (1796·5, 2033·5)	2669443 (2487962, 2859043)	1798·3 (1674·8, 1925·7)
**São Tomé and Príncipe**	2252 (2129, 2388)	2605·8 (2463·2, 2761·2)	3927 (3671, 4204)	2399·4 (2244·9, 2565)
**Senegal**	90850 (85351, 96643)	1708·3 (1606·6, 1816·1)	209927 (195487, 225198)	1869·3 (1743·1, 1998·9)
**Sierra Leone**	71332 (66987, 75739)	2518·1 (2364·4, 2665·8)	122532 (114382, 131471)	2073·3 (1933·1, 2222·8)
**Togo**	48757 (45749, 51783)	1976·1 (1863·5, 2092·7)	111994 (104233, 120136)	1864·1 (1740·3, 1998·1)

**Eastern sub-Saharan Africa**	2368680 (2227599, 2511003)	1834·2 (1724·7, 1946·7)	5175192 (4837925, 5532017)	1836·2 (1717·9, 1958·3)
**Burundi**	75875 (71166, 81012)	2021·8 (1897·8, 2151·1)	146762 (136402, 157814)	1888·4 (1755·8, 2029·3)
**Comoros**	5137 (4805, 5495)	1587·6 (1483·9, 1696·2)	11103 (10355, 11931)	1776·6 (1658·1, 1906·7)
**Djibouti**	5309 (4990, 5660)	1554·7 (1463·3, 1650·1)	17243 (16065, 18466)	1742·2 (1630·1, 1859·9)
**Eritrea**	34911 (32655, 37154)	1805·5 (1691·6, 1921·8)	84023 (78556, 90146)	1882·5 (1759·2, 2013·4)
**Ethiopia**	630751 (592049, 670143)	1816·7 (1704·5, 1925·1)	1298745 (1215056, 1387272)	1780 (1667·7, 1898·6)
**Kenya**	300800 (282519, 320339)	2063·1 (1937·5, 2192·7)	706807 (660084, 756716)	1918·8 (1792·1, 2049)
**Madagascar**	144286 (135699, 153548)	1721·2 (1621·2, 1827·3)	334865 (312460, 357951)	1740·5 (1630·8, 1860·3)
**Malawi**	135590 (127263, 144116)	2043·8 (1916·5, 2170·9)	249305 (231517, 268025)	2020·4 (1875·1, 2172)
**Mozambique**	164885 (155611, 175460)	1589·6 (1500·9, 1689·4)	387728 (360379, 416171)	1872·6 (1743·8, 2011·2)
**Rwanda**	116259 (109311, 123225)	2383·3 (2236·1, 2528·6)	190541 (177459, 204168)	1987·7 (1852, 2130·2)
**Somalia**	83961 (78392, 89553)	1649·8 (1545, 1758·3)	219985 (204951, 235444)	1857·5 (1730·4, 1986·6)
**South Sudan**	72579 (67993, 77740)	1760·7 (1649·1, 1878·3)	133524 (124482, 143209)	1905·1 (1777·9, 2040)
**Tanzania**	295141 (277798, 313009)	1674 (1575·4, 1776·2)	691455 (644772, 741931)	1755·2 (1634·7, 1882·3)
**Uganda**	196684 (184611, 208996)	1759·2 (1649, 1874·3)	471299 (438456, 505293)	1830·2 (1702·6, 1959·9)
**Zambia**	105308 (98678, 112013)	2057 (1928·8, 2184·4)	228559 (213756, 244369)	1857·1 (1742·9, 1986·7)
**Central sub-Saharan Africa**	657416 (617987, 699806)	1683·1 (1583, 1786·7)	1610123 (1501437, 1720285)	1765·8 (1649·4, 1885·5)
**Angola**	130648 (122096, 139227)	1788 (1675·9, 1904·5)	356773 (332755, 381498)	1777·2 (1659·5, 1902)
**Central African Republic**	30919 (28950, 32928)	1543·7 (1446·8, 1644)	61752 (57472, 66429)	1654·5 (1537·6, 1778·7)
**Congo**	27297 (25598, 29056)	1580·5 (1483·5, 1681·7)	66405 (61740, 71209)	1574·2 (1460·6, 1685·7)
**DR Congo**	451089 (424791, 480559)	1672·4 (1572, 1779·3)	1086844 (1011625, 1163884)	1791 (1671·8, 1917·9)
**Equatorial Guinea**	4656 (4354, 4960)	1525·9 (1429·3, 1627·2)	15088 (14071, 16166)	1579·8 (1473·6, 1691·8)
**Gabon**	12807 (12040, 13592)	1722·5 (1616·9, 1828·9)	23262 (21686, 24907)	1555·7 (1454, 1665·3)

Appendix Table 7: Proportion of causes for deaths due to cirrhosis in 2017 in both sexes

	Hepatitis B	Hepatitis C	Alcohol	NASH	Other causes
**Global**	29·0	25·9	25·1	8·9	11·1
**High-income North America**	5·7	34·7	28·2	10·7	20·6
**Canada**	6	31	31·8	9·4	21·9
**Greenland**	6·1	34·4	32·3	9·7	17·6
**USA**	5·7	35·0	27·9	10·8	20·6
**Australasia**	16·7	36·2	28·2	11·7	7·1
**Australia**	16·6	36·3	28·4	11·7	7·1
**New Zealand**	17·3	35·7	27·2	12·2	7·5
**High-income Asia Pacific**	21·2	41·3	22·1	4·7	10·6
**Brunei**	33·8	23·7	25·6	5·2	11·7
**Japan**	15·9	53·8	15·2	4·6	10·6
**Singapore**	54·2	9·7	16·6	2·7	16·8
**South Korea**	34·7	8·8	40·9	5·2	10·4
**Western Europe**	8·1	21·4	41·7	9·5	19·3
**Andorra**	7·9	18·7	45·7	9·2	18·5
**Austria**	7·1	17·8	47·2	9·2	18·7
**Belgium**	9·6	14·6	53·5	7·5	14·8
**Cyprus**	8·2	18·1	42·4	9·9	21·4
**Denmark**	8·4	17·2	47·3	9·2	17·9
**Finland**	8·9	18·4	45·1	10·9	16·7
**France**	7·9	17·5	45·9	8·9	19·7
**Germany**	7·4	17·2	45·5	10·1	19·9
**Greece**	10·1	16·7	41·5	10·1	21·7
**Iceland**	7·5	7·6	39·7	17·1	28·1
**Ireland**	8·7	17·8	44·1	10·4	19
**Israel**	8·7	19·8	32·9	11·8	26·8
**Italy**	10·8	48·5	18·6	6	16·1
**Luxembourg**	7·3	17·2	46·6	9·8	19
**Malta**	8·2	18·2	40·9	12·1	20·6
**Netherlands**	8·4	17·1	40	10·6	23·9
**Norway**	8·7	17·8	42·2	9·8	21·6
**Portugal**	8	18·4	43·9	10·3	19·4

**Spain**	7·6	19·7	52·2	11·6	8·9
**Sweden**	8·7	16·3	42	11	22·1
**Switzerland**	8·8	17·7	43·2	9·1	21·2
**United Kingdom**	6·2	8·7	44·3	10·8	30
**Southern Latin America**	12·9	29·4	34·6	9·3	13·8
**Argentina**	12·8	28·9	35·6	8·8	14
**Chile**	12·9	29·9	33·7	10·1	13·4
**Uruguay**	13·4	30·4	29·6	10·2	16·4
**Eastern Europe**	18·2	24·4	37·3	11	9
**Belarus**	16·7	23·2	40·5	10·7	8·8
**Estonia**	16·2	22·3	43·2	10·3	8
**Latvia**	16·8	23·3	40·1	11	8·7
**Lithuania**	16·8	23·1	41·7	10·5	7·9
**Moldova**	17·9	24·9	35·5	11·8	10
**Russia**	18·1	24·7	36·7	11·2	9·4
**Ukraine**	18·7	24	38·5	10·6	8·2
**Central Europe**	22·3	20·4	44	7·2	6
**Albania**	25·2	22·4	36·6	7·2	8·6
**Bosnia and Herzegovina**	25·6	23	36·2	8·1	7·1
**Bulgaria**	22·9	20·6	43·8	6·9	5·8
**Croatia**	23·2	20·3	43	7·2	6·3
**Czech Republic**	21·2	19·9	46	6·9	5·9
**Hungary**	24·5	19·4	43·5	7·2	5·3
**Montenegro**	24·1	21·8	40·9	7·3	5·9
**North Macedonia**	25	22·4	37·7	8·3	6·6
**Poland**	21·7	20·4	45·1	7·1	5·7
**Romania**	21·7	20·4	44	7·3	6·5
**Serbia**	23·1	21·6	41·5	7·5	6·3
**Slovakia**	21·7	20·3	45·3	7·1	5·5
**Slovenia**	22·6	20·8	42·7	7·8	6·1
**Central Asia**	21·9	24	36·6	8·1	9·4
**Armenia**	21·3	25	34·9	8·3	10·5
**Azerbaijan**	22·3	24·2	35·5	8·8	9·2
**Georgia**	21·9	24·2	38·5	8·2	7·3
**Kazakhstan**	20·6	23·8	39·5	8	8

**Kyrgyzstan**	22·9	25·1	36	7·7	8·4
**Mongolia**	21·9	24·8	36·6	7·7	9
**Tajikistan**	21·7	23·4	33·7	7	14·2
**Turkmenistan**	22·5	24·2	35·8	8·1	9·4
**Uzbekistan**	22·3	23·8	35·6	8·2	10·1
**Central Latin America**	6·3	31·1	36·6	16·1	9·9
**Colombia**	6·9	27·8	40·1	14·6	10·6
**Costa Rica**	6·2	28·3	40·9	16·2	8·4
**El Salvador**	6·7	30·2	36·8	16·9	9·5
**Guatemala**	7·6	33·2	33·3	15·3	10·5
**Honduras**	7	32·2	32·8	16·5	11·4
**Mexico**	6	31·4	36·6	16·2	9·9
**Nicaragua**	6·6	30·9	35·7	18·1	8·8
**Panama**	6·9	30·3	37·9	14	10·9
**Venezuela**	6·8	29·6	38·6	17·3	7·8
**Andean Latin America**	11·7	13·7	38·1	22·2	14·3
**Bolivia**	11·6	14·1	37	22·4	14·9
**Ecuador**	11·9	14·4	33	25·2	15·5
**Peru**	11·5	13·1	41·8	20·2	13·4
**Caribbean**	11	22·7	34·8	19·1	12·4
**Antigua and Barbuda**	10·1	22·1	33·2	22·3	12·3
**The Bahamas**	10·3	22·6	36·5	19·4	11·1
**Barbados**	10·4	22·1	34·3	21·8	11·5
**Belize**	10·6	22·4	34·6	20·7	11·8
**Bermuda**	11	20	36·1	22	10·9
**Cuba**	10·4	23	34·8	20·7	11·1
**Dominica**	10·4	21·8	34·6	20	13·2
**Dominican Republic**	10·8	22·1	35·4	18	13·7
**Grenada**	10·3	22·3	35·3	19·7	12·4
**Guyana**	10·8	23·3	35·9	19·5	10·5
**Haiti**	12·5	23·7	35·1	15·9	12·8
**Jamaica**	10·7	22·7	32·1	20·4	14·1
**Puerto Rico**	10·1	21·7	34·1	21·9	12·3
**Saint Lucia**	10	21·9	34·7	21·7	11·7
**Saint Vincent and the Grenadines**	10·2	21·4	34·2	24·5	9·7

**Suriname**	11·1	22·3	35·1	19·7	11·8
**Trinidad and Tobago**	11	22·5	33·1	22·3	11
**Virgin Islands**	10	21·3	36·6	21·4	10·7
**Tropical Latin America**	15·9	26·2	22·1	22·6	13·2
**Brazil**	15·9	26·2	22	22·6	13·2
**Paraguay**	16·1	23·3	26·8	20·5	13·3
**East Asia**	48·8	22·4	18·1	6·9	3·8
**China**	48·9	22·2	18·1	7	3·8
**North Korea**	50	23·6	16·8	5·2	4·4
**Taiwan (province of China)**	45	25·6	18·3	7·2	4
**Southeast Asia**	33	34·8	14·9	9·9	7·3
**Cambodia**	34·9	29·6	16·6	10·4	8·5
**Indonesia**	28·6	41·9	11·9	10·1	7·5
**Laos**	37·7	27·7	18	8·8	7·8
**Malaysia**	43·7	19·3	18	12·8	6·3
**Maldives**	34·9	28·7	16·6	12·4	7·4
**Mauritius**	34·5	29·1	19	11·4	6
**Myanmar**	38·1	30·4	16	8·4	7·2
**Philippines**	36·2	28·8	18·7	9·1	7·1
**Sri Lanka**	36	30·4	17·7	9·1	6·7
**Seychelles**	34·2	29	17·5	13·4	5·9
**Thailand**	34·2	27·3	17·6	13·8	7·1
**East Timor**	37·8	29·1	15·5	9	8·7
**Vietnam**	38	28·3	18·7	8	6·9
**Oceania**	43·9	24	16·1	7·5	8·6
**American Samoa**	40·9	21·5	14·5	16·5	6·6
**Federated States of Micronesia**	41·3	23·9	15·4	11·3	8·1
**Fiji**	40·3	24·6	15·8	10·9	8·5
**Guam**	42·7	22·5	17	11·7	6
**Kiribati**	42·8	22·9	14·2	11·1	9
**Marshall Islands**	42·7	23·1	15·5	11·2	7·6
**Northern Mariana Islands**	43·2	23·2	16·4	11·5	5·8
**Papua New Guinea**	44·2	24·1	16·2	6·7	8·8
**Samoa**	41·6	22·5	15·2	12·8	7·9
**Solomon Islands**	43·3	24·1	15·7	9	7·8

**Tonga**	39·9	22·7	14·9	14	8·6
**Vanuatu**	45	23·8	14·9	8·3	8
**North Africa and Middle East**	38·1	35·4	5·3	12·9	8·2
**Afghanistan**	32·5	35·2	5·3	8·9	18
**Algeria**	31·8	39·3	6·2	12·8	9·9
**Bahrain**	31	40·4	7·3	15·3	6·1
**Egypt**	41·5	34·4	4·8	12·8	6·5
**Iran**	28·7	43·3	5·4	13·8	8·7
**Iraq**	31	36·4	5·8	12	14·7
**Jordan**	30·2	38·1	6·1	15·4	10·1
**Kuwait**	30·3	39·5	6·7	17·8	5·9
**Lebanon**	34·4	36·5	5·9	13·4	9·7
**Libya**	30	37·7	6	16·5	9·9
**Morocco**	31·7	39·4	6	13	10
**Palestine**	31·2	38·2	5·9	13	11·6
**Oman**	30·7	39·5	6·6	15·2	7·9
**Qatar**	30·7	39·3	7·1	16·8	6·1
**Saudi Arabia**	22·3	48·6	6·4	16·3	6·4
**Sudan**	33	36	6·3	10·6	14·1
**Syria**	31·7	38·6	6·1	13·4	10·3
**Tunisia**	12	55·7	7·2	14·9	10·1
**Turkey**	54·7	16·2	6·7	14·1	8·4
**United Arab Emirates**	31·7	41·7	7·9	14·9	3·8
**Yemen**	31·3	38·7	6·1	9·4	14·5
**South Asia**	32·6	20·8	29·4	5·4	11·8
**Bangladesh**	28·2	24·5	27·7	5·6	14
**Bhutan**	29	23·2	30·1	5·9	11·9
**India**	34·7	16·7	33·1	5·2	10·4
**Nepal**	22·6	15	46·7	5	10·7
**Pakistan**	26·4	39·5	10·4	6·3	17·4
**Southern sub-Saharan Africa**	21·9	33·9	19·7	9·5	15
**Botswana**	21·8	35·6	19·3	8·5	14·8
**eSwatini**	22·9	36·1	18·2	8·6	14·1
**Lesotho**	22·8	35·7	19·4	7·9	14·2
**Namibia**	23·9	34·7	21	6·6	13·9

**South Africa**	21·1	33·4	20·5	10·6	14·5
**Zimbabwe**	24·1	34·7	17·2	6·8	17·1
**Western sub-Saharan Africa**	48·9	7·8	19·9	6·2	17·1
**Benin**	49·9	7·8	16·9	7	18·4
**Burkina Faso**	49·5	7·6	17·5	6	19·4
**Cameroon**	49·3	8·2	18·6	7·9	16
**Cape Verde**	49·6	8·8	19·3	7·6	14·7
**Chad**	54·8	7·4	16	5	16·8
**Côte d'Ivoire**	51·6	8·1	18·5	6·2	15·6
**The Gambia**	51·5	8	17·2	6·6	16·7
**Ghana**	48·4	8·7	18·3	7·5	17·1
**Guinea**	54	7·7	15·6	5·7	17
**Guinea-Bissau**	53·9	7·5	16·1	6·6	15·9
**Liberia**	53·1	7·3	16·1	6·7	16·8
**Mali**	51·7	7·4	13·1	5·7	22
**Mauritania**	52	8·3	14·6	8·2	16·9
**Niger**	53·8	7·1	13·8	5·1	20·3
**Nigeria**	47·5	7·8	21·8	6	17
**São Tomé and Príncipe**	50·4	8·2	18·6	6·9	15·9
**Senegal**	54·6	7·9	14·2	6·4	16·8
**Sierra Leone**	51·2	7·8	18·2	6·2	16·7
**Togo**	54·7	7·9	15·7	6·2	15·4
**Eastern sub-Saharan Africa**	25·9	28·9	20·3	7	17·9
**Burundi**	23	30·8	23·2	6·4	16·5
**Comoros**	24·6	32·2	17·3	7·6	18·3
**Djibouti**	25·7	30·5	20	8·3	15·5
**Eritrea**	24·9	31·7	19·7	6·3	17·5
**Ethiopia**	29·3	33·9	16·2	5·6	15
**Kenya**	27·7	13·5	27·5	10	21·2
**Madagascar**	24·9	31·6	19·4	6·5	17·6
**Malawi**	25·4	30·5	18·2	6·9	19
**Mozambique**	25·8	27·4	18·4	6·6	21·7
**Rwanda**	14·1	47·1	18·9	5·5	14·4
**Somalia**	26·2	30·7	19	6·8	17·3
**South Sudan**	27·3	29·3	19·5	7·3	16·6

**Tanzania**	22·9	29·6	20·1	7·3	20·2
**Uganda**	22·9	29·5	23	6·6	18·1
**Zambia**	25	30·7	19·5	6·9	17·9
**Central sub-Saharan Africa**	31·2	32·4	16·8	5·7	14
**Angola**	29·9	32·1	17·3	6·7	14·1
**Central African Republic**	32·4	32·4	16·6	5·3	13·4
**Congo**	30·3	33	17·3	6·4	13·1
**DR Congo**	31·8	32·4	16·5	5·1	14·2
**Equatorial Guinea**	29·2	30·8	17·4	8·9	13·7
**Gabon**	28·1	33·7	18·4	7·4	12·4

NASH=non-alcoholic steatohepatitis.

Appendix Table 8: Proportion of causes for prevalent cases of decompensated cirrhosis in 2017 in both sexes

	Hepatitis B	Hepatitis C	Alcohol	NASH	Other causes
**Global**	27·9	24·8	23·1	8·6	15·5
**High-income North America**	5·1	33	26·9	10·2	24·8
**Canada**	5·4	30·5	30·9	9·3	23·9
**Greenland**	5·7	32·3	30·5	8·4	23
**USA**	5·1	33·2	26·5	10·3	24·9
**Australasia**	23	28·3	24·9	12·9	10·8
**Australia**	23	28·5	25	12·8	10·7
**New Zealand**	23·1	27·6	24·1	13·3	11·8
**High-income Asia Pacific**	20	43·6	19·7	4·3	12·4
**Brunei**	32·2	21·8	22·7	4·2	19·1
**Japan**	14·9	55·6	14	3·8	11·8
**Singapore**	50	13	16·4	2·2	18·4
**South Korea**	33·6	9·4	37·2	6·2	13·6
**Western Europe**	8·1	23·6	43·1	8·8	16·3
**Andorra**	8	18·6	49·3	9·1	15·1
**Austria**	7	17·6	50·5	8·9	16·1
**Belgium**	8·3	22·4	49·4	5·6	14·4
**Cyprus**	8·2	18·9	46	10·2	16·7
**Denmark**	8·2	16·6	50	8·6	16·6
**Finland**	8·9	18	45·2	10·7	17·2
**France**	7·7	17·1	49·3	8·6	17·2
**Germany**	7·3	17	49·8	9·7	16·1
**Greece**	10·2	17	45·8	10·3	16·6
**Iceland**	7·3	8·8	37·4	22·3	24·2
**Ireland**	8·7	17·4	47	9·7	17·2
**Israel**	9	20·7	36·5	11·6	22·2
**Italy**	10·6	50·3	19·8	6·2	13·2
**Luxembourg**	7·2	16·8	51·2	9·1	15·7
**Malta**	8·3	18·7	45·2	11·8	16
**Netherlands**	8·7	17·5	46·3	10·2	17·3
**Norway**	8·7	17·8	44·6	9·7	19·1
**Portugal**	8	18·5	47·4	10·2	15·9

**Spain**	7·1	25·7	50·8	8·9	7·4
**Sweden**	8·3	15·7	45·1	10·4	20·6
**Switzerland**	8·9	17·9	48·1	8·9	16·2
**UK**	6·3	8·7	46·7	10·4	27·9
**Southern Latin America**	12	26·6	33·4	9·1	18·8
**Argentina**	11·9	26·2	34	8·6	19·4
**Chile**	12·1	27·3	33·1	9·9	17·6
**Uruguay**	12·6	28	29·4	10·3	19·7
**Eastern Europe**	16·6	30·1	33	9·4	10·9
**Belarus**	15·7	28·9	35·8	8·9	10·6
**Estonia**	15·2	27·9	37·6	8·9	10·4
**Latvia**	15·8	29	35·3	9·4	10·5
**Lithuania**	15·6	28·8	36·3	8·9	10·3
**Moldova**	17·2	30·9	32·4	9·4	10·1
**Russia**	16·5	30·3	32·4	9·5	11·2
**Ukraine**	17·2	30	33·6	9·2	10
**Central Europe**	20·8	15·8	46	8·1	9·2
**Albania**	23·4	18	39	8·5	11
**Bosnia and Herzegovina**	24	18·2	38·5	9·3	10
**Bulgaria**	21	15·9	46·4	7·9	8·7
**Croatia**	21·3	15·8	45·5	8·4	8·9
**Czech Republic**	19·8	15·4	48	7·7	9
**Hungary**	23	15	45·4	8	8·6
**Montenegro**	22	16·6	42·7	8·2	10·5
**North Macedonia**	23·2	17·5	39·7	9·6	10
**Poland**	20	15·5	47	7·9	9·6
**Romania**	20·6	16	46·4	8·3	8·8
**Serbia**	21·5	16·8	43·6	8·6	9·6
**Slovakia**	20·2	15·6	47·1	7·8	9·3
**Slovenia**	20·9	16·2	45·3	9	8·6
**Central Asia**	19·3	22·3	33·6	7·9	16·9
**Armenia**	19·8	24	34·5	8·8	12·9
**Azerbaijan**	20·4	22·9	33·5	8·7	14·4
**Georgia**	19·6	23	36·6	8·8	12
**Kazakhstan**	18·1	22	36·5	7·9	15·5

**Kyrgyzstan**	19·7	22·6	31·3	7·3	19·2
**Mongolia**	19·7	22·8	33	7·1	17·4
**Tajikistan**	18·9	21·3	30·3	6·6	23
**Turkmenistan**	19·4	22·6	32·9	8·1	17
**Uzbekistan**	19·5	22	32·6	7·9	18
**Central Latin America**	5·3	30·4	38·1	14·4	11·8
**Colombia**	5·6	28·3	42	13	11·1
**Costa Rica**	5·1	28·5	42·1	14·3	10
**El Salvador**	5·3	29·8	37·7	15·4	11·8
**Guatemala**	5·9	32·4	33·5	14·2	14
**Honduras**	5·8	31·9	33·8	14·4	14·1
**Mexico**	5	31·2	37·1	14·6	12·1
**Nicaragua**	5·3	30·4	35·7	16·3	12·2
**Panama**	5·7	30·7	39·7	12·5	11·4
**Venezuela**	5·6	29·5	38·6	15·6	10·7
**Andean Latin America**	15·1	16·7	29·8	18·1	20·3
**Bolivia**	14·9	17	27·6	17·7	22·7
**Ecuador**	15·3	17·6	25·4	20·8	20·8
**Peru**	15	16·1	32·6	17	19·3
**Caribbean**	10·5	24·8	29·8	18·1	16·7
**Antigua and Barbuda**	10	24·6	29·3	20·4	15·7
**The Bahamas**	10	24·6	31·3	17·6	16·5
**Barbados**	10	24·4	29·6	21·1	14·9
**Belize**	10	23·8	28·6	17·9	19·8
**Bermuda**	10·6	22·4	31·8	22	13·3
**Cuba**	10·3	25·4	30·2	19·7	14·4
**Dominica**	10·4	24·2	30·9	18·5	16
**Dominican Republic**	10·5	24·4	31·1	16·2	17·8
**Grenada**	10·2	24·3	30·4	18·2	16·8
**Guyana**	10·1	24·7	29·2	17·4	18·6
**Haiti**	12	24·6	27·6	13	22·8
**Jamaica**	10·4	25·3	28·1	18·8	17·4
**Puerto Rico**	9·9	24	29·8	21·3	15·1
**Saint Lucia**	10	24·1	30·5	19·8	15·5
**Saint Vincent and the Grenadines**	9·6	23	28·3	23·6	15·5

**Suriname**	10·7	24·4	30·1	18	16·8
**Trinidad and Tobago**	10·7	24·7	28·4	20·9	15·3
**Virgin Islands**	9·7	23·5	31·6	20·6	14·6
**Tropical Latin America**	15	28·4	19·6	20·3	16·8
**Brazil**	15	28·5	19·5	20·3	16·7
**Paraguay**	14·9	25·8	22·8	17·8	18·5
**East Asia**	51·6	18·3	16·5	7·2	6·4
**China**	51·7	18·1	16·5	7·3	6·4
**North Korea**	52·1	20·7	14·5	5·3	7·4
**Taiwan (province of China)**	45·1	26·8	15·6	7·3	5·2
**Southeast Asia**	31·9	31·8	13·7	11·4	11·1
**Cambodia**	32·8	29	14·3	9·9	14
**Indonesia**	27	41·5	10·2	10·2	11
**Laos**	34·6	26·5	14·8	9	15
**Malaysia**	43·2	20·1	14	12	10·6
**Maldives**	34·4	29·3	14·1	12·1	10·2
**Mauritius**	31·8	28·5	16·4	14·8	8·6
**Myanmar**	34	29·5	13·2	11·2	12
**Philippines**	33·1	27·6	15·3	10·2	13·9
**Sri Lanka**	32·8	29·8	15·1	12·2	10
**Seychelles**	31·5	28·2	14·9	16·3	9·1
**Thailand**	31·5	26·6	16·4	16·9	8·6
**East Timor**	34·2	27·4	12·3	8·9	17·1
**Vietnam**	35	28	15·9	9·9	11·2
**Oceania**	39·2	20·9	11·8	8	20·2
**American Samoa**	38·5	19·2	11·4	16·9	14
**Federated States of Micronesia**	38·9	21·4	12·1	11·1	16·5
**Fiji**	39·1	22·8	13	10·9	14·2
**Guam**	41·5	21·1	14·3	12·5	10·5
**Kiribati**	38·9	19·7	10·6	10·3	20·4
**Marshall Islands**	39·3	20·5	11·8	11·1	17·4
**Northern Mariana Islands**	42·8	22·3	14·6	12·4	8
**Papua New Guinea**	39·2	20·7	11·6	6·9	21·7
**Samoa**	38·6	20	11·7	12·3	17·4
**Solomon Islands**	38·4	20·2	11·1	8·5	21·7

**Tonga**	37·3	20·9	11·8	14·1	15·9
**Vanuatu**	40·2	20·3	10·5	8	21
**North Africa and Middle East**	27·3	36·5	3·8	13·7	18·7
**Afghanistan**	23·4	29·0	2·9	7·8	37
**Algeria**	25·9	38·7	4·4	14·4	16·6
**Bahrain**	26·6	39·8	5·1	16·7	11·8
**Egypt**	23	42·1	3	13·1	18·8
**Iran**	27·9	42·5	2·9	12·1	14·6
**Iraq**	24·3	33·2	3·5	12·3	26·6
**Jordan**	23·1	34·7	3·7	15·4	23
**Kuwait**	25·2	37·9	4·3	19·5	13·1
**Lebanon**	27·5	35·5	4·1	14·8	18·1
**Libya**	24·1	36·6	4·1	18	17·2
**Morocco**	25·6	38·7	4·3	14·6	16·8
**Palestine**	23·8	34·3	3·6	12·5	25·8
**Oman**	26·9	38·2	4·3	15·4	15·1
**Qatar**	28·1	39·2	4·8	17·5	10·5
**Saudi Arabia**	15·3	52·3	3·8	15	13·5
**Sudan**	24·2	32·7	3·8	10·8	28·6
**Syria**	24·4	36·4	4	14·7	20·5
**Tunisia**	20·8	31·4	7·1	23	17·7
**Turkey**	46·7	19·7	4·5	15·8	13·3
**United Arab Emirates**	29·5	40·9	5·4	15·5	8·7
**Yemen**	23·3	33·9	3·5	8·9	30·4
**South Asia**	28	17·9	23·8	5·2	25·1
**Bangladesh**	26	21·2	21·4	5·9	25·6
**Bhutan**	26·1	20·4	22·5	6·3	24·6
**India**	29·2	15·2	26·3	5·1	24·3
**Nepal**	18·8	10·2	38·1	5·1	27·8
**Pakistan**	22	36·9	5·9	5·3	29·9
**Southern sub-Saharan Africa**	17·3	20	15·3	9·7	37·7
**Botswana**	17·8	21·6	15·6	8·8	36·3
**eSwatini**	16·5	19·4	12·3	8·2	43·6
**Lesotho**	17·3	20·1	14·1	7·7	40·7
**Namibia**	18·6	20	15·6	6·6	39·1

**South Africa**	17·3	20·5	16·2	10·9	35·2
**Zimbabwe**	17	18	12	5·9	47·1
**Western sub-Saharan Africa**	34·5	9·9	12·2	5·4	38
**Benin**	34·1	9·6	10·8	5·8	39·7
**Burkina Faso**	33·8	9·4	11·5	4·9	40·4
**Cameroon**	33·3	9·9	11·7	6·4	38·6
**Cape Verde**	38·3	12·2	14·1	7·8	27·5
**Chad**	35·1	8·5	9·1	3·9	43·4
**Côte d'Ivoire**	35·4	10·2	11·5	5·4	37·5
**The Gambia**	35·2	9·8	10·9	5·5	38·7
**Ghana**	34·9	11·4	13	6·5	34
**Guinea**	37·2	9·6	9·9	4·9	38·4
**Guinea-Bissau**	35·8	8·8	9·7	5·3	40·4
**Liberia**	38	9·4	10·9	5·8	35·9
**Mali**	36·6	9·4	8·5	4·8	40·7
**Mauritania**	36·7	10·7	9·7	7·3	35·7
**Niger**	35·8	8·3	8·3	4	43·6
**Nigeria**	33·1	10	14·2	5·4	37·2
**São Tomé and Príncipe**	36·8	10·9	12·9	6·3	33·1
**Senegal**	38·8	10·1	9·2	5·6	36·2
**Sierra Leone**	35·9	9·8	12·1	5·1	37·1
**Togo**	38·9	10·1	10·2	5·3	35·6
**Eastern sub-Saharan Africa**	18·3	25·7	12·2	5·4	38·5
**Burundi**	15·3	26·3	14·6	4·9	38·9
**Comoros**	18·7	31·3	12·7	6·7	30·5
**Djibouti**	19·5	29·8	14·4	7·4	28·9
**Eritrea**	16·3	27·9	12·1	5·2	38·6
**Ethiopia**	20·5	27·5	9·6	4·3	38·1
**Kenya**	24·8	17·2	14	6·7	37·2
**Madagascar**	17	28	12·5	5·2	37·3
**Malawi**	16·7	26·6	11·3	5·8	39·6
**Mozambique**	17·1	24·2	12·2	5·3	41·2
**Rwanda**	8·7	36·7	14·7	5·2	34·7
**Somalia**	17·5	26·3	11·6	5·3	39·4
**South Sudan**	18·1	25·4	11·9	5·9	38·7

**Tanzania**	16·1	26·9	13·6	6·1	37·3
**Uganda**	14·6	24·2	13·7	5	42·5
**Zambia**	16·2	26·4	11·9	5·6	40
**Central sub-Saharan Africa**	26·8	22·2	10·6	4·7	35·8
**Angola**	24·8	21·5	10·7	5·5	37·5
**Central African Republic**	28·1	22·1	10·5	4·3	34·9
**Congo**	27·7	24·2	12·1	5·6	30·5
**DR Congo**	27·3	22·2	10·4	4·3	35·7
**Equatorial Guinea**	24·4	20·5	10·9	7·4	36·9
**Gabon**	26·1	25·4	13	7	28·4

NASH=non-alcoholic steatohepatitis.

Appendix Table 9: Proportion of causes for prevalent cases of compensated cirrhosis in 2017 in both sexes

	Hepatitis B	Hepatitis C	Alcohol	NASH	Other causes
**Global**	32·6	24·7	21	8·4	13·3
**High-income North America**	5·1	33·7	27·1	10·5	23·6
**Canada**	5·3	31·1	30·7	9·6	23·2
**Greenland**	5·7	32·9	30	8·9	22·5
**USA**	5·1	33·9	26·7	10·6	23·7
**Australasia**	22·8	28·8	24·1	12·6	11·7
**Australia**	22·9	29	24·3	12·5	11·2
**New Zealand**	22·5	27·6	23·2	12·8	13·8
**High-income Asia Pacific**	21·1	40·5	19·5	4·3	14·5
**Brunei**	31·4	20·9	18·9	4	24·7
**Japan**	14·7	54·9	13·3	3·6	13·4
**Singapore**	47·8	12·3	14	2·1	23·9
**South Korea**	33·9	9·5	34·2	6	16·3
**Western Europe**	8·5	24·1	42·1	8·7	16·5
**Andorra**	8·4	19·1	48·4	8·9	15·1
**Austria**	7·5	18·2	49·9	8·8	15·7
**Belgium**	8·7	23·2	48·2	5·5	14·3
**Cyprus**	8·8	19·5	45·2	9·8	16·7
**Denmark**	8·7	17·1	49	8·4	16·7
**Finland**	9·5	18·5	44·4	10·3	17·2
**France**	8·2	17·7	48·6	8·4	17·1
**Germany**	7·8	17·6	49·1	9·7	15·8
**Greece**	10·7	17·5	45·1	10·1	16·6
**Iceland**	8	8·8	36·5	21·4	25·4
**Ireland**	9·2	17·9	45·6	9·5	17·7
**Israel**	9·5	21·3	35·3	11·4	22·5
**Italy**	10·9	51·1	19·3	6·1	12·6
**Luxembourg**	7·8	17·5	50·4	9·1	15·2
**Malta**	8·8	19·2	43·9	11·5	16·6
**Netherlands**	9·1	17·9	44·9	10·1	18
**Norway**	9·1	17·9	42·6	9·3	21·1
**Portugal**	8·5	19·1	47·2	9·9	15·2
**Spain**	7·5	26·5	50	8·8	7·2

**Sweden**	8·8	15·6	42·1	10·3	23·2
**Switzerland**	9·4	18·4	46·7	8·8	16·6
**UK**	6·6	8·5	45·5	10·2	29·2
**Southern Latin America**	12·6	27·9	33·9	9·3	16·2
**Argentina**	12·6	27·5	34·5	8·7	16·7
**Chile**	12·7	28·6	33·4	10·2	15·2
**Uruguay**	13·1	29·2	29·6	10·4	17·6
**Eastern Europe**	17·2	30	31·4	9·1	12·2
**Belarus**	16·3	28·8	34·3	8·6	12
**Estonia**	15·7	27·7	35·9	8·5	12·1
**Latvia**	16·3	28·8	33·7	9	12·1
**Lithuania**	16·2	28·6	34·7	8·6	11·9
**Moldova**	17·8	31·3	30·5	9·3	11
**Russia**	17·1	30·2	31	9·2	12.5
**Ukraine**	17·8	29·8	31·8	8·9	11·7
**Central Europe**	21·5	15·9	43·6	7·8	11·2
**Albania**	23·6	17·6	36	8·1	14·7
**Bosnia and Herzegovina**	24·3	18	36·2	9	12·6
**Bulgaria**	21·8	16	44·1	7·7	10·4
**Croatia**	22·2	15·9	43·2	8·1	10·6
**Czech Republic**	20·3	15·5	45·6	7·6	11·1
**Hungary**	23·9	15·1	42·9	7·8	10·3
**Montenegro**	22·1	16·2	39·6	7·9	14·1
**North Macedonia**	23·7	17·2	37	9·1	12·9
**Poland**	20·7	15·5	44·4	7·6	11·7
**Romania**	21·4	16·3	44·2	8	10
**Serbia**	21·9	16·7	40·9	8·3	12·3
**Slovakia**	21	15·6	44·6	7·6	11·2
**Slovenia**	21·6	16·3	43	8·7	10·3
**Central Asia**	20·1	22·6	32·2	7·8	17·3
**Armenia**	20·3	24	32·6	8·4	14·7
**Azerbaijan**	21	23·1	31·9	8·5	15·5
**Georgia**	20·4	23·2	34·8	8·4	13·3
**Kazakhstan**	18·9	22·4	35·3	7·7	15·6
**Kyrgyzstan**	20·7	23·1	30·3	7·2	18·7

**Mongolia**	20·6	23·6	32·7	7·2	15·8
**Tajikistan**	19·6	21·5	28·8	6·5	23·6
**Turkmenistan**	20·5	23	31·8	7·9	16·8
**Uzbekistan**	20·3	22·4	31·5	7·8	18
**Central Latin America**	5·5	32·2	38·1	14·2	10
**Colombia**	5·8	29·5	41·5	12·7	10·5
**Costa Rica**	5·4	29·9	42	13·9	8·9
**El Salvador**	5·6	31·6	38·1	15	9·8
**Guatemala**	6·3	34·7	34	13·9	11
**Honduras**	6·1	34·1	34·3	14·4	11·1
**Mexico**	5·2	32·8	37·8	14·4	9·9
**Nicaragua**	5·6	32·2	35·9	16	10·3
**Panama**	5·9	32	39·4	12·2	10·5
**Venezuela**	5·9	31	38·5	15·2	9·5
**Andean Latin America**	15·5	17·6	29·6	18	19·3
**Bolivia**	15·6	18·1	27·9	17·8	20·6
**Ecuador**	15·8	18·6	25·5	20·7	19·5
**Peru**	15·4	16·9	32·3	16·6	18·8
**Caribbean**	11·3	26·3	30·1	17·2	15·1
**Antigua and Barbuda**	10·5	25·8	29·3	20	14·3
**The Bahamas**	10·7	26	31·6	17·3	14·4
**Barbados**	10·4	25·5	29·7	20·5	13·9
**Belize**	10·9	25·6	29·2	17·8	16·6
**Bermuda**	11	23·3	32	21·3	12·4
**Cuba**	10·7	26·4	30·3	19·2	13·4
**Dominica**	10·9	25·3	30·8	18	15
**Dominican Republic**	11·2	26	31·5	15·8	15·4
**Grenada**	10·7	25·7	30·6	17·7	15·4
**Guyana**	11	26·5	30·3	17·2	14·9
**Haiti**	13·2	27	28·8	13·2	17·8
**Jamaica**	10·9	26·4	27·7	18·2	16·7
**Puerto Rico**	10·3	25·2	30·5	20·6	13·4
**Saint Lucia**	10·4	25·4	30·7	19·5	14
**Saint Vincent and the Grenadines**	10·2	24·4	28·7	22·9	13·9
**Suriname**	11·4	26	30·9	17·6	14·1

**Trinidad and Tobago**	11·3	26	28·5	20·3	13·9
**Virgin Islands**	10·1	24·6	32·3	20·1	12·9
**Tropical Latin America**	15·7	30	20	20	14·4
**Brazil**	15·7	30	19·9	20	14·3
**Paraguay**	15·9	27·8	23	17·9	15·3
**East Asia**	53·1	18·9	16·2	7	4·8
**China**	53·3	18·6	16·3	7	4·8
**North Korea**	53·6	21·4	14·4	5·1	5·4
**Taiwan (province of China)**	45·9	27·6	15·3	6·9	4·4
**Southeast Asia**	33·1	33·6	13·8	10·8	8·8
**Cambodia**	34·9	30·8	14·8	9·7	9·9
**Indonesia**	27·8	42·9	10·3	10·2	8·8
**Laos**	37	28·2	15·3	8·9	10·7
**Malaysia**	44·5	20·7	13·8	11·9	9·1
**Maldives**	35·2	30	13·6	12	9·2
**Mauritius**	32·8	29·4	16·5	13·9	7·5
**Myanmar**	36·1	31	13·7	10·4	8·8
**Philippines**	35·3	29·1	15·7	9·5	10·4
**Sri Lanka**	34	30·8	15·2	11·4	8·6
**Seychelles**	33	29·4	15·3	15·3	7
**Thailand**	32·4	27·6	16·2	16·1	7·6
**East Timor**	36·1	29·1	12·6	8·8	13·4
**Vietnam**	36·6	29·2	16·2	9·4	8·6
**Oceania**	43·6	22·9	12·3	8	13·2
**American Samoa**	40·4	20·1	11·3	16·8	11·4
**Federated States of Micronesia**	41·7	22·8	12·2	11·2	12·1
**Fiji**	41	23·8	12·8	10·8	11·5
**Guam**	43·2	21·8	14	12·2	8·8
**Kiribati**	43·4	21·8	11·2	10·7	12·8
**Marshall Islands**	42·8	22·1	12·2	11·3	11·5
**Northern Mariana Islands**	43·5	22·6	14·1	12·1	7·6
**Papua New Guinea**	44	23	12·4	7·2	13·5
**Samoa**	41·4	21·3	11·9	12·4	13·1
**Solomon Islands**	42·6	22·4	11·7	8·9	14·4
**Tonga**	39·7	22·2	11·9	14·1	12·1

**Vanuatu**	44·6	22·4	11·1	8·3	13·6
**North Africa and Middle East**	28	39·2	3·7	13·4	15·7
**Afghanistan**	27·8	34·3	3·2	8·8	25·9
**Algeria**	27·1	40·1	4·2	13·7	14·9
**Bahrain**	27·8	40·6	4·9	16·4	10·2
**Egypt**	24·9	44·9	3·1	13·4	13·7
**Iran**	29·1	43·5	2·8	12·1	12·5
**Iraq**	26·1	35·4	3·4	12·3	22·7
**Jordan**	24·2	36·4	3·5	15·5	20·4
**Kuwait**	26·4	39	4	18·9	11·7
**Lebanon**	29	36·8	3·9	14·1	16·3
**Libya**	25·5	38·4	4	17·7	14·5
**Morocco**	26·6	40	4	14·1	15·2
**Palestine**	25·5	36·4	3·5	12·5	22·2
**Oman**	29	39·9	4·2	14·9	12
**Qatar**	29·7	40·1	4·5	17·1	8·6
**Saudi Arabia**	16·1	54·1	3·7	15·1	11
**Sudan**	26·8	35·9	3·8	11	22·4
**Syria**	25·4	37·6	3·9	14·2	18·8
**Tunisia**	20·6	32·5	6·8	22·4	17·6
**Turkey**	48·1	20	4·4	14·6	12·9
**United Arab Emirates**	30·8	41·6	5·2	15·3	7
**Yemen**	26·2	37·5	3·6	9·1	23·6
**South Asia**	30·4	19·9	24·8	5·2	19·7
**Bangladesh**	27·8	23	22·1	5·9	21·2
**Bhutan**	28·6	22·3	23·8	6·4	18·9
**India**	31·8	16·4	27·6	5·1	19·1
**Nepal**	21·1	11·3	41·2	5·3	21·1
**Pakistan**	24·5	41·2	6·4	5·6	22·4
**Southern sub-Saharan Africa**	19·8	22·8	16·9	10·2	30·3
**Botswana**	20·2	24·6	17·2	9·5	28·5
**eSwatini**	20	23·4	14·6	9·4	32·5
**Lesotho**	20·6	23·7	16·3	8·6	30·8
**Namibia**	21·4	23	17·6	7·2	30·8
**South Africa**	19·2	22·8	17·5	11·4	29·1

**Zimbabwe**	21·4	22·7	14·8	7	34·1
**Western sub-Saharan Africa**	39·3	11·2	13·6	5·9	30
**Benin**	39·2	11	12·1	6·5	31·2
**Burkina Faso**	39·7	11	13·2	5·5	30·6
**Cameroon**	38·4	11·5	13·2	7·2	29·7
**Cape Verde**	40·2	12·8	14·5	7·7	24·7
**Chad**	41·7	10·1	10·6	4·6	33
**Côte d'Ivoire**	41	11·8	13·1	6	28·2
**The Gambia**	40	11·1	12·1	6·1	30·8
**Ghana**	38·7	12·6	14	7	27·6
**Guinea**	42·4	10·9	11	5·4	30·4
**Guinea-Bissau**	42·6	10·5	11·3	6·1	29·5
**Liberia**	43·2	10·7	12·1	6·4	27·5
**Mali**	42·2	10·8	9·5	5·4	32·2
**Mauritania**	40·3	11·7	10·3	7·8	29·8
**Niger**	42·6	9·9	9·6	4·6	33·3
**Nigeria**	37·3	11·3	15·7	5·9	29·8
**São Tomé and Príncipe**	40·8	12·1	14	6·7	26·4
**Senegal**	43·2	11·3	10	6·1	29·4
**Sierra Leone**	40·7	11·1	13·3	5·6	29·3
**Togo**	44	11·4	11·3	5·8	27·5
**Eastern sub-Saharan Africa**	21·2	29·3	13·7	5·8	30
**Burundi**	18·2	30·6	16·4	5·4	29·4
**Comoros**	20·5	33·5	13·2	6·8	26
**Djibouti**	21·5	32·2	14·8	7·7	23·8
**Eritrea**	19·5	32·2	13·8	5·6	29
**Ethiopia**	23·6	31·3	10·9	4·7	29·3
**Kenya**	27·7	19	15·6	7·2	30·5
**Madagascar**	19·4	31·4	13·5	5·6	30·2
**Malawi**	19·9	30·9	12·8	6·2	30·3
**Mozambique**	20·4	28·1	13·8	5·8	31·9
**Rwanda**	9·8	41·2	16·4	5·6	26·9
**Somalia**	20·8	30·6	13·1	5·8	29·7
**South Sudan**	21·3	29·3	13·3	6·5	29·5
**Tanzania**	18·5	30·4	14·8	6·5	29·8

**Uganda**	17·7	28·7	15·8	5·5	32·2
**Zambia**	19·5	30·9	13·6	6·1	29·9
**Central sub-Saharan Africa**	30·7	25·2	11·8	5·1	27·2
**Angola**	28·9	24·9	12·2	6·1	28
**Central African Republic**	32·3	25·2	11·8	4·7	26
**Congo**	30·8	26·8	13·1	6	23·3
**DR Congo**	31·3	25·2	11·6	4·7	27·4
**Equatorial Guinea**	28·1	23·4	12·2	8	28·2
**Gabon**	28·4	27·5	13·8	7·2	23

Appendix Table 10: Proportion of causes for DALYs due to cirrhosis in 2017 in both sexes DALYs=disability-adjusted life years.

	Hepatitis B	Hepatitis C	Alcohol	NASH	Other causes
**Global**	29·5	25·1	24·6	8·3	12·5
**High-income North America**	5·8	36·1	29·2	10·8	18·1
**Canada**	5·9	32·5	33·6	9·6	18·5
**Greenland**	6·1	34·8	32·2	9·6	17·4
**USA**	5·7	36·4	28·9	10·9	18·1
**Australasia**	17	36	29·1	11·9	6·1
**Australia**	16·9	36·1	29·2	11·8	6
**New Zealand**	17·6	35·3	27·9	12·5	6·7
**High-income Asia Pacific**	22·5	39	25·1	4·7	8·7
**Brunei**	34·6	23·1	25·5	4·8	12
**Japan**	15·6	55·4	16	4·4	8·7
**Singapore**	53·5	10·9	17·3	2·6	15·6
**South Korea**	34·7	9·1	42·4	5·2	8·6
**Western Europe**	8·2	21·3	44·9	9·6	16
**Andorra**	8·1	19·2	48·1	9·2	15·3
**Austria**	7·2	18·3	49·6	9·2	15·6
**Belgium**	9·5	15·8	55·7	7·4	11·5
**Cyprus**	8·4	18·7	44·9	10	18
**Denmark**	8·4	17·6	49·2	9·3	15·4
**Finland**	9·1	18·8	46·5	10·8	14·9
**France**	8	18	48·7	9	16·3
**Germany**	7·5	17·7	48·2	10·2	16·4
**Greece**	10·3	17·2	45	10·2	17·3
**Iceland**	7·7	8·3	41	18·7	24·3
**Ireland**	8·9	18·2	46·3	10·3	16·3
**Israel**	8·9	20·4	36	11·8	22·9
**Italy**	10·9	50·6	20·2	6	12·3
**Luxembourg**	7·5	17·7	49·2	9·8	15·8
**Malta**	8·5	18·8	43·3	12·1	17·4
**Netherlands**	8·6	17·7	44·2	10·7	18·8
**Norway**	8·9	18·3	45·2	9·8	17·9
**Portugal**	8·1	19	46·6	10·3	15·9

**Spain**	7·6	20·7	54·1	11·1	6·5
**Sweden**	8·8	16·9	44·7	11	18·6
**Switzerland**	8·9	18·3	46·4	9·3	17·2
**UK**	6·6	9·3	48·2	11·1	24·9
**Southern Latin America**	12·8	29·5	35·9	9·2	12·6
**Argentina**	12·7	29	36·8	8·6	13
**Chile**	12·9	30·3	35·1	10	11·7
**Uruguay**	13·4	30·7	31·4	10·1	14·5
**Eastern Europe**	18·7	24·6	37·3	10·7	8·7
**Belarus**	17·1	23·4	40·7	10·3	8·4
**Estonia**	16·4	22·6	43·3	10	7·6
**Latvia**	17·1	23·6	40·4	10·8	8·1
**Lithuania**	17·1	23·3	42	10·2	7·4
**Moldova**	18·2	25	36·1	11·6	9·1
**Russia**	18·7	24·8	36·7	10·8	9
**Ukraine**	19·3	24·1	38·2	10·2	8·3
**Central Europe**	22·6	20·5	43·9	7·3	5·8
**Albania**	25·1	21·8	36·1	7·3	9·7
**Bosnia and Herzegovina**	25·7	22·9	36·3	8·2	6·9
**Bulgaria**	23·2	20·7	43·6	6·9	5·6
**Croatia**	23·3	20·4	43·1	7·4	5·7
**Czech Republic**	21·4	19·9	45·9	7	5·7
**Hungary**	24·6	19·6	43·4	7·3	5·1
**Montenegro**	24	21·1	41	7·5	6·5
**North Macedonia**	24·8	21·9	37·5	8·4	7·3
**Poland**	22·1	20·3	44·9	7·1	5·6
**Romania**	22	20·7	43·8	7·4	6
**Serbia**	23·3	21·6	41·5	7·6	6
**Slovakia**	21·9	20·2	45·2	7·1	5·5
**Slovenia**	22·7	20·9	42·8	8	5·6
**Central Asia**	22·4	23·5	35·3	7·7	11·2
**Armenia**	21·7	24·9	35·9	8·2	9·3
**Azerbaijan**	22·6	23·8	35	8·6	10
**Georgia**	22·3	24·2	39	8	6·5
**Kazakhstan**	21·1	23·7	39	7·7	8·5

**Kyrgyzstan**	23·4	24·8	35·1	7·3	9·5
**Mongolia**	22·4	24·5	36·1	7·3	9·6
**Tajikistan**	21·4	21·8	30·2	6·2	20·4
**Turkmenistan**	23	23·6	34·3	7·5	11·5
**Uzbekistan**	22·8	23·1	33·8	7·7	12·5
**Central Latin America**	6·4	31·9	36·3	15·4	9·9
**Colombia**	6·6	28	40·1	13·8	11·5
**Costa Rica**	6·1	28·8	41·4	15·6	8
**El Salvador**	6·9	31·1	37·1	15·8	9·1
**Guatemala**	8	33·5	32·3	13·8	12·4
**Honduras**	7·2	32·4	31·8	15·1	13·5
**Mexico**	6·1	32·4	36·5	15·6	9·4
**Nicaragua**	6·9	31·6	35·6	16·9	9·1
**Panama**	6·7	30·4	37·7	13·2	12·1
**Venezuela**	6·8	30	38·5	16·5	8·3
**Andean Latin America**	11·9	14	38	21	15·1
**Bolivia**	11·5	14·1	36·1	21·1	17·1
**Ecuador**	12·2	14·9	33·1	24	15·8
**Peru**	11·8	13·5	41·6	19·2	13·9
**Caribbean**	11·1	23	34·8	18·4	12·7
**Antigua and Barbuda**	10·3	22·6	33·6	21·6	11·8
**The Bahamas**	10·7	23·1	36·6	18·7	11
**Barbados**	10·6	22·7	34·9	21·5	10·3
**Belize**	10·9	22·5	33·9	19·5	13·2
**Bermuda**	11	20·7	36·9	21·9	9·5
**Cuba**	10·5	23·7	35·5	20·4	9·9
**Dominica**	10·7	22·3	35·1	19·2	12·6
**Dominican Republic**	10·9	22·3	35·1	17·2	14·4
**Grenada**	10·6	22·9	36·2	19·2	11·1
**Guyana**	11	23·4	35·5	18·7	11·4
**Haiti**	12·5	23·4	33·9	14·9	15·3
**Jamaica**	10·9	23	31·5	19·3	15·3
**Puerto Rico**	10·2	22·3	35·4	21·8	10·3
**Saint Lucia**	10·3	22·5	35·3	21·1	10·8
**Saint Vincent and the Grenadines**	10·4	21·9	34·3	23·9	9·6

**Suriname**	11·3	22·6	35·1	19	11·9
**Trinidad and Tobago**	11·2	22·9	32·9	21·6	11·4
**Virgin Islands**	10·1	21·8	37·4	21·3	9·5
**Tropical Latin America**	16·4	27	22·6	21·7	12·3
**Brazil**	16·4	27·1	22·5	21·7	12·3
**Paraguay**	16·4	23·9	27·1	19·5	13·1
**East Asia**	48·9	22·3	18·1	7	3·7
**China**	49	22·1	18·1	7·1	3·7
**North Korea**	50·3	23·4	17	5·2	4·1
**Taiwan (province of China)**	44·8	26	18·9	7·3	3
**Southeast Asia**	33·5	34·5	14·6	8·9	8·5
**Cambodia**	35·3	29	15·9	9·1	10·6
**Indonesia**	28·8	41·7	11·3	9	9·2
**Laos**	37·9	27	17·2	7·9	10·1
**Malaysia**	44·2	19·7	18·1	11·8	6·3
**Maldives**	35·4	29	16·4	11·5	7·6
**Mauritius**	35	29·3	19	10·7	5·9
**Myanmar**	38·4	29·8	15·4	7·5	9
**Philippines**	36·5	28·5	18·3	8·2	8·6
**Sri Lanka**	36·4	30·7	17·8	8·4	6·6
**Seychelles**	35·1	29·5	17·7	12·1	5·5
**Thailand**	35	28·1	17·8	12·7	6·5
**East Timor**	37·4	28·2	14·7	8	11·7
**Vietnam**	38·6	28·5	18·8	7·1	7
**Oceania**	43·7	22·8	14·6	6·5	12·4
**American Samoa**	41·1	21·1	13·7	15	9·1
**Federated States of Micronesia**	41·5	23	14·2	10	11·3
**Fiji**	40·2	23·6	14·7	9·6	11·9
**Guam**	43·1	22·3	16·6	10·8	7·3
**Kiribati**	43·1	21·8	13	9·5	12·5
**Marshall Islands**	42·8	22·3	14·2	10	10·6
**Northern Mariana Islands**	43	23·2	16·1	10·9	6·8
**Papua New Guinea**	43·9	22·8	14·7	5·9	12·7
**Samoa**	41·4	21·8	14·2	11·3	11·2
**Solomon Islands**	43·4	23·3	14·5	8	10·8

**Tonga**	39·7	21·9	13·9	12·3	12·2
**Vanuatu**	44·8	22·9	13·7	7·4	11·3
**North Africa and Middle East**	36·1	34·4	5·1	11·9	12·6
**Afghanistan**	28·2	29·7	4·3	7·1	30·8
**Algeria**	29·7	37·8	5·8	11·9	14·8
**Bahrain**	30·3	40·1	7	14·7	7·9
**Egypt**	40·6	34·1	4·7	12	8·6
**Iran**	27·7	41·9	4·9	12·4	13·1
**Iraq**	27·4	32·1	4·8	10·1	25·6
**Jordan**	27·8	35·6	5·4	13·9	17·3
**Kuwait**	29·1	39·4	6·3	17·5	7·7
**Lebanon**	32·9	35	5·5	12·3	14·3
**Libya**	28·4	36	5·5	15	15·1
**Morocco**	29·6	37·7	5·6	12	15
**Palestine**	28·3	34·8	5·1	11·3	20·4
**Oman**	29·6	38·2	6·1	14·1	12
**Qatar**	30·4	38·6	6·6	15·9	8·5
**Saudi Arabia**	21·6	49	6·1	15·5	7·8
**Sudan**	28·5	31·4	5·2	8·8	26
**Syria**	29·3	36·1	5·5	12·1	17
**Tunisia**	12·7	51·8	7·2	15·3	12·9
**Turkey**	52·4	16·6	6·4	13·6	11
**United Arab Emirates**	31·6	41·6	7·6	14·6	4·6
**Yemen**	27·6	33·9	5·1	7·8	25·7
**South Asia**	32·8	20·3	27·4	4·9	14·5
**Bangladesh**	27·6	23·5	25	5·1	18·7
**Bhutan**	29·5	22·6	27·6	5·4	14·9
**India**	35·3	16·3	31·5	4·8	12·1
**Nepal**	22·6	14·8	44	4·6	13·9
**Pakistan**	25·7	36·8	9	5·4	23·1
**Southern sub-Saharan Africa**	21·8	32·8	18·9	8·9	17·6
**Botswana**	21·4	34·3	18·5	8	17·8
**eSwatini**	22·7	34·5	17·1	8	17·6
**Lesotho**	22·6	34·5	18·6	7·4	16·9
**Namibia**	23·5	33·3	20	6·1	17

**South Africa**	21	32·6	19·9	10·1	16·3
**Zimbabwe**	23·7	32·7	16	6·1	21·5
**Western sub-Saharan Africa**	46	7·2	18	5·5	23·4
**Benin**	46·5	7·1	15	6·1	25·3
**Burkina Faso**	45·2	6·9	15·3	5·1	27·5
**Cameroon**	47·6	7·7	17·3	7·1	20·2
**Cape Verde**	49·5	8·6	19·3	7·2	15·4
**Chad**	50·9	6·7	14·1	4·3	23·9
**Côte d'Ivoire**	49·4	7·6	16·9	5·6	20·6
**The Gambia**	49·2	7·5	15·8	5·9	21·5
**Ghana**	47·2	8·3	17·2	6·9	20·4
**Guinea**	50·5	7·1	14·1	5	23·3
**Guinea-Bissau**	51·6	6·9	14·6	5·9	21
**Liberia**	49·9	6·7	14·6	5·9	22·9
**Mali**	44·7	6·4	10·7	4·6	33·5
**Mauritania**	48·9	7·8	13·4	7·4	22·5
**Niger**	47·3	6·2	11·4	4·2	30·9
**Nigeria**	44·6	7·1	19·7	5·3	23·3
**São Tomé and Príncipe**	49·1	7·7	17·4	6·2	19·6
**Senegal**	51	7·4	12·8	5·7	23
**Sierra Leone**	48·9	7·3	16·7	5·5	21·5
**Togo**	52·5	7·6	14·5	5·6	19·8
**Eastern sub-Saharan Africa**	25·6	27·3	19	6·4	21·7
**Burundi**	23·1	29·5	22·1	5·9	19·4
**Comoros**	24·5	31·1	16·7	7·2	20·6
**Djibouti**	25·7	29·4	19·1	7·7	18
**Eritrea**	25·1	30	18·7	5·7	20·5
**Ethiopia**	28·9	32	15	5	19·1
**Kenya**	28·2	13·3	26·4	9·4	22·7
**Madagascar**	24·7	29·9	18·2	5·9	21·3
**Malawi**	25·1	28·5	17	6·2	23·2
**Mozambique**	25·1	24·7	16·3	5·7	28·1
**Rwanda**	14·3	44·9	18·2	5·1	17·5
**Somalia**	25·8	28·6	17·5	6·1	22
**South Sudan**	26·5	27·4	18	6·6	21·4

**Tanzania**	21·9	27·2	18·3	6·5	26·2
**Uganda**	22·7	27·7	21·4	6	22·2
**Zambia**	24·8	28·9	18·3	6·3	21·7
**Central sub-Saharan Africa**	30·7	30·3	15·4	5·1	18·5
**Angola**	29·6	30·1	15·8	6	18·4
**Central African Republic**	32·1	30·5	15·4	4·8	17·2
**Congo**	30·4	31·7	16·4	5·9	15·6
**DR Congo**	31·2	30·2	15·1	4·6	18·9
**Equatorial Guinea**	29·3	29·1	16·1	8·1	17·4
**Gabon**	28·4	32·7	17·8	6·9	14·1

Appendix Table 11: Number of deaths and age-standardised death rates of cirrhosis due to hepatitis B per 100 000 in 1990 and 2017 for both sexes and the percentage change by location

	1990		2017		Percentage change in age-standardised rates between 1990 and 2017
	Counts (95% UI)	Rate (95% UI)	Counts (95% UI)	Rate (95% UI)	
**Global**	287011 (251678, 318059)	6.7 (5.8, 7.4)	383971 (349069, 441666)	4.8 (4.3, 5.5)	-28.3 (-35.9, -15.2)
**High-income North America**	2647 (2442, 2856)	0.8 (0.7, 0.8)	3871 (3529, 4215)	0.7 (0.6, 0.7)	-11.6 (-15.6, -7.4)
**Canada**	186 (164, 212)	0.6 (0.5, 0.6)	289 (249, 332)	0.5 (0.4, 0.5)	-20.1 (-27.3, -12.2)
**Greenland**	0 (0, 0)	0.6 (0.5, 0.7)	0 (0, 1)	0.6 (0.4, 0.7)	0.8 (-29.2, 29.2)
**USA**	2461 (2275, 2649)	0.8 (0.7, 0.9)	3582 (3265, 3894)	0.7 (0.7, 0.8)	-10.8 (-14.9, -6.2)
**Australasia**	298 (268, 331)	1.3 (1.2, 1.4)	414 (356, 477)	0.9 (0.8, 1)	-29.3 (-37.2, -19.6)
**Australia**	259 (231, 289)	1.3 (1.2, 1.5)	363 (307, 423)	0.9 (0.8, 1.1)	-29.1 (-37.9, -18.1)
**New Zealand**	38 (35, 42)	1 (0.9, 1.1)	51 (45, 56)	0.7 (0.6, 0.8)	-31.3 (-37.1, -24.8)
**High-income Asia Pacific**	9377 (8606, 10190)	4.6 (4.2, 5)	7449 (6608, 8318)	1.9 (1.7, 2.2)	-58.3 (-62.6, -54.2)
**Brunei**	5 (4, 7)	4.1 (3.2, 5.2)	8 (6, 11)	2.2 (1.7, 3.1)	-46.7 (-57.2, -24.5)
**Japan**	3486 (3225, 3746)	2.1 (1.9, 2.2)	4021 (3651, 4389)	1.2 (1.1, 1.3)	-42.6 (-46.5, -39.3)
**Singapore**	111 (101, 121)	4.6 (4.1, 5)	138 (120, 158)	2 (1.7, 2.3)	-56.9 (-61.6, -51.9)
**South Korea**	5774 (5173, 6419)	16.6 (15, 18.4)	3282 (2732, 3887)	3.9 (3.3, 4.6)	-76.4 (-79.9, -72.6)
**Western Europe**	7532 (6299, 8975)	1.4 (1.2, 1.6)	6027 (4964, 7272)	0.7 (0.6, 0.9)	-46 (-49.6, -42.4)

**Andorra**	1<br>(0, 1)	0.9<br>(0.6, 1.3)	1<br>(1, 1)	0.6<br>(0.4, 0.9)	-36<br>(-52.2, -14.8)
**Austria**	185<br>(144, 233)	1.7<br>(1.4, 2.2)	133<br>(103, 171)	0.8<br>(0.7, 1.1)	-51.4<br>(-56.8, -45.4)
**Belgium**	191<br>(155, 234)	1.3<br>(1.1, 1.6)	192<br>(152, 238)	0.9<br>(0.7, 1.2)	-29.3<br>(-38, -21.4)
**Cyprus**	8<br>(6, 11)	1<br>(0.7, 1.4)	10<br>(7, 13)	0.5<br>(0.4, 0.7)	-46.2<br>(-59.9, -29.9)
**Denmark**	64<br>(51, 81)	0.9<br>(0.7, 1.2)	79<br>(62, 102)	0.8<br>(0.6, 1)	-14.6<br>(-26.1, -1.1)
**Finland**	54<br>(43, 68)	0.8<br>(0.6, 1)	105<br>(80, 138)	1.1<br>(0.9, 1.5)	39.3<br>(19.9, 60.8)
**France**	1087<br>(867, 1348)	1.4<br>(1.1, 1.8)	840<br>(646, 1083)	0.7<br>(0.6, 0.9)	-49.2<br>(-55.7, -42.7)
**Germany**	1704<br>(1349, 2119)	1.5<br>(1.2, 1.8)	1440<br>(1081, 1863)	0.9<br>(0.7, 1.2)	-38.9<br>(-47.4, -29.7)
**Greece**	184<br>(143, 233)	1.2<br>(1, 1.5)	132<br>(103, 166)	0.6<br>(0.5, 0.8)	-50.1<br>(-55.9, -43.2)
**Iceland**	1<br>(1, 1)	0.3<br>(0.3, 0.4)	1<br>(1, 2)	0.3<br>(0.2, 0.4)	-16<br>(-25.5, -4.4)
**Ireland**	17<br>(14, 21)	0.4<br>(0.3, 0.5)	34<br>(27, 43)	0.5<br>(0.4, 0.6)	16<br>(-0.1, 32)
**Israel**	40<br>(31, 51)	0.8<br>(0.6, 1)	61<br>(48, 78)	0.5<br>(0.4, 0.7)	-33.7<br>(-40.7, -25.8)
**Italy**	2123<br>(1870, 2413)	2.4<br>(2.1, 2.7)	1268<br>(1072, 1492)	0.9<br>(0.8, 1.1)	-62.8<br>(-67.1, -58.4)
**Luxembourg**	8<br>(7, 11)	1.6<br>(1.3, 2)	7<br>(6, 10)	0.8<br>(0.6, 1)	-50.1<br>(-59.2, -40.4)
**Malta**	3<br>(3, 4)	0.8<br>(0.6, 0.9)	4<br>(3, 5)	0.5<br>(0.4, 0.6)	-39.5<br>(-46.2, -30.7)
**Netherlands**	124<br>(100, 152)	0.6<br>(0.5, 0.8)	141<br>(109, 183)	0.4<br>(0.3, 0.6)	-31<br>(-38.2, -23.6)

**Norway**	31<br>(27, 35)	0.5<br>(0.4, 0.6)	32<br>(28, 37)	0.4<br>(0.3, 0.4)	-28.7<br>(-36.4, -22.7)
**Portugal**	315<br>(249, 394)	2.4<br>(1.9, 3)	170<br>(131, 218)	0.8<br>(0.7, 1.1)	-65<br>(-69.1, -60.1)
**Spain**	938<br>(753, 1170)	1.8<br>(1.4, 2.2)	618<br>(486, 776)	0.7<br>(0.5, 0.8)	-62<br>(-66.1, -57.8)
**Sweden**	72<br>(64, 81)	0.5<br>(0.5, 0.6)	93<br>(80, 108)	0.5<br>(0.4, 0.6)	-8.5<br>(-16.3, 0.6)
**Switzerland**	71<br>(56, 89)	0.7<br>(0.6, 0.9)	82<br>(64, 105)	0.5<br>(0.4, 0.7)	-28.5<br>(-38.1, -17.3)
**UK**	303<br>(269, 339)	0.4<br>(0.3, 0.4)	577<br>(506, 648)	0.6<br>(0.5, 0.6)	47.8<br>(41.7, 57.5)
**Southern Latin America**	1381<br>(1185, 1608)	2.9<br>(2.5, 3.4)	1648<br>(1361, 1953)	2<br>(1.7, 2.4)	-30<br>(-36.8, -22.4)
**Argentina**	750<br>(640, 875)	2.3<br>(2, 2.7)	939<br>(765, 1127)	1.8<br>(1.5, 2.2)	-21.8<br>(-32.5, -10.2)
**Chile**	553<br>(462, 652)	5.1<br>(4.3, 6.1)	643<br>(525, 778)	2.8<br>(2.3, 3.4)	-45.9<br>(-53.1, -37.4)
**Uruguay**	78<br>(66, 92)	2<br>(1.7, 2.4)	67<br>(54, 80)	1.3<br>(1, 1.5)	-37.1<br>(-45.8, -27.1)
**Eastern Europe**	6291<br>(5711, 6938)	2.3<br>(2.1, 2.5)	14211<br>(12984, 15516)	4.8<br>(4.4, 5.2)	112.5<br>(102.8, 124.2)
**Belarus**	168<br>(142, 201)	1.3<br>(1.1, 1.5)	424<br>(353, 505)	3<br>(2.5, 3.5)	130<br>(91.4, 168)
**Estonia**	28<br>(24, 33)	1.4<br>(1.2, 1.7)	47<br>(38, 58)	2.4<br>(1.9, 2.9)	66.9<br>(37.4, 97.5)
**Latvia**	48<br>(41, 57)	1.4<br>(1.2, 1.6)	73<br>(61, 87)	2.4<br>(2, 2.9)	76.6<br>(50.4, 106.1)
**Lithuania**	71<br>(61, 83)	1.6<br>(1.4, 1.9)	158<br>(134, 185)	3.7<br>(3.2, 4.4)	134.1<br>(110.6, 161.2)
**Moldova**	640<br>(552, 745)	13.6<br>(11.8, 15.7)	555<br>(469, 644)	10<br>(8.6, 11.5)	-26<br>(-32.7, -19.6)

**Russia**	3635<br>(3230, 4046)	2<br>(1.8, 2.2)	9199<br>(8434, 9943)	4.5<br>(4.1, 4.8)	122.7<br>(109.8, 146.2)
**Ukraine**	1701<br>(1548, 1888)	2.4<br>(2.2, 2.7)	3756<br>(3344, 4178)	6<br>(5.4, 6.7)	147.9<br>(120.4, 175.5)
**Central Europe**	7615<br>(6833, 8501)	5.1<br>(4.6, 5.7)	6919<br>(6134, 7770)	3.6<br>(3.2, 4.1)	-28.4<br>(-32, -25)
**Albania**	72<br>(61, 82)	3.3<br>(2.7, 3.7)	78<br>(60, 106)	1.9<br>(1.5, 2.6)	-40.6<br>(-55.3, -12.4)
**Bosnia and Herzegovina**	217<br>(190, 260)	4.8<br>(4.2, 5.6)	148<br>(120, 209)	2.6<br>(2.1, 3.7)	-47<br>(-54.7, -32.5)
**Bulgaria**	483<br>(427, 550)	3.9<br>(3.5, 4.4)	469<br>(407, 538)	3.8<br>(3.3, 4.4)	-2<br>(-12.3, 9)
**Croatia**	459<br>(405, 518)	7<br>(6.3, 7.8)	254<br>(218, 293)	3.2<br>(2.8, 3.7)	-54.2<br>(-58.3, -49.4)
**Czech Republic**	549<br>(489, 624)	4.1<br>(3.7, 4.7)	462<br>(399, 531)	2.6<br>(2.2, 3)	-37.9<br>(-44.5, -30.6)
**Hungary**	1469<br>(1314, 1641)	10.7<br>(9.6, 11.9)	791<br>(679, 914)	4.7<br>(4.1, 5.4)	-55.8<br>(-59.9, -50.9)
**Montenegro**	10<br>(9, 12)	1.6<br>(1.3, 1.9)	12<br>(9, 14)	1.3<br>(1, 1.5)	-21.1<br>(-36.8, -3.9)
**North Macedonia**	43<br>(37, 51)	2.2<br>(1.9, 2.6)	59<br>(47, 70)	1.8<br>(1.4, 2.1)	-18.1<br>(-32.6, -3.4)
**Poland**	1221<br>(1084, 1397)	2.7<br>(2.4, 3.1)	1692<br>(1460, 1963)	2.8<br>(2.4, 3.3)	3.5<br>(-8.7, 15.4)
**Romania**	2065<br>(1839, 2342)	7.3<br>(6.5, 8.2)	2199<br>(1908, 2519)	6.6<br>(5.8, 7.5)	-8.8<br>(-16.7, -0.5)
**Serbia**	414<br>(321, 503)	3.5<br>(2.7, 4.2)	301<br>(257, 353)	2.1<br>(1.8, 2.4)	-40.2<br>(-50.6, -24.5)
**Slovakia**	415<br>(350, 519)	7<br>(5.9, 8.7)	332<br>(266, 394)	3.9<br>(3.1, 4.6)	-44.2<br>(-59.8, -31.9)
**Slovenia**	197<br>(174, 222)	7.9<br>(7, 8.9)	122<br>(105, 144)	3.3<br>(2.8, 3.8)	-58.7<br>(-63.3, -54.2)

**Central Asia**	3146 (2772, 3579)	6.3 (5.5, 7.1)	6755 (5861, 7734)	8.4 (7.4, 9.6)	34.8 (24.2, 44.1)
**Armenia**	103 (89, 119)	3.7 (3.2, 4.2)	236 (200, 270)	5.8 (5, 6.7)	58.6 (43.8, 75.4)
**Azerbaijan**	343 (300, 390)	6.5 (5.6, 7.4)	701 (554, 864)	8 (6.2, 10.1)	23.2 (3.2, 51.4)
**Georgia**	354 (306, 410)	5.6 (4.9, 6.4)	302 (253, 360)	5.7 (4.7, 6.8)	0.2 (-10.9, 13.3)
**Kazakhstan**	502 (437, 584)	3.7 (3.3, 4.3)	1409 (1113, 1688)	7.9 (6.3, 9.4)	110.8 (74.2, 142.2)
**Kyrgyzstan**	263 (231, 300)	8.3 (7.2, 9.4)	494 (414, 577)	9.7 (8.2, 11.3)	17 (1.9, 29.2)
**Mongolia**	177 (149, 215)	15.7 (13.5, 18.7)	302 (253, 359)	13.3 (10.9, 15.6)	-15.2 (-32.5, -1.5)
**Tajikistan**	181 (156, 230)	5.8 (4.9, 7.5)	334 (282, 399)	5.4 (4.6, 6.6)	-5.8 (-17.2, 7)
**Turkmenistan**	207 (184, 234)	9.6 (8.4, 10.8)	526 (441, 612)	11.5 (9.7, 13.2)	19.8 (5.4, 34.6)
**Uzbekistan**	1016 (895, 1154)	8 (7.1, 9.1)	2452 (2063, 2897)	9.6 (8.1, 11.3)	19.3 (5.1, 34.7)
**Central Latin America**	2342 (2169, 2509)	2.4 (2.2, 2.6)	3720 (3390, 4064)	1.6 (1.4, 1.7)	-34.8 (-38.2, -30.7)
**Colombia**	155 (139, 172)	0.8 (0.7, 0.9)	266 (224, 315)	0.5 (0.4, 0.6)	-40.6 (-47.9, -31.4)
**Costa Rica**	22 (20, 25)	1.2 (1.1, 1.3)	52 (44, 61)	1.1 (0.9, 1.2)	-10.3 (-20.3, 2.5)
**El Salvador**	74 (62, 84)	2.2 (1.9, 2.5)	99 (78, 131)	1.7 (1.3, 2.3)	-22.8 (-38.8, 8.6)
**Guatemala**	176 (160, 195)	3.8 (3.5, 4.3)	353 (304, 410)	2.9 (2.5, 3.3)	-24.6 (-34.2, -13.7)
**Honduras**	80 (64, 113)	3.2 (2.6, 4.4)	168 (124, 235)	2.6 (1.9, 3.6)	-19.1 (-38.3, 1.4)

**Mexico**	1633<br>(1516, 1741)	3.2<br>(3, 3.5)	2420<br>(2214, 2624)	2.1<br>(1.9, 2.2)	-36.3<br>(-39.6, -32.5)
**Nicaragua**	34<br>(29, 39)	1.8<br>(1.6, 2.1)	75<br>(54, 89)	1.5<br>(1.1, 1.8)	-16.7<br>(-36.4, -2.2)
**Panama**	16<br>(14, 18)	1<br>(0.9, 1.1)	31<br>(27, 36)	0.8<br>(0.7, 0.9)	-22.8<br>(-30.9, -14)
**Venezuela**	152<br>(137, 170)	1.4<br>(1.3, 1.6)	256<br>(209, 315)	0.9<br>(0.7, 1.1)	-36.7<br>(-47.4, -23.7)
**Andean Latin America**	809<br>(704, 938)	3.6<br>(3.1, 4.2)	1311<br>(1129, 1528)	2.4<br>(2.1, 2.8)	-32.6<br>(-41.7, -23.7)
**Bolivia**	152<br>(110, 206)	4.5<br>(3.2, 6)	257<br>(196, 339)	3<br>(2.3, 4)	-32.5<br>(-48, -8.1)
**Ecuador**	218<br>(195, 243)	3.7<br>(3.3, 4.1)	412<br>(352, 476)	2.8<br>(2.4, 3.2)	-23.2<br>(-32.1, -12.6)
**Peru**	439<br>(375, 510)	3.3<br>(2.8, 3.9)	641<br>(516, 789)	2.1<br>(1.7, 2.6)	-37.4<br>(-49.3, -24.1)
**Caribbean**	679<br>(586, 782)	2.5<br>(2.1, 2.9)	800<br>(671, 982)	1.6<br>(1.3, 1.9)	-36.6<br>(-43.3, -20.9)
**Antigua and Barbuda**	1<br>(1, 1)	2.1<br>(1.8, 2.4)	1<br>(1, 1)	1.1<br>(0.9, 1.3)	-46.3<br>(-52.5, -38.7)
**The Bahamas**	5<br>(4, 5)	2.6<br>(2.3, 3)	6<br>(5, 7)	1.4<br>(1.2, 1.6)	-46.1<br>(-53.1, -38.2)
**Barbados**	4<br>(4, 5)	1.6<br>(1.3, 1.8)	4<br>(3, 5)	0.9<br>(0.7, 1)	-43.4<br>(-50.4, -34.9)
**Belize**	3<br>(2, 3)	2.6<br>(2.2, 3)	6<br>(5, 7)	2.1<br>(1.8, 2.5)	-18.4<br>(-27, -8.3)
**Bermuda**	1<br>(1, 1)	1.5<br>(1.3, 1.7)	1<br>(1, 1)	0.6<br>(0.5, 0.8)	-56.7<br>(-63.2, -46.5)
**Cuba**	139<br>(122, 159)	1.3<br>(1.2, 1.5)	194<br>(160, 235)	1.1<br>(0.9, 1.3)	-17.9<br>(-28.7, -6.2)
**Dominica**	1<br>(1, 1)	1.6<br>(1.4, 1.9)	1<br>(1, 1)	1.2<br>(1, 1.3)	-29.3<br>(-37.8, -20.3)

**Dominican Republic**	167<br>(111, 197)	4.1<br>(2.7, 4.9)	173<br>(127, 294)	1.9<br>(1.4, 3.1)	-54.2<br>(-67.4, 3.7)
**Grenada**	2<br>(2, 2)	2.6<br>(2.3, 3)	2<br>(2, 2)	1.4<br>(1.2, 1.6)	-48.2<br>(-53.9, -42.1)
**Guyana**	22<br>(19, 25)	4.8<br>(4.2, 5.4)	19<br>(15, 23)	2.8<br>(2.4, 3.4)	-40.8<br>(-49.9, -30.8)
**Haiti**	126<br>(84, 186)	3.6<br>(2.4, 5.5)	207<br>(142, 313)	2.9<br>(2, 4.5)	-17.8<br>(-36.1, 8.5)
**Jamaica**	20<br>(17, 23)	1.1<br>(0.9, 1.3)	20<br>(16, 27)	0.7<br>(0.6, 0.9)	-35.9<br>(-49.9, -11.2)
**Puerto Rico**	130<br>(113, 147)	3.5<br>(3.1, 4)	96<br>(82, 111)	1.5<br>(1.3, 1.7)	-57.5<br>(-61.9, -51.8)
**Saint Lucia**	3<br>(2, 3)	2.8<br>(2.4, 3.2)	3<br>(2, 3)	1.3<br>(1.1, 1.5)	-53.1<br>(-58.3, -47)
**Saint Vincent and the Grenadines**	1<br>(1, 2)	2<br>(1.7, 2.3)	2<br>(1, 2)	1.3<br>(1.1, 1.5)	-34.8<br>(-43.3, -24.3)
**Suriname**	9<br>(8, 10)	3.3<br>(2.8, 3.7)	14<br>(11, 16)	2.3<br>(1.9, 2.8)	-28.3<br>(-38.3, -17)
**Trinidad and Tobago**	19<br>(17, 22)	2.1<br>(1.9, 2.4)	20<br>(15, 25)	1.1<br>(0.9, 1.4)	-46.9<br>(-57.5, -34.4)
**Virgin Islands**	2<br>(2, 3)	2.6<br>(2.2, 3)	3<br>(2, 4)	1.7<br>(1.4, 2.1)	-33.6<br>(-46.9, -20.6)
**Tropical Latin America**	4367<br>(4085, 4637)	4.1<br>(3.8, 4.3)	5882<br>(5491, 6297)	2.5<br>(2.3, 2.6)	-39.8<br>(-42.5, -37.4)
**Brazil**	4309<br>(4032, 4573)	4.1<br>(3.9, 4.4)	5774<br>(5394, 6174)	2.5<br>(2.3, 2.6)	-40.1<br>(-42.7, -37.5)
**Paraguay**	57<br>(47, 79)	2.4<br>(2, 3.3)	108<br>(82, 163)	2<br>(1.5, 2.9)	-19.3<br>(-38.3, 6.2)
**East Asia**	87005<br>(70808, 94967)	8.9<br>(7.3, 9.8)	81733<br>(74282, 105303)	4.1<br>(3.7, 5.3)	-54.6<br>(-60.7, -35.6)
**China**	81337<br>(65309, 88976)	8.8<br>(7.1, 9.6)	75159<br>(68002, 97674)	3.9<br>(3.5, 5.1)	-55.4<br>(-61.7, -35.4)

**North Korea**	1558<br>(1246, 1955)	9<br>(7.2, 11.1)	2329<br>(1752, 3011)	7.5<br>(5.6, 9.6)	-16.7<br>(-38.4, 8)
**Taiwan (province of China)**	2660<br>(2469, 2851)	16.7<br>(15.5, 17.8)	2928<br>(2661, 3235)	8<br>(7.3, 8.8)	-52.2<br>(-55.7, -48.4)
**Southeast Asia**	38833<br>(33051, 44367)	13.6<br>(11.5, 15.5)	58253<br>(51057, 66332)	9.7<br>(8.5, 11)	-28.6<br>(-36.9, -18.1)
**Cambodia**	1592<br>(1201, 2084)	32<br>(24.6, 42.1)	3147<br>(2498, 3988)	27.5<br>(22, 34.8)	-14.2<br>(-31.9, 11.2)
**Indonesia**	17652<br>(15091, 19915)	16.7<br>(14, 19)	23532<br>(21116, 26665)	11.6<br>(10.3, 13)	-30.7<br>(-39.1, -18.8)
**Laos**	348<br>(242, 477)	14.7<br>(10.6, 20.3)	601<br>(401, 824)	13.1<br>(9, 17.9)	-11.2<br>(-39.4, 27.6)
**Malaysia**	708<br>(593, 816)	7.2<br>(5.8, 8.3)	1346<br>(1070, 1700)	5.3<br>(4.2, 6.7)	-26.4<br>(-41.1, -10.9)
**Maldives**	4<br>(2, 6)	4.4<br>(2.9, 5.9)	6<br>(5, 8)	2.1<br>(1.6, 2.6)	-51.7<br>(-64.8, -20.3)
**Mauritius**	97<br>(81, 112)	11.4<br>(9.6, 13.2)	82<br>(68, 97)	5<br>(4.2, 5.8)	-56.5<br>(-61.3, -50.7)
**Myanmar**	7085<br>(4998, 9296)	25.5<br>(18.1, 33.2)	8821<br>(6929, 10992)	17.8<br>(14.2, 22.2)	-30.2<br>(-47.7, -1.1)
**Philippines**	1554<br>(1323, 1834)	4.3<br>(3.6, 5)	4601<br>(3656, 5668)	6.1<br>(4.9, 7.5)	43.4<br>(20.8, 69.9)
**Sri Lanka**	1405<br>(1126, 1658)	11.6<br>(9.5, 13.6)	1448<br>(1103, 1868)	6<br>(4.6, 7.6)	-48.3<br>(-58.6, -36.2)
**Seychelles**	5<br>(4, 6)	9.2<br>(7.5, 11.3)	9<br>(8, 11)	8.4<br>(6.9, 10.1)	-9.1<br>(-22.9, 7.9)
**Thailand**	3549<br>(2911, 4257)	8.5<br>(7, 10.2)	5899<br>(4556, 7207)	6.1<br>(4.7, 7.4)	-28.6<br>(-40.9, -12.7)
**East Timor**	34<br>(22, 48)	9.3<br>(5.6, 13.5)	86<br>(50, 124)	10.7<br>(6.3, 15.3)	15.1<br>(-17.2, 64.6)
**Vietnam**	4748<br>(3445, 6319)	11.5<br>(8.3, 15.3)	8597<br>(6684, 11167)	9.2<br>(7.2, 11.8)	-20.4<br>(-45.1, 15.2)

**Oceania**	447<br>(384, 523)	11.7<br>(10, 13.5)	761<br>(625, 937)	9.1<br>(7.7, 10.9)	-22<br>(-36, -5.9)
**American Samoa**	2<br>(2, 2)	7.1<br>(5.5, 8.8)	2<br>(2, 3)	5.5<br>(4.8, 6.3)	-21.7<br>(-38.6, -2.1)
**Federated States of Micronesia**	8<br>(6, 11)	13.8<br>(10.4, 21.1)	6<br>(4, 8)	7.8<br>(5.4, 11)	-43.1<br>(-58.9, -21.6)
**Fiji**	17<br>(14, 21)	3.9<br>(3.1, 4.7)	26<br>(22, 31)	3.5<br>(3, 4.1)	-9.5<br>(-28.6, 16.3)
**Guam**	9<br>(7, 11)	10.7<br>(8.5, 12.7)	15<br>(13, 17)	8.4<br>(7.3, 9.9)	-21.2<br>(-36.2, 2.5)
**Kiribati**	6<br>(4, 8)	13.2<br>(9.4, 18.6)	8<br>(5, 11)	10.1<br>(6.6, 15.3)	-23<br>(-39, -3.1)
**Marshall Islands**	3<br>(2, 4)	14<br>(10.1, 17.4)	4<br>(2, 5)	10.4<br>(6.7, 13.8)	-26.2<br>(-41.8, -8.5)
**Northern Mariana Islands**	2<br>(2, 3)	10.3<br>(8.5, 12.8)	4<br>(3, 4)	7.7<br>(6.7, 8.9)	-24.9<br>(-41.8, -6.6)
**Papua New Guinea**	318<br>(264, 387)	12.7<br>(10.6, 15.3)	578<br>(446, 745)	9.6<br>(7.6, 12.2)	-23.9<br>(-40.7, -4.3)
**Samoa**	9<br>(7, 14)	9.6<br>(6.9, 15.3)	10<br>(7, 13)	6.9<br>(5.1, 9.6)	-28.2<br>(-42.8, -10.9)
**Solomon Islands**	20<br>(15, 24)	11.3<br>(8.9, 13.9)	32<br>(26, 39)	8.3<br>(6.8, 9.8)	-26.7<br>(-42, -8.1)
**Tonga**	7<br>(6, 8)	12.3<br>(10.4, 14.3)	8<br>(6, 10)	9.9<br>(8, 11.9)	-19.5<br>(-34.8, 0)
**Vanuatu**	17<br>(8, 28)	19.8<br>(9.4, 33.4)	27<br>(13, 48)	14.2<br>(6.8, 26.4)	-28.3<br>(-45.9, -3.3)
**North Africa and Middle East**	20545<br>(14326, 25080)	12.9<br>(8.3, 16.1)	29522<br>(22515, 37062)	7.4<br>(5.5, 9.4)	-42.5<br>(-55.9, -29.2)
**Afghanistan**	524<br>(231, 869)	7.9<br>(3.5, 13.3)	729<br>(434, 1061)	6.6<br>(4.1, 10.2)	-16.4<br>(-34.8, 46)
**Algeria**	566<br>(365, 884)	4.7<br>(2.9, 7.5)	933<br>(656, 1285)	3<br>(2, 4.1)	-37.4<br>(-51.9, -18)

**Bahrain**	14<br>(10, 17)	7.6<br>(5.6, 9.8)	26<br>(20, 34)	2.9<br>(2.2, 3.7)	-61.6<br>(-68.7, -53.5)
**Egypt**	13378<br>(8392, 15975)	62.2<br>(34.1, 76.5)	18553<br>(12843, 25048)	43.1<br>(26.6, 58.7)	-30.8<br>(-49.1, -11.9)
**Iran**	810<br>(678, 948)	3.2<br>(2.6, 3.8)	1549<br>(1382, 1726)	2.3<br>(2.1, 2.6)	-26.2<br>(-39.1, -12)
**Iraq**	323<br>(238, 436)	3.9<br>(2.9, 5.3)	368<br>(293, 452)	1.5<br>(1.2, 1.9)	-61.5<br>(-68.7, -51.8)
**Jordan**	66<br>(49, 90)	4.5<br>(3.3, 6.1)	134<br>(101, 171)	2.5<br>(1.8, 3.2)	-45<br>(-58.2, -24.6)
**Kuwait**	19<br>(15, 23)	2.4<br>(1.9, 2.9)	48<br>(37, 62)	1.8<br>(1.4, 2.3)	-24.2<br>(-39.1, -10.8)
**Lebanon**	111<br>(82, 146)	5.2<br>(3.9, 6.8)	179<br>(120, 240)	3.1<br>(2, 4.1)	-41.6<br>(-64.7, -22.8)
**Libya**	105<br>(71, 154)	5.5<br>(3.6, 8.3)	170<br>(108, 236)	3.9<br>(2.4, 5.3)	-30.2<br>(-51.3, 7)
**Morocco**	725<br>(493, 1062)	5.1<br>(3.3, 7.8)	1045<br>(771, 1370)	3.5<br>(2.6, 4.6)	-31.4<br>(-51.5, -1.5)
**Palestine**	40<br>(27, 55)	4.4<br>(3, 6.1)	69<br>(54, 87)	2.9<br>(2.2, 3.8)	-32.9<br>(-47.8, -7.2)
**Oman**	32<br>(22, 44)	4.4<br>(3, 6.1)	54<br>(36, 73)	2.6<br>(1.8, 3.6)	-40<br>(-57.7, -18.9)
**Qatar**	9<br>(7, 12)	8.2<br>(5.9, 10.8)	35<br>(24, 47)	4.2<br>(3, 5.6)	-48.9<br>(-60.8, -32.9)
**Saudi Arabia**	418<br>(179, 602)	7.1<br>(2.7, 10.5)	549<br>(345, 739)	4.2<br>(2.3, 5.7)	-41.8<br>(-60.1, -6.3)
**Sudan**	792<br>(397, 1383)	8.9<br>(4, 16.1)	1037<br>(588, 1659)	6<br>(3.1, 10.1)	-32.9<br>(-52.5, 1.7)
**Syria**	287<br>(226, 363)	5.3<br>(4.1, 6.7)	484<br>(356, 625)	4<br>(2.9, 5.2)	-24.2<br>(-41.1, -3.4)
**Tunisia**	76<br>(47, 115)	1.7<br>(1, 2.6)	120<br>(81, 172)	1.1<br>(0.7, 1.5)	-36.4<br>(-56.6, -9.5)

**Turkey**	1864<br>(1307, 2496)	5.2<br>(3.7, 7)	2746<br>(2323, 3208)	3.2<br>(2.7, 3.7)	-39.3<br>(-58.3, -13.1)
**United Arab Emirates**	27<br>(11, 42)	5.4<br>(2, 8.5)	131<br>(62, 196)	3.4<br>(1.5, 5.1)	-36.9<br>(-58.2, -2)
**Yemen**	343<br>(164, 611)	7<br>(3.2, 12.7)	535<br>(351, 797)	4.3<br>(2.7, 6.8)	-38.1<br>(-60.2, 8.3)
**South Asia**	51706<br>(45598, 60610)	7.5<br>(6.7, 8.7)	96367<br>(84335, 126218)	6.8<br>(5.9, 8.9)	-10.5<br>(-20.9, 7.2)
**Bangladesh**	6642<br>(4710, 8124)	10.7<br>(7.6, 13.2)	7440<br>(6103, 9425)	6<br>(4.9, 7.6)	-44.1<br>(-56.3, -14.9)
**Bhutan**	33<br>(26, 43)	11<br>(8.5, 14.3)	45<br>(31, 63)	6.6<br>(4.5, 9.2)	-39.9<br>(-57.7, -14.5)
**India**	37317<br>(31791, 48066)	6.7<br>(5.8, 8.4)	75603<br>(65343, 104321)	6.5<br>(5.7, 9.1)	-2.5<br>(-15.2, 13.9)
**Nepal**	822<br>(591, 1104)	7.2<br>(5.3, 9.7)	1281<br>(890, 1735)	5.7<br>(4, 7.7)	-20.4<br>(-42.2, 5.6)
**Pakistan**	6892<br>(4424, 9262)	11<br>(6.9, 14.9)	11998<br>(8750, 15707)	9.8<br>(6.9, 12.7)	-11<br>(-29.8, 17.4)
**Southern sub-Saharan Africa**	1510<br>(1187, 1744)	4.9<br>(3.7, 5.7)	1486<br>(1191, 1796)	2.5<br>(2, 3)	-48<br>(-60.5, -30.5)
**Botswana**	31<br>(20, 45)	4.8<br>(3.2, 6.9)	41<br>(29, 58)	2.8<br>(2.1, 3.9)	-41.7<br>(-56.6, -22.5)
**eSwatini**	27<br>(20, 37)	7.9<br>(5.8, 11)	29<br>(21, 40)	4.5<br>(3.4, 6.3)	-42.6<br>(-59.9, -18.5)
**Lesotho**	50<br>(21, 78)	4.7<br>(2, 7.3)	48<br>(22, 75)	3.7<br>(1.7, 5.7)	-21.7<br>(-47.6, 25.7)
**Namibia**	23<br>(11, 35)	3.1<br>(1.5, 4.7)	52<br>(31, 81)	3.4<br>(2, 5.3)	10.6<br>(-28.2, 64.1)
**South Africa**	1059<br>(814, 1257)	4.4<br>(3.3, 5.3)	969<br>(853, 1112)	2.1<br>(1.8, 2.4)	-52.6<br>(-59.5, -37.1)
**Zimbabwe**	321<br>(255, 390)	7.3<br>(5.9, 8.9)	347<br>(106, 620)	4.8<br>(1.5, 8.5)	-34.5<br>(-81.1, 17.6)

**Western sub-Saharan Africa**	26585 (16526, 37775)	27.9 (17.1, 39.7)	35529 (23817, 50557)	17.9 (12, 25.4)	-35.8 (-52.1, -14.8)
**Benin**	474 (326, 698)	22 (14.9, 32.6)	634 (446, 884)	12.3 (8.6, 17.1)	-43.9 (-70.8, -14.3)
**Burkina Faso**	1011 (803, 1191)	23 (18.5, 27.2)	1034 (77, 1886)	11.2 (0.8, 20.4)	-51.4 (-96.8, -6.3)
**Cameroon**	1254 (970, 1515)	25.9 (19.9, 31.3)	2012 (911, 3017)	15.6 (6.9, 23.5)	-39.5 (-67.8, -13)
**Cape Verde**	20 (16, 25)	8.9 (6.9, 11.1)	43 (37, 50)	9.1 (7.8, 10.7)	2 (-22.8, 35.1)
**Chad**	571 (360, 814)	18.9 (11.7, 26.9)	1118 (830, 1451)	18.2 (13.4, 23.9)	-3.5 (-27.7, 40.6)
**Côte d'Ivoire**	1052 (785, 1420)	22.1 (16.2, 30.3)	1810 (1359, 2366)	15.2 (11.5, 19.9)	-31.2 (-54.6, -7.7)
**The Gambia**	71 (42, 106)	17.8 (10.3, 26.1)	147 (104, 191)	14.4 (10.4, 18.4)	-18.9 (-44.5, 24)
**Ghana**	1407 (1131, 1745)	20.2 (16, 25)	1939 (635, 3447)	11.6 (3.7, 20.5)	-42.3 (-83.6, 0.9)
**Guinea**	738 (448, 1245)	21.4 (12.7, 36.4)	911 (586, 1421)	16 (10.1, 25)	-25.3 (-44.7, 13.1)
**Guinea-Bissau**	114 (75, 156)	25.7 (16.9, 34.1)	156 (117, 214)	19.3 (14.7, 25.9)	-25.2 (-44, 7.8)
**Liberia**	339 (239, 466)	29.2 (20.1, 41)	389 (286, 515)	18.2 (13.2, 24.3)	-37.7 (-53.3, -19.8)
**Mali**	997 (636, 1547)	23.9 (14.8, 38)	923 (336, 1635)	10.4 (3.6, 18.2)	-56.6 (-89, -21.8)
**Mauritania**	201 (150, 278)	18.7 (13.9, 25.7)	221 (154, 300)	10.6 (7.5, 14.4)	-43.1 (-56.5, -27.3)
**Niger**	562 (397, 884)	17.7 (12.4, 27.8)	809 (207, 1451)	10 (2.5, 17.6)	-43.3 (-82, -12.7)
**Nigeria**	16271 (7624, 25806)	33.8 (15.7, 53.9)	21601 (9263, 37489)	23.4 (10.1, 40.3)	-30.7 (-50.6, -0.3)

**São Tomé and Príncipe**	27 (17, 34)	37.9 (24.7, 48.2)	40 (30, 56)	35.2 (26.5, 48.8)	-7.3 (-39.2, 37.9)
**Senegal**	544 (340, 765)	15.7 (9.7, 22)	776 (308, 1184)	10.1 (3.9, 15.3)	-35.6 (-61.6, -15.5)
**Sierra Leone**	611 (409, 917)	29.6 (19.6, 44.8)	556 (219, 817)	14.5 (5.6, 21.7)	-50.8 (-87.5, -5.8)
**Togo**	320 (238, 428)	22.6 (16.7, 30.4)	409 (133, 631)	10.5 (3.3, 16.2)	-53.4 (-88.2, -16.9)
**Eastern sub-Saharan Africa**	10396 (8243, 13106)	13.1 (10.6, 16.1)	14977 (11326, 18744)	8.9 (6.6, 11.3)	-31.7 (-49.5, -8.2)
**Burundi**	425 (264, 645)	17.8 (11.4, 26.8)	395 (115, 690)	8.7 (2.4, 15.1)	-50.8 (-86.6, -17.3)
**Comoros**	12 (2, 23)	5.8 (0.7, 10.7)	22 (4, 37)	4.7 (0.9, 8.1)	-18.9 (-37.3, 28.8)
**Djibouti**	12 (8, 18)	7.2 (4.6, 10.4)	37 (23, 56)	6.2 (3.8, 9.5)	-13.8 (-40.6, 23.3)
**Eritrea**	145 (84, 238)	13.3 (8.4, 21.5)	277 (196, 381)	10.5 (7.6, 14.1)	-20.9 (-45.4, 24.7)
**Ethiopia**	3110 (1919, 4916)	15.2 (9.7, 22.9)	4708 (3938, 5651)	11.2 (9.5, 13.5)	-26 (-51.4, 23.6)
**Kenya**	1613 (1003, 2347)	18.1 (11.4, 26.2)	2884 (1883, 4083)	12.7 (8.5, 18.2)	-29.8 (-57.7, -7)
**Madagascar**	557 (365, 862)	9.9 (6.5, 15.4)	731 (503, 988)	6.4 (4.4, 8.6)	-35.5 (-64.9, 1.4)
**Malawi**	656 (357, 916)	15.4 (9.3, 21.2)	669 (130, 1129)	8.3 (1.6, 14)	-46.3 (-90.2, 12.5)
**Mozambique**	558 (347, 790)	8.7 (5.4, 12.2)	615 (94, 1079)	5.1 (0.8, 8.8)	-41.4 (-91.2, 22.9)
**Rwanda**	431 (318, 561)	13.8 (10.3, 17.8)	377 (231, 549)	6.2 (3.8, 8.9)	-55.1 (-73.4, -33)
**Somalia**	251 (113, 451)	9.1 (4.8, 15.5)	661 (392, 1030)	9.8 (5.8, 15.2)	8.4 (-26, 74.4)

**South Sudan**	231<br>(120, 392)	9.3<br>(5.3, 15.1)	382<br>(244, 576)	9.5<br>(6.2, 14.2)	3<br>(-29.2, 58)
**Tanzania**	1138<br>(763, 1562)	10<br>(7.1, 13.6)	1409<br>(539, 2294)	5.6<br>(2.1, 9.2)	-43.9<br>(-78.8, -10)
**Uganda**	668<br>(407, 979)	9.6<br>(5.7, 13.9)	988<br>(377, 1579)	6.6<br>(2.4, 10.4)	-31<br>(-62.8, 0.7)
**Zambia**	582<br>(417, 753)	18.7<br>(13.8, 24.3)	813<br>(618, 1047)	11.6<br>(8.8, 15)	-38.2<br>(-52.8, -20.3)
**Central sub-Saharan Africa**	3503<br>(2528, 4837)	13.7<br>(9.7, 19.1)	6335<br>(4743, 8277)	10.7<br>(7.9, 14.2)	-22.1<br>(-36.5, -1.9)
**Angola**	722<br>(393, 1175)	16.1<br>(8.5, 26.9)	1585<br>(1155, 2223)	12.8<br>(9.2, 18.6)	-20.5<br>(-44.7, 31)
**Central African Republic**	179<br>(100, 268)	12.8<br>(7.1, 18.6)	226<br>(95, 379)	8.8<br>(3.6, 14.3)	-31.4<br>(-53.7, -8.7)
**Congo**	186<br>(125, 286)	15.3<br>(10.2, 23.3)	307<br>(194, 450)	11<br>(6.8, 16.4)	-28.1<br>(-46.1, -0.9)
**DR Congo**	2286<br>(1619, 3218)	12.9<br>(9.1, 18.3)	4055<br>(2849, 5485)	10.1<br>(7.1, 14)	-21.6<br>(-39.8, 1.2)
**Equatorial Guinea**	25<br>(8, 45)	11.3<br>(3.4, 19.4)	44<br>(24, 70)	7.7<br>(4.1, 12.5)	-31.6<br>(-56.6, 44)
**Gabon**	104<br>(69, 149)	17.3<br>(11.4, 25.4)	118<br>(78, 178)	10.5<br>(6.8, 16.1)	-39.4<br>(-52.7, -22.5)

Appendix Table 12: Number of deaths and age-standardised death rates of cirrhosis due to hepatitis C per 100 000 in 1990 and 2017 for both sexes and the percentage change by location

	1990		2017		Percentage change in age-standardised rates between 1990 and 2017
	Counts (95% UI)	Rate (95% UI)	Counts (95% UI)	Rate (95% UI)	
**Global**	225266 (201660, 248588)	5.3 (4.8, 5.9)	342243 (312603, 381099)	4.2 (3.9, 4.7)	-20.5 (-26.7, -11.5)
**High-income North America**	13546 (12874, 14136)	4.1 (3.9, 4.2)	23355 (22041, 24592)	4.2 (3.9, 4.4)	2.8 (-1.3, 7.6)
**Canada**	937 (871, 1005)	2.9 (2.7, 3.1)	1501 (1359, 1646)	2.4 (2.2, 2.6)	-17.7 (-24.3, -10.3)
**Greenland**	2 (1, 2)	3.2 (2.7, 3.9)	2 (2, 3)	3.1 (2.3, 3.6)	-2.3 (-31.6, 25.9)
**USA**	12607 (11958, 13148)	4.2 (4, 4.4)	21851 (20620, 23005)	4.4 (4.1, 4.6)	4.6 (0.3, 9.9)
**Australasia**	545 (505, 586)	2.4 (2.2, 2.5)	898 (792, 1001)	2 (1.7, 2.2)	-16.4 (-25.5, -5.6)
**Australia**	476 (437, 516)	2.5 (2.3, 2.7)	793 (694, 894)	2.1 (1.8, 2.4)	-15.8 (-25.9, -3.5)
**New Zealand**	69 (64, 74)	1.8 (1.7, 1.9)	104 (95, 115)	1.4 (1.3, 1.5)	-21.5 (-28.2, -14.4)
**High-income Asia Pacific**	13673 (13109, 14189)	6.8 (6.5, 7)	14491 (13379, 15403)	3.4 (3.1, 3.6)	-50.2 (-53.8, -47.6)
**Brunei**	3 (2, 5)	2.6 (1.5, 3.9)	6 (4, 9)	1.6 (1, 2.5)	-38.3 (-50.7, -11.9)
**Japan**	12389 (11925, 12793)	7.3 (7, 7.5)	13630 (12602, 14484)	4.2 (3.8, 4.4)	-42.9 (-46.9, -40)
**Singapore**	18 (15, 22)	0.7 (0.6, 0.9)	25 (20, 31)	0.3 (0.3, 0.4)	-51.6 (-57.6, -45.1)
**South Korea**	1263 (1102, 1445)	3.6 (3.2, 4.1)	831 (684, 1000)	1 (0.8, 1.2)	-72.9 (-77, -68.7)
**Western Europe**	18996 (16045, 22487)	3.5 (2.9, 4.1)	15873 (13078, 19098)	1.9 (1.6, 2.3)	-44.4 (-48.2, -40.3)

**Andorra**	1<br>(1, 2)	1.9<br>(1.2, 2.8)	2<br>(1, 3)	1.4<br>(0.9, 2.1)	-26.7<br>(-45.5, -1.6)
**Austria**	410<br>(291, 556)	3.9<br>(2.7, 5.3)	331<br>(234, 448)	2.1<br>(1.5, 2.9)	-45.6<br>(-51.7, -39.2)
**Belgium**	252<br>(196, 313)	1.8<br>(1.4, 2.2)	293<br>(226, 371)	1.4<br>(1.1, 1.8)	-18<br>(-27.4, -8.9)
**Cyprus**	16<br>(11, 24)	2<br>(1.3, 2.9)	22<br>(16, 30)	1.2<br>(0.9, 1.6)	-39.3<br>(-54.5, -20.8)
**Denmark**	133<br>(94, 182)	1.9<br>(1.4, 2.7)	163<br>(112, 224)	1.7<br>(1.2, 2.3)	-14.6<br>(-27.4, 0.7)
**Finland**	109<br>(78, 150)	1.7<br>(1.2, 2.3)	217<br>(149, 301)	2.4<br>(1.6, 3.3)	41.9<br>(19.3, 62.9)
**France**	2245<br>(1596, 3023)	3<br>(2.1, 4)	1861<br>(1326, 2536)	1.6<br>(1.1, 2.2)	-45.8<br>(-52.9, -39.3)
**Germany**	3469<br>(2478, 4708)	3<br>(2.1, 4.1)	3358<br>(2335, 4586)	2.1<br>(1.5, 2.8)	-29.5<br>(-39.5, -18.4)
**Greece**	259<br>(184, 351)	1.7<br>(1.2, 2.3)	218<br>(153, 297)	1<br>(0.7, 1.4)	-41.1<br>(-48, -32.9)
**Iceland**	1<br>(1, 1)	0.4<br>(0.3, 0.5)	1<br>(1, 2)	0.3<br>(0.2, 0.4)	-20.4<br>(-30.1, -9.4)
**Ireland**	35<br>(25, 46)	0.9<br>(0.6, 1.2)	70<br>(50, 96)	1<br>(0.7, 1.4)	15.8<br>(0.8, 31.5)
**Israel**	78<br>(56, 105)	1.6<br>(1.2, 2.2)	140<br>(100, 189)	1.2<br>(0.9, 1.7)	-23.1<br>(-30.9, -14.4)
**Italy**	8224<br>(7605, 8861)	9.4<br>(8.8, 10.1)	5668<br>(5045, 6336)	4.1<br>(3.6, 4.6)	-56.7<br>(-61.1, -51.9)
**Luxembourg**	17<br>(12, 24)	3.2<br>(2.3, 4.5)	17<br>(12, 23)	1.9<br>(1.3, 2.6)	-42.7<br>(-52.8, -32.4)
**Malta**	7<br>(5, 9)	1.5<br>(1.1, 2)	8<br>(6, 11)	1<br>(0.7, 1.4)	-33.1<br>(-40.3, -23.3)
**Netherlands**	242<br>(176, 322)	1.2<br>(0.9, 1.7)	288<br>(205, 386)	0.9<br>(0.6, 1.2)	-28.7<br>(-36.2, -20.8)

**Norway**	63<br>(54, 71)	1<br>(0.9, 1.2)	66<br>(57, 77)	0.7<br>(0.6, 0.9)	-29.1<br>(-36.8, -23)
**Portugal**	625<br>(451, 848)	4.8<br>(3.5, 6.4)	393<br>(283, 538)	2<br>(1.4, 2.7)	-59<br>(-64.2, -53.6)
**Spain**	2098<br>(1681, 2557)	4<br>(3.2, 4.8)	1598<br>(1255, 1993)	1.8<br>(1.4, 2.2)	-55.9<br>(-60.3, -51.2)
**Sweden**	139<br>(121, 160)	1.1<br>(0.9, 1.2)	174<br>(150, 203)	0.9<br>(0.8, 1.1)	-11.5<br>(-19.5, -3.4)
**Switzerland**	143<br>(102, 194)	1.5<br>(1.1, 2)	166<br>(120, 223)	1<br>(0.8, 1.4)	-28.9<br>(-38.8, -16.7)
**UK**	413<br>(354, 487)	0.5<br>(0.4, 0.6)	803<br>(677, 956)	0.8<br>(0.7, 0.9)	49.4<br>(42, 60.1)
**Southern Latin America**	2754<br>(2405, 3125)	5.8<br>(5.1, 6.6)	3765<br>(3223, 4347)	4.7<br>(4, 5.4)	-19.6<br>(-27.4, -11.2)
**Argentina**	1488<br>(1303, 1702)	4.6<br>(4, 5.2)	2126<br>(1764, 2535)	4.1<br>(3.4, 4.9)	-10.2<br>(-22.7, 3.2)
**Chile**	1111<br>(954, 1273)	10.3<br>(8.9, 11.8)	1489<br>(1245, 1744)	6.4<br>(5.4, 7.5)	-38<br>(-46.3, -28.7)
**Uruguay**	155<br>(135, 178)	4<br>(3.5, 4.6)	150<br>(126, 178)	2.9<br>(2.4, 3.4)	-28.2<br>(-37.5, -16.9)
**Eastern Europe**	7357<br>(6704, 8010)	2.6<br>(2.4, 2.8)	19117<br>(17561, 20686)	6.3<br>(5.8, 6.8)	140.3<br>(129.3, 152.7)
**Belarus**	197<br>(166, 232)	1.5<br>(1.3, 1.8)	588<br>(474, 705)	4<br>(3.3, 4.9)	166.7<br>(122, 207.6)
**Estonia**	33<br>(28, 39)	1.6<br>(1.4, 1.9)	65<br>(53, 79)	3.2<br>(2.6, 3.9)	97.2<br>(61.1, 132.3)
**Latvia**	56<br>(48, 66)	1.6<br>(1.4, 1.9)	101<br>(83, 122)	3.3<br>(2.7, 3.9)	106.1<br>(75.3, 140.7)
**Lithuania**	82<br>(71, 95)	1.8<br>(1.6, 2.1)	217<br>(182, 255)	5<br>(4.2, 5.8)	174<br>(143.8, 206.2)
**Moldova**	768<br>(660, 892)	16.3<br>(14.1, 18.8)	770<br>(648, 894)	13.8<br>(11.7, 16)	-15.2<br>(-23.3, -8.1)

**Russia**	4231<br>(3770, 4623)	2.3<br>(2.1, 2.5)	12552<br>(11616, 13476)	6<br>(5.5, 6.4)	156.6<br>(140.9, 182.9)
**Ukraine**	1990<br>(1829, 2170)	2.8<br>(2.6, 3.1)	4823<br>(4282, 5393)	7.6<br>(6.7, 8.5)	168.4<br>(139.7, 198.2)
**Central Europe**	5877<br>(5212, 6624)	3.9<br>(3.5, 4.4)	6329<br>(5556, 7181)	3.3<br>(2.9, 3.8)	-14.9<br>(-19.1, -10.7)
**Albania**	53<br>(44, 61)	2.4<br>(1.9, 2.8)	69<br>(53, 93)	1.7<br>(1.3, 2.3)	-28.9<br>(-45.8, 4.2)
**Bosnia and Herzegovina**	167<br>(145, 197)	3.7<br>(3.2, 4.2)	133<br>(109, 188)	2.3<br>(1.9, 3.3)	-37.5<br>(-46.6, -18.8)
**Bulgaria**	359<br>(314, 409)	2.9<br>(2.5, 3.2)	421<br>(360, 492)	3.4<br>(2.9, 4)	19.4<br>(6.5, 33.2)
**Croatia**	346<br>(301, 395)	5.3<br>(4.6, 6)	222<br>(192, 256)	2.8<br>(2.5, 3.3)	-46.3<br>(-51.7, -40.6)
**Czech Republic**	418<br>(368, 473)	3.2<br>(2.8, 3.6)	433<br>(373, 502)	2.4<br>(2.1, 2.8)	-23.2<br>(-32.2, -13.6)
**Hungary**	1136<br>(997, 1289)	8.2<br>(7.2, 9.4)	628<br>(537, 721)	3.8<br>(3.2, 4.3)	-54.1<br>(-58.9, -49.2)
**Montenegro**	8<br>(6, 10)	1.2<br>(1, 1.5)	11<br>(8, 13)	1.1<br>(0.9, 1.4)	-6.4<br>(-25.1, 14.3)
**North Macedonia**	32<br>(28, 39)	1.6<br>(1.4, 1.9)	53<br>(42, 63)	1.6<br>(1.3, 1.9)	-2.7<br>(-21, 14.2)
**Poland**	939<br>(826, 1071)	2.1<br>(1.8, 2.4)	1589<br>(1378, 1839)	2.6<br>(2.3, 3.1)	25.6<br>(11.2, 41.3)
**Romania**	1613<br>(1415, 1833)	5.6<br>(4.9, 6.4)	2066<br>(1764, 2392)	6.2<br>(5.4, 7.2)	10.7<br>(0.5, 21.2)
**Serbia**	332<br>(255, 408)	2.7<br>(2.1, 3.3)	282<br>(238, 333)	1.9<br>(1.6, 2.3)	-29<br>(-42, -10.1)
**Slovakia**	318<br>(267, 391)	5.4<br>(4.5, 6.7)	310<br>(247, 366)	3.7<br>(2.9, 4.3)	-32.3<br>(-51.5, -16.8)
**Slovenia**	155<br>(135, 177)	6.2<br>(5.4, 7)	112<br>(95, 131)	3<br>(2.5, 3.5)	-51.6<br>(-57.3, -46)

**Central Asia**	2982<br>(2637, 3402)	6<br>(5.3, 6.9)	7419<br>(6439, 8525)	9.4<br>(8.1, 10.7)	55.3<br>(42.5, 66.4)
**Armenia**	99<br>(86, 115)	3.6<br>(3.1, 4.1)	277<br>(236, 318)	6.8<br>(5.9, 7.8)	92.2<br>(72.6, 111.2)
**Azerbaijan**	331<br>(284, 381)	6.4<br>(5.4, 7.3)	759<br>(595, 956)	8.7<br>(6.7, 11.2)	37<br>(14.8, 69.2)
**Georgia**	347<br>(299, 400)	5.5<br>(4.8, 6.3)	334<br>(282, 395)	6.2<br>(5.2, 7.3)	12.1<br>(-0.4, 27.5)
**Kazakhstan**	496<br>(430, 573)	3.7<br>(3.2, 4.3)	1629<br>(1278, 1961)	9.2<br>(7.3, 11)	145.6<br>(100.1, 183.1)
**Kyrgyzstan**	249<br>(218, 284)	7.9<br>(7, 9)	542<br>(449, 634)	10.7<br>(9, 12.5)	35.3<br>(17.9, 49.8)
**Mongolia**	163<br>(138, 196)	14.9<br>(12.7, 17.6)	341<br>(283, 407)	15.4<br>(12.2, 18.5)	3.4<br>(-20.2, 21.4)
**Tajikistan**	169<br>(145, 215)	5.6<br>(4.8, 7.2)	360<br>(304, 427)	6<br>(5.1, 7.4)	8<br>(-4.4, 22.1)
**Turkmenistan**	191<br>(167, 216)	9.1<br>(8, 10.3)	567<br>(478, 660)	12.5<br>(10.7, 14.4)	37.8<br>(22.7, 54.5)
**Uzbekistan**	936<br>(814, 1071)	7.6<br>(6.6, 8.7)	2609<br>(2185, 3113)	10.4<br>(8.8, 12.3)	37<br>(20.8, 55.9)
**Central Latin America**	10243<br>(9767, 10718)	10.7<br>(10.2, 11.2)	18383<br>(17306, 19616)	7.7<br>(7.2, 8.2)	-27.8<br>(-31.5, -23.4)
**Colombia**	541<br>(498, 585)	2.9<br>(2.7, 3.2)	1080<br>(930, 1250)	2<br>(1.7, 2.3)	-31.8<br>(-40, -21.6)
**Costa Rica**	89<br>(83, 96)	4.8<br>(4.4, 5.1)	238<br>(210, 272)	4.8<br>(4.2, 5.5)	0.3<br>(-10.4, 14)
**El Salvador**	280<br>(238, 305)	8.6<br>(7.3, 9.4)	447<br>(362, 586)	7.8<br>(6.3, 10.2)	-8.7<br>(-27.1, 27.9)
**Guatemala**	661<br>(608, 716)	14.8<br>(13.5, 16)	1536<br>(1346, 1735)	13<br>(11.4, 14.6)	-12.3<br>(-23, -0.2)
**Honduras**	300<br>(246, 420)	12.5<br>(10.4, 16.9)	770<br>(574, 1067)	12.1<br>(9, 16.9)	-3<br>(-24.7, 22.9)

**Mexico**	7576<br>(7240, 7908)	15.3<br>(14.6, 15.9)	12720<br>(12004, 13533)	10.7<br>(10.1, 11.4)	-29.6<br>(-32.9, -25.4)
**Nicaragua**	133<br>(117, 148)	7.3<br>(6.5, 8.2)	348<br>(250, 411)	7.2<br>(5.2, 8.4)	-2.4<br>(-25.2, 13.9)
**Panama**	61<br>(56, 67)	4<br>(3.7, 4.4)	138<br>(124, 154)	3.5<br>(3.1, 3.9)	-13.8<br>(-22.3, -3.9)
**Venezuela**	603<br>(556, 648)	5.6<br>(5.2, 6.1)	1107<br>(924, 1335)	3.8<br>(3.2, 4.6)	-32.6<br>(-43.7, -19.6)
**Andean Latin America**	782<br>(677, 903)	3.5<br>(3, 4.1)	1541<br>(1321, 1803)	2.8<br>(2.4, 3.3)	-19.3<br>(-29.8, -8.8)
**Bolivia**	148<br>(106, 204)	4.4<br>(3.2, 5.9)	312<br>(234, 414)	3.6<br>(2.7, 4.8)	-16.8<br>(-35.4, 12.4)
**Ecuador**	209<br>(185, 236)	3.6<br>(3.2, 4)	499<br>(431, 584)	3.4<br>(2.9, 4)	-4.8<br>(-15.4, 9.5)
**Peru**	425<br>(365, 496)	3.3<br>(2.8, 3.8)	729<br>(588, 888)	2.4<br>(1.9, 2.9)	-27.6<br>(-41.6, -11.4)
**Caribbean**	1217<br>(1046, 1425)	4.5<br>(3.9, 5.3)	1656<br>(1393, 2009)	3.2<br>(2.7, 3.9)	-28.1<br>(-35.4, -10.5)
**Antigua and Barbuda**	2<br>(2, 2)	3.8<br>(3.3, 4.4)	2<br>(2, 3)	2.4<br>(2.1, 2.8)	-36.7<br>(-44.2, -27.8)
**The Bahamas**	8<br>(7, 10)	4.9<br>(4.2, 5.6)	12<br>(10, 14)	3.1<br>(2.6, 3.6)	-37.1<br>(-45.3, -28.3)
**Barbados**	8<br>(7, 9)	2.8<br>(2.4, 3.2)	9<br>(7, 10)	1.8<br>(1.6, 2.2)	-34.5<br>(-42.7, -25.2)
**Belize**	5<br>(4, 5)	4.8<br>(4, 5.5)	13<br>(11, 15)	4.5<br>(3.9, 5.3)	-5.2<br>(-15.2, 6.4)
**Bermuda**	2<br>(1, 2)	2.7<br>(2.3, 3.2)	1<br>(1, 2)	1.2<br>(1, 1.4)	-57.2<br>(-63.5, -47.6)
**Cuba**	254<br>(221, 290)	2.4<br>(2.1, 2.8)	430<br>(353, 517)	2.4<br>(1.9, 2.8)	-2.2<br>(-14.9, 11.3)
**Dominica**	2<br>(2, 2)	3<br>(2.7, 3.5)	2<br>(2, 3)	2.4<br>(2.1, 2.8)	-20.9<br>(-29.9, -10.6)

**Dominican Republic**	295<br>(195, 356)	7.4<br>(4.8, 8.9)	357<br>(263, 603)	3.8<br>(2.8, 6.4)	-48.1<br>(-62.9, 18.6)
**Grenada**	3<br>(3, 4)	4.8<br>(4.2, 5.5)	4<br>(4, 5)	2.9<br>(2.5, 3.4)	-38.5<br>(-45, -31)
**Guyana**	38<br>(33, 43)	8.5<br>(7.4, 9.8)	40<br>(33, 50)	6.1<br>(5, 7.3)	-28.9<br>(-39.7, -16.5)
**Haiti**	231<br>(155, 348)	6.6<br>(4.5, 10.1)	392<br>(279, 588)	5.5<br>(4, 8.4)	-15.9<br>(-34.7, 10.8)
**Jamaica**	35<br>(30, 41)	2<br>(1.7, 2.3)	44<br>(35, 58)	1.5<br>(1.2, 2)	-25.2<br>(-40.9, 2.7)
**Puerto Rico**	231<br>(201, 262)	6.3<br>(5.5, 7.1)	207<br>(178, 238)	3.2<br>(2.8, 3.7)	-48.7<br>(-53.8, -42)
**Saint Lucia**	5<br>(4, 5)	5.2<br>(4.5, 6)	6<br>(5, 7)	2.8<br>(2.4, 3.3)	-45.6<br>(-51.9, -38.4)
**Saint Vincent and the Grenadines**	3<br>(2, 3)	3.6<br>(3, 4.1)	4<br>(3, 4)	2.7<br>(2.3, 3.1)	-25<br>(-34.4, -12.4)
**Suriname**	16<br>(14, 19)	5.9<br>(5.1, 6.9)	28<br>(23, 32)	4.7<br>(3.9, 5.5)	-21.7<br>(-32.2, -9.8)
**Trinidad and Tobago**	34<br>(30, 39)	3.8<br>(3.3, 4.3)	41<br>(32, 52)	2.3<br>(1.8, 2.9)	-40.1<br>(-51.6, -25.9)
**Virgin Islands**	4<br>(4, 5)	4.8<br>(4, 5.6)	6<br>(5, 8)	3.7<br>(2.9, 4.4)	-23.4<br>(-38.6, -8.9)
**Tropical Latin America**	6043<br>(5709, 6367)	5.7<br>(5.4, 6)	9676<br>(9169, 10175)	4<br>(3.8, 4.2)	-29.9<br>(-33, -27.2)
**Brazil**	5971<br>(5640, 6292)	5.8<br>(5.5, 6.1)	9520<br>(9007, 9997)	4.1<br>(3.8, 4.3)	-30.2<br>(-33.2, -27.4)
**Paraguay**	72<br>(58, 97)	3.1<br>(2.5, 4.1)	156<br>(119, 231)	2.8<br>(2.2, 4.1)	-7.8<br>(-28.3, 17.4)
**East Asia**	33749<br>(27528, 37632)	3.5<br>(2.9, 3.9)	37564<br>(33346, 48492)	1.8<br>(1.6, 2.4)	-47.3<br>(-54.6, -24.5)
**China**	31317<br>(25280, 35024)	3.4<br>(2.8, 3.8)	34198<br>(30212, 44510)	1.8<br>(1.6, 2.3)	-48.3<br>(-55.8, -24.3)

**North Korea**	625<br>(473, 814)	3.5<br>(2.7, 4.6)	1097<br>(783, 1457)	3.5<br>(2.5, 4.6)	-1.9<br>(-26.6, 27)
**Taiwan (province of China)**	1244<br>(1079, 1419)	7.8<br>(6.7, 8.8)	1664<br>(1431, 1921)	4.5<br>(3.9, 5.2)	-42.1<br>(-46.7, -36.3)
**Southeast Asia**	38269<br>(32943, 43413)	13.5<br>(11.5, 15.4)	61411<br>(54042, 69393)	10.2<br>(9, 11.5)	-24.5<br>(-33.1, -14.3)
**Cambodia**	1158<br>(851, 1569)	23.8<br>(17.6, 31.8)	2670<br>(2032, 3476)	23.4<br>(17.8, 30.1)	-1.7<br>(-22.5, 27.4)
**Indonesia**	24126<br>(20607, 26709)	22.8<br>(19.1, 25.4)	34444<br>(31516, 38528)	16.7<br>(15.2, 18.5)	-26.9<br>(-35.9, -14.3)
**Laos**	233<br>(160, 334)	10<br>(6.9, 14.2)	442<br>(296, 637)	9.7<br>(6.4, 13.9)	-3<br>(-33.6, 40.6)
**Malaysia**	276<br>(220, 334)	2.8<br>(2.2, 3.4)	594<br>(465, 759)	2.3<br>(1.8, 2.9)	-16.7<br>(-33.2, 1.9)
**Maldives**	3<br>(2, 4)	3<br>(2, 4.2)	5<br>(4, 7)	1.8<br>(1.3, 2.2)	-42.2<br>(-58.4, -5.2)
**Mauritius**	68<br>(53, 84)	8.2<br>(6.5, 10.1)	69<br>(55, 88)	4.1<br>(3.3, 5.1)	-49.7<br>(-55.4, -42.4)
**Myanmar**	4734<br>(3301, 6469)	17.4<br>(11.9, 23.3)	7034<br>(5293, 9041)	14.1<br>(10.7, 18)	-18.8<br>(-39.2, 14.3)
**Philippines**	1025<br>(805, 1285)	2.9<br>(2.3, 3.6)	3660<br>(2734, 4725)	4.8<br>(3.7, 6.2)	69<br>(41.6, 100.6)
**Sri Lanka**	990<br>(747, 1242)	8.2<br>(6.3, 10.1)	1223<br>(901, 1607)	5<br>(3.7, 6.5)	-38.8<br>(-50.9, -24.9)
**Seychelles**	4<br>(3, 5)	6.7<br>(5.1, 8.5)	8<br>(6, 10)	7<br>(5.5, 8.8)	4.8<br>(-10.8, 25.8)
**Thailand**	2466<br>(1981, 3021)	5.9<br>(4.8, 7.2)	4707<br>(3514, 5812)	4.8<br>(3.6, 5.9)	-19.3<br>(-33.2, -1.1)
**East Timor**	24<br>(15, 36)	6.5<br>(3.9, 10)	66<br>(36, 97)	8.2<br>(4.7, 12)	25.7<br>(-11.4, 80.5)
**Vietnam**	3112<br>(2210, 4287)	7.6<br>(5.4, 10.5)	6407<br>(4842, 8512)	6.8<br>(5.1, 9)	-11.3<br>(-38.3, 28.5)

**Oceania**	210<br>(177, 251)	5.6<br>(4.8, 6.6)	416<br>(336, 507)	5<br>(4.2, 6)	-10.5<br>(-25.6, 8.6)
**American Samoa**	1<br>(1, 1)	3.4<br>(2.7, 4.3)	1<br>(1, 1)	2.8<br>(2.4, 3.3)	-17.6<br>(-36.5, 2.6)
**Federated States of Micronesia**	3<br>(3, 5)	6.5<br>(4.9, 9.9)	3<br>(2, 5)	4.5<br>(3, 6.3)	-31<br>(-49.4, -6)
**Fiji**	9<br>(7, 11)	2<br>(1.6, 2.4)	16<br>(14, 19)	2.1<br>(1.8, 2.5)	6.2<br>(-16.5, 35.7)
**Guam**	5<br>(4, 6)	5.3<br>(4.2, 6.3)	8<br>(7, 9)	4.3<br>(3.7, 5)	-18.2<br>(-33.9, 5.7)
**Kiribati**	3<br>(2, 4)	6.1<br>(4.3, 8.8)	4<br>(3, 6)	5.5<br>(3.6, 8.4)	-9.9<br>(-28.6, 12.3)
**Marshall Islands**	1<br>(1, 2)	6.7<br>(4.7, 8.3)	2<br>(1, 3)	5.5<br>(3.6, 7.5)	-16.8<br>(-33.7, 3.9)
**Northern Mariana Islands**	1<br>(1, 2)	5.1<br>(4.1, 6.4)	2<br>(2, 2)	3.9<br>(3.4, 4.5)	-23.3<br>(-40.3, -4.8)
**Papua New Guinea**	150<br>(122, 184)	6.1<br>(5.1, 7.5)	314<br>(243, 401)	5.3<br>(4.2, 6.6)	-13<br>(-31.1, 8.8)
**Samoa**	4<br>(3, 6)	4.5<br>(3.2, 7.1)	5<br>(4, 7)	3.7<br>(2.7, 5.3)	-17.4<br>(-33.8, 2.6)
**Solomon Islands**	9<br>(7, 11)	5.3<br>(4.1, 6.5)	18<br>(14, 22)	4.6<br>(3.8, 5.5)	-12.5<br>(-30.6, 9.4)
**Tonga**	3<br>(3, 4)	5.8<br>(4.9, 6.7)	5<br>(4, 6)	5.6<br>(4.5, 6.8)	-3<br>(-21.6, 21)
**Vanuatu**	7<br>(3, 12)	8.9<br>(4.2, 14.8)	14<br>(7, 25)	7.6<br>(3.6, 13.9)	-14.2<br>(-35.8, 15.2)
**North Africa and Middle East**	16461<br>(11125, 20677)	10.2<br>(6.5, 13)	27411<br>(20485, 34220)	6.8<br>(5, 8.6)	-33.3<br>(-47.1, -17.3)
**Afghanistan**	528<br>(242, 868)	7.7<br>(3.6, 12.8)	791<br>(480, 1154)	7.2<br>(4.5, 11.1)	-7<br>(-27.8, 57.8)
**Algeria**	591<br>(386, 932)	4.9<br>(3.1, 7.8)	1154<br>(844, 1573)	3.6<br>(2.6, 4.9)	-26.5<br>(-43.2, -3)

**Bahrain**	15<br>(11, 18)	8<br>(5.9, 10.1)	34<br>(26, 42)	3.6<br>(2.7, 4.6)	-54.6<br>(-62.5, -44.8)
**Egypt**	9766<br>(5894, 12352)	43.8<br>(23.4, 56.8)	15377<br>(10243, 21268)	34.7<br>(20.8, 48.4)	-20.9<br>(-41.5, 1.1)
**Iran**	1057<br>(886, 1227)	4.1<br>(3.4, 4.8)	2341<br>(2099, 2544)	3.5<br>(3.1, 3.8)	-15.5<br>(-30.2, 1)
**Iraq**	339<br>(254, 455)	4.2<br>(3.2, 5.6)	432<br>(350, 521)	1.8<br>(1.4, 2.2)	-57.4<br>(-65.5, -46.7)
**Jordan**	69<br>(52, 93)	4.8<br>(3.5, 6.3)	170<br>(129, 211)	3.1<br>(2.3, 3.9)	-35.9<br>(-52, -12.2)
**Kuwait**	21<br>(17, 25)	2.6<br>(2.1, 3.1)	63<br>(48, 78)	2.3<br>(1.8, 2.8)	-10.9<br>(-28.9, 5.1)
**Lebanon**	107<br>(78, 138)	5<br>(3.7, 6.6)	189<br>(127, 258)	3.2<br>(2.1, 4.4)	-35.1<br>(-60.5, -12.9)
**Libya**	111<br>(77, 164)	5.8<br>(4, 8.7)	214<br>(140, 292)	4.8<br>(3.2, 6.4)	-18.3<br>(-42.2, 27.2)
**Morocco**	750<br>(518, 1096)	5.3<br>(3.5, 7.9)	1300<br>(967, 1679)	4.3<br>(3.2, 5.6)	-18.6<br>(-42.2, 15.4)
**Palestine**	40<br>(28, 55)	4.5<br>(3.2, 6.2)	85<br>(68, 104)	3.6<br>(2.8, 4.5)	-21<br>(-38.2, 10.3)
**Oman**	33<br>(23, 45)	4.6<br>(3.1, 6.2)	70<br>(47, 93)	3.3<br>(2.3, 4.4)	-26.8<br>(-47.8, -1.9)
**Qatar**	10<br>(7, 13)	8.6<br>(6.3, 11)	45<br>(32, 60)	5.1<br>(3.7, 6.7)	-41<br>(-54.4, -23.4)
**Saudi Arabia**	737<br>(300, 1049)	12.6<br>(4.7, 18)	1197<br>(751, 1585)	8.7<br>(4.8, 11.7)	-30.6<br>(-52.8, 11)
**Sudan**	777<br>(417, 1303)	8.7<br>(4.2, 15)	1133<br>(669, 1733)	6.5<br>(3.6, 10.2)	-25.4<br>(-47.2, 12.5)
**Syria**	298<br>(235, 370)	5.5<br>(4.3, 6.9)	588<br>(453, 755)	4.7<br>(3.7, 6.1)	-14.1<br>(-34.1, 9.7)
**Tunisia**	294<br>(200, 408)	6.3<br>(4.3, 8.9)	557<br>(414, 737)	4.8<br>(3.6, 6.4)	-23.2<br>(-47.4, 10.1)

**Turkey**	508<br>(337, 716)	1.4<br>(0.9, 2)	813<br>(644, 1024)	0.9<br>(0.7, 1.2)	-34.2<br>(-54.4, -5.1)
**United Arab Emirates**	30<br>(12, 47)	5.9<br>(2.2, 9.2)	172<br>(79, 245)	4.3<br>(1.8, 6.1)	-28.2<br>(-52.1, 12.1)
**Yemen**	369<br>(186, 610)	7.5<br>(3.5, 12.9)	662<br>(449, 962)	5.3<br>(3.5, 7.8)	-29.2<br>(-55.5, 23.6)
**South Asia**	31893<br>(28294, 36259)	4.9<br>(4.2, 5.5)	61624<br>(54056, 76037)	4.4<br>(3.8, 5.4)	-10.2<br>(-21.8, 10.1)
**Bangladesh**	4548<br>(3011, 5816)	7.8<br>(5.3, 9.9)	6465<br>(5053, 8326)	5.2<br>(4.1, 6.7)	-33.3<br>(-47.6, 3.5)
**Bhutan**	23<br>(17, 30)	7.7<br>(5.7, 10.3)	36<br>(24, 51)	5.5<br>(3.7, 7.7)	-29.1<br>(-50.1, -0.8)
**India**	17911<br>(15287, 22942)	3.3<br>(2.8, 4.1)	36300<br>(31201, 50637)	3.2<br>(2.7, 4.5)	-3.1<br>(-15.8, 12.8)
**Nepal**	456<br>(325, 642)	4.1<br>(2.9, 5.6)	849<br>(584, 1178)	3.8<br>(2.7, 5.2)	-6.5<br>(-32.9, 24.8)
**Pakistan**	8955<br>(5647, 11775)	14.6<br>(9.1, 19.4)	17974<br>(13057, 22963)	14.9<br>(10.6, 19.1)	2<br>(-20, 35)
**Southern sub-Saharan Africa**	1821<br>(1427, 2086)	6<br>(4.5, 6.9)	2301<br>(1881, 2720)	3.9<br>(3.2, 4.6)	-34<br>(-49.4, -12.5)
**Botswana**	41<br>(27, 59)	6.4<br>(4.3, 9.1)	67<br>(49, 94)	4.6<br>(3.5, 6.3)	-29.2<br>(-47.2, -4.1)
**eSwatini**	36<br>(26, 49)	10.5<br>(7.6, 14.5)	46<br>(33, 62)	7.3<br>(5.4, 9.9)	-31<br>(-51.4, -2.6)
**Lesotho**	63<br>(27, 98)	6<br>(2.5, 9.3)	75<br>(34, 116)	5.9<br>(2.7, 8.9)	-2.5<br>(-35.2, 52.5)
**Namibia**	33<br>(16, 50)	4.4<br>(2.1, 6.7)	75<br>(45, 116)	5<br>(3, 7.6)	12.2<br>(-27.6, 67.6)
**South Africa**	1279<br>(981, 1510)	5.4<br>(4, 6.5)	1538<br>(1377, 1717)	3.3<br>(3, 3.7)	-38.8<br>(-48, -18.8)
**Zimbabwe**	369<br>(298, 440)	8.5<br>(6.9, 10.1)	500<br>(154, 889)	7<br>(2.1, 12.2)	-17.6<br>(-76, 47.3)

**Western sub-Saharan Africa**	4225<br>(2453, 6318)	4.5<br>(2.6, 6.7)	5691<br>(3574, 8317)	2.9<br>(1.9, 4.3)	-34.7<br>(-51.4, -13.1)
**Benin**	67<br>(44, 100)	3.2<br>(2.1, 4.8)	99<br>(67, 141)	2<br>(1.3, 2.8)	-37.7<br>(-67.2, -3.7)
**Burkina Faso**	131<br>(97, 164)	3<br>(2.3, 3.8)	160<br>(12, 303)	1.8<br>(0.1, 3.3)	-40.9<br>(-96.1, 14.5)
**Cameroon**	198<br>(147, 256)	4.1<br>(3, 5.3)	334<br>(149, 514)	2.7<br>(1.2, 4.1)	-35<br>(-65.3, -6.3)
**Cape Verde**	3<br>(2, 4)	1.4<br>(1, 1.8)	8<br>(6, 9)	1.6<br>(1.3, 2)	19.8<br>(-10.3, 61.8)
**Chad**	76<br>(47, 113)	2.6<br>(1.6, 3.8)	151<br>(108, 208)	2.5<br>(1.8, 3.5)	-1.4<br>(-26.3, 44.3)
**Côte d'Ivoire**	155<br>(107, 217)	3.3<br>(2.3, 4.7)	286<br>(204, 389)	2.5<br>(1.8, 3.3)	-25.8<br>(-51.2, 0.7)
**The Gambia**	10<br>(6, 15)	2.5<br>(1.5, 3.9)	23<br>(16, 31)	2.3<br>(1.6, 3.1)	-8.5<br>(-37.3, 40.4)
**Ghana**	231<br>(176, 306)	3.4<br>(2.6, 4.4)	347<br>(113, 637)	2.1<br>(0.7, 3.9)	-37<br>(-81.8, 9.3)
**Guinea**	103<br>(61, 179)	3<br>(1.8, 5.2)	131<br>(79, 207)	2.3<br>(1.4, 3.8)	-22.6<br>(-43, 18.8)
**Guinea-Bissau**	16<br>(10, 23)	3.6<br>(2.3, 5.1)	22<br>(15, 30)	2.8<br>(2, 3.8)	-23.8<br>(-43.2, 8.7)
**Liberia**	48<br>(32, 69)	4.2<br>(2.8, 6)	53<br>(37, 74)	2.5<br>(1.8, 3.6)	-39.5<br>(-55, -21.7)
**Mali**	141<br>(86, 226)	3.4<br>(2, 5.5)	133<br>(46, 240)	1.5<br>(0.5, 2.7)	-55<br>(-88.4, -19.2)
**Mauritania**	30<br>(22, 43)	2.9<br>(2.1, 4)	35<br>(24, 51)	1.7<br>(1.2, 2.4)	-40.1<br>(-54.2, -22.9)
**Niger**	75<br>(49, 126)	2.4<br>(1.6, 4)	107<br>(27, 191)	1.4<br>(0.3, 2.4)	-43.5<br>(-81.9, -12.6)
**Nigeria**	2742<br>(1224, 4581)	5.8<br>(2.6, 9.6)	3539<br>(1458, 6248)	3.9<br>(1.6, 6.9)	-32.2<br>(-51.9, -1.9)

**São Tomé and Príncipe**	4 (2, 5)	5.7 (3.5, 7.7)	7 (5, 9)	5.9 (4.1, 8.3)	2.8 (-33.7, 56.6)
**Senegal**	71 (43, 105)	2.1 (1.2, 3.1)	113 (43, 179)	1.5 (0.6, 2.4)	-28.5 (-57.7, -5.1)
**Sierra Leone**	79 (51, 123)	3.9 (2.5, 6.1)	85 (33, 130)	2.3 (0.9, 3.5)	-41.2 (-84.5, 12.4)
**Togo**	45 (33, 63)	3.3 (2.4, 4.6)	59 (19, 93)	1.5 (0.5, 2.4)	-53.1 (-88, -15.4)
**Eastern sub-Saharan Africa**	11402 (8802, 14371)	14.5 (11.4, 18.1)	16741 (12400, 21230)	10.3 (7.6, 13.2)	-29.1 (-47.6, -5)
**Burundi**	489 (292, 753)	21.2 (13.5, 32.1)	530 (156, 900)	12.1 (3.4, 20.8)	-42.9 (-84.5, -6.2)
**Comoros**	14 (2, 25)	6.7 (0.8, 12.2)	28 (6, 48)	6.2 (1.2, 10.5)	-7 (-29.4, 48.6)
**Djibouti**	14 (8, 21)	8.2 (5.2, 12.5)	44 (26, 67)	7.5 (4.4, 11.3)	-8.4 (-37.2, 32.2)
**Eritrea**	158 (91, 257)	15.1 (9.4, 24.3)	352 (249, 498)	14.2 (10.1, 19.4)	-6.2 (-34.8, 45.1)
**Ethiopia**	3749 (2335, 5757)	18.6 (12, 27)	5444 (4577, 6565)	13.4 (11.3, 16.2)	-27.9 (-52, 19.2)
**Kenya**	654 (402, 966)	7.6 (4.7, 11.1)	1409 (919, 2047)	6.4 (4.2, 9.3)	-15.8 (-48.9, 10.9)
**Madagascar**	595 (388, 908)	11 (7.2, 17.1)	929 (643, 1286)	8.4 (5.7, 11.5)	-23.8 (-58.4, 21.5)
**Malawi**	687 (388, 972)	16.6 (10, 23)	805 (159, 1365)	10.4 (2, 17.6)	-37.1 (-88.5, 25.3)
**Mozambique**	516 (315, 768)	8.3 (5, 12.3)	653 (101, 1140)	5.8 (0.9, 9.9)	-30.4 (-89.4, 43.5)
**Rwanda**	1236 (958, 1508)	41.1 (32.5, 49.5)	1256 (790, 1795)	21.5 (13.3, 30.4)	-47.7 (-69.2, -22.9)
**Somalia**	296 (134, 533)	10.9 (5.6, 18.9)	773 (450, 1245)	11.9 (7, 19.2)	9.3 (-24.4, 71.4)

**South Sudan**	245<br>(131, 428)	10.1<br>(5.7, 17.1)	410<br>(252, 627)	10.5<br>(6.6, 15.9)	4.1<br>(-28.2, 62.7)
**Tanzania**	1316<br>(914, 1767)	11.8<br>(8.4, 15.7)	1823<br>(696, 2903)	7.4<br>(2.8, 11.9)	-36.6<br>(-75.5, 0)
**Uganda**	780<br>(458, 1190)	11.5<br>(6.7, 17.5)	1272<br>(490, 2005)	8.9<br>(3.3, 14.1)	-22.5<br>(-57.7, 9.8)
**Zambia**	648<br>(458, 858)	21.4<br>(15.3, 28.5)	1001<br>(747, 1277)	14.9<br>(11, 19.1)	-30.3<br>(-45.3, -11.6)
**Central sub-Saharan Africa**	3221<br>(2339, 4372)	12.7<br>(9.1, 17.6)	6579<br>(5026, 8571)	11.3<br>(8.4, 15.2)	-11.1<br>(-26.9, 11.1)
**Angola**	642<br>(342, 1038)	14.6<br>(7.6, 24.2)	1698<br>(1239, 2368)	14<br>(10.2, 20.4)	-3.6<br>(-34.2, 59.1)
**Central African Republic**	169<br>(94, 246)	12.3<br>(6.8, 17.6)	226<br>(96, 375)	9<br>(3.7, 14.6)	-27.1<br>(-50.8, -3.2)
**Congo**	187<br>(125, 283)	15.6<br>(10.4, 23.7)	334<br>(218, 486)	12.1<br>(7.6, 17.9)	-22.5<br>(-42.2, 6.1)
**DR Congo**	2089<br>(1497, 2899)	11.9<br>(8.6, 16.5)	4134<br>(2954, 5678)	10.6<br>(7.5, 14.5)	-11.4<br>(-32.5, 12.7)
**Equatorial Guinea**	24<br>(8, 42)	11<br>(3.4, 18.7)	46<br>(25, 74)	8.5<br>(4.5, 13.8)	-22.5<br>(-51.7, 64.6)
**Gabon**	110<br>(72, 154)	18.5<br>(12.2, 26.9)	142<br>(95, 211)	12.8<br>(8.3, 19.4)	-31.2<br>(-46.7, -11.7)

Appendix Table 13: Number of deaths and age-standardised death rates of cirrhosis due to alcohol-related liver disease per 100 000 in 1990 and 2017 for both sexes and the percentage change by location

	1990		2017		Percentage change in age-standardised rates between 1990 and 2017
	Counts (95% UI)	Rate (95% UI)	Counts (95% UI)	Rate (95% UI)	
**Global**	215190 (194899, 234586)	5.1 (4.6, 5.5)	332268 (302998, 373279)	4.1 (3.7, 4.6)	-19.4 (-25, -10.7)
**High-income North America**	10978 (10403, 11511)	3.3 (3.1, 3.4)	18972 (17896, 20101)	3.4 (3.2, 3.6)	2.3 (-1.9, 7.1)
**Canada**	959 (881, 1036)	3 (2.7, 3.2)	1540 (1380, 1712)	2.4 (2.2, 2.7)	-17.5 (-24.8, -9.6)
**Greenland**	1 (1, 2)	2.9 (2.4, 3.4)	2 (2, 3)	2.9 (2.1, 3.4)	1.6 (-28.3, 29.9)
**USA**	10017 (9488, 10505)	3.3 (3.2, 3.5)	17430 (16416, 18494)	3.5 (3.3, 3.7)	4.3 (-0.1, 9.6)
**Australasia**	451 (412, 487)	2 (1.8, 2.1)	700 (612, 799)	1.6 (1.4, 1.8)	-20.3 (-29.1, -10.2)
**Australia**	396 (358, 430)	2.1 (1.9, 2.2)	621 (537, 718)	1.6 (1.4, 1.9)	-19.9 (-29.8, -8.3)
**New Zealand**	55 (50, 59)	1.4 (1.3, 1.5)	79 (72, 88)	1.1 (1, 1.2)	-23.9 (-30.7, -16.4)
**High-income Asia Pacific**	9652 (8791, 10428)	4.6 (4.2, 5)	7754 (6852, 8547)	2.1 (1.8, 2.4)	-54.5 (-59.3, -49.5)
**Brunei**	3 (2, 5)	2.7 (2, 3.5)	6 (4, 8)	1.6 (1.1, 2.2)	-39.6 (-51.8, -17)
**Japan**	3671 (3417, 3923)	2.1 (2, 2.3)	3842 (3504, 4169)	1.2 (1.1, 1.3)	-43 (-46.7, -39.8)
**Singapore**	31 (26, 37)	1.2 (1, 1.5)	42 (34, 51)	0.6 (0.5, 0.7)	-52.5 (-58.2, -46.8)
**South Korea**	5946 (5242, 6567)	16.9 (15, 18.5)	3864 (3214, 4483)	4.5 (3.8, 5.2)	-73.2 (-77, -68.8)

**Western Europe**	35118<br>(30850, 39220)	6.5<br>(5.7, 7.2)	30953<br>(26859, 35057)	4<br>(3.4, 4.4)	-39.1<br>(-42.5, -35.3)
**Andorra**	3<br>(2, 4)	5.1<br>(3.6, 6.7)	5<br>(3, 6)	3.5<br>(2.4, 4.6)	-32.3<br>(-50.3, -9.8)
**Austria**	1126<br>(953, 1282)	10.6<br>(8.9, 12)	878<br>(710, 1028)	5.6<br>(4.5, 6.5)	-46.9<br>(-52, -41.3)
**Belgium**	902<br>(815, 988)	6.2<br>(5.7, 6.8)	1073<br>(944, 1207)	5.3<br>(4.6, 6)	-14.8<br>(-23, -6.6)
**Cyprus**	38<br>(28, 51)	4.7<br>(3.5, 6.3)	52<br>(42, 64)	2.8<br>(2.2, 3.5)	-39.7<br>(-54.4, -20.2)
**Denmark**	365<br>(312, 417)	5.3<br>(4.5, 6.1)	448<br>(368, 524)	4.6<br>(3.7, 5.3)	-14.4<br>(-24.4, -2.7)
**Finland**	259<br>(217, 302)	3.9<br>(3.3, 4.6)	532<br>(428, 634)	5.8<br>(4.6, 6.9)	47<br>(28.7, 66.9)
**France**	6596<br>(5647, 7536)	8.6<br>(7.4, 9.8)	4876<br>(4003, 5731)	4.3<br>(3.5, 5)	-50.6<br>(-56.6, -44.9)
**Germany**	9797<br>(8359, 11159)	8.3<br>(7.1, 9.4)	8893<br>(7237, 10713)	5.6<br>(4.5, 6.7)	-33.2<br>(-41.6, -23.6)
**Greece**	690<br>(572, 810)	4.5<br>(3.8, 5.3)	543<br>(442, 644)	2.6<br>(2.1, 3)	-43.1<br>(-48.9, -36.3)
**Iceland**	5<br>(4, 6)	1.8<br>(1.5, 2)	8<br>(6, 9)	1.5<br>(1.3, 1.8)	-12.8<br>(-22.8, -1.4)
**Ireland**	81<br>(67, 95)	2<br>(1.7, 2.4)	173<br>(140, 206)	2.5<br>(2, 3)	23.4<br>(6.8, 39.7)
**Israel**	135<br>(108, 163)	2.8<br>(2.3, 3.4)	233<br>(184, 287)	2.1<br>(1.7, 2.6)	-24.8<br>(-32.1, -15.9)
**Italy**	3887<br>(3403, 4390)	4.4<br>(3.9, 5)	2174<br>(1882, 2493)	1.6<br>(1.4, 1.8)	-63.9<br>(-68.2, -59.3)
**Luxembourg**	42<br>(36, 49)	8<br>(6.7, 9.3)	46<br>(36, 56)	5<br>(3.9, 6.1)	-37.3<br>(-48.2, -25.5)
**Malta**	15<br>(12, 17)	3.4<br>(2.8, 4)	18<br>(14, 21)	2.3<br>(1.9, 2.7)	-33.1<br>(-40.6, -24)

**Netherlands**	572<br>(482, 670)	2.9<br>(2.5, 3.4)	673<br>(553, 803)	2.1<br>(1.7, 2.5)	-28.6<br>(-34.8, -21.3)
**Norway**	147<br>(134, 161)	2.5<br>(2.3, 2.7)	157<br>(139, 175)	1.8<br>(1.6, 2)	-27.5<br>(-35.2, -21.6)
**Portugal**	1701<br>(1441, 1955)	12.8<br>(10.9, 14.6)	937<br>(768, 1131)	4.7<br>(3.8, 5.7)	-63.1<br>(-67.4, -58.5)
**Spain**	5882<br>(5094, 6563)	11<br>(9.6, 12.1)	4245<br>(3540, 4888)	4.7<br>(4, 5.4)	-56.9<br>(-60.9, -52.2)
**Sweden**	380<br>(351, 408)	2.9<br>(2.7, 3.1)	449<br>(401, 498)	2.4<br>(2.2, 2.7)	-16<br>(-22.9, -8.5)
**Switzerland**	381<br>(320, 440)	3.9<br>(3.3, 4.5)	406<br>(327, 484)	2.6<br>(2.1, 3.1)	-33.9<br>(-42.4, -24)
**UK**	2082<br>(1945, 2207)	2.6<br>(2.5, 2.8)	4104<br>(3837, 4344)	4<br>(3.8, 4.3)	52.1<br>(46.7, 61)
**Southern Latin America**	3548<br>(3203, 3910)	7.5<br>(6.8, 8.2)	4437<br>(3848, 5028)	5.5<br>(4.8, 6.3)	-25.8<br>(-33.4, -18)
**Argentina**	2067<br>(1870, 2273)	6.3<br>(5.7, 6.9)	2612<br>(2191, 3030)	5<br>(4.2, 5.9)	-19.8<br>(-31.3, -8.6)
**Chile**	1324<br>(1170, 1482)	12.3<br>(10.8, 13.7)	1678<br>(1421, 1943)	7.2<br>(6.1, 8.3)	-41.1<br>(-48.8, -32.7)
**Uruguay**	157<br>(140, 177)	4.1<br>(3.7, 4.6)	147<br>(125, 172)	2.9<br>(2.4, 3.4)	-29.7<br>(-39.4, -19.2)
**Eastern Europe**	10818<br>(10013, 11611)	3.8<br>(3.5, 4.1)	29236<br>(27406, 31119)	9.6<br>(9, 10.2)	151.5<br>(141.2, 163.3)
**Belarus**	334<br>(293, 377)	2.5<br>(2.2, 2.8)	1027<br>(867, 1183)	7.1<br>(6, 8.1)	177.6<br>(130.2, 222)
**Estonia**	53<br>(47, 60)	2.6<br>(2.4, 3)	126<br>(105, 151)	6.2<br>(5.2, 7.5)	136.7<br>(95.1, 180.8)
**Latvia**	92<br>(82, 104)	2.6<br>(2.3, 2.9)	174<br>(148, 201)	5.7<br>(4.8, 6.5)	120.6<br>(89.1, 153.6)
**Lithuania**	135<br>(122, 150)	3<br>(2.7, 3.3)	393<br>(349, 439)	9.1<br>(8, 10.1)	202.9<br>(174.6, 235.1)

**Moldova**	1134<br>(1013, 1260)	23.9<br>(21.5, 26.4)	1097<br>(956, 1222)	19.7<br>(17.3, 21.8)	-17.6<br>(-24.4, -11.3)
**Russia**	6040<br>(5422, 6548)	3.3<br>(3, 3.6)	18683<br>(17546, 19773)	8.9<br>(8.4, 9.4)	169.7<br>(153.9, 195.1)
**Ukraine**	3031<br>(2822, 3254)	4.3<br>(4, 4.6)	7735<br>(6977, 8500)	12.1<br>(10.9, 13.3)	182.8<br>(152.9, 210)
**Central Europe**	13005<br>(12183, 13917)	8.6<br>(8.1, 9.2)	13626<br>(12563, 14656)	7.1<br>(6.5, 7.6)	-18<br>(-21.6, -14.4)
**Albania**	79<br>(65, 89)	3.7<br>(2.9, 4.2)	113<br>(89, 152)	2.8<br>(2.2, 3.7)	-25.1<br>(-42.6, 11.6)
**Bosnia and Herzegovina**	264<br>(237, 306)	5.9<br>(5.3, 6.6)	209<br>(175, 297)	3.6<br>(3, 5.1)	-39.1<br>(-47.7, -20.7)
**Bulgaria**	802<br>(737, 866)	6.3<br>(5.8, 6.8)	894<br>(795, 988)	7.1<br>(6.3, 7.8)	12<br>(0.6, 24.5)
**Croatia**	746<br>(688, 806)	11.3<br>(10.5, 12.2)	471<br>(420, 526)	5.9<br>(5.3, 6.6)	-47.8<br>(-52.3, -42.9)
**Czech Republic**	957<br>(888, 1037)	7.1<br>(6.6, 7.7)	1001<br>(899, 1110)	5.5<br>(5, 6.1)	-23<br>(-30.8, -14.6)
**Hungary**	2669<br>(2495, 2842)	19<br>(17.8, 20.2)	1403<br>(1258, 1557)	8.3<br>(7.4, 9.2)	-56.6<br>(-60.4, -52.4)
**Montenegro**	14<br>(12, 17)	2.2<br>(1.8, 2.5)	20<br>(17, 24)	2.1<br>(1.8, 2.5)	-3.9<br>(-23, 15.1)
**North Macedonia**	56<br>(49, 68)	2.8<br>(2.5, 3.4)	89<br>(73, 103)	2.7<br>(2.2, 3.1)	-6.3<br>(-23.9, 10.1)
**Poland**	2076<br>(1911, 2266)	4.6<br>(4.2, 5)	3514<br>(3176, 3874)	5.7<br>(5.2, 6.3)	24.4<br>(12.1, 38)
**Romania**	3670<br>(3398, 3955)	12.7<br>(11.8, 13.7)	4449<br>(4017, 4883)	13.2<br>(11.9, 14.4)	3.4<br>(-4.1, 11.9)
**Serbia**	587<br>(461, 699)	4.8<br>(3.8, 5.7)	541<br>(473, 633)	3.7<br>(3.2, 4.2)	-24.4<br>(-38, -4.9)
**Slovakia**	740<br>(650, 919)	12.5<br>(11, 15.4)	692<br>(561, 794)	8.1<br>(6.5, 9.2)	-35.4<br>(-53.4, -22.2)

**Slovenia**	345<br>(317, 370)	13.7<br>(12.6, 14.7)	231<br>(204, 258)	6.1<br>(5.4, 6.8)	-55.7<br>(-60, -50.9)
**Central Asia**	4469<br>(4038, 4918)	9<br>(8.2, 9.9)	11285<br>(9912, 12533)	14.2<br>(12.5, 15.7)	57<br>(43.9, 68.1)
**Armenia**	145<br>(128, 164)	5.1<br>(4.5, 5.8)	386<br>(343, 434)	9.5<br>(8.4, 10.5)	84.3<br>(67.9, 101.7)
**Azerbaijan**	477<br>(417, 536)	9.2<br>(7.9, 10.3)	1116<br>(893, 1361)	12.6<br>(9.9, 15.5)	37.8<br>(15.2, 68.5)
**Georgia**	552<br>(498, 616)	8.7<br>(7.8, 9.7)	532<br>(464, 619)	9.9<br>(8.6, 11.4)	13.2<br>(1, 27.1)
**Kazakhstan**	805<br>(717, 900)	6<br>(5.4, 6.7)	2708<br>(2179, 3148)	15.1<br>(12.3, 17.6)	151.5<br>(106.8, 187.8)
**Kyrgyzstan**	363<br>(325, 404)	11.6<br>(10.3, 12.8)	777<br>(652, 881)	15.4<br>(13.1, 17.4)	32.8<br>(16.7, 47.1)
**Mongolia**	226<br>(192, 266)	20.7<br>(17.9, 24)	503<br>(426, 582)	22.2<br>(17.9, 25.8)	7.2<br>(-15.5, 26)
**Tajikistan**	254<br>(219, 330)	8.5<br>(7.4, 11.1)	517<br>(441, 610)	8.8<br>(7.6, 10.6)	3.5<br>(-8.7, 17.4)
**Turkmenistan**	274<br>(246, 305)	13.2<br>(11.8, 14.6)	839<br>(742, 957)	18.5<br>(16.5, 21)	40.8<br>(26, 56.3)
**Uzbekistan**	1372<br>(1222, 1522)	11.2<br>(10, 12.5)	3906<br>(3350, 4518)	15.6<br>(13.5, 17.8)	39<br>(22.5, 57)
**Central Latin America**	11864<br>(11295, 12399)	12.6<br>(12, 13.2)	21602<br>(20333, 23081)	9.1<br>(8.5, 9.7)	-28.1<br>(-31.7, -24.1)
**Colombia**	784<br>(727, 841)	4.3<br>(4, 4.6)	1559<br>(1351, 1800)	2.9<br>(2.5, 3.3)	-33.2<br>(-41, -23.3)
**Costa Rica**	130<br>(121, 139)	7<br>(6.5, 7.5)	344<br>(302, 391)	6.9<br>(6.1, 7.9)	-1.6<br>(-12.3, 11.1)
**El Salvador**	324<br>(279, 354)	10.1<br>(8.7, 11.1)	544<br>(434, 722)	9.6<br>(7.6, 12.7)	-5.6<br>(-25.3, 31)
**Guatemala**	667<br>(615, 718)	15.3<br>(14, 16.5)	1542<br>(1353, 1752)	13.2<br>(11.6, 15)	-13.5<br>(-24.7, -1.1)

**Honduras**	292<br>(237, 413)	12.6<br>(10.2, 17.3)	785<br>(568, 1085)	12.5<br>(9, 17.3)	-0.3<br>(-23, 25.1)
**Mexico**	8620<br>(8224, 8981)	17.7<br>(16.9, 18.5)	14811<br>(13974, 15717)	12.6<br>(11.9, 13.4)	-29.1<br>(-32.4, -25.4)
**Nicaragua**	151<br>(133, 169)	8.6<br>(7.5, 9.6)	402<br>(289, 479)	8.4<br>(6, 9.9)	-2.6<br>(-25.2, 15)
**Panama**	75<br>(68, 82)	4.9<br>(4.5, 5.4)	173<br>(156, 192)	4.4<br>(3.9, 4.8)	-11.9<br>(-20.9, -1.8)
**Venezuela**	820<br>(763, 880)	7.8<br>(7.2, 8.4)	1444<br>(1203, 1733)	5<br>(4.2, 5.9)	-36.3<br>(-47.3, -24.1)
**Andean Latin America**	2297<br>(2046, 2637)	10.4<br>(9.3, 12)	4283<br>(3745, 4838)	7.9<br>(6.9, 8.9)	-24.3<br>(-34.5, -13.3)
**Bolivia**	392<br>(280, 542)	11.6<br>(8.5, 15.9)	819<br>(617, 1076)	9.5<br>(7.2, 12.5)	-18<br>(-37.7, 11.4)
**Ecuador**	468<br>(427, 511)	8<br>(7.3, 8.8)	1140<br>(991, 1304)	7.7<br>(6.7, 8.8)	-3.8<br>(-14.7, 8.2)
**Peru**	1438<br>(1262, 1652)	11.2<br>(9.8, 13)	2325<br>(1905, 2771)	7.6<br>(6.2, 9)	-32.4<br>(-45.3, -18.2)
**Caribbean**	1841<br>(1595, 2091)	6.9<br>(5.9, 7.8)	2541<br>(2160, 3086)	5<br>(4.2, 6.1)	-27.4<br>(-35, -9.5)
**Antigua and Barbuda**	3<br>(2, 3)	5.6<br>(4.8, 6.3)	4<br>(3, 4)	3.6<br>(3.1, 4.2)	-35.2<br>(-42.6, -25.8)
**The Bahamas**	15<br>(13, 17)	8.7<br>(7.7, 9.8)	20<br>(17, 23)	4.9<br>(4.2, 5.7)	-43.8<br>(-51.7, -35.2)
**Barbados**	12<br>(11, 14)	4.4<br>(3.9, 4.9)	13<br>(11, 15)	2.8<br>(2.4, 3.3)	-35.4<br>(-43.6, -26.1)
**Belize**	7<br>(6, 8)	7.2<br>(6.2, 8.2)	20<br>(17, 22)	7<br>(6.1, 8)	-2.5<br>(-13.4, 9.3)
**Bermuda**	3<br>(3, 4)	5.4<br>(4.7, 6.2)	3<br>(2, 3)	2.1<br>(1.8, 2.5)	-61.4<br>(-67.1, -52.5)
**Cuba**	354<br>(315, 399)	3.4<br>(3, 3.8)	649<br>(535, 775)	3.6<br>(3, 4.3)	5.6<br>(-8.3, 21.5)

**Dominica**	3 (3, 4)	4.9 (4.4, 5.6)	3 (3, 4)	3.8 (3.3, 4.3)	-22.7 (-31.7, -12.9)
**Dominican Republic**	455 (305, 536)	11.6 (7.6, 13.7)	571 (425, 956)	6.2 (4.6, 10.2)	-46.8 (-61.7, 20.8)
**Grenada**	5 (5, 6)	7.9 (7, 9)	7 (6, 7)	4.7 (4, 5.3)	-41.2 (-47.8, -34)
**Guyana**	61 (55, 68)	14.1 (12.6, 15.7)	62 (52, 75)	9.3 (7.9, 11.1)	-33.9 (-43.6, -22.2)
**Haiti**	342 (229, 510)	9.7 (6.6, 15)	581 (404, 901)	8.2 (5.7, 12.8)	-15.9 (-34.9, 11.1)
**Jamaica**	50 (43, 57)	2.8 (2.5, 3.2)	62 (48, 81)	2.1 (1.6, 2.8)	-25.5 (-42, 3.1)
**Puerto Rico**	373 (332, 414)	10.2 (9.1, 11.3)	325 (283, 369)	5.1 (4.5, 5.8)	-49.7 (-54.4, -43.9)
**Saint Lucia**	8 (7, 9)	8.7 (7.8, 9.7)	10 (8, 11)	4.5 (3.9, 5.2)	-48.6 (-54.1, -41.6)
**Saint Vincent and the Grenadines**	4 (4, 5)	5.6 (4.8, 6.3)	6 (5, 7)	4.2 (3.7, 5)	-24.5 (-34.4, -12.6)
**Suriname**	24 (21, 27)	8.9 (7.7, 10.1)	43 (37, 51)	7.3 (6.2, 8.5)	-18 (-29.7, -4.2)
**Trinidad and Tobago**	50 (44, 56)	5.6 (5, 6.3)	60 (47, 76)	3.3 (2.6, 4.2)	-41.3 (-52.9, -27.8)
**Virgin Islands**	7 (6, 9)	8 (6.9, 9.3)	11 (9, 14)	6.2 (4.9, 7.6)	-22.2 (-38.3, -6.7)
**Tropical Latin America**	5030 (4760, 5319)	4.8 (4.5, 5.1)	8164 (7657, 8651)	3.4 (3.2, 3.6)	-29.6 (-32.6, -26.6)
**Brazil**	4946 (4677, 5238)	4.8 (4.6, 5.1)	7984 (7488, 8468)	3.4 (3.2, 3.6)	-29.9 (-32.9, -26.9)
**Paraguay**	84 (69, 119)	3.6 (3, 5)	180 (135, 276)	3.3 (2.5, 5)	-10.3 (-31.9, 16.7)
**East Asia**	25807 (20853, 28549)	2.7 (2.2, 2.9)	30306 (27061, 39325)	1.5 (1.3, 1.9)	-45 (-52.2, -22)

**China**	24055<br>(19228, 26604)	2.6<br>(2.1, 2.9)	27846<br>(24769, 36498)	1.4<br>(1.3, 1.9)	-45.9<br>(-53.5, -21.7)
**North Korea**	453<br>(350, 585)	2.5<br>(2, 3.2)	783<br>(571, 1070)	2.4<br>(1.8, 3.3)	-2.9<br>(-28.4, 28.4)
**Taiwan (province of China)**	869<br>(779, 972)	5.4<br>(4.8, 6)	1189<br>(1046, 1344)	3.2<br>(2.8, 3.6)	-40.4<br>(-45.1, -34.5)
**Southeast Asia**	14775<br>(12078, 17669)	5.3<br>(4.4, 6.3)	26282<br>(21815, 31232)	4.3<br>(3.6, 5.1)	-18.7<br>(-28.4, -7.1)
**Cambodia**	577<br>(414, 791)	12<br>(8.8, 16.1)	1496<br>(1118, 2007)	13.1<br>(9.9, 17.3)	8.5<br>(-14.5, 41)
**Indonesia**	6691<br>(5477, 7806)	6.5<br>(5.3, 7.6)	9763<br>(8533, 11353)	4.8<br>(4.1, 5.5)	-27<br>(-36.4, -13.6)
**Laos**	154<br>(103, 214)	6.7<br>(4.6, 9.3)	287<br>(189, 421)	6.3<br>(4.2, 9.2)	-5.4<br>(-35.1, 35.9)
**Malaysia**	260<br>(204, 321)	2.6<br>(2, 3.2)	554<br>(426, 743)	2.1<br>(1.6, 2.9)	-18.5<br>(-34.8, -0.6)
**Maldives**	2<br>(1, 2)	1.8<br>(1.1, 2.5)	3<br>(2, 4)	1<br>(0.7, 1.3)	-42.2<br>(-57.9, -4.5)
**Mauritius**	46<br>(36, 57)	5.5<br>(4.3, 6.9)	45<br>(35, 57)	2.7<br>(2.1, 3.3)	-51.8<br>(-57.3, -44.8)
**Myanmar**	2428<br>(1604, 3368)	9<br>(6, 12.5)	3708<br>(2747, 4887)	7.4<br>(5.6, 9.6)	-17.7<br>(-39.5, 18.3)
**Philippines**	680<br>(532, 844)	1.9<br>(1.5, 2.4)	2378<br>(1797, 3094)	3.1<br>(2.4, 4)	62.5<br>(35.6, 94.2)
**Sri Lanka**	556<br>(415, 705)	4.6<br>(3.6, 5.7)	714<br>(527, 948)	2.9<br>(2.2, 3.8)	-37.3<br>(-50.1, -22.1)
**Seychelles**	2<br>(2, 3)	4<br>(3.1, 5.2)	5<br>(4, 6)	4.2<br>(3.3, 5.3)	5.4<br>(-11.3, 27.9)
**Thailand**	1531<br>(1179, 1927)	3.7<br>(2.9, 4.7)	3029<br>(2244, 3840)	3<br>(2.3, 3.8)	-18.5<br>(-32.8, -0.4)
**East Timor**	12<br>(7, 18)	3.5<br>(2.1, 5.4)	35<br>(20, 53)	4.4<br>(2.5, 6.6)	26.2<br>(-9.2, 80.4)

**Vietnam**	1816<br>(1290, 2464)	4.5<br>(3.2, 6.1)	4230<br>(3132, 5739)	4.4<br>(3.3, 6)	-0.9<br>(-31.3, 44.6)
**Oceania**	142<br>(117, 170)	3.9<br>(3.2, 4.6)	279<br>(225, 344)	3.4<br>(2.8, 4.1)	-11.8<br>(-28.1, 7.3)
**American Samoa**	1<br>(0, 1)	2.4<br>(1.9, 3)	1<br>(1, 1)	1.9<br>(1.6, 2.3)	-21.4<br>(-39.5, -0.5)
**Federated States of Micronesia**	2<br>(2, 4)	4.3<br>(3.2, 6.7)	2<br>(1, 3)	2.9<br>(1.9, 4.1)	-33.9<br>(-52.1, -7.6)
**Fiji**	5<br>(4, 7)	1.3<br>(1, 1.5)	10<br>(8, 13)	1.3<br>(1.1, 1.6)	6.3<br>(-17.8, 37.3)
**Guam**	3<br>(3, 4)	4<br>(3.1, 4.8)	6<br>(5, 7)	3.2<br>(2.8, 3.8)	-18.8<br>(-34.6, 6.1)
**Kiribati**	2<br>(1, 2)	3.8<br>(2.7, 5.5)	3<br>(2, 4)	3.4<br>(2.2, 5.1)	-11.7<br>(-30.5, 11.6)
**Marshall Islands**	1<br>(1, 1)	4.5<br>(3.1, 5.7)	1<br>(1, 2)	3.8<br>(2.4, 5.1)	-16.8<br>(-34.4, 3.8)
**Northern Mariana Islands**	1<br>(1, 1)	3.6<br>(2.9, 4.6)	1<br>(1, 2)	2.7<br>(2.3, 3.2)	-24.5<br>(-42.4, -6.3)
**Papua New Guinea**	102<br>(82, 127)	4.3<br>(3.6, 5.3)	212<br>(161, 272)	3.7<br>(2.9, 4.6)	-14.5<br>(-34, 8.2)
**Samoa**	3<br>(2, 4)	3.1<br>(2.2, 4.9)	3<br>(2, 5)	2.5<br>(1.8, 3.6)	-18.6<br>(-35.9, 1)
**Solomon Islands**	6<br>(4, 7)	3.5<br>(2.7, 4.4)	12<br>(9, 14)	3.1<br>(2.5, 3.8)	-12.6<br>(-31.2, 9.3)
**Tonga**	2<br>(2, 3)	3.8<br>(3.1, 4.5)	3<br>(2, 4)	3.7<br>(2.9, 4.6)	-1.9<br>(-20.9, 23.7)
**Vanuatu**	5<br>(2, 8)	5.7<br>(2.7, 10.1)	9<br>(4, 16)	4.8<br>(2.3, 9.2)	-15.9<br>(-37, 14)
**North Africa and Middle East**	2414<br>(1689, 3008)	1.5<br>(1, 1.9)	4131<br>(3207, 5039)	1<br>(0.8, 1.2)	-31.4<br>(-46.3, -15.4)
**Afghanistan**	84<br>(39, 134)	1.2<br>(0.6, 2)	120<br>(74, 174)	1.1<br>(0.7, 1.7)	-8.5<br>(-30, 59.7)

**Algeria**	91<br>(58, 145)	0.8<br>(0.5, 1.2)	183<br>(133, 251)	0.6<br>(0.4, 0.8)	-25.3<br>(-42.5, -2.4)
**Bahrain**	3<br>(2, 3)	1.4<br>(1.1, 1.8)	6<br>(5, 8)	0.6<br>(0.5, 0.8)	-56.9<br>(-64.7, -46.5)
**Egypt**	1302<br>(805, 1586)	5.8<br>(3.2, 7.1)	2132<br>(1476, 2953)	4.6<br>(2.9, 6.4)	-19.3<br>(-40.9, 5.4)
**Iran**	131<br>(107, 154)	0.5<br>(0.4, 0.6)	291<br>(259, 320)	0.4<br>(0.4, 0.5)	-16.3<br>(-31.1, 1.8)
**Iraq**	54<br>(41, 71)	0.7<br>(0.5, 0.9)	69<br>(57, 84)	0.3<br>(0.2, 0.3)	-57.4<br>(-66, -45)
**Jordan**	11<br>(8, 14)	0.7<br>(0.5, 1)	27<br>(21, 34)	0.5<br>(0.4, 0.6)	-33.8<br>(-49.8, -7)
**Kuwait**	3<br>(3, 4)	0.4<br>(0.4, 0.5)	11<br>(8, 13)	0.4<br>(0.3, 0.5)	-9.2<br>(-27.2, 7.7)
**Lebanon**	18<br>(14, 24)	0.9<br>(0.6, 1.1)	31<br>(21, 41)	0.5<br>(0.4, 0.7)	-38.6<br>(-63.2, -17.6)
**Libya**	18<br>(12, 26)	0.9<br>(0.6, 1.4)	34<br>(22, 47)	0.7<br>(0.5, 1)	-18.8<br>(-44, 26.5)
**Morocco**	113<br>(77, 167)	0.8<br>(0.5, 1.2)	197<br>(152, 257)	0.6<br>(0.5, 0.8)	-18.9<br>(-42.8, 16.8)
**Palestine**	6<br>(4, 8)	0.7<br>(0.5, 1)	13<br>(11, 16)	0.6<br>(0.4, 0.7)	-20.8<br>(-39.1, 13.2)
**Oman**	5<br>(3, 7)	0.7<br>(0.5, 1)	12<br>(8, 16)	0.6<br>(0.4, 0.7)	-23.3<br>(-46.1, 5)
**Qatar**	2<br>(1, 2)	1.4<br>(1, 1.9)	8<br>(6, 11)	0.9<br>(0.6, 1.1)	-39.5<br>(-54.2, -20.6)
**Saudi Arabia**	93<br>(37, 135)	1.6<br>(0.6, 2.3)	156<br>(98, 209)	1.1<br>(0.6, 1.5)	-29<br>(-52.3, 12.7)
**Sudan**	132<br>(67, 225)	1.5<br>(0.7, 2.6)	199<br>(111, 314)	1.1<br>(0.6, 1.9)	-22.6<br>(-45.9, 18)
**Syria**	45<br>(35, 56)	0.8<br>(0.7, 1.1)	93<br>(71, 119)	0.7<br>(0.6, 1)	-11<br>(-31.1, 15.3)

**Tunisia**	38<br>(25, 54)	0.8<br>(0.5, 1.1)	72<br>(51, 97)	0.6<br>(0.4, 0.8)	-22.1<br>(-47.4, 13.7)
**Turkey**	199<br>(133, 274)	0.6<br>(0.4, 0.8)	336<br>(277, 407)	0.4<br>(0.3, 0.5)	-30.5<br>(-51.9, 1.6)
**United Arab Emirates**	6<br>(2, 9)	1.2<br>(0.4, 1.8)	33<br>(16, 47)	0.8<br>(0.3, 1.1)	-32.7<br>(-56.5, 6.1)
**Yemen**	59<br>(28, 101)	1.2<br>(0.5, 2.1)	105<br>(69, 156)	0.8<br>(0.6, 1.3)	-28.6<br>(-54.7, 27.2)
**South Asia**	41714<br>(35585, 49680)	6.2<br>(5.4, 7.3)	86765<br>(75288, 114808)	6.2<br>(5.3, 8.2)	-1.5<br>(-12.7, 17)
**Bangladesh**	4802<br>(3286, 6215)	8.7<br>(5.9, 11.3)	7302<br>(5486, 9386)	6<br>(4.5, 7.7)	-31.7<br>(-46.3, 5.7)
**Bhutan**	29<br>(20, 40)	10.3<br>(7.3, 13.9)	47<br>(31, 67)	7.3<br>(5, 10.4)	-29.2<br>(-50.5, 1.5)
**India**	33225<br>(28115, 42426)	6.1<br>(5.2, 7.7)	72040<br>(62060, 99290)	6.3<br>(5.4, 8.7)	2.1<br>(-11.4, 19.3)
**Nepal**	1279<br>(935, 1706)	11.6<br>(8.5, 15.5)	2650<br>(1905, 3511)	12<br>(8.7, 15.9)	2.9<br>(-25.7, 38.4)
**Pakistan**	2379<br>(1423, 3329)	4<br>(2.4, 5.6)	4725<br>(3250, 6349)	4.1<br>(2.8, 5.5)	2.3<br>(-19.7, 37.6)
**Southern sub-Saharan Africa**	1132<br>(877, 1309)	3.7<br>(2.8, 4.3)	1335<br>(1125, 1565)	2.3<br>(1.9, 2.7)	-38.7<br>(-51.9, -19.4)
**Botswana**	22<br>(14, 31)	3.4<br>(2.2, 4.9)	36<br>(26, 52)	2.5<br>(1.8, 3.5)	-27.9<br>(-46.2, -1.6)
**eSwatini**	19<br>(14, 26)	5.6<br>(4, 7.8)	23<br>(17, 31)	3.7<br>(2.7, 5)	-34.6<br>(-55.3, -6.6)
**Lesotho**	34<br>(15, 53)	3.2<br>(1.4, 5)	41<br>(19, 64)	3.2<br>(1.5, 4.9)	-1.9<br>(-35, 57.8)
**Namibia**	17<br>(8, 25)	2.2<br>(1.1, 3.4)	46<br>(27, 71)	3<br>(1.8, 4.7)	36<br>(-11.5, 104.2)
**South Africa**	851<br>(647, 1010)	3.6<br>(2.7, 4.4)	942<br>(817, 1064)	2<br>(1.8, 2.3)	-44.3<br>(-52.5, -26.9)

**Zimbabwe**	189<br>(150, 226)	4.4<br>(3.5, 5.2)	248<br>(76, 447)	3.5<br>(1, 6.2)	-20.5<br>(-76.8, 45)
**Western sub-Saharan Africa**	10916<br>(6147, 16263)	11.4<br>(6.4, 17)	14465<br>(8900, 20856)	7.3<br>(4.5, 10.4)	-36<br>(-52.3, -13.6)
**Benin**	139<br>(92, 200)	6.6<br>(4.3, 9.5)	214<br>(148, 309)	4.2<br>(2.9, 6)	-35.8<br>(-66, -1.8)
**Burkina Faso**	295<br>(222, 364)	6.6<br>(5.1, 8)	366<br>(26, 695)	4<br>(0.3, 7.5)	-40.3<br>(-96.1, 18.3)
**Cameroon**	424<br>(326, 528)	8.6<br>(6.6, 10.7)	760<br>(337, 1175)	5.9<br>(2.6, 9.2)	-31.2<br>(-63.9, -0.9)
**Cape Verde**	6<br>(5, 8)	2.9<br>(2.2, 3.7)	17<br>(14, 20)	3.6<br>(3.1, 4.2)	24.1<br>(-7.6, 67.8)
**Chad**	149<br>(94, 213)	5<br>(3.1, 7.3)	326<br>(233, 435)	5.4<br>(3.8, 7.2)	7.4<br>(-20.8, 57.2)
**Côte d'Ivoire**	337<br>(242, 459)	7<br>(5, 9.7)	649<br>(481, 875)	5.4<br>(4.1, 7.4)	-22.9<br>(-49.5, 5.4)
**The Gambia**	21<br>(12, 32)	5.2<br>(3.1, 8)	49<br>(35, 64)	4.9<br>(3.5, 6.4)	-6.5<br>(-36.9, 47.6)
**Ghana**	472<br>(370, 618)	6.7<br>(5.3, 8.7)	733<br>(225, 1409)	4.4<br>(1.3, 8.3)	-35.2<br>(-81.8, 15.8)
**Guinea**	199<br>(115, 322)	5.7<br>(3.3, 9.4)	264<br>(164, 421)	4.7<br>(2.9, 7.5)	-18.9<br>(-41, 24)
**Guinea-Bissau**	33<br>(22, 47)	7.5<br>(4.8, 10.5)	47<br>(34, 65)	5.8<br>(4.3, 8)	-22.7<br>(-43.5, 12.3)
**Liberia**	111<br>(75, 155)	9.5<br>(6.4, 13.4)	118<br>(84, 165)	5.5<br>(3.9, 7.6)	-42.3<br>(-57.6, -22.9)
**Mali**	240<br>(143, 374)	5.6<br>(3.3, 8.9)	233<br>(87, 430)	2.6<br>(1, 4.8)	-53.3<br>(-87.5, -13.2)
**Mauritania**	52<br>(37, 72)	4.8<br>(3.5, 6.7)	62<br>(41, 89)	3<br>(2, 4.3)	-38.3<br>(-52.7, -18.3)
**Niger**	142<br>(95, 231)	4.5<br>(3, 7.3)	207<br>(54, 376)	2.6<br>(0.7, 4.6)	-42.6<br>(-81.6, -8.2)

**Nigeria**	7900<br>(3560, 12858)	16.3<br>(7.4, 26.6)	9891<br>(4136, 16858)	10.7<br>(4.5, 18.4)	-34.6<br>(-53.4, -4.5)
**São Tomé and Príncipe**	8<br>(5, 10)	11.6<br>(7.2, 15.1)	15<br>(11, 21)	13<br>(9.8, 18.6)	11.8<br>(-27.7, 68.3)
**Senegal**	123<br>(77, 180)	3.6<br>(2.2, 5.2)	202<br>(83, 320)	2.6<br>(1.1, 4.1)	-26.8<br>(-56, -2.8)
**Sierra Leone**	175<br>(113, 268)	8.6<br>(5.5, 13.3)	197<br>(80, 292)	5.2<br>(2, 7.8)	-39.3<br>(-83.3, 16.8)
**Togo**	89<br>(64, 119)	6.3<br>(4.5, 8.5)	117<br>(38, 189)	3<br>(0.9, 4.7)	-53.3<br>(-88.3, -13.7)
**Eastern sub-Saharan Africa**	7581<br>(6263, 9086)	9.7<br>(8.1, 11.4)	11742<br>(8581, 14972)	7.1<br>(5.2, 9.1)	-26.4<br>(-47.2, -1.9)
**Burundi**	376<br>(234, 572)	16<br>(10.3, 24)	399<br>(121, 682)	8.9<br>(2.6, 15)	-44.7<br>(-84.5, -6)
**Comoros**	8<br>(1, 14)	3.6<br>(0.5, 6.6)	15<br>(3, 25)	3.3<br>(0.7, 5.5)	-8.6<br>(-30.6, 45.2)
**Djibouti**	9<br>(6, 13)	5.2<br>(3.5, 7.6)	29<br>(17, 42)	4.8<br>(2.9, 7.1)	-6.9<br>(-36.4, 34.3)
**Eritrea**	98<br>(60, 160)	9.1<br>(5.9, 14.4)	219<br>(158, 294)	8.5<br>(6.3, 11.1)	-7.6<br>(-36.3, 44.9)
**Ethiopia**	1743<br>(1087, 2709)	8.5<br>(5.5, 12.7)	2610<br>(2180, 3113)	6.4<br>(5.4, 7.7)	-24.8<br>(-50.9, 26.1)
**Kenya**	1380<br>(850, 2003)	15.8<br>(9.8, 22.9)	2854<br>(1867, 3998)	12.8<br>(8.4, 18)	-19.4<br>(-51.2, 6.7)
**Madagascar**	371<br>(254, 578)	6.9<br>(4.7, 10.5)	570<br>(403, 762)	5<br>(3.6, 6.7)	-26.6<br>(-59, 17)
**Malawi**	412<br>(233, 554)	9.8<br>(6.1, 13)	482<br>(100, 811)	6.2<br>(1.3, 10.4)	-37<br>(-88.4, 34.3)
**Mozambique**	339<br>(215, 473)	5.4<br>(3.5, 7.4)	438<br>(69, 753)	3.8<br>(0.6, 6.4)	-29.5<br>(-89.6, 48.9)
**Rwanda**	523<br>(400, 645)	17.2<br>(13.7, 21.1)	505<br>(311, 729)	8.4<br>(5.1, 12)	-51.2<br>(-70.6, -27.5)

**Somalia**	184<br>(86, 325)	6.6<br>(3.5, 11)	479<br>(291, 726)	7.2<br>(4.5, 10.9)	9<br>(-24.8, 72.6)
**South Sudan**	161<br>(87, 272)	6.5<br>(3.8, 10.7)	272<br>(175, 409)	6.8<br>(4.5, 10)	4.4<br>(-28.4, 62.3)
**Tanzania**	934<br>(672, 1234)	8.2<br>(6.2, 10.8)	1238<br>(494, 1991)	5<br>(2, 8)	-39.3<br>(-76.6, -2.6)
**Uganda**	619<br>(384, 892)	9<br>(5.6, 13)	991<br>(387, 1542)	6.8<br>(2.6, 10.6)	-24.4<br>(-59.3, 11.7)
**Zambia**	421<br>(309, 538)	13.8<br>(10.4, 17.5)	635<br>(494, 796)	9.3<br>(7.3, 11.5)	-32.6<br>(-48.8, -12.6)
**Central sub-Saharan Africa**	1639<br>(1182, 2221)	6.5<br>(4.6, 8.9)	3408<br>(2576, 4464)	5.8<br>(4.3, 7.8)	-10.1<br>(-26.2, 13.8)
**Angola**	319<br>(172, 514)	7.3<br>(3.8, 11.9)	913<br>(650, 1288)	7.5<br>(5.3, 11.1)	4<br>(-28.4, 73.8)
**Central African Republic**	90<br>(50, 137)	6.6<br>(3.7, 9.7)	116<br>(50, 194)	4.5<br>(1.9, 7.6)	-31<br>(-54.9, -6.7)
**Congo**	100<br>(66, 156)	8.4<br>(5.6, 13.1)	175<br>(113, 261)	6.3<br>(4, 9.4)	-25.2<br>(-44.5, 5.4)
**DR Congo**	1052<br>(735, 1469)	6<br>(4.2, 8.4)	2101<br>(1476, 2921)	5.3<br>(3.7, 7.4)	-11.4<br>(-32.7, 14.9)
**Equatorial Guinea**	13<br>(4, 22)	5.7<br>(1.8, 9.9)	26<br>(14, 42)	4.9<br>(2.6, 7.9)	-14.9<br>(-46.3, 83)
**Gabon**	64<br>(41, 91)	10.8<br>(7, 15.6)	78<br>(50, 116)	6.9<br>(4.4, 10.4)	-36.1<br>(-50.3, -17.8)

Appendix Table 14: Number of deaths and age-standardised death rates of cirrhosis due to non-alcoholic steatohepatitis (NASH) per 100 000 in 1990 and 2017 for both sexes and the percentage change by location

	1990		2017		Percentage change in age-standardised rates between 1990 and 2017
	Counts (95% UI)	Rate (95% UI)	Counts (95% UI)	Rate (95% UI)	
**Global**	61880 (55395, 67979)	1.5 (1.3, 1.6)	118030 (108618, 128577)	1.5 (1.3, 1.6)	-1.5 (-9.4, 8.9)
**High-income North America**	3431 (3220, 3619)	1 (1, 1.1)	7234 (6744, 7656)	1.3 (1.2, 1.3)	23.3 (18.6, 29)
**Canada**	246 (220, 274)	0.8 (0.7, 0.8)	453 (399, 509)	0.7 (0.6, 0.8)	-7 (-15.2, 1.4)
**Greenland**	0 (0, 0)	0.9 (0.7, 1.1)	1 (0, 1)	0.9 (0.6, 1)	1.1 (-30.1, 32.1)
**USA**	3185 (2996, 3360)	1.1 (1, 1.1)	6780 (6320, 7178)	1.3 (1.2, 1.4)	26.2 (21.3, 32.5)
**Australasia**	142 (125, 158)	0.6 (0.5, 0.7)	291 (253, 335)	0.6 (0.5, 0.7)	3.3 (-7.7, 16.9)
**Australia**	123 (107, 139)	0.6 (0.6, 0.7)	255 (218, 297)	0.7 (0.6, 0.8)	4 (-8.7, 19.4)
**New Zealand**	19 (17, 21)	0.5 (0.4, 0.5)	36 (32, 40)	0.5 (0.4, 0.5)	-2 (-10.7, 7.5)
**High-income Asia Pacific**	1689 (1485, 1905)	0.8 (0.7, 0.9)	1664 (1459, 1888)	0.4 (0.3, 0.5)	-50.6 (-55.2, -46.7)
**Brunei**	1 (0, 1)	0.5 (0.3, 0.7)	1 (1, 2)	0.3 (0.2, 0.5)	-28.5 (-42.9, 3.2)
**Japan**	1037 (938, 1137)	0.6 (0.6, 0.7)	1165 (1038, 1290)	0.3 (0.3, 0.4)	-42.8 (-46.9, -39.7)
**Singapore**	4 (3, 5)	0.2 (0.1, 0.2)	7 (5, 9)	0.1 (0.1, 0.1)	-41.4 (-48.7, -33.6)
**South Korea**	648 (524, 772)	1.9 (1.6, 2.3)	490 (383, 613)	0.6 (0.5, 0.7)	-70.5 (-75, -65.5)
**Western Europe**	6024 (4441, 7856)	1.1 (0.8, 1.4)	7035 (5262, 9162)	0.9 (0.6, 1.1)	-20.9 (-25.6, -15.6)

**Andorra**	0<br>(0, 1)	0.8<br>(0.5, 1.2)	1<br>(1, 1)	0.7<br>(0.4, 1)	-15.8<br>(-37.5, 14.9)
**Austria**	175<br>(120, 242)	1.6<br>(1.1, 2.2)	171<br>(117, 237)	1.1<br>(0.7, 1.5)	-33.9<br>(-40.8, -25.6)
**Belgium**	104<br>(84, 127)	0.7<br>(0.6, 0.9)	150<br>(118, 186)	0.7<br>(0.6, 0.9)	2.7<br>(-9.2, 15.1)
**Cyprus**	7<br>(5, 11)	0.9<br>(0.6, 1.4)	12<br>(8, 17)	0.7<br>(0.5, 0.9)	-28.8<br>(-46.5, -7.4)
**Denmark**	57<br>(38, 79)	0.8<br>(0.5, 1.2)	88<br>(59, 124)	0.9<br>(0.6, 1.2)	7.1<br>(-8.3, 23.6)
**Finland**	50<br>(34, 69)	0.7<br>(0.5, 1)	128<br>(83, 184)	1.3<br>(0.9, 1.9)	82<br>(55.5, 110.2)
**France**	935<br>(643, 1293)	1.2<br>(0.8, 1.7)	949<br>(650, 1307)	0.8<br>(0.5, 1.1)	-34.2<br>(-42.1, -26.3)
**Germany**	1641<br>(1128, 2272)	1.4<br>(0.9, 1.9)	1976<br>(1354, 2756)	1.2<br>(0.8, 1.7)	-12.2<br>(-23.7, 1.2)
**Greece**	128<br>(87, 180)	0.8<br>(0.6, 1.2)	132<br>(93, 182)	0.6<br>(0.4, 0.8)	-28.6<br>(-37.3, -18.5)
**Iceland**	2<br>(1, 2)	0.7<br>(0.5, 0.8)	3<br>(3, 4)	0.7<br>(0.5, 0.8)	-2.8<br>(-14.7, 10.5)
**Ireland**	17<br>(11, 23)	0.4<br>(0.3, 0.6)	41<br>(28, 56)	0.6<br>(0.4, 0.8)	40<br>(21.6, 58.7)
**Israel**	39<br>(26, 54)	0.8<br>(0.6, 1.1)	84<br>(59, 116)	0.7<br>(0.5, 1)	-7.3<br>(-16.8, 5.3)
**Italy**	896<br>(743, 1058)	1<br>(0.8, 1.2)	699<br>(573, 846)	0.5<br>(0.4, 0.6)	-51.2<br>(-57, -44.9)
**Luxembourg**	8<br>(5, 11)	1.5<br>(1, 2.1)	10<br>(7, 14)	1.1<br>(0.7, 1.5)	-29.5<br>(-42.5, -16.6)
**Malta**	3<br>(2, 5)	0.8<br>(0.5, 1.1)	5<br>(4, 7)	0.7<br>(0.5, 0.9)	-16.5<br>(-26, -5.4)
**Netherlands**	120<br>(85, 163)	0.6<br>(0.4, 0.8)	178<br>(127, 245)	0.5<br>(0.4, 0.7)	-11.7<br>(-20.7, -1.5)

**Norway**	29<br>(25, 33)	0.5<br>(0.4, 0.5)	36<br>(31, 42)	0.4<br>(0.3, 0.5)	-16<br>(-24.7, -9.4)
**Portugal**	280<br>(191, 390)	2.1<br>(1.5, 2.9)	219<br>(151, 303)	1.1<br>(0.7, 1.5)	-49.2<br>(-54.9, -42.5)
**Spain**	967<br>(675, 1338)	1.8<br>(1.3, 2.5)	947<br>(662, 1276)	1<br>(0.7, 1.4)	-43.3<br>(-49.3, -36.6)
**Sweden**	73<br>(63, 84)	0.5<br>(0.5, 0.6)	117<br>(99, 136)	0.6<br>(0.5, 0.7)	13.1<br>(3, 24.7)
**Switzerland**	62<br>(42, 85)	0.6<br>(0.4, 0.9)	86<br>(59, 119)	0.5<br>(0.4, 0.7)	-16<br>(-26.9, -3)
**UK**	426<br>(359, 500)	0.5<br>(0.4, 0.6)	997<br>(846, 1168)	0.9<br>(0.8, 1.1)	79.4<br>(72.1, 92.7)
**Southern Latin America**	708<br>(595, 824)	1.5<br>(1.3, 1.7)	1196<br>(998, 1406)	1.5<br>(1.2, 1.7)	-1.1<br>(-10.8, 9.2)
**Argentina**	372<br>(314, 435)	1.1<br>(1, 1.3)	644<br>(534, 774)	1.2<br>(1, 1.5)	8.6<br>(-6.1, 24.9)
**Chile**	295<br>(242, 348)	2.8<br>(2.3, 3.3)	502<br>(406, 599)	2.1<br>(1.7, 2.6)	-22.6<br>(-32.3, -10.9)
**Uruguay**	41<br>(35, 49)	1.1<br>(0.9, 1.3)	50<br>(42, 61)	1<br>(0.8, 1.2)	-10.3<br>(-22.5, 3.3)
**Eastern Europe**	2955<br>(2729, 3183)	1<br>(1, 1.1)	8646<br>(8081, 9241)	2.8<br>(2.6, 3)	168.4<br>(157.5, 182.5)
**Belarus**	78<br>(68, 89)	0.6<br>(0.5, 0.7)	271<br>(230, 314)	1.8<br>(1.5, 2.1)	209<br>(157.3, 256.3)
**Estonia**	13<br>(11, 15)	0.6<br>(0.6, 0.7)	30<br>(25, 36)	1.5<br>(1.2, 1.8)	128.2<br>(88.8, 168.9)
**Latvia**	24<br>(21, 27)	0.7<br>(0.6, 0.8)	48<br>(41, 56)	1.5<br>(1.3, 1.8)	127.2<br>(90.4, 163.5)
**Lithuania**	32<br>(28, 36)	0.7<br>(0.6, 0.8)	99<br>(86, 112)	2.2<br>(1.9, 2.5)	213.6<br>(184.3, 247.6)
**Moldova**	320<br>(283, 360)	6.7<br>(6, 7.5)	364<br>(314, 418)	6.5<br>(5.6, 7.4)	-3.8<br>(-12.1, 4.5)

**Russia**	1692<br>(1517, 1828)	0.9<br>(0.8, 1)	5708<br>(5354, 6056)	2.7<br>(2.5, 2.8)	189.3<br>(173.8, 220.3)
**Ukraine**	796<br>(740, 860)	1.1<br>(1, 1.2)	2127<br>(1911, 2340)	3.3<br>(2.9, 3.6)	193.9<br>(162.9, 222.6)
**Central Europe**	1791<br>(1619, 1987)	1.2<br>(1.1, 1.3)	2239<br>(2007, 2482)	1.2<br>(1, 1.3)	-2<br>(-6.6, 3.1)
**Albania**	14<br>(12, 16)	0.7<br>(0.5, 0.7)	22<br>(17, 30)	0.5<br>(0.4, 0.7)	-17.2<br>(-37.1, 23.6)
**Bosnia and Herzegovina**	46<br>(41, 54)	1<br>(0.9, 1.2)	47<br>(38, 67)	0.8<br>(0.7, 1.1)	-22.3<br>(-34.5, 4)
**Bulgaria**	114<br>(101, 128)	0.9<br>(0.8, 1)	140<br>(122, 158)	1.1<br>(1, 1.3)	24.5<br>(10.9, 38.9)
**Croatia**	108<br>(96, 121)	1.6<br>(1.4, 1.8)	79<br>(69, 90)	1<br>(0.9, 1.1)	-38.4<br>(-44.4, -32.3)
**Czech Republic**	125<br>(112, 140)	0.9<br>(0.8, 1)	151<br>(132, 170)	0.8<br>(0.7, 0.9)	-10.9<br>(-20.9, -0.4)
**Hungary**	375<br>(334, 419)	2.7<br>(2.4, 3)	233<br>(203, 263)	1.4<br>(1.2, 1.6)	-48.2<br>(-53.2, -42.9)
**Montenegro**	2<br>(2, 3)	0.4<br>(0.3, 0.4)	4<br>(3, 4)	0.4<br>(0.3, 0.5)	2.6<br>(-17.9, 23.2)
**North Macedonia**	10<br>(8, 12)	0.5<br>(0.4, 0.6)	20<br>(16, 23)	0.6<br>(0.5, 0.7)	16.9<br>(-6.3, 38)
**Poland**	276<br>(248, 310)	0.6<br>(0.6, 0.7)	552<br>(485, 631)	0.9<br>(0.8, 1)	46.3<br>(28.7, 63.9)
**Romania**	477<br>(425, 531)	1.6<br>(1.5, 1.8)	743<br>(654, 845)	2.2<br>(1.9, 2.5)	34.5<br>(23.1, 46.8)
**Serbia**	99<br>(77, 122)	0.8<br>(0.6, 1)	98<br>(84, 116)	0.7<br>(0.6, 0.8)	-17.9<br>(-33.4, 4.1)
**Slovakia**	97<br>(83, 123)	1.6<br>(1.4, 2.1)	109<br>(86, 126)	1.3<br>(1, 1.5)	-23.1<br>(-45.3, -5.7)
**Slovenia**	48<br>(43, 54)	1.9<br>(1.7, 2.1)	42<br>(37, 48)	1.1<br>(1, 1.3)	-41.2<br>(-47.4, -34.7)

**Central Asia**	877<br>(783, 970)	1.8<br>(1.6, 2)	2502<br>(2187, 2825)	3.2<br>(2.8, 3.6)	78.4<br>(63.4, 90.7)
**Armenia**	29<br>(25, 32)	1<br>(0.9, 1.2)	91<br>(80, 103)	2.2<br>(2, 2.5)	118.6<br>(97, 142.8)
**Azerbaijan**	98<br>(83, 110)	1.9<br>(1.6, 2.1)	276<br>(219, 336)	3.1<br>(2.4, 3.9)	66.5<br>(40, 106.9)
**Georgia**	105<br>(92, 117)	1.6<br>(1.5, 1.8)	113<br>(96, 131)	2.1<br>(1.8, 2.4)	25.1<br>(12.1, 41.9)
**Kazakhstan**	156<br>(138, 176)	1.2<br>(1, 1.3)	551<br>(435, 653)	3.1<br>(2.5, 3.6)	164.5<br>(118, 203.7)
**Kyrgyzstan**	71<br>(63, 80)	2.3<br>(2, 2.6)	165<br>(138, 190)	3.3<br>(2.8, 3.8)	45.7<br>(27.7, 61.2)
**Mongolia**	44<br>(37, 53)	4<br>(3.5, 4.7)	105<br>(88, 123)	4.8<br>(3.8, 5.7)	19.3<br>(-10.8, 41.6)
**Tajikistan**	46<br>(40, 59)	1.5<br>(1.3, 2)	107<br>(90, 127)	1.8<br>(1.5, 2.2)	17.7<br>(3.5, 33.7)
**Turkmenistan**	54<br>(47, 60)	2.6<br>(2.3, 2.9)	189<br>(163, 216)	4.2<br>(3.7, 4.8)	63.8<br>(46.8, 83.8)
**Uzbekistan**	275<br>(244, 307)	2.2<br>(2, 2.5)	904<br>(762, 1062)	3.6<br>(3.1, 4.2)	61.5<br>(41.8, 82)
**Central Latin America**	4000<br>(3757, 4237)	4.4<br>(4.1, 4.6)	9517<br>(8875, 10143)	4<br>(3.8, 4.3)	-7.8<br>(-12.3, -3)
**Colombia**	222<br>(201, 245)	1.2<br>(1.1, 1.4)	569<br>(490, 655)	1.1<br>(0.9, 1.2)	-15.9<br>(-25.9, -3.7)
**Costa Rica**	39<br>(36, 43)	2.2<br>(2, 2.4)	136<br>(118, 156)	2.7<br>(2.4, 3.1)	26.8<br>(12.9, 43.9)
**El Salvador**	114<br>(97, 126)	3.6<br>(3.1, 4)	249<br>(200, 325)	4.4<br>(3.5, 5.7)	21.2<br>(-3.8, 66.6)
**Guatemala**	239<br>(214, 264)	5.7<br>(5.1, 6.2)	707<br>(619, 807)	6.3<br>(5.5, 7.1)	10.8<br>(-2.6, 26.4)
**Honduras**	115<br>(93, 158)	5.1<br>(4.2, 6.8)	394<br>(289, 549)	6.4<br>(4.7, 8.9)	25.9<br>(-3.4, 58.9)

**Mexico**	2915<br>(2746, 3072)	6.1<br>(5.8, 6.5)	6547<br>(6119, 6934)	5.6<br>(5.2, 5.9)	-8.9<br>(-13.1, -4.7)
**Nicaragua**	56<br>(48, 63)	3.3<br>(2.9, 3.7)	204<br>(145, 238)	4.3<br>(3.1, 5.1)	31.8<br>(-0.4, 53.8)
**Panama**	24<br>(21, 26)	1.6<br>(1.4, 1.8)	64<br>(57, 71)	1.6<br>(1.4, 1.8)	0.9<br>(-9.7, 12.2)
**Venezuela**	276<br>(250, 305)	2.7<br>(2.4, 3)	647<br>(544, 778)	2.2<br>(1.9, 2.7)	-16.6<br>(-29.6, -0.5)
**Andean Latin America**	948<br>(837, 1098)	4.4<br>(3.9, 5.1)	2489<br>(2167, 2814)	4.6<br>(4, 5.2)	5<br>(-8.8, 18.5)
**Bolivia**	180<br>(128, 244)	5.5<br>(3.9, 7.3)	497<br>(385, 634)	5.8<br>(4.6, 7.5)	6.7<br>(-17.1, 42.9)
**Ecuador**	274<br>(247, 301)	4.8<br>(4.4, 5.3)	871<br>(750, 986)	6<br>(5.1, 6.7)	23.2<br>(10.2, 39)
**Peru**	494<br>(424, 576)	3.9<br>(3.4, 4.6)	1122<br>(917, 1336)	3.7<br>(3, 4.4)	-6.4<br>(-23.9, 13.3)
**Caribbean**	861<br>(754, 974)	3.2<br>(2.8, 3.7)	1395<br>(1204, 1668)	2.7<br>(2.4, 3.3)	-15.8<br>(-23.9, 4.1)
**Antigua and Barbuda**	2<br>(1, 2)	3.1<br>(2.7, 3.4)	2<br>(2, 3)	2.4<br>(2.1, 2.8)	-21<br>(-30.2, -9.3)
**The Bahamas**	6<br>(5, 7)	3.7<br>(3.3, 4.2)	10<br>(9, 12)	2.6<br>(2.3, 3)	-28.8<br>(-37.6, -19.2)
**Barbados**	7<br>(6, 7)	2.4<br>(2.1, 2.7)	8<br>(7, 10)	1.8<br>(1.6, 2)	-25.6<br>(-34.7, -15.4)
**Belize**	4<br>(3, 4)	3.7<br>(3.2, 4.2)	12<br>(10, 13)	4.3<br>(3.7, 4.9)	16.5<br>(4.5, 30.1)
**Bermuda**	2<br>(1, 2)	2.5<br>(2.2, 2.8)	2<br>(1, 2)	1.2<br>(1.1, 1.5)	-50<br>(-57.3, -38.9)
**Cuba**	197<br>(175, 222)	1.9<br>(1.7, 2.1)	387<br>(325, 459)	2.1<br>(1.8, 2.5)	11.8<br>(-2.3, 26.1)
**Dominica**	2<br>(1, 2)	2.3<br>(2.1, 2.6)	2<br>(2, 2)	2.2<br>(1.9, 2.5)	-7<br>(-18.4, 5)

**Dominican Republic**	201<br>(131, 238)	5.2<br>(3.3, 6.2)	290<br>(220, 479)	3.2<br>(2.4, 5.2)	-39.2<br>(-56, 40)
**Grenada**	2<br>(2, 3)	3.4<br>(3, 3.8)	4<br>(3, 4)	2.6<br>(2.2, 2.9)	-24<br>(-32.1, -15)
**Guyana**	25<br>(22, 28)	5.8<br>(5.2, 6.5)	34<br>(28, 40)	5.1<br>(4.3, 6.1)	-11.9<br>(-24, 2.2)
**Haiti**	138<br>(93, 204)	4<br>(2.7, 6.1)	263<br>(188, 390)	3.8<br>(2.7, 5.7)	-5.2<br>(-25.8, 24.4)
**Jamaica**	26<br>(22, 29)	1.5<br>(1.3, 1.7)	39<br>(31, 51)	1.3<br>(1.1, 1.7)	-9.1<br>(-27.5, 23.5)
**Puerto Rico**	176<br>(156, 196)	4.8<br>(4.3, 5.3)	209<br>(186, 233)	3.2<br>(2.8, 3.6)	-33.4<br>(-39.9, -25.9)
**Saint Lucia**	4<br>(3, 4)	4.2<br>(3.7, 4.7)	6<br>(5, 7)	2.8<br>(2.5, 3.2)	-32.5<br>(-40, -23.7)
**Saint Vincent and the Grenadines**	2<br>(2, 2)	2.9<br>(2.6, 3.3)	4<br>(4, 5)	3<br>(2.7, 3.5)	3.5<br>(-10.1, 20.1)
**Suriname**	11<br>(10, 13)	4.2<br>(3.7, 4.8)	24<br>(21, 28)	4.1<br>(3.5, 4.7)	-2.5<br>(-15.8, 12)
**Trinidad and Tobago**	26<br>(23, 29)	3<br>(2.6, 3.3)	40<br>(32, 51)	2.2<br>(1.8, 2.8)	-25<br>(-39.8, -7.8)
**Virgin Islands**	4<br>(3, 4)	4<br>(3.5, 4.7)	7<br>(5, 8)	3.6<br>(2.9, 4.2)	-11.2<br>(-27.7, 5.3)
**Tropical Latin America**	3700<br>(3492, 3907)	3.7<br>(3.5, 3.9)	8350<br>(7874, 8783)	3.5<br>(3.3, 3.7)	-5.5<br>(-9.4, -1.7)
**Brazil**	3650<br>(3441, 3851)	3.7<br>(3.5, 3.9)	8213<br>(7750, 8640)	3.5<br>(3.3, 3.7)	-5.8<br>(-9.5, -2)
**Paraguay**	51<br>(41, 68)	2.2<br>(1.8, 2.9)	137<br>(108, 207)	2.5<br>(2, 3.8)	13.3<br>(-10.9, 44.4)
**East Asia**	8216<br>(6724, 9069)	0.9<br>(0.7, 0.9)	11622<br>(10408, 15004)	0.6<br>(0.5, 0.7)	-34.3<br>(-43, -7.7)
**China**	7668<br>(6236, 8464)	0.8<br>(0.7, 0.9)	10725<br>(9566, 13996)	0.5<br>(0.5, 0.7)	-35.2<br>(-44.3, -7.2)

**North Korea**	131<br>(100, 168)	0.7<br>(0.6, 0.9)	242<br>(177, 330)	0.8<br>(0.6, 1)	3.8<br>(-22.8, 36.4)
**Taiwan (province of China)**	280<br>(249, 311)	1.7<br>(1.6, 1.9)	468<br>(409, 531)	1.2<br>(1.1, 1.4)	-28.5<br>(-34.7, -21)
**Southeast Asia**	8816<br>(7621, 9880)	3.3<br>(2.8, 3.7)	17454<br>(15681, 19403)	3<br>(2.7, 3.3)	-9.1<br>(-19.8, 2.2)
**Cambodia**	380<br>(289, 495)	8.3<br>(6.3, 10.7)	935<br>(753, 1172)	8.6<br>(6.9, 10.9)	4.2<br>(-22.3, 38.7)
**Indonesia**	4710<br>(4051, 5220)	4.8<br>(4, 5.3)	8260<br>(7516, 9291)	4.2<br>(3.9, 4.8)	-10.7<br>(-20.9, 4.2)
**Laos**	65<br>(47, 88)	3<br>(2.2, 4.1)	141<br>(98, 193)	3.3<br>(2.3, 4.5)	10.2<br>(-25.8, 60.9)
**Malaysia**	135<br>(105, 159)	1.4<br>(1.1, 1.7)	394<br>(299, 504)	1.6<br>(1.2, 2.1)	10.5<br>(-12.2, 34.6)
**Maldives**	1<br>(1, 1)	1<br>(0.6, 1.3)	2<br>(2, 3)	0.8<br>(0.6, 1)	-16<br>(-42.8, 33.4)
**Mauritius**	19<br>(16, 21)	2.4<br>(2, 2.7)	27<br>(23, 32)	1.6<br>(1.4, 1.9)	-31.2<br>(-38.2, -23)
**Myanmar**	1114<br>(800, 1435)	4.3<br>(3.1, 5.6)	1935<br>(1569, 2331)	4<br>(3.3, 4.8)	-6.8<br>(-28.3, 29.6)
**Philippines**	253<br>(214, 297)	0.8<br>(0.7, 0.9)	1152<br>(937, 1391)	1.6<br>(1.3, 2)	112.7<br>(81, 148.4)
**Sri Lanka**	237<br>(196, 279)	2.1<br>(1.7, 2.4)	368<br>(285, 461)	1.5<br>(1.2, 1.9)	-25.8<br>(-39.4, -10.9)
**Seychelles**	1<br>(1, 2)	2.4<br>(2, 2.9)	4<br>(3, 4)	3.4<br>(2.8, 3.9)	41.5<br>(21.8, 66.9)
**Thailand**	1005<br>(803, 1198)	2.6<br>(2, 3.1)	2385<br>(1850, 2860)	2.4<br>(1.9, 2.9)	-7.5<br>(-23.8, 9.6)
**East Timor**	6<br>(4, 9)	2<br>(1.3, 2.8)	20<br>(13, 28)	2.6<br>(1.6, 3.5)	32.1<br>(-4.3, 87.2)
**Vietnam**	878<br>(663, 1143)	2.2<br>(1.7, 2.9)	1806<br>(1425, 2289)	2<br>(1.6, 2.5)	-10.2<br>(-35.4, 28.3)

**Oceania**	59 (50, 69)	1.8 (1.5, 2.1)	130 (109, 156)	1.8 (1.5, 2.1)	-0.3 (-15.1, 16.3)
**American Samoa**	1 (0, 1)	2.1 (1.7, 2.7)	1 (1, 1)	2.3 (1.9, 2.6)	5.4 (-17.7, 32.7)
**Federated States of Micronesia**	1 (1, 2)	2.5 (1.9, 3.8)	2 (1, 2)	2.2 (1.5, 3.2)	-10.8 (-32.9, 20.7)
**Fiji**	3 (2, 4)	0.8 (0.6, 0.9)	7 (6, 9)	1 (0.8, 1.2)	29.2 (1.9, 65.3)
**Guam**	2 (1, 2)	2.2 (1.7, 2.6)	4 (3, 5)	2.2 (1.9, 2.6)	2.2 (-17.3, 30.7)
**Kiribati**	1 (1, 1)	2.6 (1.8, 3.8)	2 (1, 3)	2.9 (1.9, 4.6)	13.9 (-9.4, 40.8)
**Marshall Islands**	0 (0, 1)	2.8 (2, 3.6)	1 (1, 1)	2.9 (1.9, 3.9)	1.9 (-19.2, 26.2)
**Northern Mariana Islands**	0 (0, 1)	2.3 (1.8, 2.8)	1 (1, 1)	1.9 (1.6, 2.2)	-14.7 (-33.1, 5.8)
**Papua New Guinea**	38 (31, 47)	1.7 (1.4, 2)	88 (68, 111)	1.6 (1.3, 2)	-4.3 (-23.9, 18.6)
**Samoa**	2 (1, 3)	2.1 (1.5, 3.4)	3 (2, 4)	2.2 (1.6, 3.1)	2.8 (-17.3, 28)
**Solomon Islands**	3 (2, 4)	1.8 (1.4, 2.2)	7 (5, 8)	1.9 (1.5, 2.2)	5.7 (-14.5, 30.9)
**Tonga**	2 (1, 2)	3 (2.5, 3.6)	3 (2, 3)	3.6 (2.9, 4.3)	19 (-3.8, 46.2)
**Vanuatu**	2 (1, 3)	2.7 (1.3, 4.5)	5 (2, 9)	2.8 (1.3, 5.1)	3.1 (-22.1, 37.3)
**North Africa and Middle East**	4444 (2996, 5419)	2.8 (1.8, 3.5)	9983 (7572, 12362)	2.5 (1.9, 3.1)	-9.6 (-29.7, 11.3)
**Afghanistan**	130 (64, 202)	1.9 (1, 3)	200 (123, 289)	1.9 (1.2, 2.9)	1.5 (-20.5, 68)
**Algeria**	141 (90, 216)	1.2 (0.8, 1.9)	375 (275, 501)	1.2 (0.9, 1.6)	-0.1 (-23.3, 34.3)

**Bahrain**	4<br>(3, 5)	2.3<br>(1.8, 2.9)	13<br>(10, 16)	1.4<br>(1.1, 1.8)	-38<br>(-48.6, -24.6)
**Egypt**	2599<br>(1540, 3173)	12<br>(6.3, 15)	5715<br>(3824, 7748)	13.4<br>(7.7, 18.1)	12.2<br>(-17.2, 42.5)
**Iran**	243<br>(204, 284)	1<br>(0.8, 1.2)	747<br>(673, 809)	1.1<br>(1, 1.2)	15.3<br>(-5.2, 37.8)
**Iraq**	105<br>(81, 140)	1.3<br>(1, 1.8)	142<br>(118, 171)	0.6<br>(0.5, 0.7)	-54.6<br>(-62.8, -43.2)
**Jordan**	21<br>(16, 27)	1.5<br>(1.1, 2)	69<br>(53, 86)	1.3<br>(1, 1.6)	-14<br>(-34.8, 17.3)
**Kuwait**	6<br>(5, 8)	0.9<br>(0.7, 1)	28<br>(22, 34)	1.1<br>(0.9, 1.3)	24.5<br>(-0.7, 45.1)
**Lebanon**	29<br>(22, 36)	1.4<br>(1, 1.7)	70<br>(47, 92)	1.2<br>(0.8, 1.6)	-9.8<br>(-44.1, 22.7)
**Libya**	36<br>(25, 51)	1.9<br>(1.3, 2.7)	94<br>(63, 126)	2.2<br>(1.5, 2.9)	13.2<br>(-19.2, 77)
**Morocco**	184<br>(132, 260)	1.3<br>(0.9, 1.9)	427<br>(329, 547)	1.4<br>(1.1, 1.8)	8.4<br>(-22, 51.3)
**Palestine**	11<br>(8, 15)	1.3<br>(0.9, 1.7)	29<br>(24, 35)	1.3<br>(1, 1.6)	-2.6<br>(-23.4, 33.1)
**Oman**	8<br>(6, 11)	1.1<br>(0.8, 1.6)	27<br>(19, 35)	1.4<br>(1, 1.8)	19.5<br>(-14.1, 57.1)
**Qatar**	4<br>(3, 5)	3.5<br>(2.6, 4.4)	19<br>(14, 25)	2.3<br>(1.7, 3)	-32.4<br>(-47.6, -12.5)
**Saudi Arabia**	168<br>(66, 238)	2.9<br>(1.1, 4.2)	402<br>(247, 537)	3<br>(1.6, 4)	3.6<br>(-29.1, 68.1)
**Sudan**	184<br>(100, 305)	2.1<br>(1, 3.6)	334<br>(196, 506)	2<br>(1.1, 3.1)	-6.4<br>(-33.6, 38.3)
**Syria**	81<br>(65, 100)	1.5<br>(1.2, 1.9)	204<br>(160, 256)	1.7<br>(1.3, 2.1)	7.8<br>(-14.6, 35.6)
**Tunisia**	60<br>(40, 84)	1.3<br>(0.9, 1.8)	149<br>(109, 198)	1.3<br>(1, 1.7)	0.8<br>(-31.2, 42.8)

**Turkey**	340<br>(235, 479)	1<br>(0.7, 1.4)	709<br>(584, 840)	0.8<br>(0.7, 1)	-16.2<br>(-42.9, 21)
**United Arab Emirates**	8<br>(3, 13)	1.7<br>(0.7, 2.7)	62<br>(29, 86)	1.7<br>(0.7, 2.4)	-4.7<br>(-36, 48.5)
**Yemen**	78<br>(39, 130)	1.6<br>(0.8, 2.8)	160<br>(108, 237)	1.3<br>(0.9, 2)	-19<br>(-48.3, 41)
**South Asia**	6965<br>(6210, 7857)	1.1<br>(1, 1.2)	15939<br>(13946, 20628)	1.2<br>(1, 1.5)	5.7<br>(-7.3, 29.3)
**Bangladesh**	832<br>(554, 1019)	1.5<br>(1, 1.8)	1478<br>(1204, 1869)	1.2<br>(1, 1.5)	-19.4<br>(-36.2, 26.2)
**Bhutan**	5<br>(4, 6)	1.8<br>(1.3, 2.2)	9<br>(6, 13)	1.5<br>(1, 2)	-16.7<br>(-39.7, 13.2)
**India**	4762<br>(4094, 6141)	0.9<br>(0.8, 1.2)	11290<br>(9675, 15901)	1<br>(0.9, 1.5)	10.7<br>(-3.8, 30)
**Nepal**	118<br>(87, 156)	1.1<br>(0.8, 1.5)	283<br>(208, 367)	1.3<br>(1, 1.7)	17<br>(-14, 55.2)
**Pakistan**	1249<br>(755, 1702)	2.1<br>(1.3, 2.9)	2879<br>(2002, 3763)	2.6<br>(1.8, 3.4)	20.8<br>(-6.7, 59.8)
**Southern sub-Saharan Africa**	431<br>(336, 499)	1.4<br>(1.1, 1.7)	642<br>(555, 734)	1.1<br>(1, 1.3)	-22.4<br>(-37.5, 0.1)
**Botswana**	7<br>(5, 10)	1.1<br>(0.7, 1.6)	16<br>(12, 22)	1.1<br>(0.8, 1.5)	-2.7<br>(-27.7, 31.7)
**eSwatini**	7<br>(5, 10)	2.2<br>(1.6, 3.1)	11<br>(8, 15)	1.8<br>(1.3, 2.4)	-19.4<br>(-43.6, 12.6)
**Lesotho**	11<br>(5, 18)	1.1<br>(0.5, 1.7)	17<br>(8, 25)	1.3<br>(0.6, 2)	20.4<br>(-19.4, 88.4)
**Namibia**	5<br>(3, 8)	0.7<br>(0.4, 1.1)	14<br>(9, 22)	1<br>(0.6, 1.5)	30.3<br>(-17.5, 99.3)
**South Africa**	336<br>(258, 399)	1.5<br>(1.1, 1.8)	486<br>(428, 552)	1.1<br>(0.9, 1.2)	-27.3<br>(-37.8, -4.6)
**Zimbabwe**	64<br>(50, 77)	1.5<br>(1.2, 1.8)	98<br>(31, 171)	1.4<br>(0.4, 2.4)	-5.7<br>(-71.7, 66.4)

**Western sub-Saharan Africa**	2866 (1751, 4193)	3.1 (1.9, 4.5)	4505 (2966, 6224)	2.3 (1.5, 3.2)	-23.9 (-43.4, 1.6)
**Benin**	46 (31, 68)	2.2 (1.5, 3.3)	89 (62, 121)	1.8 (1.2, 2.5)	-17.7 (-56.4, 26)
**Burkina Faso**	87 (70, 105)	2 (1.6, 2.4)	125 (9, 223)	1.4 (0.1, 2.5)	-29.8 (-95.1, 32.2)
**Cameroon**	165 (127, 204)	3.5 (2.6, 4.2)	322 (146, 486)	2.6 (1.2, 4)	-24.5 (-59.4, 7.4)
**Cape Verde**	2 (2, 3)	1 (0.7, 1.2)	7 (6, 8)	1.4 (1.2, 1.7)	51 (15, 100.7)
**Chad**	49 (31, 69)	1.7 (1.1, 2.4)	102 (75, 136)	1.7 (1.3, 2.3)	3.8 (-22.4, 49.1)
**Côte d'Ivoire**	99 (71, 135)	2.2 (1.6, 3)	219 (161, 291)	1.9 (1.4, 2.6)	-11.1 (-41.2, 20)
**The Gambia**	7 (4, 10)	1.8 (1.1, 2.7)	19 (13, 24)	1.9 (1.4, 2.5)	5.3 (-27.3, 59.4)
**Ghana**	154 (121, 194)	2.3 (1.8, 2.9)	302 (104, 526)	1.9 (0.6, 3.2)	-17.7 (-75.2, 39.1)
**Guinea**	70 (42, 121)	2 (1.2, 3.6)	96 (61, 152)	1.7 (1.1, 2.8)	-14.8 (-37.1, 30)
**Guinea-Bissau**	11 (7, 15)	2.6 (1.7, 3.5)	19 (14, 26)	2.5 (1.9, 3.4)	-4.2 (-28.7, 35.3)
**Liberia**	38 (26, 51)	3.3 (2.3, 4.5)	49 (36, 66)	2.4 (1.7, 3.2)	-26.8 (-44.8, -4.9)
**Mali**	90 (58, 146)	2.2 (1.4, 3.5)	102 (35, 177)	1.2 (0.4, 2)	-46.1 (-86.1, -5.6)
**Mauritania**	24 (18, 34)	2.3 (1.7, 3.2)	35 (25, 49)	1.7 (1.2, 2.4)	-25.1 (-42.4, -4.3)
**Niger**	47 (32, 76)	1.5 (1, 2.5)	76 (19, 133)	1 (0.2, 1.7)	-36.2 (-79.4, -2.9)
**Nigeria**	1841 (848, 3017)	3.9 (1.8, 6.4)	2734 (1143, 4594)	3.1 (1.3, 5.2)	-22.1 (-45.2, 12.6)

**São Tomé and Príncipe**	3<br>(2, 4)	4.1<br>(2.6, 5.3)	5<br>(4, 8)	5<br>(3.7, 6.8)	20.1<br>(-22.7, 83.3)
**Senegal**	50<br>(30, 73)	1.5<br>(0.9, 2.1)	91<br>(35, 138)	1.2<br>(0.5, 1.8)	-18.4<br>(-52.4, 7)
**Sierra Leone**	54<br>(36, 80)	2.7<br>(1.8, 4)	67<br>(26, 102)	1.8<br>(0.7, 2.7)	-31.8<br>(-82.3, 30.7)
**Togo**	29<br>(22, 39)	2.2<br>(1.6, 2.9)	46<br>(15, 72)	1.2<br>(0.4, 1.9)	-43.1<br>(-85.3, 1)
**Eastern sub-Saharan Africa**	2432<br>(2037, 2909)	3.2<br>(2.7, 3.8)	4047<br>(2969, 5144)	2.5<br>(1.8, 3.2)	-20<br>(-42.3, 5)
**Burundi**	101<br>(64, 153)	4.5<br>(2.9, 6.6)	111<br>(33, 185)	2.6<br>(0.7, 4.3)	-42.3<br>(-84, -6.4)
**Comoros**	3<br>(0, 6)	1.5<br>(0.2, 2.7)	7<br>(1, 11)	1.5<br>(0.3, 2.4)	-0.6<br>(-23.6, 58.7)
**Djibouti**	3<br>(2, 4)	1.8<br>(1.2, 2.7)	12<br>(7, 18)	2.1<br>(1.3, 3.1)	13.6<br>(-22.8, 62.5)
**Eritrea**	29<br>(18, 47)	2.9<br>(1.9, 4.6)	70<br>(51, 92)	3<br>(2.1, 3.9)	0.4<br>(-30.6, 53.7)
**Ethiopia**	594<br>(385, 884)	3<br>(2, 4.3)	894<br>(753, 1068)	2.3<br>(1.9, 2.7)	-24.7<br>(-48.5, 25.9)
**Kenya**	459<br>(287, 654)	5.4<br>(3.4, 7.6)	1043<br>(700, 1453)	4.8<br>(3.3, 6.7)	-10.1<br>(-44.9, 17.5)
**Madagascar**	117<br>(81, 175)	2.2<br>(1.5, 3.3)	191<br>(132, 251)	1.8<br>(1.2, 2.3)	-19.8<br>(-54.9, 24.7)
**Malawi**	147<br>(88, 195)	3.6<br>(2.3, 4.7)	183<br>(37, 297)	2.4<br>(0.5, 3.9)	-32.3<br>(-87.3, 28.7)
**Mozambique**	116<br>(74, 162)	1.9<br>(1.2, 2.6)	157<br>(26, 261)	1.4<br>(0.2, 2.3)	-24<br>(-88.1, 55.4)
**Rwanda**	133<br>(102, 165)	4.4<br>(3.5, 5.5)	146<br>(89, 207)	2.5<br>(1.5, 3.5)	-43.4<br>(-65.8, -17.9)
**Somalia**	62<br>(31, 108)	2.3<br>(1.3, 3.9)	171<br>(104, 264)	2.7<br>(1.7, 4.1)	14.1<br>(-20.1, 75.1)

**South Sudan**	58<br>(32, 96)	2.4<br>(1.5, 3.9)	102<br>(65, 152)	2.6<br>(1.8, 3.9)	8.9<br>(-24.7, 67.2)
**Tanzania**	300<br>(219, 399)	2.7<br>(2.1, 3.5)	449<br>(178, 698)	1.9<br>(0.7, 2.9)	-30.7<br>(-73.3, 8.7)
**Uganda**	168<br>(101, 246)	2.5<br>(1.5, 3.6)	284<br>(111, 438)	2<br>(0.8, 3.1)	-18.2<br>(-55.3, 15.8)
**Zambia**	140<br>(104, 176)	4.7<br>(3.5, 5.8)	226<br>(173, 281)	3.5<br>(2.7, 4.3)	-25.8<br>(-40.9, -6.6)
**Central sub-Saharan Africa**	525<br>(380, 726)	2.1<br>(1.5, 3)	1151<br>(876, 1471)	2<br>(1.5, 2.7)	-3.5<br>(-20.4, 18.5)
**Angola**	105<br>(59, 175)	2.5<br>(1.3, 4.2)	352<br>(256, 498)	3<br>(2.2, 4.4)	22.2<br>(-17.7, 98.8)
**Central African Republic**	27<br>(15, 39)	2<br>(1.1, 2.9)	37<br>(16, 61)	1.5<br>(0.6, 2.4)	-24.3<br>(-47.9, -0.1)
**Congo**	32<br>(21, 49)	2.7<br>(1.8, 4.1)	65<br>(41, 94)	2.4<br>(1.5, 3.6)	-11.1<br>(-32.8, 21.5)
**DR Congo**	338<br>(242, 468)	2<br>(1.4, 2.7)	653<br>(477, 880)	1.7<br>(1.2, 2.4)	-12.2<br>(-32.4, 12.8)
**Equatorial Guinea**	4<br>(1, 7)	2<br>(0.6, 3.4)	13<br>(7, 21)	2.6<br>(1.3, 4.2)	27.1<br>(-20.6, 168.8)
**Gabon**	20<br>(14, 28)	3.3<br>(2.3, 4.9)	31<br>(21, 46)	2.8<br>(1.8, 4.3)	-14.9<br>(-33.9, 8.9)

Appendix Table 15: Number of deaths and age-standardised death rates of cirrhosis due to other causes per 100 000 in 1990 and 2017 for both sexes and the percentage change by location

	1990		2017		Percentage change in age-standardised rates between 1990 and 2017
	Counts (95% UI)	Rate (95% UI)	Counts (95% UI)	Rate (95% UI)	
**Global**	109643 (96648, 126682)	2.5 (2.2, 2.8)	146356 (130858, 164570)	1.9 (1.7, 2.1)	-23.4 (-28.7, -16.8)
**High-income North America**	8156 (7490, 8860)	2.3 (2.1, 2.5)	13914 (12612, 15160)	2.3 (2.1, 2.6)	1.5 (-3.3, 7.7)
**Canada**	616 (519, 708)	1.9 (1.6, 2.1)	1062 (893, 1230)	1.6 (1.3, 1.8)	-16.6 (-24.4, -8.1)
**Greenland**	1 (1, 1)	1.9 (1.5, 2.5)	1 (1, 2)	1.8 (1.3, 2.1)	-4.9 (-35.4, 23.1)
**USA**	7539 (6916, 8179)	2.4 (2.2, 2.6)	12850 (11689, 13999)	2.4 (2.2, 2.7)	3.4 (-1.8, 9.9)
**Australasia**	98 (83, 114)	0.4 (0.4, 0.5)	176 (146, 211)	0.4 (0.3, 0.4)	-16 (-25.3, -4.5)
**Australia**	84 (70, 99)	0.4 (0.4, 0.5)	154 (125, 187)	0.4 (0.3, 0.4)	-14.9 (-25.4, -2.3)
**New Zealand**	14 (12, 16)	0.4 (0.3, 0.4)	22 (19, 25)	0.3 (0.2, 0.3)	-22.8 (-30.2, -14.6)
**High-income Asia Pacific**	3583 (3098, 4446)	1.9 (1.7, 2.3)	3720 (3260, 4212)	0.8 (0.7, 0.9)	-58.4 (-65.9, -54.7)
**Brunei**	2 (1, 2)	1.5 (1.1, 1.9)	3 (2, 4)	0.9 (0.6, 1.5)	-38.5 (-52.4, -3.9)
**Japan**	2100 (1874, 2303)	1.3 (1.2, 1.4)	2693 (2383, 3023)	0.7 (0.6, 0.7)	-49.8 (-53.9, -46.3)
**Singapore**	29 (25, 35)	1.4 (1.1, 1.6)	43 (35, 52)	0.6 (0.5, 0.8)	-53.1 (-59.1, -46.5)
**South Korea**	1452 (1176, 2312)	4.8 (3.9, 6.9)	981 (795, 1207)	1.2 (1, 1.5)	-74.5 (-82.2, -69.6)
**Western Europe**	13760 (10757, 17465)	2.4 (1.9, 3)	14341 (11253, 17933)	1.6 (1.3, 2)	-33.3 (-38.1, -28.6)

**Andorra**	1<br>(1, 2)	1.9<br>(1.2, 3)	2<br>(1, 3)	1.4<br>(0.9, 2)	-29.1<br>(-46.8, -2.9)
**Austria**	392<br>(267, 551)	3.4<br>(2.3, 4.8)	348<br>(237, 483)	2<br>(1.4, 2.8)	-41<br>(-48.9, -31.2)
**Belgium**	252<br>(208, 309)	1.6<br>(1.4, 2)	297<br>(244, 363)	1.3<br>(1, 1.6)	-23.4<br>(-32.8, -13.7)
**Cyprus**	20<br>(12, 29)	2.5<br>(1.6, 3.7)	26<br>(17, 37)	1.4<br>(0.9, 1.9)	-43.9<br>(-59.6, -28.1)
**Denmark**	116<br>(79, 163)	1.6<br>(1.1, 2.3)	170<br>(112, 239)	1.6<br>(1, 2.2)	-0.6<br>(-15.7, 15.5)
**Finland**	100<br>(70, 136)	1.4<br>(1, 2)	196<br>(127, 283)	1.9<br>(1.3, 2.8)	31.7<br>(7.2, 56.7)
**France**	2372<br>(1632, 3287)	2.9<br>(2, 4)	2096<br>(1440, 2884)	1.6<br>(1.1, 2.2)	-45.9<br>(-53.1, -38.8)
**Germany**	3725<br>(2599, 5031)	3<br>(2.1, 4)	3892<br>(2636, 5369)	2.1<br>(1.5, 3)	-28.3<br>(-38.2, -16.1)
**Greece**	338<br>(227, 474)	2.2<br>(1.5, 3.1)	284<br>(193, 388)	1.1<br>(0.8, 1.5)	-49.3<br>(-56.5, -40.6)
**Iceland**	4<br>(3, 4)	1.3<br>(1.1, 1.5)	5<br>(4, 6)	1<br>(0.8, 1.2)	-20.6<br>(-29.7, -11.1)
**Ireland**	41<br>(28, 55)	1<br>(0.7, 1.3)	75<br>(51, 101)	1.1<br>(0.7, 1.4)	5<br>(-9.9, 19.8)
**Israel**	102<br>(71, 138)	2.1<br>(1.5, 2.8)	189<br>(132, 252)	1.6<br>(1.1, 2.1)	-22.8<br>(-32.3, -13.2)
**Italy**	2559<br>(2035, 3226)	2.8<br>(2.3, 3.5)	1886<br>(1549, 2326)	1.1<br>(0.9, 1.4)	-59.3<br>(-68.7, -51.4)
**Luxembourg**	18<br>(12, 24)	3.3<br>(2.3, 4.4)	19<br>(12, 26)	2<br>(1.3, 2.8)	-40<br>(-52, -28.4)
**Malta**	7<br>(5, 9)	1.6<br>(1.1, 2.2)	9<br>(6, 12)	1.1<br>(0.7, 1.4)	-34.5<br>(-42.8, -25.1)
**Netherlands**	326<br>(228, 428)	1.6<br>(1.2, 2.1)	401<br>(271, 542)	1.1<br>(0.8, 1.5)	-29.3<br>(-37.2, -20.4)

**Norway**	75<br>(65, 87)	1.1<br>(1, 1.3)	80<br>(69, 94)	0.8<br>(0.7, 1)	-27.6<br>(-35.3, -20)
**Portugal**	619<br>(426, 860)	4.7<br>(3.3, 6.3)	414<br>(277, 573)	1.8<br>(1.2, 2.5)	-61.2<br>(-66.9, -55.2)
**Spain**	805<br>(628, 1043)	1.5<br>(1.2, 1.9)	725<br>(555, 969)	0.7<br>(0.5, 0.9)	-54.5<br>(-59.5, -48.7)
**Sweden**	163<br>(148, 181)	1.1<br>(1, 1.2)	236<br>(211, 263)	1.1<br>(1, 1.3)	2.9<br>(-6, 12.5)
**Switzerland**	149<br>(103, 205)	1.4<br>(1, 2)	199<br>(136, 269)	1.1<br>(0.8, 1.6)	-20.8<br>(-32, -8)
**UK**	1565<br>(1452, 1681)	1.8<br>(1.6, 1.9)	2777<br>(2555, 3037)	2.4<br>(2.2, 2.6)	34.2<br>(27.8, 46.1)
**Southern Latin America**	1194<br>(996, 1407)	2.6<br>(2.1, 3)	1775<br>(1447, 2120)	2.2<br>(1.8, 2.6)	-14.8<br>(-23.9, -5)
**Argentina**	685<br>(575, 804)	2.1<br>(1.8, 2.5)	1026<br>(837, 1248)	1.9<br>(1.6, 2.3)	-9.3<br>(-21, 3.3)
**Chile**	435<br>(358, 517)	4.1<br>(3.4, 4.9)	669<br>(536, 813)	2.9<br>(2.3, 3.5)	-29.9<br>(-39, -19.8)
**Uruguay**	75<br>(62, 89)	1.9<br>(1.6, 2.3)	81<br>(65, 98)	1.4<br>(1.2, 1.7)	-25.5<br>(-35.1, -14.7)
**Eastern Europe**	3143<br>(2790, 3501)	1.2<br>(1.1, 1.3)	7079<br>(6364, 7814)	2.4<br>(2.1, 2.6)	102<br>(90.8, 113.4)
**Belarus**	89<br>(74, 107)	0.7<br>(0.6, 0.8)	224<br>(182, 270)	1.6<br>(1.3, 1.9)	120.5<br>(83.4, 152.7)
**Estonia**	13<br>(11, 16)	0.7<br>(0.6, 0.8)	23<br>(18, 29)	1.1<br>(0.9, 1.4)	65.3<br>(36.5, 93.9)
**Latvia**	24<br>(20, 30)	0.7<br>(0.6, 0.8)	38<br>(31, 47)	1.2<br>(1, 1.4)	70.3<br>(44.8, 98.3)
**Lithuania**	32<br>(26, 38)	0.7<br>(0.6, 0.9)	75<br>(61, 91)	1.7<br>(1.4, 2)	132.1<br>(109, 155.9)
**Moldova**	325<br>(266, 397)	7.1<br>(5.9, 8.5)	309<br>(252, 380)	5.6<br>(4.6, 6.7)	-21<br>(-27, -14.8)

**Russia**	1854<br>(1627, 2062)	1.1<br>(1, 1.2)	4768<br>(4282, 5261)	2.3<br>(2.1, 2.5)	113.6<br>(99.3, 137.6)
**Ukraine**	807<br>(724, 908)	1.2<br>(1.1, 1.3)	1642<br>(1447, 1869)	2.7<br>(2.4, 3.1)	123.8<br>(95.3, 146.7)
**Central Europe**	1795<br>(1504, 2127)	1.3<br>(1.1, 1.5)	1873<br>(1533, 2268)	1<br>(0.8, 1.1)	-22.9<br>(-27, -19.1)
**Albania**	25<br>(17, 30)	1<br>(0.7, 1.3)	27<br>(20, 34)	0.7<br>(0.6, 0.9)	-30.1<br>(-45.1, 7)
**Bosnia and Herzegovina**	49<br>(40, 60)	1.2<br>(1, 1.4)	41<br>(32, 59)	0.7<br>(0.6, 1)	-38.2<br>(-47.5, -12.8)
**Bulgaria**	109<br>(92, 130)	1<br>(0.8, 1.1)	119<br>(96, 145)	1<br>(0.8, 1.1)	-2.1<br>(-11.3, 7.3)
**Croatia**	97<br>(79, 118)	1.5<br>(1.3, 1.8)	69<br>(55, 86)	0.8<br>(0.7, 1)	-46.2<br>(-50.7, -41.5)
**Czech Republic**	124<br>(103, 150)	0.9<br>(0.8, 1.1)	128<br>(104, 155)	0.7<br>(0.6, 0.8)	-26.4<br>(-34.5, -18.2)
**Hungary**	292<br>(241, 347)	2.1<br>(1.7, 2.5)	173<br>(136, 215)	1<br>(0.8, 1.2)	-53.1<br>(-58.2, -48.3)
**Montenegro**	2<br>(2, 3)	0.3<br>(0.3, 0.4)	3<br>(2, 4)	0.3<br>(0.2, 0.4)	-10.2<br>(-26.7, 5.2)
**North Macedonia**	11<br>(9, 13)	0.6<br>(0.5, 0.7)	16<br>(12, 19)	0.5<br>(0.4, 0.6)	-14<br>(-27.8, 0.5)
**Poland**	316<br>(263, 381)	0.7<br>(0.6, 0.9)	441<br>(363, 543)	0.7<br>(0.6, 0.9)	-1.9<br>(-14.6, 10.3)
**Romania**	554<br>(467, 657)	2.1<br>(1.8, 2.4)	657<br>(529, 803)	1.9<br>(1.6, 2.3)	-7.6<br>(-16.1, 0.1)
**Serbia**	88<br>(68, 112)	0.8<br>(0.6, 1)	82<br>(66, 103)	0.6<br>(0.4, 0.7)	-31.9<br>(-42.2, -17.6)
**Slovakia**	85<br>(68, 115)	1.4<br>(1.2, 1.9)	84<br>(66, 105)	1<br>(0.8, 1.2)	-30.6<br>(-49.6, -16.3)
**Slovenia**	42<br>(34, 51)	1.7<br>(1.4, 2)	33<br>(26, 41)	0.8<br>(0.7, 1)	-51.3<br>(-56.3, -46.2)

**Central Asia**	1587<br>(1416, 1778)	2.8<br>(2.5, 3.2)	2902<br>(2479, 3351)	3.8<br>(3.2, 4.5)	36.9<br>(27.5, 45.4)
**Armenia**	38<br>(32, 45)	1.4<br>(1.2, 1.7)	117<br>(94, 145)	3<br>(2.4, 3.6)	110.5<br>(87, 136.2)
**Azerbaijan**	156<br>(132, 182)	2.9<br>(2.3, 3.4)	289<br>(222, 370)	3.7<br>(2.7, 4.8)	28.3<br>(7.7, 62.3)
**Georgia**	117<br>(98, 139)	2<br>(1.7, 2.3)	101<br>(80, 126)	1.8<br>(1.5, 2.2)	-7.5<br>(-16.7, 5.5)
**Kazakhstan**	212<br>(181, 247)	1.6<br>(1.3, 1.9)	551<br>(431, 677)	3.3<br>(2.6, 4.1)	109.2<br>(72.6, 137.1)
**Kyrgyzstan**	113<br>(98, 130)	3.3<br>(2.8, 3.8)	180<br>(151, 212)	3.7<br>(3.1, 4.3)	11.7<br>(-1.3, 22.3)
**Mongolia**	107<br>(82, 129)	6.9<br>(5.6, 8.2)	123<br>(97, 149)	6.5<br>(4.3, 8.1)	-6.1<br>(-29, 12.6)
**Tajikistan**	168<br>(138, 197)	3.3<br>(2.9, 3.8)	218<br>(181, 256)	3<br>(2.6, 3.6)	-9.5<br>(-19.9, 0.6)
**Turkmenistan**	115<br>(102, 130)	4.2<br>(3.7, 4.9)	220<br>(186, 255)	5<br>(4.3, 5.8)	18.5<br>(5.9, 31.1)
**Uzbekistan**	560<br>(500, 625)	3.5<br>(3.1, 4)	1103<br>(916, 1309)	4.4<br>(3.7, 5.3)	27<br>(13.1, 42.4)
**Central Latin America**	3569<br>(3320, 3827)	3.4<br>(3.2, 3.7)	5831<br>(5218, 6452)	2.5<br>(2.3, 2.8)	-26.5<br>(-30.8, -20.9)
**Colombia**	251<br>(222, 282)	1.2<br>(1, 1.3)	412<br>(339, 500)	0.8<br>(0.6, 0.9)	-32.8<br>(-41.3, -22.6)
**Costa Rica**	28<br>(24, 33)	1.4<br>(1.2, 1.7)	71<br>(57, 88)	1.5<br>(1.2, 1.8)	2.4<br>(-9.5, 17.2)
**El Salvador**	122<br>(77, 145)	2.9<br>(2.1, 3.5)	140<br>(111, 182)	2.4<br>(1.9, 3.1)	-18.1<br>(-36, 27.5)
**Guatemala**	321<br>(281, 362)	5<br>(4.4, 5.6)	485<br>(411, 563)	3.9<br>(3.3, 4.5)	-22.6<br>(-31.8, -12.7)
**Honduras**	194<br>(154, 233)	5.1<br>(4.3, 6)	272<br>(197, 360)	4.1<br>(2.9, 5.6)	-20.4<br>(-40.8, 4.2)

**Mexico**	2360<br>(2206, 2521)	4.7<br>(4.4, 5)	4012<br>(3623, 4418)	3.6<br>(3.2, 3.9)	-23.8<br>(-28.1, -17.8)
**Nicaragua**	54<br>(45, 65)	2.2<br>(1.9, 2.6)	99<br>(69, 120)	2.1<br>(1.5, 2.5)	-7.2<br>(-32.9, 10.6)
**Panama**	28<br>(24, 32)	1.6<br>(1.4, 1.9)	50<br>(42, 59)	1.3<br>(1.1, 1.5)	-21.1<br>(-29.7, -10.8)
**Venezuela**	211<br>(185, 239)	1.7<br>(1.5, 2)	291<br>(232, 353)	1.1<br>(0.9, 1.3)	-38.8<br>(-47.8, -27.8)
**Andean Latin America**	1185<br>(1011, 1402)	4.1<br>(3.5, 4.8)	1609<br>(1345, 1905)	3<br>(2.5, 3.5)	-27.4<br>(-37.5, -15.2)
**Bolivia**	229<br>(148, 350)	5.2<br>(3.6, 7.2)	330<br>(253, 417)	3.9<br>(3, 5)	-24.4<br>(-43.2, 4.1)
**Ecuador**	252<br>(223, 284)	3.6<br>(3.1, 4.1)	534<br>(448, 631)	3.7<br>(3.1, 4.4)	2.6<br>(-9.6, 16.3)
**Peru**	704<br>(584, 827)	4<br>(3.5, 4.7)	745<br>(589, 913)	2.4<br>(1.9, 3)	-40.4<br>(-51.1, -27.5)
**Caribbean**	856<br>(718, 1027)	2.9<br>(2.5, 3.5)	902<br>(736, 1160)	1.8<br>(1.5, 2.3)	-38.2<br>(-45.1, -23.5)
**Antigua and Barbuda**	1<br>(1, 1)	2<br>(1.7, 2.4)	1<br>(1, 2)	1.4<br>(1.2, 1.7)	-29.2<br>(-37.8, -18.7)
**The Bahamas**	5<br>(4, 5)	2.6<br>(2.2, 3.1)	6<br>(5, 7)	1.7<br>(1.4, 2)	-35.9<br>(-43.6, -27)
**Barbados**	5<br>(4, 5)	1.5<br>(1.3, 1.8)	4<br>(4, 5)	1<br>(0.8, 1.2)	-36.1<br>(-43.9, -27.6)
**Belize**	4<br>(3, 4)	3.1<br>(2.6, 3.6)	7<br>(6, 8)	2.4<br>(2, 2.9)	-20.9<br>(-30.5, -9.4)
**Bermuda**	1<br>(1, 1)	1.6<br>(1.3, 1.9)	1<br>(1, 1)	0.6<br>(0.5, 0.8)	-60.4<br>(-66.3, -52)
**Cuba**	166<br>(141, 197)	1.6<br>(1.3, 1.9)	208<br>(166, 259)	1.2<br>(0.9, 1.4)	-25.7<br>(-34.9, -15.8)
**Dominica**	1<br>(1, 2)	1.9<br>(1.6, 2.3)	1<br>(1, 2)	1.4<br>(1.2, 1.7)	-24.7<br>(-33.4, -13.1)

**Dominican Republic**	280<br>(213, 330)	5.5<br>(3.8, 6.5)	221<br>(162, 365)	2.4<br>(1.8, 4)	-56.2<br>(-67.7, -6.8)
**Grenada**	2<br>(2, 2)	2.6<br>(2.2, 3.1)	2<br>(2, 3)	1.5<br>(1.2, 1.8)	-43.2<br>(-48.9, -36.6)
**Guyana**	20<br>(18, 23)	4.2<br>(3.6, 4.9)	18<br>(15, 22)	3<br>(2.5, 3.6)	-29.9<br>(-39.2, -19.4)
**Haiti**	167<br>(92, 269)	4<br>(2.5, 5.9)	212<br>(147, 310)	3.1<br>(2.2, 4.7)	-22.9<br>(-44.7, 3.6)
**Jamaica**	30<br>(27, 34)	1.4<br>(1.2, 1.6)	27<br>(21, 35)	0.9<br>(0.7, 1.2)	-34.4<br>(-46.6, -14.4)
**Puerto Rico**	109<br>(91, 130)	2.9<br>(2.5, 3.5)	117<br>(95, 142)	1.7<br>(1.4, 2)	-43.4<br>(-50.2, -36.4)
**Saint Lucia**	3<br>(3, 4)	3.1<br>(2.7, 3.6)	3<br>(3, 4)	1.6<br>(1.3, 1.9)	-48.3<br>(-54.1, -41.2)
**Saint Vincent and the Grenadines**	2<br>(1, 2)	1.9<br>(1.5, 2.2)	2<br>(1, 2)	1.2<br>(1, 1.5)	-33.8<br>(-42.4, -23.5)
**Suriname**	10<br>(9, 12)	3.7<br>(3.1, 4.3)	15<br>(12, 18)	2.7<br>(2.2, 3.2)	-27.5<br>(-36.8, -17.2)
**Trinidad and Tobago**	19<br>(17, 22)	2<br>(1.8, 2.4)	20<br>(16, 25)	1.2<br>(1, 1.5)	-40.3<br>(-51.1, -28.8)
**Virgin Islands**	2<br>(2, 3)	2.7<br>(2.3, 3.2)	3<br>(3, 4)	1.9<br>(1.5, 2.3)	-30<br>(-42.1, -17.6)
**Tropical Latin America**	3315<br>(3073, 3547)	3.2<br>(3, 3.4)	4868<br>(4442, 5280)	2.1<br>(2, 2.3)	-33.3<br>(-36.8, -29.9)
**Brazil**	3258<br>(3016, 3487)	3.2<br>(3, 3.5)	4778<br>(4369, 5183)	2.2<br>(2, 2.3)	-33.5<br>(-36.9, -30.1)
**Paraguay**	57<br>(48, 67)	2.2<br>(1.8, 2.7)	89<br>(69, 131)	1.7<br>(1.3, 2.4)	-22.9<br>(-40, 0.6)
**East Asia**	6754<br>(5289, 7719)	0.7<br>(0.6, 0.8)	6411<br>(5430, 8846)	0.4<br>(0.3, 0.5)	-51<br>(-59, -25.5)
**China**	6341<br>(4911, 7247)	0.7<br>(0.6, 0.8)	5842<br>(4904, 8178)	0.3<br>(0.3, 0.5)	-52.2<br>(-60.6, -25.3)

**North Korea**	113<br>(84, 150)	0.7<br>(0.5, 1)	205<br>(144, 282)	0.7<br>(0.5, 1)	1.1<br>(-27.7, 32.5)
**Taiwan (province of China)**	188<br>(156, 223)	1.4<br>(1.1, 1.6)	261<br>(211, 319)	0.7<br>(0.6, 0.9)	-47.6<br>(-53.5, -40.7)
**Southeast Asia**	10294<br>(8360, 12929)	3.1<br>(2.6, 3.6)	12918<br>(11323, 14711)	2.3<br>(2, 2.7)	-25.2<br>(-33.2, -12.9)
**Cambodia**	530<br>(371, 766)	7.6<br>(5.7, 10.1)	770<br>(620, 952)	6.9<br>(5.6, 8.8)	-9.4<br>(-28.3, 21.7)
**Indonesia**	5669<br>(4599, 7338)	4.5<br>(3.9, 5.3)	6144<br>(5436, 6805)	3.3<br>(2.9, 3.7)	-27.7<br>(-36.7, -16.1)
**Laos**	77<br>(48, 122)	2.7<br>(1.9, 3.7)	125<br>(92, 163)	2.7<br>(1.9, 3.6)	-1.1<br>(-36.9, 48)
**Malaysia**	97<br>(78, 117)	1<br>(0.8, 1.2)	193<br>(142, 253)	0.8<br>(0.6, 1.1)	-15.7<br>(-32.3, 3.7)
**Maldives**	1<br>(1, 2)	0.8<br>(0.6, 1.2)	1<br>(1, 2)	0.5<br>(0.4, 0.6)	-43.1<br>(-60.7, -9.8)
**Mauritius**	15<br>(13, 18)	1.8<br>(1.5, 2.2)	14<br>(12, 18)	1<br>(0.8, 1.1)	-47.5<br>(-53.2, -40.1)
**Myanmar**	1526<br>(989, 2305)	4.6<br>(3.1, 6.6)	1673<br>(1363, 2050)	3.6<br>(2.9, 4.4)	-22.2<br>(-44.6, 14.7)
**Philippines**	284<br>(241, 334)	0.7<br>(0.6, 0.8)	905<br>(736, 1112)	1.3<br>(1, 1.6)	83.2<br>(56.6, 112.2)
**Sri Lanka**	243<br>(200, 290)	2<br>(1.6, 2.4)	268<br>(203, 355)	1.2<br>(0.9, 1.6)	-39.4<br>(-50.8, -27)
**Seychelles**	1<br>(1, 1)	1.4<br>(1.1, 1.8)	2<br>(1, 2)	1.6<br>(1.3, 2)	12.6<br>(-1.9, 31.2)
**Thailand**	815<br>(671, 982)	2<br>(1.6, 2.4)	1219<br>(965, 1521)	1.3<br>(1.1, 1.6)	-33<br>(-45.2, -20.4)
**East Timor**	9<br>(6, 16)	1.9<br>(1.3, 2.5)	20<br>(12, 27)	2.2<br>(1.4, 3.1)	19.8<br>(-26.4, 83.6)
**Vietnam**	1013<br>(792, 1275)	2.2<br>(1.7, 2.9)	1566<br>(1202, 2012)	1.8<br>(1.4, 2.4)	-17.2<br>(-39.2, 13.4)

**Oceania**	82<br>(68, 100)	1.6<br>(1.4, 2)	149<br>(122, 185)	1.5<br>(1.3, 1.8)	-6.4<br>(-21.1, 11.1)
**American Samoa**	0<br>(0, 0)	1.1<br>(0.8, 1.4)	0<br>(0, 0)	0.9<br>(0.8, 1.1)	-15<br>(-34.3, 6.1)
**Federated States of Micronesia**	2<br>(1, 2)	2.1<br>(1.5, 3.1)	1<br>(1, 2)	1.5<br>(1, 2.2)	-27.8<br>(-45, -6)
**Fiji**	4<br>(3, 4)	0.7<br>(0.5, 0.8)	6<br>(5, 7)	0.8<br>(0.6, 0.9)	13.6<br>(-7.1, 42.9)
**Guam**	2<br>(1, 2)	1.7<br>(1.3, 2)	2<br>(2, 3)	1.3<br>(1.1, 1.5)	-24<br>(-38.2, -4.1)
**Kiribati**	1<br>(1, 2)	2.1<br>(1.5, 3)	2<br>(1, 2)	2<br>(1.3, 3.2)	-4.9<br>(-23.8, 17.4)
**Marshall Islands**	1<br>(0, 1)	2.1<br>(1.5, 2.7)	1<br>(0, 1)	1.7<br>(1.1, 2.3)	-16.8<br>(-33.5, 4.7)
**Northern Mariana Islands**	0<br>(0, 0)	1.5<br>(1.2, 1.9)	1<br>(0, 1)	1.2<br>(1, 1.4)	-18.5<br>(-34.6, 2.8)
**Papua New Guinea**	58<br>(46, 73)	1.7<br>(1.4, 2.1)	115<br>(91, 148)	1.6<br>(1.3, 2)	-8.5<br>(-25.3, 13.8)
**Samoa**	2<br>(1, 3)	1.4<br>(1, 2.3)	2<br>(1, 3)	1.2<br>(0.9, 1.7)	-15.2<br>(-32.3, 4.4)
**Solomon Islands**	3<br>(3, 4)	1.4<br>(1.1, 1.7)	6<br>(5, 7)	1.3<br>(1.1, 1.6)	-5.6<br>(-22.2, 14)
**Tonga**	1<br>(1, 2)	2<br>(1.6, 2.4)	2<br>(1, 2)	2<br>(1.6, 2.4)	-2<br>(-20.4, 19.5)
**Vanuatu**	3<br>(1, 4)	2.4<br>(1.2, 4.1)	5<br>(3, 8)	2.2<br>(1.1, 4)	-8.6<br>(-30.3, 20.1)
**North Africa and Middle East**	5125<br>(3916, 7336)	2.4<br>(1.9, 2.9)	6344<br>(5383, 7482)	1.5<br>(1.2, 1.9)	-35.3<br>(-46.4, -22.5)
**Afghanistan**	187<br>(64, 366)	2<br>(0.9, 3.3)	403<br>(256, 578)	1.8<br>(1.2, 2.8)	-12.3<br>(-46.5, 68.3)
**Algeria**	224<br>(163, 302)	1.3<br>(0.9, 1.9)	290<br>(214, 377)	0.9<br>(0.7, 1.2)	-27.4<br>(-43.4, -8.4)

**Bahrain**	3<br>(2, 4)	1.5<br>(1.1, 2)	5<br>(4, 6)	0.7<br>(0.6, 1)	-51.2<br>(-60.6, -40.5)
**Egypt**	2261<br>(1738, 3740)	9.3<br>(6.9, 11.9)	2915<br>(2210, 3768)	7.5<br>(4.4, 10.5)	-19.2<br>(-40.7, 3.6)
**Iran**	482<br>(372, 620)	1<br>(0.9, 1.2)	472<br>(419, 526)	0.7<br>(0.7, 0.8)	-27.1<br>(-37.4, -12)
**Iraq**	144<br>(104, 190)	1<br>(0.8, 1.3)	174<br>(146, 206)	0.5<br>(0.4, 0.6)	-52.3<br>(-62.5, -38.4)
**Jordan**	28<br>(21, 37)	1.2<br>(0.9, 1.5)	45<br>(36, 56)	0.7<br>(0.6, 0.9)	-36.7<br>(-51.9, -12.9)
**Kuwait**	4<br>(4, 5)	0.5<br>(0.4, 0.6)	9<br>(7, 11)	0.4<br>(0.3, 0.5)	-17<br>(-34.4, -2)
**Lebanon**	32<br>(26, 41)	1.2<br>(0.9, 1.6)	51<br>(35, 73)	0.8<br>(0.6, 1.2)	-30.5<br>(-56.7, -1.8)
**Libya**	39<br>(29, 51)	1.4<br>(1, 2)	56<br>(41, 74)	1.2<br>(0.9, 1.7)	-12.2<br>(-32.3, 16.1)
**Morocco**	276<br>(202, 357)	1.4<br>(1.1, 1.9)	329<br>(255, 417)	1.1<br>(0.9, 1.5)	-19.7<br>(-37.7, 5.5)
**Palestine**	20<br>(13, 30)	1.2<br>(0.8, 1.7)	26<br>(22, 31)	0.8<br>(0.7, 1.1)	-29.4<br>(-46.2, -1.2)
**Oman**	11<br>(8, 16)	1<br>(0.7, 1.3)	14<br>(11, 18)	0.7<br>(0.5, 0.9)	-28.6<br>(-46.6, -5.3)
**Qatar**	2<br>(2, 3)	1.8<br>(1.4, 2.4)	7<br>(5, 9)	1<br>(0.7, 1.3)	-44.1<br>(-56, -28.2)
**Saudi Arabia**	160<br>(120, 211)	2.2<br>(1.1, 3.2)	157<br>(108, 208)	1.4<br>(0.8, 2)	-35.3<br>(-53.8, -0.3)
**Sudan**	362<br>(157, 756)	2.4<br>(1.2, 3.9)	444<br>(310, 613)	1.6<br>(1, 2.6)	-30.4<br>(-54, 18.9)
**Syria**	135<br>(100, 186)	1.4<br>(1.1, 1.8)	157<br>(119, 197)	1.2<br>(0.9, 1.5)	-18.1<br>(-35.2, 7.4)
**Tunisia**	81<br>(63, 103)	1.4<br>(1, 1.9)	101<br>(75, 133)	1<br>(0.7, 1.3)	-29.2<br>(-49.1, -5.1)

**Turkey**	493 (304, 731)	1 (0.7, 1.5)	420 (340, 513)	0.5 (0.4, 0.6)	-50.1 (-65.7, -22.5)
**United Arab Emirates**	4 (2, 6)	0.9 (0.4, 1.4)	16 (8, 23)	0.6 (0.3, 0.9)	-29.1 (-51.1, 7.9)
**Yemen**	171 (78, 278)	1.9 (1, 3.2)	247 (175, 335)	1.3 (0.8, 1.9)	-31.6 (-53.7, 11.4)
**South Asia**	27122 (22200, 36349)	3.2 (2.8, 3.9)	34920 (29914, 46161)	2.5 (2.2, 3.4)	-21 (-30.7, -7.9)
**Bangladesh**	5420 (4070, 7095)	5.3 (4.3, 6.4)	3705 (2983, 4701)	2.9 (2.3, 3.7)	-45.4 (-58.7, -28.6)
**Bhutan**	17 (12, 25)	4.2 (3.2, 5.5)	19 (13, 25)	2.8 (1.9, 3.7)	-34 (-53.3, -7.8)
**India**	16876 (13270, 24088)	2.5 (2.1, 3.4)	22663 (18874, 33063)	2.1 (1.7, 3.1)	-18 (-29.2, -3.4)
**Nepal**	465 (320, 648)	3.2 (2.3, 4.2)	609 (439, 826)	2.7 (1.9, 3.6)	-15.4 (-37.1, 14.7)
**Pakistan**	4344 (3505, 5322)	5.6 (4, 7.5)	7924 (6068, 9992)	5.8 (4.1, 7.8)	5.2 (-14.8, 30.7)
**Southern sub-Saharan Africa**	895 (737, 1022)	2.7 (2, 3.1)	1020 (836, 1204)	1.8 (1.5, 2.1)	-33.2 (-47.2, -14.3)
**Botswana**	17 (11, 25)	2.4 (1.5, 3.5)	28 (20, 39)	1.9 (1.4, 2.6)	-18.8 (-39, 11.8)
**eSwatini**	14 (10, 20)	3.7 (2.6, 5.4)	18 (13, 25)	2.7 (2, 3.9)	-26 (-45.8, -1.6)
**Lesotho**	25 (11, 38)	2.2 (0.9, 3.4)	30 (13, 44)	2.3 (1, 3.4)	4.9 (-27.2, 57.4)
**Namibia**	15 (8, 22)	1.8 (0.8, 2.7)	30 (19, 46)	1.9 (1.1, 2.9)	6.7 (-31.4, 55.5)
**South Africa**	643 (524, 744)	2.5 (1.9, 3)	669 (574, 762)	1.5 (1.3, 1.7)	-40.3 (-47.5, -25)
**Zimbabwe**	181 (146, 217)	3.7 (2.8, 4.6)	246 (82, 423)	3.3 (1, 5.7)	-11 (-73, 48.4)

**Western sub-Saharan Africa**	8676 (6314, 11867)	7.7 (4.9, 11.3)	12408 (8549, 16701)	5.2 (3.5, 7.2)	-31.7 (-48.4, -11.9)
**Benin**	173 (122, 248)	6 (4, 9)	233 (167, 309)	3.7 (2.6, 5.1)	-37.3 (-65.7, -8.1)
**Burkina Faso**	323 (263, 393)	5.9 (4.6, 7.2)	406 (76, 673)	3.6 (0.4, 6.2)	-39.4 (-93.9, 5.4)
**Cameroon**	410 (309, 515)	7.3 (5.5, 9.2)	653 (309, 973)	4.7 (2.1, 7.3)	-35.6 (-64.7, -8.9)
**Cape Verde**	6 (5, 8)	2.3 (1.7, 2.9)	13 (10, 15)	2.7 (2.2, 3.3)	16.9 (-10.6, 57)
**Chad**	179 (119, 247)	4.7 (3, 6.6)	343 (261, 446)	4.3 (3.2, 5.7)	-8.9 (-29.3, 28.4)
**Côte d'Ivoire**	329 (233, 461)	5.5 (3.9, 8.2)	547 (414, 734)	4.1 (3, 5.7)	-25.7 (-50.1, -1.3)
**The Gambia**	24 (15, 33)	4.6 (2.8, 6.7)	47 (34, 64)	4.1 (2.9, 5.7)	-10 (-36.2, 31.2)
**Ghana**	484 (383, 616)	6.2 (4.6, 7.9)	683 (255, 1067)	4 (1.4, 6.4)	-34.8 (-80.2, 3.9)
**Guinea**	260 (164, 426)	5.9 (3.7, 10.1)	287 (182, 457)	4.2 (2.6, 7)	-28.5 (-44.7, -0.8)
**Guinea-Bissau**	36 (24, 54)	6.4 (4.2, 8.7)	46 (35, 63)	4.9 (3.7, 6.8)	-22.8 (-41.1, 10.4)
**Liberia**	114 (81, 157)	8 (5.6, 11.6)	123 (81, 177)	4.8 (3.3, 6.7)	-39.9 (-52.3, -24.4)
**Mali**	358 (217, 578)	6.4 (4.1, 10.5)	393 (177, 591)	3 (1.1, 4.8)	-53.9 (-86.8, -22.7)
**Mauritania**	65 (47, 90)	5.2 (3.7, 7.5)	72 (51, 96)	3.1 (2.2, 4.2)	-41 (-54.6, -26.3)
**Niger**	242 (144, 390)	4.6 (3.1, 7)	305 (113, 471)	2.5 (0.7, 4.2)	-45.4 (-79.5, -20.2)
**Nigeria**	5171 (3063, 7851)	9.4 (4.9, 15.2)	7708 (3661, 12391)	6.9 (3, 11.4)	-26.8 (-47.9, 2.5)

**São Tomé and Príncipe**	10<br>(7, 13)	10.8<br>(7.5, 14.1)	13<br>(10, 17)	10.4<br>(7.7, 14.1)	-4.3<br>(-35.6, 34.5)
**Senegal**	192<br>(122, 266)	4<br>(2.4, 5.5)	239<br>(111, 342)	2.7<br>(1.1, 4)	-32.1<br>(-58, -14)
**Sierra Leone**	184<br>(129, 280)	7.2<br>(4.9, 11.1)	181<br>(72, 278)	4.2<br>(1.5, 6.6)	-42.1<br>(-85.4, 7.6)
**Togo**	114<br>(86, 155)	6<br>(4.4, 8.4)	115<br>(44, 173)	2.8<br>(0.9, 4.4)	-53<br>(-87.2, -20.8)
**Eastern sub-Saharan Africa**	6921<br>(5550, 8713)	8.1<br>(6.7, 9.9)	10339<br>(7549, 12917)	5.9<br>(4.2, 7.6)	-26.9<br>(-48.6, -3.8)
**Burundi**	280<br>(176, 438)	11.6<br>(7.4, 17.5)	284<br>(88, 466)	6.6<br>(1.8, 10.9)	-43.3<br>(-85, -9.3)
**Comoros**	9<br>(2, 16)	3.9<br>(0.6, 6.7)	16<br>(4, 25)	3.6<br>(0.7, 5.8)	-7.2<br>(-27.9, 35.7)
**Djibouti**	9<br>(5, 13)	4.7<br>(3, 6.9)	22<br>(14, 33)	4<br>(2.5, 6)	-13.9<br>(-38.4, 21.1)
**Eritrea**	89<br>(50, 150)	8.1<br>(5.2, 13.2)	194<br>(139, 261)	8<br>(5.7, 10.8)	-1.5<br>(-31.8, 54.4)
**Ethiopia**	1690<br>(1034, 2640)	7.8<br>(5.3, 10.9)	2413<br>(1993, 2913)	5.3<br>(4.5, 6.5)	-31.8<br>(-52.1, 14.1)
**Kenya**	1167<br>(767, 1642)	12.8<br>(8.4, 18)	2208<br>(1494, 3122)	10.4<br>(7.2, 14.8)	-18.7<br>(-50, 4.7)
**Madagascar**	409<br>(276, 625)	6.3<br>(4.2, 9.7)	518<br>(377, 678)	4.6<br>(3.2, 6)	-27.1<br>(-60.3, 13.5)
**Malawi**	426<br>(235, 594)	9.2<br>(5.9, 12)	500<br>(107, 790)	5.7<br>(1.1, 9.3)	-37.4<br>(-88.4, 15.1)
**Mozambique**	404<br>(257, 539)	5.5<br>(3.4, 7.5)	517<br>(107, 846)	3.8<br>(0.7, 6.3)	-29.9<br>(-87.5, 30)
**Rwanda**	393<br>(297, 482)	11.6<br>(8.8, 14.7)	384<br>(233, 547)	6.3<br>(3.7, 9.1)	-45.8<br>(-69, -20.8)
**Somalia**	167<br>(70, 302)	5.7<br>(3, 9.6)	437<br>(258, 702)	6.2<br>(3.7, 9.9)	9<br>(-25.1, 68)

**South Sudan**	141<br>(71, 239)	5.3<br>(3.1, 8.6)	232<br>(153, 335)	5.4<br>(3.6, 7.8)	0.7<br>(-27.2, 53.1)
**Tanzania**	853<br>(570, 1150)	6.8<br>(5, 9.3)	1243<br>(539, 1816)	4.4<br>(1.7, 6.9)	-34.5<br>(-74.1, -1.3)
**Uganda**	489<br>(293, 704)	6.5<br>(3.8, 9.5)	781<br>(311, 1179)	5<br>(1.9, 7.6)	-22.9<br>(-58.4, 7.1)
**Zambia**	390<br>(274, 518)	11.3<br>(8.5, 14.5)	582<br>(464, 704)	8<br>(6.2, 10.2)	-28.9<br>(-42.5, -10.9)
**Central sub-Saharan Africa**	1532<br>(1079, 2141)	5<br>(3.4, 7.5)	2856<br>(2223, 3787)	4.4<br>(3.2, 6.2)	-12.8<br>(-27, 8.7)
**Angola**	314<br>(158, 517)	5.8<br>(3, 10.3)	746<br>(526, 1085)	5.5<br>(3.8, 8.7)	-5.8<br>(-35.1, 53.9)
**Central African Republic**	73<br>(37, 110)	4.6<br>(2.4, 6.8)	94<br>(40, 157)	3.5<br>(1.5, 6)	-23.6<br>(-46.8, 0.2)
**Congo**	85<br>(59, 124)	6.2<br>(4.1, 9.3)	133<br>(85, 200)	4.9<br>(2.9, 7.7)	-20.8<br>(-39.9, 3.3)
**DR Congo**	1006<br>(732, 1391)	4.7<br>(3.3, 6.9)	1811<br>(1357, 2542)	4.1<br>(2.9, 5.9)	-13.6<br>(-30.9, 7.2)
**Equatorial Guinea**	11<br>(3, 20)	4.3<br>(1.3, 7.3)	20<br>(10, 34)	3.4<br>(1.6, 5.8)	-21.9<br>(-51.1, 59.4)
**Gabon**	44<br>(30, 65)	7<br>(4.6, 10.9)	52<br>(34, 79)	4.8<br>(3, 7.7)	-31.6<br>(-46.4, -15.5)

Appendix Table 16: Number of prevalent cases and age-standardised prevalence rates of decompensated cirrhosis due to hepatitis B per 100 000 in 1990 and 2017 for both sexes by location

	1990		2017	
	Counts (95% UI)	Rate (95% UI)	Counts (95% UI)	Rate (95% UI)
**Global**	1442433 (1371115, 1505204)	30·9 (29·3, 32·2)	2974160 (2810195, 3124432)	36·6 (34·7, 38·4)
**High-income North America**	18872 (17782, 20038)	5·7 (5·3, 6)	29199 (27284, 31174)	5·8 (5·4, 6·1)
**Canada**	1547 (1413, 1699)	4·8 (4·4, 5·2)	2992 (2649, 3350)	5·6 (4·9, 6·2)
**Greenland**	2 (2, 2)	3·9 (3·5, 4·3)	3 (3, 4)	5 (4·4, 5·6)
**USA**	17322 (16364, 18379)	5·7 (5·4, 6·1)	26202 (24596, 27891)	5·8 (5·5, 6·1)
**Australasia**	3103 (2860, 3357)	13·6 (12·5, 14·7)	6533 (5945, 7103)	17 (15·5, 18·4)
**Australia**	2554 (2334, 2788)	13·4 (12·3, 14·7)	5555 (5008, 6086)	17·1 (15·4, 18·7)
**New Zealand**	549 (518, 578)	14·6 (13·7, 15·4)	978 (918, 1039)	16·4 (15·4, 17·4)
**High-income Asia-Pacific**	98563 (91451, 105353)	48·2 (44·8, 51·4)	155070 (142676, 167552)	51·5 (47·3, 55·6)
**Brunei**	104 (97, 111)	59·5 (55·4, 63·5)	305 (275, 335)	72·3 (65·8, 78·7)
**Japan**	57170 (53582, 60911)	35 (32·9, 37·2)	84931 (79238, 90518)	38·3 (35·9, 40·8)
**Singapore**	2051 (1880, 2220)	69·2 (63·1, 75·1)	6655 (6007, 7295)	93·8 (85, 102·6)
**South Korea**	39239 (35099, 43093)	101·1 (90, 111·1)	63178 (55965, 70627)	80·2 (71·3, 88·9)
**Western Europe**	54779 (49927, 59777)	11·2 (10·2, 12·2)	86128 (77551, 95015)	13·7 (12·3, 15·1)
**Andorra**	7 (6, 7)	10·3 (9·4, 11·3)	16 (15, 18)	13·6 (12·3, 15)
**Austria**	1444 (1316, 1583)	14·8 (13·4, 16·2)	1910 (1728, 2112)	14·9 (13·5, 16·4)
**Belgium**	1346 (1100, 1608)	10·5 (8·6, 12·6)	2209 (1756, 2671)	13·8 (11·1, 16·8)
**Cyprus**	55 (51, 60)	6·7 (6·1, 7·3)	150 (135, 167)	8·9 (8, 9·9)
**Denmark**	595 (545, 648)	9·2 (8·4, 10)	1210 (1108, 1315)	15·1 (13·7, 16·4)
**Finland**	589 (540, 641)	9·4 (8·6, 10·2)	1286 (1174, 1400)	16·2 (14·8, 17·7)
**France**	7335 (6676, 8039)	10·5 (9·5, 11·5)	11458 (10444, 12494)	12·6 (11·5, 13·7)

**Germany**	11527 (10509, 12573)	11 (10, 12)	17939 (16256, 19826)	14·3 (12·9, 15·7)
**Greece**	1500 (1362, 1642)	11·3 (10·3, 12·4)	2452 (2178, 2757)	15·6 (13·8, 17·6)
**Iceland**	17 (15, 19)	6·3 (5·5, 7·1)	54 (48, 60)	12·6 (11·2, 14·2)
**Ireland**	274 (251, 300)	7·4 (6·8, 8·1)	856 (779, 939)	13·8 (12·6, 15·1)
**Israel**	448 (411, 484)	9·4 (8·7, 10·2)	1326 (1194, 1458)	13·7 (12·3, 15·1)
**Italy**	15942 (14177, 17629)	20·8 (18·6, 23)	21113 (18536, 23799)	22·2 (19·6, 25)
**Luxembourg**	71 (65, 77)	14·3 (13·1, 15·6)	133 (118, 148)	16·4 (14·7, 18·2)
**Malta**	28 (25, 30)	6·5 (5·9, 7·1)	58 (52, 64)	9·1 (8·2, 10)
**Netherlands**	1412 (1296, 1535)	7·8 (7·1, 8·5)	3165 (2871, 3454)	13·1 (11·9, 14·2)
**Norway**	482 (456, 510)	9·4 (8·9, 10)	842 (793, 891)	12 (11·3, 12·7)
**Portugal**	1224 (1103, 1365)	9·9 (8·9, 11)	1686 (1511, 1876)	10·4 (9·4, 11·6)
**Spain**	5196 (4221, 6213)	10·8 (8·8, 12·9)	7930 (6402, 9536)	11·5 (9·4, 13·9)
**Sweden**	806 (747, 865)	7·4 (6·9, 8)	1575 (1460, 1692)	11·6 (10·7, 12·4)
**Switzerland**	743 (682, 809)	8·3 (7·6, 9·1)	1469 (1336, 1599)	11·7 (10·7, 12·7)
**United Kingdom**	3686 (3397, 3982)	5·3 (4·9, 5·7)	7200 (6606, 7783)	8·1 (7·4, 8·7)
**Eastern Europe**	65913 (61693, 70107)	24·8 (23·3, 26·4)	111512 (103568, 119006)	39·5 (36·8, 42·1)
**Belarus**	2808 (2526, 3104)	23·5 (21·2, 26)	4566 (4046, 5119)	35·4 (31·5, 39·3)
**Estonia**	480 (431, 532)	26·2 (23·5, 28·8)	633 (558, 708)	35·5 (31·4, 39·6)
**Latvia**	748 (670, 826)	23·3 (21, 25·7)	891 (790, 994)	33·2 (29·5, 37)
**Lithuania**	1155 (1041, 1274)	27·3 (24·5, 30)	1540 (1355, 1724)	39·5 (35·1, 44·1)
**Moldova**	2962 (2642, 3308)	62 (55·5, 68·9)	2483 (2201, 2792)	48·7 (43·3, 54·3)
**Russia**	40663 (38088, 43109)	23·2 (21·8, 24·6)	74761 (69651, 79541)	38·3 (35·7, 40·6)
**Ukraine**	17097 (15883, 18261)	26·7 (24·9, 28·5)	26638 (24618, 28527)	44 (40·8, 47·1)
**Central Europe**	63985 (58608, 69293)	44·4 (40·7, 48)	86698 (78931, 94805)	51·8 (47·2, 56·2)
**Albania**	1127 (1030, 1223)	42·8 (39·1, 46·6)	2045 (1853, 2236)	55·9 (51, 61·1)

**Bosnia and Herzegovina**	2206 (2016, 2404)	46·2 (42·4, 50·1)	2772 (2499, 3051)	55·4 (50·2, 60·3)
**Bulgaria**	4379 (4000, 4773)	38·1 (34·9, 41·3)	5282 (4772, 5795)	48·8 (44·4, 53·3)
**Croatia**	3383 (3079, 3688)	54·2 (49·5, 58·9)	3044 (2752, 3367)	46·9 (42·5, 51·6)
**Czech Republic**	4745 (4325, 5187)	38 (34·7, 41·4)	6901 (6251, 7582)	43·8 (39·7, 47·9)
**Hungary**	8461 (7715, 9199)	63·9 (58·2, 69·3)	7873 (7032, 8727)	53·7 (48·2, 59·1)
**Macedonia**	805 (733, 873)	38·8 (35·4, 42·1)	1570 (1420, 1723)	52·5 (47·6, 57·1)
**Montenegro**	250 (229, 273)	38 (34·6, 41·3)	420 (379, 462)	49·5 (44·9, 54)
**Poland**	15997 (14645, 17377)	36·9 (33·8, 40)	29178 (26534, 32126)	53·5 (48·8, 58·3)
**Romania**	13995 (12748, 15349)	50·6 (46·4, 55·2)	16257 (14637, 17850)	55·5 (50·1, 60·6)
**Serbia**	3519 (3179, 3854)	30·6 (27·9, 33·5)	4541 (4095, 4996)	36·3 (33, 39·7)
**Slovakia**	3722 (3412, 4061)	64 (58·7, 69·7)	5284 (4746, 5804)	68·3 (61·6, 74·8)
**Slovenia**	1397 (1276, 1527)	57·8 (52·9, 63·2)	1532 (1385, 1682)	48·3 (44, 52·8)
**Central Asia**	15717 (14696, 16748)	28 (26·1, 29·8)	33767 (31213, 36328)	38·6 (35·8, 41·6)
**Armenia**	816 (760, 875)	25·6 (23·9, 27·3)	1417 (1307, 1538)	37·4 (34·7, 40·4)
**Azerbaijan**	1702 (1579, 1825)	28·2 (26·2, 30·2)	4245 (3877, 4592)	39·3 (36·2, 42·5)
**Georgia**	1723 (1594, 1863)	27·9 (25·9, 29·9)	1827 (1672, 1990)	37·5 (34·5, 40·5)
**Kazakhstan**	3280 (3048, 3510)	21·7 (20·2, 23·2)	6597 (6063, 7138)	35·6 (32·7, 38·4)
**Kyrgyzstan**	1117 (1040, 1197)	32·1 (29·8, 34·4)	2248 (2070, 2428)	40·9 (37·7, 44)
**Mongolia**	432 (401, 461)	31 (28·6, 33·3)	1032 (951, 1112)	34·6 (32, 37·2)
**Tajikistan**	1008 (936, 1078)	28·3 (26·2, 30·3)	2679 (2467, 2889)	37·7 (34·8, 40·7)
**Turkmenistan**	843 (780, 902)	33·1 (30·7, 35·5)	1981 (1828, 2137)	41·9 (38·7, 45·2)
**Uzbekistan**	4797 (4470, 5122)	33·1 (30·7, 35·4)	11741 (10818, 12656)	40·5 (37·4, 43·5)
**Central Latin America**	9219 (8491, 9905)	8·5 (7·8, 9·2)	26745 (24365, 29127)	10·8 (9·9, 11·8)
**Colombia**	1259 (1122, 1400)	5·4 (4·8, 6)	5218 (4619, 5848)	9·8 (8·7, 10·9)
**Costa Rica**	144 (129, 159)	6·6 (5·9, 7·3)	526 (466, 588)	10·4 (9·3, 11·7)

**El Salvador**	249 (223, 278)	7·1 (6·4, 8)	580 (513, 647)	10·1 (8·9, 11·2)
**Guatemala**	410 (367, 456)	8·5 (7·6, 9·5)	1374 (1220, 1537)	11 (9·7, 12·2)
**Honduras**	224 (200, 251)	8·5 (7·6, 9·5)	762 (677, 854)	11 (9·8, 12·3)
**Mexico**	5908 (5513, 6323)	10·7 (9·9, 11·4)	14042 (12957, 15032)	11·4 (10·5, 12·1)
**Nicaragua**	152 (136, 168)	7·3 (6·5, 8·1)	548 (488, 613)	10·5 (9·4, 11·7)
**Panama**	111 (99, 123)	6·2 (5·6, 6·9)	418 (371, 467)	10·4 (9·3, 11·6)
**Venezuela**	762 (684, 846)	6 (5·4, 6·6)	3279 (2902, 3674)	10·6 (9·5, 11·9)
**Andean Latin America**	3100 (2788, 3421)	12·7 (11·4, 14)	12125 (10853, 13502)	21·6 (19·3, 24·1)
**Bolivia**	445 (399, 492)	11·4 (10·3, 12·7)	1863 (1665, 2067)	19·7 (17·6, 21·8)
**Ecuador**	855 (769, 940)	13·3 (12, 14·6)	3404 (3047, 3781)	22·2 (19·9, 24·7)
**Peru**	1800 (1615, 1999)	12·7 (11·4, 14·1)	6859 (6140, 7662)	21·8 (19·5, 24·5)
**Caribbean**	2333 (2177, 2491)	8·1 (7·6, 8·7)	5739 (5311, 6136)	11·4 (10·5, 12·2)
**Antigua and Barbuda**	4 (4, 5)	8·2 (7·6, 8·8)	11 (11, 12)	11 (10·2, 11·9)
**The Bahamas**	16 (14, 17)	8·1 (7·5, 8·7)	43 (40, 46)	10·5 (9·8, 11·3)
**Barbados**	20 (19, 22)	7·5 (6·9, 8·1)	46 (42, 49)	10·9 (10, 11·7)
**Belize**	9 (8, 10)	8·2 (7·6, 8·8)	36 (33, 38)	11·4 (10·6, 12·2)
**Bermuda**	5 (5, 6)	7·7 (7·1, 8·3)	13 (12, 14)	12·7 (11·6, 13·8)
**Cuba**	787 (732, 844)	7·3 (6·8, 7·9)	1940 (1793, 2094)	11·8 (10·9, 12·6)
**Dominica**	5 (4, 5)	7·2 (6·7, 7·8)	9 (9, 10)	11·3 (10·5, 12·2)
**Dominican Republic**	400 (371, 430)	8·7 (8·1, 9·4)	1148 (1064, 1237)	11·7 (10·8, 12·6)
**Grenada**	5 (5, 6)	7·9 (7·3, 8·5)	14 (13, 15)	10·7 (9·9, 11·5)
**Guyana**	46 (42, 49)	9·2 (8·5, 9·8)	72 (66, 78)	10·5 (9·7, 11·3)
**Haiti**	256 (237, 276)	6·4 (5·9, 6·9)	896 (817, 971)	10·4 (9·6, 11·3)
**Jamaica**	128 (119, 137)	7 (6·4, 7·5)	320 (297, 345)	11 (10·2, 11·8)
**Puerto Rico**	450 (419, 481)	12·3 (11·4, 13·1)	669 (617, 722)	12 (11·1, 12·9)

**Saint Lucia**	8 (7, 9)	8·3 (7·6, 8·9)	22 (21, 24)	10·8 (10, 11·6)
**Saint Vincent and the Grenadines**	6 (5, 6)	7·4 (6·8, 7·9)	14 (13, 15)	10·4 (9·6, 11·3)
**Suriname**	25 (23, 27)	8·4 (7·8, 9)	69 (64, 74)	11·4 (10·5, 12·3)
**Trinidad and Tobago**	75 (70, 81)	7·7 (7·1, 8·3)	193 (178, 209)	11·1 (10·3, 12)
**Virgin Islands**	8 (7, 9)	8·2 (7·6, 8·7)	17 (16, 19)	11·2 (10·3, 12)
**Tropical Latin America**	15938 (15021, 16805)	13·9 (13·1, 14·6)	32415 (30406, 34276)	13·4 (12·6, 14·2)
**Brazil**	15618 (14735, 16459)	13·9 (13·1, 14·7)	31503 (29562, 33302)	13·4 (12·6, 14·1)
**Paraguay**	320 (289, 352)	11·8 (10·6, 13)	912 (822, 1008)	15·3 (13·8, 16·9)
**Southern Latin America**	6918 (6485, 7389)	14·4 (13·5, 15·4)	16219 (15135, 17349)	21·3 (19·9, 22·8)
**Argentina**	4065 (3801, 4355)	12·4 (11·6, 13·3)	10096 (9388, 10801)	20·3 (18·9, 21·8)
**Chile**	2407 (2243, 2570)	21·1 (19·6, 22·5)	5214 (4843, 5602)	23·6 (21·9, 25·4)
**Uruguay**	446 (416, 477)	12·5 (11·6, 13·3)	909 (847, 973)	20·8 (19·4, 22·3)
**East Asia**	720200 (693693, 745196)	64·3 (61·9, 66·5)	1542217 (1473507, 1606200)	78·1 (74·9, 81·2)
**China**	691782 (666122, 715824)	65·1 (62·6, 67·3)	1477532 (1412281, 1538714)	78·7 (75·5, 81·7)
**North Korea**	7647 (7230, 8031)	39·6 (37·5, 41·5)	19621 (18481, 20713)	62·6 (59·2, 65·9)
**Taiwan (Province of China)**	8772 (8257, 9289)	47·2 (44·5, 49·8)	20220 (18770, 21568)	59·7 (55·7, 63·5)
**Southeast Asia**	41012 (38044, 44050)	12·1 (11·2, 13)	114434 (105240, 123878)	17·2 (15·8, 18·5)
**Cambodia**	725 (681, 768)	11·7 (11, 12·4)	2358 (2192, 2517)	17·2 (16·1, 18·4)
**Indonesia**	16828 (15469, 18187)	12·6 (11·6, 13·7)	30391 (27813, 33052)	12·1 (11·1, 13·1)
**Laos**	309 (289, 329)	11·5 (10·7, 12·2)	1003 (940, 1071)	18·2 (17·1, 19·4)
**Malaysia**	1674 (1519, 1828)	13 (11·8, 14·2)	7134 (6389, 7869)	24 (21·6, 26·5)
**Maldives**	15 (14, 16)	11·8 (11·1, 12·5)	89 (82, 95)	20 (18·6, 21·4)
**Mauritius**	129 (121, 137)	13·6 (12·8, 14·4)	277 (256, 298)	17·3 (16·1, 18·5)
**Myanmar**	3550 (3327, 3772)	12 (11·3, 12·7)	8666 (7966, 9309)	16·9 (15·5, 18)
**Philippines**	2748 (2578, 2926)	6·2 (5·8, 6·6)	12447 (11473, 13417)	14·1 (13, 15·1)

**Sri Lanka**	1736 (1628, 1847)	12·4 (11·7, 13·1)	4598 (4254, 4920)	18·7 (17·4, 19·9)
**Seychelles**	7 (7, 8)	11·7 (11, 12·4)	22 (20, 24)	19·2 (17·7, 20·5)
**Thailand**	5291 (4597, 6072)	11·3 (9·9, 12·9)	17189 (14645, 19851)	18·1 (15·5, 20·8)
**East Timor**	55 (51, 59)	11·6 (10·9, 12·3)	180 (167, 192)	19·4 (18·1, 20·8)
**Vietnam**	7891 (7363, 8370)	16·3 (15·2, 17·4)	29930 (27642, 32259)	29·3 (27·2, 31·5)
**Oceania**	831 (789, 873)	18·2 (17·3, 19)	2503 (2360, 2640)	25·3 (24, 26·6)
**American Samoa**	7 (7, 8)	20·7 (19·3, 21·9)	16 (15, 18)	31·6 (29·3, 33·7)
**Federated States of Micronesia**	15 (14, 16)	21·4 (20·3, 22·5)	26 (24, 27)	28·1 (26·4, 29·5)
**Fiji**	97 (92, 103)	16·5 (15·7, 17·3)	226 (213, 238)	25·5 (24·1, 26·9)
**Guam**	27 (26, 29)	23·7 (22·4, 24·9)	62 (58, 65)	34·5 (32·4, 36·4)
**Kiribati**	11 (10, 12)	20·1 (19·2, 21)	26 (25, 28)	27·5 (25·9, 28·9)
**Marshall Islands**	6 (5, 6)	20·9 (19·8, 21·9)	14 (13, 15)	28·8 (27·2, 30·4)
**Northern Mariana Islands**	10 (9, 10)	25·4 (24·1, 26·8)	20 (19, 22)	36·4 (34·3, 38·4)
**Papua New Guinea**	492 (465, 518)	17·3 (16·4, 18·1)	1695 (1596, 1793)	24·2 (22·9, 25·6)
**Samoa**	27 (25, 28)	23·4 (22·2, 24·7)	55 (52, 58)	33·4 (31·7, 35·3)
**Solomon Islands**	43 (41, 46)	20·2 (19·1, 21·2)	131 (123, 139)	27·3 (25·8, 28·7)
**Tonga**	16 (15, 17)	23·3 (22·1, 24·4)	29 (27, 30)	31·8 (29·9, 33·6)
**Vanuatu**	24 (23, 25)	23·6 (22·4, 24·8)	65 (62, 69)	29·4 (27·9, 31)
**North Africa and Middle East**	96065 (88650, 103330)	43·4 (40, 46·8)	218467 (200829, 235258)	43·8 (40·2, 47·2)
**Afghanistan**	2967 (2773, 3165)	39·3 (36·9, 41·8)	6795 (6359, 7272)	40·7 (38·1, 43·2)
**Algeria**	6646 (6250, 7037)	41·6 (39·1, 44)	15846 (14872, 16747)	43 (40·3, 45·6)
**Bahrain**	150 (141, 158)	44·3 (41·6, 46·9)	647 (602, 688)	44·1 (41·3, 46·8)
**Egypt**	18554 (15671, 21392)	51·8 (44, 60·2)	31515 (26117, 36859)	43·5 (36·1, 50·6)
**Iran**	13815 (12903, 14686)	36·5 (34, 38·8)	32053 (29735, 34313)	39·3 (36·5, 42·1)
**Iraq**	3937 (3699, 4164)	37·8 (35·5, 40)	11540 (10837, 12294)	37·6 (35·2, 40)

**Jordan**	904 (845, 960)	43 (40·4, 45·7)	3572 (3331, 3797)	44·8 (41·8, 47·5)
**Kuwait**	481 (452, 511)	38·7 (36·4, 40·9)	1628 (1514, 1742)	43·2 (40·4, 46·1)
**Lebanon**	1187 (1119, 1254)	43·1 (40·5, 45·6)	3499 (3290, 3694)	49·8 (46·8, 52·7)
**Libya**	1047 (987, 1110)	41·5 (39·1, 43·9)	2445 (2284, 2596)	42·4 (39·6, 45·1)
**Morocco**	6944 (6535, 7362)	39·3 (36·9, 41·8)	13728 (12839, 14592)	40·2 (37·5, 42·7)
**Palestine**	476 (447, 505)	41·3 (38·8, 43·7)	1393 (1304, 1479)	42·6 (39·8, 45·2)
**Oman**	533 (500, 564)	47 (44, 49·7)	1596 (1491, 1698)	44·8 (41·9, 47·5)
**Qatar**	140 (131, 150)	47·1 (43·9, 50·1)	1120 (1042, 1206)	49·3 (45·9, 52·8)
**Saudi Arabia**	2605 (2186, 3027)	28·6 (23·9, 33·8)	7412 (6079, 8935)	27·7 (22·8, 32·7)
**Sudan**	5257 (4935, 5578)	43·3 (40·7, 45·9)	10655 (10010, 11291)	43·9 (41·2, 46·7)
**Syria**	2888 (2724, 3052)	39·9 (37·7, 42·1)	6362 (5949, 6774)	43 (40·2, 45·8)
**Tunisia**	2017 (1547, 2536)	33·6 (26·1, 41·4)	4261 (3193, 5439)	34 (25·9, 43)
**Turkey**	22207 (20512, 23899)	52 (47·9, 56)	51007 (46640, 55218)	57·9 (53, 62·7)
**United Arab Emirates**	539 (504, 576)	44·7 (42·1, 47·2)	4309 (3984, 4639)	46·4 (43·5, 49·3)
**Yemen**	2711 (2541, 2877)	38·5 (36·1, 40·9)	6880 (6425, 7320)	38·3 (35·7, 40·9)
**South Asia**	109207 (103112, 115038)	13·1 (12·4, 13·8)	246927 (231917, 260786)	15·1 (14·3, 16)
**Bangladesh**	10389 (9745, 11049)	14·5 (13·7, 15·4)	20525 (19252, 21768)	14·2 (13·3, 15)
**Bhutan**	58 (54, 62)	15·4 (14·5, 16·3)	131 (122, 140)	15·1 (14·1, 16)
**India**	87717 (82943, 92191)	13 (12·3, 13·6)	203150 (191776, 213953)	15·7 (14·8, 16·5)
**Nepal**	1390 (1215, 1574)	10·2 (9, 11·5)	2567 (2240, 2910)	9·9 (8·7, 11·2)
**Pakistan**	9652 (8615, 10825)	13·2 (11·8, 14·8)	20554 (18013, 23231)	12·8 (11·3, 14·4)
**Southern sub-Saharan Africa**	7225 (6718, 7722)	20·3 (18·9, 21·7)	9870 (9142, 10568)	14·7 (13·7, 15·7)
**Botswana**	135 (122, 150)	17·3 (15·6, 19·1)	293 (260, 324)	15·8 (14·1, 17·5)
**Lesotho**	224 (202, 246)	18·5 (16·8, 20·3)	225 (201, 249)	14·9 (13·4, 16·4)
**Namibia**	124 (111, 137)	13·7 (12·3, 15·2)	278 (248, 311)	15·7 (14, 17·5)

**South Africa**	5344 (5007, 5669)	20·1 (18·8, 21·4)	7222 (6757, 7672)	14·1 (13·2, 14·9)
**Swaziland**	76 (69, 84)	17·9 (16·1, 19·6)	120 (108, 132)	15·5 (13·9, 17·1)
**Zimbabwe**	1321 (1194, 1444)	23·4 (21·1, 25·6)	1732 (1552, 1913)	18·3 (16·5, 20·2)
**Western sub-Saharan Africa**	68435 (63946, 72384)	60·2 (56·5, 63·9)	148929 (138802, 158243)	60·2 (56, 64·1)
**Benin**	1720 (1614, 1827)	66·1 (62, 70·1)	4120 (3825, 4389)	63·5 (59, 67·9)
**Burkina Faso**	4225 (3977, 4471)	77·6 (73·2, 81·9)	8020 (7450, 8549)	67·5 (63·1, 72·2)
**Cameroon**	3397 (3167, 3609)	57·7 (53·9, 61·5)	8983 (8362, 9598)	55·9 (52·1, 60·1)
**Cape Verde**	155 (146, 165)	66·4 (62·3, 70·4)	303 (282, 323)	63·4 (58·9, 67·8)
**Chad**	2549 (2393, 2693)	73·6 (69·3, 77·6)	5259 (4896, 5585)	70·6 (65·9, 75)
**Côte d'Ivoire**	3741 (3495, 3982)	59·4 (55·6, 63·1)	8697 (8078, 9278)	58·2 (54·1, 62)
**The Gambia**	353 (330, 373)	68·3 (64·2, 72)	823 (769, 878)	65·6 (61·2, 70)
**Ghana**	4533 (4226, 4826)	52·2 (48·9, 55·6)	10922 (10145, 11722)	53·2 (49·3, 57)
**Guinea**	2642 (2477, 2792)	67·1 (63·1, 70·8)	4503 (4200, 4804)	65·5 (60·9, 70)
**Guinea-Bissau**	356 (334, 377)	65·3 (61·2, 69)	642 (599, 685)	61·9 (58, 65·9)
**Liberia**	907 (851, 963)	70 (65·9, 74·2)	2063 (1928, 2199)	73·4 (68·4, 78·2)
**Mali**	4111 (3862, 4346)	80·6 (76·1, 85·2)	9026 (8446, 9634)	82·9 (77·7, 88·3)
**Mauritania**	787 (737, 834)	62·5 (58·5, 66·3)	1607 (1498, 1710)	64·5 (60·2, 68·9)
**Niger**	3253 (3058, 3436)	79·7 (75·2, 84·1)	8578 (8029, 9120)	83·3 (78·1, 88·4)
**Nigeria**	29542 (27376, 31531)	53·1 (49·2, 56·9)	62685 (57605, 67187)	54·5 (50·4, 58·7)
**São Tomé and Príncipe**	58 (54, 61)	77·4 (72·6, 82)	97 (91, 104)	72·5 (67·5, 77·6)
**Senegal**	2897 (2720, 3059)	68·5 (64·6, 72·2)	6397 (5977, 6816)	69·3 (64·8, 73·8)
**Sierra Leone**	2003 (1891, 2118)	83·9 (79·2, 88·8)	3107 (2902, 3308)	66·6 (62·1, 71·1)
**Togo**	1204 (1126, 1277)	65·5 (61·4, 69·2)	3095 (2864, 3303)	64·5 (59·8, 69)
**Eastern sub-Saharan Africa**	28114 (25673, 30566)	28·2 (25·7, 30·7)	59036 (53788, 64274)	27·5 (25, 30)
**Burundi**	692 (627, 760)	23·4 (21·2, 25·7)	1292 (1165, 1419)	21·9 (19·8, 24·1)

**Comoros**	70 (63, 76)	26·8 (24·3, 29·4)	142 (128, 155)	26 (23·4, 28·4)
**Djibouti**	67 (60, 73)	26·7 (24·2, 29·2)	225 (202, 247)	28 (25·4, 30·8)
**Eritrea**	338 (306, 373)	23·8 (21·6, 26·1)	747 (674, 825)	21·9 (19·9, 24·1)
**Ethiopia**	7995 (7313, 8661)	30 (27·4, 32·5)	17205 (15788, 18670)	31·3 (28·5, 33·9)
**Kenya**	4669 (4253, 5064)	42·8 (39, 46·5)	10703 (9650, 11700)	38 (34·4, 41·6)
**Madagascar**	1772 (1612, 1937)	26·6 (24·2, 29·3)	3707 (3371, 4080)	25 (22·8, 27·4)
**Malawi**	1494 (1351, 1636)	28·4 (25·7, 31·2)	2470 (2249, 2699)	25·5 (23·1, 28·2)
**Mozambique**	2120 (1926, 2318)	26·2 (23·8, 28·6)	4008 (3630, 4391)	26 (23·6, 28·5)
**Rwanda**	597 (468, 739)	15·6 (12·3, 19·2)	976 (757, 1219)	12·8 (10, 15·9)
**Somalia**	904 (817, 991)	23·7 (21·4, 26)	2292 (2058, 2534)	25·3 (22·8, 27·9)
**South Sudan**	942 (857, 1027)	29 (26·4, 31·7)	1557 (1406, 1714)	29·5 (26·6, 32·5)
**Tanzania**	3406 (3103, 3721)	24·3 (22, 26·6)	7366 (6675, 8068)	23·6 (21·4, 26)
**Uganda**	2023 (1844, 2201)	23·4 (21·3, 25·7)	4206 (3825, 4618)	22 (19·9, 24·1)
**Zambia**	1010 (921, 1102)	26·1 (23·7, 28·5)	2103 (1898, 2308)	22·9 (20·7, 25·1)
**Central sub-Saharan Africa**	12904 (11858, 13899)	41·3 (38, 44·6)	29627 (26916, 32127)	40·9 (37·4, 44·5)
**Angola**	2522 (2317, 2733)	44·2 (40·8, 48)	6167 (5629, 6717)	39·6 (36·1, 43·1)
**Central African Republic**	544 (497, 591)	34·2 (31·4, 37·1)	1047 (948, 1144)	35·4 (32·2, 38·5)
**Congo**	465 (424, 503)	32·8 (30, 35·6)	1198 (1090, 1308)	34·6 (31·6, 37·8)
**DR Congo**	9049 (8297, 9748)	41·9 (38·6, 45·3)	20493 (18590, 22303)	42·4 (38·7, 46·1)
**Equatorial Guinea**	92 (84, 100)	36·5 (33·5, 39·7)	279 (252, 305)	38·2 (34·5, 41·6)
**Gabon**	232 (211, 251)	35 (31·8, 37·9)	443 (400, 486)	34·1 (31·1, 37·3)

Appendix Table 17: Number of prevalent cases and age-standardised prevalence rates of decompensated cirrhosis due to hepatitis C per 100 000 in 1990 and 2017 for both sexes by location

	1990		2017	
	**Counts (95% UI)**	**Rate (95% UI)**	**Counts (95% UI)**	**Rate (95% UI)**
**Global**	1233294 (1162816, 1300880)	27·2 (25·7, 28·7)	2641473 (2487047, 2806144)	32·5 (30·6, 34·5)
**High-income North America**	104668 (100354, 109176)	31·8 (30·4, 33·1)	187598 (178813, 196786)	36·9 (35·2, 38·6)
**Canada**	8587 (8058, 9128)	26·8 (25, 28·4)	17043 (15845, 18199)	31·6 (29·5, 33·7)
**Greenland**	11 (10, 12)	21·8 (20·4, 23·2)	19 (18, 20)	27 (25·2, 28·8)
**USA**	96068 (92156, 100147)	32·3 (31, 33·7)	170532 (162684, 178761)	37·5 (35·9, 39·2)
**Australasia**	3292 (3062, 3521)	14·6 (13·5, 15·6)	8044 (7446, 8608)	21 (19·4, 22·4)
**Australia**	2729 (2517, 2938)	14·5 (13·3, 15·6)	6873 (6299, 7393)	21·2 (19·5, 22·9)
**New Zealand**	564 (535, 594)	15·1 (14·3, 15·9)	1171 (1107, 1236)	19·6 (18·5, 20·7)
**High-income Asia-Pacific**	229080 (221268, 237380)	110·5 (106·9, 114·4)	337525 (324277, 352397)	107·3 (103·3, 111·9)
**Brunei**	57 (52, 61)	34·1 (31·4, 36·8)	207 (188, 224)	48·9 (44·9, 52·8)
**Japan**	219137 (212261, 226922)	132·7 (128·5, 137·2)	317879 (306205, 331418)	145·7 (140·5, 151·6)
**Singapore**	456 (382, 533)	15·4 (13, 18·1)	1725 (1431, 2033)	24 (20·1, 28·2)
**South Korea**	9430 (8042, 10741)	24·5 (21·1, 27·9)	17715 (15420, 20161)	22 (19·4, 24·9)
**Western Europe**	146385 (135507, 157195)	29·7 (27·4, 31·9)	249319 (231759, 267818)	39·3 (36·4, 42·1)
**Andorra**	13 (12, 14)	20·6 (18·9, 22·3)	38 (35, 41)	31·3 (28·9, 33·7)
**Austria**	3185 (2939, 3430)	32·6 (30·1, 35·3)	4773 (4399, 5156)	36·9 (33·9, 39·8)
**Belgium**	3093 (2530, 3686)	24·2 (19·8, 28·9)	5957 (4710, 7200)	37 (29·7, 44·4)
**Cyprus**	109 (101, 117)	13·3 (12·3, 14·3)	347 (321, 373)	20·6 (19, 22·2)
**Denmark**	1197 (1108, 1283)	18·5 (17·1, 19·9)	2442 (2260, 2637)	30·2 (27·9, 32·6)
**Finland**	1184 (1094, 1270)	18·8 (17·4, 20·2)	2615 (2408, 2812)	32·8 (30·2, 35·4)
**France**	15150 (13978, 16344)	21·7 (19·9, 23·4)	25306 (23509, 27162)	27·6 (25·5, 29·6)

**Germany**	23583 (21698, 25356)	22·2 (20·4, 24)	41706 (38541, 45033)	32·9 (30·4, 35·5)
**Greece**	2172 (2011, 2323)	16·3 (15·1, 17·4)	4085 (3794, 4394)	26·1 (24·1, 28·2)
**Iceland**	19 (13, 26)	7·4 (5, 10·1)	65 (43, 90)	14·9 (10·1, 20·6)
**Ireland**	541 (500, 581)	14·7 (13·5, 15·8)	1718 (1583, 1860)	27·5 (25·3, 29·8)
**Israel**	879 (819, 937)	18·8 (17·5, 20·1)	3040 (2821, 3258)	31·5 (29·1, 33·9)
**Italy**	64411 (59813, 68800)	83·7 (77·8, 89·3)	99945 (94033, 106188)	104·7 (98·5, 111·3)
**Luxembourg**	143 (132, 154)	28·9 (26·6, 31·1)	308 (283, 336)	37·7 (34·7, 40·9)
**Malta**	55 (51, 59)	12·9 (11·9, 13·9)	129 (120, 139)	20·2 (18·5, 21·8)
**Netherlands**	2720 (2525, 2926)	15·1 (14, 16·2)	6399 (5914, 6882)	26·1 (24·1, 28)
**Norway**	959 (907, 1012)	18·9 (17·8, 20)	1714 (1618, 1810)	24·2 (22·8, 25·6)
**Portugal**	2471 (2259, 2719)	19·8 (18, 21·8)	3894 (3565, 4235)	24 (22, 26·1)
**Spain**	16512 (13606, 19507)	34·2 (28·2, 40·2)	28657 (23668, 33778)	41·5 (34·4, 48·6)
**Sweden**	1488 (1289, 1692)	13·6 (11·7, 15·5)	2975 (2587, 3405)	21·7 (18·8, 24·9)
**Switzerland**	1490 (1375, 1591)	16·7 (15·4, 17·9)	2960 (2724, 3187)	23·5 (21·6, 25·3)
**United Kingdom**	4868 (4467, 5271)	7 (6·3, 7·6)	9989 (9117, 10926)	11·1 (10·1, 12·1)
**Eastern Europe**	99439 (93688, 104762)	36·9 (34·8, 38·9)	201797 (189966, 213261)	70 (65·7, 74)
**Belarus**	4200 (3791, 4590)	34·6 (31·4, 37·8)	8428 (7613, 9250)	63·7 (57·4, 69·7)
**Estonia**	731 (664, 797)	39·1 (35·6, 42·4)	1164 (1059, 1268)	63·8 (57·7, 69·9)
**Latvia**	1139 (1039, 1249)	34·7 (31·6, 37·9)	1638 (1491, 1794)	59·6 (53·7, 65·5)
**Lithuania**	1733 (1579, 1889)	40·4 (36·8, 44·1)	2841 (2577, 3112)	71 (64·1, 77·6)
**Moldova**	4666 (4210, 5136)	97·3 (88·2, 106·9)	4461 (4042, 4894)	86·1 (77·9, 93·8)
**Russia**	60998 (57824, 64163)	34·4 (32·6, 36·2)	136707 (128778, 144492)	68·6 (64·5, 72·5)
**Ukraine**	25972 (24351, 27361)	39·7 (37·3, 41·8)	46558 (43680, 49404)	75·5 (70·7, 80·2)
**Central Europe**	40837 (36565, 44944)	28 (25·1, 30·7)	66030 (59113, 72567)	38·9 (35, 42·7)
**Albania**	681 (609, 751)	26·4 (23·6, 29·1)	1572 (1408, 1745)	42·4 (38·1, 46·9)

**Bosnia and Herzegovina**	1382 (1227, 1531)	28·8 (25·6, 31·7)	2106 (1873, 2343)	41·3 (36·9, 45·8)
**Bulgaria**	2721 (2423, 3019)	23·1 (20·7, 25·4)	4001 (3576, 4439)	36·4 (32·8, 40·5)
**Croatia**	2111 (1864, 2364)	33·2 (29·5, 37·1)	2256 (2013, 2516)	34·3 (30·7, 38)
**Czech Republic**	3019 (2707, 3323)	23·9 (21·4, 26·3)	5363 (4819, 5942)	33·6 (30·1, 37·2)
**Hungary**	5451 (4823, 6063)	40·5 (35·9, 45)	5124 (4547, 5710)	34·5 (30·6, 38·2)
**Macedonia**	503 (448, 558)	24·2 (21·6, 26·6)	1185 (1046, 1322)	39 (34·5, 43·4)
**Montenegro**	156 (139, 173)	23·5 (21·1, 26)	317 (281, 350)	36·8 (32·8, 40·7)
**Poland**	10097 (9060, 11144)	23·1 (20·7, 25·5)	22605 (20110, 24926)	40·7 (36·2, 45·1)
**Romania**	9114 (8088, 10172)	32·4 (28·9, 35·9)	12679 (11279, 14027)	42·7 (38·1, 47·3)
**Serbia**	2348 (2074, 2613)	20 (17·8, 22·1)	3559 (3182, 3957)	28·1 (25·2, 31·3)
**Slovakia**	2339 (2089, 2582)	40 (35·8, 44·3)	4076 (3644, 4516)	51·9 (46·2, 57·3)
**Slovenia**	915 (808, 1014)	37·5 (33·2, 41·5)	1188 (1061, 1315)	36·8 (33·1, 40·6)
**Central Asia**	15273 (14227, 16294)	27·8 (26, 29·5)	39001 (36098, 41748)	44·8 (41·7, 47·7)
**Armenia**	799 (738, 854)	25·3 (23·4, 27)	1713 (1595, 1833)	44·6 (41·6, 47·7)
**Azerbaijan**	1671 (1553, 1792)	28·3 (26·3, 30·3)	4765 (4393, 5149)	43·9 (40·6, 47·1)
**Georgia**	1783 (1651, 1916)	28·5 (26·4, 30·5)	2148 (1990, 2303)	43·2 (40·1, 46·4)
**Kazakhstan**	3267 (3028, 3494)	21·9 (20·3, 23·4)	8024 (7416, 8622)	43·1 (39·9, 46·2)
**Kyrgyzstan**	1086 (1009, 1161)	31·9 (29·6, 34·1)	2582 (2376, 2779)	47·5 (44·1, 50·9)
**Mongolia**	403 (374, 431)	30·2 (28·2, 32·3)	1195 (1098, 1292)	40·5 (37·4, 43·4)
**Tajikistan**	964 (899, 1034)	28·1 (26·2, 30·2)	3019 (2776, 3238)	43·3 (40·3, 46·3)
**Turkmenistan**	794 (735, 851)	32·5 (30·2, 34·7)	2303 (2117, 2472)	49 (45·2, 52·3)
**Uzbekistan**	4507 (4167, 4830)	32·3 (29·9, 34·5)	13254 (12216, 14314)	46·1 (42·8, 49·5)
**Central Latin America**	48117 (45284, 50656)	44·8 (42·3, 47·2)	154965 (145109, 165098)	62·5 (58·7, 66·5)
**Colombia**	5418 (4975, 5852)	23·4 (21·5, 25·3)	26370 (24296, 28735)	49·2 (45·4, 53·6)
**Costa Rica**	697 (641, 752)	32·4 (29·8, 35·1)	2923 (2681, 3196)	57·6 (53, 62·7)

**El Salvador**	1193 (1099, 1287)	34·4 (31·7, 37·1)	3277 (3018, 3565)	57·2 (52·7, 62·3)
**Guatemala**	1954 (1813, 2103)	40·7 (37·8, 43·7)	7559 (6992, 8186)	61 (56·3, 66·1)
**Honduras**	1054 (975, 1138)	40·1 (37·1, 43·3)	4203 (3863, 4561)	60·9 (55·9, 66·1)
**Mexico**	32822 (31095, 34371)	59·7 (56·7, 62·5)	87890 (82953, 92628)	70·8 (67·1, 74·6)
**Nicaragua**	744 (688, 806)	36·1 (33·5, 39·2)	3133 (2875, 3402)	60·1 (55·2, 65·3)
**Panama**	539 (498, 580)	30·4 (28·1, 32·8)	2263 (2083, 2450)	56·3 (51·9, 60·9)
**Venezuela**	3697 (3413, 3975)	29·3 (27, 31·5)	17347 (15901, 18920)	56·1 (51·4, 61)
**Andean Latin America**	2874 (2589, 3165)	11·7 (10·5, 12·9)	13437 (12157, 14798)	23·9 (21·6, 26·4)
**Bolivia**	409 (367, 452)	10·5 (9·4, 11·6)	2133 (1923, 2360)	22·4 (20·1, 24·8)
**Ecuador**	789 (706, 872)	12·2 (11, 13·6)	3915 (3528, 4316)	25·5 (23, 28·1)
**Peru**	1676 (1501, 1855)	11·8 (10·5, 13·1)	7389 (6691, 8184)	23·5 (21·2, 26)
**Caribbean**	4748 (4492, 5010)	16·7 (15·7, 17·6)	13503 (12709, 14348)	26·7 (25·1, 28·3)
**Antigua and Barbuda**	9 (8, 9)	17 (16, 18·1)	28 (26, 30)	26·8 (25·1, 28·5)
**The Bahamas**	32 (30, 34)	16·9 (15·8, 18)	105 (98, 112)	25·4 (23·9, 27·1)
**Barbados**	41 (38, 43)	15·5 (14·5, 16·5)	111 (104, 118)	26·2 (24·6, 27·8)
**Belize**	18 (17, 19)	16·8 (15·8, 17·9)	85 (79, 91)	27·1 (25·4, 28·9)
**Bermuda**	11 (10, 11)	15·9 (14·8, 17·1)	28 (26, 30)	26·5 (24·7, 28·3)
**Cuba**	1607 (1514, 1703)	15 (14·2, 16)	4778 (4469, 5116)	28·6 (26·8, 30·5)
**Dominica**	10 (9, 10)	15 (14·2, 15·9)	22 (21, 23)	26 (24·4, 27·7)
**Dominican Republic**	816 (764, 866)	17·9 (16·8, 19·1)	2669 (2499, 2861)	27·1 (25·4, 29)
**Grenada**	11 (10, 11)	16·2 (15·2, 17·2)	34 (32, 36)	25·7 (24·1, 27·4)
**Guyana**	91 (85, 97)	18·5 (17·4, 19·7)	176 (164, 189)	25·4 (23·7, 27·1)
**Haiti**	520 (485, 555)	13 (12·1, 13·9)	1845 (1725, 1964)	21·6 (20·2, 23)
**Jamaica**	258 (243, 273)	14·3 (13·4, 15·1)	777 (726, 827)	26·5 (24·8, 28·2)
**Puerto Rico**	915 (859, 972)	25·1 (23·5, 26·6)	1627 (1526, 1732)	29·2 (27·4, 31·2)

**Saint Lucia**	16 (15, 17)	17·2 (16·1, 18·2)	54 (51, 58)	25·9 (24·3, 27·6)
**Saint Vincent and the Grenadines**	12 (11, 12)	15·1 (14·2, 16·1)	33 (31, 35)	24·8 (23·1, 26·6)
**Suriname**	51 (48, 54)	17·3 (16·3, 18·4)	158 (147, 169)	25·8 (24·2, 27·6)
**Trinidad and Tobago**	152 (142, 161)	15·7 (14·8, 16·7)	446 (416, 476)	25·4 (23·8, 27·2)
**Virgin Islands**	17 (16, 18)	17 (15·9, 18·1)	42 (39, 45)	26·7 (24·9, 28·6)
**Tropical Latin America**	25946 (24671, 27161)	22·7 (21·6, 23·7)	61538 (58422, 64711)	25·4 (24·1, 26·6)
**Brazil**	25469 (24227, 26639)	22·8 (21·7, 23·8)	59963 (56931, 63046)	25·3 (24·1, 26·6)
**Paraguay**	477 (437, 518)	17·7 (16·2, 19·2)	1575 (1436, 1721)	26·4 (24·1, 28·7)
**Southern Latin America**	13441 (12710, 14157)	28·1 (26·6, 29·6)	35954 (33914, 38014)	47·2 (44·5, 49·9)
**Argentina**	7874 (7429, 8349)	24 (22·7, 25·4)	22194 (20857, 23590)	44·8 (42·1, 47·6)
**Chile**	4702 (4422, 4971)	41·5 (39, 44)	11732 (10986, 12432)	52·5 (49·3, 55·6)
**Uruguay**	865 (816, 911)	24·2 (22·8, 25·5)	2026 (1906, 2142)	46·5 (43·8, 49·1)
**East Asia**	212886 (197171, 228983)	19·2 (17·8, 20·7)	545950 (503175, 589606)	27·1 (25·2, 29·2)
**China**	202481 (187448, 217798)	19·2 (17·8, 20·7)	517360 (477117, 558840)	27 (25·1, 29·1)
**North Korea**	2644 (2436, 2865)	13·6 (12·6, 14·7)	7795 (7197, 8418)	24·6 (22·8, 26·4)
**Taiwan (Province of China)**	4215 (3750, 4670)	22·8 (20·2, 25·2)	12001 (10622, 13462)	34·8 (31, 38·9)
**Southeast Asia**	40629 (37734, 43361)	12 (11·2, 12·8)	114082 (105450, 122791)	16·9 (15·7, 18·2)
**Cambodia**	549 (513, 586)	8·9 (8·4, 9·5)	2083 (1932, 2233)	15·2 (14·1, 16·2)
**Indonesia**	23841 (22255, 25462)	17·9 (16·7, 19)	46713 (43519, 49848)	18·4 (17·3, 19·6)
**Laos**	215 (199, 230)	8 (7·4, 8·6)	768 (713, 824)	13·9 (13, 14·9)
**Malaysia**	674 (561, 794)	5·3 (4·4, 6·2)	3316 (2770, 3879)	11·1 (9·4, 13)
**Maldives**	11 (10, 12)	8·6 (8, 9·2)	76 (70, 82)	17·3 (16·1, 18·5)
**Mauritius**	96 (89, 103)	10·2 (9·5, 11)	248 (230, 266)	15·2 (14·1, 16·3)
**Myanmar**	2560 (2383, 2740)	8·7 (8·1, 9·3)	7502 (6959, 8046)	14·5 (13·5, 15·5)
**Philippines**	1923 (1781, 2067)	4·4 (4·1, 4·7)	10381 (9591, 11177)	11·7 (10·9, 12·5)

**Sri Lanka**	1312 (1218, 1404)	9·4 (8·8, 10·1)	4175 (3887, 4462)	16·8 (15·7, 17·9)
**Seychelles**	5 (5, 6)	8·9 (8·3, 9·5)	20 (18, 21)	16·9 (15·6, 18·2)
**Thailand**	3912 (3293, 4549)	8·4 (7, 9·7)	14530 (12044, 17060)	15·1 (12·6, 17·5)
**East Timor**	40 (37, 43)	8·4 (7·8, 9)	144 (135, 154)	15·7 (14·6, 16·8)
**Vietnam**	5438 (5065, 5820)	11·4 (10·6, 12·2)	23975 (22013, 25861)	23·2 (21·5, 25)
**Oceania**	378 (353, 404)	8·4 (7·9, 8·9)	1332 (1238, 1422)	13·5 (12·7, 14·4)
**American Samoa**	3 (3, 4)	9·8 (9·1, 10·4)	8 (7, 9)	15·5 (14·3, 16·7)
**Federated States of Micronesia**	7 (6, 7)	9·6 (8·9, 10·2)	14 (13, 15)	15·3 (14·3, 16·4)
**Fiji**	46 (43, 50)	8 (7·5, 8·5)	131 (123, 141)	14·7 (13·9, 15·8)
**Guam**	13 (12, 14)	11·6 (10·8, 12·3)	31 (29, 34)	17·3 (16·1, 18·5)
**Kiribati**	5 (4, 5)	8·8 (8·2, 9·3)	13 (12, 14)	14 (13·1, 15)
**Marshall Islands**	3 (2, 3)	9·5 (8·9, 10·1)	7 (7, 8)	15 (14·1, 16)
**Northern Mariana Islands**	5 (4, 5)	12·3 (11·5, 13·1)	10 (10, 11)	18 (16·8, 19·3)
**Papua New Guinea**	223 (208, 240)	7·9 (7·4, 8·5)	896 (828, 960)	12·9 (12, 13·8)
**Samoa**	12 (11, 13)	10·6 (10, 11·3)	28 (26, 30)	17·3 (16·2, 18·4)
**Solomon Islands**	19 (18, 20)	8·9 (8·3, 9·5)	69 (64, 74)	14·5 (13·6, 15·4)
**Tonga**	7 (7, 8)	10·6 (10, 11·3)	16 (15, 17)	17·8 (16·6, 19·1)
**Vanuatu**	10 (9, 11)	10 (9·3, 10·6)	33 (31, 35)	15 (14, 16·1)
**North Africa and Middle East**	108100 (100819, 115152)	48·9 (45·6, 52·2)	291557 (272067, 309328)	57·7 (53·9, 61·4)
**Afghanistan**	3448 (3261, 3643)	44·7 (42·4, 47)	8402 (7896, 8948)	51·3 (48·6, 54·2)
**Algeria**	8201 (7786, 8632)	52 (49·6, 54·8)	23715 (22557, 24940)	64·5 (61·3, 67·8)
**Bahrain**	177 (168, 187)	55·3 (52·7, 58·1)	967 (912, 1025)	66·1 (62·8, 69·6)
**Egypt**	28227 (25215, 31412)	79 (70·1, 87·8)	57722 (51379, 63868)	79·1 (70·4, 87·4)
**Iran**	17915 (16880, 19012)	47·9 (45·5, 50·6)	48896 (46322, 51596)	60·8 (57·6, 64)
**Iraq**	4746 (4496, 5017)	46·4 (44·1, 48·9)	15763 (14906, 16652)	51·8 (49·2, 54·5)

**Jordan**	1095 (1033, 1160)	53 (50·2, 55·9)	5354 (5043, 5664)	67·5 (63·9, 71·1)
**Kuwait**	569 (537, 603)	48 (45·7, 50·5)	2443 (2290, 2591)	65·5 (62·1, 69·2)
**Lebanon**	1356 (1286, 1429)	49·5 (47, 52)	4510 (4290, 4754)	65·3 (62, 69)
**Libya**	1299 (1233, 1371)	52·2 (49·6, 55)	3723 (3530, 3936)	64·9 (61·4, 68·3)
**Morocco**	8372 (7975, 8816)	48 (45·7, 50·6)	20726 (19600, 21843)	60·2 (57·1, 63·5)
**Palestine**	580 (549, 611)	51·2 (48·7, 53·8)	2012 (1910, 2125)	62 (58·9, 65·3)
**Oman**	596 (565, 627)	55·1 (52·3, 57·9)	2270 (2144, 2398)	66·9 (63·6, 70·4)
**Qatar**	161 (151, 172)	57·9 (54·8, 61·4)	1565 (1470, 1665)	71·7 (67·9, 75·9)
**Saudi Arabia**	7158 (6588, 7715)	79·9 (73·4, 86·2)	25301 (23103, 27369)	95·8 (87·8, 103·5)
**Sudan**	6015 (5703, 6353)	50·3 (47·8, 52·9)	14419 (13673, 15182)	60 (57, 63·4)
**Syria**	3543 (3369, 3735)	49·5 (47·2, 52·1)	9495 (8974, 10021)	63·5 (60·3, 66·9)
**Tunisia**	2532 (2059, 2999)	41·7 (33·7, 49·2)	6442 (5170, 7571)	51 (41·1, 59·9)
**Turkey**	8085 (6956, 9330)	19 (16·3, 22)	21554 (18429, 24752)	24·3 (20·8, 27·8)
**United Arab Emirates**	610 (575, 647)	54·7 (51·9, 57·6)	5978 (5558, 6401)	67·3 (63·5, 70·9)
**Yemen**	3346 (3164, 3529)	48·3 (45·8, 50·9)	10025 (9485, 10599)	56·9 (53·7, 60·1)
**South Asia**	66134 (61375, 70786)	8 (7·5, 8·6)	158235 (146704, 169217)	9·7 (9·1, 10·4)
**Bangladesh**	7082 (6569, 7571)	10 (9·4, 10·6)	16743 (15692, 17801)	11·6 (10·9, 12·3)
**Bhutan**	38 (35, 41)	10·1 (9·5, 10·8)	103 (95, 110)	12 (11·3, 12·8)
**India**	44667 (41431, 47892)	6·7 (6·2, 7·1)	105478 (97726, 112974)	8·2 (7·6, 8·8)
**Nepal**	650 (544, 760)	4·8 (4, 5·6)	1392 (1173, 1618)	5·4 (4·5, 6·2)
**Pakistan**	13698 (12309, 15090)	18·8 (16·9, 20·7)	34520 (31322, 37873)	21·6 (19·6, 23·6)
**Southern sub-Saharan Africa**	6619 (6144, 7066)	18·8 (17·6, 20·1)	11467 (10668, 12250)	17·2 (16·1, 18·3)
**Botswana**	132 (118, 146)	17·1 (15·4, 18·8)	357 (320, 393)	19·4 (17·4, 21·3)
**Lesotho**	203 (182, 223)	16·9 (15·2, 18·7)	261 (234, 288)	17·5 (15·8, 19·3)
**Namibia**	129 (116, 141)	14·4 (13, 15·8)	300 (269, 330)	17·1 (15·4, 18·7)

**South Africa**	4987 (4671, 5300)	19 (17·8, 20·2)	8576 (8032, 9099)	16·7 (15·7, 17·7)
**Swaziland**	73 (65, 81)	17·3 (15·6, 19·1)	142 (127, 156)	18·6 (16·8, 20·4)
**Zimbabwe**	1096 (990, 1211)	19·6 (17·8, 21·6)	1832 (1640, 2011)	19·6 (17·6, 21·5)
**Western sub-Saharan Africa**	18545 (16352, 20795)	16·6 (14·7, 18·6)	42619 (37503, 47839)	17·4 (15·4, 19·5)
**Benin**	426 (376, 476)	16·9 (15, 18·9)	1157 (1013, 1306)	18·2 (16·1, 20·5)
**Burkina Faso**	952 (841, 1078)	17·7 (15·7, 20)	2223 (1949, 2498)	19 (16·7, 21·4)
**Cameroon**	935 (819, 1059)	16·1 (14·2, 18·1)	2684 (2348, 3029)	17 (15, 19·1)
**Cape Verde**	39 (35, 44)	17·5 (15·6, 19·7)	97 (85, 109)	20·4 (18·1, 23)
**Chad**	588 (521, 657)	17·4 (15·4, 19·6)	1273 (1107, 1440)	17·4 (15·4, 19·7)
**Côte d'Ivoire**	971 (848, 1094)	15·7 (13·9, 17·6)	2502 (2194, 2810)	16·9 (15, 19·1)
**The Gambia**	85 (75, 96)	17 (15, 19·1)	229 (201, 260)	18·7 (16·6, 21·2)
**Ghana**	1332 (1163, 1491)	15·5 (13·7, 17·4)	3572 (3137, 4012)	17·5 (15·5, 19·6)
**Guinea**	655 (577, 733)	16·8 (14·8, 18·9)	1159 (1009, 1306)	17·1 (15·1, 19·4)
**Guinea-Bissau**	85 (75, 96)	15·9 (14·1, 17·9)	159 (139, 179)	15·6 (13·7, 17·6)
**Liberia**	230 (204, 258)	17·8 (15·8, 20·1)	509 (441, 579)	18·2 (15·9, 20·7)
**Mali**	1011 (893, 1142)	19·9 (17·6, 22·5)	2306 (2018, 2592)	21·5 (18·9, 24·1)
**Mauritania**	210 (185, 236)	17 (15·1, 19·1)	467 (412, 526)	19 (16·8, 21·3)
**Niger**	757 (660, 853)	18·8 (16·6, 21·2)	1997 (1744, 2260)	19·7 (17·4, 22·3)
**Nigeria**	8832 (7743, 9935)	16·1 (14·2, 18·1)	18934 (16573, 21389)	16·6 (14·7, 18·6)
**São Tomé and Príncipe**	15 (13, 17)	20·5 (18·2, 23)	29 (26, 33)	21·7 (19·3, 24·4)
**Senegal**	668 (593, 751)	16·1 (14·3, 18·1)	1671 (1463, 1882)	18·3 (16·2, 20·6)
**Sierra Leone**	452 (398, 509)	19·4 (17·1, 21·9)	849 (739, 956)	18·6 (16·3, 20·9)
**Togo**	300 (264, 339)	16·7 (14·8, 18·8)	803 (694, 910)	16·8 (14·7, 18·9)
**Eastern sub-Saharan Africa**	36452 (33485, 39326)	37 (34, 39·9)	82950 (75946, 89624)	39·4 (36·2, 42·6)
**Burundi**	1017 (927, 1106)	35·4 (32·2, 38·6)	2217 (2010, 2415)	38·3 (34·9, 41·7)

**Comoros**	100 (91, 109)	39 (35·6, 42·5)	237 (216, 258)	43·7 (40·1, 47·6)
**Djibouti**	92 (84, 101)	38·5 (35·2, 41·9)	344 (311, 377)	43·5 (39·6, 47·4)
**Eritrea**	481 (434, 525)	34·9 (31·9, 37·9)	1274 (1162, 1381)	38·7 (35·5, 42)
**Ethiopia**	10904 (10128, 11651)	41·4 (38·6, 44·1)	23113 (21469, 24712)	43·1 (40·2, 46)
**Kenya**	2620 (2326, 2938)	24·4 (21·5, 27·3)	7431 (6546, 8275)	26·6 (23·5, 29·6)
**Madagascar**	2513 (2294, 2732)	38·5 (35·1, 42)	6125 (5575, 6663)	42·1 (38·4, 45·8)
**Malawi**	2011 (1833, 2182)	39·2 (35·7, 42·8)	3941 (3601, 4263)	42·1 (38·3, 45·9)
**Mozambique**	2541 (2300, 2771)	31·8 (29, 34·6)	5671 (5168, 6188)	37·8 (34·5, 41·2)
**Rwanda**	2141 (1896, 2364)	56·8 (50·4, 62·8)	4141 (3694, 4549)	55·2 (49·2, 60·9)
**Somalia**	1370 (1238, 1494)	36·3 (33·1, 39·5)	3458 (3138, 3775)	39·4 (35·8, 42·9)
**South Sudan**	1268 (1147, 1389)	40 (36·4, 43·4)	2186 (1973, 2388)	41·9 (38·1, 45·5)
**Tanzania**	4994 (4540, 5425)	36·3 (33, 39·4)	12337 (11232, 13447)	40·5 (37, 44)
**Uganda**	2915 (2647, 3184)	34·5 (31·2, 37·6)	6997 (6371, 7641)	37·7 (34·4, 41·1)
**Zambia**	1464 (1330, 1591)	38·4 (35, 41·9)	3426 (3122, 3729)	38·5 (35·2, 41·8)
**Central sub-Saharan Africa**	9452 (8479, 10390)	30·6 (27·6, 33·7)	24569 (22010, 27135)	34·4 (31, 37·7)
**Angola**	1802 (1613, 1987)	32 (28·9, 35)	5349 (4810, 5922)	34·7 (31·3, 38·2)
**Central African Republic**	405 (363, 447)	25·6 (23·1, 28·2)	823 (734, 911)	27·9 (25·2, 30·7)
**Congo**	376 (337, 412)	26·9 (24·2, 29·6)	1045 (938, 1161)	30·3 (27·4, 33·5)
**DR Congo**	6602 (5926, 7257)	30·9 (27·9, 34·2)	16685 (14911, 18470)	35 (31·6, 38·7)
**Equatorial Guinea**	70 (63, 77)	28 (25·3, 30·8)	234 (209, 259)	33 (29·8, 36·3)
**Gabon**	199 (180, 219)	30·4 (27·5, 33·5)	432 (390, 475)	33·4 (30·1, 36·6)

Appendix Table 18: Number of prevalent cases and age-standardised prevalence rates of decompensated cirrhosis due to alcohol-related liver disease per 100 000 in 1990 and 2017 for both sexes by location

	1990		2017	
	**Counts (95% UI)**	**Rate (95% UI)**	**Counts (95% UI)**	**Rate (95% UI)**
**Global**	1126841 (1065993, 1190048)	25·3 (23·9, 26·7)	2458059 (2314716, 2610708)	30 (28·2, 31·8)
**High-income North America**	82517 (78419, 87080)	25 (23·8, 26·4)	153144 (144514, 162150)	29·2 (27·6, 30·8)
**Canada**	8302 (7681, 8914)	25·8 (24, 27·7)	17244 (15947, 18627)	30·9 (28·6, 33·3)
**Greenland**	9 (9, 10)	19·3 (17·9, 21)	18 (16, 19)	24·7 (22·8, 26·7)
**USA**	74204 (70579, 78267)	24·9 (23·7, 26·3)	135879 (128459, 143704)	29 (27·4, 30·5)
**Australasia**	2919 (2679, 3171)	12·9 (11·8, 14)	7066 (6459, 7663)	17·9 (16·3, 19·4)
**Australia**	2435 (2220, 2666)	12·9 (11·7, 14·1)	6043 (5500, 6583)	18·1 (16·5, 19·8)
**New Zealand**	484 (453, 518)	12·9 (12·1, 13·8)	1023 (955, 1091)	16·5 (15·4, 17·6)
**High-income Asia-Pacific**	93690 (86823, 100843)	44·5 (41·3, 47·9)	152228 (140629, 163935)	49·4 (45·5, 53·2)
**Brunei**	57 (52, 62)	36·7 (33·5, 40)	216 (194, 238)	51 (46·3, 55·9)
**Japan**	56004 (52685, 59918)	32·9 (31, 35·1)	79796 (74665, 85282)	35·5 (33·1, 37·9)
**Singapore**	577 (486, 684)	19·9 (16·8, 23·5)	2185 (1827, 2595)	30·1 (25·3, 35·6)
**South Korea**	37052 (32401, 41503)	97 (85, 108·3)	70031 (62621, 77342)	84·4 (75·7, 93·1)
**Western Europe**	272481 (258161, 287355)	55·1 (52·3, 58·1)	456368 (429786, 482753)	70·6 (66·6, 74·6)
**Andorra**	37 (35, 39)	59·4 (55·7, 63)	101 (95, 107)	80·8 (75·7, 85·5)
**Austria**	9606 (9077, 10123)	97·3 (92, 102·6)	13735 (12946, 14517)	103·1 (97·4, 108·6)
**Belgium**	6813 (6163, 7459)	52·2 (47, 57·2)	13156 (11802, 14452)	79·1 (70·7, 86·9)
**Cyprus**	274 (259, 289)	33·4 (31·5, 35·2)	844 (793, 894)	49·6 (46·4, 52·4)
**Denmark**	3586 (3418, 3769)	54·9 (52·2, 57·7)	7349 (6952, 7771)	88·2 (83·3, 93·1)
**Finland**	2851 (2697, 3002)	44·7 (42·5, 47·1)	6575 (6167, 6953)	80·4 (75·7, 85)
**France**	49647 (46992, 52329)	70·2 (66·4, 74·1)	72919 (68803, 77216)	77·5 (73·3, 82·1)

**Germany**	74253 (70809, 78177)	68·8 (65·6, 72·4)	121744 (114772, 128500)	93·2 (88·2, 98·4)
**Greece**	6171 (5834, 6511)	45·2 (42·8, 47·7)	11005 (10374, 11623)	68·4 (64·5, 72·3)
**Iceland**	77 (68, 86)	29·4 (25·8, 33)	276 (240, 310)	62·5 (54·5, 70·4)
**Ireland**	1313 (1238, 1395)	35·6 (33·5, 37·8)	4642 (4359, 4935)	72·8 (68·3, 77·4)
**Israel**	1507 (1420, 1600)	32·4 (30·6, 34·4)	5356 (5021, 5710)	55·2 (51·7, 58·7)
**Italy**	30306 (27136, 33656)	38·7 (34·7, 42·9)	39332 (35581, 43251)	39·9 (36·1, 43·9)
**Luxembourg**	381 (360, 402)	75·8 (71·7, 79·9)	939 (879, 1001)	112 (105, 119·4)
**Malta**	129 (122, 136)	29·9 (28·2, 31·6)	313 (294, 333)	47·6 (44·7, 50·6)
**Netherlands**	6975 (6628, 7326)	38·4 (36·5, 40·4)	16878 (15902, 17847)	66·7 (63, 70·5)
**Norway**	2277 (2180, 2376)	44·3 (42·3, 46·2)	4301 (4090, 4504)	59 (56·1, 61·8)
**Portugal**	7441 (6929, 7974)	58·7 (54·6, 62·7)	9990 (9297, 10691)	59·8 (55·8, 64·1)
**Spain**	33688 (30503, 36539)	68·1 (61·9, 73·8)	56601 (51129, 61670)	79 (71·4, 86·3)
**Sweden**	4337 (4084, 4586)	38·4 (36·1, 40·7)	8556 (7975, 9097)	60 (56, 63·9)
**Switzerland**	4231 (4028, 4444)	47·1 (44·8, 49·4)	7938 (7477, 8404)	61·4 (57·8, 64·9)
**United Kingdom**	26318 (25152, 27610)	37·2 (35·5, 39·1)	53347 (50786, 56049)	58·3 (55·4, 61·2)
**Eastern Europe**	103452 (97773, 109203)	37·5 (35·5, 39·5)	220955 (208345, 233576)	74·4 (70·2, 78·5)
**Belarus**	4994 (4585, 5399)	40·2 (37, 43·5)	10436 (9569, 11291)	76·4 (70·3, 82·7)
**Estonia**	818 (751, 889)	42·7 (39·2, 46·5)	1568 (1452, 1689)	83·2 (77, 89·7)
**Latvia**	1311 (1206, 1415)	38·9 (35·9, 42·1)	1990 (1831, 2143)	69·9 (64·1, 75·3)
**Lithuania**	1969 (1815, 2118)	45·1 (41·5, 48·4)	3583 (3289, 3864)	86·6 (79·5, 93·8)
**Moldova**	5133 (4677, 5600)	106·4 (96·9, 115·7)	4674 (4257, 5093)	88·1 (80·6, 95·9)
**Russia**	61553 (58199, 64943)	34 (32·2, 35·8)	146611 (138100, 154665)	71·4 (67·3, 75·2)
**Ukraine**	27675 (26102, 29234)	41·1 (38·9, 43·5)	52094 (49036, 55323)	81·8 (76·8, 86·8)
**Central Europe**	119057 (112898, 125159)	80 (75·9, 84)	191787 (180842, 202749)	108·3 (102·3, 114·5)
**Albania**	1290 (1199, 1388)	51·5 (47·9, 55·4)	3405 (3160, 3639)	89·1 (83, 95·2)

**Bosnia and Herzegovina**	2880 (2671, 3075)	60 (55·9, 64)	4451 (4106, 4791)	83·9 (77·8, 90)
**Bulgaria**	8171 (7679, 8651)	67·2 (63·3, 71·1)	11671 (10901, 12407)	101·1 (94·7, 107·5)
**Croatia**	6182 (5797, 6533)	95·3 (89·5, 100·7)	6498 (6093, 6902)	94·2 (88·7, 99·9)
**Czech Republic**	9190 (8701, 9714)	71·1 (67·4, 75·1)	16701 (15744, 17622)	100·3 (94·9, 105·9)
**Hungary**	17458 (16483, 18403)	126·9 (119·8, 133·7)	15563 (14569, 16551)	100·2 (94·4, 106·4)
**Macedonia**	1158 (1080, 1240)	55·7 (51·9, 59·5)	2687 (2487, 2883)	86·1 (79·8, 92·2)
**Montenegro**	380 (355, 406)	57·2 (53·4, 61)	816 (763, 869)	91·5 (85·7, 97·4)
**Poland**	29087 (27423, 30697)	65·5 (61·7, 69)	68449 (64426, 72550)	118·3 (111·5, 125·8)
**Romania**	27560 (26008, 29087)	96·3 (91, 101·5)	36671 (34319, 38835)	118·8 (111·6, 125·4)
**Serbia**	5676 (5262, 6089)	47·5 (44·2, 50·8)	9211 (8568, 9846)	69·7 (65, 74·6)
**Slovakia**	7291 (6888, 7703)	123·1 (116·3, 129·9)	12349 (11574, 13047)	151·6 (142·8, 160·4)
**Slovenia**	2734 (2574, 2885)	110·4 (104·1, 116·4)	3317 (3105, 3533)	98·7 (92·6, 105)
**Central Asia**	22743 (21603, 23954)	42·2 (40·2, 44·5)	58795 (55619, 62148)	68 (64·5, 71·7)
**Armenia**	1131 (1070, 1197)	35·9 (34, 37·9)	2467 (2322, 2614)	62·6 (59·1, 66·2)
**Azerbaijan**	2421 (2281, 2566)	41·6 (39·2, 44)	6956 (6535, 7389)	63·9 (60·1, 67·5)
**Georgia**	2835 (2671, 2999)	44·5 (42·1, 47)	3417 (3216, 3630)	66·5 (62·6, 70·3)
**Kazakhstan**	5233 (4942, 5525)	35·5 (33·6, 37·5)	13329 (12565, 14153)	71·3 (67·5, 75·6)
**Kyrgyzstan**	1582 (1493, 1676)	47·3 (44·7, 50)	3582 (3369, 3807)	67 (63·3, 71·2)
**Mongolia**	537 (506, 570)	42·5 (40, 45·1)	1728 (1620, 1835)	59·6 (56·1, 63·2)
**Tajikistan**	1430 (1349, 1514)	43·7 (41·2, 46·2)	4302 (4026, 4585)	64·1 (60, 68)
**Turkmenistan**	1115 (1052, 1182)	47·5 (44·8, 50·2)	3360 (3166, 3565)	72·2 (67·9, 76·4)
**Uzbekistan**	6459 (6097, 6831)	48·1 (45·4, 50·8)	19655 (18457, 20841)	69·6 (65·7, 73·5)
**Central Latin America**	55721 (52826, 58707)	54·5 (51·7, 57·5)	193861 (182519, 205640)	78·5 (74·1, 83·2)
**Colombia**	7430 (6919, 7968)	34·1 (32, 36·5)	39220 (36388, 41975)	72·9 (67·7, 78)
**Costa Rica**	980 (918, 1047)	48·1 (45, 51·2)	4317 (4005, 4633)	84·8 (78·8, 91)

**El Salvador**	1384 (1287, 1488)	41·6 (38·7, 44·6)	4139 (3857, 4430)	72·8 (67·8, 78)
**Guatemala**	1985 (1843, 2132)	43·1 (40·1, 46·2)	7817 (7239, 8384)	65 (60·2, 69·8)
**Honduras**	1031 (956, 1107)	41·3 (38·4, 44·3)	4460 (4133, 4790)	66·3 (61·3, 71·3)
**Mexico**	36652 (34840, 38548)	69·9 (66·5, 73·4)	104569 (99221, 110201)	84·7 (80·5, 89·2)
**Nicaragua**	822 (765, 886)	42·7 (39·6, 45·8)	3679 (3401, 3956)	72·2 (66·8, 77·6)
**Panama**	630 (588, 672)	37 (34·6, 39·4)	2929 (2729, 3142)	72·8 (67·9, 78·1)
**Venezuela**	4806 (4507, 5096)	40·3 (37·8, 42·7)	22732 (21018, 24446)	73·7 (68·4, 79·3)
**Andean Latin America**	5408 (4987, 5855)	22·9 (21·1, 24·8)	24018 (22047, 25921)	43 (39·5, 46·5)
**Bolivia**	676 (619, 738)	18 (16·5, 19·6)	3459 (3161, 3761)	37 (33·8, 40·2)
**Ecuador**	1091 (1000, 1192)	17·7 (16·2, 19·4)	5646 (5153, 6165)	37 (33·7, 40·4)
**Peru**	3641 (3354, 3950)	26·6 (24·5, 28·8)	14913 (13697, 16090)	47·8 (43·8, 51·5)
**Caribbean**	5518 (5202, 5860)	19·8 (18·6, 21)	16221 (15199, 17218)	31·9 (29·9, 33·8)
**Antigua and Barbuda**	10 (9, 10)	19·3 (17·9, 20·7)	34 (31, 36)	31·4 (29·4, 33·5)
**The Bahamas**	44 (41, 47)	24 (22·5, 25·5)	134 (125, 142)	32 (30·1, 34)
**Barbados**	48 (46, 52)	18·7 (17·5, 20)	135 (126, 144)	30·9 (28·9, 32·9)
**Belize**	21 (20, 22)	20·4 (19·1, 21·7)	102 (95, 109)	33·3 (31·3, 35·5)
**Bermuda**	17 (15, 18)	24·9 (22·9, 26·9)	39 (36, 42)	36·2 (33·5, 39)
**Cuba**	1746 (1643, 1862)	16·5 (15·5, 17·6)	5688 (5285, 6076)	33·2 (30·8, 35·3)
**Dominica**	13 (12, 13)	19·5 (18·3, 20·7)	28 (26, 30)	32·8 (30·8, 34·9)
**Dominican Republic**	993 (931, 1061)	22·8 (21·3, 24·3)	3398 (3171, 3613)	34·8 (32·5, 37·1)
**Grenada**	14 (13, 15)	21·2 (19·9, 22·6)	42 (40, 45)	31·8 (29·8, 33·9)
**Guyana**	114 (107, 121)	24·2 (22·8, 25·7)	207 (194, 222)	30 (28·1, 31·9)
**Haiti**	594 (553, 636)	15·3 (14·3, 16·4)	2072 (1929, 2216)	25 (23·3, 26·7)
**Jamaica**	275 (259, 292)	15·7 (14·7, 16·7)	865 (807, 921)	29·6 (27·6, 31·5)
**Puerto Rico**	1156 (1086, 1229)	31·7 (29·8, 33·7)	2020 (1892, 2157)	35·6 (33·3, 38)

**Saint Lucia**	21 (20, 22)	22·9 (21·4, 24·4)	69 (65, 73)	32·4 (30·3, 34·4)
**Saint Vincent and the Grenadines**	14 (13, 14)	18·5 (17·4, 19·7)	40 (37, 44)	30 (27·8, 32·3)
**Suriname**	59 (55, 63)	20·6 (19·3, 22)	195 (183, 209)	31·5 (29·7, 33·7)
**Trinidad and Tobago**	170 (160, 182)	18·1 (17, 19·4)	512 (476, 546)	28·6 (26·7, 30·5)
**Virgin Islands**	22 (21, 24)	22 (20·7, 23·5)	57 (53, 60)	34·8 (32·5, 37)
**Tropical Latin America**	17109 (16225, 18063)	15·4 (14·6, 16·2)	42366 (39991, 44976)	17·4 (16·4, 18·4)
**Brazil**	16679 (15826, 17610)	15·4 (14·6, 16·2)	40974 (38671, 43525)	17·2 (16·2, 18·2)
**Paraguay**	430 (393, 470)	16·6 (15·2, 18·1)	1393 (1276, 1518)	23·6 (21·6, 25·7)
**Southern Latin America**	18152 (17221, 19113)	38 (36, 40)	45131 (42664, 47637)	58·7 (55·6, 62)
**Argentina**	11427 (10818, 12040)	34·8 (32·9, 36·6)	28815 (27207, 30517)	57·9 (54·6, 61·4)
**Chile**	5804 (5482, 6137)	51·9 (49, 55)	14190 (13301, 15043)	62·6 (58·8, 66·2)
**Uruguay**	920 (867, 973)	25·4 (24, 26·9)	2124 (1993, 2258)	48·1 (45, 51)
**East Asia**	169813 (158576, 181318)	15·8 (14·8, 16·9)	492776 (457805, 528978)	23·5 (21·9, 25·1)
**China**	162779 (152005, 173766)	16 (15, 17·1)	472369 (438846, 506879)	23·7 (22·1, 25·3)
**North Korea**	1781 (1598, 2004)	9·1 (8·3, 10·2)	5455 (4861, 6109)	16·7 (15, 18·5)
**Taiwan (Province of China)**	2424 (2157, 2688)	13·3 (11·8, 14·8)	7012 (6236, 7840)	19·5 (17·3, 21·6)
**Southeast Asia**	13858 (12574, 15271)	4·4 (4, 4·8)	49256 (44140, 54832)	7·3 (6·6, 8·1)
**Cambodia**	229 (209, 251)	4·1 (3·7, 4·4)	1025 (931, 1134)	7·7 (7, 8·5)
**Indonesia**	5534 (5106, 5966)	4·4 (4·1, 4·7)	11432 (10527, 12381)	4·5 (4·2, 4·9)
**Laos**	120 (109, 132)	4·8 (4·3, 5·2)	429 (391, 471)	8·2 (7·5, 8·9)
**Malaysia**	446 (358, 547)	3·8 (3, 4·6)	2308 (1861, 2798)	7·9 (6·4, 9·5)
**Maldives**	5 (5, 6)	4·4 (4, 4·8)	36 (33, 40)	8·8 (7·9, 9·6)
**Mauritius**	53 (48, 58)	5·9 (5·4, 6·5)	143 (129, 157)	8·4 (7·6, 9·2)
**Myanmar**	1088 (984, 1208)	3·9 (3·5, 4·3)	3372 (3049, 3734)	6·5 (5·9, 7·2)
**Philippines**	1008 (916, 1108)	2·5 (2·3, 2·8)	5744 (5174, 6322)	6·6 (6, 7·2)

**Sri Lanka**	604 (549, 668)	4·5 (4·1, 5)	2115 (1917, 2330)	8·3 (7·6, 9·1)
**Seychelles**	3 (2, 3)	4·5 (4·1, 5)	10 (9, 12)	8·7 (7·9, 9·7)
**Thailand**	2192 (1745, 2715)	4·9 (3·9, 6·1)	8928 (7000, 11066)	8·9 (7, 11)
**East Timor**	17 (15, 19)	4 (3·6, 4·4)	65 (59, 71)	7·4 (6·7, 8·1)
**Vietnam**	2542 (2311, 2802)	5·7 (5·2, 6·3)	13584 (12256, 14961)	13·1 (11·9, 14·3)
**Oceania**	207 (192, 223)	5 (4·7, 5·4)	752 (692, 820)	8·1 (7·5, 8·8)
**American Samoa**	2 (2, 2)	5·8 (5·3, 6·4)	5 (4, 5)	9·2 (8·4, 10·1)
**Federated States of Micronesia**	3 (3, 4)	5·7 (5·2, 6·1)	8 (7, 9)	8·8 (8·1, 9·6)
**Fiji**	24 (22, 26)	4·4 (4·1, 4·8)	75 (69, 82)	8·4 (7·7, 9·2)
**Guam**	8 (8, 9)	7·6 (7, 8·2)	21 (19, 23)	11·4 (10·5, 12·4)
**Kiribati**	2 (2, 3)	4·9 (4·6, 5·3)	7 (7, 8)	7·9 (7·3, 8·6)
**Marshall Islands**	1 (1, 1)	5·7 (5·2, 6·1)	4 (4, 5)	9·1 (8·3, 9·8)
**Northern Mariana Islands**	3 (2, 3)	7·7 (7·1, 8·4)	7 (6, 8)	11·1 (10·2, 12·1)
**Papua New Guinea**	124 (115, 135)	4·9 (4·5, 5·3)	502 (461, 551)	7·8 (7·2, 8·5)
**Samoa**	6 (6, 7)	6·3 (5·8, 6·9)	17 (15, 18)	10·4 (9·6, 11·4)
**Solomon Islands**	10 (9, 11)	5·2 (4·8, 5·7)	38 (35, 41)	8·6 (7·9, 9·3)
**Tonga**	4 (4, 4)	6 (5·6, 6·5)	9 (8, 10)	10·3 (9·5, 11·3)
**Vanuatu**	5 (5, 5)	5·6 (5·2, 6·1)	17 (16, 19)	8·3 (7·6, 9)
**North Africa and Middle East**	10543 (9583, 11534)	5·2 (4·7, 5·7)	30315 (27543, 33099)	6·2 (5·6, 6·8)
**Afghanistan**	394 (357, 433)	5·3 (4·8, 5·8)	829 (747, 913)	6 (5·4, 6·5)
**Algeria**	859 (787, 938)	6 (5·5, 6·6)	2717 (2476, 2974)	7·5 (6·9, 8·2)
**Bahrain**	21 (19, 23)	7·4 (6·7, 8)	125 (113, 138)	8·4 (7·7, 9·2)
**Egypt**	1982 (1708, 2271)	5·9 (5·1, 6·8)	4111 (3541, 4725)	5·8 (5, 6·7)
**Iran**	1082 (998, 1169)	3·3 (3, 3·5)	3335 (3084, 3583)	4·1 (3·8, 4·4)
**Iraq**	499 (458, 543)	5·5 (5, 6)	1674 (1525, 1832)	6 (5·5, 6·5)

**Jordan**	108 (98, 118)	6 (5·5, 6·6)	576 (520, 633)	7·7 (7, 8·5)
**Kuwait**	59 (54, 65)	5·7 (5·2, 6·2)	276 (250, 304)	7·8 (7·1, 8·5)
**Lebanon**	157 (144, 172)	6·2 (5·7, 6·7)	519 (474, 565)	7·9 (7·2, 8·6)
**Libya**	136 (124, 148)	6·1 (5·5, 6·6)	416 (379, 457)	7·5 (6·9, 8·2)
**Morocco**	870 (797, 950)	5·4 (4·9, 5·9)	2296 (2078, 2507)	6·7 (6·1, 7·3)
**Palestine**	57 (52, 61)	5·7 (5·2, 6·2)	209 (190, 228)	7 (6·4, 7·6)
**Oman**	62 (57, 68)	6·5 (5·9, 7·1)	257 (233, 283)	8·2 (7·5, 8·9)
**Qatar**	19 (17, 21)	7·5 (6·8, 8·2)	191 (172, 212)	9·4 (8·5, 10·3)
**Saudi Arabia**	488 (429, 558)	6·1 (5·3, 7)	1829 (1582, 2085)	7·2 (6·2, 8·2)
**Sudan**	686 (627, 751)	6·4 (5·8, 6·9)	1668 (1513, 1826)	7·7 (7, 8·5)
**Syria**	346 (316, 380)	5·5 (5, 6)	1046 (950, 1153)	7·1 (6·5, 7·8)
**Tunisia**	532 (450, 620)	9·2 (7·8, 10·7)	1458 (1246, 1686)	11·4 (9·8, 13·2)
**Turkey**	1751 (1543, 1979)	4·3 (3·8, 4·9)	4925 (4356, 5535)	5·5 (4·9, 6·2)
**United Arab Emirates**	79 (70, 90)	8·1 (7·3, 9)	794 (707, 894)	9 (8·2, 9·9)
**Yemen**	348 (319, 380)	5·7 (5·2, 6·2)	1035 (937, 1132)	6·6 (5·9, 7·2)
**South Asia**	83541 (78284, 89395)	10·7 (10·1, 11·5)	210476 (197272, 224812)	13·4 (12·5, 14·3)
**Bangladesh**	6560 (6107, 7057)	10·3 (9·6, 11)	16885 (15713, 18125)	12 (11·2, 12·9)
**Bhutan**	41 (39, 44)	12·2 (11·4, 13)	113 (105, 122)	14 (13, 15)
**India**	72516 (68134, 77393)	11·5 (10·8, 12·2)	182734 (171481, 195081)	14·5 (13·6, 15·5)
**Nepal**	2197 (1989, 2414)	17·4 (15·8, 19·1)	5189 (4745, 5647)	20·9 (19·1, 22·7)
**Pakistan**	2227 (1845, 2681)	3·3 (2·7, 4)	5554 (4578, 6713)	3·7 (3·1, 4·5)
**Southern sub-Saharan Africa**	5092 (4796, 5407)	15·1 (14·2, 16)	8751 (8208, 9301)	13·4 (12·5, 14·2)
**Botswana**	88 (82, 96)	12·1 (11·2, 13·1)	257 (236, 278)	14·3 (13·2, 15·5)
**Lesotho**	139 (128, 151)	12·1 (11·1, 13·1)	183 (168, 199)	12·7 (11·7, 13·8)
**Namibia**	83 (75, 92)	9·8 (8·9, 10·8)	234 (215, 254)	13·8 (12·6, 14·8)

**South Africa**	4005 (3786, 4244)	15·8 (14·9, 16·7)	6766 (6379, 7185)	13·3 (12·5, 14·1)
**Swaziland**	48 (44, 52)	12·2 (11·2, 13·2)	90 (82, 97)	12·3 (11·3, 13·2)
**Zimbabwe**	728 (670 , 790)	13·9 (12·8, 15·1)	1221 (1126, 1326)	13·7 (12·6, 14·8)
**Western sub-Saharan Africa**	22994 (21285, 24794)	21·1 (19·5, 22·8)	52634 (48678, 56863)	22·1 (20·3, 23·8)
**Benin**	453 (417, 492)	18·6 (17·1, 20·2)	1308 (1196, 1428)	21·2 (19·4, 23·2)
**Burkina Faso**	1118 (1035, 1202)	21·4 (19·9, 23)	2719 (2496, 2951)	23·9 (21·9, 25·8)
**Cameroon**	1036 (954, 1131)	18·2 (16·8, 19·9)	3159 (2904, 3425)	20·6 (18·9, 22·3)
**Cape Verde**	41 (38, 45)	18·8 (17·4, 20·5)	112 (103, 121)	23·7 (21·8, 25·7)
**Chad**	590 (547, 635)	18 (16·7, 19·4)	1367 (1255, 1485)	19·5 (17·8, 21·2)
**Côte d'Ivoire**	1042 (960, 1128)	17·5 (16·2, 19)	2836 (2611, 3095)	19·7 (18, 21·4)
**The Gambia**	91 (83, 98)	18·8 (17·3, 20·3)	254 (234, 276)	21·4 (19·7, 23·3)
**Ghana**	1429 (1315, 1549)	17·1 (15·8, 18·5)	4075 (3753, 4401)	20·3 (18·7, 21·9)
**Guinea**	651 (598, 711)	17·1 (15·7, 18·7)	1204 (1101, 1316)	18·4 (16·8, 20·1)
**Guinea-Bissau**	90 (83, 97)	17·5 (16·2, 18·9)	174 (159, 189)	17·7 (16·2, 19·3)
**Liberia**	281 (259, 303)	22·2 (20·5, 24)	594 (546, 647)	21·9 (20·1, 23·7)
**Mali**	887 (818, 959)	17·9 (16·5, 19·3)	2097 (1920, 2283)	20·2 (18·5, 22)
**Mauritania**	184 (170, 199)	15·3 (14·1, 16·5)	425 (391, 462)	17·6 (16·2, 19·1)
**Niger**	728 (667, 797)	18·8 (17·2, 20·6)	1977 (1805, 2167)	20·3 (18·6, 22·2)
**Nigeria**	12957 (11963, 14045)	24 (22·1, 26)	26919 (24799, 29209)	24·1 (22·2, 26·1)
**São Tomé and Príncipe**	16 (14, 17)	22 (20·3, 23·6)	34 (31, 37)	26 (23·8, 28·2)
**Senegal**	583 (539, 631)	14·6 (13·4, 15·8)	1521 (1398, 1654)	17·1 (15·7, 18·6)
**Sierra Leone**	523 (486, 566)	23·1 (21·4, 24·9)	1047 (964, 1139)	23·6 (21·6, 25·5)
**Togo**	294 (272, 318)	17·1 (15·8, 18·5)	810 (742, 885)	17·2 (15·7, 18·7)
**Eastern sub-Saharan Africa**	17566 (16289, 18974)	18·6 (17·3, 20)	39434 (36429, 42583)	19·5 (18, 21)
**Burundi**	582 (531, 637)	21·1 (19·3, 23·1)	1233 (1128, 1349)	22·2 (20·3, 24·3)

**Comoros**	41 (38, 44)	16·6 (15·2, 18)	96 (88, 105)	18·1 (16·6, 19·6)
**Djibouti**	43 (40, 48)	19·2 (17·6, 20·8)	166 (151, 182)	21·4 (19·6, 23·4)
**Eritrea**	211 (192, 230)	16 (14·7, 17·4)	553 (504, 608)	17·4 (15·9, 18·9)
**Ethiopia**	3811 (3531, 4100)	14·9 (13·9, 16)	8073 (7464, 8701)	15·7 (14·5, 16·8)
**Kenya**	2296 (2131, 2477)	21·8 (20·2, 23·5)	6051 (5605, 6518)	21·9 (20·4, 23·6)
**Madagascar**	1166 (1071, 1263)	18·6 (17·1, 20·1)	2731 (2497, 2976)	19·4 (17·9, 21·1)
**Malawi**	874 (801, 944)	17·8 (16·3, 19·2)	1671 (1545, 1815)	18·7 (17·2, 20·3)
**Mozambique**	1278 (1163, 1402)	16·6 (15·1, 18·3)	2867 (2631, 3126)	20·1 (18·3, 21·9)
**Rwanda**	901 (806, 1006)	24·7 (22·1, 27·6)	1659 (1485, 1853)	22·4 (20·1, 25)
**Somalia**	618 (559, 682)	17 (15·6, 18·7)	1521 (1385, 1672)	18·1 (16·5, 19·8)
**South Sudan**	611 (560, 668)	20·2 (18·5, 22·1)	1026 (929, 1129)	20·6 (18·8, 22·6)
**Tanzania**	2661 (2451, 2889)	20·1 (18·5, 21·9)	6254 (5769, 6798)	21·3 (19·6, 23·1)
**Uganda**	1774 (1646, 1915)	22·1 (20·5, 23·9)	3966 (3658, 4298)	22·6 (20·9, 24·5)
**Zambia**	691 (637, 749)	19·1 (17·6, 20·7)	1541 (1413, 1672)	18·2 (16·7, 19·7)
**Central sub-Saharan Africa**	4462 (4093, 4858)	15 (13·8, 16·3)	11726 (10751, 12746)	16·9 (15·5, 18·3)
**Angola**	835 (763, 915)	15·4 (14·1, 16·8)	2664 (2432, 2902)	17·9 (16·4, 19·3)
**Central African Republic**	202 (185, 222)	13·2 (12·1, 14·4)	393 (357, 430)	13·6 (12·4, 14·9)
**Congo**	191 (176, 207)	14·1 (13·1, 15·4)	523 (477, 572)	15·4 (14·1, 16·8)
**DR Congo**	3089 (2826, 3369)	15 (13·8, 16·4)	7801 (7128, 8524)	16·8 (15·4, 18·3)
**Equatorial Guinea**	34 (31, 38)	14·1 (12·9, 15·4)	124 (114, 136)	18·5 (17, 20·1)
**Gabon**	110 (101, 120)	17·2 (15·7, 18·8)	221 (202, 240)	17·3 (15·9, 18·8)

Appendix Table 19: Number of prevalent cases and age-standardised prevalence rates of decompensated cirrhosis due to non-alcoholic steatohepatitis (NASH) per 100 000 in 1990 and 2017 for both sexes by location

	1990		2017	
	Counts (95% UI)	Rate (95% UI)	Counts (95% UI)	Rate (95% UI)
**Global**	324736 (301647, 349163)	7·3 (6·8, 7·8)	917070 (850423, 986107)	11·3 (10·4, 12·1)
**High-income North America**	27379 (25826, 29034)	8·3 (7·8, 8·8)	58036 (54368, 61702)	11 (10·3, 11·7)
**Canada**	2293 (2071, 2530)	7·1 (6·4, 7·9)	5209 (4657, 5798)	9·3 (8·3, 10·3)
**Greenland**	3 (2, 3)	5·5 (4·9, 6)	5 (4, 6)	6·9 (6·1, 7·8)
**USA**	25083 (23722, 26596)	8·4 (7·9, 8·9)	52821 (49482, 56049)	11·2 (10·5, 11·9)
**Australasia**	1257 (1143, 1391)	5·6 (5, 6·2)	3663 (3338, 4025)	9·3 (8·4, 10·2)
**Australia**	1036 (931, 1158)	5·5 (4·9, 6·1)	3099 (2806, 3441)	9·3 (8·4, 10·4)
**New Zealand**	221 (208, 235)	5·9 (5·5, 6·3)	564 (528, 602)	9·1 (8·5, 9·7)
**High-income Asia-Pacific**	19588 (17372, 22097)	9·4 (8·3, 10·5)	33512 (29493, 37871)	10·5 (9·1, 12)
**Brunei**	9 (8, 10)	5·7 (5·1, 6·3)	39 (34, 45)	9·7 (8·6, 10·9)
**Japan**	14458 (13123, 15922)	8·6 (7·8, 9·4)	21593 (19721, 23567)	9·3 (8·5, 10·2)
**Singapore**	61 (50, 76)	2·2 (1·8, 2·7)	288 (227, 364)	4 (3·2, 5·1)
**South Korea**	5059 (3924, 6386)	13·6 (10·5, 17·1)	11591 (8958, 14507)	14 (11, 17·3)
**Western Europe**	41878 (37120, 47634)	8·5 (7·5, 9·7)	93388 (83334, 105134)	14·4 (12·8, 16·1)
**Andorra**	5 (4, 6)	8·2 (7·2, 9·4)	19 (16, 21)	14·8 (13, 16·8)
**Austria**	1294 (1137, 1456)	13·1 (11·5, 14·7)	2409 (2135, 2728)	18 (15·9, 20·4)
**Belgium**	602 (462, 773)	4·6 (3·5, 6)	1480 (1114, 1883)	8·9 (6·7, 11·4)
**Cyprus**	47 (41, 53)	5·7 (5, 6·4)	187 (167, 211)	11 (9·8, 12·4)
**Denmark**	495 (434, 562)	7·5 (6·6, 8·6)	1264 (1116, 1424)	15·1 (13·3, 17·1)
**Finland**	530 (467, 596)	8·3 (7·3, 9·4)	1551 (1380, 1740)	18·8 (16·6, 21·2)
**France**	6040 (5271, 6845)	8·6 (7·4, 9·8)	12710 (11262, 14227)	13·4 (11·8, 15·1)

**Germany**	10523 (9273, 11793)	9·7 (8·5, 10·9)	23802 (21150, 26906)	18·1 (16, 20·4)
**Greece**	1034 (919, 1154)	7·6 (6·7, 8·5)	2481 (2209, 2769)	15·3 (13·6, 17·2)
**Iceland**	39 (33, 45)	14·7 (12·4, 17·2)	164 (138, 191)	37·2 (31·4, 43·4)
**Ireland**	233 (206, 262)	6·3 (5·5, 7·1)	955 (847, 1071)	15 (13·3, 16·9)
**Israel**	391 (349, 439)	8·4 (7·5, 9·4)	1703 (1524, 1913)	17·5 (15·5, 19·6)
**Italy**	7300 (5787, 10318)	9·4 (7·4, 13·4)	12246 (10109, 14469)	12·3 (10, 14·6)
**Luxembourg**	62 (55, 70)	12·4 (10·9, 13·9)	167 (147, 191)	20 (17·6, 22·7)
**Malta**	26 (23, 29)	6·1 (5·4, 6·8)	82 (73, 92)	12·4 (10·9, 13·9)
**Netherlands**	1232 (1096, 1378)	6·8 (6, 7·6)	3726 (3330, 4171)	14·6 (13, 16·5)
**Norway**	421 (393, 450)	8·1 (7·6, 8·8)	934 (869, 999)	12·8 (11·9, 13·7)
**Portugal**	1056 (922, 1212)	8·4 (7·3, 9·6)	2159 (1918, 2438)	12·8 (11·3, 14·6)
**Spain**	4345 (3747, 5014)	8·9 (7·6, 10·3)	9951 (8592, 11537)	14 (12·1, 16·3)
**Sweden**	795 (724, 873)	7·1 (6·4, 7·8)	1971 (1799, 2164)	14 (12·7, 15·4)
**Switzerland**	609 (542, 686)	6·8 (6, 7·7)	1470 (1295, 1670)	11·3 (10, 12·8)
**United Kingdom**	4761 (4401, 5129)	6·7 (6·2, 7·3)	11858 (10913, 12821)	12·9 (11·9, 14)
**Eastern Europe**	27219 (25697, 28734)	10 (9·5, 10·6)	62994 (59357, 66926)	21·5 (20·3, 22·9)
**Belarus**	1122 (1024, 1225)	9·2 (8·3, 10)	2593 (2367, 2841)	19·3 (17·5, 21·1)
**Estonia**	197 (180, 215)	10·4 (9·5, 11·4)	369 (338, 403)	19·7 (18, 21·6)
**Latvia**	325 (296, 357)	9·8 (8·9, 10·7)	529 (483, 579)	18·7 (16·9, 20·6)
**Lithuania**	463 (423, 504)	10·7 (9·8, 11·7)	883 (804, 969)	21·4 (19·4, 23·5)
**Moldova**	1271 (1148, 1404)	26·4 (23·9, 29·2)	1356 (1233, 1484)	25·8 (23·4, 28·2)
**Russia**	16771 (15869, 17697)	9·4 (8·9, 10)	42962 (40464, 45546)	21·3 (20, 22·6)
**Ukraine**	7070 (6664, 7481)	10·7 (10·1, 11·3)	14301 (13446, 15229)	22·8 (21·4, 24·3)
**Central Europe**	18009 (16659, 19492)	12·2 (11·3, 13·3)	33800 (31196, 36546)	19·4 (17·9, 21)
**Albania**	262 (237, 288)	10·3 (9·3, 11·3)	740 (668, 812)	19·6 (17·8, 21·6)

**Bosnia and Herzegovina**	549 (497, 604)	11·5 (10·4, 12·6)	1075 (982, 1175)	20·6 (18·9, 22·5)
**Bulgaria**	1283 (1179, 1403)	10·8 (9·9, 11·8)	1989 (1823, 2163)	17·6 (16·1, 19·2)
**Croatia**	950 (871, 1034)	14·8 (13·6, 16·1)	1195 (1098, 1303)	17·6 (16·1, 19·2)
**Czech Republic**	1338 (1234, 1455)	10·5 (9·6, 11·4)	2691 (2474, 2931)	16·5 (15·1, 18)
**Hungary**	2659 (2408, 2947)	19·5 (17·6, 21·6)	2756 (2527, 3004)	18·1 (16·6, 19·7)
**Macedonia**	228 (209, 249)	11 (10·1, 12)	647 (589, 709)	21 (19·2, 23·1)
**Montenegro**	70 (64, 77)	10·6 (9·8, 11·6)	157 (143, 171)	17·9 (16·4, 19·6)
**Poland**	4297 (3940, 4654)	9·8 (9, 10·6)	11496 (10573, 12449)	20·3 (18·6, 22)
**Romania**	3874 (3550, 4215)	13·7 (12·6, 14·8)	6530 (5961, 7140)	21·5 (19·8, 23·4)
**Serbia**	1032 (935, 1123)	8·7 (7·9, 9·5)	1811 (1657, 1972)	13·9 (12·7, 15·2)
**Slovakia**	1061 (971, 1158)	18·1 (16·5, 19·7)	2052 (1878, 2235)	25·7 (23·5, 28·1)
**Slovenia**	405 (374, 441)	16·5 (15·2, 17·9)	660 (604, 723)	19·9 (18·3, 21·7)
**Central Asia**	4732 (4379, 5115)	8·7 (8·1, 9·4)	13855 (12772, 15060)	16·2 (15, 17·5)
**Armenia**	245 (225, 266)	7·9 (7·3, 8·5)	626 (576, 679)	16·1 (14·8, 17·4)
**Azerbaijan**	519 (478, 562)	8·9 (8·3, 9·6)	1809 (1643, 1991)	17 (15·5, 18·5)
**Georgia**	585 (536, 641)	9·3 (8·5, 10·1)	826 (755, 902)	16·2 (14·8, 17·6)
**Kazakhstan**	1069 (982, 1163)	7·3 (6·7, 7·9)	2891 (2654, 3143)	15·7 (14·5, 17·1)
**Kyrgyzstan**	327 (302, 354)	9·7 (8·9, 10·5)	833 (760, 910)	15·7 (14·4, 17·2)
**Mongolia**	112 (103, 121)	8·6 (7·9, 9·3)	373 (341, 409)	13·1 (12·1, 14·3)
**Tajikistan**	277 (254, 301)	8·2 (7·6, 8·9)	936 (851, 1036)	13·9 (12·8, 15·3)
**Turkmenistan**	232 (213, 251)	9·7 (9, 10·5)	824 (757, 903)	18 (16·5, 19·5)
**Uzbekistan**	1365 (1256, 1477)	10 (9·2, 10·8)	4737 (4341, 5169)	17 (15·6, 18·4)
**Central Latin America**	17291 (15920, 18807)	17 (15·7, 18·4)	73489 (67250, 80088)	30 (27·5, 32·6)
**Colombia**	1844 (1640, 2075)	8·6 (7·6, 9·6)	12141 (10886, 13655)	22·5 (20·2, 25·3)
**Costa Rica**	262 (234, 291)	12·9 (11·6, 14·3)	1464 (1315, 1632)	28·9 (26, 32·1)

**El Salvador**	449 (403, 503)	13·5 (12·1, 15·1)	1689 (1520, 1876)	29·7 (26·7, 33)
**Guatemala**	661 (584, 744)	14·5 (12·9, 16·2)	3315 (2959, 3715)	28 (24·9, 31·3)
**Honduras**	362 (320, 409)	14·6 (12·9, 16·4)	1901 (1689, 2124)	28·7 (25·6, 31·9)
**Mexico**	11783 (10961, 12710)	22·6 (21·1, 24·3)	41162 (38227, 44253)	33·6 (31·3, 36)
**Nicaragua**	284 (255, 315)	14·8 (13·4, 16·4)	1682 (1509, 1872)	33·5 (30·1, 37·1)
**Panama**	176 (157, 197)	10·4 (9·3, 11·7)	920 (802, 1047)	22·9 (20, 26·1)
**Venezuela**	1471 (1321, 1632)	12·5 (11·2, 13·8)	9214 (8248, 10265)	30·4 (27·3, 33·7)
**Andean Latin America**	2409 (2215, 2628)	10·3 (9·5, 11·2)	14607 (13472, 15790)	26·3 (24·2, 28·5)
**Bolivia**	328 (300, 358)	8·9 (8·1, 9·7)	2216 (2036, 2408)	24 (22, 26)
**Ecuador**	731 (670, 801)	11·9 (10·9, 13)	4630 (4246, 5038)	30·5 (28, 33·2)
**Peru**	1350 (1238, 1482)	9·9 (9·1, 10·9)	7761 (7143, 8446)	24·9 (22·9, 27·1)
**Caribbean**	2833 (2624, 3055)	10·2 (9·5, 11)	9883 (9086, 10728)	19·4 (17·9, 21)
**Antigua and Barbuda**	6 (5, 6)	11·5 (10·6, 12·5)	23 (21, 25)	22·1 (20·4, 23·9)
**The Bahamas**	19 (18, 21)	10·9 (10, 11·8)	75 (69, 82)	18·4 (17, 19·9)
**Barbados**	30 (27, 32)	11·2 (10·2, 12·2)	96 (88, 104)	21·7 (20, 23·5)
**Belize**	11 (10, 12)	10·6 (9·9, 11·5)	64 (59, 70)	21·3 (19·7, 23·1)
**Bermuda**	8 (7, 9)	12·3 (11·1, 13·6)	27 (25, 30)	24·6 (22·4, 26·9)
**Cuba**	996 (923, 1071)	9·4 (8·7, 10·2)	3703 (3397, 4033)	21·3 (19·6, 23·1)
**Dominica**	6 (6, 7)	9·4 (8·7, 10·2)	17 (15, 18)	19·4 (17·9, 20·9)
**Dominican Republic**	448 (413, 488)	10·4 (9·6, 11·3)	1771 (1620, 1934)	18·4 (16·8, 20)
**Grenada**	6 (6, 7)	9·6 (8·8, 10·4)	25 (23, 27)	18·6 (17·1, 20·2)
**Guyana**	50 (46, 54)	10·9 (10, 11·8)	124 (114, 135)	18·2 (16·9, 19·8)
**Haiti**	245 (217, 275)	6·5 (5·8, 7·2)	973 (851, 1103)	12·2 (10·7, 13·7)
**Jamaica**	154 (143, 166)	8·7 (8, 9·4)	579 (533, 629)	19·8 (18·3, 21·5)
**Puerto Rico**	602 (556, 648)	16·5 (15·2, 17·8)	1443 (1326, 1562)	24·5 (22·5, 26·5)

**Saint Lucia**	10 (10, 11)	11·3 (10·5, 12·3)	45 (41, 49)	21·1 (19·4, 22·8)
**Saint Vincent and the Grenadines**	8 (7, 9)	10·5 (9·7, 11·5)	34 (30, 38)	25 (22·4, 27·9)
**Suriname**	29 (27, 32)	10·2 (9·4, 11·1)	116 (107, 127)	19·1 (17·6, 20·6)
**Trinidad and Tobago**	97 (89, 104)	10·4 (9·6, 11·3)	376 (344, 409)	21·1 (19·4, 22·9)
**Virgin Islands**	12 (11, 13)	12 (11, 13·1)	37 (34, 40)	22·2 (20·3, 24)
**Tropical Latin America**	13484 (12648, 14415)	12·5 (11·7, 13·2)	43885 (41229, 46777)	18·2 (17·1, 19·4)
**Brazil**	13219 (12400, 14119)	12·5 (11·8, 13·3)	42797 (40235, 45616)	18·2 (17·1, 19·3)
**Paraguay**	265 (238, 296)	10·4 (9·3, 11·6)	1088 (976, 1215)	18·8 (16·9, 20·9)
**Southern Latin America**	3684 (3404, 3978)	7·7 (7·1, 8·3)	12265 (11268, 13216)	15·9 (14·6, 17·2)
**Argentina**	2116 (1943, 2295)	6·4 (5·9, 7)	7290 (6667, 7891)	14·6 (13·4, 15·9)
**Chile**	1323 (1221, 1428)	11·9 (11, 12·8)	4230 (3893, 4589)	18·7 (17·2, 20·2)
**Uruguay**	246 (226, 266)	6·8 (6·2, 7·3)	745 (685, 808)	16·6 (15·3, 18)
**East Asia**	67661 (63310, 72383)	6·3 (5·9, 6·7)	216086 (201093, 231857)	10·6 (9·9, 11·3)
**China**	64999 (60858, 69504)	6·3 (5·9, 6·8)	207346 (192968, 222462)	10·7 (10, 11·4)
**North Korea**	608 (534, 691)	3·2 (2·8, 3·6)	2000 (1740, 2286)	6·3 (5·5, 7·1)
**Taiwan (Province of China)**	927 (822, 1042)	5·1 (4·5, 5·8)	3260 (2881, 3686)	9·3 (8·2, 10·4)
**Southeast Asia**	10680 (9793, 11631)	3·4 (3·1, 3·7)	40846 (36819, 45179)	6·3 (5·7, 6·9)
**Cambodia**	172 (155, 190)	3 (2·8, 3·4)	712 (630, 797)	5·5 (4·9, 6·1)
**Indonesia**	4795 (4453, 5161)	3·8 (3·6, 4·1)	11515 (10627, 12441)	4·7 (4·4, 5·1)
**Laos**	66 (59, 73)	2·6 (2·4, 2·9)	261 (233, 293)	5·1 (4·6, 5·7)
**Malaysia**	302 (262, 344)	2·5 (2·2, 2·9)	1988 (1735, 2262)	6·9 (6·1, 7·8)
**Maldives**	3 (3, 4)	2·9 (2·6, 3·2)	31 (27, 35)	7·9 (7·1, 8·9)
**Mauritius**	33 (29, 37)	3·7 (3·3, 4·1)	129 (114, 143)	7·7 (6·9, 8·6)
**Myanmar**	780 (699, 868)	2·8 (2·5, 3·1)	2851 (2552, 3183)	5·7 (5·1, 6·3)
**Philippines**	578 (517, 643)	1·4 (1·3, 1·6)	3820 (3424, 4256)	4·5 (4·1, 5)

**Sri Lanka**	418 (375, 466)	3·2 (2·9, 3·5)	1707 (1542, 1893)	6·8 (6·1, 7·5)
**Seychelles**	2 (2, 3)	3·9 (3·5, 4·4)	11 (10, 13)	9·9 (8·6, 11·3)
**Thailand**	1751 (1510, 1996)	4 (3·4, 4·5)	9211 (7969, 10506)	9·4 (8·2, 10·7)
**East Timor**	11 (10, 12)	2·6 (2·4, 2·9)	47 (42, 53)	5·3 (4·7, 5·9)
**Vietnam**	1754 (1582, 1942)	3·9 (3·5, 4·3)	8511 (7508, 9671)	8·5 (7·6, 9·6)
**Oceania**	128 (111, 148)	3·1 (2·7, 3·6)	509 (435, 594)	5·6 (4·9, 6·5)
**American Samoa**	2 (2, 3)	6·9 (5·8, 8·2)	7 (6, 9)	13·9 (11·4, 16·5)
**Federated States of Micronesia**	3 (2, 3)	4 (3·6, 4·5)	7 (7, 8)	8·3 (7·4, 9·2)
**Fiji**	17 (15, 19)	3·2 (2·8, 3·6)	63 (55, 71)	7·2 (6·4, 8·1)
**Guam**	6 (5, 6)	5·3 (4·7, 5·9)	19 (17, 21)	10·1 (9, 11·3)
**Kiribati**	2 (2, 2)	3·9 (3·5, 4·4)	7 (6, 8)	7·8 (7, 8·7)
**Marshall Islands**	1 (1, 1)	4·4 (3·9, 4·9)	4 (3, 4)	8·6 (7·7, 9·6)
**Northern Mariana Islands**	2 (2, 2)	5·9 (5·3, 6·7)	6 (5, 7)	9·8 (8·7, 10·9)
**Papua New Guinea**	68 (58, 80)	2·6 (2·2, 3)	298 (249, 356)	4·7 (4, 5·5)
**Samoa**	6 (5, 7)	5·6 (4·9, 6·4)	17 (15, 20)	10·9 (9·6, 12·3)
**Solomon Islands**	7 (6, 8)	3·4 (2·9, 3·8)	29 (25, 33)	6·6 (5·8, 7·4)
**Tonga**	4 (3, 4)	5·9 (5·2, 6·6)	11 (9, 12)	12·3 (10·7, 14)
**Vanuatu**	3 (3, 4)	3·6 (3·1, 4·1)	13 (11, 15)	6·2 (5·4, 7·2)
**North Africa and Middle East**	30363 (27577, 33146)	14·8 (13·5, 16·1)	109375 (99728, 119573)	22·8 (20·9, 24·9)
**Afghanistan**	972 (876, 1070)	12·9 (11·7, 14·1)	2253 (1941, 2584)	15·7 (13·8, 18)
**Algeria**	2191 (1984, 2411)	14·9 (13·5, 16·4)	8796 (8022, 9625)	24·8 (22·6, 27)
**Bahrain**	53 (49, 58)	19·1 (17·6, 20·6)	407 (370, 448)	30·2 (28, 32·7)
**Egypt**	6555 (5747, 7553)	19·4 (17, 22·2)	17897 (15669, 20478)	26 (22·8, 29·6)
**Iran**	3610 (3393, 3832)	10·4 (9·8, 11·1)	13906 (13022, 14779)	17·7 (16·6, 18·7)
**Iraq**	1640 (1523, 1771)	17·4 (16·2, 18·8)	5814 (5331, 6309)	20·7 (19, 22·4)

**Jordan**	352 (324, 381)	19·1 (17·7, 20·6)	2377 (2194, 2577)	32·5 (30·1, 35)
**Kuwait**	197 (177, 218)	19 (17·2, 20·9)	1259 (1121, 1401)	36·7 (33·2, 40·5)
**Lebanon**	415 (382, 449)	16·1 (14·9, 17·4)	1882 (1733, 2030)	28·7 (26·5, 31)
**Libya**	465 (427, 503)	20·3 (18·6, 21·9)	1829 (1673, 1994)	33·9 (31·4, 36·8)
**Morocco**	2293 (2071, 2515)	14 (12·6, 15·3)	7806 (7095, 8562)	23·2 (21·1, 25·3)
**Palestine**	181 (167, 194)	17·3 (16, 18·6)	735 (674, 801)	24·7 (22·7, 26·8)
**Oman**	150 (137, 165)	15·8 (14·5, 17·3)	914 (837, 996)	31·3 (28·9, 33·8)
**Qatar**	64 (54, 75)	27·7 (24·2, 31·6)	697 (611, 788)	37·7 (34, 41·5)
**Saudi Arabia**	1387 (1234, 1558)	17 (15, 19·2)	7278 (6342, 8315)	30·1 (26·5, 34)
**Sudan**	1613 (1461, 1782)	14·5 (13·1, 16)	4743 (4252, 5251)	21·5 (19·2, 23·8)
**Syria**	1071 (987, 1151)	16·3 (15·1, 17·6)	3833 (3531, 4155)	26·6 (24·6, 28·7)
**Tunisia**	1392 (1192, 1610)	23·8 (20·3, 27·5)	4729 (4047, 5466)	37·7 (32·5, 43·5)
**Turkey**	4802 (4271, 5357)	11·9 (10·6, 13·2)	17210 (15324, 19160)	19·4 (17·4, 21·6)
**United Arab Emirates**	167 (151, 184)	18·1 (16·7, 19·6)	2271 (2017, 2544)	29·7 (27·4, 32)
**Yemen**	771 (678, 873)	12·3 (10·8, 13·9)	2636 (2279, 3035)	16·6 (14·3, 19·2)
**South Asia**	15893 (14851, 17036)	2·1 (1·9, 2·2)	45518 (42330, 49120)	2·9 (2·7, 3·2)
**Bangladesh**	1543 (1393, 1707)	2·4 (2·2, 2·7)	4624 (4164, 5132)	3·4 (3·1, 3·7)
**Bhutan**	10 (9, 11)	2·9 (2·6, 3·2)	32 (29, 36)	4·1 (3·7, 4·6)
**India**	12385 (11637, 13236)	2 (1·9, 2·1)	35256 (32953, 37769)	2·9 (2·7, 3·1)
**Nepal**	241 (214, 270)	1·9 (1·7, 2·2)	692 (619, 777)	2·8 (2·5, 3·1)
**Pakistan**	1714 (1507, 1932)	2·5 (2·2, 2·8)	4914 (4323, 5559)	3·4 (3, 3·8)
**Southern sub-Saharan Africa**	2613 (2425, 2819)	7·8 (7·2, 8·4)	5548 (5096, 6046)	8·6 (8, 9·4)
**Botswana**	41 (37, 45)	5·5 (5, 6·2)	145 (130, 162)	8·3 (7·5, 9·3)
**Lesotho**	65 (59, 72)	5·7 (5·1, 6·3)	101 (90, 112)	7·1 (6·4, 7·9)
**Namibia**	37 (32, 42)	4·3 (3·8, 4·9)	100 (87, 114)	5·9 (5·2, 6·8)

**South Africa**	2116 (1974, 2270)	8·4 (7·8, 9)	4542 (4205, 4914)	9·1 (8·4, 9·8)
**Swaziland**	27 (24, 30)	6·8 (6·1, 7·5)	60 (54, 67)	8·4 (7·5, 9·2)
**Zimbabwe**	328 (292, 370)	6·2 (5·6, 6·9)	601 (526, 692)	6·8 (6, 7·8)
**Western sub-Saharan Africa**	8675 (7922, 9474)	8 (7·4, 8·7)	23301 (21222, 25498)	10 (9·1, 10·9)
**Benin**	201 (183, 219)	8·2 (7·5, 9)	707 (639, 782)	11·6 (10·5, 12·8)
**Burkina Faso**	422 (385, 465)	8·1 (7·3, 8·9)	1158 (1045, 1278)	10·3 (9·4, 11·3)
**Cameroon**	526 (470, 584)	9·4 (8·5, 10·4)	1736 (1567, 1928)	11·6 (10·4, 12·8)
**Cape Verde**	20 (18, 22)	8·9 (8·2, 9·7)	62 (56, 67)	13·3 (12, 14·5)
**Chad**	258 (234, 282)	7·8 (7·1, 8·6)	591 (525, 659)	8·4 (7·5, 9·4)
**Côte d'Ivoire**	426 (388, 469)	7·3 (6·7, 8)	1319 (1195, 1457)	9·5 (8·6, 10·4)
**The Gambia**	41 (37, 45)	8·5 (7·8, 9·4)	129 (118, 141)	11 (10·1, 12)
**Ghana**	587 (533, 645)	7·2 (6·5, 7·8)	2042 (1854, 2242)	10·5 (9·6, 11·5)
**Guinea**	301 (274, 331)	7·9 (7·2, 8·7)	588 (532, 652)	9 (8·1, 10)
**Guinea-Bissau**	41 (38, 45)	8 (7·3, 8·8)	96 (87, 106)	10 (9·1, 11)
**Liberia**	120 (109, 132)	9·5 (8·6, 10·4)	312 (278, 349)	11·8 (10·6, 13·1)
**Mali**	436 (396, 478)	8·9 (8·1, 9·7)	1181 (1066, 1306)	11·4 (10·3, 12·5)
**Mauritania**	111 (101, 123)	9·3 (8·4, 10·2)	320 (285, 358)	13·4 (12, 14·9)
**Niger**	318 (288, 351)	8·3 (7·5, 9·1)	951 (852, 1060)	9·8 (8·8, 10·9)
**Nigeria**	4194 (3818, 4601)	7·9 (7·2, 8·6)	10303 (9348, 11326)	9·4 (8·6, 10·4)
**São Tomé and Príncipe**	8 (7, 8)	10·6 (9·6, 11·6)	17 (15, 18)	13·1 (12, 14·3)
**Senegal**	323 (296, 354)	8·1 (7·4, 8·8)	925 (841, 1020)	10·5 (9·5, 11·5)
**Sierra Leone**	211 (193, 232)	9·3 (8·5, 10·2)	442 (400, 487)	10·1 (9·2, 11·1)
**Togo**	131 (119, 144)	7·7 (7, 8·5)	423 (381, 469)	9·3 (8·5, 10·3)
**Eastern sub-Saharan Africa**	7024 (6491, 7599)	7·6 (7, 8·2)	17295 (15976, 18707)	8·8 (8·1, 9·5)
**Burundi**	194 (177, 213)	7·3 (6·7, 8)	414 (373, 458)	7·7 (7, 8·6)

**Comoros**	20 (18, 22)	8·3 (7·5, 9·1)	51 (46, 56)	9·9 (9, 10·9)
**Djibouti**	17 (16, 19)	8·1 (7·4, 8·8)	86 (76, 97)	11·7 (10·5, 13)
**Eritrea**	85 (76, 94)	6·8 (6·2, 7·5)	236 (212, 262)	7·9 (7·1, 8·8)
**Ethiopia**	1600 (1492, 1712)	6·4 (6, 6·8)	3613 (3376, 3856)	7·1 (6·6, 7·6)
**Kenya**	984 (916, 1058)	9·5 (8·8, 10·2)	2888 (2676, 3128)	10·8 (10, 11·6)
**Madagascar**	461 (419, 508)	7·4 (6·8, 8·2)	1135 (1021, 1267)	8·4 (7·6, 9·3)
**Malawi**	409 (373, 448)	8·5 (7·7, 9·2)	861 (786, 938)	9·8 (8·9, 10·7)
**Mozambique**	530 (481, 580)	7·1 (6·4, 7·7)	1252 (1139, 1378)	9 (8·2, 9·9)
**Rwanda**	277 (245, 311)	7·8 (6·8, 8·7)	588 (524, 662)	8·3 (7·3, 9·3)
**Somalia**	263 (238, 291)	7·6 (6·9, 8·3)	693 (628, 765)	8·5 (7·8, 9·4)
**South Sudan**	280 (251, 312)	9·4 (8·5, 10·5)	508 (455, 568)	10·4 (9·4, 11·6)
**Tanzania**	1041 (948, 1140)	8 (7·3, 8·7)	2800 (2553, 3070)	9·8 (8·9, 10·7)
**Uganda**	564 (517, 618)	7·1 (6·5, 7·8)	1438 (1308, 1579)	8·4 (7·6, 9·2)
**Zambia**	296 (269, 323)	8·3 (7·6, 9·1)	722 (658, 792)	8·8 (8, 9·7)
**Central sub-Saharan Africa**	1936 (1759, 2122)	6·6 (6, 7·2)	5214 (4621, 5857)	7·7 (6·9, 8·6)
**Angola**	368 (333, 407)	6·9 (6·3, 7·6)	1358 (1227, 1503)	9·3 (8·5, 10·2)
**Central African Republic**	80 (72, 89)	5·4 (4·8, 5·9)	162 (142, 184)	5·8 (5·1, 6·6)
**Congo**	80 (73, 88)	6 (5·5, 6·6)	243 (219, 270)	7·4 (6·7, 8·2)
**DR Congo**	1347 (1223, 1482)	6·6 (6, 7·2)	3249 (2852, 3702)	7·2 (6·4, 8·1)
**Equatorial Guinea**	16 (14, 18)	6·6 (6, 7·3)	84 (71, 99)	12·9 (11, 15·3)
**Gabon**	45 (41, 49)	7·1 (6·4, 7·8)	119 (107, 133)	9·6 (8·6, 10·6)

Appendix Table 20: Number of prevalent cases and age-standardised prevalence rates of decompensated cirrhosis due to other causes per 100 000 in 1990 and 2017 for both sexes by location

	1990		2017	
	Counts (95% UI)	Rate (95% UI)	Counts (95% UI)	Rate (95% UI)
**Global**	1075930 (1023619, 1131884)	20 (18·9, 21·1)	1652969 (1550496, 1755854)	22·2 (20·9, 23·5)
**High-income North America**	82363 (76782, 88030)	26·1 (24·5, 27·7)	141068 (130611, 151041)	29·4 (27·6, 31·2)
**Canada**	6994 (6264, 7822)	23·3 (21·1, 25·7)	13353 (11723, 15163)	26·6 (24, 29·5)
**Greenland**	9 (8, 10)	18·7 (16·9, 20·6)	13 (12, 15)	22 (19·8, 24·3)
**USA**	75358 (70402, 80383)	26·4 (24·8, 27·9)	127699 (118660, 136505)	29·8 (28, 31·5)
**Australasia**	1452 (1333, 1594)	7·4 (6·8, 8)	3080 (2782, 3409)	10·7 (9·9, 11·5)
**Australia**	1144 (1037, 1273)	7 (6·4, 7·7)	2580 (2309, 2883)	10·6 (9·7, 11·5)
**New Zealand**	308 (290, 326)	9·3 (8·8, 9·9)	500 (468, 534)	11·2 (10·6, 11·8)
**High-income Asia-Pacific**	80414 (74244, 88664)	48·2 (45·2, 52·2)	95817 (87249, 104860)	48·7 (45·9, 51·8)
**Brunei**	90 (84, 97)	39 (35·8, 42·6)	181 (162, 202)	48·7 (44·1, 54)
**Japan**	57488 (54196, 61087)	48·1 (45·8, 50·4)	67679 (62386, 73166)	51 (48·6, 53·5)
**Singapore**	905 (815, 1012)	33·3 (30, 37)	2454 (2126, 2834)	43·8 (39·1, 49)
**South Korea**	21930 (18367, 28419)	56 (46·8, 72·9)	25503 (21763, 29977)	45·3 (40·9, 50·7)
**Western Europe**	107609 (97635, 118244)	26·2 (24·3, 28·2)	172621 (155471, 190528)	34·3 (31·8, 37·1)
**Andorra**	12 (11, 14)	23·7 (21·6, 25·9)	31 (27, 35)	34·5 (31·4, 38)
**Austria**	3104 (2783, 3474)	38·3 (35·1, 41·8)	4366 (3864, 4952)	44·1 (40·3, 48·3)
**Belgium**	2224 (1964, 2493)	21·6 (19·6, 23·7)	3832 (3297, 4421)	30·4 (27·2, 33·6)
**Cyprus**	117 (106, 129)	14·9 (13·6, 16·4)	306 (270, 343)	22·4 (20·3, 24·5)
**Denmark**	1191 (1070, 1324)	22·7 (20·9, 24·7)	2439 (2162, 2746)	37·5 (34·3, 40·9)
**Finland**	1253 (1135, 1387)	25·1 (23·2, 27·3)	2504 (2213, 2827)	37·7 (34·4, 41·2)
**France**	15567 (13876, 17347)	25·2 (22·8, 27·6)	25489 (22593, 28916)	33 (30·1, 36)

**Germany**	22694 (20187, 25525)	26·4 (24·1, 28·8)	39514 (34529, 45301)	39·8 (36·3, 43·7)
**Greece**	2479 (2239, 2785)	22·2 (20·5, 24·2)	3982 (3524, 4499)	32·4 (29·7, 35·3)
**Iceland**	62 (55, 68)	23·7 (21·5, 26·1)	178 (156, 200)	46·8 (42·1, 51·6)
**Ireland**	737 (683, 802)	19·9 (18·5, 21·6)	1698 (1520, 1881)	32·8 (29·9, 35·7)
**Israel**	1143 (1055, 1242)	22·7 (20·9, 24·7)	3267 (2962, 3577)	34·7 (31·6, 37·8)
**Italy**	21295 (18340, 25159)	35·1 (31·6, 39·9)	26153 (22864, 29755)	38·5 (35·3, 42·3)
**Luxembourg**	141 (127, 157)	35·6 (32·8, 38·9)	289 (256, 323)	46·6 (42·6, 50·8)
**Malta**	52 (47, 58)	13·6 (12·4, 15·1)	111 (97, 127)	21 (19, 23·1)
**Netherlands**	3087 (2793, 3413)	20·6 (19, 22·4)	6297 (5552, 7131)	31·8 (29·2, 34·7)
**Norway**	1240 (1174, 1311)	29·9 (28·5, 31·3)	1844 (1725, 1964)	33·2 (31·6, 35)
**Portugal**	2041 (1765, 2348)	17·4 (15·4, 19·7)	3345 (2918, 3788)	25·6 (23·2, 28·3)
**Spain**	5739 (5122, 6466)	14·8 (13·5, 16·3)	8267 (7188, 9421)	19·4 (17·7, 21·4)
**Sweden**	2112 (1955, 2263)	24·1 (22·7, 25·5)	3906 (3594, 4252)	34·4 (32·3, 36·5)
**Switzerland**	1446 (1298, 1625)	19·8 (18·1, 21·7)	2670 (2359, 3011)	26·3 (24, 28·9)
**United Kingdom**	19770 (18840, 20735)	31·9 (30·5, 33·3)	31953 (29972, 33907)	39·1 (37, 41·1)
**Eastern Europe**	60947 (57762, 64231)	29·3 (27·9, 30·8)	72874 (67800, 77870)	38·7 (36·6, 40·8)
**Belarus**	2781 (2568, 3009)	29·2 (27·2, 31·5)	3090 (2762, 3435)	37 (34·2, 40·1)
**Estonia**	419 (385, 457)	29·3 (27·2, 31·6)	435 (390, 483)	37·1 (34·3, 40·2)
**Latvia**	659 (606, 718)	26·8 (24·9, 28·9)	594 (533, 664)	34·4 (31·8, 37·3)
**Lithuania**	1109 (1025, 1203)	32·2 (29·9, 34·7)	1021 (914, 1147)	40 (36·9, 43·4)
**Moldova**	2012 (1801, 2255)	44·5 (40·2, 49·4)	1453 (1281, 1641)	41 (37·5, 44·7)
**Russia**	39639 (37610, 41660)	28·8 (27·4, 30·2)	50768 (47325, 54149)	38·5 (36·4, 40·5)
**Ukraine**	14329 (13541, 15126)	29·9 (28·4, 31·4)	15512 (14388, 16575)	39·9 (37·6, 42)
**Central Europe**	37851 (34979, 41032)	32·2 (30, 34·5)	38550 (34423, 43236)	37 (34·4, 40)
**Albania**	996 (932, 1071)	28·4 (26·4, 30·8)	961 (863, 1073)	35·4 (32·6, 38·4)

**Bosnia and Herzegovina**	1290 (1185, 1399)	30·3 (28·1, 32·7)	1150 (1022, 1301)	35·8 (33, 38·9)
**Bulgaria**	2324 (2129, 2541)	29 (27·1, 31·1)	2190 (1931, 2482)	34·1 (31·6, 36·7)
**Croatia**	1443 (1309, 1604)	31·3 (28·9, 33·8)	1273 (1123, 1451)	31 (28·7, 33·7)
**Czech Republic**	2529 (2325, 2754)	26·4 (24·5, 28·4)	3123 (2773, 3517)	32·4 (30·1, 35)
**Hungary**	3516 (3185, 3873)	35·2 (32·4, 38·1)	2955 (2624, 3347)	33·6 (31·2, 36·4)
**Macedonia**	551 (511, 593)	28·3 (26·3, 30·4)	677 (609, 757)	34·4 (31·7, 37·2)
**Montenegro**	169 (157, 183)	28·1 (26·2, 30·3)	200 (180, 223)	34·1 (31·5, 37)
**Poland**	12720 (11893, 13658)	34·7 (32·5, 37·3)	13969 (12511, 15541)	42·4 (39·4, 45·6)
**Romania**	7553 (6903, 8273)	32·7 (30·3, 35·4)	6955 (6155, 7927)	36·9 (34, 40·2)
**Serbia**	1673 (1525, 1843)	18·7 (17·3, 20·2)	2026 (1804, 2277)	23·8 (21·9, 25·8)
**Slovakia**	2481 (2295, 2668)	49·2 (45·6, 52·8)	2438 (2180, 2728)	52 (48·3, 56·1)
**Slovenia**	606 (547, 674)	31·9 (29·2, 34·8)	632 (556, 721)	31·9 (29·5, 34·7)
**Central Asia**	17932 (16774, 19149)	24 (22·4, 25·8)	29632 (27462, 31963)	34·2 (31·7, 36·9)
**Armenia**	738 (683, 798)	21·6 (20, 23·4)	920 (835, 1017)	32·8 (30·3, 35·5)
**Azerbaijan**	1678 (1557, 1808)	22·4 (20·7, 24·3)	2990 (2730, 3273)	32·6 (30, 35·2)
**Georgia**	1176 (1078, 1280)	21·8 (20·2, 23·6)	1118 (1012, 1240)	32·3 (29·8, 34·9)
**Kazakhstan**	3597 (3338, 3866)	21 (19·4, 22·6)	5670 (5222, 6141)	33·6 (31, 36·3)
**Kyrgyzstan**	1180 (1100, 1263)	24·7 (22·8, 26·7)	2192 (2029, 2372)	34·9 (32·1, 37·7)
**Mongolia**	582 (543, 622)	24 (22·2, 25·8)	909 (841, 985)	30·5 (28·2, 33·1)
**Tajikistan**	1608 (1503, 1718)	25·7 (23·9, 27·7)	3259 (3047, 3505)	34·5 (32, 37·2)
**Turkmenistan**	1089 (1015, 1170)	26·7 (24·6, 28·8)	1739 (1602, 1880)	36·5 (33·7, 39·4)
**Uzbekistan**	6285 (5862, 6750)	27 (25, 29·1)	10835 (10052, 11710)	35·1 (32·5, 38)
**Central Latin America**	30128 (28670, 31725)	19·8 (18·6, 21·1)	60251 (55600, 64966)	24·7 (22·8, 26·7)
**Colombia**	4541 (4265, 4839)	13·9 (12·9, 15)	10384 (9261, 11636)	20·5 (18·4, 22·8)
**Costa Rica**	476 (445, 509)	16 (14·7, 17·4)	1023 (900, 1158)	22·4 (19·9, 25·1)

**El Salvador**	811 (757, 866)	15·6 (14·2, 17·1)	1301 (1163, 1457)	22·2 (19·8, 25·1)
**Guatemala**	1340 (1256, 1433)	16·6 (15, 18·3)	3262 (2977, 3574)	22·2 (19·7, 24·9)
**Honduras**	829 (778, 882)	17·3 (15·8, 19)	1865 (1702, 2042)	22·2 (19·8, 24·8)
**Mexico**	18436 (17582, 19247)	24·5 (23, 25·8)	33993 (31735, 36241)	28·3 (26·4, 30·2)
**Nicaragua**	592 (557, 633)	15·3 (14, 16·8)	1259 (1143, 1388)	22·1 (19·6, 24·8)
**Panama**	344 (320, 370)	14·9 (13·7, 16·2)	843 (752, 943)	21·6 (19·3, 24·1)
**Venezuela**	2760 (2582, 2947)	14·9 (13·7, 16·2)	6320 (5642, 7046)	21·8 (19·5, 24·3)
**Andean Latin America**	4650 (4324, 4981)	13 (11·8, 14·3)	16303 (14934, 17871)	27·6 (25·1, 30·5)
**Bolivia**	717 (666, 768)	11·8 (10·6, 13)	2843 (2618, 3070)	26 (23·6, 28·6)
**Ecuador**	1243 (1155, 1334)	13·4 (12·1, 14·7)	4616 (4217, 5081)	29 (26·4, 32·1)
**Peru**	2689 (2500, 2892)	13·2 (11·9, 14·5)	8843 (8086, 9698)	27·3 (24·8, 30·2)
**Caribbean**	4343 (4007, 4707)	13 (11·9, 14·3)	9119 (8202, 10145)	19·6 (17·7, 21·6)
**Antigua and Barbuda**	8 (7, 9)	13·5 (12·3, 14·7)	18 (16, 20)	20·6 (18·8, 22·7)
**The Bahamas**	31 (29, 34)	13·7 (12·5, 15·1)	70 (63, 78)	20 (18·2, 22)
**Barbados**	34 (31, 37)	13 (12, 14·3)	68 (59, 77)	20·3 (18·5, 22·4)
**Belize**	24 (22, 25)	13·1 (12, 14·5)	71 (65, 76)	20 (18·1, 21·9)
**Bermuda**	8 (7, 8)	14 (12·9, 15·3)	16 (14, 19)	21·3 (19·4, 23·4)
**Cuba**	1329 (1218, 1458)	12·9 (11·9, 14·1)	2706 (2383, 3072)	20·9 (19, 23)
**Dominica**	10 (9, 11)	12·8 (11·8, 14·1)	15 (13, 16)	19·8 (18·1, 21·8)
**Dominican Republic**	891 (828, 958)	13·7 (12·5, 15·2)	1940 (1763, 2130)	19·9 (18, 21·9)
**Grenada**	12 (11, 13)	13·1 (12, 14·4)	23 (21, 26)	19·5 (17·7, 21·3)
**Guyana**	92 (86, 99)	13·2 (12·1, 14·5)	133 (121, 144)	19·1 (17·5, 21)
**Haiti**	624 (582, 673)	10·1 (9·2, 11·1)	1709 (1580, 1842)	16·3 (14·8, 18)
**Jamaica**	289 (269, 310)	12 (11·1, 13·1)	535 (485, 589)	19·8 (18, 21·7)
**Puerto Rico**	606 (546, 673)	16·6 (15, 18·5)	1020 (892, 1177)	22 (19·9, 24·4)

**Saint Lucia**	18 (17, 20)	13·9 (12·7, 15·2)	35 (31, 39)	20·1 (18·3, 22)
**Saint Vincent and the Grenadines**	14 (13, 15)	12·5 (11·5, 13·6)	22 (20, 25)	19·2 (17·6, 21·1)
**Suriname**	47 (43, 51)	13·1 (11·9, 14·5)	109 (99, 120)	19·5 (17·8, 21·5)
**Trinidad and Tobago**	144 (134, 156)	12·5 (11·5, 13·7)	276 (245, 309)	19·5 (17·7, 21·3)
**Virgin Islands**	14 (13, 15)	13·8 (12·6, 15·1)	26 (23, 30)	20·7 (18·9, 22·8)
**Tropical Latin America**	21700 (20666, 22665)	15·6 (14·7, 16·4)	36375 (34113, 38393)	17 (16·1, 17·9)
**Brazil**	21165 (20156, 22109)	15·6 (14·8, 16·4)	35243 (33066, 37206)	17 (16·1, 17·9)
**Paraguay**	535 (502, 571)	13·8 (12·7, 15·1)	1131 (1035, 1227)	17·7 (16·1, 19·4)
**Southern Latin America**	10113 (9176, 11135)	20·5 (18·5, 22·5)	25445 (22798, 28455)	37·1 (33·7, 40·8)
**Argentina**	6328 (5759, 6944)	18·8 (17·1, 20·6)	16462 (14803, 18305)	35·9 (32·7, 39·4)
**Chile**	3146 (2836, 3485)	26·2 (23·4, 29·3)	7561 (6710, 8544)	39·6 (35·9, 43·9)
**Uruguay**	639 (576, 711)	18·8 (17·1, 20·6)	1420 (1274, 1588)	36·9 (33·7, 40·4)
**East Asia**	202203 (192297, 213069)	16·3 (15·5, 17·1)	189830 (177729, 202093)	15·6 (14·8, 16·5)
**China**	195881 (186204, 206468)	16·6 (15·7, 17·4)	181656 (169962, 193239)	15·7 (15, 16·6)
**North Korea**	1725 (1599, 1863)	8·8 (8·2, 9·5)	2776 (2518, 3062)	13 (12, 14·1)
**Taiwan (Province of China)**	1229 (1122, 1346)	6·6 (6·1, 7·3)	2339 (2071, 2621)	11·6 (10·7, 12·6)
**Southeast Asia**	22903 (21521, 24447)	4·5 (4·2, 4·8)	39790 (36814, 42966)	6·5 (6, 7)
**Cambodia**	505 (471, 542)	4 (3·7, 4·3)	1010 (933, 1095)	6·3 (5·8, 6·9)
**Indonesia**	10170 (9568, 10764)	5·1 (4·8, 5·4)	12394 (11654, 13150)	5·2 (4·9, 5·5)
**Laos**	184 (172, 198)	3·7 (3·4, 4)	434 (402, 469)	6·2 (5·7, 6·7)
**Malaysia**	685 (629, 745)	3·5 (3·2, 3·8)	1756 (1602, 1934)	6·2 (5·7, 6·8)
**Maldives**	11 (10, 12)	3·9 (3·6, 4·2)	26 (24, 29)	6·9 (6·4, 7·6)
**Mauritius**	46 (42, 50)	4·3 (4, 4·6)	75 (67, 82)	6·8 (6·3, 7·4)
**Myanmar**	1632 (1509, 1768)	3·7 (3·4, 4·1)	3065 (2822, 3342)	6·2 (5·7, 6·7)
**Philippines**	1683 (1550, 1825)	2·2 (2·1, 2·4)	5227 (4786, 5701)	5 (4·6, 5·5)

**Sri Lanka**	697 (645, 758)	4 (3·7, 4·4)	1405 (1278, 1544)	6·8 (6·3, 7·5)
**Seychelles**	3 (3, 3)	4·1 (3·8, 4·5)	6 (6, 7)	7·2 (6·6, 7·8)
**Thailand**	2439 (2238, 2661)	4·5 (4·1, 4·9)	4692 (4104, 5275)	7·6 (6·9, 8·4)
**East Timor**	33 (30, 35)	3·6 (3·3, 3·9)	90 (83, 97)	6·3 (5·8, 6·8)
**Vietnam**	4786 (4397, 5196)	6·1 (5·7, 6·7)	9558 (8704, 10470)	11·3 (10·3, 12·3)
**Oceania**	484 (447, 522)	6 (5·6, 6·5)	1292 (1196, 1395)	9 (8·3, 9·7)
**American Samoa**	4 (4, 4)	6·8 (6·3, 7·4)	6 (5, 6)	10 (9·2, 10·8)
**Federated States of Micronesia**	10 (9, 10)	6·9 (6·4, 7·5)	11 (10, 12)	9·9 (9·1, 10·6)
**Fiji**	50 (47, 55)	5·7 (5·3, 6·2)	82 (76, 89)	9·1 (8·4, 9·8)
**Guam**	9 (9, 10)	6·8 (6·2, 7·4)	16 (14, 17)	9·7 (8·9, 10·5)
**Kiribati**	6 (6, 7)	6·8 (6·3, 7·3)	14 (13, 15)	10·1 (9·4, 11)
**Marshall Islands**	4 (4, 5)	6·9 (6·4, 7·4)	6 (6, 7)	10 (9·2, 10·9)
**Northern Mariana Islands**	3 (3, 3)	6·7 (6·2, 7·3)	4 (3, 4)	9·7 (8·9, 10·5)
**Papua New Guinea**	299 (276, 323)	5·8 (5·4, 6·2)	937 (866, 1013)	8·7 (8·1, 9·4)
**Samoa**	15 (14, 16)	7·2 (6·7, 7·8)	25 (23, 27)	10·5 (9·8, 11·4)
**Solomon Islands**	29 (27, 31)	6·2 (5·8, 6·7)	74 (68, 80)	9·6 (8·9, 10·4)
**Tonga**	9 (8, 10)	7·3 (6·8, 7·9)	12 (11, 13)	10·8 (9·9, 11·7)
**Vanuatu**	14 (13, 15)	7·2 (6·6, 7·7)	34 (32, 37)	9·9 (9·2, 10·8)
**North Africa and Middle East**	89906 (84376, 95996)	22·6 (21·1, 24·3)	149697 (139848, 160369)	25·7 (23·9, 27·5)
**Afghanistan**	3065 (2861, 3285)	24 (22·4, 25·9)	10720 (9984, 11555)	26 (24·1, 28)
**Algeria**	7039 (6559, 7535)	23·6 (21·8, 25·6)	10156 (9434, 10928)	27·3 (25·3, 29·5)
**Bahrain**	112 (104, 120)	24·1 (22·4, 26)	287 (264, 313)	27·6 (25·6, 29·9)
**Egypt**	15723 (14418, 17127)	25·5 (23·4, 28)	25800 (23626, 28161)	25·9 (23·7, 28·4)
**Iran**	15581 (14708, 16459)	20·8 (19·6, 21·9)	16760 (15828, 17756)	23·9 (22·6, 25·3)
**Iraq**	5191 (4852, 5573)	23·8 (22·2, 25·7)	12634 (11740, 13567)	26·4 (24·6, 28·5)

**Jordan**	1209 (1113, 1320)	26·8 (24·6, 29·1)	3556 (3270, 3883)	32·8 (30·3, 35·7)
**Kuwait**	380 (353, 410)	22·6 (21·1, 24·3)	843 (779, 914)	27·6 (25·6, 29·7)
**Lebanon**	1112 (1037, 1197)	23·5 (21·8, 25·5)	2301 (2143, 2485)	28·1 (26, 30·4)
**Libya**	1171 (1093, 1256)	23·9 (22·3, 25·8)	1751 (1630, 1896)	28·1 (26, 30·4)
**Morocco**	6578 (6132, 7065)	23·3 (21·7, 25·2)	8971 (8330, 9694)	26·8 (24·9, 28·8)
**Palestine**	623 (581, 669)	24·5 (22·8, 26·5)	1510 (1404, 1627)	28·6 (26·5, 31)
**Oman**	547 (511, 584)	25·6 (23·8, 27·8)	899 (831, 975)	27·8 (25·8, 30·1)
**Qatar**	87 (81, 94)	24·6 (22·8, 26·6)	419 (381, 461)	27·7 (25·7, 30)
**Saudi Arabia**	4205 (3870, 4551)	22·4 (20·6, 24·4)	6548 (5901, 7173)	25·3 (23·1, 27·5)
**Sudan**	6096 (5679, 6539)	24·9 (23·2, 27)	12629 (11738, 13561)	27·6 (25·5, 29·7)
**Syria**	3766 (3522, 4048)	23·8 (22·2, 25·8)	5345 (4958, 5761)	27·5 (25·4, 29·7)
**Tunisia**	2645 (2442, 2866)	29·5 (26·9, 32·4)	3640 (3276, 4036)	34·3 (31·2, 37·7)
**Turkey**	10072 (9301, 10942)	16·5 (15·1, 18)	14515 (13116, 16042)	19·5 (17·8, 21·4)
**United Arab Emirates**	398 (370, 427)	24 (22·3, 26)	1279 (1155, 1407)	26·9 (24·9, 29)
**Yemen**	4249 (3977, 4536)	23·8 (22·2, 25·7)	8995 (8397, 9665)	26·1 (24·2, 28·1)
**South Asia**	135878 (129136, 142998)	10·8 (10·3, 11·4)	221471 (209587, 233518)	12·4 (11·7, 13)
**Bangladesh**	16352 (15261, 17486)	12·5 (11·7, 13·5)	20225 (18710, 21781)	13 (12·1, 14)
**Bhutan**	80 (74, 86)	12·3 (11·5, 13·3)	123 (114, 133)	13·6 (12·6, 14·7)
**India**	102849 (97857, 108113)	10·6 (10·1, 11·1)	169429 (160726, 178116)	12·4 (11·8, 13)
**Nepal**	2396 (2243, 2563)	10·6 (9·9, 11·4)	3781 (3511, 4074)	12·1 (11·2, 13)
**Pakistan**	14201 (13234, 15290)	11 (10·2, 12)	27912 (25762, 30058)	12·1 (11·2, 13·1)
**Southern sub-Saharan Africa**	17304 (16453, 18164)	31·5 (29·9, 33·1)	21563 (20495, 22668)	28·7 (27·3, 30·2)
**Botswana**	365 (344, 388)	25·8 (24, 27·8)	598 (557, 643)	28·1 (26·1, 30·4)
**Lesotho**	541 (508, 576)	27·4 (25·5, 29·5)	528 (493, 565)	27·3 (25·3, 29·3)
**Namibia**	326 (305, 348)	21·5 (19·9, 23·1)	587 (547, 625)	25 (23·2, 26·9)

**South Africa**	12221 (11664, 12831)	32·4 (30·8, 34)	14745 (14014, 15488)	28·3 (26·9, 29·7)
**Swaziland**	234 (221, 248)	26·3 (24·5, 28·2)	318 (297, 339)	28·1 (26, 30·1)
**Zimbabwe**	3617 (3399, 3841)	31·4 (29·2, 33·8)	4788 (4483, 5082)	31·1 (28·9, 33·4)
**Western sub-Saharan Africa**	72146 (68007, 76511)	36·1 (33·3, 39)	164206 (154385, 175152)	37·9 (35, 41·1)
**Benin**	2025 (1906, 2153)	38·7 (35·7, 42)	4803 (4513, 5125)	40·8 (37·8, 44·4)
**Burkina Faso**	4930 (4652, 5254)	45·3 (42·1, 49)	9583 (8976, 10190)	43·9 (40·5, 47·7)
**Cameroon**	3823 (3588, 4066)	35·6 (32·7, 38·7)	10414 (9753, 11115)	37·7 (34·7, 40·8)
**Cape Verde**	154 (144, 164)	40·2 (37·1, 43·6)	218 (201, 235)	42·5 (38·9, 46·1)
**Chad**	2789 (2626, 2975)	41·7 (38·5, 45·1)	6505 (6117, 6940)	39·3 (36·2, 42·6)
**Côte d'Ivoire**	4514 (4227, 4808)	35·4 (32·7, 38·3)	9212 (8639, 9840)	37·7 (34·7, 40·8)
**The Gambia**	402 (377, 427)	38·8 (35·8, 41·9)	905 (848, 964)	42·3 (39, 45·8)
**Ghana**	5010 (4708, 5314)	33·3 (30·7, 36·1)	10640 (9955, 11431)	37·3 (34·3, 40·6)
**Guinea**	2536 (2381, 2701)	38·4 (35·5, 41·4)	4651 (4367, 4970)	38 (35, 41·4)
**Guinea-Bissau**	439 (412, 465)	39·2 (36·4, 42·5)	726 (681, 774)	38·2 (35·3, 41·3)
**Liberia**	874 (821, 934)	40·1 (37, 43·6)	1950 (1830, 2084)	41·2 (38·1, 44·7)
**Mali**	4266 (4020, 4551)	45·4 (41·9, 49·2)	10044 (9403, 10674)	46·7 (43, 50·6)
**Mauritania**	791 (743, 842)	36·7 (33·9, 39·8)	1563 (1461, 1671)	39·6 (36·4, 43)
**Niger**	3862 (3632, 4106)	43·4 (40·1, 47·1)	10457 (9803, 11152)	44·6 (41, 48·4)
**Nigeria**	29208 (27402, 31171)	32·5 (29·9, 35·3)	70422 (65964, 75457)	34·9 (32, 38·1)
**São Tomé and Príncipe**	60 (57, 64)	45·3 (41·8, 49·1)	88 (82, 94)	45·5 (41·8, 49·3)
**Senegal**	3068 (2873, 3272)	37·2 (34·3, 40·2)	5974 (5590, 6400)	40·4 (37·1, 43·8)
**Sierra Leone**	1874 (1759, 2000)	46·3 (42·8, 50·1)	3217 (3018, 3445)	41·1 (38, 44·7)
**Togo**	1520 (1426, 1619)	38·9 (35·9, 42)	2833 (2638, 3049)	38·6 (35·6, 42)
**Eastern sub-Saharan Africa**	58349 (55165, 61561)	31·4 (29·2, 33·7)	124376 (117811, 131765)	33·4 (31, 35·9)
**Burundi**	1621 (1518, 1729)	31·1 (28·5, 33·9)	3289 (3078, 3522)	32·1 (29·4, 35)

**Comoros**	143 (134, 152)	32·1 (29·4, 35)	231 (215, 250)	34·8 (31·9, 38·1)
**Djibouti**	146 (137, 156)	32·2 (29·6, 35·1)	334 (311, 361)	34·7 (31·9, 38)
**Eritrea**	848 (795, 904)	31·3 (28·8, 34·1)	1764 (1650, 1893)	33·9 (31·1, 36·9)
**Ethiopia**	15026 (14311, 15798)	28·8 (27·1, 30·4)	31977 (30457, 33665)	30·6 (28·8, 32·2)
**Kenya**	7502 (7145, 7877)	35·5 (33·2, 37·7)	16015 (15244, 16865)	37·2 (34·8, 39·4)
**Madagascar**	3691 (3465, 3927)	31·9 (29·3, 34·8)	8142 (7623, 8716)	34 (31, 37·2)
**Malawi**	3183 (2989, 3399)	34·3 (31·5, 37·5)	5858 (5494, 6264)	36·4 (33·3, 39·7)
**Mozambique**	4357 (4086, 4655)	30·7 (28·1, 33·4)	9683 (9089, 10357)	34·3 (31·5, 37·6)
**Rwanda**	2395 (2234, 2571)	33·4 (30·4, 36·6)	3918 (3638, 4216)	33·2 (30·2, 36·5)
**Somalia**	2047 (1923, 2186)	29·6 (27·1, 32·3)	5169 (4851, 5537)	31·9 (29·3, 34·8)
**South Sudan**	1875 (1753, 2009)	33·1 (30·4, 36·2)	3330 (3126, 3566)	34·6 (31·7, 37·8)
**Tanzania**	7870 (7357, 8403)	31·4 (28·8, 34·4)	17122 (16061, 18326)	33·9 (31, 37·3)
**Uganda**	5215 (4894, 5558)	30·6 (28, 33·4)	12270 (11521, 13086)	33·1 (30·4, 36·1)
**Zambia**	2401 (2262, 2559)	32 (29·3, 35·1)	5196 (4867, 5542)	32·5 (29·8, 35·6)
**Central sub-Saharan Africa**	17252 (16231, 18413)	29·7 (27·5, 32·2)	39610 (37196, 42350)	31·9 (29·4, 34·6)
**Angola**	3268 (3064, 3490)	30·6 (28·2, 33·2)	9312 (8722, 9932)	32 (29·4, 34·6)
**Central African Republic**	761 (714, 812)	26·4 (24·5, 28·6)	1301 (1216, 1396)	27·8 (25·7, 30·2)
**Congo**	663 (622, 709)	26·1 (24, 28·2)	1320 (1233, 1416)	27·9 (25·7, 30·2)
**DR Congo**	12155 (11399, 13010)	30 (27·8, 32·5)	26771 (25120, 28631)	32·4 (29·9, 35·3)
**Equatorial Guinea**	128 (120, 136)	28·1 (25·8, 30·4)	422 (395, 453)	31·3 (28·8, 33·9)
**Gabon**	277 (259, 296)	27·5 (25·3, 29·9)	483 (450, 519)	29·5 (27·2, 32)

Appendix Table 21: Number of prevalent cases and age-standardised prevalence rates of compensated cirrhosis due to hepatitis B per 100 000 in 1990 and 2017 for both sexes by location

	1990		2017	
	**Counts (95% UI)**	**Rate (95% UI)**	**Counts (95% UI)**	**Rate (95% UI)**
**Global**	22476685 (21192220, 23949930)	461·8 (435·8, 491·2)	36666899 (34057354, 39502936)	451·9 (420, 485·9)
**High-income North America**	70334 (65293, 75497)	21·6 (20·1, 23·2)	93385 (86034, 100916)	19·4 (17·8, 20·9)
**Canada**	5183 (4616, 5772)	16·2 (14·4, 18)	8411 (7250, 9552)	16·6 (14·4, 18·9)
**Greenland**	8 (7, 9)	14·4 (12·8, 16·1)	11 (10, 13)	16·9 (14·6, 19·2)
**USA**	65141 (60509, 69943)	22·2 (20·7, 23·9)	84961 (78235, 91437)	19·7 (18·1, 21·2)
**Australasia**	30140 (27274, 32972)	134·8 (122·1, 147·4)	43338 (39065, 47734)	126·1 (113·8, 139·2)
**Australia**	24580 (22033, 27109)	131·5 (118·3, 144·8)	36170 (32323, 40171)	124·4 (111, 138·4)
**New Zealand**	5560 (5155, 5959)	151·3 (140·4, 162·3)	7168 (6602, 7759)	135·9 (125·5, 146·9)
**High-income Asia-Pacific**	1332253 (1224859, 1434949)	678·5 (623·3, 729·5)	1200685 (1099016, 1312735)	497·7 (454·3, 545·8)
**Brunei**	1529 (1400, 1652)	670·1 (618·3, 723)	3208 (2881, 3565)	669·1 (603·4, 736·2)
**Japan**	554533 (516166, 595096)	368·8 (343·5, 395)	570486 (528503, 615185)	334·7 (310·7, 363)
**Singapore**	30549 (27893, 33183)	885·4 (808·6, 961·8)	58675 (52407, 65035)	834·7 (746·9, 926·3)
**South Korea**	745642 (670761, 819043)	1669·2 (1500·1, 1830·2)	568317 (501176, 634130)	818·9 (725·4, 915·2)
**Western Europe**	620541 (561234, 684040)	137·3 (124, 151·8)	676099 (608191, 750868)	124·1 (111·1, 138·3)
**Andorra**	71 (63, 80)	107·2 (95·5, 120·2)	123 (109, 139)	113·3 (100·5, 127·8)
**Austria**	15775 (14151, 17607)	173·9 (155, 194·6)	16245 (14676, 17980)	145·1 (130·5, 161·4)
**Belgium**	14486 (11660, 17717)	121·7 (97·4, 150·6)	16281 (12990, 20022)	116·9 (93·2, 148·1)
**Cyprus**	657 (589, 732)	79·6 (71·4, 88·6)	1183 (1044, 1332)	73·3 (65, 82·4)
**Denmark**	5429 (4918, 5964)	88·7 (80·3, 97·6)	9098 (8200, 10010)	132·1 (118·6, 146·4)
**Finland**	5479 (4993, 6047)	90·6 (82, 100·1)	9515 (8625, 10456)	142·9 (128·5, 158·9)
**France**	86310 (77911, 95863)	130·7 (117·8, 145·2)	85271 (76650, 94116)	109·7 (97·6, 121·8)

**Germany**	122730 (111892, 134829)	126·3 (114·1, 138·6)	141528 (126748, 156448)	133·1 (118·2, 147·8)
**Greece**	17807 (16042, 19661)	146·8 (132·2, 163)	16487 (14474, 18693)	122·1 (106·3, 139·7)
**Iceland**	127 (111, 146)	47·6 (41·5, 54·6)	306 (267, 349)	79·2 (69, 91·2)
**Ireland**	2470 (2231, 2717)	68·2 (61·5, 75·1)	6074 (5448, 6747)	105·4 (94·8, 117·5)
**Israel**	5106 (4609, 5603)	108·2 (98·1, 118·9)	10361 (9152, 11641)	112·3 (99·4, 126·3)
**Italy**	183803 (163656, 205217)	268·7 (238, 302·6)	167813 (146851, 191109)	206·8 (180·5, 237·5)
**Luxembourg**	823 (743, 903)	173·3 (156·6, 190·7)	1138 (1000, 1280)	151·4 (132·6, 171·1)
**Malta**	303 (273, 336)	71·5 (64·7, 79·3)	377 (337, 419)	68·4 (60·9, 76·3)
**Netherlands**	15901 (14423, 17537)	89·9 (81·5, 99·3)	21819 (19597, 24021)	104·5 (93·3, 115·6)
**Norway**	5198 (4841, 5598)	105·7 (98·2, 113·9)	7547 (6981, 8148)	119·8 (110·6, 129·1)
**Portugal**	17393 (15524, 19508)	154·5 (137·5, 173·6)	11931 (10441, 13453)	87·2 (76·3, 98·6)
**Spain**	64656 (52513, 78819)	147·1 (118·4, 181·4)	61142 (49354, 74698)	100·9 (81, 125·2)
**Sweden**	9723 (8962, 10577)	95·4 (87·6, 103·9)	16755 (15277, 18161)	139 (126·3, 151·8)
**Switzerland**	7127 (6491, 7844)	84 (76·6, 92·6)	10266 (9239, 11404)	93·7 (84·5, 104·1)
**United Kingdom**	38568 (35227, 42110)	57·9 (52·7, 63·5)	64139 (58383, 70063)	79·9 (72·3, 87·7)
**Eastern Europe**	757805 (706047, 810099)	299·3 (278·9, 320·2)	986353 (912112, 1065280)	383·2 (354·5, 414·9)
**Belarus**	28791 (25690, 32012)	249·5 (221·2, 278·2)	37745 (33372, 42504)	322·4 (283·6, 363·8)
**Estonia**	4355 (3885, 4859)	249·4 (222·2, 279·3)	4710 (4191, 5298)	295·4 (260·8, 333·7)
**Latvia**	7146 (6387, 7957)	234·2 (208·2, 261·1)	6882 (6113, 7711)	289·5 (255·7, 326·2)
**Lithuania**	11289 (10048, 12561)	274·7 (244·3, 306·1)	12229 (10815, 13774)	357·1 (316·4, 404)
**Moldova**	30187 (26913, 33648)	654·9 (584·7, 729·8)	24529 (21639, 27726)	524·6 (463·7, 595)
**Russia**	497145 (463511, 531884)	294·5 (274·4, 315·1)	671039 (620299, 722651)	375·8 (347·3, 405·5)
**Ukraine**	178892 (166278, 191617)	302·4 (281, 323·9)	229219 (211712, 248744)	416·2 (383·7, 450·9)
**Central Europe**	658705 (600591, 720759)	482 (439, 527·7)	624506 (565914, 688710)	436·1 (393·6, 482·6)
**Albania**	12492 (11298, 13697)	434 (394·7, 474·4)	13493 (12092, 14927)	424·2 (380, 472·1)

**Bosnia and Herzegovina**	23408 (21251, 25682)	482·7 (438·9, 528·8)	18140 (16246, 20125)	427·4 (382·8, 473·9)
**Bulgaria**	43168 (39314, 47479)	418 (379·6, 461·1)	37017 (33309, 40996)	414·9 (371·9, 464·4)
**Croatia**	34665 (31399, 38077)	602·6 (545·7, 661)	22683 (20394, 25003)	421·5 (378·7, 467)
**Czech Republic**	44530 (40185, 48919)	380·8 (344·1, 418·5)	45524 (41078, 50644)	338·8 (305·3, 379)
**Hungary**	80406 (72616, 88126)	667·5 (602·2, 731·2)	56828 (50456, 63501)	464·8 (412·9, 522·1)
**Macedonia**	7224 (6518, 7986)	345·6 (312, 382·4)	9948 (8875, 11083)	370·7 (330·7, 412·6)
**Montenegro**	2066 (1868, 2275)	315·9 (284·8, 347·9)	2509 (2257, 2790)	338·3 (303·6, 378)
**Poland**	173592 (157215, 191236)	410·5 (372·8, 453·4)	208605 (187339, 231913)	435·7 (388·9, 484·7)
**Romania**	155877 (141564, 170651)	614·4 (556·5, 675·4)	132177 (119009, 146401)	539·7 (486, 599·3)
**Serbia**	30203 (27348, 33394)	284·7 (256·5, 315·8)	27817 (24935, 30762)	260·9 (233·1, 290·2)
**Slovakia**	37073 (33471, 40786)	656·3 (593·8, 722·1)	39077 (34965, 43305)	572·8 (510, 634·9)
**Slovenia**	14003 (12685, 15506)	611·8 (553·4, 677·3)	10687 (9626, 11807)	402·1 (362·6, 446·4)
**Central Asia**	165940 (152290, 179671)	276·5 (254·2, 299·1)	290109 (263535, 319352)	307·4 (280·3, 338·3)
**Armenia**	7083 (6517, 7707)	212·8 (196·2, 231·4)	8730 (7904, 9567)	247·9 (224·3, 272·1)
**Azerbaijan**	17468 (16031, 19030)	269·5 (247·7, 293·3)	32031 (28902, 35468)	279·2 (252·7, 308·3)
**Georgia**	14631 (13441, 15965)	250 (229·1, 272·7)	10530 (9331, 11667)	248·5 (219·3, 275·9)
**Kazakhstan**	33349 (30383, 36305)	207·8 (189·7, 226)	55138 (49752, 61021)	287·2 (259·4, 317·6)
**Kyrgyzstan**	12091 (11065, 13115)	329·7 (302·1, 357·5)	20558 (18564, 22711)	335·4 (303·9, 369·1)
**Mongolia**	5829 (5307, 6406)	370·2 (337·4, 404·6)	10650 (9588, 11873)	306 (276, 340)
**Tajikistan**	10691 (9793, 11631)	270·1 (248·3, 292·8)	22793 (20621, 25294)	271·8 (246·1, 299·9)
**Turkmenistan**	10033 (9196, 10902)	351·6 (323·6, 382)	19336 (17581, 21292)	371·7 (338·8, 408·6)
**Uzbekistan**	54764 (50163, 59333)	340·7 (312·7, 368)	110343 (100113, 121203)	336·6 (305·7, 368·7)
**Central Latin America**	152534 (139327, 166253)	127·8 (116·7, 139·3)	315549 (286291, 347568)	124 (112·6, 136·2)
**Colombia**	18688 (16361, 21183)	73·6 (65, 83·1)	44400 (38908, 50339)	84·1 (73·7, 95·2)
**Costa Rica**	2054 (1801, 2321)	87·1 (76·5, 98·2)	5232 (4566, 5933)	102·8 (89·9, 116·6)

**El Salvador**	4288 (3763, 4878)	114·4 (100·6, 130·2)	7278 (6345, 8345)	125 (108·7, 143·3)
**Guatemala**	8024 (7046, 9120)	151·6 (133·3, 171·3)	21590 (18849, 24806)	157·4 (138, 180·4)
**Honduras**	4445 (3896, 5075)	151·8 (133·2, 172·2)	11390 (9866, 13117)	151·8 (131·5, 174·2)
**Mexico**	98599 (91034, 106397)	161·7 (149·6, 174·4)	183113 (167984, 199559)	142·9 (131·3, 155·4)
**Nicaragua**	2442 (2147, 2793)	105 (92·5, 118·9)	6398 (5585, 7386)	113·2 (99, 130·4)
**Panama**	1518 (1338, 1707)	79·3 (70, 88·9)	3821 (3341, 4334)	95·2 (83·3, 107·9)
**Venezuela**	12476 (10917, 14181)	87·6 (77·4, 99·5)	32327 (28347, 37090)	101 (88·6, 115·5)
**Andean Latin America**	60065 (53452, 67363)	214·8 (191·1, 240·6)	140640 (123823, 158423)	239·2 (210·5, 269·2)
**Bolivia**	9595 (8480, 10845)	215 (189·2, 241·4)	25533 (22155, 29362)	249·7 (216·1, 286·9)
**Ecuador**	16178 (14414, 18093)	219·5 (196·3, 244·1)	39738 (35104, 44808)	248·3 (219·6, 279·4)
**Peru**	34291 (30313, 38635)	212·5 (187·7, 239·6)	75369 (65914, 84977)	231·5 (202·4, 260·6)
**Caribbean**	38941 (35960, 42228)	129·1 (119, 140·2)	69862 (63396, 76430)	140·8 (127·6, 154·3)
**Antigua and Barbuda**	66 (60, 72)	122·2 (111·6, 133·5)	125 (114, 137)	119·4 (108·5, 130·9)
**The Bahamas**	296 (269, 327)	135·7 (123·7, 148·9)	550 (498, 604)	129·4 (117·4, 142·2)
**Barbados**	274 (250, 300)	106 (96·8, 115·9)	424 (385, 467)	113·3 (102·7, 125)
**Belize**	157 (144, 171)	132 (121·2, 143·8)	516 (469, 567)	146·6 (133·6, 161·1)
**Bermuda**	71 (65, 78)	102·6 (92·9, 112·5)	108 (96, 119)	119·7 (106·8, 132·4)
**Cuba**	10932 (9940, 11964)	100·3 (91·2, 109·7)	18420 (16747, 20271)	124·2 (112·8, 136·1)
**Dominica**	66 (60, 71)	104·7 (95·3, 114·1)	96 (87, 106)	123·7 (111·9, 136·9)
**Dominican Republic**	8142 (7439, 8924)	155·9 (142·5, 170·5)	15440 (13884, 17111)	148·8 (134, 164·7)
**Grenada**	87 (79, 95)	131·5 (119·5, 144·1)	150 (136, 163)	124 (112, 135·6)
**Guyana**	1032 (936, 1130)	179·7 (163·7, 196·5)	1166 (1052, 1294)	160·9 (145·3, 178·7)
**Haiti**	5727 (5179, 6321)	125·7 (113·8, 138·9)	16826 (14909, 18921)	167·3 (149, 187)
**Jamaica**	1764 (1608, 1920)	94·3 (85·8, 102·8)	3266 (2952, 3602)	110·9 (100, 122·2)
**Puerto Rico**	6866 (6264, 7452)	189·2 (172·7, 205·4)	6605 (6003, 7241)	141·8 (129·3, 155·3)

**Saint Lucia**	134 (123, 147)	133·8 (122·3, 146·3)	257 (233, 284)	124·3 (112·7, 136·6)
**Saint Vincent and the Grenadines**	90 (82, 98)	111·5 (101·8, 122)	148 (133, 164)	116·8 (105·1, 129·1)
**Suriname**	472 (431, 513)	149·2 (136·6, 162·5)	963 (870, 1067)	156·5 (141·2, 172·9)
**Trinidad and Tobago**	1290 (1178, 1402)	122·9 (112·4, 133·5)	2105 (1891, 2323)	125·6 (113·1, 138·7)
**Virgin Islands**	142 (130, 156)	132·2 (120·7, 144·1)	186 (169, 205)	140 (126·7, 154·4)
**Tropical Latin America**	251013 (232973, 268541)	197·9 (184·2, 211·4)	387413 (358276, 419850)	157·2 (145·5, 170·1)
**Brazil**	246863 (229092, 264089)	199·3 (185·6, 212·8)	377339 (348829, 409328)	157·1 (145·5, 170·2)
**Paraguay**	4149 (3650, 4687)	140·9 (124·4, 158·8)	10074 (8819, 11434)	158·6 (139, 179·5)
**Southern Latin America**	87970 (81187, 94706)	184·4 (170·2, 198·5)	141633 (130168, 153571)	193·8 (178·1, 210·4)
**Argentina**	50917 (46802, 55014)	157·8 (145, 170·5)	87129 (79558, 95186)	181·4 (165·5, 198·6)
**Chile**	32124 (29666, 34766)	266 (246, 286·9)	47870 (43864, 51925)	226·5 (207·7, 246)
**Uruguay**	4924 (4544, 5304)	148·9 (137·2, 160·3)	6627 (6065, 7237)	168·8 (154·2, 185·1)
**East Asia**	12786010 (12128637, 13557521)	1080·8 (1023·6, 1143·9)	20551191 (19189803, 21983487)	1064·2 (995·6, 1137·4)
**China**	12120402 (11491947, 12855743)	1078·8 (1021·2, 1142·1)	19402687 (18113823, 20757376)	1056·2 (989, 1129·1)
**North Korea**	184738 (173011, 197970)	922 (865, 985·2)	386728 (358284, 416113)	1243 (1151, 1335·2)
**Taiwan (Province of China)**	267853 (251108, 285890)	1339·7 (1257·7, 1429·5)	430708 (397912, 465421)	1339·7 (1241, 1442·2)
**Southeast Asia**	614496 (560146, 672495)	165·3 (150·2, 181·7)	1308329 (1179061, 1455088)	185·3 (167·7, 205)
**Cambodia**	14057 (12885, 15242)	204·8 (187·8, 222·9)	35293 (31966, 38923)	238·3 (215·9, 262·7)
**Indonesia**	255224 (229662, 282681)	171·4 (154·4, 189·8)	376117 (336318, 423076)	137·9 (124, 154·1)
**Laos**	4957 (4526, 5412)	169·3 (154, 185)	13395 (12006, 14835)	216·6 (195·4, 239·6)
**Malaysia**	20414 (18247, 22737)	144·3 (128·7, 160)	62935 (55068, 71376)	200·7 (175·5, 227·1)
**Maldives**	167 (153, 182)	117·7 (107·9, 127·5)	739 (669, 821)	147·4 (133·7, 162·4)
**Mauritius**	1852 (1698, 2004)	182·4 (167, 197·2)	2406 (2182, 2653)	153·4 (139·6, 168·7)
**Myanmar**	70257 (64214, 76241)	219·3 (200·2, 237·9)	125892 (113426, 139631)	229·6 (207·8, 253·8)
**Philippines**	35850 (32862, 38943)	74 (68·4, 80·2)	111283 (99208, 122552)	116·2 (103·6, 127·6)

**Sri Lanka**	24435 (22496, 26522)	159·9 (147·5, 173·3)	42044 (37976, 46346)	172·8 (156·7, 190·3)
**Seychelles**	90 (83, 98)	148·5 (136·2, 161·1)	227 (204, 254)	188·3 (170·7, 209·7)
**Thailand**	72612 (61818, 85092)	140·4 (119·2, 164·5)	155336 (128611, 185075)	168 (140·4, 199·2)
**East Timor**	836 (763, 920)	149·6 (136·6, 163·6)	2048 (1851, 2262)	213·9 (193·3, 236·9)
**Vietnam**	112929 (103700, 122548)	224·6 (205·4, 243·8)	378893 (343858, 417401)	350·5 (319·6, 384·5)
**Oceania**	17420 (16229, 18680)	345·1 (321·7, 369·4)	41785 (38524, 45235)	379·5 (350·1, 409·5)
**American Samoa**	112 (104, 121)	290·6 (269·2, 313·5)	172 (157, 189)	330·1 (300·9, 360·5)
**Federated States of Micronesia**	294 (272, 317)	396·3 (367·4, 425·4)	362 (332, 394)	376 (345·9, 408·5)
**Fiji**	1458 (1353, 1565)	227·9 (212·3, 244·1)	2612 (2412, 2831)	285·7 (264·1, 309·5)
**Guam**	492 (457, 530)	386·4 (359, 415)	708 (653, 767)	403·5 (372·9, 436·7)
**Kiribati**	232 (215, 251)	388 (358·8, 417·6)	434 (399, 472)	416·8 (383·8, 453·4)
**Marshall Islands**	120 (111, 129)	398·2 (369·2, 427·8)	212 (194, 230)	404·1 (371·8, 438·5)
**Northern Mariana Islands**	168 (156, 182)	381·1 (356, 410·7)	212 (195, 231)	401·9 (370·8, 434·9)
**Papua New Guinea**	11293 (10504, 12145)	356·6 (331·9, 383·8)	30671 (28221, 33262)	386 (354·6, 416·7)
**Samoa**	439 (408, 475)	360·9 (335·7, 389)	643 (592, 698)	382·6 (351·9, 415·4)
**Solomon Islands**	847 (788, 911)	366·8 (341·1, 393·8)	2011 (1852, 2189)	381·3 (352·4, 412·9)
**Tonga**	281 (262, 302)	387·9 (361·9, 417·3)	365 (337, 397)	396·8 (366·7, 430·8)
**Vanuatu**	534 (494, 579)	482·5 (445·6, 522·5)	1081 (990, 1177)	452·8 (415·9, 492·5)
**North Africa and Middle East**	1308356 (1173967, 1448144)	524·2 (473·5, 580·8)	2619892 (2350719, 2927757)	458·8 (412·6, 509·7)
**Afghanistan**	40715 (37432, 44174)	554·9 (511·2, 602·2)	123530 (112601, 135173)	588 (537·8, 643·1)
**Algeria**	80087 (73814, 86631)	448·1 (414, 484·9)	175564 (161110, 191441)	426·3 (391·8, 462·6)
**Bahrain**	2197 (2018, 2385)	486·1 (449·1, 526·4)	7577 (6902, 8298)	413·2 (378, 447·3)
**Egypt**	369401 (305326, 438593)	879·6 (728·8, 1035·7)	515902 (419441, 627425)	607·4 (495·7, 737·5)
**Iran**	151291 (137933, 164915)	365·9 (335·3, 398·2)	335619 (303231, 369709)	376·5 (341·5, 412·4)
**Iraq**	47841 (44242, 51722)	403·7 (375·5, 435·8)	127438 (116544, 138553)	359 (329·4, 389·3)

**Jordan**	12111 (11150, 13102)	487·6 (449·5, 525·4)	43835 (40121, 47743)	467 (428·4, 507·8)
**Kuwait**	5383 (4948, 5830)	333·8 (308·4, 360)	16899 (15400, 18518)	336 (308·3, 365·8)
**Lebanon**	14248 (13148, 15352)	466·6 (430·7, 501·8)	38422 (35261, 41690)	483·5 (444·5, 523·4)
**Libya**	12974 (11979, 14023)	446 (413·4, 481·3)	29958 (27324, 32908)	428·2 (393·1, 468·3)
**Morocco**	89326 (82848, 96777)	453·2 (418·7, 489·7)	157057 (143272, 170753)	431·6 (394·4, 468·7)
**Palestine**	5543 (5121, 6004)	430·4 (399·9, 464)	16286 (14857, 17744)	423·8 (387·7, 460·2)
**Oman**	7124 (6570, 7743)	491·6 (454·4, 531)	21193 (19260, 23185)	426·1 (389·2, 462·3)
**Qatar**	2148 (1955, 2359)	487·7 (448·7, 529·3)	14833 (13481, 16294)	440·6 (402·2, 479·9)
**Saudi Arabia**	35574 (28808, 43427)	313·1 (257·5, 377)	99978 (78771, 126116)	269 (218·2, 328·3)
**Sudan**	76686 (70691, 82818)	556 (513·5, 600·2)	154799 (141351, 169842)	526·5 (482, 576·7)
**Syria**	36065 (33401, 38851)	434·8 (404·5, 468·4)	69555 (63675, 75766)	431·6 (395·1, 468·4)
**Tunisia**	22026 (16796, 28141)	336·3 (256·5, 431·4)	41462 (31004, 54594)	322·2 (241·3, 420·4)
**Turkey**	246859 (224594, 269545)	518·4 (472·8, 563·9)	457627 (413399, 504038)	513·5 (464·7, 564·8)
**United Arab Emirates**	8319 (7603, 9115)	471 (431·9, 509·8)	64606 (57999, 72157)	463·7 (423·1, 507·5)
**Yemen**	41597 (38296, 45076)	508 (469·3, 548·2)	105304 (95494, 115363)	477·5 (434·6, 521)
**South Asia**	1540870 (1422865, 1663771)	170·1 (157, 183·9)	3093938 (2833834, 3386833)	179·2 (164·3, 195·3)
**Bangladesh**	167898 (154404, 181832)	213·4 (197, 230·7)	249089 (227394, 272647)	163·5 (149·7, 178·7)
**Bhutan**	918 (844, 999)	224·3 (205·7, 244·1)	1687 (1522, 1861)	174·1 (157·8, 190·7)
**India**	1203408 (1113263, 1296481)	163·9 (151·3, 176·4)	2517500 (2311990, 2744670)	184·2 (168·7, 200·4)
**Nepal**	21376 (18271, 25142)	144·7 (124·2, 169·3)	32548 (27164, 38563)	119·7 (100·3, 141·4)
**Pakistan**	147272 (127847, 169572)	190·3 (164·4, 219·4)	293114 (250317, 343625)	164 (140·2, 191·1)
**Southern sub-Saharan Africa**	123038 (112266, 134573)	315·5 (287·6, 342·8)	148187 (133628, 164291)	205 (185·4, 226·2)
**Botswana**	2257 (1995, 2563)	264·6 (235·9, 297)	4655 (4090, 5320)	220·1 (193·8, 249·6)
**Lesotho**	3849 (3425, 4362)	301·6 (267·8, 340·3)	4048 (3545, 4636)	242·3 (213·1, 277·3)
**Namibia**	1976 (1738, 2245)	205·4 (181·9, 231·6)	4454 (3862, 5138)	228·9 (199·3, 262·2)

**South Africa**	87503 (80240, 95122)	300·5 (275·9, 326)	99303 (90602, 108702)	181·7 (165·5, 197·8)
**Swaziland**	1527 (1342, 1734)	316·3 (279·1, 357·6)	2176 (1905, 2475)	248 (218·7, 281·1)
**Zimbabwe**	25926 (22884, 29150)	412·7 (367·1, 461·5)	33552 (29189, 38426)	308·3 (270·8, 350·9)
**Western sub-Saharan Africa**	1114698 (1021113, 1209023)	869·3 (800·1, 941·8)	2344260 (2116621, 2576909)	804·9 (730·1, 884·4)
**Benin**	26973 (24698, 29319)	919·9 (847·3, 996·1)	61323 (55567, 67581)	804·7 (735·5, 882·1)
**Burkina Faso**	64821 (59686, 69897)	1089 (1005·5, 1166·8)	132379 (119129, 146290)	952·7 (861·8, 1049·1)
**Cameroon**	58015 (52837, 63022)	861·8 (790·6, 934·7)	144561 (130678, 159436)	757·8 (686·4, 832·2)
**Cape Verde**	1616 (1489, 1744)	692·7 (641·2, 747·1)	3461 (3152, 3776)	666·5 (607·5, 726·4)
**Chad**	37502 (34453, 40542)	993·4 (913·3, 1069·9)	83587 (76037, 91850)	959 (877·8, 1042·8)
**Côte d'Ivoire**	67249 (61300, 73343)	870·1 (797·6, 941·4)	147118 (132840, 161516)	801·1 (729·7, 875·6)
**The Gambia**	5332 (4871, 5780)	877·2 (807·1, 943·9)	12116 (10985, 13263)	832·4 (757·5, 908·9)
**Ghana**	77128 (70335, 83765)	765·5 (700·9, 826·6)	168632 (151048, 186280)	706·1 (634·7, 778·4)
**Guinea**	39786 (36483, 43094)	938·3 (863·5, 1015·8)	70210 (63603, 77338)	896·3 (816·3, 982·2)
**Guinea-Bissau**	6265 (5767, 6777)	1001 (924·9, 1079·3)	11312 (10258, 12471)	897·6 (821·7, 978)
**Liberia**	13838 (12722, 15005)	999·7 (919·7, 1080·9)	32572 (29578, 35760)	954 (871·3, 1041·7)
**Mali**	64854 (59658, 70388)	1136·8 (1048, 1225·9)	134063 (121282, 146568)	1078·1 (982, 1177·7)
**Mauritania**	11186 (10259, 12172)	809·3 (744·6, 878·5)	20676 (18820, 22659)	746·1 (679·1, 817·4)
**Niger**	49575 (45519, 53783)	1035 (955·6, 1119·4)	134562 (122220, 147569)	1124·7 (1026, 1232·2)
**Nigeria**	496203 (451687, 545331)	795 (723·8, 870·6)	996134 (891730, 1104468)	731·2 (658·4, 806·5)
**São Tomé and Príncipe**	937 (858, 1012)	1209·4 (1111·7, 1310·6)	1603 (1457, 1763)	1024·9 (936·2, 1122·7)
**Senegal**	40214 (36858, 43692)	849 (782·2, 917·2)	90773 (82612, 99726)	872·3 (796·1, 956·6)
**Sierra Leone**	32549 (29913, 35163)	1231·8 (1136·5, 1324·1)	49874 (45045, 54961)	907·6 (823·3, 996·4)
**Togo**	20624 (18989, 22457)	940·3 (869·1, 1016·5)	49281 (44689, 54301)	867·6 (791·2, 951·5)
**Eastern sub-Saharan Africa**	530321 (473179, 592646)	447·7 (401·2, 501·7)	1095551 (969740, 1241424)	417·4 (370·2, 470·5)
**Burundi**	15592 (13770, 17670)	437 (387·4, 493)	26776 (23381, 30610)	364·6 (322, 414·8)

**Comoros**	1086 (956, 1218)	366 (322·6, 411·8)	2272 (1996, 2586)	370·9 (325·8, 420·5)
**Djibouti**	1126 (991, 1267)	353·5 (313·6, 395·2)	3703 (3259, 4205)	371·4 (329·3, 419·1)
**Eritrea**	7574 (6658, 8558)	421·7 (374·4, 474·8)	16381 (14283, 18701)	380·3 (334·3, 428·8)
**Ethiopia**	150647 (134702, 168092)	473·9 (424·5, 528·2)	306992 (272853, 343905)	458·9 (409·9, 513·3)
**Kenya**	90087 (80547, 100634)	690·5 (615·9, 770·8)	196120 (172968, 223443)	568·8 (502·7, 642·6)
**Madagascar**	30612 (27137, 34280)	394·2 (351·4, 438·9)	65069 (57128, 73719)	359·1 (316·4, 405·5)
**Malawi**	30529 (27055, 34232)	493·3 (438·2, 550·2)	49576 (43244, 56416)	425 (372·6, 481)
**Mozambique**	37835 (33480, 42249)	397·5 (354·8, 442·6)	79136 (68982, 90042)	414·3 (364·1, 468·7)
**Rwanda**	13050 (9889, 16955)	288·2 (220·5, 370·4)	18721 (14016, 24256)	205·3 (156, 264·8)
**Somalia**	17433 (15394, 19821)	366·5 (325·8, 412·1)	45733 (40045, 52387)	411·2 (361·5, 470·5)
**South Sudan**	15889 (14141, 17910)	414·1 (369·1, 464·8)	28438 (25071, 32232)	437·7 (387·7, 494·8)
**Tanzania**	58388 (51922, 65416)	360·7 (321·7, 402·4)	128035 (112423, 145773)	344·6 (303·6, 391)
**Uganda**	38304 (33934, 43052)	374·8 (332·4, 419·5)	83345 (73393, 95200)	352·4 (312·3, 400·5)
**Zambia**	21902 (19413, 24605)	466·9 (414·4, 523·3)	44566 (39045, 50337)	382·6 (338·6, 430·3)
**Central sub-Saharan Africa**	215235 (193438, 238565)	598·6 (539·1, 658)	494194 (440810, 556276)	580·7 (520·1, 649·9)
**Angola**	43924 (39336, 48877)	649·3 (582·5, 721·4)	103250 (91290, 116676)	557·1 (494·4, 625·7)
**Central African Republic**	10066 (9025, 11190)	540·3 (486·7, 596·9)	19951 (17630, 22582)	565·9 (503·4, 633·7)
**Congo**	8359 (7490, 9275)	523·5 (467·5, 580·2)	20434 (18073, 23066)	497·2 (441·9, 554·8)
**DR Congo**	147529 (132488, 163985)	596·5 (537·4, 656·3)	339699 (302699, 382376)	600·4 (537·2, 671·2)
**Equatorial Guinea**	1468 (1315, 1629)	523·3 (470·4, 580·1)	4243 (3741, 4791)	475·8 (425, 534·4)
**Gabon**	3889 (3488, 4320)	549·9 (493·4, 611·2)	6616 (5868, 7463)	452·3 (401·5, 507)

Appendix Table 22: Number of prevalent cases and age-standardised prevalence rates of compensated cirrhosis due to hepatitis C per 100 000 in 1990 and 2017 for both sexes by location

	1990		2017	
	**Counts (95% UI)**	**Rate (95% UI)**	**Counts (95% UI)**	**Rate (95% UI)**
**Global**	15562947 (14472714, 16699392)	327 (303·9, 349·7)	27719000 (25515383, 29987680)	341·1 (314·1, 368·7)
**High-income North America**	395271 (370358, 419677)	122·9 (115·2, 130·9)	615533 (573242, 656658)	125·8 (117·8, 134·3)
**Canada**	29464 (27176, 31952)	92·7 (85·4, 100·5)	49471 (44713, 54307)	96·4 (87·2, 105·6)
**Greenland**	45 (41, 50)	82·2 (74·9, 90)	67 (60, 74)	94·9 (86·2, 104·5)
**USA**	365753 (342776, 388506)	126·3 (118·4, 134·3)	565985 (528950, 603277)	129·3 (121·2, 137·9)
**Australasia**	32256 (29470, 35150)	145 (132·5, 157·8)	54582 (49599, 59667)	157·9 (143·4, 172·9)
**Australia**	26465 (23891, 29100)	142·3 (128·3, 156·5)	45790 (41393, 50345)	156·6 (141·3, 172·5)
**New Zealand**	5791 (5405, 6166)	158·5 (147·8, 168·6)	8792 (8170, 9450)	165·1 (153·2, 177·9)
**High-income Asia-Pacific**	2243865 (2136722, 2341918)	1110·4 (1056·8, 1159)	2302801 (2170683, 2429765)	893·1 (845·5, 939·8)
**Brunei**	812 (740, 888)	377·5 (347·4, 407·4)	2137 (1917, 2354)	450·4 (407·8, 493)
**Japan**	2069200 (1973830, 2155855)	1349 (1288, 1406·9)	2125771 (2010625, 2242541)	1231·3 (1168·8, 1296·4)
**Singapore**	6650 (5541, 7899)	196·6 (165·3, 230·5)	15046 (12476, 17821)	212·6 (176·9, 250·9)
**South Korea**	167203 (143923, 192074)	383·3 (331·6, 439·3)	159848 (138247, 181533)	222·8 (192·1, 253·4)
**Western Europe**	1628087 (1499187, 1756754)	356·4 (327·4, 385·3)	1913716 (1759252, 2071518)	342 (314·3, 371·2)
**Andorra**	135 (122, 149)	206·7 (187·4, 228·7)	280 (253, 308)	250·8 (227·2, 276·7)
**Austria**	33381 (30302, 37269)	365·9 (331·4, 410·8)	39474 (36154, 42987)	345·1 (315·4, 375·8)
**Belgium**	32910 (26783, 39555)	274·7 (223·3, 331·2)	43538 (34975, 52195)	308 (245·6, 372·5)
**Cyprus**	1264 (1153, 1391)	154·1 (140·4, 169·3)	2625 (2381, 2870)	162·3 (147, 177·3)
**Denmark**	10449 (9599, 11335)	169·9 (155·6, 184·1)	17812 (16276, 19463)	253 (229·7, 277·2)
**Finland**	10595 (9732, 11524)	174·3 (159·5, 189·5)	18638 (17080, 20215)	274·6 (250·7, 297·7)
**France**	170936 (156796, 186197)	258·6 (236·1, 282·3)	183147 (167490, 199859)	230·8 (211, 253·7)

**Germany**	242259 (223903, 261617)	245·8 (226·6, 265·8)	321476 (294182, 350478)	295·4 (269·5, 322·6)
**Greece**	25443 (23468, 27641)	206·7 (190, 224·8)	27006 (24524, 29578)	197·8 (179·5, 217·2)
**Iceland**	135 (92, 191)	51·4 (34·9, 72·7)	339 (228, 474)	84·4 (56·7, 121·4)
**Ireland**	4759 (4352, 5159)	131·8 (120·5, 142·8)	11797 (10702, 13016)	202 (183·5, 222·9)
**Israel**	9849 (9077, 10652)	211·2 (194·1, 228·1)	23140 (21004, 25338)	250 (226·9, 274·4)
**Italy**	727076 (676476, 777237)	1048·3 (971·5, 1119·8)	784482 (725669, 845896)	947·7 (878·7, 1021·3)
**Luxembourg**	1605 (1471, 1742)	336·4 (307·9, 366·3)	2570 (2323, 2845)	336·6 (304·2, 373·1)
**Malta**	585 (536, 636)	137·9 (126·7, 149·9)	816 (744, 895)	146·2 (132·7, 160·2)
**Netherlands**	30137 (27776, 32675)	170·7 (157·1, 185·1)	43172 (39148, 47236)	201·5 (182·5, 220·3)
**Norway**	10108 (9415, 10790)	206·3 (191·7, 220·4)	14866 (13762, 16032)	231·9 (214·4, 250·1)
**Portugal**	33778 (30467, 37449)	296·7 (266·6, 330)	26782 (24025, 29715)	191·8 (171·1, 211·9)
**Spain**	201755 (167950, 239298)	455·5 (376·7, 541·9)	216845 (177713, 257342)	352·9 (289·5, 418·6)
**Sweden**	17057 (14654, 19446)	164·2 (140·1, 188·5)	29471 (25420, 33871)	237·8 (202·7, 274)
**Switzerland**	13775 (12637, 14880)	161·9 (148·4, 175·3)	20194 (18340, 22030)	180·6 (164·2, 197·6)
**United Kingdom**	48526 (44207, 53154)	72·6 (65·8, 79·8)	83262 (75677, 91977)	102·1 (92·1, 112·7)
**Eastern Europe**	1091132 (1023112, 1158511)	425·2 (397·8, 451·3)	1716143 (1599028, 1835689)	649·3 (604, 694)
**Belarus**	41330 (37196, 45555)	353·1 (315, 390·4)	66749 (59753, 74069)	552·8 (493·3, 616·6)
**Estonia**	6302 (5665, 6936)	353·4 (317·8, 389·7)	8304 (7435, 9207)	504·5 (449·2, 561·6)
**Latvia**	10373 (9302, 11457)	332·5 (296·4, 367·5)	12165 (10938, 13443)	493·5 (442, 548·8)
**Lithuania**	16127 (14615, 17818)	387·8 (349·7, 428·4)	21585 (19373, 23721)	607·2 (542·5, 670·8)
**Moldova**	45666 (41192, 50274)	990·6 (892·4, 1093)	43007 (38375, 47716)	902·3 (802·7, 1005·1)
**Russia**	712269 (667053, 755613)	417·3 (390·3, 442·7)	1180813 (1097179, 1262470)	644·4 (601·5, 688·4)
**Ukraine**	259065 (241358, 276342)	428·6 (399·9, 457·9)	383520 (355168, 412657)	678·1 (626·4, 730·2)
**Central Europe**	400664 (356575, 444427)	289·2 (257·1, 320·5)	461992 (410318, 513811)	314 (278·1, 350·2)
**Albania**	7185 (6344, 8022)	256·3 (226·9, 285·4)	10028 (8856, 11202)	308·5 (272·1, 346·8)

**Bosnia and Herzegovina**	13899 (12269, 15507)	286·3 (252·8, 318·9)	13442 (11853, 15006)	306·6 (269·4, 343·8)
**Bulgaria**	25835 (22952, 28746)	243·4 (216·6, 271·3)	27251 (24081, 30174)	295·8 (259·4, 330·9)
**Croatia**	20371 (17992, 22895)	347·2 (307·5, 388·7)	16317 (14544, 18267)	294·4 (258·6, 330·4)
**Czech Republic**	27258 (24081, 30320)	228·9 (202·3, 254·2)	34637 (30802, 38657)	250·5 (221·1, 280·4)
**Hungary**	48829 (43235, 54336)	396·9 (350·8, 440·7)	35961 (31802, 40155)	285·9 (251·2, 320·1)
**Macedonia**	4296 (3787, 4799)	205·4 (181·8, 229·1)	7239 (6380, 8147)	264·2 (232·3, 296·1)
**Montenegro**	1224 (1084, 1370)	186·7 (165·6, 209)	1839 (1627, 2058)	241·6 (213·7, 272·1)
**Poland**	105330 (93376, 117805)	247·1 (219, 276·2)	156204 (138345, 175302)	318·1 (280·7, 357)
**Romania**	96656 (86161, 108356)	374 (331·1, 419·5)	100680 (89231, 112707)	399·1 (351·3, 446·3)
**Serbia**	19112 (16900, 21479)	176·1 (155·3, 197·7)	21235 (18875, 23752)	194·4 (172·4, 218·2)
**Slovakia**	22093 (19481, 24707)	389·7 (343·8, 435·9)	29089 (25645, 32612)	416·2 (365, 467)
**Slovenia**	8575 (7578, 9673)	370·6 (327·6, 417·2)	8071 (7172, 9019)	294 (258·7, 330·6)
**Central Asia**	153460 (141332, 166459)	264 (242·8, 285·7)	326350 (297196, 357693)	348·1 (317·4, 381·8)
**Armenia**	6658 (6111, 7261)	203·7 (186·4, 221·7)	10301 (9392, 11310)	288·4 (262·6, 316·6)
**Azerbaijan**	16461 (15028, 18016)	262 (239·5, 286·3)	35209 (31777, 38806)	305·6 (276·3, 335·9)
**Georgia**	14472 (13210, 15744)	244·7 (223·2, 266·2)	11989 (10557, 13291)	275·1 (241·1, 305·1)
**Kazakhstan**	31805 (29103, 34804)	202·3 (185·2, 220·7)	65287 (58960, 71961)	339·3 (306·3, 374)
**Kyrgyzstan**	11176 (10214, 12136)	315·2 (287·8, 342·8)	22909 (20829, 25162)	379·8 (346·5, 417)
**Mongolia**	5259 (4756, 5785)	352·2 (319·7, 386·4)	12203 (10970, 13546)	354·1 (320·1, 391·4)
**Tajikistan**	9775 (8892, 10722)	259·8 (236·8, 283·8)	25048 (22488, 27645)	307·3 (277·4, 337·6)
**Turkmenistan**	9025 (8220, 9841)	333·4 (302·5, 363·4)	21681 (19619, 23744)	420·3 (381·5, 459·2)
**Uzbekistan**	48830 (44534, 53290)	319·3 (291·7, 347·6)	121724 (110599, 134818)	376·5 (342, 415·8)
**Central Latin America**	789666 (737225, 841284)	672·4 (628·7, 717·5)	1854932 (1728568, 1997641)	728·3 (679·3, 782·9)
**Colombia**	80517 (72968, 87967)	322·2 (292·3, 353·1)	226431 (204817, 250893)	426·9 (387, 472·2)
**Costa Rica**	9892 (8993, 10839)	428·1 (391·6, 469·7)	29201 (26325, 32424)	571·7 (515, 635·3)

**El Salvador**	20279 (18408, 22025)	548·5 (496·9, 595·8)	41278 (37446, 45642)	711·7 (645·2, 786)
**Guatemala**	37801 (34673, 40982)	722·6 (663·4, 783)	118202 (106750, 129958)	877·6 (793·2, 962·3)
**Honduras**	21047 (19198, 23031)	730·8 (665·5, 802·2)	63542 (57419, 70567)	857 (773·3, 949·6)
**Mexico**	541258 (507803, 575506)	901·3 (847·5, 958·1)	1149308 (1074480, 1228749)	895·4 (838, 955·7)
**Nicaragua**	11819 (10736, 12874)	518·8 (472·4, 567·3)	36538 (33173, 40303)	652·8 (593·1, 718·7)
**Panama**	7388 (6756, 8068)	391·3 (358·1, 426·7)	20888 (18895, 23014)	518·9 (471·5, 571·5)
**Venezuela**	59665 (54075, 65247)	427·1 (388·2, 466·8)	169545 (153712, 187582)	529·4 (480·3, 584·7)
**Andean Latin America**	56022 (49668, 62540)	201·7 (178·8, 225·9)	159314 (140354, 179911)	271·3 (239·5, 306·6)
**Bolivia**	8931 (7881, 10098)	201·4 (177, 228·5)	29723 (25964, 33740)	291·5 (254·8, 330·8)
**Ecuador**	15012 (13275, 16707)	205·3 (181·8, 228·1)	46719 (41110, 52803)	292·2 (257·5, 329·4)
**Peru**	32079 (28289, 35965)	200·1 (176·1, 225·5)	82872 (72625, 94089)	254·8 (224, 289)
**Caribbean**	78788 (73355, 84634)	264·3 (245·8, 283·4)	161605 (148237, 175739)	323·7 (297·2, 351·9)
**Antigua and Barbuda**	132 (122, 143)	250·7 (231·6, 271·2)	308 (283, 337)	291·2 (267·4, 316·8)
**The Bahamas**	596 (547, 648)	278·8 (257·3, 302·3)	1339 (1230, 1468)	312 (287·2, 340·9)
**Barbados**	548 (505, 592)	215·8 (198·8, 233·3)	1034 (945, 1129)	270·4 (246·6, 295·4)
**Belize**	314 (290, 339)	269·9 (249·6, 292·4)	1216 (1116, 1322)	348·3 (319·6, 378·1)
**Bermuda**	145 (132, 159)	209·9 (191·4, 229·6)	228 (209, 250)	247·8 (226·5, 271)
**Cuba**	22442 (20687, 24184)	207·5 (191·1, 223·4)	45607 (41801, 49573)	300 (276·1, 325·8)
**Dominica**	134 (124, 145)	216·2 (199·3, 233·1)	224 (206, 243)	283·7 (260·3, 308·3)
**Dominican Republic**	16488 (15138, 17858)	322·4 (297·2, 350·8)	35703 (32286, 39048)	345·8 (313·6, 378·6)
**Grenada**	174 (161, 187)	266·9 (246, 287·6)	360 (331, 390)	296·1 (271·8, 321·5)
**Guyana**	2008 (1843, 2195)	358·3 (327·6, 389)	2800 (2560, 3054)	384·1 (352·1, 418·4)
**Haiti**	11531 (10585, 12595)	256·7 (235·5, 280)	34462 (31111, 38016)	348·8 (315·8, 384)
**Jamaica**	3537 (3281, 3808)	192·3 (177·9, 206·8)	7900 (7259, 8632)	267·8 (245·7, 292·5)
**Puerto Rico**	13780 (12730, 14910)	381 (352, 412·1)	16162 (14890, 17453)	342·1 (313·3, 370·2)

**Saint Lucia**	272 (252, 294)	277·7 (255·7, 301·3)	624 (570, 679)	299·1 (273·8, 325·4)
**Saint Vincent and the Grenadines**	177 (163, 191)	225·1 (206·6, 244·4)	353 (323, 386)	275·2 (251·6, 300·1)
**Suriname**	961 (885, 1038)	305·9 (280·9, 330·9)	2192 (2002, 2378)	352·4 (323·4, 382·8)
**Trinidad and Tobago**	2558 (2362, 2766)	248·3 (229·9, 268·5)	4830 (4429, 5274)	285 (261·2, 311·4)
**Virgin Islands**	294 (267, 321)	272·8 (249·6, 296·9)	453 (416, 495)	332·5 (304·7, 363·4)
**Tropical Latin America**	409108 (383162, 435223)	325·7 (306·1, 345·2)	738733 (688733, 791599)	299·1 (279·1, 320·1)
**Brazil**	402856 (377481, 428635)	328·4 (308·5, 348)	721144 (672385, 772639)	299·7 (279·6, 320·4)
**Paraguay**	6251 (5606, 6922)	214·9 (193·4, 238)	17589 (15777, 19672)	278·1 (249·7, 311·2)
**Southern Latin America**	169768 (158544, 180627)	356·6 (333, 379·5)	312995 (290033, 335486)	426·3 (395·2, 457·5)
**Argentina**	98453 (91932, 104942)	304·6 (283·8, 324·8)	190407 (175337, 205975)	396·5 (365, 429·2)
**Chile**	61687 (57366, 65977)	518·1 (482·3, 554·3)	107796 (99685, 115851)	502·4 (464·9, 539·3)
**Uruguay**	9622 (8960, 10228)	289 (268·8, 307·8)	14778 (13652, 15915)	372·6 (344·5, 401·5)
**East Asia**	3780313 (3465148, 4111873)	324·9 (298, 352·5)	7301772 (6647724, 7955290)	370·3 (338·2, 403·1)
**China**	3524343 (3231731, 3835862)	318·9 (292·7, 345·7)	6770357 (6156257, 7382194)	360·9 (329·2, 393·4)
**North Korea**	63627 (57836, 69702)	316·8 (288·7, 346·5)	154613 (139986, 169613)	489·5 (445·1, 537·1)
**Taiwan (Province of China)**	129363 (113853, 146107)	657·4 (578·9, 741·4)	259174 (227018, 291580)	786 (691·2, 883·8)
**Southeast Asia**	610254 (558763, 663530)	166·2 (151·8, 180·5)	1328453 (1200835, 1459855)	187·3 (170, 205·6)
**Cambodia**	10638 (9713, 11625)	157·9 (143·8, 173·2)	31109 (28210, 34360)	211·2 (191·5, 232·6)
**Indonesia**	360937 (331086, 391177)	244·8 (224, 264·9)	579392 (523161, 633852)	211·6 (192·8, 231·2)
**Laos**	3426 (3113, 3786)	118·5 (107·1, 130·8)	10217 (9234, 11346)	167·1 (151, 184·5)
**Malaysia**	8248 (6769, 9857)	59·3 (48·8, 70·3)	29291 (23960, 35195)	93·8 (77·1, 112·6)
**Maldives**	122 (111, 133)	86·9 (79·5, 94·6)	629 (564, 699)	128·5 (117, 141·6)
**Mauritius**	1364 (1245, 1486)	136·9 (125·3, 148·7)	2157 (1959, 2371)	135 (123, 148·4)
**Myanmar**	49956 (45300, 54815)	158·2 (142·7, 174·5)	107965 (97060, 119514)	196·5 (177·1, 217·2)
**Philippines**	24767 (22731, 27068)	52·1 (47·8, 56·7)	91705 (81892, 101106)	96 (85·7, 105·7)

**Sri Lanka**	18309 (16766, 19997)	121·3 (111·2, 132·1)	38111 (34585, 41905)	155·1 (141·3, 170·1)
**Seychelles**	67 (61, 73)	112·1 (102·2, 122·3)	202 (181, 224)	165·8 (150·2, 182·9)
**Thailand**	53563 (44429, 64024)	104·5 (87, 124)	132078 (108025, 159739)	140·4 (115·6, 168·8)
**East Timor**	597 (543, 661)	109 (99, 120·2)	1650 (1501, 1825)	174·2 (157·6, 193·4)
**Vietnam**	77449 (71068, 84628)	156·8 (143·2, 171·1)	302201 (272217, 334897)	278·1 (251·3, 306·5)
**Oceania**	7825 (7215, 8458)	158·3 (146·4, 170·4)	21985 (20054, 23934)	202·3 (186, 220·2)
**American Samoa**	52 (48, 57)	137·3 (126·8, 149·6)	86 (78, 94)	162·5 (147·5, 178·5)
**Federated States of Micronesia**	128 (117, 140)	177·6 (162·6, 192·9)	198 (181, 217)	205·6 (188·8, 224·8)
**Fiji**	697 (640, 755)	111·2 (102·8, 120)	1517 (1385, 1651)	165·3 (151·4, 179·3)
**Guam**	234 (215, 253)	187·6 (173·4, 202·3)	357 (327, 390)	200·4 (183·5, 218·3)
**Kiribati**	99 (91, 107)	169·1 (155·2, 183·7)	218 (198, 239)	211·7 (192·8, 232)
**Marshall Islands**	52 (48, 57)	180·5 (165·6, 195·8)	110 (100, 120)	211·2 (193·5, 231)
**Northern Mariana Islands**	79 (72, 86)	183·5 (168·7, 198·3)	110 (100, 120)	198·5 (181·6, 215·4)
**Papua New Guinea**	5055 (4632, 5499)	163·1 (150·1, 176·3)	16042 (14601, 17523)	205·2 (188·3, 224)
**Samoa**	195 (179, 212)	163·2 (150·1, 177·1)	332 (301, 363)	197·3 (180, 216·4)
**Solomon Islands**	369 (339, 400)	163 (149·7, 176·4)	1056 (961, 1154)	203·7 (186·4, 222·5)
**Tonga**	128 (118, 139)	178·3 (164, 192·5)	204 (186, 223)	222·1 (203, 242·7)
**Vanuatu**	221 (202, 242)	204·6 (187·3, 224)	543 (493, 596)	231 (209·6, 253·3)
**North Africa and Middle East**	1519854 (1390124, 1658846)	618·6 (566·7, 673)	3665267 (3354878, 4004934)	643·2 (591·9, 700·7)
**Afghanistan**	47706 (44063, 51514)	633·6 (585·8, 684·3)	152340 (139498, 165457)	746·4 (688·6, 806·4)
**Algeria**	96666 (89724, 103911)	555·6 (516·1, 596·7)	259606 (240022, 282026)	637·5 (591, 690·2)
**Bahrain**	2513 (2315, 2717)	596 (554·4, 635·7)	11055 (10151, 12004)	612·7 (567·2, 662)
**Egypt**	547770 (474960, 617451)	1323·8 (1154·8, 1490·8)	930947 (816457, 1056716)	1102·4 (971·9, 1245·7)
**Iran**	191322 (176993, 207342)	471·3 (438, 506·8)	501829 (462874, 542767)	572·1 (528·9, 617)
**Iraq**	56485 (52367, 60876)	490·4 (455·8, 525)	172800 (159886, 186356)	496·1 (460·3, 531·2)

**Jordan**	14511 (13437, 15598)	601·6 (560, 645)	65881 (60759, 71290)	713·7 (662·6, 771·2)
**Kuwait**	6215 (5721, 6701)	408·2 (380·3, 435·5)	24904 (22840, 27299)	508·4 (470·2, 551·7)
**Lebanon**	15918 (14819, 17071)	528·4 (492·9, 565·5)	48813 (45269, 52795)	629·2 (583·9, 680·4)
**Libya**	15697 (14504, 16882)	553·8 (514, 594·6)	44995 (41475, 48998)	653·9 (603·2, 709·3)
**Morocco**	106177 (98438, 113787)	551·6 (512·2, 591·1)	235977 (218057, 255191)	648 (599·2, 701·4)
**Palestine**	6610 (6140, 7113)	529·4 (493·2, 568·7)	23236 (21490, 25118)	616·9 (572·9, 663·6)
**Oman**	7693 (7120, 8336)	563·2 (524, 604·2)	29171 (26702, 31732)	624·7 (578·2, 671·1)
**Qatar**	2386 (2181, 2601)	585·5 (541·5, 630·2)	20068 (18286, 22045)	631·2 (583·1, 683)
**Saudi Arabia**	96104 (86791, 106109)	869·1 (792·6, 952·3)	335898 (300542, 373955)	931·4 (840·6, 1024·6)
**Sudan**	86336 (80053, 93427)	642·8 (596·2, 693·1)	207484 (190868, 225504)	723·5 (668·1, 783·7)
**Syria**	43278 (40126, 46463)	535 (500·1, 572)	102800 (95085, 110888)	634·1 (587·5, 683·8)
**Tunisia**	28482 (22779, 34353)	434·3 (345·8, 522·5)	65464 (52610, 79022)	506·9 (409·8, 608·5)
**Turkey**	87715 (74991, 101721)	187·6 (159·8, 217·3)	190389 (162429, 220816)	212·4 (182·1, 246)
**United Arab Emirates**	9135 (8388, 9949)	563·4 (522·5, 607·8)	87183 (79087, 96070)	662 (610·6, 719·6)
**Yemen**	50154 (46470, 54119)	630·7 (587·1, 679·7)	151004 (138481, 164319)	708·8 (653, 766·9)
**South Asia**	944643 (863189, 1032373)	105·7 (96·4, 115·7)	2020503 (1830433, 2224217)	117·9 (106·6, 129·6)
**Bangladesh**	114101 (104658, 124337)	147·5 (135·6, 159·8)	205553 (188492, 225493)	135·9 (124·6, 148·9)
**Bhutan**	591 (542, 641)	146·6 (134·9, 159·2)	1314 (1194, 1444)	139·2 (126·8, 152·3)
**India**	609804 (555839, 665766)	84·1 (76·7, 91·8)	1302550 (1177682, 1438571)	96·1 (86·8, 106)
**Nepal**	9863 (7975, 11783)	67·5 (54·9, 80·4)	17519 (14278, 21132)	64·8 (53, 78·5)
**Pakistan**	210284 (186910, 235947)	274·4 (242·8, 307)	493568 (438086, 556780)	280·1 (249·5, 314·4)
**Southern sub-Saharan Africa**	112003 (101927, 122690)	292·2 (266·7, 318)	170604 (154621, 187816)	238·5 (216·7, 261·9)
**Botswana**	2190 (1934, 2486)	262 (232·1, 295·4)	5660 (4959, 6406)	271·6 (239·3, 304·5)
**Lesotho**	3470 (3053, 3923)	275·7 (242·8, 311·3)	4656 (4085, 5274)	284·7 (251·3, 320·7)
**Namibia**	2051 (1804, 2317)	217·6 (192·2, 244·3)	4796 (4203, 5443)	250·6 (219·5, 282·6)

**South Africa**	81258 (74549, 88271)	283·5 (260·8, 307·6)	117506 (107732, 128314)	216·5 (199·3, 235·9)
**Swaziland**	1451 (1269, 1646)	306·4 (270·9, 343·9)	2545 (2220, 2891)	297·3 (262·5, 334·9)
**Zimbabwe**	21583 (19096, 24279)	350·9 (311·6, 392·4)	35442 (30857, 40147)	332·3 (291·7, 375·3)
**Western sub-Saharan Africa**	305877 (264715, 350052)	245·8 (213, 280·8)	670924 (573171, 779012)	236·5 (203·6, 272·7)
**Benin**	6748 (5829, 7787)	240·2 (207·4, 274·2)	17191 (14697, 19908)	233·4 (200·6, 270·3)
**Burkina Faso**	14912 (12931, 17143)	257 (222·4, 295·1)	36715 (31372, 42549)	271·7 (232·9, 313·1)
**Cameroon**	16095 (13870, 18564)	246 (213, 282·3)	43112 (36858, 50398)	233·8 (201·9, 271·4)
**Cape Verde**	407 (357, 461)	185·4 (161·4, 211·7)	1101 (951, 1265)	215·9 (187·6, 245·6)
**Chad**	8793 (7633, 10035)	241 (209·4, 274·7)	20270 (17255, 23408)	240·5 (205·8, 277·6)
**Côte d’Ivoire**	17453 (14862, 20318)	235·7 (202·8, 270·1)	42189 (35955, 49169)	236·4 (203·3, 272·4)
**The Gambia**	1293 (1114, 1494)	222·5 (192, 255·9)	3380 (2880, 3947)	241·1 (207·5, 278·3)
**Ghana**	22768 (19595, 26072)	233 (201·4, 265·7)	55070 (47026, 64110)	236 (203·3, 272·7)
**Guinea**	10007 (8653, 11546)	241·3 (208·8, 277·5)	18092 (15433, 21033)	238·5 (203·6, 276·6)
**Guinea-Bissau**	1516 (1301, 1748)	251·1 (216·5, 287·6)	2786 (2366, 3251)	230·6 (197·4, 266)
**Liberia**	3573 (3078, 4081)	261 (226, 298)	8047 (6807, 9426)	240·6 (205·3, 280·3)
**Mali**	16096 (13835, 18630)	287·9 (247·7, 332)	34220 (29387, 39760)	283·5 (243·1, 328·1)
**Mauritania**	3003 (2599, 3441)	224·6 (194·9, 256·7)	6007 (5176, 6990)	221·8 (190·5, 256·4)
**Niger**	11598 (9888, 13427)	250·4 (215·5, 287·6)	31309 (26848, 36352)	270·9 (231·7, 313·5)
**Nigeria**	149392 (128326, 172299)	246·1 (211·4, 282)	300875 (254756, 349573)	225·8 (194·4, 261·2)
**São Tomé and Príncipe**	243 (211, 277)	326·4 (281·2, 374·8)	476 (406, 552)	310·2 (266·8, 359·1)
**Senegal**	9326 (8103, 10744)	204·3 (177·5, 234·2)	23713 (20224, 27530)	233·8 (200·1, 270·5)
**Sierra Leone**	7462 (6394, 8593)	292·4 (251·7, 335·7)	13596 (11576, 15927)	256·3 (220·7, 296·6)
**Togo**	5184 (4458, 5985)	246·6 (212·9, 282·5)	12768 (10834, 14934)	229 (196, 265·9)
**Eastern sub-Saharan Africa**	678608 (611643, 748680)	588·7 (530·8, 646)	1515372 (1358843, 1682760)	595·5 (533·5, 658·5)
**Burundi**	22371 (19900, 24947)	654·1 (582·8, 728)	44848 (39888, 50558)	633·2 (562·1, 707·9)

**Comoros**	1536 (1371, 1716)	531·8 (473·9, 591·1)	3719 (3311, 4166)	617 (549·4, 687·6)
**Djibouti**	1528 (1365, 1708)	505·3 (452·5, 560·8)	5550 (4906, 6224)	571·3 (509·6, 637·1)
**Eritrea**	10396 (9214, 11656)	603·6 (537·8, 671·9)	27046 (24007, 30281)	654·9 (585·2, 731·3)
**Ethiopia**	202042 (183729, 221001)	650·7 (593·1, 711·4)	406739 (368684, 447358)	628·7 (572·7, 687·7)
**Kenya**	50318 (43471, 57774)	396·9 (342·1, 452·1)	134478 (115392, 155977)	398·7 (343·7, 457·8)
**Madagascar**	42515 (38175, 47216)	567 (508·7, 626·7)	105071 (94025, 117697)	597·2 (536·6, 665·1)
**Malawi**	40118 (35905, 44723)	672·9 (600·7, 746·3)	76975 (68433, 86380)	689·6 (614·1, 769·1)
**Mozambique**	44502 (39713, 49527)	479·2 (426·8, 529·4)	108996 (96744, 122418)	593·2 (527·4, 663·4)
**Rwanda**	46199 (39961, 52616)	1038·9 (909·2, 1164·9)	78533 (67645, 89758)	876·1 (766·3, 983·4)
**Somalia**	25962 (23133, 29227)	558·8 (499, 622·6)	67365 (59572, 75843)	631·8 (562·8, 706·4)
**South Sudan**	20944 (18630, 23366)	566·2 (502·5, 630·4)	39169 (34587, 44287)	617 (545, 690·5)
**Tanzania**	84315 (75513, 93117)	537·8 (481·7, 590·3)	209975 (186385, 236195)	583 (520, 651·8)
**Uganda**	54425 (48429, 60481)	553·6 (493·3, 614·1)	135244 (120539, 151761)	597 (531·3, 664·7)
**Zambia**	31093 (27811, 34451)	685·3 (611·1, 756·5)	70713 (62673, 79156)	633·6 (563·3, 703·8)
**Central sub-Saharan Africa**	155482 (137587, 175615)	443·8 (393·9, 498)	405425 (355265, 457994)	487 (428·8, 548·5)
**Angola**	30948 (27161, 35028)	469·4 (413·4, 529·5)	88722 (77585, 100859)	489·3 (429·5, 551)
**Central African Republic**	7384 (6471, 8423)	406·3 (358·7, 458·9)	15551 (13440, 17676)	448·5 (389·4, 508·1)
**Congo**	6672 (5892, 7506)	429·8 (378·1, 481·2)	17815 (15565, 20209)	438·8 (385·7, 495·5)
**DR Congo**	106076 (93668, 119687)	440·1 (389·3, 492·8)	273395 (240145, 309291)	494·4 (435·4, 555·8)
**Equatorial Guinea**	1111 (974, 1261)	402·1 (354·3, 454·3)	3535 (3068, 4017)	413·7 (362·9, 466·1)
**Gabon**	3291 (2908, 3699)	476·7 (421·2, 533·9)	6406 (5589, 7243)	443·7 (391·1, 500·8)

Appendix Table 23: Number of prevalent cases and age-standardised prevalence rates of compensated cirrhosis due to alcohol-related liver disease per 100 000 in 1990 and 2017 for both sexes by location

	1990		2017	
	**Counts (95% UI)**	**Rate (95% UI)**	**Counts (95% UI)**	**Rate (95% UI)**
**Global**	13488036 (12642698, 14408446)	290 (271·9, 309·9)	23590407 (21884257, 25482908)	288·1 (267·5, 311·3)
**High-income North America**	305887 (286978, 326982)	95·2 (89·2, 102·1)	494656 (458731, 532120)	97·8 (90·6, 105)
**Canada**	28087 (25627, 30729)	88·4 (80·6, 96·8)	48909 (43807, 54475)	91·6 (82·1, 101·6)
**Greenland**	37 (33, 41)	69·9 (63·1, 76·9)	61 (54, 68)	83·5 (74·8, 93)
**USA**	277756 (260831, 296482)	96 (90·1, 102·8)	445677 (413789, 478503)	98·5 (91·5, 105·6)
**Australasia**	27325 (24682, 29935)	122·3 (110·4, 134·2)	45771 (41276, 50431)	127·1 (114·4, 140·6)
**Australia**	22529 (20277, 24875)	120·6 (108·3, 133·3)	38384 (34420, 42641)	126·3 (113·4, 140·3)
**New Zealand**	4797 (4416, 5194)	130·8 (120·5, 141·8)	7387 (6748, 8051)	131·5 (120·3, 143·7)
**High-income Asia-Pacific**	1112974 (1019399, 1210847)	545·2 (499·2, 593·2)	1107930 (1013299, 1210597)	422·1 (384·4, 461·4)
**Brunei**	705 (632, 784)	355·9 (321·5, 391·6)	1928 (1716, 2163)	408·8 (368, 454·9)
**Japan**	503246 (469655, 538760)	313·7 (292·7, 336·1)	514272 (476081, 552637)	277·6 (257·6, 299·6)
**Singapore**	7501 (6259, 8935)	228 (192·3, 270·7)	17135 (14343, 20397)	236·9 (198·6, 280·9)
**South Korea**	601522 (531038, 670926)	1412·3 (1246·9, 1571)	574595 (511411, 642117)	758·4 (676·7, 848·4)
**Western Europe**	2853257 (2688362, 3019374)	617·9 (581·8, 655·8)	3344353 (3108094, 3586975)	582·5 (542·8, 625·4)
**Andorra**	365 (338, 394)	563·7 (522·9, 609·1)	706 (649, 769)	605·7 (557·1, 659·1)
**Austria**	95802 (89504, 105027)	1037·6 (969·4, 1135·5)	108326 (101154, 115910)	910·4 (851·8, 973·6)
**Belgium**	69226 (62350, 76331)	567·2 (507·5, 627·6)	90535 (80616, 100604)	613·8 (544·6, 686·5)
**Cyprus**	3031 (2813, 3268)	369·9 (343·5, 398·7)	6083 (5615, 6610)	370 (342·1, 402·4)
**Denmark**	29894 (28046, 31724)	477·1 (447·5, 506·3)	51073 (47467, 55096)	696·2 (649·6, 750·9)
**Finland**	24591 (22997, 26229)	396·5 (370·1, 422·9)	44655 (41356, 48161)	633·7 (588·5, 682·5)
**France**	532149 (501218, 564827)	795·3 (749·4, 843·9)	504574 (468112, 543703)	611·3 (569·3, 658·6)

**Germany**	731460 (693139, 774275)	728·1 (690·3, 771·1)	895658 (830808, 961933)	787·9 (731·8, 846·5)
**Greece**	70239 (65690, 74572)	557·4 (521·1, 591·5)	69701 (64115, 74992)	487·5 (449·6, 525·6)
**Iceland**	525 (458, 597)	200·1 (174·7, 227·8)	1404 (1225, 1592)	345·5 (299·9, 393·8)
**Ireland**	11039 (10218, 11825)	305·3 (283, 327·6)	30048 (27675, 32747)	498·4 (459·2, 543·8)
**Israel**	16148 (15026, 17288)	348·9 (325·2, 373·8)	38358 (35308, 41729)	409·9 (376·6, 446·3)
**Italy**	330369 (294249, 368856)	466·9 (414·4, 522·9)	297200 (266263, 328914)	342·3 (305·4, 380·9)
**Luxembourg**	4089 (3837, 4350)	846·5 (793·4, 901)	7394 (6832, 8015)	938·3 (867·2, 1018·8)
**Malta**	1297 (1213, 1381)	302·9 (283·4, 322·3)	1872 (1729, 2031)	322·7 (297·6, 350)
**Netherlands**	74544 (69825, 79090)	419·1 (392·8, 444·9)	108160 (99647, 116893)	482·4 (444·7, 520·9)
**Norway**	23184 (21857, 24546)	466·5 (440·1, 494·3)	35300 (33030, 37783)	529·5 (496·1, 566·3)
**Portugal**	96550 (89356, 104009)	832·4 (769·8, 896·4)	66145 (60310, 72516)	452·4 (414·3, 496·1)
**Spain**	389850 (353760, 424089)	864·4 (781·7, 942·9)	409390 (366627, 453230)	633·5 (567·4, 703·8)
**Sweden**	47196 (43987, 50531)	442 (411·4, 475·8)	79724 (73632, 86446)	615·7 (567, 669)
**Switzerland**	37349 (35085, 39627)	433·3 (406·4, 459·4)	51180 (47454, 55288)	440·7 (409, 474·5)
**United Kingdom**	261611 (246553, 278250)	385 (363·2, 409·3)	443403 (414844, 473635)	530·5 (497·1, 566·9)
**Eastern Europe**	1091230 (1020929, 1167220)	416·3 (389·3, 446)	1793963 (1672667, 1928327)	655 (609·8, 702·6)
**Belarus**	48275 (43500, 53064)	404 (362·6, 444·8)	79466 (72163, 87417)	634·4 (575·5, 699·5)
**Estonia**	6879 (6244, 7612)	376·5 (340·7, 416·8)	10764 (9823, 11858)	629·6 (569·7, 695·3)
**Latvia**	11676 (10607, 12834)	364·7 (331·3, 403·1)	14249 (12998, 15686)	555·1 (501·6, 616·6)
**Lithuania**	17951 (16391, 19720)	424·2 (385·6, 467·2)	26249 (24008, 28818)	710·2 (645·9, 782·3)
**Moldova**	47248 (42982, 51705)	1019·7 (927·4, 1115·5)	41947 (37986, 46219)	859·7 (777·1, 947·2)
**Russia**	694567 (648566, 744136)	399·1 (372·3, 427·2)	1211199 (1127551, 1300413)	638·3 (592·1, 684·4)
**Ukraine**	264635 (247117, 282679)	425·5 (397·4, 455·2)	410089 (382340, 440706)	698·1 (649·6, 749·5)
**Central Europe**	1102807 (1035695, 1172986)	783·6 (735·1, 834)	1267846 (1181162, 1364873)	824·1 (765·3, 890·7)
**Albania**	12927 (11815, 14192)	474·9 (435·2, 518·9)	20575 (18799, 22537)	611 (556·1, 671·3)

**Bosnia and Herzegovina**	27464 (25227, 29914)	565·1 (518·6, 614·3)	27067 (24601, 29689)	588·9 (534·4, 649·3)
**Bulgaria**	73590 (68615, 79076)	672·5 (625·9, 725)	75035 (69366, 81287)	771·1 (710·4, 836·5)
**Croatia**	56060 (51950, 60247)	935·6 (865·5, 1006·9)	44239 (41060, 47853)	757·7 (698·3, 821)
**Czech Republic**	78887 (74156, 84439)	650·2 (609·4, 695·8)	102159 (94827, 109974)	704·5 (654·4, 762·5)
**Hungary**	148119 (139088, 157664)	1180·8 (1104·2, 1259·8)	102121 (94528, 110412)	775·3 (716, 843·3)
**Macedonia**	9389 (8611, 10197)	449 (412·8, 487·5)	15560 (14194, 17038)	548·2 (499, 601·6)
**Montenegro**	2841 (2612, 3085)	431·3 (397·1, 468·5)	4490 (4103, 4890)	566·9 (516·4, 617·9)
**Poland**	288296 (268047, 308842)	667·2 (621·3, 715·5)	447464 (413296, 485447)	870·9 (801·8, 946·4)
**Romania**	272663 (255941, 290816)	1038·5 (970·5, 1111·6)	272835 (252859, 293066)	1039·3 (959·6, 1116·8)
**Serbia**	43581 (40112, 47391)	393·3 (360·5, 429)	52042 (47957, 56655)	455·4 (417·6, 496·8)
**Slovakia**	65009 (60407, 69884)	1135·8 (1055·6, 1220·2)	82958 (76720, 89688)	1141·7 (1054·9, 1238·6)
**Slovenia**	23982 (22379, 25640)	1021·8 (953·1, 1095)	21300 (19629, 23058)	738·7 (681·7, 799·9)
**Central Asia**	216006 (202353, 230832)	380·6 (356·5, 405·4)	464824 (429064, 501955)	496·8 (459·3, 535·9)
**Armenia**	8975 (8295, 9644)	275·9 (255·8, 296·2)	14023 (12950, 15218)	381·9 (352·3, 416)
**Azerbaijan**	22659 (21020, 24338)	367·4 (341·3, 395·1)	48650 (44574, 53344)	418·9 (384·4, 457·6)
**Georgia**	21842 (20339, 23477)	363·8 (337·9, 391·6)	18014 (16143, 19705)	399·6 (356·9, 439·1)
**Kazakhstan**	48807 (45389, 52346)	314·4 (292·5, 336·1)	102604 (94361, 111313)	529·4 (487·9, 573·7)
**Kyrgyzstan**	15409 (14306, 16585)	444·9 (413·3, 477·3)	30095 (27729, 32775)	504·7 (465·6, 546·7)
**Mongolia**	6669 (6150, 7245)	469·2 (432·5, 509·9)	16876 (15414, 18401)	493·2 (452·7, 536·1)
**Tajikistan**	13621 (12600, 14642)	381 (354·7, 408)	33534 (30575, 36550)	423·2 (388·5, 460·7)
**Turkmenistan**	12028 (11175, 12989)	463·1 (429·9, 497·5)	30009 (27512, 32434)	583·4 (535, 630·1)
**Uzbekistan**	65994 (61677, 71061)	449·8 (420·2, 482·7)	171019 (156666, 185744)	534·7 (492, 579·7)
**Central Latin America**	872423 (819465, 924014)	782·4 (738·5, 828)	2191522 (2049327, 2344069)	865·3 (810·2, 925·4)
**Colombia**	107606 (98806, 116975)	456·2 (420·4, 494·5)	319030 (293689, 348027)	598 (550·3, 652·4)
**Costa Rica**	13419 (12328, 14506)	612·7 (564·1, 663·1)	41055 (37697, 44953)	802·2 (735·8, 879·1)

**El Salvador**	22372 (20503, 24394)	632·7 (580·4, 689·2)	49753 (45331, 54650)	865·6 (788·2, 950·7)
**Guatemala**	36470 (33508, 39369)	729·3 (670·8, 786·4)	115725 (106096, 125964)	892·6 (816·7, 971·4)
**Honduras**	19706 (17948, 21515)	722·6 (660·1, 789·2)	64031 (58157, 70638)	890·6 (808, 981·8)
**Mexico**	577358 (545531, 609107)	1010·5 (957·4, 1067·4)	1324926 (1246092, 1406978)	1036·6 (976·4, 1099·6)
**Nicaragua**	12538 (11508, 13629)	588·2 (542·1, 639·1)	40661 (37053, 44616)	746·5 (681, 818·7)
**Panama**	8306 (7609, 9012)	459·4 (421·7, 497·4)	25713 (23594, 28067)	636·9 (583·1, 694·9)
**Venezuela**	74649 (69204, 80545)	565·5 (524·4, 607·6)	210628 (192640, 231432)	661·6 (605·8, 725·9)
**Andean Latin America**	97495 (88621, 106335)	371·6 (337·8, 406·6)	268367 (242260, 296021)	462·5 (417·2, 509·6)
**Bolivia**	13858 (12422, 15294)	329·6 (295·5, 364·5)	45857 (40811, 51084)	460·4 (410·7, 512·6)
**Ecuador**	19171 (17223, 21097)	278·5 (251·7, 306·4)	64221 (57280, 71529)	406·3 (362·1, 451·7)
**Peru**	64466 (58595, 70404)	425 (386, 465·7)	158289 (142954, 174856)	490·7 (443·6, 541·3)
**Caribbean**	88095 (81995, 94280)	303 (282·2, 325)	185272 (170219, 201590)	368·5 (338·7, 401)
**Antigua and Barbuda**	140 (129, 152)	275·1 (252·7, 299·2)	350 (320, 381)	324·9 (297·7, 352·9)
**The Bahamas**	783 (727, 847)	382·2 (354·2, 412·3)	1630 (1490, 1770)	375·1 (343·2, 407·3)
**Barbados**	626 (579, 677)	251·1 (232·5, 271·5)	1206 (1103, 1312)	303·5 (278·1, 330·6)
**Belize**	344 (318, 370)	312·9 (289·6, 337·1)	1386 (1273, 1503)	407·4 (374·9, 441·6)
**Bermuda**	218 (198, 240)	317·1 (287·7, 347·6)	314 (284, 345)	323·3 (293·5, 354·6)
**Cuba**	23413 (21592, 25331)	219·6 (202·3, 237·1)	52244 (47898, 57226)	330·2 (303, 361·4)
**Dominica**	163 (152, 175)	267·9 (248·8, 288·1)	272 (250, 296)	338·3 (310·7, 367·8)
**Dominican Republic**	19019 (17532, 20701)	391 (360·7, 425·1)	43327 (39223, 47492)	424 (384·7, 464·6)
**Grenada**	215 (198, 231)	339·1 (312·8, 366·1)	429 (395, 466)	348·1 (320·9, 377·4)
**Guyana**	2416 (2228, 2612)	452·3 (418·2, 487·1)	3198 (2932, 3481)	438·3 (402·1, 476·2)
**Haiti**	12566 (11431, 13712)	289·8 (263·4, 316·3)	36782 (33225, 40598)	385·5 (347·5, 425·4)
**Jamaica**	3574 (3316, 3849)	203 (188·2, 218·8)	8293 (7552, 9060)	282·1 (257·2, 308·4)
**Puerto Rico**	16871 (15624, 18160)	467 (432·6, 502·7)	19523 (17932, 21085)	400·8 (368·3, 432·8)

**Saint Lucia**	337 (311, 363)	357·3 (329·9, 385·4)	754 (694, 818)	355 (327·3, 385·3)
**Saint Vincent and the Grenadines**	199 (184, 216)	265·8 (245·2, 287·7)	416 (377, 456)	317·7 (288·7, 348·5)
**Suriname**	1070 (991, 1160)	349·5 (323·5, 379·3)	2606 (2399, 2842)	414 (381·3, 449·9)
**Trinidad and Tobago**	2752 (2536, 2967)	275·7 (254·3, 297·6)	5284 (4800, 5772)	305·4 (277·8, 333·8)
**Virgin Islands**	374 (344, 407)	345·9 (318·6, 374·9)	596 (543, 649)	418·3 (384·4, 455·1)
**Tropical Latin America**	256052 (240050, 273436)	211·2 (198·3, 225·7)	491990 (458036, 529199)	198·2 (184·5, 213·1)
**Brazil**	250719 (235176, 267795)	211·7 (199, 226)	477419 (444292, 513374)	197·2 (183·6, 212)
**Paraguay**	5333 (4779, 5913)	191·2 (171·7, 212·2)	14571 (12916, 16335)	234·9 (208·6, 263·2)
**Southern Latin America**	223617 (211793, 237242)	471 (445·7, 500·1)	380028 (353544, 405839)	513·3 (477·7, 548)
**Argentina**	140026 (132182, 148686)	432·5 (408·1, 459·3)	239065 (221078, 256451)	495·5 (458·7, 531·4)
**Chile**	73552 (68924, 78381)	627·7 (588·3, 669·6)	125963 (116955, 135031)	578·2 (537·3, 620)
**Uruguay**	10029 (9403, 10774)	297·9 (279·3, 319·6)	14984 (13855, 16162)	371·1 (342·6, 399·9)
**East Asia**	2916953 (2698743, 3147802)	259·3 (240·1, 279·7)	6291209 (5771875, 6864238)	306·8 (282·6, 333·3)
**China**	2757098 (2550813, 2982112)	258·4 (239, 278·5)	5942774 (5455190, 6483091)	304·7 (280·3, 330·7)
**North Korea**	41602 (36896, 46878)	207 (183·5, 232·4)	103563 (90786, 117817)	319·2 (281·4, 360·7)
**Taiwan (Province of China)**	69655 (61546, 78350)	361·1 (320, 404·4)	143524 (127310, 161525)	417·2 (370·5, 469·4)
**Southeast Asia**	199394 (178204, 223011)	57·6 (51·6, 64·4)	543840 (480431, 613912)	76·4 (67·6, 86)
**Cambodia**	4272 (3766, 4768)	68·6 (60·5, 76·3)	14925 (13109, 16948)	104·2 (91·5, 118)
**Indonesia**	81403 (73411, 90403)	58·2 (52·6, 64·5)	138839 (123934, 154455)	50·6 (45·4, 56·1)
**Laos**	1850 (1647, 2081)	67·9 (60·3, 76·4)	5536 (4840, 6246)	94·5 (82·5, 106·7)
**Malaysia**	5311 (4226, 6640)	40·8 (32·3, 50·6)	19519 (15636, 24348)	63·6 (51·2, 79·1)
**Maldives**	53 (47, 60)	41·8 (36·9, 46·8)	286 (250, 325)	61·8 (54·8, 69·6)
**Mauritius**	743 (663, 833)	77·9 (69·4, 87·2)	1210 (1075, 1358)	72·5 (64·6, 81·2)
**Myanmar**	20659 (18155, 23458)	69 (60·7, 78·5)	47667 (41594, 53719)	87 (76, 97·8)
**Philippines**	12641 (11260, 14070)	28·9 (25·8, 32)	49648 (43406, 56082)	53·2 (46·6, 59·8)

**Sri Lanka**	8235 (7326, 9237)	56·7 (50·4, 63·4)	18826 (16622, 21215)	74·8 (66·2, 84·3)
**Seychelles**	32 (28, 36)	55·9 (49·3, 63·1)	105 (92, 120)	84·2 (74·3, 95·4)
**Thailand**	28525 (22121, 36134)	58·3 (45·4, 73·4)	77741 (59894, 98691)	78·8 (61·3, 99·2)
**East Timor**	249 (219, 283)	49·4 (43·6, 55·9)	715 (633, 809)	79·5 (70·1, 90·1)
**Vietnam**	35154 (31296, 39219)	76·9 (68·2, 86·3)	168109 (148141, 189517)	153·6 (135·7, 172·3)
**Oceania**	4180 (3796, 4575)	91·8 (83·6, 100·2)	11840 (10644, 13078)	114·9 (103·7, 126·4)
**American Samoa**	27 (25, 30)	78·9 (71·9, 87·3)	48 (43, 54)	91 (81·6, 101·9)
**Federated States of Micronesia**	65 (59, 73)	100·5 (90·5, 110·8)	106 (94, 118)	112·3 (100·6, 124·3)
**Fiji**	340 (310, 373)	58·7 (53·7, 64·1)	815 (735, 904)	88·8 (80·2, 98·1)
**Guam**	141 (128, 153)	119 (108·1, 129·6)	230 (206, 254)	125·4 (112·9, 138·5)
**Kiribati**	49 (45, 55)	91·2 (82·5, 100·4)	112 (101, 125)	113·8 (101·8, 126·8)
**Marshall Islands**	27 (24, 29)	104·3 (94·1, 114·9)	61 (54, 67)	121·1 (108·6, 134·2)
**Northern Mariana Islands**	44 (40, 49)	110·8 (100·3, 120·9)	69 (61, 77)	115·4 (104·3, 127·1)
**Papua New Guinea**	2749 (2487, 3023)	96·2 (87·1, 105·5)	8630 (7765, 9572)	117·6 (106·1, 130·1)
**Samoa**	101 (91, 111)	93·1 (84·5, 102·1)	184 (165, 206)	112·8 (100·9, 125·4)
**Solomon Islands**	187 (169, 206)	91·1 (82·5, 100·1)	554 (498, 613)	113·6 (102·4, 125·3)
**Tonga**	64 (58, 71)	96·4 (87·5, 105·9)	110 (99, 122)	122·4 (110·1, 135·4)
**Vanuatu**	110 (99, 121)	110·9 (99·7, 123)	269 (242, 300)	121·5 (108·8, 134·8)
**North Africa and Middle East**	134954 (120225, 150910)	59·5 (53, 66·2)	342541 (303368, 384417)	62·1 (55·4, 69·5)
**Afghanistan**	5055 (4506, 5625)	67·6 (60·5, 75)	14425 (12736, 16256)	79·3 (70·4, 88·7)
**Algeria**	9211 (8293, 10186)	58·8 (52·7, 64·9)	26970 (24026, 30222)	67·6 (60·4, 75·6)
**Bahrain**	283 (253, 318)	75·1 (67·5, 83·4)	1339 (1181, 1509)	71·9 (64·4, 80)
**Egypt**	38126 (32138, 44674)	96·6 (81·9, 112·3)	64374 (54512, 75781)	78·7 (66·8, 92·3)
**Iran**	11135 (10125, 12248)	30·8 (28, 33·8)	32421 (29320, 35653)	36·9 (33·4, 40·5)
**Iraq**	5478 (4897, 6059)	53 (47·6, 58·5)	16674 (14810, 18555)	51·8 (46·4, 57·3)

**Jordan**	1295 (1164, 1435)	61·5 (55·5, 68·3)	6379 (5626, 7079)	73·3 (65, 81·2)
**Kuwait**	597 (535, 669)	44·2 (39·9, 48·8)	2559 (2268, 2887)	53·9 (48·2, 60·1)
**Lebanon**	1721 (1548, 1901)	61·2 (55·4, 67·5)	5151 (4581, 5722)	69·8 (62·3, 77·5)
**Libya**	1528 (1375, 1679)	59·9 (54, 66)	4641 (4123, 5216)	69·2 (61·8, 77·1)
**Morocco**	10055 (9014, 11125)	56·6 (50·9, 62·7)	23738 (21113, 26602)	65·4 (58·5, 73·3)
**Palestine**	594 (534, 659)	54·1 (48·8, 59·7)	2205 (1952, 2462)	63·6 (56·8, 70·6)
**Oman**	759 (679, 844)	62·1 (56, 69·1)	3066 (2694, 3437)	71·3 (63·8, 79·1)
**Qatar**	265 (234, 298)	71·4 (63·8, 79·7)	2270 (1978, 2556)	76·7 (68·1, 85·8)
**Saudi Arabia**	6284 (5344, 7331)	63 (54·2, 72·9)	22906 (19347, 27027)	65·8 (56·4, 76·5)
**Sudan**	9124 (8171, 10200)	75 (67·2, 83·7)	22001 (19453, 24683)	84·7 (75·3, 95)
**Syria**	3902 (3492, 4297)	54·6 (49, 60·2)	10542 (9440, 11730)	65·6 (58·8, 72·7)
**Tunisia**	5519 (4613, 6582)	88·7 (74, 104·9)	13784 (11513, 16363)	105·3 (88·4, 124·3)
**Turkey**	17869 (15547, 20378)	40·3 (35·1, 46)	41426 (36027, 47418)	45·5 (39·7, 52)
**United Arab Emirates**	1144 (988, 1315)	78·5 (69·5, 88·1)	10966 (9442, 12590)	83·1 (73·5, 93·6)
**Yemen**	4924 (4404, 5463)	69·4 (62·2, 77·4)	14385 (12739, 16101)	75·1 (66·6, 83·8)
**South Asia**	1135950 (1039268, 1237831)	133·8 (123·2, 145·9)	2527215 (2291839, 2783916)	151·6 (137·6, 166·5)
**Bangladesh**	102137 (93229, 111875)	144·7 (132·5, 158)	197785 (179958, 216598)	134 (121·7, 146·6)
**Bhutan**	637 (579, 699)	171·3 (155·9, 187·3)	1404 (1269, 1554)	156·6 (141·4, 172·3)
**India**	967413 (884947, 1053479)	140 (128·8, 152·3)	2188017 (1987144, 2404428)	164·8 (149·8, 180·5)
**Nepal**	32899 (29287, 37189)	238·2 (212·6, 267·8)	63726 (55955, 72234)	244·7 (215·1, 276·5)
**Pakistan**	32864 (26348, 40581)	45·6 (36·6, 56·3)	76283 (61508, 94029)	45·9 (37·3, 56·3)
**Southern sub-Saharan Africa**	84448 (77863, 91869)	228 (210·8, 247·2)	126137 (115529, 138080)	179·1 (164·5, 195·7)
**Botswana**	1447 (1301, 1602)	180·4 (162·7, 199·1)	3966 (3552, 4438)	194·4 (175·6, 216·3)
**Lesotho**	2345 (2099, 2624)	191·7 (171·5, 214·2)	3212 (2861, 3608)	201·1 (180·2, 226)
**Namibia**	1294 (1143, 1456)	142·8 (126·2, 160·2)	3660 (3289, 4070)	196 (177, 217)

**South Africa**	64215 (59523, 69343)	231·1 (214·4, 249·7)	90513 (83447, 98349)	168·4 (155·6, 182·6)
**Swaziland**	958 (864, 1065)	211·4 (190·2, 234·3)	1592 (1423, 1772)	192·2 (172·7, 213·4)
**Zimbabwe**	14189 (12715, 15762)	241·6 (217, 267·7)	23195 (20630, 26057)	224·7 (200·7, 250·9)
**Western sub-Saharan Africa**	372537 (338657, 409873)	306·7 (278·6, 337·2)	811691 (729513, 903406)	292·8 (264·3, 325·1)
**Benin**	6984 (6278, 7781)	256·8 (231·1, 284·3)	18973 (16943, 21170)	265 (237·9, 294·2)
**Burkina Faso**	16937 (15410, 18535)	299·2 (272·5, 327)	44059 (39441, 49415)	334 (298·5, 372·9)
**Cameroon**	17269 (15688, 19102)	269·9 (244·3, 297·9)	49550 (44361, 55372)	275·8 (247·5, 307·2)
**Cape Verde**	419 (381, 462)	195·1 (177·1, 214·6)	1252 (1124, 1396)	247 (222·4, 274·4)
**Chad**	8570 (7779, 9425)	241 (218·2, 264·5)	21271 (19042, 23562)	261·2 (234·4, 288·8)
**Côte d'Ivoire**	18404 (16631, 20404)	256·3 (233, 282·6)	46850 (42147, 52167)	268·5 (243, 298·1)
**The Gambia**	1338 (1211, 1493)	239·2 (216·5, 264·4)	3657 (3278, 4068)	268·6 (242·7, 297·5)
**Ghana**	23548 (21269, 25982)	246·8 (223·8, 271·5)	61216 (54833, 68179)	266·6 (239·4, 295·4)
**Guinea**	9573 (8619, 10654)	235·5 (212·4, 261·2)	18182 (16273, 20380)	246·6 (220·9, 275·6)
**Guinea-Bissau**	1563 (1422, 1721)	266·6 (242·8, 291·9)	2998 (2690, 3350)	255·3 (229·6, 282·9)
**Liberia**	4201 (3792, 4677)	312·2 (283·4, 344·3)	9143 (8184, 10141)	280·1 (252·1, 308·7)
**Mali**	13579 (12282, 15033)	248·1 (224·3, 273·2)	30203 (27122, 33754)	257·7 (231·6, 287·9)
**Mauritania**	2559 (2316, 2819)	195·9 (177·9, 215·7)	5290 (4755, 5906)	199·4 (178·9, 221·4)
**Niger**	10769 (9682, 12039)	240·6 (216·2, 266·6)	30294 (26939, 34185)	271 (242·4, 303·8)
**Nigeria**	215330 (194010, 237128)	362·2 (325·6, 399·4)	418252 (372267, 468130)	321 (288·6, 357·5)
**São Tomé and Príncipe**	248 (225, 273)	341·2 (308·5, 375·9)	548 (490, 617)	363·4 (326, 405·9)
**Senegal**	7905 (7199, 8753)	178·5 (162·6, 197·3)	21023 (18893, 23561)	211·7 (190·3, 236·3)
**Sierra Leone**	8379 (7563, 9238)	336·2 (304·4, 369·6)	16303 (14570, 18224)	315·8 (284·4, 350·9)
**Togo**	4952 (4482, 5443)	244 (222·6, 268·5)	12619 (11228, 14165)	230 (205·9, 256·4)
**Eastern sub-Saharan Africa**	320054 (289232, 352996)	288 (260·8, 318·4)	709379 (636528, 792397)	289·2 (261·5, 320·7)
**Burundi**	12411 (10991, 13938)	377·5 (334·8, 420·8)	24090 (21326, 27180)	354·2 (314·7, 398·2)

**Comoros**	604 (540, 671)	217 (194·3, 241)	1462 (1304, 1644)	246·7 (220·9, 276·2)
**Djibouti**	690 (616, 770)	241·8 (217·1, 268·6)	2556 (2264, 2884)	270·2 (242·1, 302·9)
**Eritrea**	4505 (4023, 5057)	272·7 (244·8, 304·8)	11568 (10235, 12941)	289·9 (259, 322·5)
**Ethiopia**	70586 (63843, 78144)	234·1 (211·6, 259·3)	142186 (127795, 157745)	228·2 (206·1, 252·1)
**Kenya**	44390 (40002, 49504)	356·6 (321·6, 395·5)	110323 (98438, 123730)	331 (298·1, 369·9)
**Madagascar**	18914 (16959, 21021)	262·7 (236·8, 290·4)	45071 (39820, 50684)	265·5 (235·9, 296·9)
**Malawi**	16917 (15166, 18823)	295·4 (264·2, 326·9)	31846 (28426, 35558)	297·6 (265·9, 333)
**Mozambique**	21470 (19073, 24044)	240·4 (214·7, 268·5)	53490 (47329, 60048)	305·3 (272·3, 342·5)
**Rwanda**	19300 (16939, 22023)	449·6 (395·2, 511·6)	31322 (27364, 36142)	357·5 (313·4, 410·3)
**Somalia**	11366 (9985, 12865)	252·5 (225, 283·8)	28770 (25389, 32695)	281·2 (250·3, 317·1)
**South Sudan**	9711 (8614, 10883)	275·3 (245·8, 308·1)	17789 (15630, 20075)	291·2 (257·9, 327)
**Tanzania**	43024 (38817, 47788)	286 (258·1, 316·6)	102676 (91887, 114586)	295·6 (265·5, 329·1)
**Uganda**	31810 (28611, 35159)	339·5 (305·2, 374·2)	74756 (67138, 83391)	346·9 (312·7, 386·1)
**Zambia**	14194 (12693, 15764)	327·3 (293·7, 362·5)	31030 (27757, 34666)	290·6 (260·3, 322·7)
**Central sub-Saharan Africa**	72399 (65040, 80698)	213·5 (192·3, 236·8)	190034 (168739, 213483)	234·8 (209·6, 262·1)
**Angola**	14141 (12671, 15818)	221·9 (199·5, 248·7)	43431 (38475, 48882)	247·2 (220·6, 276·6)
**Central African Republic**	3629 (3240, 4057)	205·6 (184, 229)	7276 (6399, 8233)	214·9 (190·2, 242·4)
**Congo**	3354 (3018, 3734)	223 (200·6, 248·5)	8693 (7682, 9778)	217·8 (193·7, 243·8)
**DR Congo**	48928 (43738, 54514)	209·8 (188, 232·9)	125575 (111219, 141312)	233·6 (207·4, 261·4)
**Equatorial Guinea**	535 (480, 598)	198·6 (178·3, 221·9)	1848 (1625, 2081)	227 (201·2, 254·9)
**Gabon**	1811 (1621, 2029)	268·5 (240, 300·2)	3211 (2854, 3608)	226·1 (201·6, 253·4)

Appendix Table 24: Number of prevalent cases and age-standardised prevalence rates of compensated cirrhosis due to non-alcoholic steatohepatitis (NASH) per 100 000 in 1990 and 2017 for both sexes by location

	1990		2017	
	**Counts (95% UI)**	**Rate (95% UI)**	**Counts (95% UI)**	**Rate (95% UI)**
**Global**	4060384 (3697171, 4446518)	86·7 (79, 94·6)	9424419 (8570840, 10335527)	115·5 (105, 126·5)
**High-income North America**	101741 (94321, 109605)	31·5 (29·2, 34)	191460 (175784, 207365)	37·5 (34·4, 40·6)
**Canada**	7908 (7050, 8841)	24·8 (22·1, 27·8)	15338 (13270, 17652)	28·4 (24·6, 32·5)
**Greenland**	11 (9, 12)	21·1 (18·3, 23·9)	18 (15, 21)	24·9 (21·1, 28·7)
**USA**	93820 (87008, 100906)	32·3 (29·9, 34·8)	176101 (161684, 190239)	38·6 (35·4, 41·7)
**Australasia**	11804 (10503, 13221)	52·9 (47·1, 59·3)	23869 (21198, 26716)	66·5 (58·9, 74·3)
**Australia**	9631 (8453, 10906)	51·6 (45·1, 58·6)	19811 (17504, 22388)	65·4 (57·5, 73·8)
**New Zealand**	2173 (2006, 2357)	59·4 (54·8, 64·4)	4058 (3712, 4425)	72·6 (66·5, 79)
**High-income Asia-Pacific**	216093 (186309, 249877)	105·9 (91·4, 122·3)	244744 (210036, 283125)	91·2 (78, 105·3)
**Brunei**	120 (104, 139)	61·3 (54·2, 69·6)	409 (352, 477)	88·9 (77, 102·9)
**Japan**	132654 (117500, 148635)	83·1 (73·8, 93)	140447 (126629, 154911)	75 (67, 83)
**Singapore**	867 (675, 1095)	26·8 (21·3, 33·2)	2530 (1991, 3167)	35·5 (28·1, 44·2)
**South Korea**	82452 (63669, 104881)	197·4 (153·2, 249·3)	101358 (77990, 127772)	133·6 (103·7, 168·3)
**Western Europe**	436291 (379934, 503177)	94·2 (81·4, 108·8)	691199 (609830, 778069)	119·1 (104·5, 135·1)
**Andorra**	50 (43, 58)	78·5 (67·5, 90·3)	131 (112, 152)	111·5 (95·9, 129)
**Austria**	12722 (11035, 14662)	136·9 (117·8, 157·9)	19053 (16655, 21678)	158·5 (137·2, 181·6)
**Belgium**	6114 (4584, 8096)	50 (37·2, 66·6)	10381 (7811, 13360)	69·9 (51·8, 92·3)
**Cyprus**	512 (443, 587)	62·6 (54, 71·8)	1321 (1151, 1497)	80·7 (70·2, 91·6)
**Denmark**	4063 (3511, 4621)	64·5 (55·8, 73·8)	8784 (7688, 9929)	118·3 (102·6, 134·4)
**Finland**	4469 (3853, 5057)	72 (62·1, 81·7)	10399 (9191, 11763)	145·1 (127, 165·2)
**France**	63397 (53918, 72644)	94·7 (80, 108·8)	87135 (76065, 98386)	104·2 (90, 118·6)

**Germany**	101910 (88668, 115186)	100·8 (86·9, 114·3)	176045 (154927, 199675)	152·8 (132·3, 174·3)
**Greece**	11559 (10129, 13021)	91·5 (79·6, 103·9)	15647 (13710, 17727)	108·1 (94·6, 123)
**Iceland**	261 (219, 308)	99·5 (83·5, 117·9)	822 (691, 962)	202·1 (169, 238)
**Ireland**	1956 (1715, 2222)	53·8 (47, 61·5)	6268 (5429, 7127)	103·9 (89·8, 118·2)
**Israel**	4217 (3685, 4802)	91 (79·5, 103·8)	12414 (10906, 14024)	132·2 (115·8, 149·6)
**Italy**	80057 (62209, 118131)	113 (86·9, 167·5)	93241 (76780, 110762)	105·6 (86·7, 126·2)
**Luxembourg**	661 (575, 749)	136·8 (118·4, 155·2)	1341 (1158, 1539)	169·9 (147·2, 195·3)
**Malta**	264 (228, 300)	61·8 (53·6, 70·2)	489 (425, 554)	83·7 (72·4, 95·3)
**Netherlands**	13159 (11560, 14919)	73·9 (64·8, 83·9)	24322 (21286, 27458)	107·3 (93·1, 122·2)
**Norway**	4245 (3904, 4615)	84·9 (77·9, 92·3)	7725 (7067, 8342)	115 (104·7, 124·4)
**Portugal**	13490 (11657, 15551)	116·3 (99·4, 134·6)	13946 (12038, 15922)	94·5 (81·6, 108·8)
**Spain**	51243 (44093, 59727)	114·2 (97·2, 133·8)	72269 (61832, 84286)	112·7 (95·6, 131·2)
**Sweden**	9008 (8108, 9924)	84·5 (75·9, 93·6)	19593 (17657, 21637)	152·2 (137·1, 169·1)
**Switzerland**	5286 (4599, 5997)	61·2 (53, 69·5)	9645 (8393, 10957)	82·3 (71·5, 93·2)
**United Kingdom**	47228 (43154, 51418)	69·1 (62·8, 75·4)	99511 (90676, 108182)	117·8 (106·6, 128·9)
**Eastern Europe**	287402 (268311, 308469)	111·4 (104, 119·3)	522272 (486363, 563067)	194·8 (181·7, 209·5)
**Belarus**	10719 (9624, 11860)	90·7 (81·5, 100·9)	19932 (17795, 22056)	162 (144·4, 179·2)
**Estonia**	1646 (1482, 1827)	91·4 (82, 101·5)	2558 (2289, 2839)	150·8 (134·3, 167·8)
**Latvia**	2856 (2563, 3172)	90·6 (81·2, 100·3)	3822 (3432, 4223)	150·2 (134·6, 166·7)
**Lithuania**	4166 (3742, 4620)	99·4 (89·5, 110·2)	6510 (5833, 7220)	177·3 (158·1, 197·1)
**Moldova**	12003 (10783, 13268)	260·1 (233, 288·1)	12842 (11511, 14294)	265·9 (238·4, 296·4)
**Russia**	187810 (175314, 202066)	109·7 (102·4, 117·9)	361428 (336313, 389565)	194·7 (181·2, 209·7)
**Ukraine**	68204 (63452, 73343)	111·7 (103·9, 120·6)	115181 (106997, 124408)	200·3 (185·6, 215·9)
**Central Europe**	168686 (153737, 184677)	121·1 (110·1, 133·2)	228182 (206614, 249517)	151·4 (137, 166·6)
**Albania**	2678 (2389, 2995)	96·4 (86·6, 107·6)	4601 (4106, 5105)	138·4 (123, 154·6)

**Bosnia and Herzegovina**	5336 (4782, 5940)	110·4 (99, 122·8)	6700 (5974, 7448)	149 (133·2, 165·8)
**Bulgaria**	11784 (10693, 13063)	109·7 (99·5, 121·8)	13026 (11742, 14368)	137 (123·4, 152)
**Croatia**	8701 (7851, 9581)	147·2 (132·4, 162·9)	8250 (7485, 9051)	144·1 (130·5, 159·4)
**Czech Republic**	11729 (10653, 12964)	97·8 (88·7, 108·2)	16925 (15265, 18613)	119·4 (108, 132·1)
**Hungary**	22667 (20269, 25247)	182·3 (162·3, 203·3)	18457 (16680, 20358)	143 (129, 158·5)
**Macedonia**	1890 (1706, 2098)	90·7 (81·9, 100·7)	3835 (3433, 4247)	138·1 (124·2, 153·4)
**Montenegro**	539 (486, 595)	82·2 (74·2, 90·9)	892 (795, 993)	114·8 (102, 127·7)
**Poland**	42987 (38884, 47417)	100·7 (90·6, 111·2)	76933 (69116, 85114)	153·2 (137·3, 170·2)
**Romania**	39107 (35462, 42805)	149·9 (135·5, 164·7)	49646 (44619, 54763)	192·2 (172·6, 212·8)
**Serbia**	8080 (7247, 8922)	73·9 (66·3, 81·7)	10506 (9409, 11601)	93·7 (83·9, 103·9)
**Slovakia**	9597 (8684, 10619)	169 (152·7, 186·5)	14093 (12695, 15594)	198·4 (178·3, 220·1)
**Slovenia**	3593 (3265, 3948)	154·7 (140·2, 170)	4318 (3878, 4758)	152·8 (137·1, 168·6)
**Central Asia**	45065 (40927, 49694)	78·3 (71, 86·6)	111887 (99488, 124947)	120·5 (107·4, 134·4)
**Armenia**	1964 (1775, 2173)	60·4 (54·6, 66·8)	3616 (3238, 4012)	99·7 (89·3, 110·7)
**Azerbaijan**	4900 (4396, 5397)	78·7 (70·6, 86·7)	13020 (11472, 14603)	113·6 (100·6, 126·8)
**Georgia**	4485 (4053, 4976)	75 (67·8, 83·2)	4334 (3774, 4852)	96·9 (83·8, 108·5)
**Kazakhstan**	9820 (8815, 10959)	63·1 (56·6, 70·2)	22548 (20085, 25063)	117·6 (104·9, 130·4)
**Kyrgyzstan**	3181 (2863, 3531)	90·4 (81·1, 100·6)	7107 (6230, 7987)	119·6 (105·1, 134·1)
**Mongolia**	1407 (1259, 1561)	96 (85·9, 106·9)	3736 (3267, 4248)	110·9 (97·8, 125·2)
**Tajikistan**	2702 (2428, 2990)	72·7 (64·6, 80·4)	7588 (6675, 8594)	95·2 (84, 107·3)
**Turkmenistan**	2514 (2277, 2774)	94·5 (85·2, 104·2)	7472 (6633, 8351)	146·7 (130·5, 163·4)
**Uzbekistan**	14092 (12783, 15620)	93·5 (84·7, 103·4)	42466 (37609, 47425)	133·5 (118·8, 149·3)
**Central Latin America**	263944 (238106, 291706)	238 (215·3, 261·7)	818825 (738767, 903568)	324·8 (293·6, 357·6)
**Colombia**	25941 (22593, 29536)	111·2 (97·1, 126·2)	97694 (85639, 111402)	182·8 (160·3, 208·6)
**Costa Rica**	3472 (3027, 3949)	160·2 (140, 182)	13639 (11969, 15539)	267·3 (234·8, 304·4)

**El Salvador**	6982 (6122, 7915)	197·7 (173, 224·9)	19582 (17266, 22144)	340·8 (300·1, 385·5)
**Guatemala**	11865 (10305, 13567)	238·7 (209, 274)	47412 (41396, 54013)	370·8 (324·9, 420·9)
**Honduras**	6801 (5910, 7769)	250·5 (218·3, 286·5)	26844 (23292, 30623)	377·5 (328·4, 430)
**Mexico**	180526 (164105, 198565)	317·2 (289·5, 347·5)	504560 (459772, 551168)	396·9 (362·4, 432·7)
**Nicaragua**	4176 (3652, 4704)	197·1 (173·2, 221·8)	18124 (15863, 20540)	337·4 (296·3, 381)
**Panama**	2256 (1965, 2602)	125·6 (109·4, 144·7)	7944 (6782, 9154)	196·9 (168·7, 226·5)
**Venezuela**	21924 (19255, 24995)	168·2 (148·2, 189·9)	83025 (72961, 93782)	263·6 (232·5, 297·1)
**Andean Latin America**	43643 (39212, 48548)	165·5 (148·6, 184·5)	162687 (146358, 181356)	281·1 (252·7, 312·6)
**Bolivia**	6780 (6027, 7572)	161·2 (143·2, 179·9)	29191 (25939, 32890)	294·2 (262·1, 331·2)
**Ecuador**	12885 (11576, 14350)	186·3 (167·2, 207·4)	52093 (46480, 58069)	330·2 (294·8, 366·8)
**Peru**	23978 (21346, 26645)	157·3 (140·3, 175·8)	81403 (72695, 91048)	253 (226, 282)
**Caribbean**	42674 (38592, 47131)	147·8 (133·7, 163·2)	105841 (94526, 117380)	209·7 (187·3, 232·7)
**Antigua and Barbuda**	80 (72, 88)	156·7 (141·6, 173·2)	239 (214, 264)	222·4 (200·3, 245·3)
**The Bahamas**	330 (294, 365)	164·3 (147·5, 181·7)	890 (794, 994)	207 (185·3, 230·1)
**Barbados**	360 (324, 397)	143 (128·2, 158·6)	832 (747, 922)	205·6 (184·5, 227·4)
**Belize**	173 (156, 190)	157·6 (141·8, 174)	846 (760, 938)	252·2 (226·3, 278·4)
**Bermuda**	101 (89, 113)	148·6 (130·8, 166·1)	208 (185, 232)	210 (186·6, 234·3)
**Cuba**	12968 (11740, 14304)	122 (110·1, 134·6)	33122 (29526, 36601)	205·4 (183·7, 226·9)
**Dominica**	77 (70, 85)	124·9 (112·1, 138·3)	159 (142, 176)	195·2 (174·5, 215·7)
**Dominican Republic**	8293 (7437, 9217)	172·5 (155·1, 191·9)	21742 (19182, 24406)	214·6 (189·6, 240·7)
**Grenada**	93 (84, 102)	144·9 (129·9, 160·4)	249 (224, 275)	198 (177·5, 219·5)
**Guyana**	1002 (895, 1116)	191 (171·1, 212·9)	1815 (1621, 2019)	250·8 (224·7, 277·7)
**Haiti**	5067 (4384, 5856)	118·4 (102·5, 136·3)	16886 (14321, 19603)	181·4 (154·3, 209·1)
**Jamaica**	1935 (1753, 2129)	108·9 (98·6, 120·1)	5435 (4887, 6036)	184·8 (166·4, 205·5)
**Puerto Rico**	8284 (7451, 9150)	229·1 (206, 252·8)	13174 (11924, 14548)	261·1 (235·1, 289·3)

**Saint Lucia**	159 (143, 176)	169·2 (151·1, 187·1)	479 (426, 535)	225·4 (201·4, 250·9)
**Saint Vincent and the Grenadines**	108 (97, 119)	143·8 (128·7, 159·4)	331 (287, 376)	252·2 (218·1, 286·1)
**Suriname**	504 (451, 558)	165·6 (148·6, 183·6)	1490 (1331, 1659)	237·6 (212·5, 263·5)
**Trinidad and Tobago**	1491 (1340, 1661)	151·3 (136, 167·4)	3767 (3375, 4179)	217·5 (195·1, 241·3)
**Virgin Islands**	189 (167, 211)	177·9 (158·5, 197·5)	370 (330, 411)	253·2 (225·8, 282·1)
**Tropical Latin America**	198969 (182280, 215191)	166·8 (153·7, 180·1)	492530 (449825, 536995)	199·7 (182·6, 217·6)
**Brazil**	195694 (179542, 211780)	167·9 (154·8, 181·3)	481200 (439973, 524471)	200·1 (183·1, 217·7)
**Paraguay**	3275 (2831, 3760)	118·6 (103·3, 135·7)	11330 (9816, 12921)	184·7 (160·1, 210·1)
**Southern Latin America**	44940 (40751, 49409)	94·6 (85·8, 104)	104162 (94050, 115488)	140·4 (126·9, 155·7)
**Argentina**	25522 (23080, 28099)	78·7 (71, 86·8)	60612 (54289, 67686)	125·4 (112·3, 140·3)
**Chile**	16786 (15114, 18596)	143·6 (129·6, 158·7)	38278 (34650, 42200)	175·6 (158·4, 193·2)
**Uruguay**	2631 (2397, 2887)	77·8 (70·4, 85·5)	5268 (4714, 5814)	129·2 (115·5, 143·2)
**East Asia**	1108634 (1017388, 1198686)	97·5 (89·5, 105·6)	2702425 (2472340, 2941968)	135·1 (123·8, 146·8)
**China**	1050721 (964184, 1136109)	97·4 (89·5, 105·4)	2557163 (2338289, 2783312)	134·4 (123·3, 145·9)
**North Korea**	13728 (11788, 15663)	68·8 (59·5, 78)	37009 (31867, 42667)	116 (100·6, 133·9)
**Taiwan (Province of China)**	25714 (22374, 29517)	133·1 (116·3, 152·2)	64719 (56405, 74030)	191·3 (165·8, 218·4)
**Southeast Asia**	150211 (134393, 167862)	43·1 (38·5, 47·8)	425104 (373523, 479735)	61 (54, 68·5)
**Cambodia**	3171 (2772, 3612)	50·4 (43·7, 57·3)	9785 (8326, 11325)	69·1 (59·2, 79·9)
**Indonesia**	70478 (63252, 77915)	50·2 (45·4, 55·4)	137709 (122823, 153494)	51·4 (46·1, 56·9)
**Laos**	982 (850, 1122)	35·5 (30·8, 40·7)	3225 (2765, 3726)	55·5 (47·7, 63·9)
**Malaysia**	3538 (2995, 4129)	26·9 (22·9, 31·2)	16790 (14409, 19550)	55·1 (47·5, 64)
**Maldives**	38 (33, 43)	28·6 (25·1, 32·5)	252 (216, 291)	56·8 (49·2, 64·5)
**Mauritius**	425 (369, 489)	44·6 (38·8, 51·3)	1020 (883, 1163)	62 (53·8, 70·2)
**Myanmar**	13560 (11734, 15645)	44·9 (38·6, 51·9)	36144 (31091, 41919)	66·9 (57·9, 77·1)
**Philippines**	6840 (6009, 7737)	15·3 (13·5, 17·3)	29956 (25716, 34333)	32·4 (28, 37·1)

**Sri Lanka**	5350 (4654, 6103)	37 (32·3, 42)	14125 (12264, 16079)	56·5 (49·4, 64·3)
**Seychelles**	26 (22, 30)	44·5 (37·8, 51·4)	105 (87, 125)	86·9 (72·6, 102)
**Thailand**	22596 (19208, 26586)	46·2 (39·5, 54)	77118 (65494, 91004)	79·9 (68·5, 93·5)
**East Timor**	160 (139, 186)	31·9 (27·6, 36·4)	501 (429, 577)	54·4 (46·3, 62·7)
**Vietnam**	22848 (20049, 25745)	48·4 (42·3, 55)	97815 (83397, 112977)	91·9 (78·8, 105·3)
**Oceania**	2462 (2106, 2851)	53·4 (45·7, 61·7)	7653 (6395, 9083)	74·9 (63·3, 88·1)
**American Samoa**	32 (26, 39)	91·3 (74·6, 110·5)	72 (58, 86)	135·4 (110·6, 163·1)
**Federated States of Micronesia**	47 (41, 54)	69·7 (60·8, 79·4)	97 (85, 111)	103·1 (89·9, 117)
**Fiji**	245 (214, 279)	41·9 (36·6, 47·4)	690 (599, 787)	76·2 (66·2, 86·6)
**Guam**	94 (82, 107)	80·2 (70·5, 90·9)	200 (174, 226)	109·6 (95·5, 124·2)
**Kiribati**	39 (34, 44)	70·4 (61·2, 79·8)	107 (94, 122)	108·9 (95, 123·9)
**Marshall Islands**	21 (18, 24)	78·6 (68·6, 89·3)	56 (49, 64)	112·8 (98·3, 127·5)
**Northern Mariana Islands**	33 (28, 38)	84 (73·4, 94·7)	59 (51, 68)	100·8 (88·1, 114·2)
**Papua New Guinea**	1442 (1203, 1699)	49·6 (41·6, 58·4)	5006 (4093, 6042)	68·2 (56·4, 81·7)
**Samoa**	91 (79, 104)	80·9 (70·2, 92·7)	193 (167, 220)	116·8 (100·9, 133)
**Solomon Islands**	123 (106, 141)	57·5 (49·5, 66·1)	421 (363, 487)	85·7 (73·7, 98·1)
**Tonga**	64 (55, 73)	92·4 (79·3, 105·2)	130 (111, 149)	143·1 (122·5, 164·3)
**Vanuatu**	69 (58, 80)	67·7 (57, 79·2)	200 (169, 234)	89·2 (74·8, 103·9)
**North Africa and Middle East**	395083 (352798, 443650)	170·1 (151·7, 190·7)	1248732 (1110015, 1402150)	228·3 (203·7, 255)
**Afghanistan**	12939 (11372, 14644)	171·1 (150·7, 193·6)	39049 (32244, 46048)	210·4 (176·1, 245·4)
**Algeria**	23933 (21282, 26697)	147·5 (130·5, 165)	88837 (78271, 98982)	225 (199·4, 250·6)
**Bahrain**	709 (629, 795)	189·7 (170·8, 208·8)	4456 (3921, 5000)	258·4 (232·2, 285·1)
**Egypt**	124238 (105150, 145475)	312·3 (264·4, 364·9)	276717 (235888, 321818)	340·2 (292, 393·6)
**Iran**	38147 (35046, 41430)	100·7 (92·2, 109·3)	139487 (127322, 152034)	162·3 (148·4, 176·2)
**Iraq**	18409 (16547, 20375)	172·1 (155·2, 189·9)	60083 (53625, 66748)	184·6 (165·8, 204·2)

**Jordan**	4504 (4055, 4989)	205·4 (185·5, 225·9)	28125 (25315, 31066)	324·5 (293·7, 357)
**Kuwait**	2062 (1805, 2332)	152·6 (135·9, 169·8)	12105 (10415, 13689)	262·3 (231·7, 293·9)
**Lebanon**	4570 (4123, 5040)	159·7 (143·8, 175·8)	18656 (16603, 20707)	253·6 (226·8, 280·2)
**Libya**	5244 (4700, 5824)	198·9 (177·8, 220·4)	20715 (18360, 23176)	315·3 (283, 348·7)
**Morocco**	27281 (24192, 30605)	150·1 (133·1, 168·2)	82982 (73157, 93288)	231·2 (204·3, 259·2)
**Palestine**	1922 (1747, 2107)	166·8 (150·8, 183·1)	7972 (7077, 8860)	227·7 (202·6, 253·3)
**Oman**	1813 (1605, 2050)	148·9 (133, 165·5)	10908 (9590, 12238)	265·3 (237·8, 294·2)
**Qatar**	909 (741, 1087)	256·1 (217·4, 297·5)	8529 (7309, 9780)	304 (269·4, 341)
**Saudi Arabia**	17810 (15356, 20673)	174·7 (152·2, 200·1)	93459 (78870, 110286)	276·3 (238·4, 318·3)
**Sudan**	21706 (19104, 24538)	172·3 (151·6, 196)	63806 (55371, 72801)	239·4 (208·1, 270·3)
**Syria**	12329 (11147, 13594)	164·8 (148·6, 181·1)	38919 (34845, 43172)	243·9 (219·8, 269·7)
**Tunisia**	14835 (12578, 17543)	234 (196·9, 276·1)	45139 (37987, 53057)	349·8 (296, 409·7)
**Turkey**	48173 (42381, 54631)	107·5 (94·5, 121·5)	138696 (122292, 157530)	154·2 (136·1, 174·8)
**United Arab Emirates**	2413 (2095, 2738)	171·4 (154, 189·7)	32152 (27745, 36819)	265·9 (237·7, 294·6)
**Yemen**	10884 (9355, 12536)	148·3 (126·2, 170·7)	36774 (30667, 43534)	189·1 (158·1, 223·8)
**South Asia**	210504 (189894, 230604)	24·7 (22·4, 27·2)	529105 (473752, 587934)	31·9 (28·6, 35·3)
**Bangladesh**	23231 (20496, 26035)	32·5 (28·5, 36·6)	53111 (46425, 59499)	36·3 (31·8, 40·7)
**Bhutan**	143 (125, 163)	38·2 (33·4, 43·7)	378 (327, 433)	43·4 (37·7, 49·6)
**India**	158631 (144332, 172708)	22·9 (20·9, 25)	400325 (360972, 439858)	30·3 (27·3, 33·3)
**Nepal**	3470 (2985, 4020)	25 (21·5, 28·9)	8187 (6976, 9481)	31·3 (26·7, 36·2)
**Pakistan**	25030 (21361, 29244)	34·2 (29·3, 40)	67103 (56856, 78847)	40·4 (34·4, 47·2)
**Southern sub-Saharan Africa**	41378 (37301, 45595)	112·6 (101·4, 123·9)	76067 (67708, 84642)	110·1 (98·6, 122·2)
**Botswana**	638 (558, 730)	80 (69·8, 91·6)	2180 (1879, 2520)	110 (96·3, 125·7)
**Lesotho**	1052 (913, 1194)	86·4 (75·6, 98·3)	1683 (1451, 1941)	107·3 (93·3, 123·2)
**Namibia**	554 (477, 641)	61·1 (52·6, 70·6)	1505 (1273, 1759)	82 (70·1, 95)

**South Africa**	32540 (29550, 35536)	118·2 (107·2, 128·9)	58714 (52806, 64627)	111·1 (100·4, 122·1)
**Swaziland**	503 (441, 571)	111·8 (98·2, 126·3)	1019 (887, 1168)	125·4 (109·8, 142·5)
**Zimbabwe**	6091 (5281, 6981)	104 (90·2, 119·2)	10966 (9201, 12952)	108·3 (91·7, 127)
**Western sub-Saharan Africa**	136431 (121562, 152996)	112·4 (100·2, 125·6)	354622 (312352, 400636)	129·1 (114·3, 145·4)
**Benin**	3038 (2699, 3422)	111·1 (98·5, 124·1)	10179 (8917, 11580)	142·9 (126·2, 161·8)
**Burkina Faso**	6386 (5633, 7161)	112 (99, 125·9)	18443 (16087, 21072)	140·8 (122·9, 159·9)
**Cameroon**	8722 (7582, 9902)	136·9 (119·7, 155·3)	27053 (23533, 30982)	151·9 (132, 173·1)
**Cape Verde**	196 (176, 217)	89 (79·4, 99·2)	662 (583, 746)	132·6 (116·8, 148·9)
**Chad**	3693 (3231, 4181)	103·3 (90, 116·5)	9132 (7880, 10476)	111·3 (96·6, 127)
**Côte d'Ivoire**	7354 (6470, 8284)	103·4 (91·9, 116·2)	21521 (18822, 24483)	125·4 (110·5, 141·3)
**The Gambia**	598 (530, 674)	106·6 (94·8, 119·8)	1840 (1623, 2075)	135·6 (120·3, 152·6)
**Ghana**	9712 (8581, 10915)	102·6 (90·9, 114·7)	30500 (26834, 34499)	135·1 (119·4, 152·1)
**Guinea**	4415 (3920, 4942)	108 (95·7, 120·9)	8920 (7797, 10188)	120·5 (105·6, 137·7)
**Guinea-Bissau**	707 (625, 791)	120·4 (107, 134·5)	1631 (1426, 1847)	140·4 (123·6, 158·3)
**Liberia**	1790 (1575, 2022)	132·4 (116·8, 149·3)	4829 (4160, 5526)	149·8 (130·7, 169·8)
**Mali**	6726 (5885, 7583)	122·8 (108·3, 138·2)	17077 (14934, 19421)	144·8 (126·5, 164·3)
**Mauritania**	1527 (1346, 1726)	117 (103·5, 132·1)	4016 (3487, 4614)	151·6 (131·8, 175)
**Niger**	4733 (4171, 5359)	105·5 (93·1, 119)	14470 (12519, 16598)	128·8 (112·6, 146·8)
**Nigeria**	66849 (59353, 75022)	112·6 (100·1, 126·2)	157985 (138484, 179368)	122·6 (107·7, 138·8)
**São Tomé and Príncipe**	117 (104, 130)	158 (140·1, 176·7)	264 (232, 299)	177·5 (156·6, 200)
**Senegal**	4352 (3862, 4866)	97·7 (86·8, 108·6)	12705 (11171, 14296)	128·5 (113·7, 144·8)
**Sierra Leone**	3336 (2950, 3745)	133·6 (118·3, 149·8)	6879 (6005, 7835)	133·9 (117, 151·1)
**Togo**	2176 (1893, 2468)	107·5 (95·3, 121·5)	6513 (5658, 7427)	121 (106·5, 137·1)
**Eastern sub-Saharan Africa**	123987 (111000, 137630)	112·1 (100·6, 124·2)	300870 (266515, 336881)	123·9 (110·5, 137·6)
**Burundi**	3976 (3484, 4498)	122·5 (108, 138·2)	7945 (6868, 9153)	118·2 (102·7, 134·8)

**Comoros**	292 (256, 328)	105·1 (92·3, 117·7)	758 (663, 858)	129·6 (113·9, 146·5)
**Djibouti**	278 (243, 312)	99·1 (87·6, 110·8)	1329 (1145, 1532)	143·6 (124·9, 163·5)
**Eritrea**	1704 (1481, 1941)	105·7 (92·3, 120·4)	4677 (4018, 5388)	121·2 (105·2, 138·1)
**Ethiopia**	28924 (26112, 31891)	96·2 (87·3, 105·5)	61653 (55736, 67834)	98·9 (89·4, 108·4)
**Kenya**	18131 (16427, 20140)	146·1 (132, 161·6)	50642 (45328, 56694)	153·8 (137·9, 171·3)
**Madagascar**	7350 (6446, 8248)	102·5 (90·7, 115·4)	18603 (16120, 21464)	111·4 (97·6, 127·4)
**Malawi**	7508 (6592, 8449)	131·8 (116·4, 148·6)	15473 (13546, 17542)	146·2 (128·2, 165·5)
**Mozambique**	8726 (7693, 9881)	98 (86·8, 110·4)	22582 (19780, 25554)	130 (114·6, 146·3)
**Rwanda**	5683 (4885, 6525)	133·8 (115·5, 152·4)	10714 (9181, 12403)	124·6 (107·4, 142·8)
**Somalia**	4759 (4134, 5434)	107·6 (94·2, 121·1)	12783 (11114, 14629)	126·4 (110·6, 142·8)
**South Sudan**	4358 (3783, 4990)	123·7 (107·6, 141·3)	8715 (7570, 10033)	143·4 (126, 163·1)
**Tanzania**	16478 (14674, 18390)	109·5 (97·6, 122·7)	44779 (39111, 50671)	130 (114·1, 146·1)
**Uganda**	9871 (8715, 11118)	105·4 (92·8, 118·9)	26060 (22718, 29612)	122 (106·6, 137·5)
**Zambia**	5888 (5181, 6641)	136 (120, 153·1)	13969 (12252, 15930)	132·9 (117·2, 151·2)
**Central sub-Saharan Africa**	30441 (26873, 34300)	90·1 (79·7, 101)	82183 (70848, 94978)	102·9 (89·3, 118·1)
**Angola**	6060 (5304, 6861)	95·8 (84, 107·8)	21585 (18907, 24509)	124·2 (109·3, 140·2)
**Central African Republic**	1403 (1227, 1606)	80·1 (70·3, 90·9)	2919 (2475, 3429)	87·4 (74·9, 102·2)
**Congo**	1352 (1196, 1521)	90·1 (79·5, 101·4)	3981 (3448, 4561)	101·8 (89·2, 115·8)
**DR Congo**	20688 (18160, 23330)	88·9 (78·3, 100)	50808 (43403, 59234)	95·9 (82·4, 111·2)
**Equatorial Guinea**	241 (211, 273)	89·8 (78·7, 101·4)	1209 (994, 1455)	151·6 (125·3, 181·2)
**Gabon**	696 (613, 783)	103 (90·6, 116·3)	1679 (1451, 1913)	119·6 (104, 136·2)

Appendix Table 25: Number of prevalent cases and age-standardised prevalence rates of compensated cirrhosis due to other causes per 100 000 in 1990 and 2017 for both sexes by location

	1990		2017	
	Counts (95% UI)	Rate (95% UI)	Counts (95% UI)	Rate (95% UI)
**Global**	10359652 (9472360, 11218885)	189 (173, 204·6)	14963062 (13571830, 16244968)	198·4 (180·1, 215·4)
**High-income North America**	281504 (257327, 303584)	90·6 (82·9, 97·8)	431998 (393515, 470270)	91·7 (84·5, 99·2)
**Canada**	22225 (19546, 25165)	73·7 (65·2, 83)	36956 (31787, 42661)	76·2 (66·6, 86·1)
**Greenland**	34 (30, 39)	65·5 (57·2, 74·2)	46 (39, 52)	71·2 (62·6, 80·5)
**USA**	259238 (238071, 279499)	92·4 (84·9, 99·8)	394989 (360603, 428858)	93·5 (86·3, 100·7)
**Australasia**	14835 (13249, 16409)	76·5 (68·4, 84·4)	22158 (19727, 24627)	85·6 (76·6, 95)
**Australia**	11464 (10122, 12773)	70·9 (62·9, 78·7)	17764 (15685, 19940)	80·8 (71·6, 90·2)
**New Zealand**	3371 (3060, 3688)	103·6 (93·7, 113·6)	4394 (4016, 4767)	110·3 (100·3, 120·2)
**High-income Asia-Pacific**	1030186 (934881, 1136660)	637·9 (584, 695·1)	826087 (754654, 903917)	551 (507·7, 593·3)
**Brunei**	1570 (1417, 1729)	582·2 (527·6, 637·6)	2521 (2252, 2809)	628·3 (570, 695·9)
**Japan**	606282 (562677, 650043)	543·8 (504, 585·6)	520637 (482378, 560539)	525·1 (484·7, 566·3)
**Singapore**	17015 (15230, 19007)	577·5 (522, 636·1)	29267 (25828, 32991)	599·6 (540·8, 664·8)
**South Korea**	405319 (347606, 494124)	945·3 (807·6, 1166·5)	273661 (239760, 317062)	604·3 (545·6, 672·3)
**Western Europe**	1035172 (933487, 1145755)	254·7 (230·5, 280·3)	1312296 (1187403, 1451965)	288·7 (263·7, 316·9)
**Andorra**	119 (105, 136)	214 (191·1, 239·9)	221 (193, 252)	269·5 (240·7, 301·8)
**Austria**	28557 (25165, 32745)	348·9 (311, 391·9)	34177 (30218, 38524)	369·1 (329·9, 409·9)
**Belgium**	20719 (17691, 23795)	200·8 (173·6, 228·3)	26915 (23080, 30897)	231·5 (201·8, 263·9)
**Cyprus**	1210 (1082, 1361)	153·2 (136·9, 171·7)	2245 (1973, 2562)	173·2 (154·4, 193·9)
**Denmark**	10240 (9143, 11494)	196·7 (177, 220)	17440 (15570, 19588)	294 (264·9, 328·1)
**Finland**	10460 (9343, 11718)	211·2 (189·6, 234·8)	17341 (15436, 19589)	297·1 (267·3, 332·5)
**France**	149339 (131114, 167927)	245·6 (217·9, 274·6)	177339 (158219, 199294)	259·6 (234·1, 290·2)

**Germany**	209038 (184107, 236882)	246·4 (220·3, 275·6)	287894 (252842, 325907)	319·8 (285·6, 356·7)
**Greece**	24426 (21553, 27606)	220·2 (196·5, 246·3)	25642 (22782, 29042)	240·4 (215·5, 267·7)
**Iceland**	445 (395, 496)	171·3 (153·4, 190·4)	975 (863, 1105)	278·7 (248, 312·9)
**Ireland**	6134 (5557, 6741)	166·1 (150·4, 183·1)	11659 (10352, 13069)	240·4 (214·8, 267·4)
**Israel**	10929 (9860, 12113)	219·6 (196·5, 245·6)	24424 (21926, 27263)	270·4 (242·5, 302·5)
**Italy**	194782 (165968, 229040)	317·7 (276·3, 367·8)	193431 (168385, 223963)	310·6 (275·4, 350·7)
**Luxembourg**	1337 (1177, 1531)	327·1 (291·6, 369·8)	2233 (1970, 2518)	363·6 (324·2, 405·4)
**Malta**	515 (458, 582)	136·8 (122·3, 153·3)	708 (619, 806)	157·5 (140·5, 176)
**Netherlands**	30649 (27256, 34402)	198·8 (178·2, 220·5)	43390 (38453, 48594)	244·7 (220·6, 271·8)
**Norway**	12150 (11255, 13048)	287·8 (265·7, 309·8)	17455 (16180, 18865)	346·9 (320·4, 373·2)
**Portugal**	23961 (20547, 27619)	215·4 (186·5, 247·1)	21369 (18730, 24435)	190·2 (169·1, 214·2)
**Spain**	56994 (48908, 65155)	142·4 (122·5, 162)	59197 (50906, 67555)	149·1 (129, 169·6)
**Sweden**	25763 (23727, 27731)	303·3 (279·9, 324·9)	43981 (40448, 47862)	440·9 (406·3, 478)
**Switzerland**	12506 (11108, 14118)	175·6 (157·5, 196)	18201 (16082, 20565)	204·6 (182·6, 227·5)
**United Kingdom**	203901 (190561, 216363)	335·2 (312·2, 356·7)	284698 (265779, 305707)	382·8 (357·7, 409·8)
**Eastern Europe**	627073 (575922, 678934)	304 (278·9, 330)	700578 (640477, 759171)	401·2 (366, 438·1)
**Belarus**	26043 (23242, 28918)	273·2 (243·6, 303·1)	27793 (24534, 31237)	359·7 (317·7, 400·3)
**Estonia**	3813 (3389, 4247)	271 (239·5, 301·3)	3618 (3201, 4047)	339·8 (301·2, 380)
**Latvia**	6137 (5497, 6830)	253·6 (228·1, 281)	5115 (4540, 5696)	328 (292·2, 363·2)
**Lithuania**	10240 (9112, 11343)	296·1 (262·7, 326·3)	8983 (7996, 10065)	382·1 (337·9, 427·1)
**Moldova**	20369 (18078, 22798)	463 (410·9, 517·6)	15164 (13270, 17212)	447·9 (395·1, 501·2)
**Russia**	414044 (379988, 448025)	300·6 (276·3, 326·8)	488790 (447532, 530231)	399·5 (365·3, 436·6)
**Ukraine**	146426 (134353, 159028)	311·7 (285·7, 339·8)	151115 (138306, 163590)	418·5 (382·5, 455·5)
**Central Europe**	399863 (362665, 438474)	346·4 (313·8, 379·5)	324349 (289749, 360324)	358·7 (322·4, 395·2)
**Albania**	11172 (10009, 12314)	306·8 (276·3, 337·4)	8399 (7451, 9376)	340·3 (305·4, 376·2)

**Bosnia and Herzegovina**	14345 (12794, 15854)	329·4 (296·2, 363·2)	9452 (8362, 10612)	342·6 (305·1, 379·3)
**Bulgaria**	23843 (21570, 26277)	307·8 (278·5, 338·9)	17714 (15738, 19813)	333·7 (298·7, 369·6)
**Croatia**	15683 (13943, 17381)	358·6 (321·4, 396·4)	10876 (9660, 12100)	315·8 (284, 347·8)
**Czech Republic**	26001 (23446, 28567)	278·3 (250·5, 306·3)	24846 (22202, 27532)	306 (275·3, 338)
**Hungary**	36353 (32631, 40375)	386·7 (345·9, 429·4)	24479 (21783, 27260)	327·8 (292·7, 363·7)
**Macedonia**	5494 (4912, 6085)	278·9 (250, 308·1)	5438 (4847, 6080)	309·4 (276·1, 342·5)
**Montenegro**	1569 (1407, 1726)	258·7 (232, 284·3)	1598 (1418, 1783)	304·8 (272·4, 338·4)
**Poland**	127719 (115760, 140351)	350·3 (317·4, 386·2)	118124 (105227, 132340)	403 (360·6, 448)
**Romania**	90026 (80498, 99067)	403·7 (360·5, 445)	61807 (54860, 69181)	384·7 (344·5, 426·9)
**Serbia**	16269 (14558, 17991)	189·5 (170, 208·8)	15644 (13950, 17370)	212·2 (191·4, 234·4)
**Slovakia**	24787 (22201, 27285)	495·1 (443·2, 546·4)	20867 (18702, 23339)	499·5 (448·7, 553·2)
**Slovenia**	6602 (5915, 7329)	362·4 (324·6, 401·3)	5106 (4533, 5678)	314·4 (281·4, 348·4)
**Central Asia**	175528 (158637, 192016)	229·9 (208, 251·8)	250080 (220986, 279386)	278·1 (247·4, 308·6)
**Armenia**	6376 (5672, 7056)	182·7 (163·4, 202·4)	6330 (5590, 7089)	241·2 (214·7, 268·5)
**Azerbaijan**	16265 (14522, 17940)	208 (186·3, 229·2)	23705 (20809, 26575)	248·2 (219·8, 276·3)
**Georgia**	10255 (9155, 11305)	194·3 (173·9, 214·5)	6860 (5705, 7738)	221·3 (182·2, 248·8)
**Kazakhstan**	31791 (28503, 35328)	181 (162·4, 201)	45475 (40068, 50994)	267·9 (236·5, 298·8)
**Kyrgyzstan**	11549 (10281, 12737)	237·5 (211·7, 261·3)	18614 (16475, 20819)	282·5 (249·8, 315·5)
**Mongolia**	6739 (6011, 7443)	269·7 (241·1, 297·9)	8178 (7172, 9197)	260 (229·3, 290)
**Tajikistan**	16267 (14655, 17895)	254·1 (228, 278·3)	27514 (24339, 30614)	273·4 (242·6, 303·1)
**Turkmenistan**	11396 (10204, 12541)	269·9 (242·1, 296·7)	15841 (14035, 17772)	319·6 (283·9, 356·6)
**Uzbekistan**	64890 (58808, 71453)	271·2 (244·4, 298·1)	97563 (86126, 109052)	296 (263·6, 329·9)
**Central Latin America**	328458 (301005, 355445)	222·6 (203·9, 240·6)	577574 (525177, 634747)	230 (209·2, 252·3)
**Colombia**	42380 (37903, 47046)	133·7 (119, 148·9)	80819 (70710, 91765)	157·9 (138·6, 179)
**Costa Rica**	4310 (3806, 4800)	150·8 (132·5, 169·5)	8661 (7491, 9902)	182·8 (158·6, 207·7)

**El Salvador**	9197 (8204, 10167)	183·5 (161·2, 206·3)	12773 (11124, 14657)	214·1 (186·2, 246·6)
**Guatemala**	17929 (16177, 19756)	236·6 (209·1, 265·6)	37519 (32604, 42842)	248·4 (213·8, 284·6)
**Honduras**	10969 (9932, 12100)	247·5 (218·3, 278·8)	20714 (18039, 23564)	245·2 (210·5, 283)
**Mexico**	207778 (191669, 223185)	282·4 (260·8, 303·6)	346620 (319462, 375599)	278·1 (256·5, 301·3)
**Nicaragua**	5864 (5199, 6512)	165·2 (145·1, 186·9)	11619 (10078, 13294)	195·3 (169·3, 224·1)
**Panama**	3359 (2999, 3738)	145·6 (128·9, 163·6)	6853 (5972, 7827)	173·9 (151·2, 198·1)
**Venezuela**	26674 (23629, 29734)	149·7 (131·5, 168·5)	51996 (45197, 59567)	171·3 (149·7, 194·9)
**Andean Latin America**	89478 (81027, 98190)	226·9 (203·6, 250·9)	175148 (156399, 193075)	287·3 (256, 316·8)
**Bolivia**	13998 (12523, 15526)	216 (191·6, 239·9)	33888 (30099, 37750)	297 (263·8, 331·4)
**Ecuador**	23379 (21255, 25766)	226·3 (203·5, 249·2)	49051 (43626, 54611)	295·2 (262·4, 328·5)
**Peru**	52101 (47085, 57400)	230·4 (206, 255·3)	92210 (82128, 101917)	280·4 (249·7, 310·1)
**Caribbean**	54771 (49005, 60727)	160·5 (142·5, 179·1)	92951 (82259, 103536)	197·7 (175·3, 219·9)
**Antigua and Barbuda**	89 (79, 99)	150·9 (134·2, 168·3)	171 (150, 192)	189·3 (167·5, 210·4)
**The Bahamas**	395 (349, 441)	163·4 (143·6, 183·1)	744 (653, 834)	197·3 (174·6, 219·8)
**Barbados**	357 (318, 398)	137·7 (122·6, 153·2)	564 (493, 633)	179·4 (158·9, 200·3)
**Belize**	285 (254, 316)	163·1 (144·2, 182·9)	789 (695, 877)	206·3 (182·5, 229·2)
**Bermuda**	81 (71, 91)	139·6 (123·7, 155·4)	122 (106, 138)	173·7 (153·8, 193·7)
**Cuba**	15000 (13346, 16746)	138·7 (123·5, 154·2)	23138 (20185, 26165)	189 (167, 211)
**Dominica**	107 (96, 119)	143 (126·8, 159·1)	133 (117, 148)	184 (162·7, 204·6)
**Dominican Republic**	13282 (11900, 14745)	196·8 (174·3, 221·2)	21199 (18584, 23706)	206·1 (180·7, 230·2)
**Grenada**	133 (118, 147)	157·6 (138·6, 176·5)	216 (191, 239)	186·7 (165·2, 207·1)
**Guyana**	1285 (1143, 1434)	181·8 (159·3, 204·4)	1572 (1386, 1758)	213·7 (188·9, 239·7)
**Haiti**	8890 (7899, 9857)	149·5 (132·2, 168)	22801 (20144, 25335)	204·4 (179·7, 228·3)
**Jamaica**	3181 (2849, 3510)	133 (118·3, 148·3)	5005 (4415, 5595)	178·1 (157·4, 198·4)
**Puerto Rico**	6908 (6091, 7760)	189·4 (167·1, 213·4)	8593 (7540, 9717)	205·8 (182·1, 229)

**Saint Lucia**	214 (190, 237)	165·8 (145·5, 187·2)	344 (303, 384)	190·9 (169·3, 211·9)
**Saint Vincent and the Grenadines**	150 (134, 167)	138·6 (123·2, 154·5)	201 (177, 225)	173·3 (153, 193·6)
**Suriname**	641 (570, 718)	173·3 (152·7, 194·5)	1193 (1047, 1339)	205·7 (182, 229·2)
**Trinidad and Tobago**	1734 (1541, 1931)	148·7 (131·4, 165·5)	2587 (2262, 2896)	182·1 (161·1, 203)
**Virgin Islands**	165 (145, 186)	159·2 (140·3, 178·6)	237 (204, 268)	202 (178·6, 224·8)
**Tropical Latin America**	243760 (224174, 262354)	170 (157·1, 182·7)	353651 (325251, 381612)	156 (143·8, 167·6)
**Brazil**	239266 (220079, 257487)	171·1 (158·2, 183·9)	343969 (316124, 370627)	156·4 (144·3, 167·9)
**Paraguay**	4494 (3976, 4996)	124·5 (109·3, 139)	9682 (8451, 10957)	145·6 (127·4, 164·8)
**Southern Latin America**	103433 (92067, 115546)	209·5 (185·6, 234·8)	181804 (159945, 204029)	266·5 (235·8, 298·5)
**Argentina**	63838 (57083, 70972)	191·3 (170·5, 213·7)	115696 (101892, 129598)	254·7 (224·1, 284·9)
**Chile**	33642 (29499, 37922)	267·9 (233·8, 303·2)	57188 (50092, 64922)	297 (262·4, 334·1)
**Uruguay**	5949 (5280, 6636)	182·6 (162·4, 204·2)	8911 (7884, 10013)	245·3 (217·9, 274·8)
**East Asia**	1748825 (1584713, 1916355)	138·9 (126·1, 151·6)	1872547 (1706600, 2046018)	136·5 (124·4, 148·1)
**China**	1671669 (1514018, 1832364)	139·4 (126·4, 152·2)	1762385 (1604886, 1924581)	135·3 (123·7, 146·8)
**North Korea**	23138 (20212, 25966)	116·5 (102·1, 129·9)	38961 (33984, 44228)	160·9 (142·7, 179·7)
**Taiwan (Province of China)**	24882 (21716, 28109)	127·1 (111·4, 142·9)	41036 (35081, 47026)	179·6 (158·4, 201·4)
**Southeast Asia**	249118 (223269, 274627)	48·6 (43·6, 53·5)	347172 (305590, 387369)	53·8 (47·4, 59·9)
**Cambodia**	6270 (5557, 7055)	52·8 (46·8, 59·2)	9985 (8723, 11280)	61·1 (53·5, 68·8)
**Indonesia**	119568 (108001, 131141)	57·9 (52·6, 63·4)	118974 (106586, 131449)	47 (42·1, 51·7)
**Laos**	1856 (1624, 2076)	39·3 (34·5, 43·9)	3876 (3395, 4362)	53·4 (47, 60·1)
**Malaysia**	5713 (4942, 6530)	29·5 (25·5, 33·3)	12906 (11208, 14660)	43·2 (37·7, 48·8)
**Maldives**	88 (76, 99)	33·4 (29·2, 37·4)	192 (165, 219)	47·2 (41·3, 53·2)
**Mauritius**	443 (385, 498)	39·9 (35, 44·7)	550 (479, 625)	47·6 (41·6, 53·7)
**Myanmar**	20507 (17992, 23069)	46·8 (41·2, 52·3)	30667 (26692, 34669)	57·7 (50·5, 65·2)
**Philippines**	14684 (12737, 16495)	20·1 (17·6, 22·5)	32797 (27729, 37186)	30·6 (26, 34·5)

**Sri Lanka**	6772 (5962, 7636)	38 (33·7, 42·6)	10602 (9207, 11971)	50·5 (44, 56·7)
**Seychelles**	27 (24, 31)	35·8 (31·3, 40·2)	48 (42, 55)	51·2 (44·7, 57·8)
**Thailand**	24725 (21432, 27873)	42·2 (36·9, 47·3)	36630 (31088, 42138)	56·9 (49·3, 64·1)
**East Timor**	311 (273, 349)	36·4 (32, 40·8)	760 (662, 854)	53·2 (46·5, 59·9)
**Vietnam**	47824 (41880, 54061)	62·5 (54·8, 70·3)	88727 (74332, 101561)	99·5 (83·3, 114)
**Oceania**	5421 (4756, 6116)	71·2 (62·6, 80·1)	12658 (11100, 14362)	90·3 (79·3, 101·9)
**American Samoa**	37 (32, 42)	66 (57·8, 74·4)	49 (43, 56)	80·2 (70·1, 91·1)
**Federated States of Micronesia**	103 (90, 116)	80·7 (70·4, 91)	105 (91, 119)	91·8 (80·2, 103·8)
**Fiji**	506 (444, 572)	58·1 (51·4, 65·2)	734 (643, 828)	80·4 (70·5, 90·6)
**Guam**	110 (96, 124)	79·3 (69·8, 89)	144 (127, 163)	88 (77·5, 99·4)
**Kiribati**	68 (60, 77)	80·2 (70·9, 90·3)	128 (112, 145)	98·1 (85·8, 110·9)
**Marshall Islands**	47 (41, 53)	80·1 (70·3, 90·5)	57 (50, 65)	93·5 (81·9, 106·4)
**Northern Mariana Islands**	32 (28, 37)	74·6 (65·8, 84·2)	37 (32, 42)	87·6 (77·4, 98·8)
**Papua New Guinea**	3443 (3012, 3903)	71·5 (62·7, 80·4)	9383 (8207, 10648)	90·4 (79·3, 101·9)
**Samoa**	154 (132, 175)	76 (66·2, 85·5)	203 (178, 231)	90·2 (79, 102·3)
**Solomon Islands**	305 (267, 346)	72·5 (63·9, 81·8)	680 (594, 770)	92·6 (81·4, 104·3)
**Tonga**	97 (85, 110)	82·8 (72·5, 93·3)	111 (97, 126)	98·1 (86·1, 110·2)
**Vanuatu**	161 (140, 181)	88·9 (78·2, 100·5)	330 (288, 374)	100·9 (88·3, 114·2)
**North Africa and Middle East**	873432 (777637, 971715)	221 (198·3, 245·1)	1470961 (1307487, 1634486)	242·5 (216·2, 268·8)
**Afghanistan**	31096 (27563, 34833)	255·1 (226·1, 285·6)	115268 (101749, 129505)	288 (255·5, 321·7)
**Algeria**	67368 (59526, 75356)	224 (198·5, 250·2)	96698 (84939, 108082)	251·9 (221·9, 281·2)
**Bahrain**	1098 (971, 1237)	222·9 (197·6, 250·2)	2786 (2443, 3123)	242·9 (215·8, 270·7)
**Egypt**	187919 (165200, 213249)	309·2 (272·4, 350·2)	283143 (247158, 321959)	278·2 (242·8, 316·5)
**Iran**	125477 (112984, 138146)	170·9 (154·4, 187·4)	144070 (129274, 158109)	198·6 (178, 218·2)
**Iraq**	45051 (39889, 50385)	212·5 (188·5, 237·1)	110626 (97668, 123570)	227·2 (200·9, 253·1)

**Jordan**	12547 (11084, 14092)	273·3 (243·7, 304·8)	36985 (32485, 41232)	324·6 (285·2, 361·1)
**Kuwait**	3227 (2831, 3621)	181·6 (161·4, 202·2)	7468 (6491, 8417)	218·7 (194, 243·3)
**Lebanon**	10478 (9306, 11705)	218·3 (193·8, 243·8)	21641 (19019, 24191)	251·7 (222·8, 281·2)
**Libya**	10921 (9648, 12238)	218·9 (194·1, 243·5)	17010 (14973, 19056)	254·3 (224·7, 284·6)
**Morocco**	64706 (57428, 72438)	227·2 (201·8, 253·7)	89771 (79431, 100222)	257·1 (228·4, 286·2)
**Palestine**	5589 (4968, 6261)	224·5 (198·8, 250·7)	14192 (12513, 15852)	259·7 (229·7, 289·5)
**Oman**	5007 (4439, 5600)	231·8 (205·4, 258·6)	8779 (7666, 9916)	240·3 (213·2, 267·3)
**Qatar**	844 (741, 952)	216·6 (192·2, 242·9)	4321 (3710, 4941)	233·6 (206·4, 261·9)
**Saudi Arabia**	39661 (34488, 44931)	211·3 (185·1, 239·8)	68312 (58556, 78792)	227·7 (199·2, 258·7)
**Sudan**	60757 (53643, 68045)	257·1 (227·2, 287·1)	129662 (114468, 144694)	280·4 (247·7, 311·6)
**Syria**	35462 (31356, 39697)	225·5 (200·6, 250·8)	51489 (45656, 57540)	250·8 (221·7, 280·2)
**Tunisia**	26361 (23348, 29814)	286·2 (252·5, 322·5)	35475 (30845, 40209)	329 (289, 369·3)
**Turkey**	94593 (83262, 106260)	148·2 (131·4, 166·2)	122293 (107761, 136866)	161·7 (143, 180·7)
**United Arab Emirates**	3797 (3352, 4273)	215·6 (190·8, 240·2)	14560 (12496, 16676)	245·7 (216·9, 274)
**Yemen**	40911 (36320, 45819)	245·7 (218·5, 274·4)	95038 (83575, 106653)	274 (243, 305·6)
**South Asia**	1229280 (1115691, 1342351)	100·5 (91·4, 109·2)	2003469 (1797158, 2196053)	107·8 (96·9, 117·9)
**Bangladesh**	173664 (155426, 192412)	139·7 (124·1, 154·8)	189283 (166185, 210926)	116·8 (102·4, 129·9)
**Bhutan**	752 (667, 835)	121·5 (108·7, 135·6)	1117 (978, 1259)	116 (102·1, 129·7)
**India**	894855 (814123, 978159)	93·7 (85·5, 101·7)	1512026 (1361352, 1655810)	105·8 (95·3, 115·6)
**Nepal**	22043 (19448, 24522)	103·3 (91, 115·1)	32570 (27737, 36973)	101·1 (85·9, 114·7)
**Pakistan**	137966 (121612, 153442)	115·9 (101·7, 130·5)	268473 (234315, 301700)	115·3 (101, 129·2)
**Southern sub-Saharan Africa**	184851 (168302, 201283)	353 (321·7, 383·1)	226147 (204623, 247460)	296·3 (268, 323·5)
**Botswana**	3672 (3253, 4068)	284·9 (252, 318·5)	6569 (5778, 7363)	296 (261·8, 332·3)
**Lesotho**	5487 (4897, 6095)	305·1 (270, 342·4)	6053 (5331, 6782)	311·4 (275, 349·2)
**Namibia**	3356 (2982, 3718)	239·8 (212, 267·7)	6420 (5647, 7166)	275·4 (243·1, 307·7)

**South Africa**	131788 (120741, 142876)	356·8 (327·2, 385·5)	150266 (136777, 163737)	278·7 (254·7, 302·7)
**Swaziland**	2438 (2160, 2709)	315·4 (278·8, 353)	3535 (3123, 3936)	317·6 (280·9, 355·2)
**Zimbabwe**	38109 (33945, 42490)	377·2 (334·2, 422·3)	53305 (47223, 59555)	367·8 (325·3, 410·8)
**Western sub-Saharan Africa**	785095 (705620, 865968)	416·2 (369·6, 465·3)	1789126 (1590093, 1986095)	420·3 (372·2, 470·1)
**Benin**	20369 (18126, 22597)	431·7 (380, 481·8)	48913 (43187, 54359)	425·9 (376·7, 476·2)
**Burkina Faso**	46663 (42039, 51551)	485·4 (431·7, 541·3)	102120 (90214, 114034)	492·3 (434·9, 552·3)
**Cameroon**	41239 (36727, 45733)	414·5 (368·1, 465)	111697 (99041, 124520)	408·6 (360·7, 457·5)
**Cape Verde**	1299 (1162, 1442)	357·9 (318·6, 397·3)	2130 (1882, 2387)	390·9 (346·2, 437)
**Chad**	26342 (23500, 29237)	441·3 (391·1, 491·6)	66100 (58681, 73485)	435·7 (383·9, 487·7)
**Côte d'Ivoire**	47375 (42230, 52905)	396·8 (353·9, 443·2)	101208 (89842, 112551)	412·7 (363·9, 461·8)
**The Gambia**	3895 (3473, 4325)	399·4 (356·8, 444·2)	9332 (8234, 10331)	439·4 (387·5, 490·5)
**Ghana**	56706 (50784, 63195)	393·9 (349·8, 440·7)	120304 (106102, 134341)	407·4 (360·6, 456·9)
**Guinea**	26022 (23292, 28819)	432·3 (384·2, 484·3)	50344 (44798, 56218)	429·9 (379·8, 481·9)
**Guinea-Bissau**	4484 (4000, 4989)	448·9 (397·3, 502·9)	7818 (6893, 8715)	424·3 (372·7, 473·7)
**Liberia**	9059 (8127, 10031)	460·1 (409·2, 515·4)	20758 (18359, 23114)	439·8 (387·5, 491·8)
**Mali**	43417 (38537, 48157)	508·6 (450·9, 569·2)	102230 (91135, 113879)	504·5 (444·6, 564·1)
**Mauritania**	7805 (6927, 8644)	386·8 (343·5, 433·3)	15312 (13628, 16942)	394 (346·9, 439·7)
**Niger**	36708 (32708, 40828)	455·4 (405·9, 508·9)	105204 (92593, 117748)	491·4 (431·6, 550·3)
**Nigeria**	348503 (311712, 385927)	396·3 (351·1, 442·8)	796196 (707658, 881943)	397·6 (353·4, 445·5)
**São Tomé and Príncipe**	707 (636, 779)	570·9 (507·2, 633·3)	1037 (914, 1155)	523·4 (460·9, 582·8)
**Senegal**	29053 (26018, 32133)	378·8 (337, 422·5)	61714 (54691, 68369)	423 (373·3, 474)
**Sierra Leone**	19606 (17418, 21788)	524 (467·2, 588·5)	35879 (31635, 39840)	459·7 (404·3, 514·5)
**Togo**	15822 (14185, 17506)	437·6 (390·2, 488)	30814 (27219, 34398)	416·4 (366·8, 467·5)
**Eastern sub-Saharan Africa**	715709 (644181, 782749)	397·7 (358, 438·7)	1554021 (1393845, 1708680)	410·2 (368·6, 452·2)
**Burundi**	21525 (19207, 23933)	430·7 (382·3, 484·4)	43103 (38067, 48121)	418·2 (368·9, 472·9)

**Comoros**	1620 (1439, 1796)	367·8 (324·5, 415·3)	2892 (2558, 3237)	412·2 (365·6, 463·2)
**Djibouti**	1688 (1496, 1880)	355 (314·8, 398)	4106 (3579, 4641)	385·8 (340·9, 431·7)
**Eritrea**	10732 (9523, 11977)	401·8 (354·5, 452·1)	24352 (21593, 27233)	436·3 (387·8, 488·5)
**Ethiopia**	178553 (162265, 194581)	361·8 (329, 392·9)	381174 (344670, 417183)	365·3 (331·7, 398·5)
**Kenya**	97873 (89064, 106543)	472·9 (430·5, 515·4)	215244 (195298, 234671)	466·4 (423·1, 508)
**Madagascar**	44895 (39975, 49875)	394·8 (350·3, 446·2)	101051 (89837, 112645)	407·3 (362·7, 455·2)
**Malawi**	40519 (36138, 44990)	450·5 (400, 506·3)	75436 (67017, 83961)	462·1 (408·4, 520·2)
**Mozambique**	52352 (47016, 58100)	374·6 (335·1, 420·3)	123525 (109266, 137133)	429·8 (380·9, 481·8)
**Rwanda**	32027 (28259, 35706)	472·8 (415·6, 535·4)	51251 (44801, 57926)	424·2 (371·4, 480·6)
**Somalia**	24442 (21753, 26999)	364·4 (323·3, 409·9)	65334 (58085, 72663)	407 (361·4, 455·6)
**South Sudan**	21677 (19113, 24147)	381·5 (336·9, 429)	39414 (34956, 43928)	415·8 (366·9, 468·8)
**Tanzania**	92937 (83082, 103005)	380 (338·4, 426·8)	205990 (182800, 228913)	402 (357·3, 450·6)
**Uganda**	62275 (55685, 69196)	386·1 (343·5, 433·8)	151894 (134271, 169696)	411·8 (364·6, 462·8)
**Zambia**	32231 (28791, 35927)	441·4 (390·2, 498)	68281 (60545, 76386)	417·3 (369·4, 469)
**Central sub-Saharan Africa**	183859 (163692, 204051)	337 (299·1, 378·1)	438288 (389771, 485837)	360·4 (318·4, 402·2)
**Angola**	35574 (31524, 39728)	351·5 (310·5, 392·6)	99784 (88198, 110654)	359·4 (317·9, 400·6)
**Central African Republic**	8437 (7489, 9367)	311·5 (275·6, 348·6)	16055 (14175, 17923)	337·8 (296·2, 379·2)
**Congo**	7561 (6723, 8419)	314 (278·2, 352·1)	15481 (13687, 17209)	318·5 (281, 356·5)
**DR Congo**	127868 (113663, 142312)	337·1 (299·5, 378·2)	297366 (263706, 329898)	366·7 (322·2, 408·7)
**Equatorial Guinea**	1300 (1154, 1442)	312 (275·2, 349·7)	4253 (3757, 4761)	311·7 (274·3, 348·5)
**Gabon**	3120 (2771, 3468)	324·5 (286·3, 363·7)	5348 (4696, 5940)	313·9 (275·6, 350)

Appendix Table 26: Data quality rating from 0 to 5 stars, maximum percentage well-certified per five-year interval and percentage well-certified across time series for 195 countries and territories, 1980–2017

Country	Data quality rating	1980–1984	1985–1989	1990–1994	1995–1999	2000–2004	2005–2009	2010–2017	1980–2017
Afghanistan	1	0.0	0.0	0.0	0.0	4.6	38.2	0.0	6.1
Albania	3	0.0	66.1	68.6	70.7	71.8	72.5	54.7	57.8
Algeria	1	0.0	0.0	0.0	0.0	0.0	17.3	0.0	2.5
American Samoa	3	0.0	0.0	0.0	73.7	78.2	83.4	79.4	45.0
Andorra	0	0.0	0.0	0.0	0.0	0.0	0.0	0.0	0.0
Angola	1	0.0	0.0	0.0	0.0	0.0	0.0	4.4	0.6
Antigua and Barbuda	4	60.9	72.0	75.1	79.5	79.3	80.0	74.7	74.5
Argentina	4	77.4	70.9	69.7	68.5	66.7	66.6	69.9	70.0
Armenia	5	74.2	78.1	85.0	88.8	88.9	92.7	91.9	85.6
Australia	5	93.2	93.6	92.9	92.5	91.1	90.5	90.0	91.9
Austria	5	89.9	90.9	90.0	88.6	91.9	90.8	89.2	90.2
Azerbaijan	3	75.5	75.4	78.1	72.0	68.2	41.8	0.0	58.7
Bahrain	3	0.0	78.1	0.0	61.6	64.6	55.9	56.8	45.3
Bangladesh	1	2.8	4.5	25.7	4.4	12.4	6.3	6.3	8.9
Barbados	4	75.0	75.8	77.6	77.4	75.6	82.1	82.0	77.9
Belarus	4	84.0	88.9	80.1	82.9	85.4	84.5	85.0	84.4
Belgium	4	77.8	77.6	81.3	84.1	82.8	82.7	80.4	81.0
Belize	4	47.5	52.4	49.8	76.6	78.3	87.1	89.1	68.7
Benin	1	0.0	0.6	0.0	0.0	0.0	0.0	0.0	0.1
Bermuda	5	90.4	88.7	90.2	93.5	91.1	86.4	90.3	90.1
Bhutan	0	0.0	0.0	0.0	0.0	0.0	0.0	0.0	0.0
Bolivia	1	0.0	0.0	0.0	0.0	14.7	0.0	0.0	2.1
Bosnia and Herzegovina	2	0.0	65.8	67.1	0.0	0.0	0.0	71.4	29.2
Botswana	0	0.0	0.0	0.0	0.0	0.0	0.0	0.0	0.0
Brazil	4	54.0	58.9	65.2	68.9	74.6	80.7	82.5	69.3
Brunei	3	0.0	0.0	0.0	66.7	73.2	76.1	74.5	41.5
Bulgaria	4	81.7	82.3	79.6	76.5	71.8	73.5	70.2	76.5

Burkina Faso	1	0.2	0.0	0.0	4.6	6.3	4.6	0.3	2.3
Burundi	1	0.0	0.0	2.2	0.0	0.0	0.0	0.0	0.3
Cambodia	1	0.0	0.0	0.0	0.0	1.6	4.4	0.0	0.9
Cameroon	0	0.0	0.0	0.0	0.0	0.0	0.0	0.0	0.0
Canada	5	89.4	90.0	89.1	88.6	89.3	89.4	90.1	89.4
Cape Verde	2	56.1	0.0	0.1	0.0	0.0	0.0	67.7	17.7
Central African Republic	0	0.0	0.0	0.0	0.0	0.0	0.0	0.0	0.0
Chad	0	0.0	0.0	0.0	0.0	0.0	0.0	0.0	0.0
Chile	4	75.7	75.5	81.7	84.7	90.8	90.8	89.8	84.1
China	3	0.0	0.9	73.8	75.1	70.3	73.0	73.5	52.4
Colombia	4	72.4	74.4	77.3	88.0	88.6	88.9	88.5	82.6
Comoros	0	0.0	0.0	0.0	0.0	0.0	0.0	0.0	0.0
Congo	0	0.0	0.0	0.0	0.0	0.0	0.0	0.0	0.0
Costa Rica	5	81.6	83.4	82.5	91.1	92.3	90.6	90.5	87.4
Côte d’Ivoire	1	0.0	1.2	1.2	0.0	0.0	0.2	0.2	0.4
Croatia	4	0.0	84.4	85.3	82.6	86.1	88.3	92.3	74.1
Cuba	5	84.7	85.1	84.9	88.9	90.2	91.0	91.5	88.0
Cyprus	2	0.0	0.0	0.0	26.8	53.8	59.5	60.9	28.7
Czech Republic	4	0.0	90.7	89.6	84.8	84.7	85.8	87.8	74.8
DR Congo	1	0.0	2.3	3.0	0.0	0.0	0.0	0.0	0.8
Denmark	4	84.5	82.9	83.1	85.8	85.4	84.5	84.6	84.4
Djibouti	0	0.0	0.0	0.0	0.0	0.0	0.0	0.0	0.0
Dominica	4	71.6	63.1	62.1	65.8	68.6	81.7	83.9	71.0
Dominican Republic	3	53.6	53.5	44.5	53.8	57.2	56.8	59.9	54.2
Ecuador	4	73.0	72.4	72.9	69.8	66.4	62.9	70.4	69.7
Egypt	3	35.0	48.2	46.8	0.0	43.9	42.7	48.2	37.8
El Salvador	3	73.3	0.0	60.4	67.2	69.4	70.8	67.4	58.4
Equatorial Guinea	0	0.0	0.0	0.0	0.0	0.0	0.0	0.0	0.0
Eritrea	0	0.0	0.0	0.0	0.0	0.0	0.0	0.0	0.0
Estonia	5	92.1	92.7	93.8	93.1	92.5	94.1	93.9	93.2
Ethiopia	1	0.0	1.2	2.5	0.6	5.0	4.6	4.6	2.6

Federated States of Micronesia	0	0.0	0.0	0.0	0.0	0.0	0.0	0.0	0.0
Fiji	2	0.0	0.0	0.0	34.8	58.0	63.9	70.0	32.4
Finland	5	83.7	91.7	91.7	95.8	95.6	94.9	95.9	92.8
France	4	76.1	78.4	79.1	79.0	79.1	79.4	77.4	78.3
Gabon	0	0.0	0.0	0.0	0.0	0.0	0.0	0.0	0.0
Georgia	4	83.4	80.7	81.3	77.7	78.7	62.2	58.9	74.7
Germany	4	78.4	80.5	84.1	83.9	83.5	83.8	84.0	82.6
Ghana	1	0.0	0.1	1.5	0.8	7.2	16.7	0.5	3.8
Greece	4	80.2	81.6	71.7	72.0	72.6	77.9	76.2	76.0
Greenland	3	0.0	0.0	0.0	90.2	89.7	89.6	87.6	51.0
Grenada	4	67.7	63.4	64.0	63.2	77.0	79.9	85.9	71.6
Guam	3	0.0	0.0	85.9	80.7	76.7	79.9	79.2	57.5
Guatemala	4	80.1	75.0	73.9	72.6	73.3	75.2	78.0	75.4
Guinea	1	0.0	0.0	0.0	3.2	0.0	0.0	0.0	0.5
Guinea-Bissau	1	0.0	0.0	0.1	1.2	0.0	0.0	0.0	0.2
Guyana	4	55.5	76.2	69.8	78.3	84.3	85.5	78.6	75.5
Haiti	1	22.3	1.3	1.0	13.9	6.1	0.0	0.0	6.4
Honduras	2	41.2	46.0	43.2	0.0	0.0	17.5	19.1	23.8
Hungary	5	91.0	89.8	89.9	91.0	92.1	92.6	93.8	91.5
Iceland	5	92.6	93.8	94.2	94.1	93.5	92.9	91.4	93.2
India	2	2.8	3.0	2.9	5.2	6.3	52.7	49.0	17.4
Indonesia	2	1.4	0.0	1.3	0.4	0.3	53.7	62.3	17.1
Iran	2	0.0	0.0	0.0	0.0	52.8	63.7	80.3	28.1
Iraq	2	0.0	0.0	0.0	0.0	0.0	45.8	56.7	14.6
Ireland	5	90.3	91.6	91.7	90.9	90.2	92.4	92.3	91.3
Israel	4	81.2	82.6	83.2	82.7	81.8	80.1	80.7	81.8
Italy	5	88.8	88.4	88.5	87.8	88.1	88.7	88.3	88.4
Jamaica	4	70.5	72.8	64.6	0.0	83.0	87.5	89.1	66.8
Japan	4	82.6	81.0	81.5	87.6	85.7	84.4	81.3	83.4
Jordan	2	0.0	0.0	0.0	1.9	70.1	76.5	74.1	31.8
Kazakhstan	5	80.0	84.9	89.1	88.6	85.0	84.2	84.1	85.1

Kenya	1	0.0	2.7	0.0	0.4	4.9	5.4	0.8	2.0
Kiribati	2	0.0	0.0	47.9	66.6	35.6	0.0	0.0	21.4
Kuwait	4	82.1	81.9	76.4	77.5	83.1	84.9	84.6	81.5
Kyrgyzstan	4	67.5	75.9	72.5	74.4	87.4	92.9	93.2	80.6
Laos	1	0.0	1.3	0.0	0.0	0.0	0.0	0.0	0.2
Latvia	5	92.5	92.7	89.3	92.0	91.1	88.7	94.3	91.5
Lebanon	1	0.0	3.5	0.0	0.0	0.0	0.0	0.0	0.5
Lesotho	0	0.0	0.0	0.0	0.0	0.0	0.0	0.0	0.0
Liberia	1	2.1	2.1	3.2	0.0	0.0	0.0	0.0	1.1
Libya	1	0.0	0.0	0.0	0.0	0.0	12.6	0.0	1.8
Lithuania	5	90.1	94.0	93.7	94.8	92.9	93.3	94.6	93.3
Luxembourg	4	86.4	85.8	85.5	84.5	80.6	77.9	82.5	83.3
Macedonia	3	0.0	0.0	82.5	83.8	84.5	81.7	79.6	58.9
Madagascar	1	2.8	3.5	2.4	2.4	0.0	0.0	0.0	1.6
Malawi	1	0.0	2.5	0.0	0.6	2.5	4.5	0.6	1.5
Malaysia	2	46.9	0.0	0.0	32.1	37.3	42.0	40.2	28.4
Maldives	2	0.0	0.0	0.0	0.0	44.0	46.6	60.1	21.5
Mali	1	4.0	0.0	0.1	0.0	0.0	0.0	0.0	0.6
Malta	5	81.2	86.3	88.7	90.0	90.3	92.8	89.5	88.4
Marshall Islands	0	0.0	0.0	0.0	0.0	0.0	0.0	0.0	0.0
Mauritania	0	0.0	0.0	0.0	0.0	0.0	0.0	0.0	0.0
Mauritius	4	75.2	79.4	78.7	78.2	83.2	85.1	86.3	80.9
Mexico	4	68.3	77.7	78.5	79.7	82.4	84.1	88.1	79.8
Moldova	5	88.8	91.2	81.9	87.8	85.0	83.5	83.7	86.0
Mongolia	2	0.0	0.0	61.2	0.0	19.6	21.4	83.4	26.5
Montenegro	2	0.0	0.0	0.0	0.0	69.2	69.2	0.0	19.8
Morocco	2	0.0	15.4	0.0	0.0	10.2	33.3	13.7	10.4
Mozambique	2	0.0	0.0	0.0	0.1	16.2	63.4	0.0	11.4
Myanmar	1	0.0	0.0	0.0	0.0	0.0	2.9	49.5	7.5
Namibia	0	0.0	0.0	0.0	0.0	0.0	0.0	0.0	0.0
Nepal	1	2.8	2.6	0.0	0.6	0.6	9.2	0.0	2.3

Netherlands	4	88.5	86.7	85.2	83.9	82.3	83.2	83.3	84.7
New Zealand	5	95.3	95.2	95.1	96.8	96.4	96.3	95.7	95.8
Nicaragua	3	0.0	54.3	56.9	69.0	77.2	84.9	90.5	61.8
Niger	1	0.0	0.0	0.0	0.0	0.0	35.7	0.0	5.1
Nigeria	1	0.0	0.0	3.3	0.0	0.0	0.1	32.3	5.1
North Korea	0	0.0	0.0	0.0	0.0	0.0	0.0	0.0	0.0
Northern Mariana Islands	3	0.0	0.0	0.0	75.3	71.4	80.3	82.5	44.2
Norway	5	88.6	89.4	88.6	88.5	86.3	84.2	84.1	87.1
Oman	2	0.0	0.0	0.0	0.0	0.0	73.2	34.4	15.4
Pakistan	1	0.0	2.8	1.6	0.0	1.5	17.8	1.9	3.7
Palestine	2	0.0	0.0	0.0	29.7	32.2	34.2	74.1	24.3
Panama	4	72.3	75.3	0.0	83.5	86.6	86.3	84.0	69.7
Papua New Guinea	1	9.4	3.4	0.0	0.0	0.0	0.0	28.8	6.0
Paraguay	3	51.3	51.2	57.2	62.7	63.6	68.8	74.0	61.3
Peru	3	54.9	36.1	37.2	51.1	63.3	65.4	65.9	53.4
Philippines	4	68.2	71.1	65.9	68.6	74.3	74.1	74.8	71.0
Poland	4	61.8	59.5	59.9	71.6	73.7	73.5	72.6	67.5
Portugal	4	76.9	77.1	76.3	76.2	79.1	77.7	80.1	77.6
Puerto Rico	4	78.6	76.7	83.9	83.2	83.6	83.6	84.4	82.0
Qatar	3	14.7	18.9	0.0	55.1	56.3	64.9	46.1	36.6
Romania	4	76.0	78.0	83.3	84.8	85.9	86.2	85.4	82.8
Russia	5	93.0	87.3	90.7	87.6	88.0	89.3	88.8	89.3
Rwanda	1	0.0	0.0	0.0	0.0	0.0	22.6	0.0	3.2
Saint Lucia	4	66.8	68.2	73.2	72.1	80.0	79.0	85.6	75.0
Saint Vincent and the Grenadines	4	72.3	63.4	61.2	85.3	85.1	87.6	87.3	77.5
Samoa	0	0.0	0.0	0.0	0.0	0.0	0.0	0.0	0.0
São Tomé and Príncipe	1	0.0	69.6	0.0	0.0	0.0	0.0	0.0	9.9
Saudi Arabia	2	0.0	0.0	0.0	23.1	27.5	29.4	28.8	15.5
Senegal	1	2.0	2.5	2.6	2.5	0.0	0.0	0.0	1.4
Serbia	3	0.0	0.0	0.0	66.7	70.8	74.9	69.6	40.3
Seychelles	3	74.4	71.5	0.0	0.0	75.9	76.9	78.1	53.8

Sierra Leone	1	0.0	0.0	3.4	0.0	0.0	0.0	0.0	0.5
Singapore	5	91.2	92.0	94.6	95.1	95.3	94.9	95.7	94.1
Slovakia	3	0.0	0.0	82.7	81.9	84.9	90.2	92.9	61.8
Slovenia	4	0.0	91.6	92.5	91.8	89.3	88.3	88.5	77.4
Solomon Islands	1	0.0	0.0	0.0	0.0	0.0	0.0	34.6	4.9
Somalia	0	0.0	0.0	0.0	0.0	0.0	0.0	0.0	0.0
South Africa	3	0.0	0.0	1.0	67.8	72.0	72.3	70.7	40.5
South Korea	4	0.0	57.8	74.2	75.4	84.5	82.2	81.9	65.1
South Sudan	0	0.0	0.0	0.0	0.0	0.0	0.0	0.0	0.0
Spain	4	77.3	79.4	82.6	83.8	83.1	84.0	84.8	82.1
Sri Lanka	3	52.0	50.2	47.7	55.4	63.7	67.4	64.8	57.3
Sudan	0	0.0	0.0	0.0	0.0	0.0	0.0	0.0	0.0
Suriname	4	60.0	63.7	61.2	66.4	74.3	73.3	73.9	67.5
Swaziland	1	0.0	0.0	0.0	0.0	0.0	0.0	0.0	0.0
Sweden	5	87.4	88.6	88.2	87.1	85.7	85.9	84.8	86.8
Switzerland	4	73.8	74.2	73.4	84.6	84.4	86.7	86.1	80.5
Syria	3	29.5	13.2	0.0	54.3	66.7	74.4	59.8	42.5
Taiwan (province of China)	4	84.3	82.4	79.2	83.4	84.4	84.8	83.7	83.2
Tajikistan	3	74.9	71.4	71.6	58.5	51.4	50.6	50.7	61.3
Tanzania	1	0.0	2.8	1.8	2.1	6.3	2.5	0.0	2.2
Thailand	3	29.9	28.3	35.9	62.6	52.0	61.6	62.3	47.5
The Bahamas	4	76.2	82.6	83.0	89.8	87.7	86.7	86.3	84.6
The Gambia	1	2.8	2.2	2.1	0.9	0.7	1.0	0.0	1.4
Timor-Leste	0	0.0	0.0	0.0	0.0	0.0	0.0	0.0	0.0
Togo	0	0.0	0.0	0.0	0.0	0.0	0.0	0.0	0.0
Tonga	1	0.0	0.0	0.0	0.0	53.6	0.0	0.0	7.7
Trinidad and Tobago	5	79.2	83.4	85.5	89.5	90.7	89.6	90.1	86.9
Tunisia	1	0.0	0.0	0.0	0.0	0.0	28.9	24.8	7.7
Turkey	3	16.8	19.6	20.7	24.0	59.7	70.3	83.9	42.2
Turkmenistan	4	88.4	88.1	83.0	80.9	85.1	81.0	83.4	84.3
Uganda	1	0.0	0.0	0.0	0.0	0.0	2.7	0.0	0.4

Ukraine	5	87.4	90.0	83.5	86.0	86.4	90.9	92.2	88.1
United Arab Emirates	1	0.0	0.0	0.0	0.0	0.0	41.5	0.0	5.9
United Kingdom	5	94.2	94.1	94.1	92.0	91.4	91.5	92.4	92.8
USA	5	88.5	88.1	88.7	88.1	88.0	87.2	86.7	87.9
Uruguay	4	77.3	76.0	79.1	79.1	79.6	78.5	77.5	78.1
Uzbekistan	4	83.1	86.3	85.0	79.0	68.3	72.2	79.8	79.1
Vanuatu	0	0.0	0.0	0.0	0.0	0.0	0.0	0.0	0.0
Venezuela	5	81.3	75.2	83.3	87.8	89.8	89.5	88.9	85.1
Vietnam	1	0.0	0.7	0.2	1.3	0.9	62.0	4.8	10.0
Virgin Islands	4	82.0	0.0	90.6	90.5	84.1	81.9	74.0	71.8
Yemen	0	0.0	0.0	0.0	0.0	0.0	0.0	0.0	0.0
Zambia	1	0.0	0.0	0.0	0.0	0.0	6.3	9.5	2.3
Zimbabwe	2	0.0	0.0	45.3	74.2	0.0	45.3	0.0	23.5

The value of 0 shows lowest quality and 5 denotes highest quality. The percentage of well-certified sources were the proportion of cause of death data that did not have garbage codes at Levels 1 and 2.

Appendix Table 27: Parent cirrhosis CODEm model covariate table

Covariate name	Level	Direction
Alcohol (litres per capita)	1	1
Schistosomiasis prevalence	1	1
Hepatitis B prevalence	1	1
Hepatitis C prevalence	1	1
Hepatitis B3 vaccine (proportion covered)	1	-1
Diabetes prevalence	2	1
BMI (mean)	2	1
Healthcare Access and Quality Index	2	-1
Education (years per capita)	3	-1
Health System Access	3	-1
LDI (per capita)	3	-1
Socio-demographic Index	3	-1

BMI=body mass index. LDI=lag-distributed income. CODEm=cause of death ensemble model

Appendix Figure 1: Trends in age-standardised death rates from 1990 to 2017 for both sexes across seven super-regions

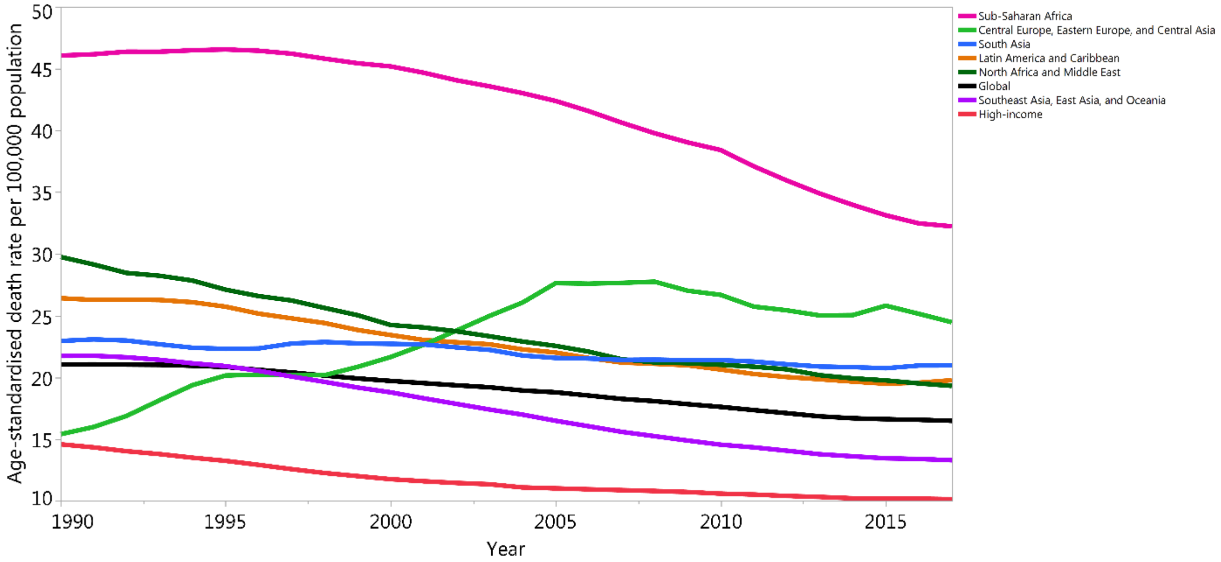

Appendix Figure 2: The trend of age-standardised death rate of cirrhosis due to hepatitis B across regions from 1990 to 2017, versus the levels of SDI. The black line represents expected rates based solely on SDI. For each region, points from left to right depict estimates for each year from 1990 to 2017. SDI=Socio-demographic Index.

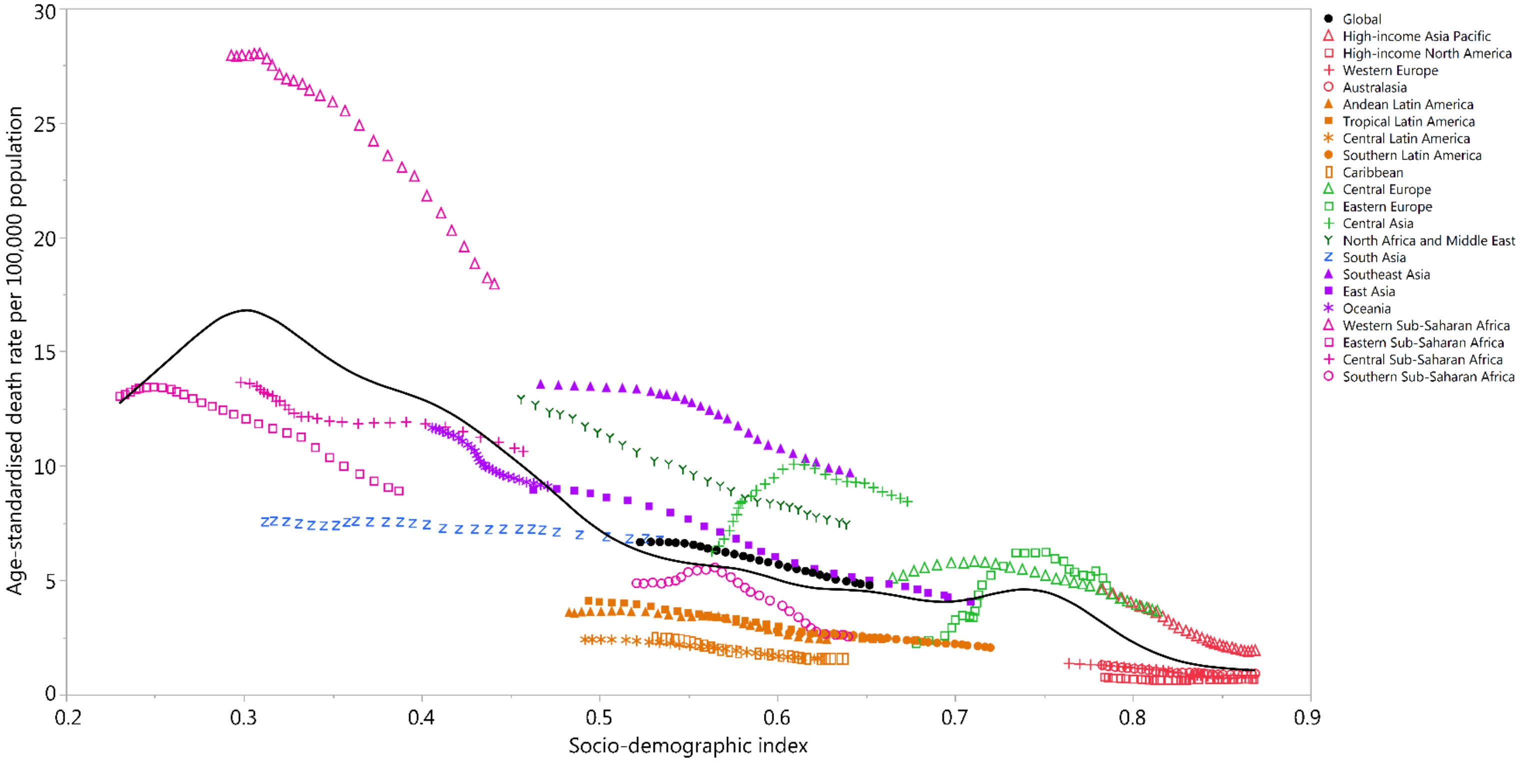

Appendix Figure 3: The trend of age-standardised death rate of cirrhosis due to hepatitis C across regions from 1990 to 2017, versus the levels of SDI. The black line represents expected rates based solely on SDI. For each region, points from left to right depict estimates for each year from 1990 to 2017. SDI=Socio-demographic Index.

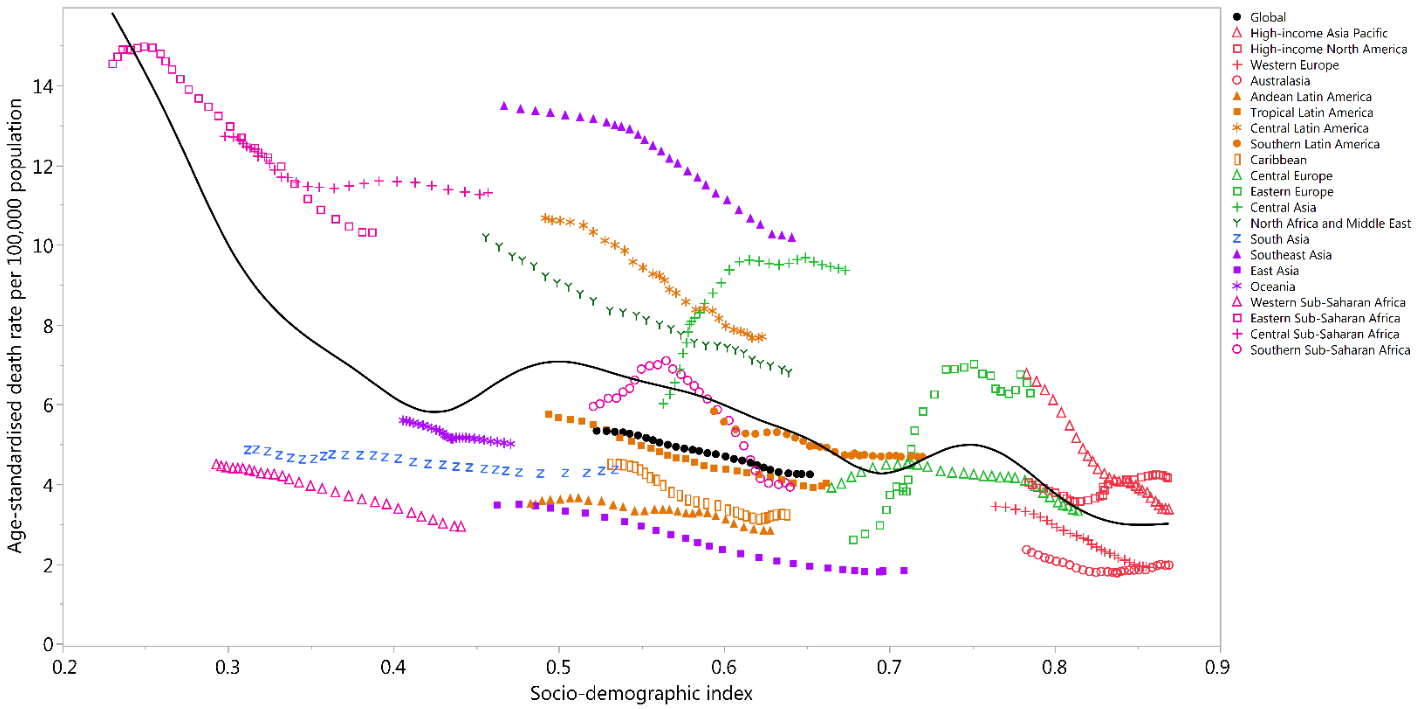

Appendix Figure 4: The trend of age-standardised death rate of cirrhosis due to alcohol-related liver disease across regions from 1990 to 2017, versus the levels of SDI. The black line represents expected rates based solely on SDI. For each region, points from left to right depict estimates for each year from 1990 to 2017. SDI=Socio-demographic Index.

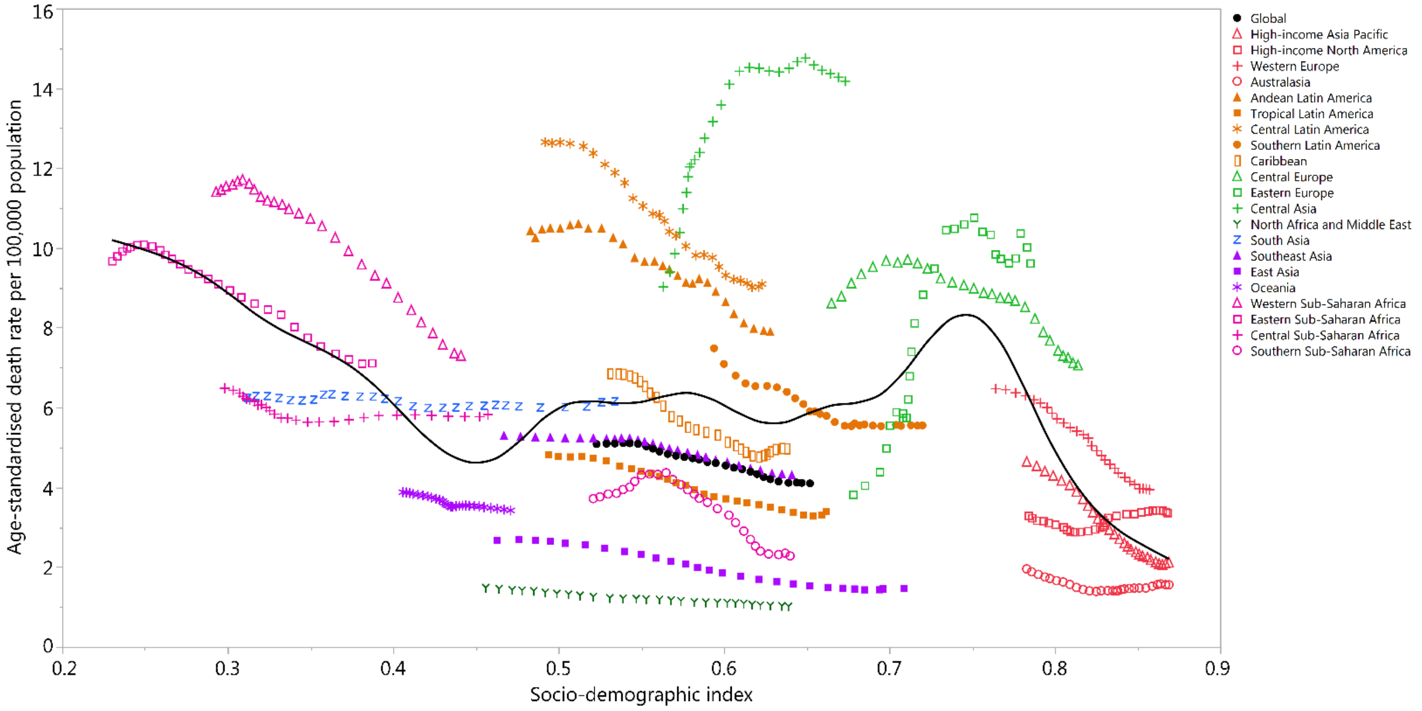

Appendix Figure 5: The trend of age-standardised death rate of cirrhosis due to NASH across regions from 1990 to 2017, versus the levels of SDI. The black line represents expected rates based solely on SDI. For each region, points from left to right depict estimates for each year from 1990 to 2017. NASH=non-alcoholic steatohepatitis. SDI=Socio-demographic Index.

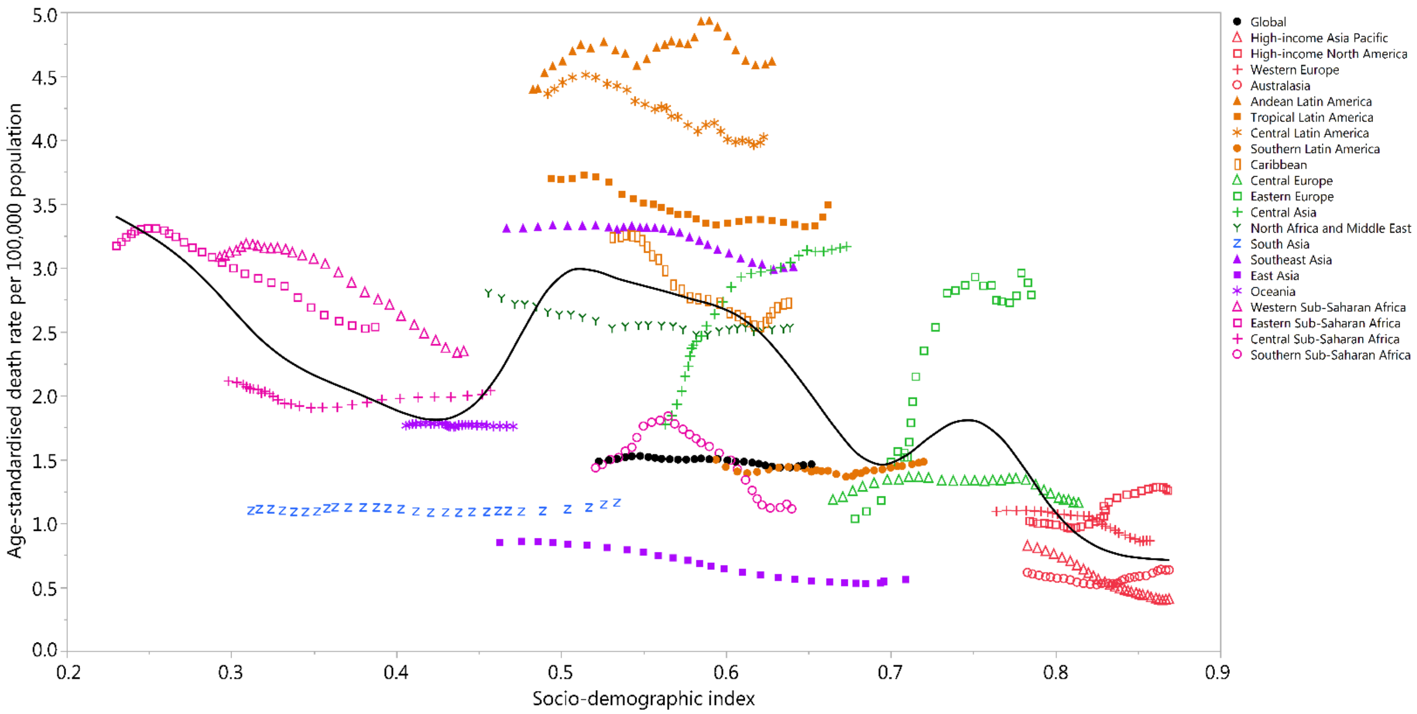

Appendix Figure 6: The trend of age-standardised death rate of cirrhosis due to other causes across regions from 1990 to 2017, versus the levels of SDI. The black line represents expected rates based solely on SDI. For each region, points from left to right depict estimates for each year from 1990 to 2017. SDI=Socio-demographic Index.

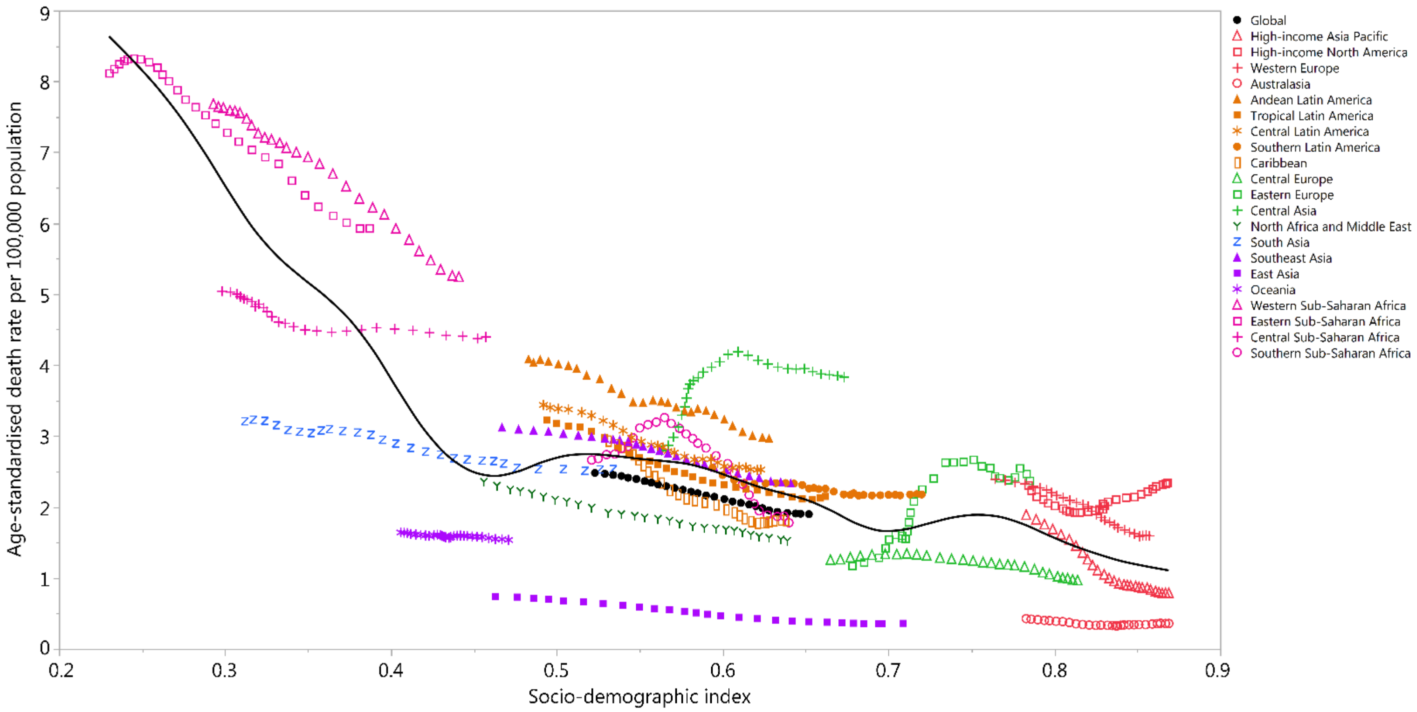

Appendix Figure 7: The counts and age-standardised rates of mortality by cause across age groups in 2017

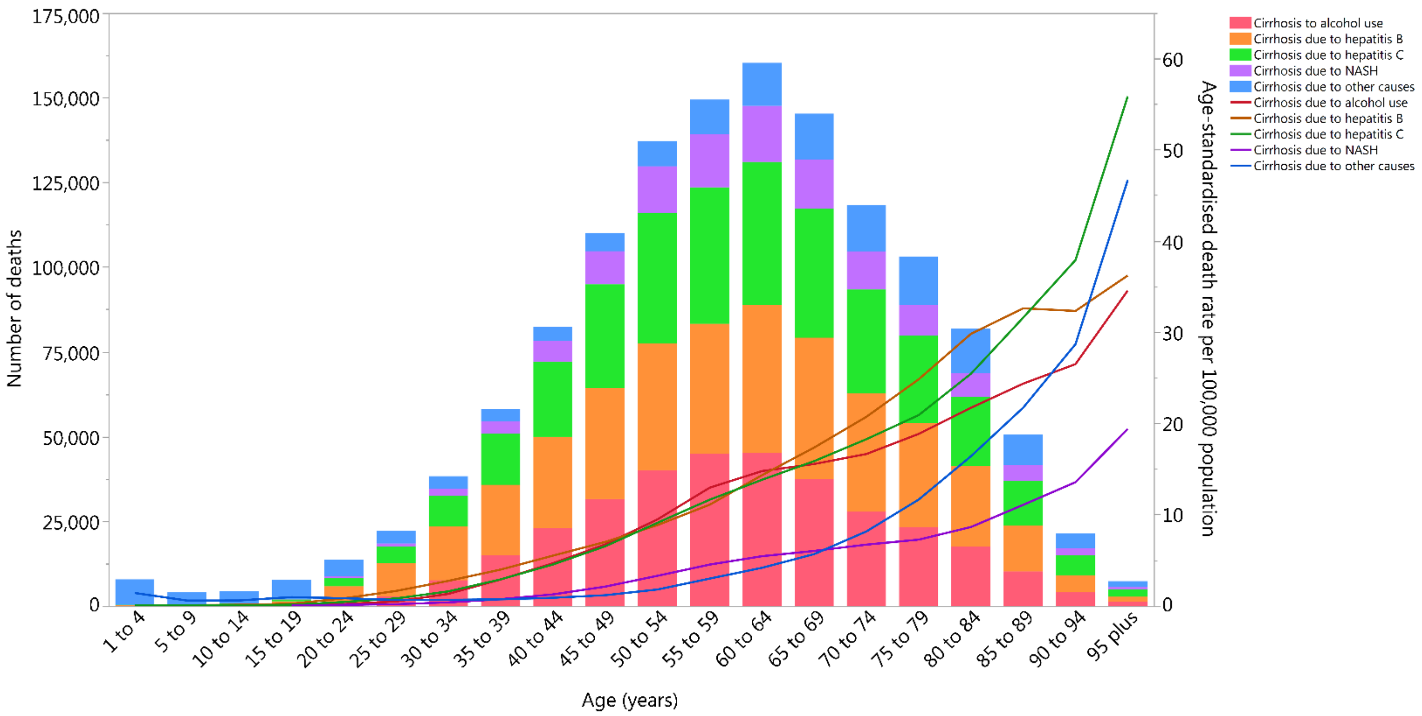

Appendix Figure 8: The proportion of YLLs and YLDs out of DALYs by age in 2017. YLLs=years of life lost due to premature death. YLDs=years of life lost due to disability. DALYs=disability-adjusted life-years.

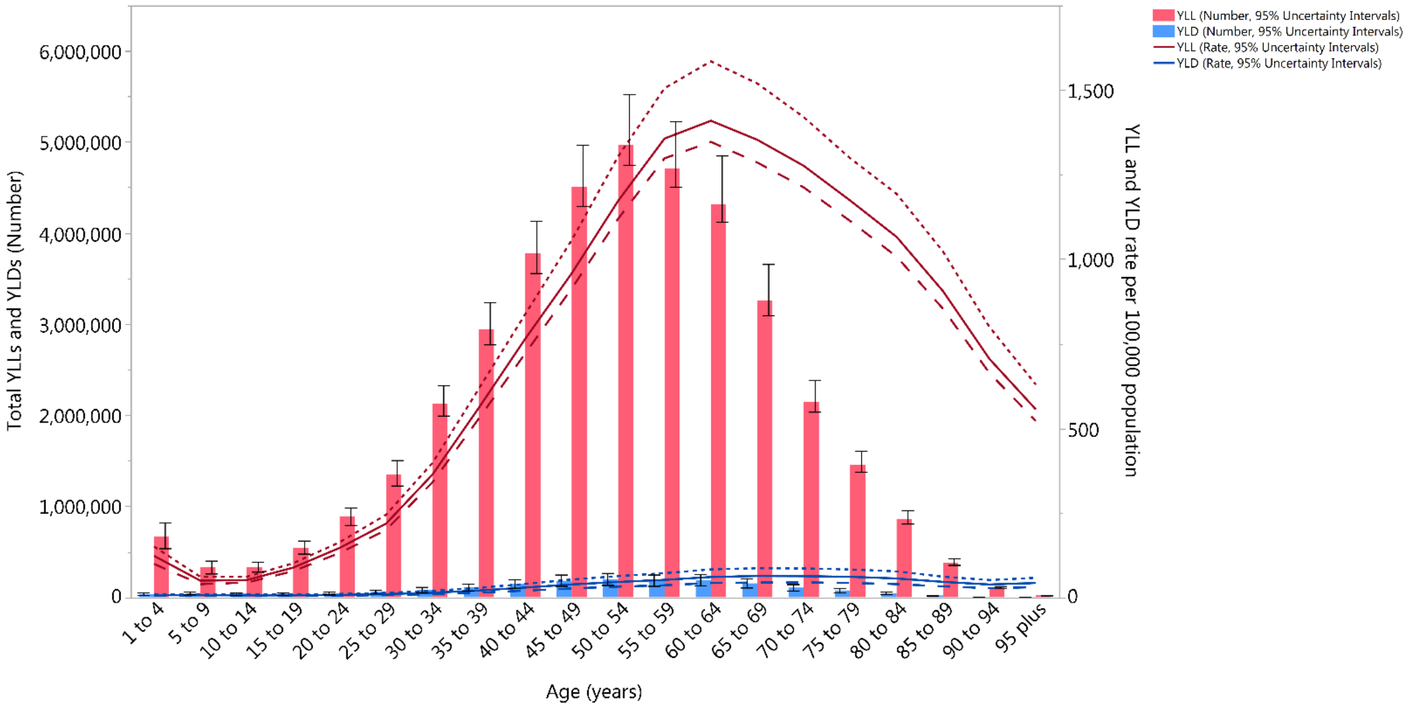

Appendix Figure 9: The age pattern of numbers and rates of death at regional level in 1990, 2000, 2010, and 2017.

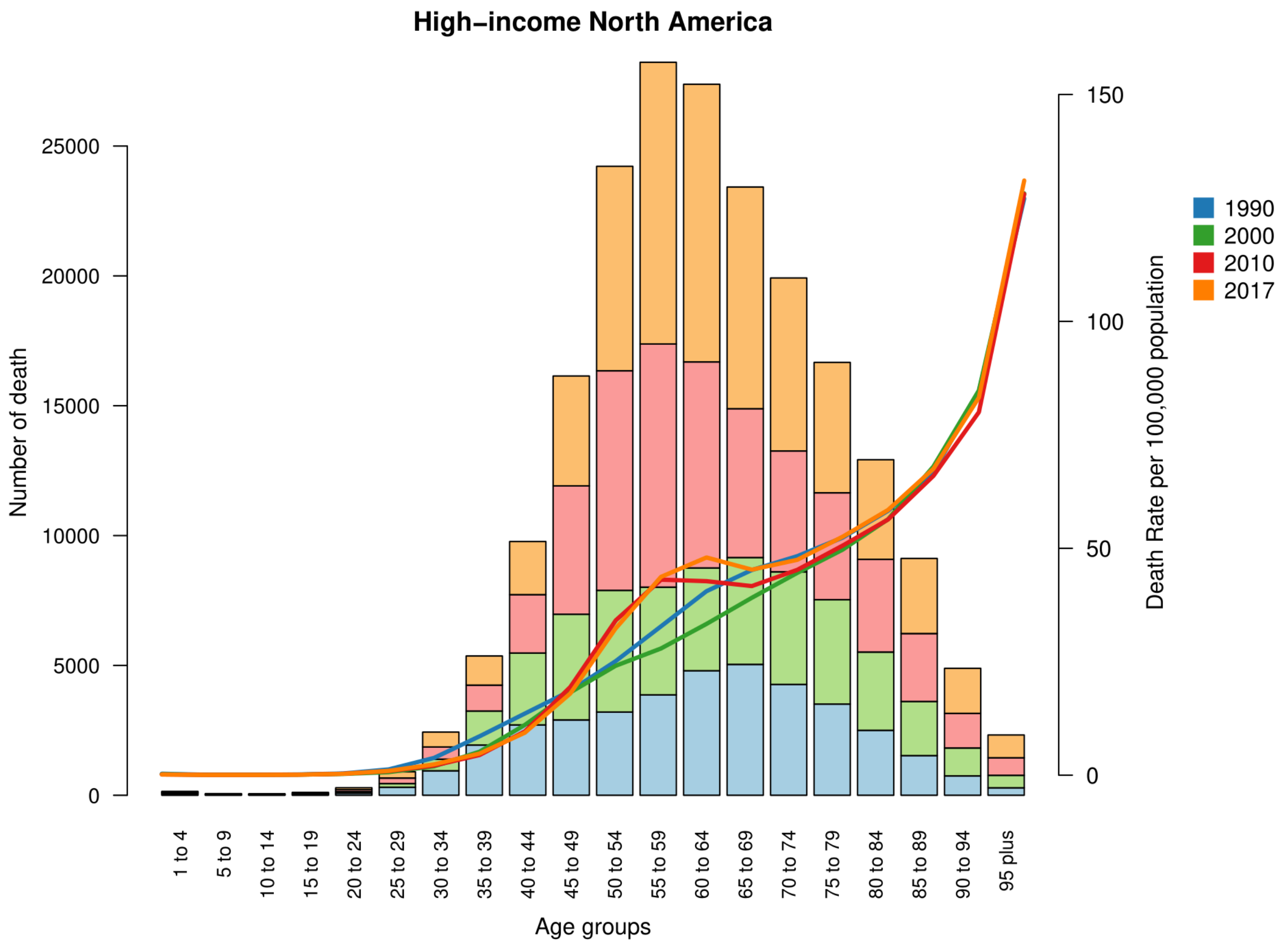

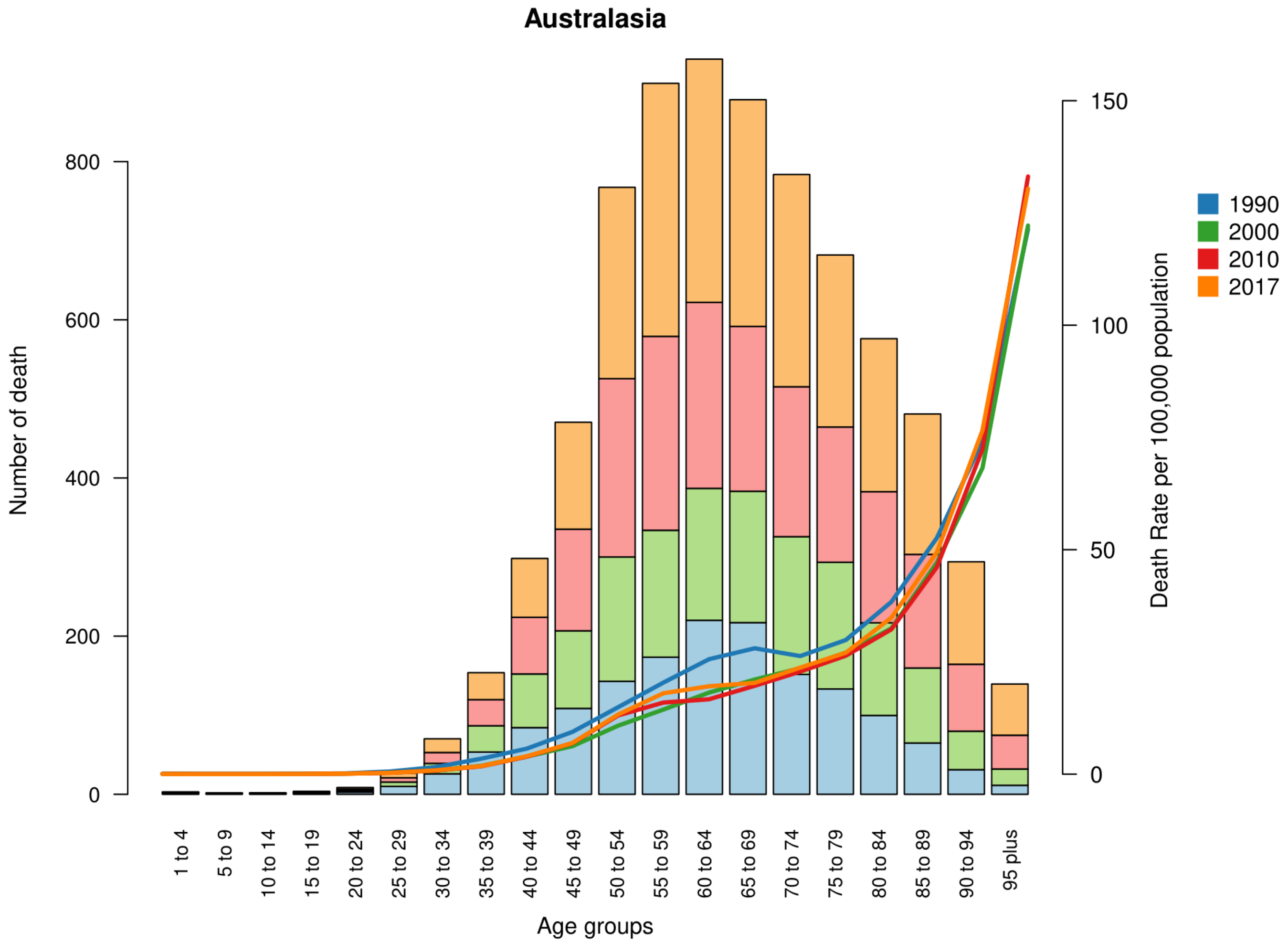

Australasia
Number of death
800
600
400
200
0
150
100
50
0
Death Rate per 100,000 population
1990
2000
2010
2017
1 to 4
5 to 9
10 to 14
15 to 19
20 to 24
25 to 29
30 to 34
35 to 39
40 to 44
45 to 49
50 to 54
55 to 59
60 to 64
65 to 69
70 to 74
75 to 79
80 to 84
85 to 89
90 to 94
95 plus
Age groups

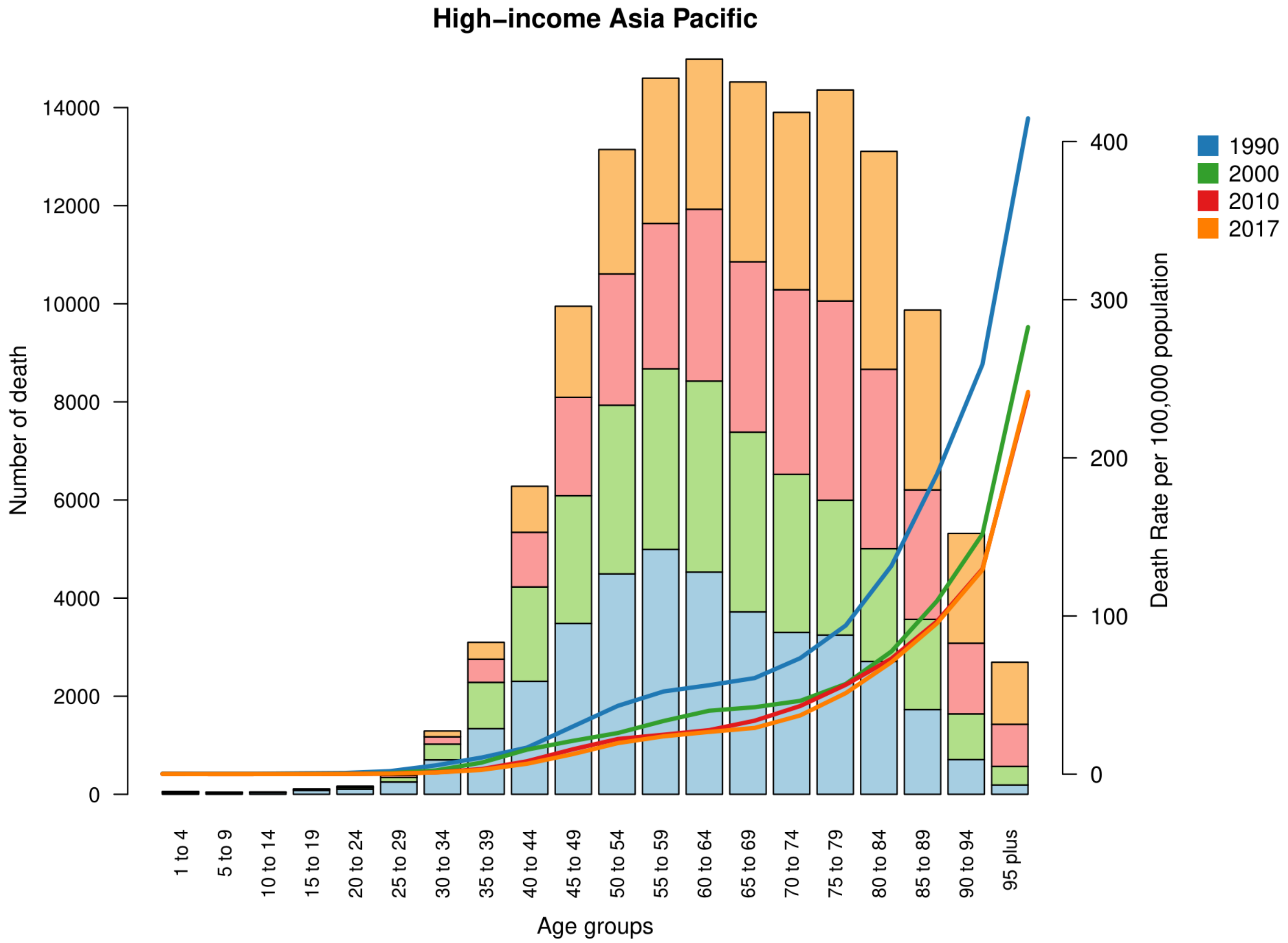

High−income Asia Pacific
Number of death
0
2000
4000
6000
8000
10000
12000
14000
Death Rate per 100,000 population
0
100
200
300
400
1990
2000
2010
2017
1 to 4
5 to 9
10 to 14
15 to 19
20 to 24
25 to 29
30 to 34
35 to 39
40 to 44
45 to 49
50 to 54
55 to 59
60 to 64
65 to 69
70 to 74
75 to 79
80 to 84
85 to 89
90 to 94
95 plus
Age groups

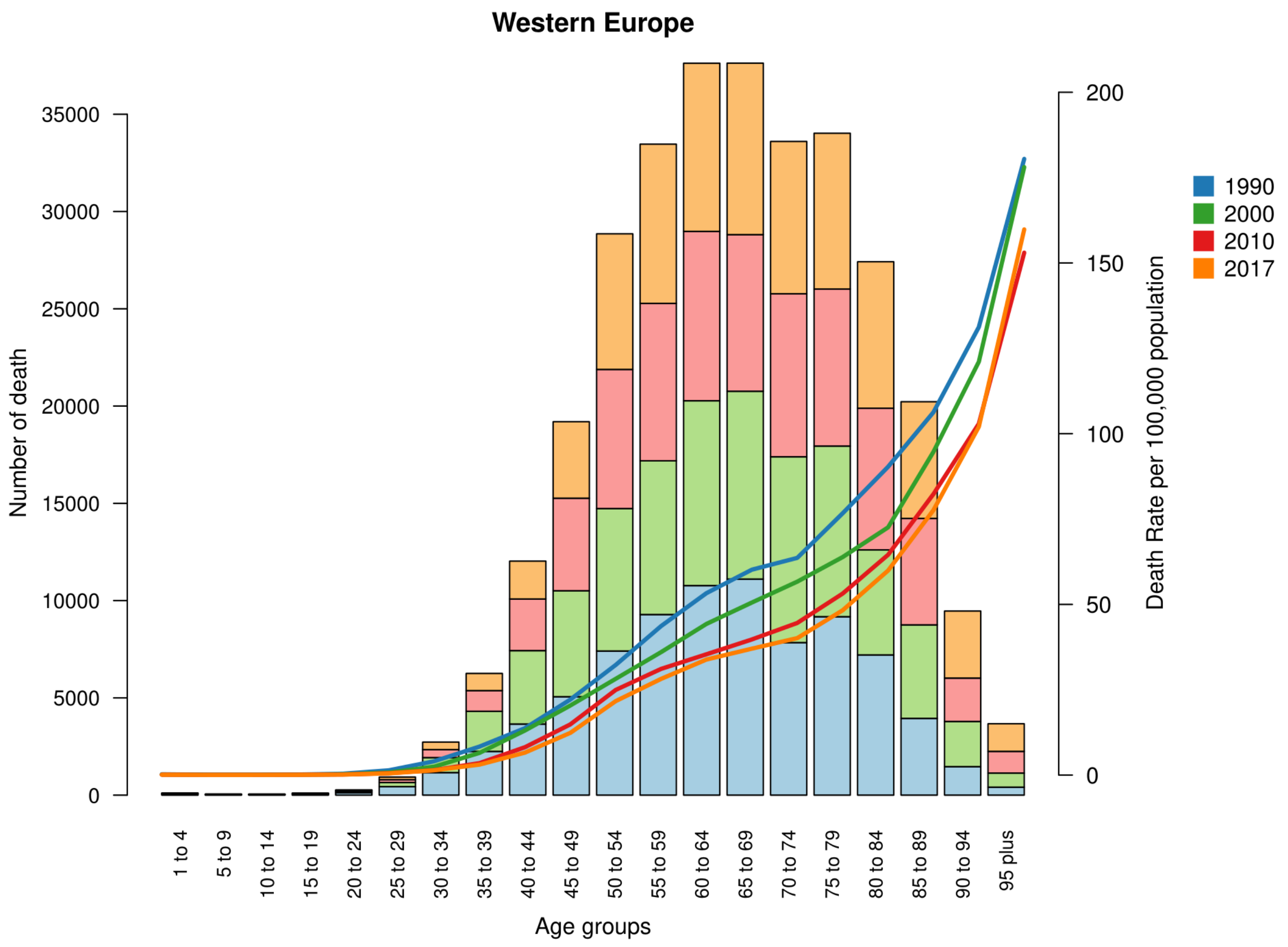

Western Europe
Number of death
35000
30000
25000
20000
15000
10000
5000
0
200
150
100
50
0
Death Rate per 100,000 population
1990
2000
2010
2017
1 to 4
5 to 9
10 to 14
15 to 19
20 to 24
25 to 29
30 to 34
35 to 39
40 to 44
45 to 49
50 to 54
55 to 59
60 to 64
65 to 69
70 to 74
75 to 79
80 to 84
85 to 89
90 to 94
95 plus
Age groups

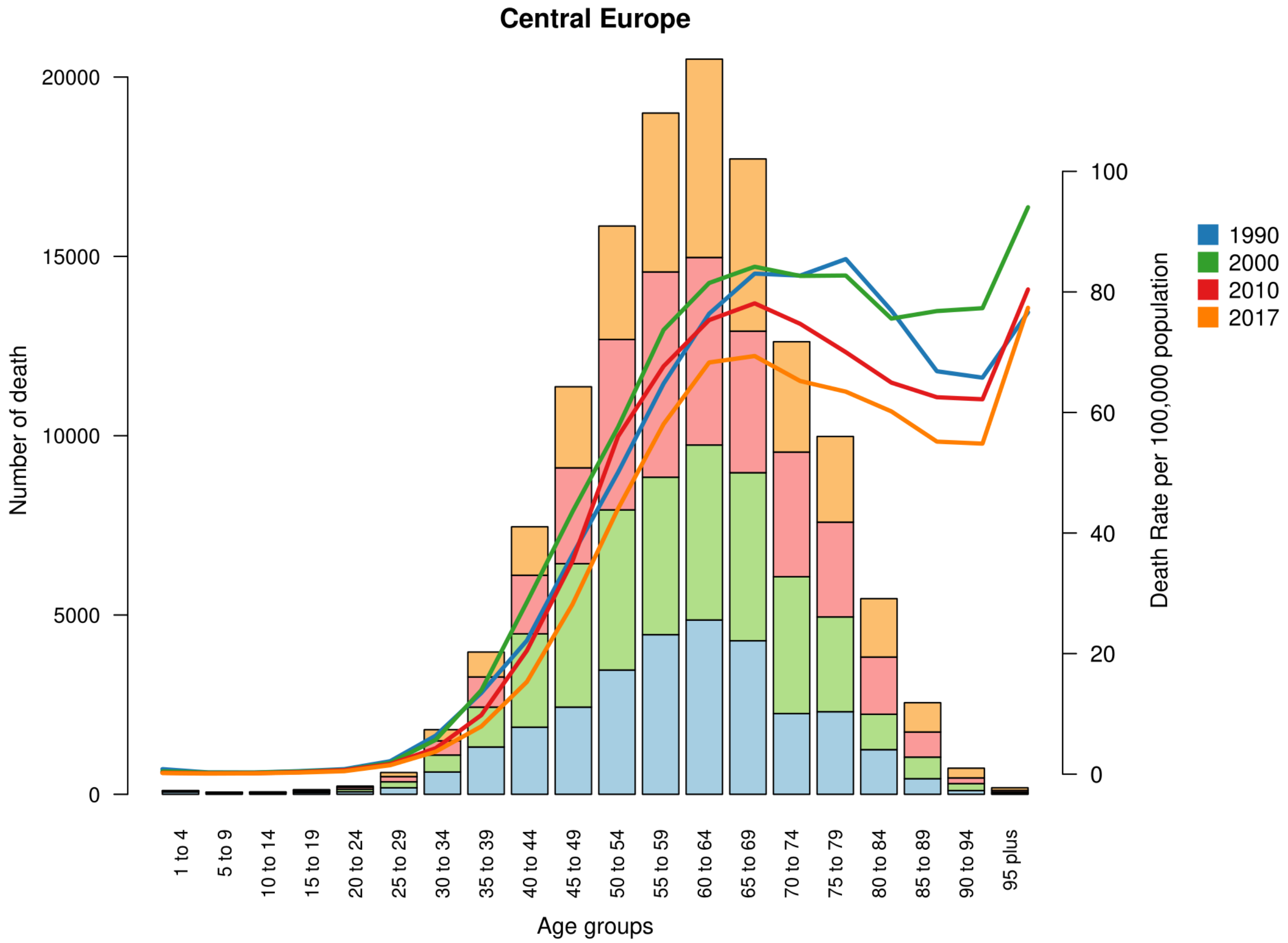
Central Europe
Number of death
20000
15000
10000
5000
0
Death Rate per 100,000 population
100
80
60
40
20
0
1990
2000
2010
2017
1 to 4
5 to 9
10 to 14
15 to 19
20 to 24
25 to 29
30 to 34
35 to 39
40 to 44
45 to 49
50 to 54
55 to 59
60 to 64
65 to 69
70 to 74
75 to 79
80 to 84
85 to 89
90 to 94
95 plus
Age groups

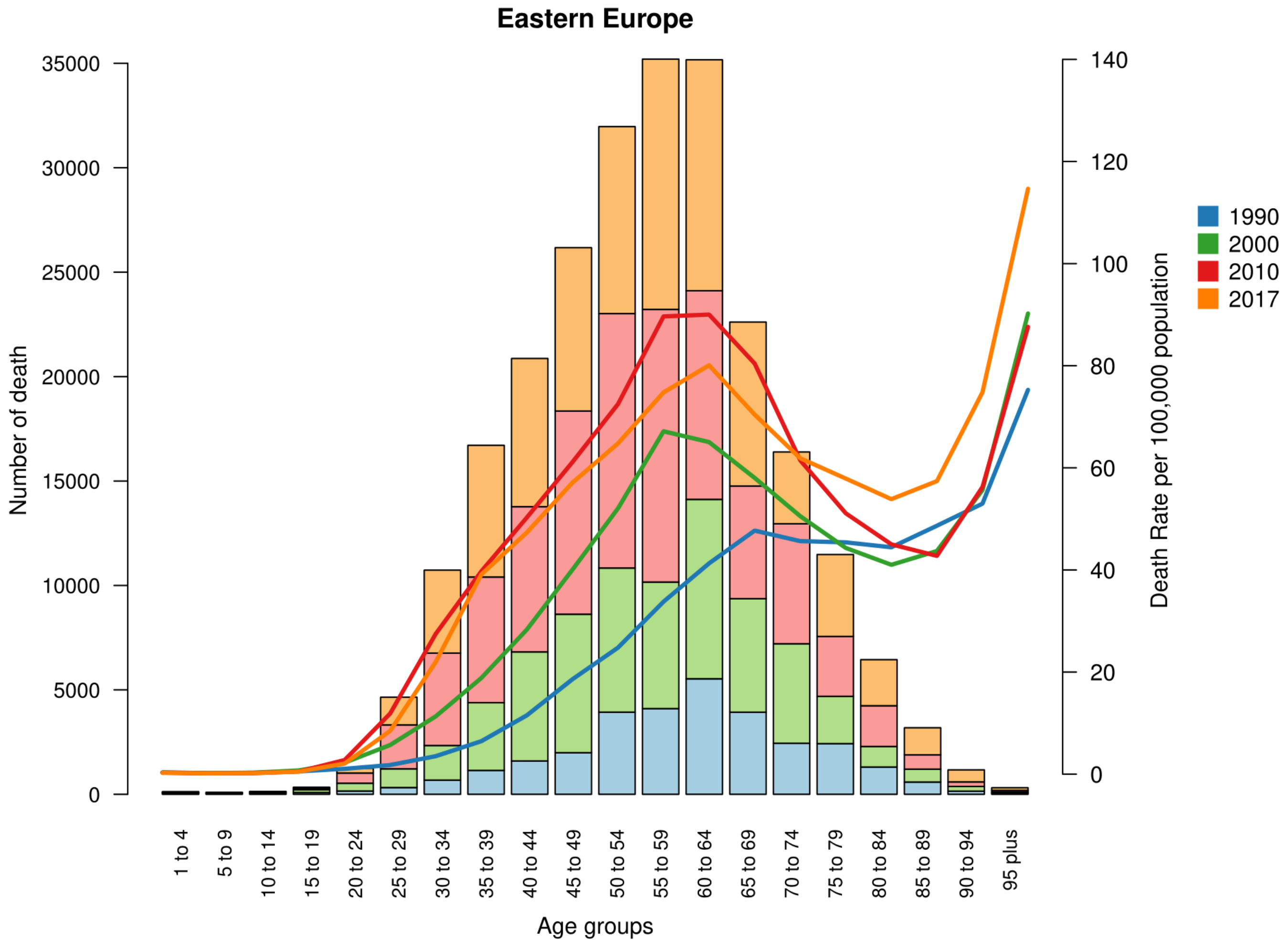

Eastern Europe
Number of death
Death Rate per 100,000 population
Age groups
1990
2000
2010
2017
0
5000
10000
15000
20000
25000
30000
35000
0
20
40
60
80
100
120
140
1 to 4
5 to 9
10 to 14
15 to 19
20 to 24
25 to 29
30 to 34
35 to 39
40 to 44
45 to 49
50 to 54
55 to 59
60 to 64
65 to 69
70 to 74
75 to 79
80 to 84
85 to 89
90 to 94
95 plus

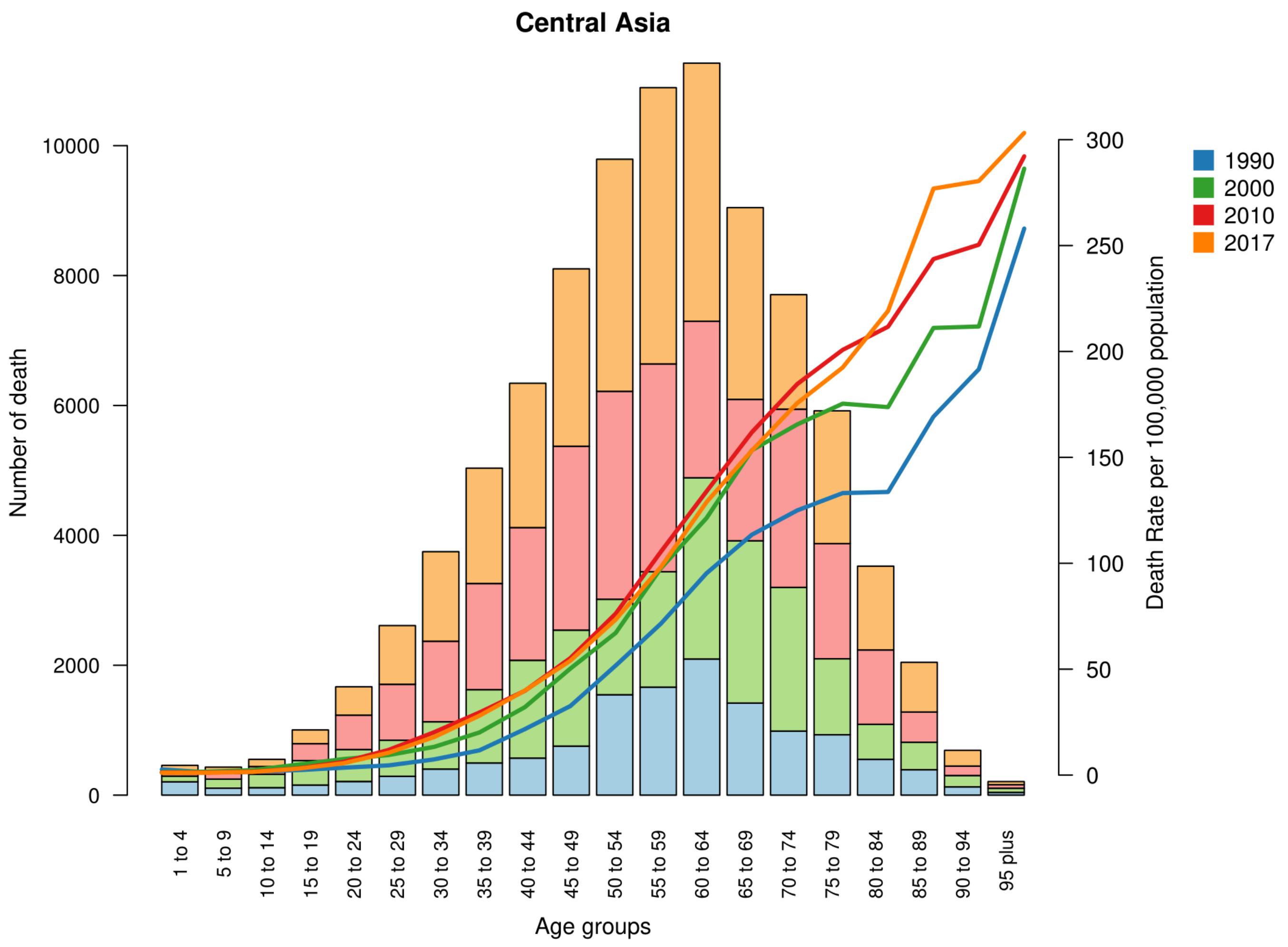
Central Asia
Number of death
10000
8000
6000
4000
2000
0
300
250
200
150
100
50
0
Death Rate per 100,000 population
1990
2000
2010
2017
1 to 4
5 to 9
10 to 14
15 to 19
20 to 24
25 to 29
30 to 34
35 to 39
40 to 44
45 to 49
50 to 54
55 to 59
60 to 64
65 to 69
70 to 74
75 to 79
80 to 84
85 to 89
90 to 94
95 plus
Age groups

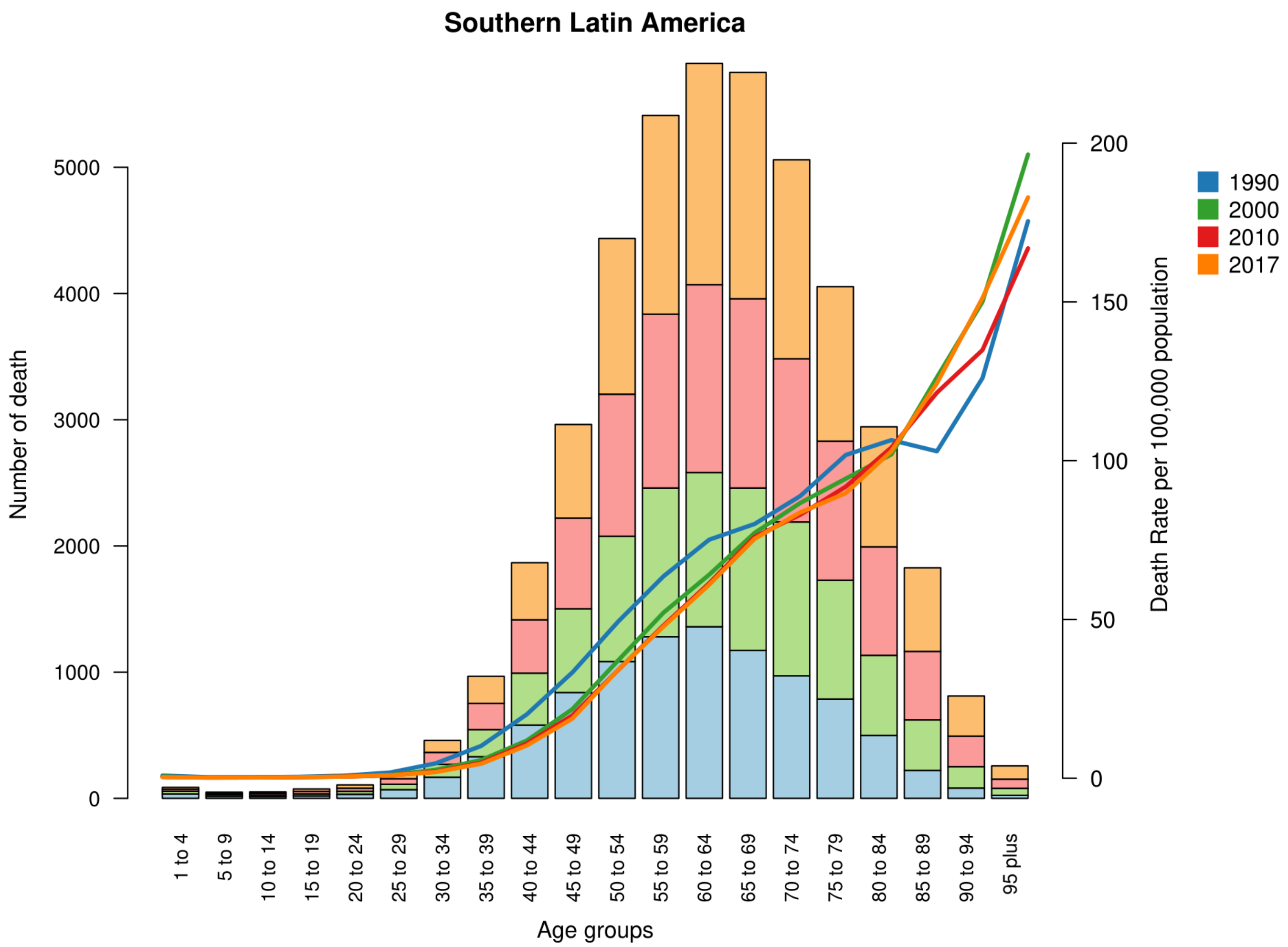
Southern Latin America
Number of death
0
1000
2000
3000
4000
5000
Death Rate per 100,000 population
0
50
100
150
200
1990
2000
2010
2017
1 to 4
5 to 9
10 to 14
15 to 19
20 to 24
25 to 29
30 to 34
35 to 39
40 to 44
45 to 49
50 to 54
55 to 59
60 to 64
65 to 69
70 to 74
75 to 79
80 to 84
85 to 89
90 to 94
95 plus
Age groups

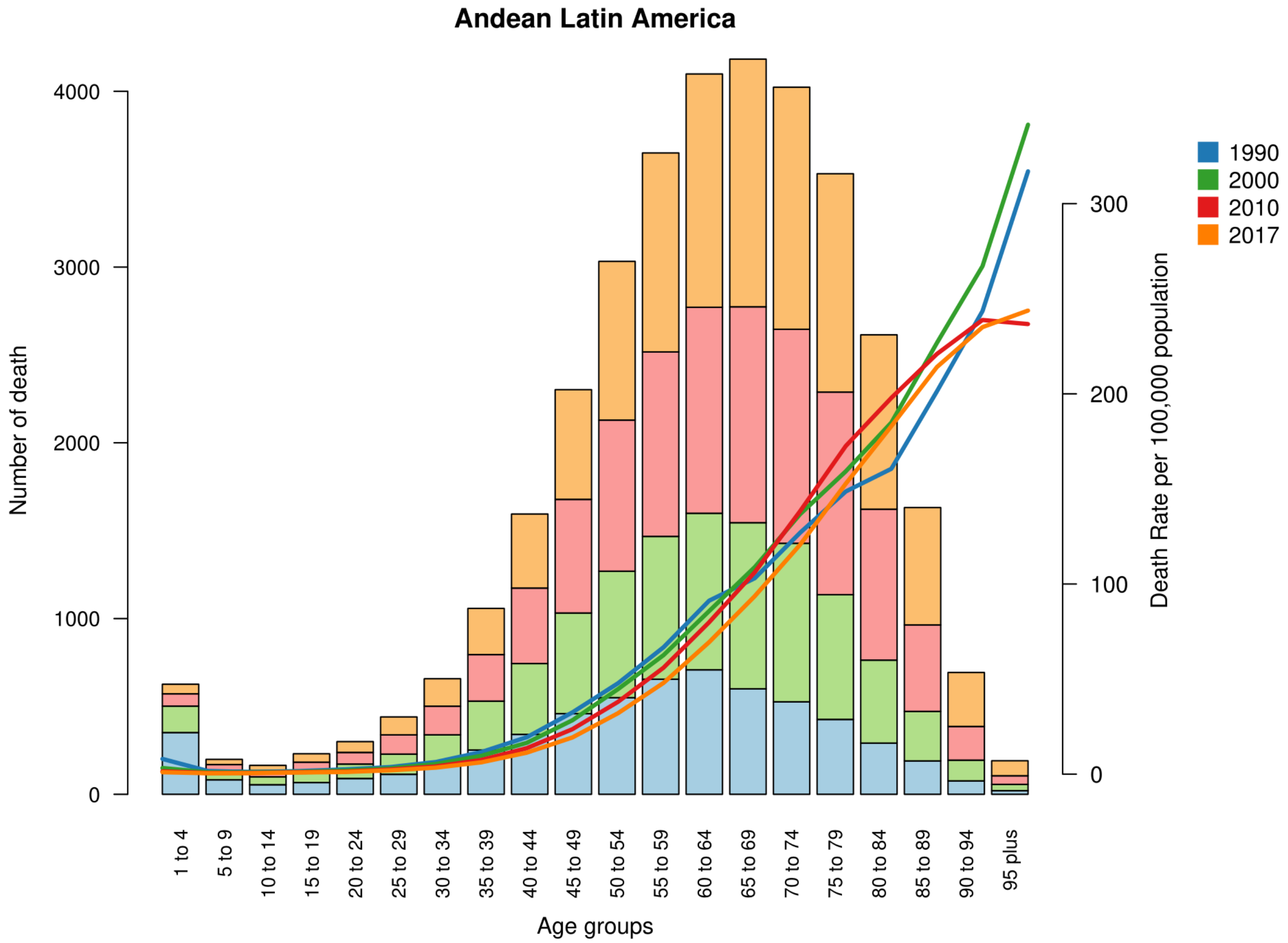

Andean Latin America
Number of death
Death Rate per 100,000 population
Age groups
1990
2000
2010
2017
0
1000
2000
3000
4000
0
100
200
300
1 to 4
5 to 9
10 to 14
15 to 19
20 to 24
25 to 29
30 to 34
35 to 39
40 to 44
45 to 49
50 to 54
55 to 59
60 to 64
65 to 69
70 to 74
75 to 79
80 to 84
85 to 89
90 to 94
95 plus

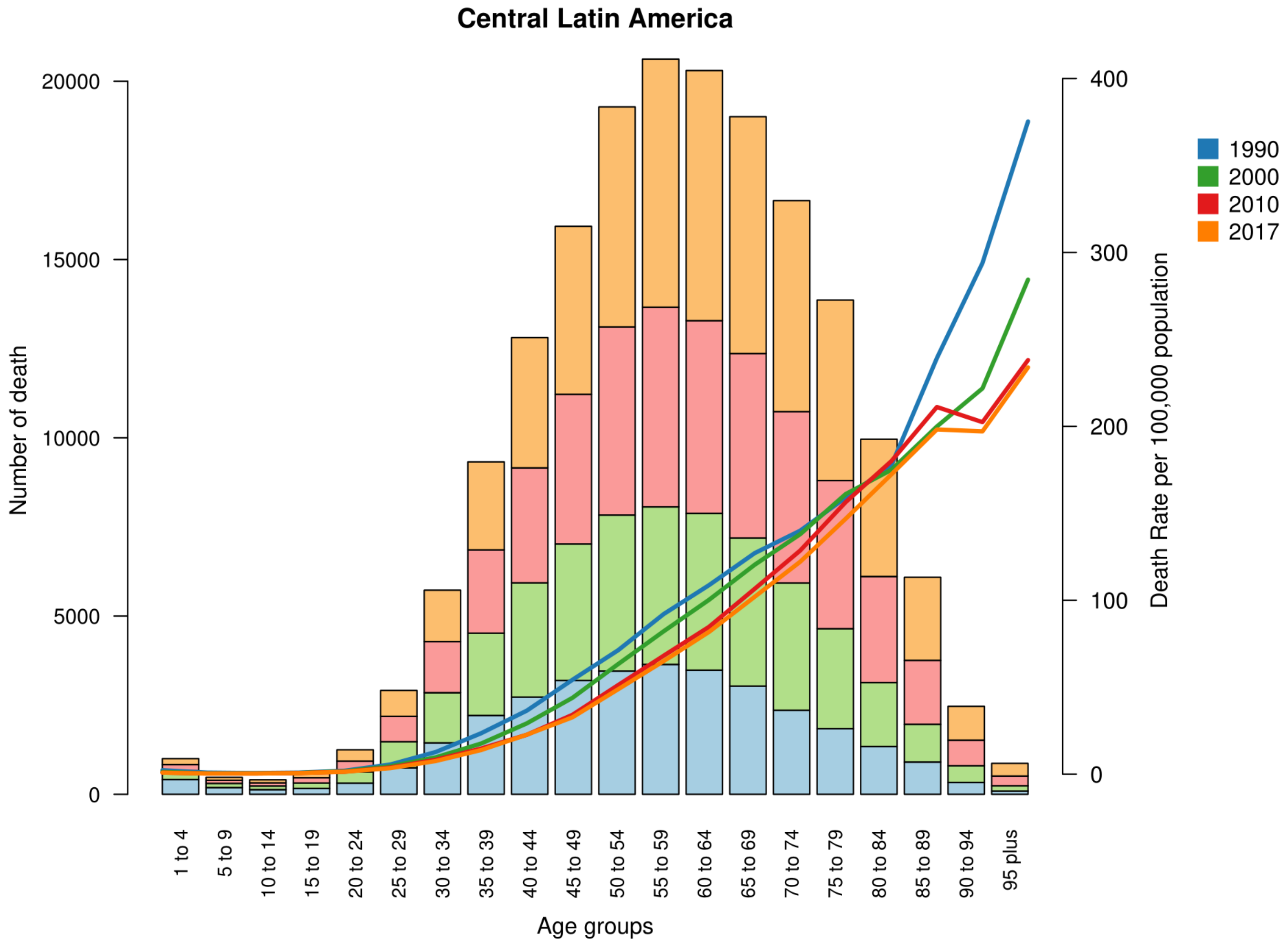
Central Latin America
Number of death
20000
15000
10000
5000
0
Death Rate per 100,000 population
400
300
200
100
0
1990
2000
2010
2017
1 to 4
5 to 9
10 to 14
15 to 19
20 to 24
25 to 29
30 to 34
35 to 39
40 to 44
45 to 49
50 to 54
55 to 59
60 to 64
65 to 69
70 to 74
75 to 79
80 to 84
85 to 89
90 to 94
95 plus
Age groups

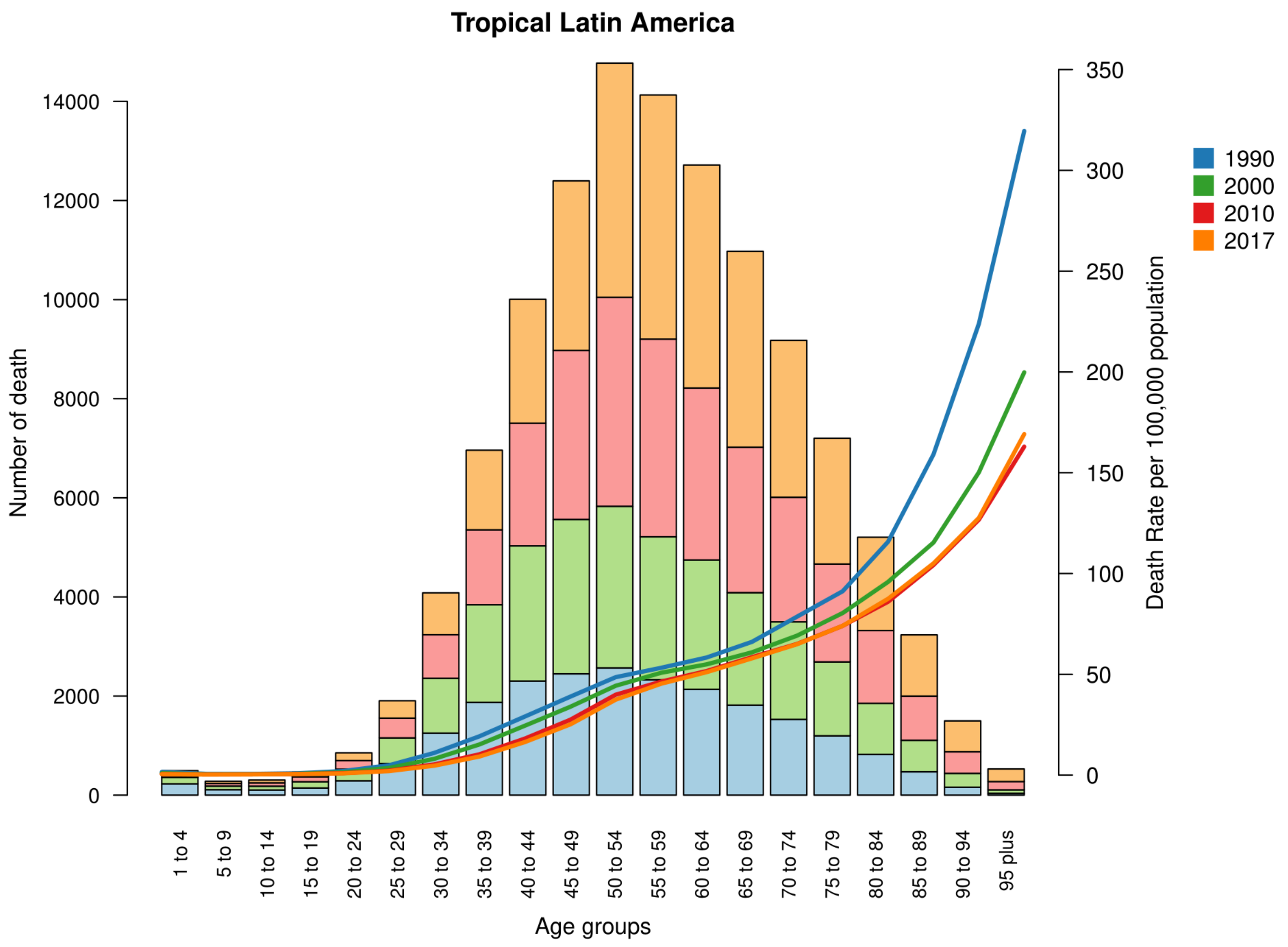

Tropical Latin America
Number of death
14000
12000
10000
8000
6000
4000
2000
0
Death Rate per 100,000 population
350
300
250
200
150
100
50
0
1990
2000
2010
2017
1 to 4
5 to 9
10 to 14
15 to 19
20 to 24
25 to 29
30 to 34
35 to 39
40 to 44
45 to 49
50 to 54
55 to 59
60 to 64
65 to 69
70 to 74
75 to 79
80 to 84
85 to 89
90 to 94
95 plus
Age groups

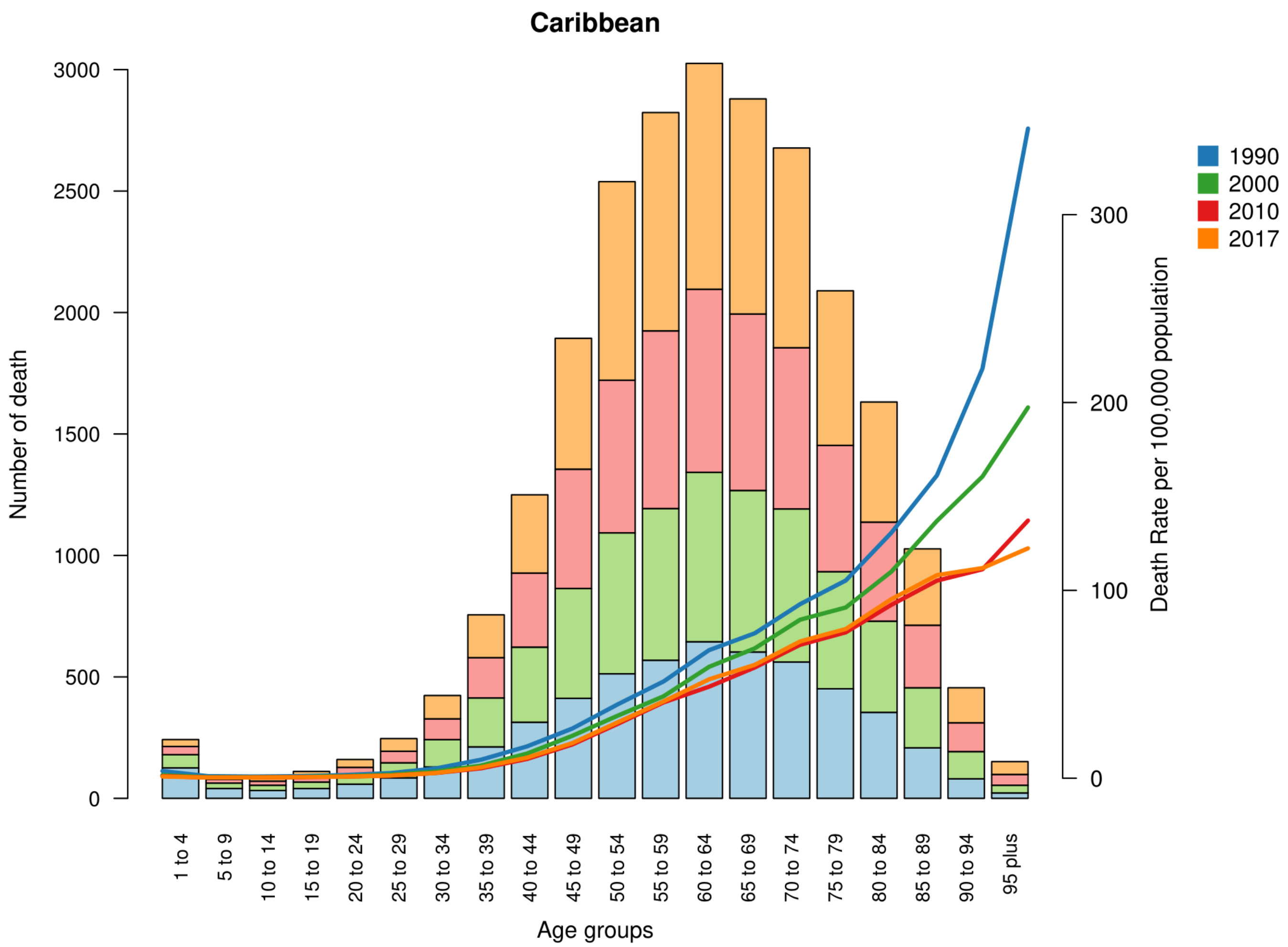
Caribbean
Number of death
Death Rate per 100,000 population
Age groups
1990
2000
2010
2017
0
500
1000
1500
2000
2500
3000
0
100
200
300
1 to 4
5 to 9
10 to 14
15 to 19
20 to 24
25 to 29
30 to 34
35 to 39
40 to 44
45 to 49
50 to 54
55 to 59
60 to 64
65 to 69
70 to 74
75 to 79
80 to 84
85 to 89
90 to 94
95 plus

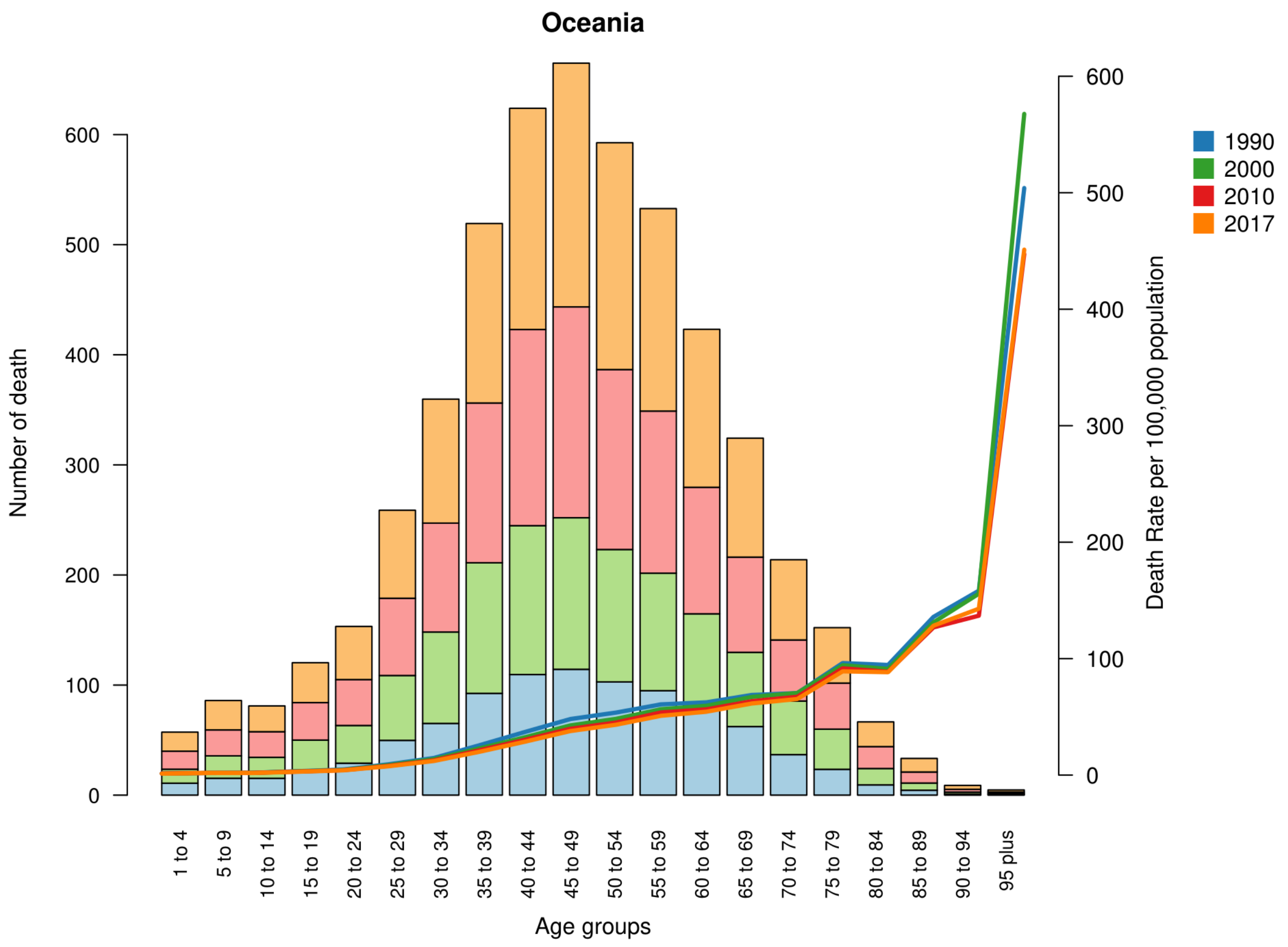
Oceania
Number of death
Death Rate per 100,000 population
Age groups
1990
2000
2010
2017
0
100
200
300
400
500
600
1 to 4
5 to 9
10 to 14
15 to 19
20 to 24
25 to 29
30 to 34
35 to 39
40 to 44
45 to 49
50 to 54
55 to 59
60 to 64
65 to 69
70 to 74
75 to 79
80 to 84
85 to 89
90 to 94
95 plus

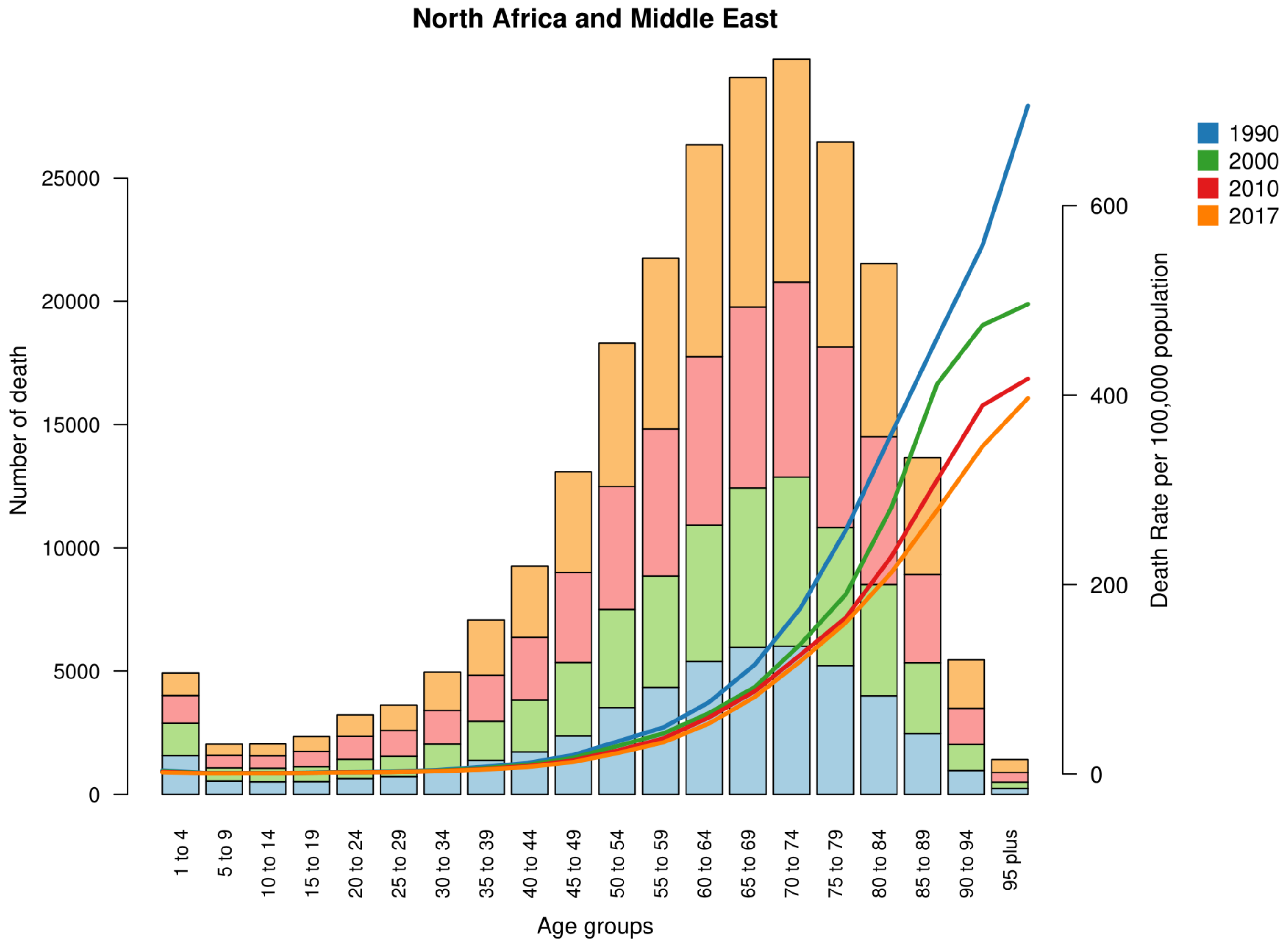
North Africa and Middle East
Number of death
25000
20000
15000
10000
5000
0
Death Rate per 100,000 population
600
400
200
0
1990
2000
2010
2017
1 to 4
5 to 9
10 to 14
15 to 19
20 to 24
25 to 29
30 to 34
35 to 39
40 to 44
45 to 49
50 to 54
55 to 59
60 to 64
65 to 69
70 to 74
75 to 79
80 to 84
85 to 89
90 to 94
95 plus
Age groups

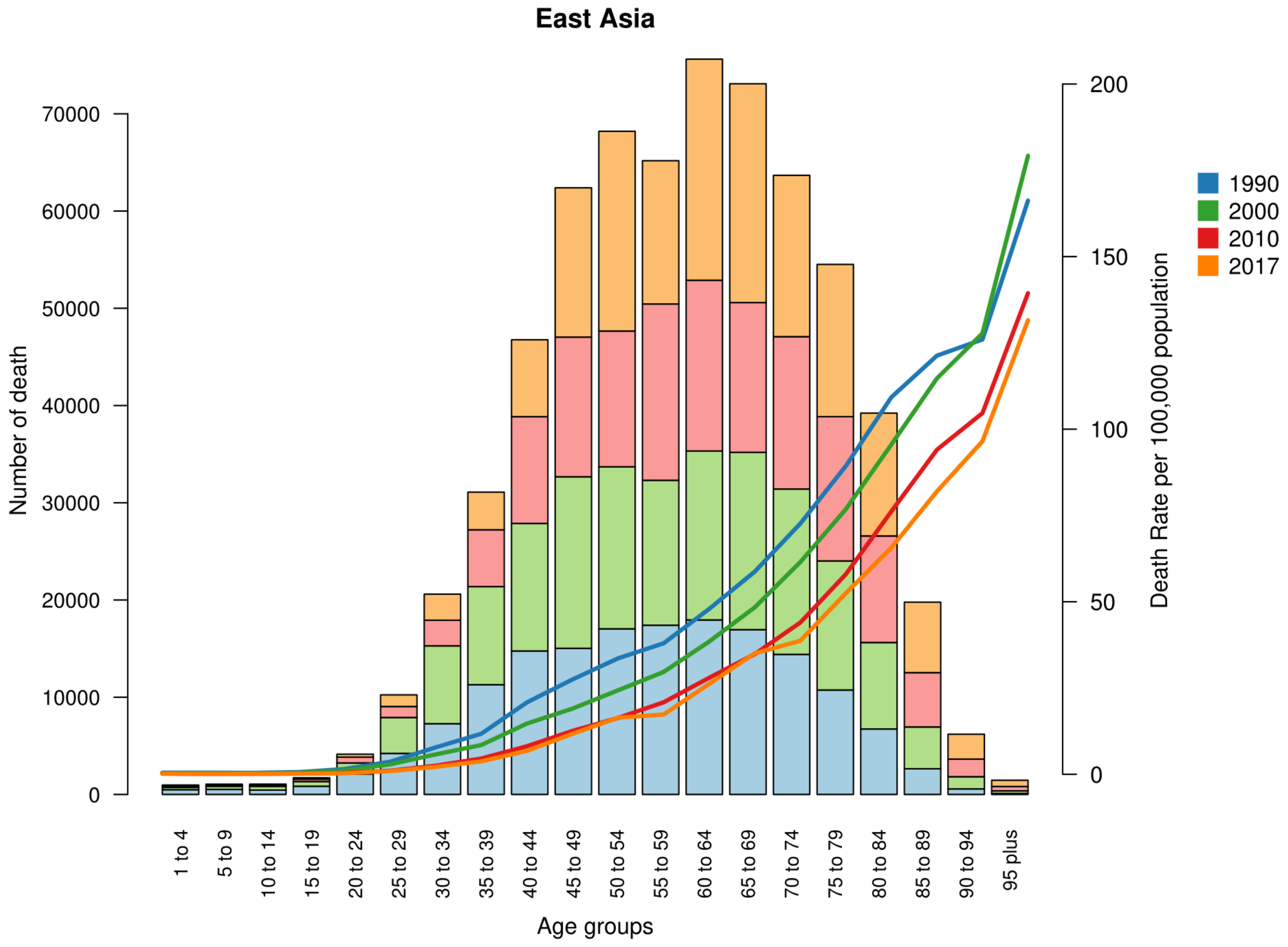

East Asia
Number of death
70000
60000
50000
40000
30000
20000
10000
0
200
150
100
50
0
Death Rate per 100,000 population
1990
2000
2010
2017
1 to 4
5 to 9
10 to 14
15 to 19
20 to 24
25 to 29
30 to 34
35 to 39
40 to 44
45 to 49
50 to 54
55 to 59
60 to 64
65 to 69
70 to 74
75 to 79
80 to 84
85 to 89
90 to 94
95 plus
Age groups

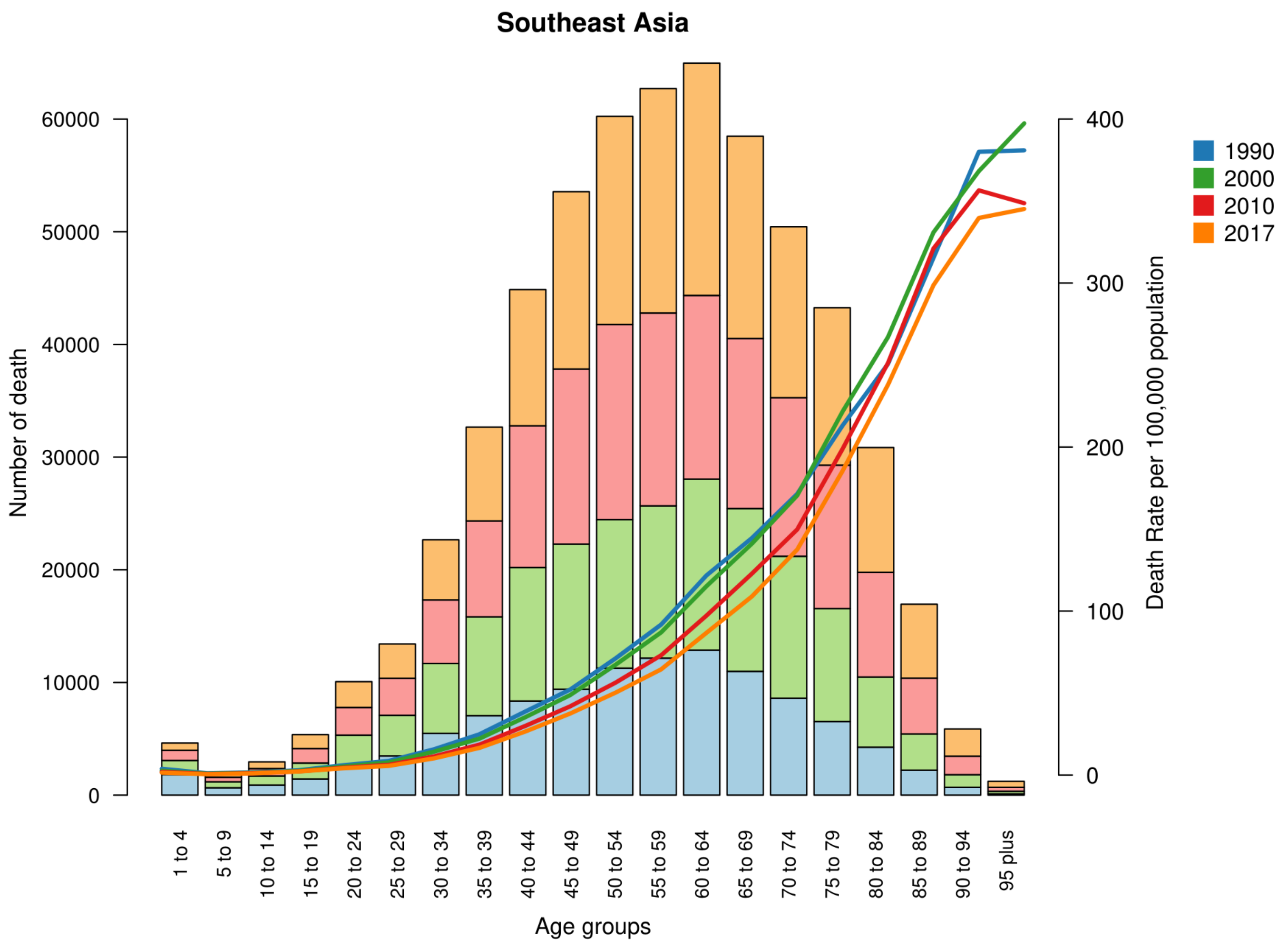
Southeast Asia
Number of death
60000
50000
40000
30000
20000
10000
0
Death Rate per 100,000 population
400
300
200
100
0
1990
2000
2010
2017
1 to 4
5 to 9
10 to 14
15 to 19
20 to 24
25 to 29
30 to 34
35 to 39
40 to 44
45 to 49
50 to 54
55 to 59
60 to 64
65 to 69
70 to 74
75 to 79
80 to 84
85 to 89
90 to 94
95 plus
Age groups

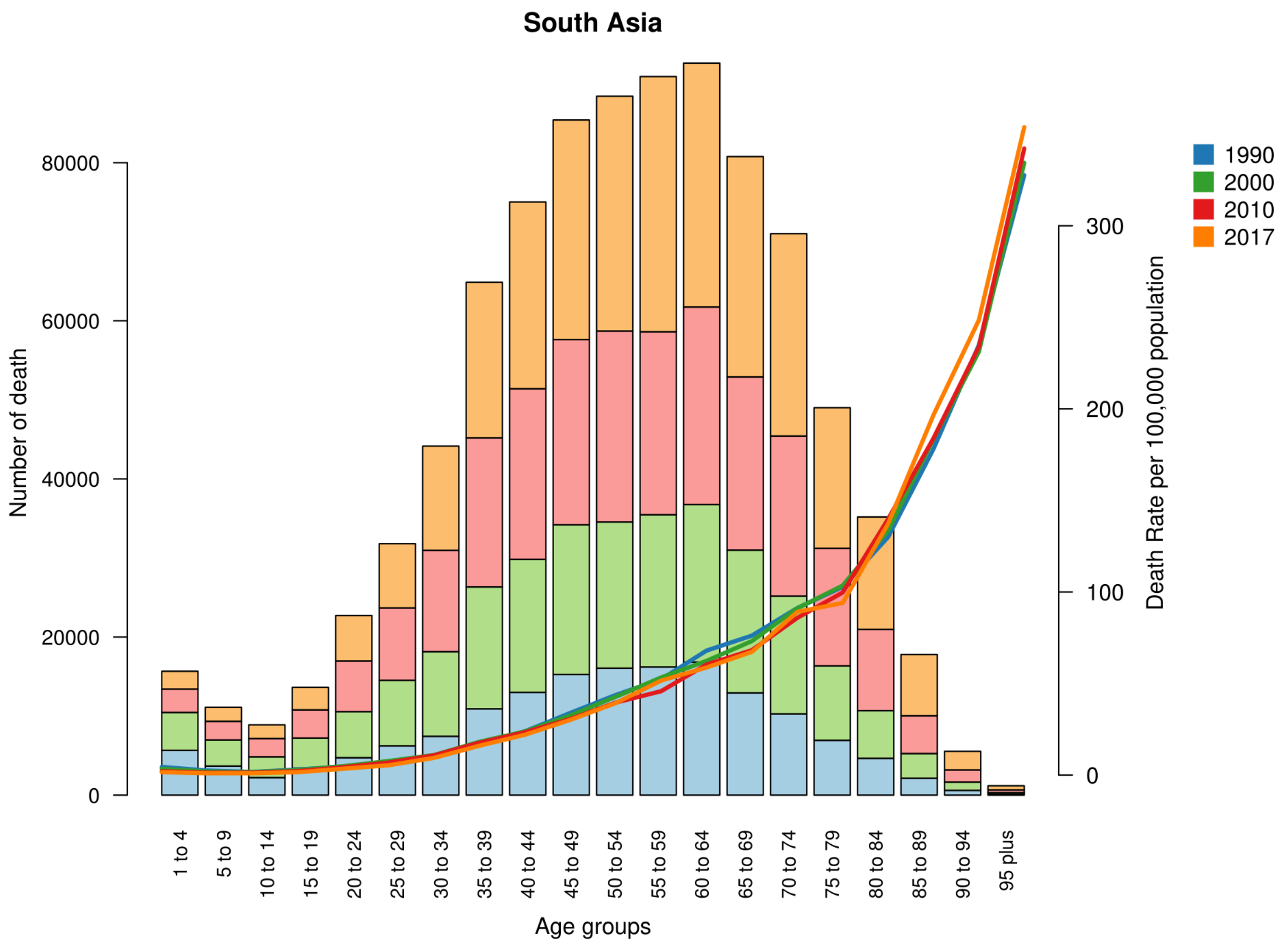
South Asia
Number of death
80000
60000
40000
20000
0
Death Rate per 100,000 population
300
200
100
0
1990
2000
2010
2017
1 to 4
5 to 9
10 to 14
15 to 19
20 to 24
25 to 29
30 to 34
35 to 39
40 to 44
45 to 49
50 to 54
55 to 59
60 to 64
65 to 69
70 to 74
75 to 79
80 to 84
85 to 89
90 to 94
95 plus
Age groups

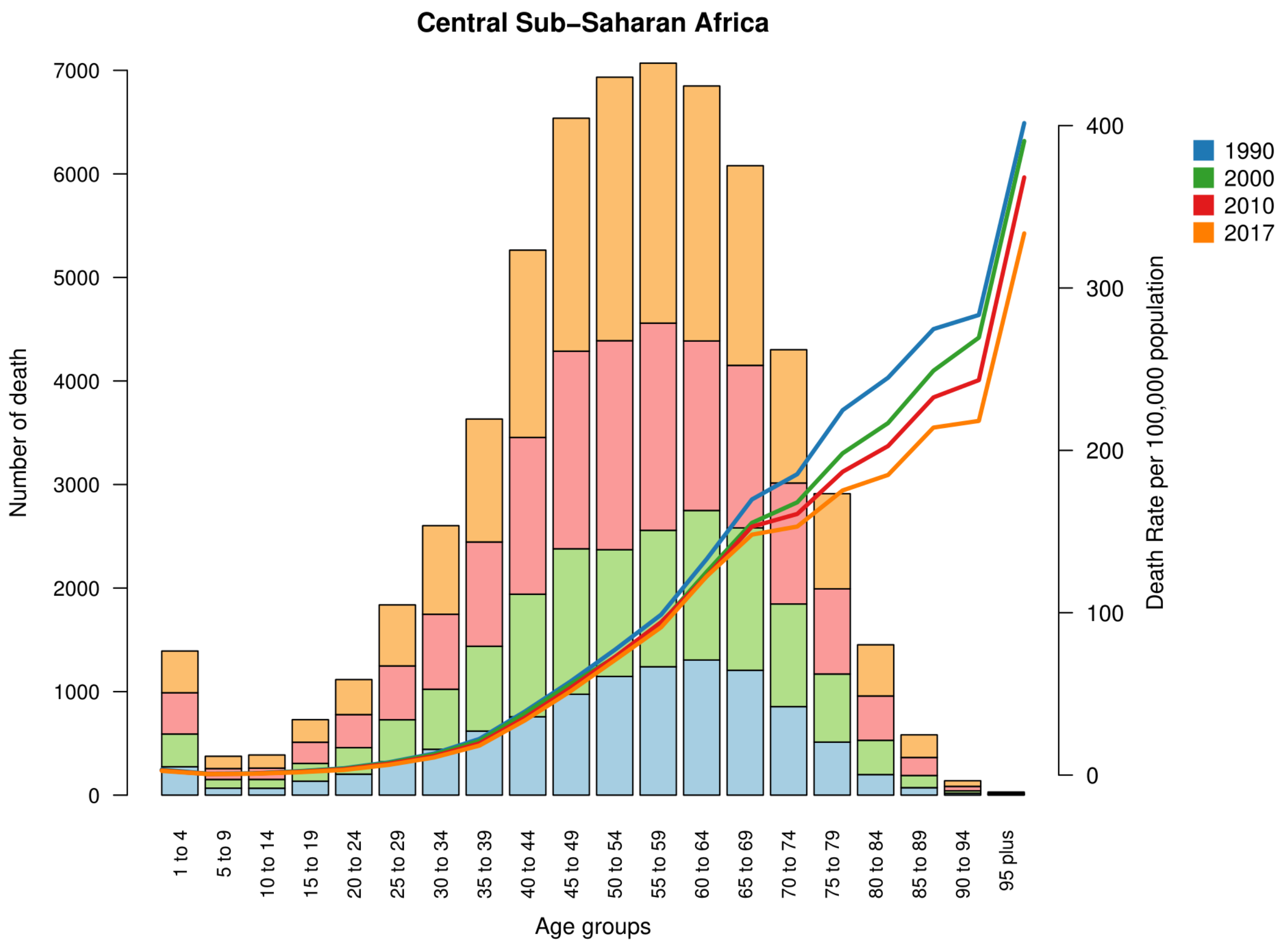
Central Sub–Saharan Africa
Number of death
Death Rate per 100,000 population
Age groups
1990
2000
2010
2017
0
1000
2000
3000
4000
5000
6000
7000
0
100
200
300
400
1 to 4
5 to 9
10 to 14
15 to 19
20 to 24
25 to 29
30 to 34
35 to 39
40 to 44
45 to 49
50 to 54
55 to 59
60 to 64
65 to 69
70 to 74
75 to 79
80 to 84
85 to 89
90 to 94
95 plus

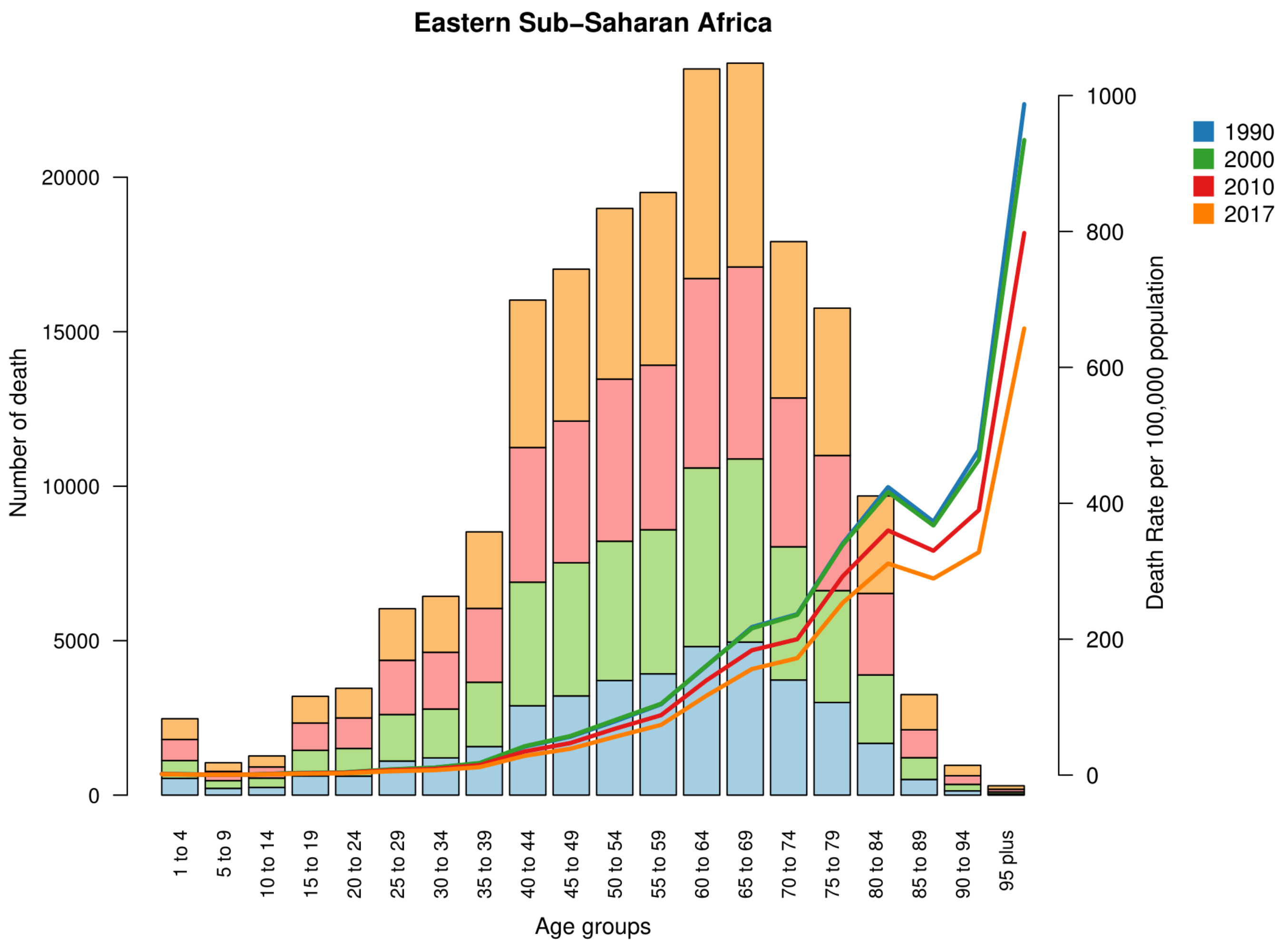

Eastern Sub−Saharan Africa
Number of death
0
5000
10000
15000
20000
Death Rate per 100,000 population
0
200
400
600
800
1000
1990
2000
2010
2017
1 to 4
5 to 9
10 to 14
15 to 19
20 to 24
25 to 29
30 to 34
35 to 39
40 to 44
45 to 49
50 to 54
55 to 59
60 to 64
65 to 69
70 to 74
75 to 79
80 to 84
85 to 89
90 to 94
95 plus
Age groups

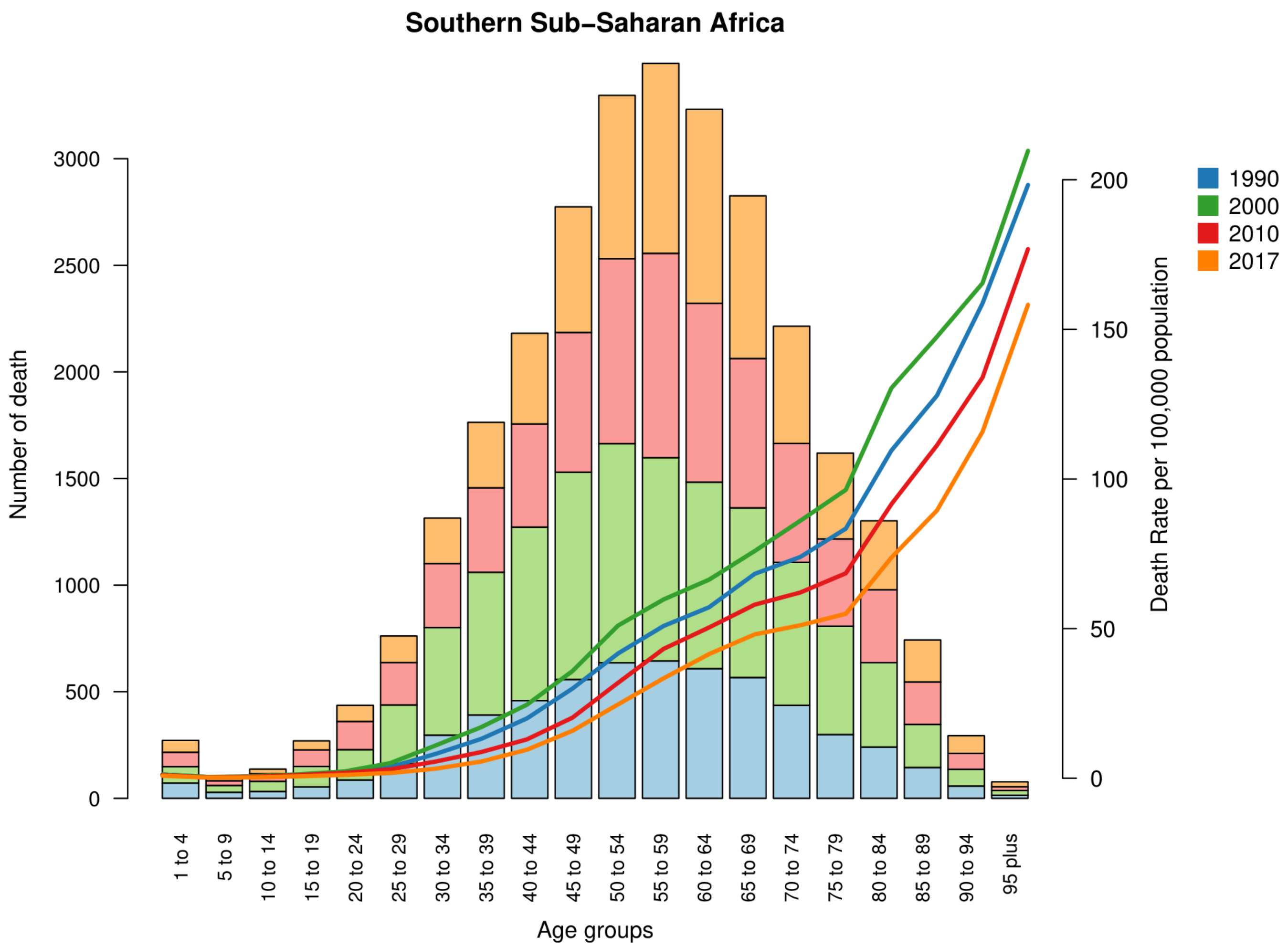

Southern Sub−Saharan Africa
Number of death
3000
2500
2000
1500
1000
500
0
Death Rate per 100,000 population
200
150
100
50
0
1990
2000
2010
2017
1 to 4
5 to 9
10 to 14
15 to 19
20 to 24
25 to 29
30 to 34
35 to 39
40 to 44
45 to 49
50 to 54
55 to 59
60 to 64
65 to 69
70 to 74
75 to 79
80 to 84
85 to 89
90 to 94
95 plus
Age groups

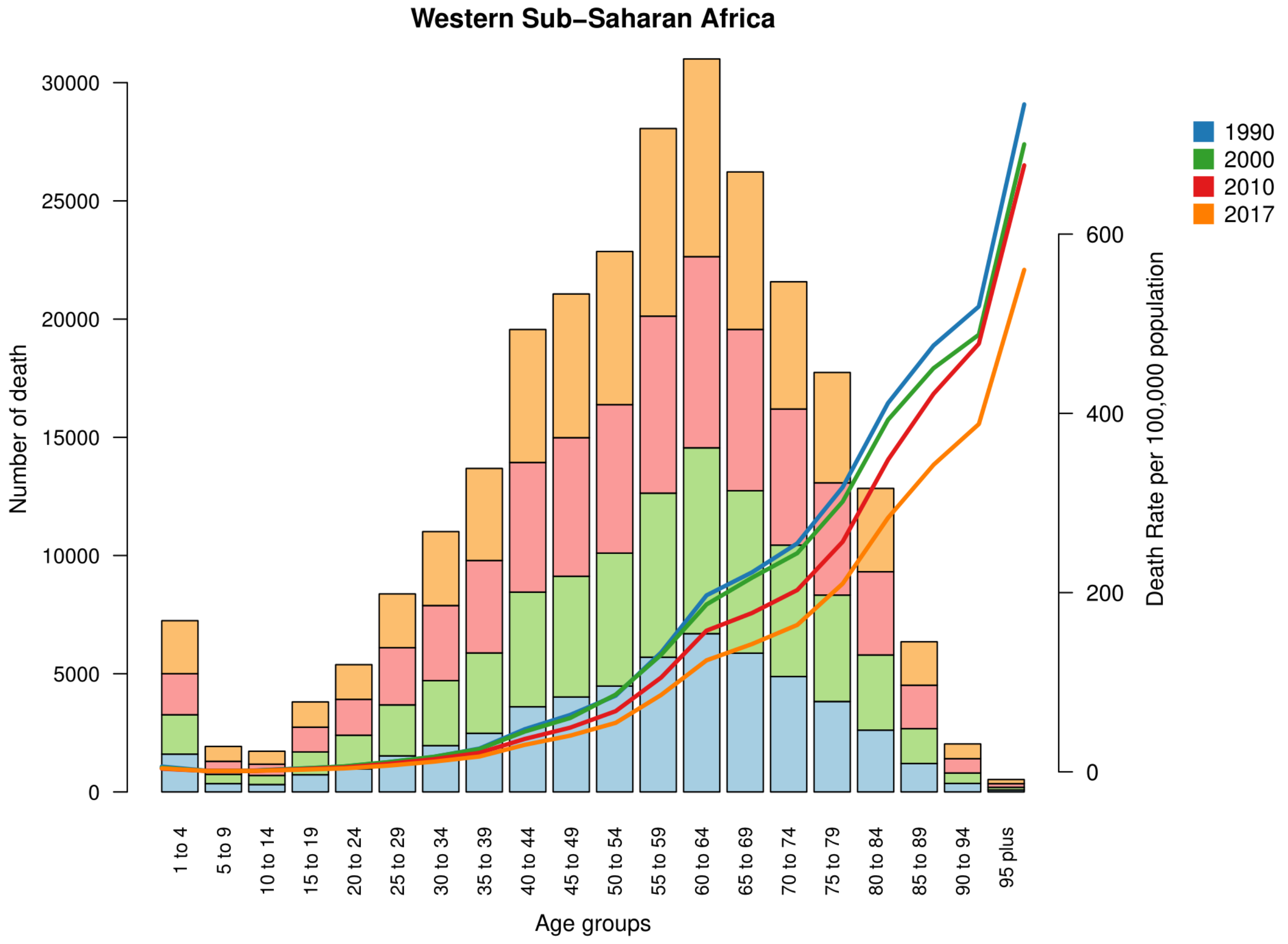
Western Sub−Saharan Africa
Number of death
30000
25000
20000
15000
10000
5000
0
Death Rate per 100,000 population
600
400
200
0
1990
2000
2010
2017
1 to 4
5 to 9
10 to 14
15 to 19
20 to 24
25 to 29
30 to 34
35 to 39
40 to 44
45 to 49
50 to 54
55 to 59
60 to 64
65 to 69
70 to 74
75 to 79
80 to 84
85 to 89
90 to 94
95 plus
Age groups

Appendix Figure 10: **The trend of age-standardised rates of A) deaths, B) DALYs due to cirrhosis across nations in 2017, versus levels of SDI.** DALYs=disability-adjusted life-years. SDI=Socio-demographic Index.

A) Deaths

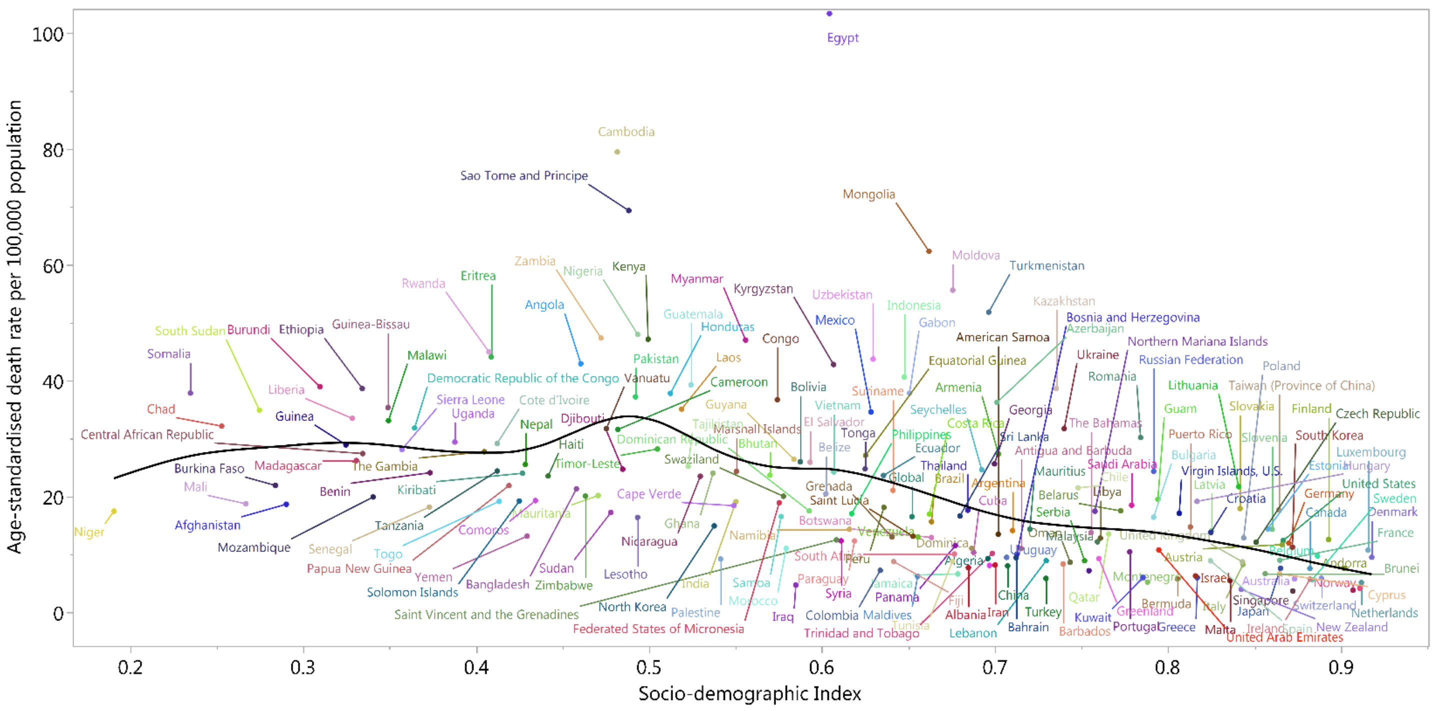

B) DALYs

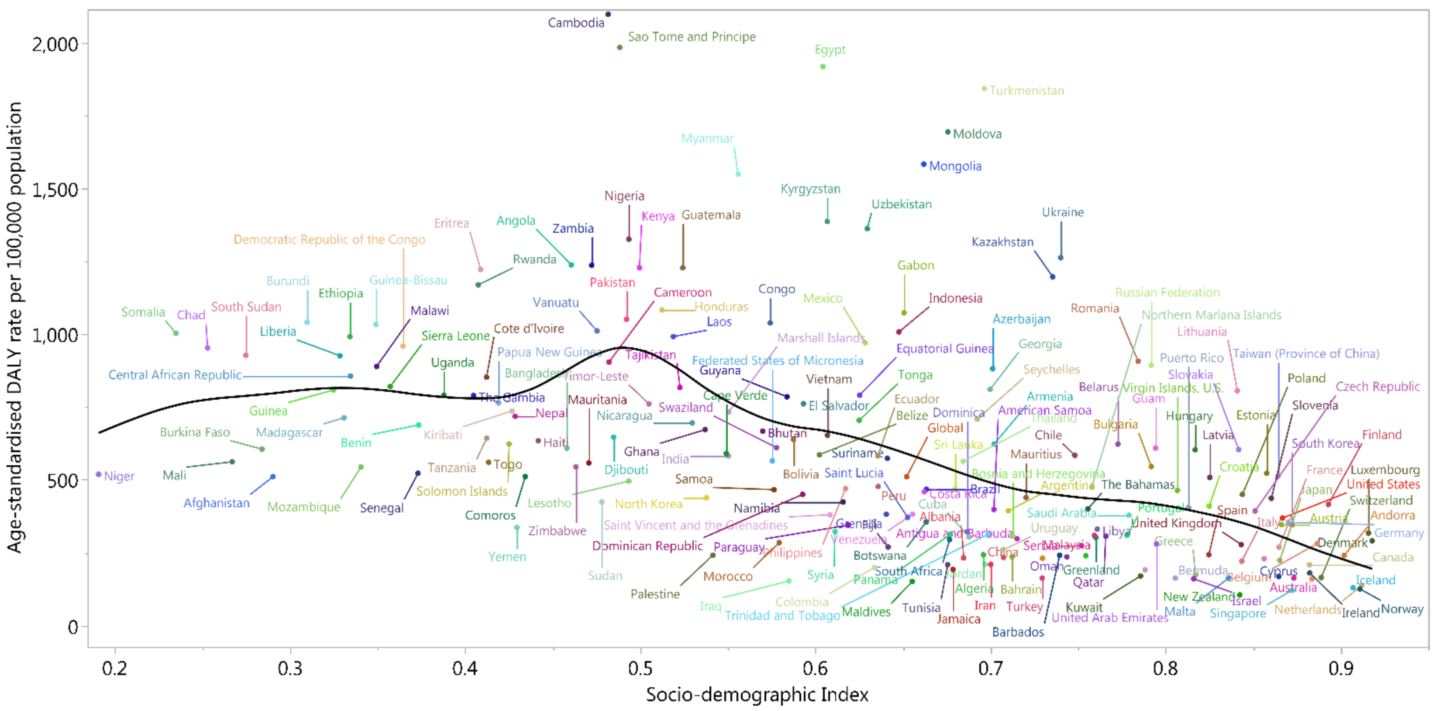

Supplement: Supplementary appendix [file mmc1.pdf]
